# Characterization of the Components and Metabolites of Achyranthes Bidentata in the Plasma and Brain Tissue of Rats Based on Ultrahigh Performance Liquid Chromatography–High-Resolution Mass Spectrometry (UHPLC–HR-MS)

**DOI:** 10.3390/molecules29122840

**Published:** 2024-06-14

**Authors:** Mengting Wu, Peilin Yang, Jianying Wang, Ruoyan Yang, Yingyuan Chen, Kun Liu, Ying Yuan, Lei Zhang

**Affiliations:** 1 School of Pharmacy, Shanghai University of Traditional Chinese Medicine, Shanghai 201203, China; w18156416733@163.com (M.W.); 15688021100@163.com (R.Y.); cyy1634239065@163.com (Y.C.); 15221365682@163.com (K.L.); 2 Shanghai Innovation Center of TCM Health Service, Shanghai University of Traditional Chinese Medicine, Shanghai 201203, China; yangpeilin@shutcm.edu.cn (P.Y.); wjy8310@163.com (J.W.)

**Keywords:** *Achyranthes bidentata*, chemical constituents, prototype components and metabolites, UHPLC–Q Exactive Orbitrap–HRMS

## Abstract

Background: Achyranthes bidentata (AR) is a traditional Chinese herb used for the treatment of hypertension and cerebral ischemia, but its pharmacological effects are not known. Aim of study: We aimed to detect and accurately identify the components and metabolites of AR in the plasma and brain tissue of Sprague Dawley rats. Methods: We employed ultrahigh performance liquid chromatography–high-resolution mass spectrometry (UHPLC–HR-MS) to detect AR components in the plasma and brain tissue of rats. The absorption and metabolites in the plasma and brain tissue of normal control rats and rats that underwent middle cerebral artery occlusion (MCAO) were characterized and compared. Results: A total of 281 compounds, including alkaloids, flavonoids, terpenoids, phenylpropanes, sugars and glycosides, steroids, triterpenes, amino acids, and peptides, was identified in samples of Achyranthes bidentata (TCM-AR). Four types of absorbable prototype components and 48 kinds of metabolites were identified in rats in the normal control plasma group which were given AR (AR plasma group), and five kinds of metabolites were identified in rats of the normal control brain tissue group which were given AR (AR brain group). Three absorbed prototype components and 13 metabolites were identified in the plasma of rats which underwent MCAO and were given AR (MCAO + AR plasma group). Six absorbed prototype components and two metabolites were identified in the brain tissue of rats who underwent MCAO and were administered AR (MCAO + AR brain group). These results showed that, after the oral administration of AR, the number of identified components in plasma was more than that in brain tissue. The number of prototype components in the AR plasma group was higher than that in the MCAO + AR plasma group, which may indicate that metabolite absorption in rats undergoing MCAO was worse. The number of prototype components in the MCAO + AR brain group was higher than that in the AR brain group, indicating that the blood–brain barrier was destroyed after MCAO, resulting in more compounds entering brain tissue. Conclusions: UHPLC–HR-MS was used to rapidly analyze the components and metabolites of AR in the blood and brain of rats under normal and pathologic conditions, and to comprehensively characterize the components of TCM-AR. We also analyzed and compared the absorbable components and metabolites of normal rats under cerebral ischemia-reperfusion injury to explore the potential mechanism of action. This method could be applied to various Chinese herbs and disease models, which could promote TCM modernization.

## 1. Introduction

Stroke is the second leading cause of death and the third leading cause of disability worldwide, affecting one in four people [1]. Stroke includes ischemic diseases and hemorrhagic cerebrovascular diseases, which are associated with high morbidity, disability, and mortality [2]. Among them, ischemic stroke accounts for about 75–85% of the total number of patients with stroke [3]. The incidence of ischemic stroke is increasing year-by-year, and up to 60–80% of patients who have suffered a stroke will die [4]. Even if patients survive, they have serious sequelae, which seriously affects their quality of life [5].

In ischemic stroke, the cerebral blood supply is insufficient due to the stenosis/occlusion of the arteries which serve it, resulting in the necrosis of brain tissue. If ischemic brain tissue receives a blood supply again, ischemic injury will be aggravated, and even more serious consequences (e.g., neuron death, fatal brain edema) may result. This phenomenon is called “cerebral ischemia-reperfusion injury” (CIRI) [6]. The cause of CIRI appears to be the sudden restoration of a blood supply of cerebral vessels in the ischemic state, which leads to a series of pathologic changes in the brain.

*Achyranthes bidentata* (AR) is the dried root of plants from the Amaranthaceae family. In traditional Chinese medicine (TCM) theory, AR has the role of tonifying the liver and kidney, promoting blood circulation, and removing blood stasis [7]. It is often used in TCM formulations to treat hypertension and stroke [8,9]. Ecdysterone and triterpenoids are the main components of AR species [10,11]. Various active ingredients in AR have been shown to have antioxidant, anti-inflammatory, and neuroprotective effects [12].

A TCM formulation is a complex mixture of many ingredients [13]. These components (and their metabolites) can interact in complex ways with various proteins in the body [14]. Most ingredients of TCM formulations must reach specific concentrations after being metabolized in the blood after oral administration to be efficacious [15]. Ultrahigh-performance liquid chromatography (UHPLC) combined with high-resolution mass spectrometry (HR-MS) is used to characterize the metabolites of biological samples based on accurate mass numbers and MS/MS(Mass Spectrometry) data [16,17]. The pathologic state of stroke can further affect the absorption and distribution of drugs.

Herein, we identified the prototype and metabolites of AR absorption in plasma and brain tissue of Sprague Dawley (SD) rats under normal and pathologic conditions. Based on our comprehensive characterization and analyses, this study could aid further research on the targets and mechanism of action of AR for CIRI treatment.

## 2. Results

### 2.1. Model Verification

**Figure 1:**
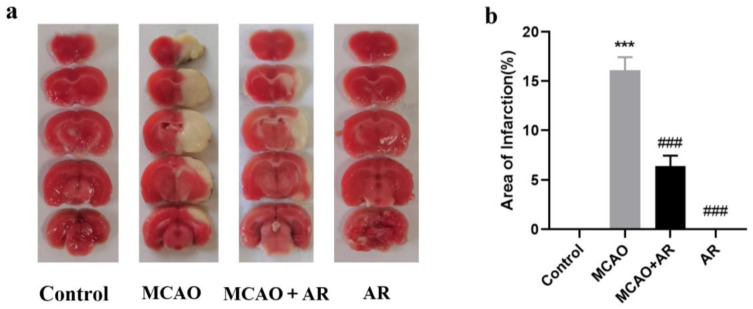
(**a**) TTC staining of rat brain tissue. (**b**) Area of the cerebral infarct. Control: sham operation group of rats; MCAO: model group of rats; MCAO + AR: model group after administration of *Achyranthes* bidentata of rats; AR: Achyranthes bidentata group of rats. (*** *p* < 0.001 vs. control; ### *p* < 0.001 vs. MD).

The MCAO method was used to model rats. Two hours after ischemia, 2,3,5-triphenyltetrazolium chloride (TTC) staining was carried out on the brain tissues of three rats in a blank group, three rats in a model group, and three rats in the drug administration group (Figure 1). After TTC staining, the brain tissue of rats in the blank control group was red. Many white infarcts appeared on the right side of the brain tissue of rats immediately after modeling. Hence, modeling was successful.

### 2.2. Component Identification and Analyses of TCM-AR

The AR was processed to obtain the samples of *Achyranthes bidentata* tested on the machine, namely TCM-AR. UPLC–MS/MS was used to detect the components of TCM-AR. MS data were processed and analyzed according to the screening platform of Progenesis QI v3.0, combined with the LuMet-CM database and HERB database. Based on multidimensional matching (retention time, precise mass number, secondary fragments, and isotope distribution), the compounds and structures of TCM-AR were identified accurately and characterized using a literature review [10,18,19].

**Figure 2:**
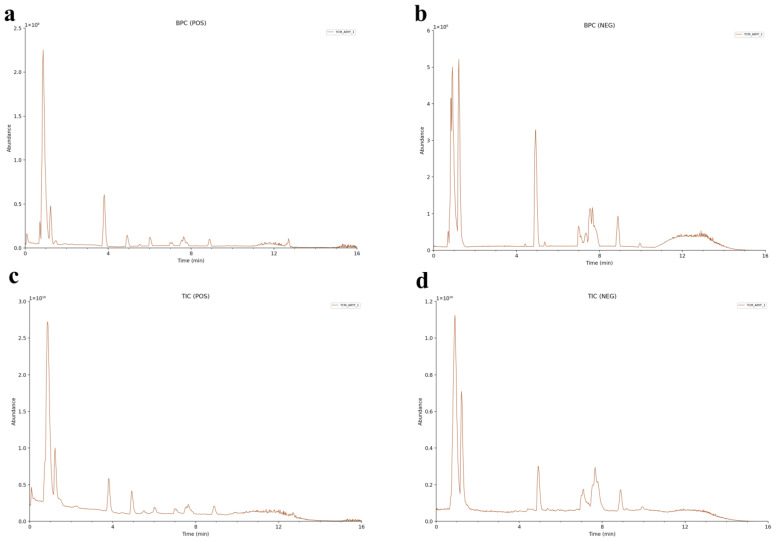
Base Peak Chromatogram (BPC) and Total Ion Chromatogram (TIC) of *Achyranthes bidentata*, obtained using UHPLC–HR-MS. (**a**,**c**) positive ion mode. (**b**,**d**) Negative ion mode.

A total of 281 compounds (142 compounds in positive ion mode and 139 compounds in negative ion mode) from *Achyranthes bidentata* for machine testing (TCM-AR) was identified. These were as follows: 43 sugars and glycosides; 40 amino acids and peptides; 31 alkaloids; 31 terpenoids; 30 phenylpropanes; 21 flavonoids; 21 fatty acylates; 12 organic acids and their derivatives; 11 steroids; 5 quinones; 5 phenols; 8 carboxylic acids and their derivatives; 3 nucleotides and their derivatives; 3 pyridines and their derivatives; and 17 other compounds (Table 1, Supplementary Table S1). Table 1 shows the compound adduction ion form, retention time, theoretical plastic–nucleus ratio, measured plastic–nucleus ratio, molecular weight deviation, characteristic fragment ion, molecular formula, name, peak area ratio, and InChIKey (International Chemical Identifier Key). The Extracted Ion Chromatogram (EIC)s and MS/MS spectra of identified compounds in comparison with databases are shown in Supplementary Figure S1. Figure 2 shows the UHPLC–MS/MS Base Peak Chromatogram (BPC) and Total Ion Chromatogram (TIC) of TCM-AR in positive and negative ion modes.

**Figure 3:**
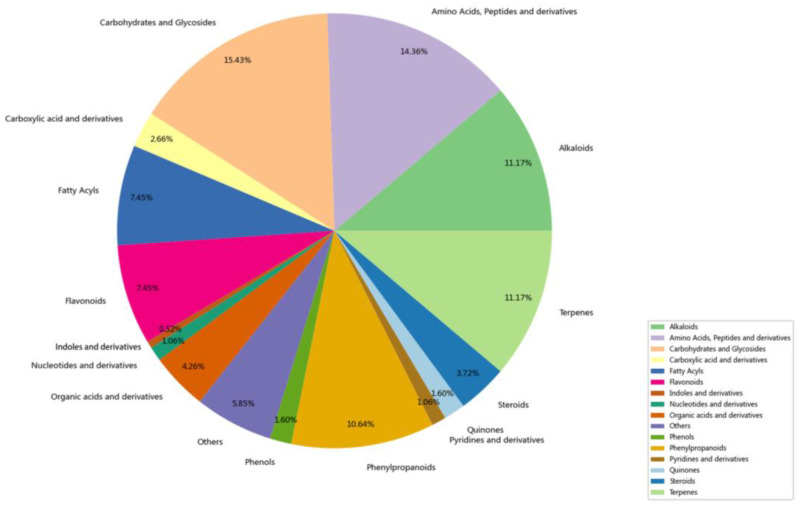
Quantities of the components in *Achyranthes bidentata*.

**Figure 4:**
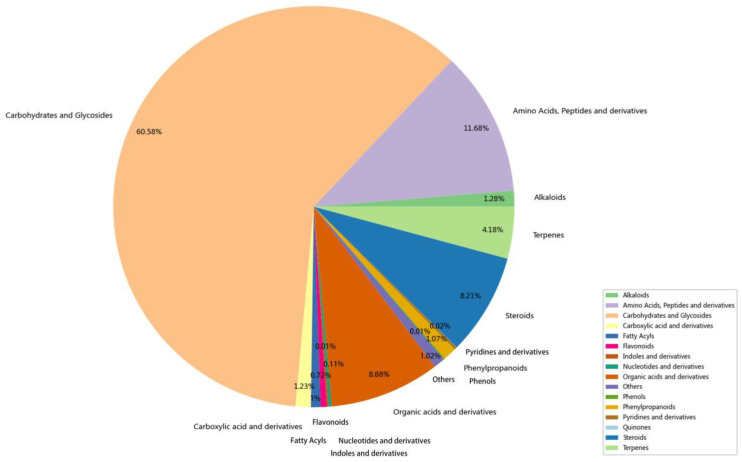
Contents of the components in *Achyranthes bidentata*.

The quantitative classification and content classification of the compounds identified from TCM-AR are shown in Figure 3 and Figure 4. The top five quantities in TCM-AR were sugar and glycosides (15.43%), amino acids and peptides (14.36%), terpenoids (11.17%), alkaloids (11.17%) and phenylc (10.64%). The top 5 contents of TCM-AR were sugar and glycosides (60.58%), amino acids and polypeptides (11.68%), organic acids and their derivatives (8.88%), steroids (8.21%) and terpenes (4.18%).

#### 2.2.1. Sugars and Glycosides

**Figure 5:**
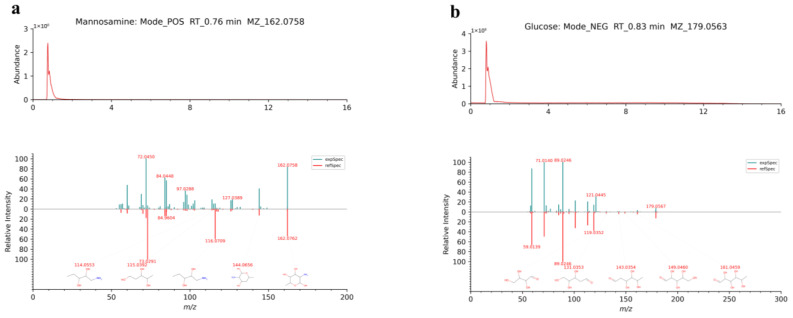

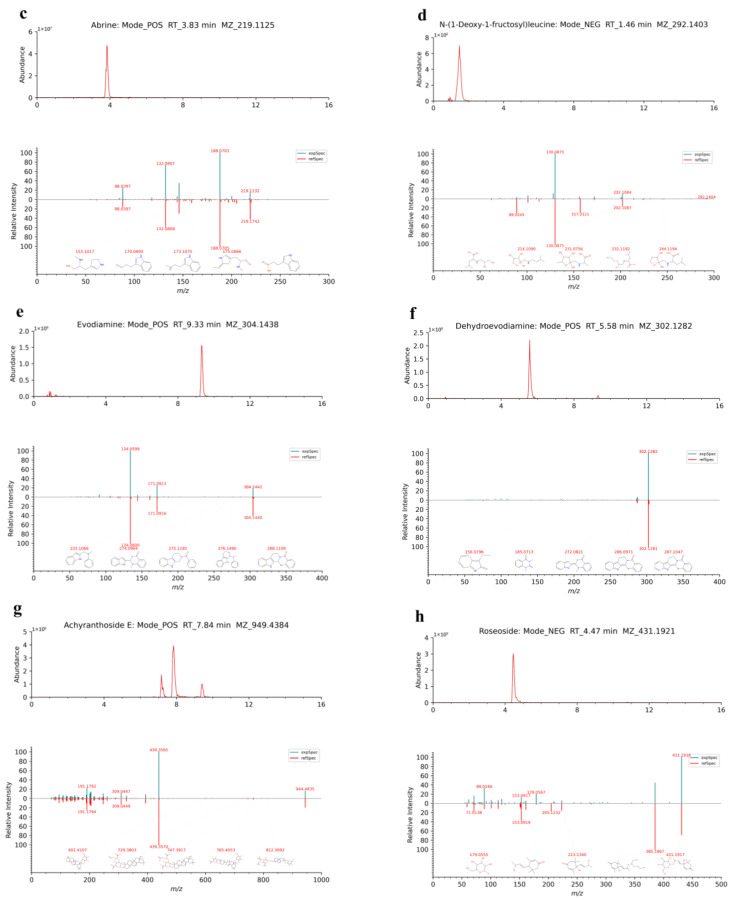

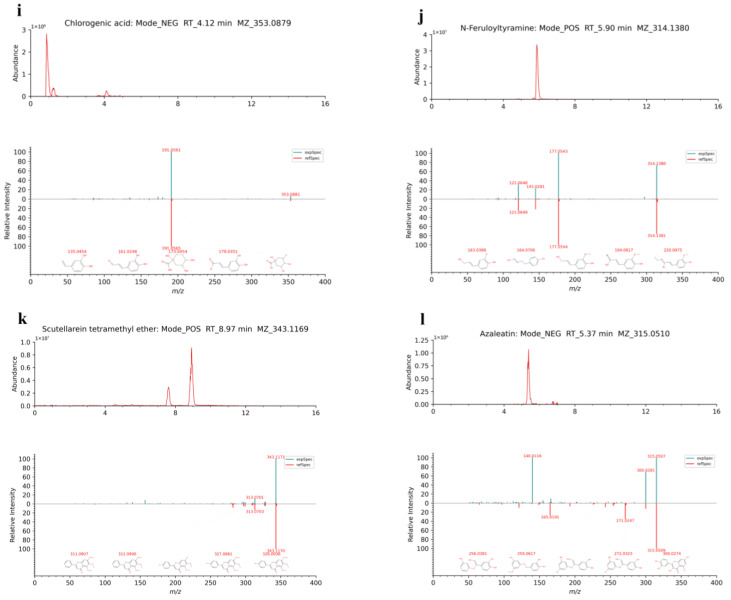
EICs of each chemical and MS/MS spectrum compared with databases. (**a**) Mannosamine. (**b**) Glucose. (**c**) Abrine. (**d**) *N-*(1-Deoxy-1-fructosyl)leucine. (**e**) Evodiamine. (**f**) Dehydroevodiamine. (**g**) Achyranthoside E. (**h**) Roseoside. (**i**) Chlorogenic acid. (**j**) *N-*Feruloyltyramine. (**k**) Scutellarein tetramethyl ether. (**l**) Azaleatin.

Through databases (HERB, LuMet-CM) and the literature, 43 types of sugars and glycosides were identified from TCM-AR. Among them, arabinose, glucose, turanose, stachyose, isomaltotetraose, 1-*β*-d-Arabinofuranosyluracil, maltopentaose, manninotriose mannosamine, palatinose, nystose, 5-*o*-Methylvisammioside, Momordin IIc, 1,4-d-Gulonolactone, l-Deoxynojirimycin, levoglucosan, macrozamin, maltohexaose, salviaflaside, prim-o glucosyl cimifugin, sibiricose a1, syringin, sequoyitol, *cis*-Ferulic acid 4-*o*-*β*-d-glucopyranoside, fructo-oligosaccharide dp7/gf6, cyclic *n*-acetyl-d-mannosamine, erythorbic acid, and mannoheptulose were obtained using the LuMet-CM database. Others were identified by the HERB database. Figure 5a,b show the EICs of mannosamine and glucose and their MS/MS spectra, in comparison with the HERB database, respectively.

Taking compound number 55 as an example, under positive ion mode and a retention time of 0.76 min, the excimer ion of the compound was *m*/*z* 162.0758 [M+H-H_2_O, M+H, M+K, M+Na]^+^. The molecular weights of secondary fragments were 101.0237, 102.0552, 103.0393, 114.0550, 115.0391, 116.0708, 126.0549, 127.0389, 144.0652, and 162.0758, and the chemical formula was C_6_H_13_NO_5_. After comparison with the LuMet-CM database, the compound was identified as mannosamine, and its EIC, molecular weight of secondary fragments, and structure are shown in Figure 5a. Other compounds were identified in a similar way, and EICs and detailed information are shown in Table S1 and Figure S2.

#### 2.2.2. Amino Acids and Peptides

Forty amino acids and peptides were identified in TCM-AR. Abrine, gamma-Glu-Phe, gamma-Glutamylleucine, pyroglutamic acid, l-Glutamine, l-Histidine, l-Leucine, l-Lysine, l-Methionine, l-Phenylalanine, *N-*(1-deoxy-1-fructosyl)leucine, *N-*(1-Deoxy-1)-fructosyl)phenylalanine, *N-*Acetyl-l-alanine, *N-*Acetyl-l-phenylalanine, l-Arginine, l-Glutamic acid, pantothenic acid, *N*6,*N*6,*N*6-trimethyl-l-Lysine, *N-*Acetyltryptophan, *N-*Acetylserine, *N-*Acetylleucine, Prolylglycine, *N*6-acetyllysine, 4-Hydroxyisoleucine, 1-Methyl-l-tryptophan, 6-Aminocaproic acid, and Leu-Leu were identified by the LuMet-CM database, and the other compounds were identified by the HERB database. Figure 5c,d show the EICs of abrine and *N-*(1-Deoxy-1-fructosyl)leucine and their MS/MS spectra compared with the HERB database, respectively.

Taking compound number 1 as an example, when the retention time was 3.83 min, the excimer ion peak *m*/*z* 219.1125 [M+H]^+^ was measured under the positive ion mode, and the predicted molecular formula was C_12_H_14_N_2_O_2_. The main fragment ions were at *m/z* values of 88.0397, 132.0807, and 144.0807, and the molecular peak was 219.1125 [M+H]^+^. After comparison with the database, *m/z* values of 146.0598, 188.0703, 200.1278, and 219.113 suggested that the compound was abrine. The EIC, molecular weight of secondary fragments, and structure are shown in Figure 5c. The EICs of other compounds and MS/MS spectra and detailed information in comparison with databases are shown in Table S1 and Figure S2.

#### 2.2.3. Alkaloids

Thirty-one alkaloids were identified in TCM-AR. Evodiamine, dehydroevodiamine, kifunensine, hypaphorine, tropine, Indole-3-methanamine, Methyl 5-hydroxypyridine-2-carboxylate, *N-*Feruloyloctopamine, pellitorine, oxyberberine, nudifloramide, nicotinic acid riboside, and *N-*Formylcytisine were identified using the LuMet-CM database, and erysodienone and evodione were identified using the HERB database. Figure 5e,f show the EICs of evodiamine and dehydroevodiamine and their MS/MS spectra compared with databases, respectively.

Taking compound number 4 as an example, at a retention time of 9.33 min, the peak of the excimer ion was at *m/z* 304.1438 [M+H]^+^, the molecular formula was C_19_H_17_N_30_, and the main fragment ions were at *m/z* values of 134.0599, 171.0913, and 304.1442, according to the literature [20]. The molecular weight and structure of the secondary fragments identified as evodiarine are shown in Figure 5e.

#### 2.2.4. Terpenoids

Thirty-one terpenoids were identified in TCM-AR. These included arjunolic acid, roseoside, achyranthoside E, pedunculoside, paeoniflorin, Bayogenin-3-*O*-[*β*-d-galactose-(1→3)-*β*-d-Glucuronic acid-28-*O*-*β*-d-glucopyranoside, 4,10-epizedoarondiol, Oleoside 11-methyl ester, 10-Hydroxymajoroside, asperulosidic acid, 6’-*O*-*β*-d-Glucosylgentiopicroside, araloside A, tarasaponin VI, l-Borneol, kauran-16,17-diol, calenduloside E, geniposide, ginsenoside Ro, gentiopicroside, Chikusetsusaponin Iva, and other compounds. Figure 5g,h show the EICs of achyranthoside E and roseoside and their MS/MS spectra compared with the databases, respectively.

Using compound number 60 as an example, at a retention time of 7.84 min, the excimer ion peak was at *m/z* 949.4384 [M+NH_4_, M+Na]^+^, and the molecular formula was predicted to be C_46_H_70_O_19_. The molecular weights of secondary fragment ions were 189.1633, 191.1792, 201.1634, 203.1791, 205.1948, 247.1689, 309.0447, 393.3502, 439.3565, and 944.4835, which were consistent with the literature [19]. The compound was identified as achyranthoside E, and its EIC, molecular weight of secondary fragments, and annotated structure are shown in Figure 5g.

#### 2.2.5. Phenylpropanoids

Thirty phenylpropanes were identified from TCM-AR. These included chlorogenic acid, polydatin, *N-trans*-sinapoyltyramine, *N-*Feruloyltyramine, pinoresinol diglucoside, 3,4-Dimethoxycinnamyl alcohol, osmundacetone, praeruptorin B, *N-trans*-caffeoyltyramine, 4-Feruloylquinic acid, 3-Coumaric acid, alloimperatorin, magnesium lithospermate B, scopoletin, pteryxin, psoralen, sibirioside a, sinapaldehyde, Episyringaresinol 4’-*O*-*β*-d-glncopyranoside, eudesmin, and other compounds. Figure 5i,j show the EICs of chlorogenic acid and *N-*Feruloyltyramine and their MS/MS spectra compared with databases, respectively.

Taking compound No. 20 as an example, the excimer ion peak *m*/*z* 353.0879 [M-H]^−^ in the negative ion mode with the retention time, tR, of 4.12 min may be generated by proton loss, and the ion is further decomposed into ionic fragments *m*/*z* 191.056 [M-H-C_9_H_6_O_3_]^−^, 179.0347 [M-H-C_7_H_10_O_5_]^−^, and 173.0454 [M-H-C_9_H_6_O_3_-H_2_O]^−^ [21], and the molecular weight of the secondary fragments included *m/z* 191.0561 and *m/z* 353.0881, which were preliminarily identified as chlorogenic acid. The EIC diagram, the molecular weight of the secondary fragments, and the annotated structure diagram are shown in Figure 5i.

#### 2.2.6. Flavonoids

Twenty-one flavonoids were identified from TCM-AR. D-Mannose, scutellarein tetramethyl ether, azaleatin, nobiletin, kakkalide, brassidin, butin, and iristectorigenin were detected using the LuMet-CM database. Chrysin 6-c-Glucoside 8-C-arabinoside, quercetin, quercetagetin 3,5,6,7,3’,4’-hexamethyl ether, antgeretin, and dracorhodin compounds such as perchloric acid and farnesylacetone were detected in the HERB database. Figure 5k,l show the EICs of scutellarein tetramethyl ether and azaleatin and their MS/MS spectra compared with databases, respectively.

Taking compound number 34 as an example, at a retention time of 8.97 min, the excimer ion peak was *m/z* 343.1169 [M+H, M+Na]^+^ in positive ion mode, the molecular formula was C_19_H_18_O_6_, and the main ion fragments were at *m/z* values of 157.0128, 313.0701, and 343.1171. The compound was inferred to be scutellarein tetramethyl ether after database comparison. The EIC, molecular weight of secondary fragments, and structure of scutellarein tetramethyl ether are shown in Figure 5k.

### 2.3. Analyses of AR Components Passing into Plasma and Brain after Administration

Rat blood samples were collected at different periods after the oral administration of AR for 3 days. After static centrifugation, we mixed the plasma samples collected at all time points to obtain a dose of plasma for analysis. To characterize the absorbable components of AR in rat plasma and brain tissue, we first established a UHPLC–HR-MS method to screen these components. The control plasma group and TCM-AR group were used as negative and positive controls, respectively.

**Figure 6:**
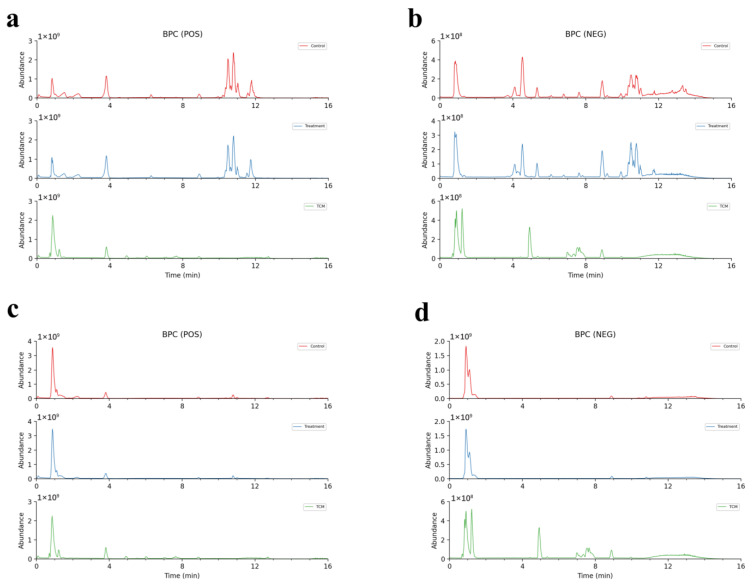
Base Peak Chromatogram (BPC) of *Achyranthes bidentata* in plasma passing into the brain according to UHPLC–HR-MS. Control (control group); treatment (AR group); TCM (TCM-AR group). (**a**) Positive ion mode for the chromatogram of *Achyranthes bidentata* in plasma. (**b**) Negative ion mode for the chromatogram of *Achyranthes bidentata* in plasma. (**c**) Positive ion mode for the chromatogram of *Achyranthes bidentata* in brain tissue. (**d**) Negative ion mode for the chromatogram of *Achyranthes bidentata* in brain tissue.

**Figure 7:**
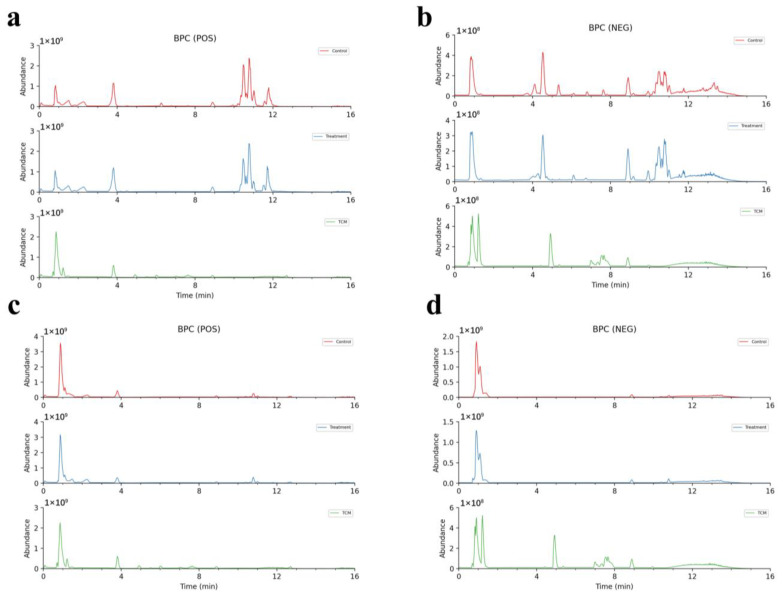
Base peak intensity (BPC) in the chromatogram of *Achyranthes bidentata* in plasma passing into the brain according to UHPLC–HR-MS. Control (control group); treatment (MCAO+AR group); TCM (TCM-AR group). (**a**) Positive ion mode for the chromatogram of *Achyranthes bidentata* in plasma. (**b**) Negative ion mode for the chromatogram of *Achyranthes bidentata* in plasma. (**c**) Positive ion mode for the chromatogram of *Achyranthes bidentata* in brain tissue. (**d**) Negative ion mode for the chromatogram of *Achyranthes bidentata* in brain tissue.

The extracted ion peak appeared in the plasma group containing AR (AR plasma group), in the plasma group for rats that underwent MCAO + AR (MCAO + AR plasma group), and in the TCM-AR group, but did not appear in the control plasma group. This was identified as the prototype component of absorption. Extracted ion peaks were detected in the AR plasma group, but not in the control plasma group or in TCM-AR, and were determined to be metabolites. Based on BPC (Figure 6a,b and Figure 7a,b), total ion chromatograms (TICs) are shown in Supplementary Figure S2. Compared with TCM-AR, 52 absorption components (four prototype components and 48 metabolites) were identified in the AR plasma group. Compared with TCM-AR, we identified 16 absorption components in the MCAO + AR plasma group (three prototype components and 13 metabolites). Compared with TCM-AR, the 52 absorptive components in the AR plasma group were 36 more than the 16 absorptive components in the MCAO + AR plasma group. We speculated that in CIRI, the metabolism of rats was disturbed, which led to a reduction in drug-absorption efficiency and a decrease in the number of absorbed components.

The brain tissues of rats were collected 1.5 h after the final administration of AR 3 days after oral administration. The extracted ion peak appeared in the brain tissue group given AR (AR brain group), the brain tissue group given AR after MCAO (MCAO+AR brain group), and the TCM-AR group, but did not appear in the control brain tissue group, which was identified as the prototype component. Extracted ion peaks were detected in the AR brain group and the MCAO + AR brain group, but not in the control brain tissue group or in the TCM-AR group, and were determined to be metabolites. Based on BPC (Figure 6c,d and Figure 7c,d) and TICs (Supplementary Figure S2), compared with TCM-AR, five absorption components were identified in the AR brain group, five of which were metabolites. Compared with TCM-AR, we identified eight absorption components in the MCAO+AR brain group (six prototype components and two metabolites). Compared with TCM-AR, the eight absorbable components in the MCAO+AR brain group were three more than the five absorbable components in the brain tissue of the AR brain group. The main function of the blood–brain barrier (BBB) is to maintain the homeostasis of the brain, protect the brain from potential endogenous/exogenous injuries, and inhibit pathogens and toxic compounds from entering the brain [22,23]. If the brain is damaged, the BBB breaks down and more compounds enter the brain tissue. Therefore, it is speculated that, after the blood–brain barrier is damaged, more AR absorption components enter the brain tissue, which is consistent with the above-mentioned MCAO+AR group, which absorbed more components than the AR group.

#### 2.3.1. Analyses of the Prototype Components of AR into Plasma and Brain

According to the EICs and MS/MS spectra of reference compounds, compared with TCM-AR, four prototypes were identified in the AR plasma group and three prototypes were identified in the MCAO + AR plasma group as absorbable components. Compared with TCM-AR, no prototype components were identified in the AR brain group, whereas six prototypes were identified in the MCAO + AR brain group as absorbable components, and their absorbable components are shown in Table 2 (serial numbers 1–4 are the prototype absorbable components identified in the AR plasma group, 5–7 are the prototype absorbable components identified in the MCAO + AR plasma group, and 8–13 are the prototype absorbable components identified in the MCAO + AR brain group). The EICs of prototype component compounds and MS/MS spectra of reference compounds are shown in Supplementary Figure S3. Table 2 shows the form, retention time, theoretical mass–charge ratio, measured mass–charge ratio, molecular weight deviation, characteristic fragment ion, molecular formula, name, peak area ratio, and InChIKey. The identified components had an error of <5 ppm, in which very little of the prototype component was absorbed and most of the metabolites were metabolized.

#### 2.3.2. Analyses of the Metabolites of AR in the Plasma and Brain

According to the EICs and MS/MS spectra of reference compounds, 48 metabolites were identified in the AR plasma group and 13 metabolites were identified in the MCAO + AR plasma group. Five metabolites were identified in the AR brain group, and two metabolites were identified in the MCAO + AR brain group (Table 3; serial numbers 1–48 are from metabolites identified in the AR plasma group, 49–61 are from metabolites identified in the MCAO + AR plasma group, 62–66 are from metabolites identified in the AR brain group, and 67–68 are from metabolites identified in the MCAO + AR brain group). The specific EICs of these compounds and MS/MS spectra of their reference compounds are shown in Supplementary Figure S4. Table 3 shows the adduct ion form, retention time, measured mass–charge ratio, molecular weight deviation, parent compound, molecular formula, name, and type of metabolite. General prototype products can be excreted directly by metabolites generated in the phase I metabolic reaction, or they can be excreted again through the phase II metabolic reaction [24,25]. The identified components had an error of <5 ppm, in which very little of the prototype component was absorbed and most of the metabolites were metabolized.

Metabolite identification was carried out according to established databases (HERB, LuMet-CM) of metabolites. After sampling, Progenesis QI was used to carry out peak alignment and peak extraction on original data. Then, we searched the databases for analysis, and then determined the metabolites.

**Figure 8:**
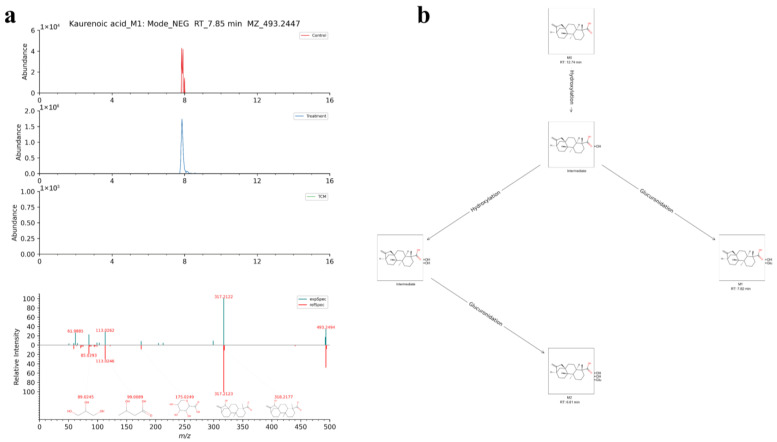
Identification of metabolite number 28. (**a**) EICs of the metabolites and MS/MS spectra of reference compounds. (**b**) Network diagram for metabolites.

Compared with TCM-AR, 39 parent components in the AR plasma group were transformed into 48 metabolites. This process occurred mainly through phase I metabolic reactions such as oxidation, reduction, hydroxylation, carboxylation, and demethylation, as well as phase II metabolic reactions such as methylation and sulfate esterification. In addition, 12 parent components in the MCAO+AR plasma group were transformed into 13 metabolite products. These transformations occurred mainly through hydroxylation, carboxylation, hydrolysis, oxidation, reduction, acetyl oxidation, demethylation, decarboxylation, and other phase I metabolic reactions (e.g., methylation and sulfate esterification), as well as phase II metabolic reactions. Taking metabolite number 28 as an example in negative ion mode at a retention time of 7.85 min, the excimer ion peak was *m/z* 493.2447 [M-H]^−^. After searching a metabolic MS database, the score for the fragment ion was 60.7 and the molecular formula was C_26_H_38_O_9_. We concluded that metabolite number 28 may be derived from the intermediate product of M0 after a hydroxylation phase I reaction, and then a glucuronidation glycolaldehyde phase II reaction. The EICs, MS/MS spectra, and metabolic network diagram of its reference compounds are shown in Figure 8. For the remainder of the metabolites, please refer to Supplementary Figures S4 and S5.

**Figure 9:**
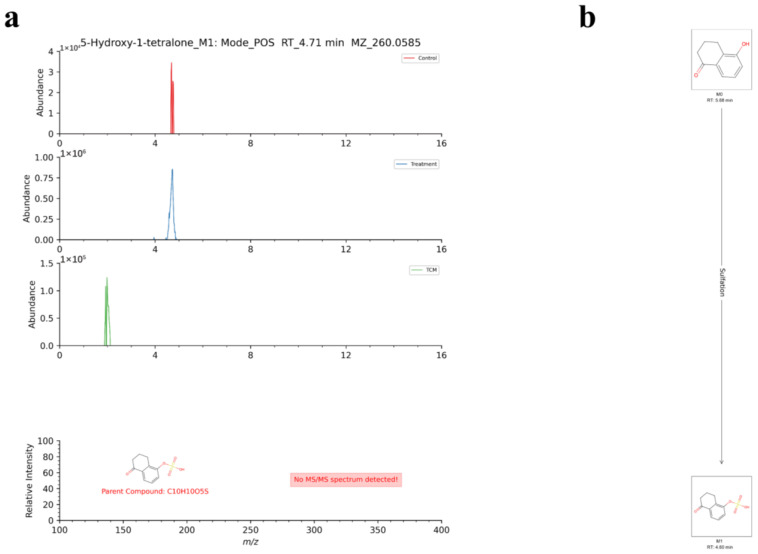
Identification of metabolite number 67. (**a**) EICs of metabolites and MS/MS spectra of reference compounds. (**b**) Network diagram for metabolites.

Compared with TCM-AR, the five parent components in the AR brain group were transformed into five metabolites. These transformations mainly involved phase I metabolic reactions such as hydroxylation and hydrolysis, and phase II metabolic reactions such as glucuronidation glycolaldehyde acidification and methylation. In addition, the transformation of two parent components into two metabolites in the MCAO + AR brain group was analyzed. This transformation included phase-I metabolic reactions such as hydroxylation and phase-II metabolic reactions such as sulfate esterification. Taking metabolite number 68 as an example, in positive ion mode at a retention time of 4.71 min, the quasi-ion peak was *m/z* 260.0585 [M+NH_4_]^+^. A search of a metabolite MS database suggested the molecular formula to be C_10_H_10_O_5_S and the parent compound to be 5-Hydroxy-1-tetralone. We concluded that metabolite number 67 may be produced by M0 in the phase II metabolic reaction of sulfuric acid esterification. The EIC, MS/MS spectra, and metabolic network diagram of its reference compounds are shown in Figure 9.

## 3. Discussion

We employed UHPLC–HR-MS to detect TCM-AR components in the plasma and brain tissue of rats. AR components are complex and varied, and are prone to change in the plasma and brain tissue of the body. Positive and negative ion modes were used to obtain the MS information of compounds in a sample with maximum intensity.

Compared with the reference and database, we identified 281 compounds in TCM-AR, including sugars and glycosides, amino acids and peptides, alkaloids, terpenoids, phenylpropanes, flavonoids, fatty acids, organic acids and their derivatives, steroids, quinones, phenols, carboxylic acids and their derivatives, nucleotides and their derivatives, pyridine and its derivatives, and other compounds. The quantities and classes of various components of TCM-AR were documented. TCM-AR contained high contents of sugars and glycosides, steroids, amino acids, and polypeptides, as well as organic acids and their derivatives; these data are consistent with results from other studies [10]. The active ingredients of AR were identified by prototype absorption in rat plasma and brain tissue after oral administration of AR in normal conditions and in tandem with MCAO, respectively. Four prototype components and 48 metabolites were characterized preliminarily in the AR plasma group. Three prototype components and 13 metabolites were identified or characterized preliminarily in the MCAO + AR plasma group. Five metabolites were identified or characterized preliminarily in the AR brain group. Six prototype components and two metabolites were identified or characterized preliminarily in the MCAO + AR brain group. The main metabolic pathways of absorbable components were oxidation, reduction, hydrolysis, dehydrogenation, dehydration, hydroxylation, carboxylation, demethylation, and other phase I metabolic reactions. Glutathione binding, acetylation, methylation, sulfate esterification, glycination glycosylation, and other phase II reactions were also possible. In addition, many previous studies have shown that many effective components of *Achyranthes bidentata* have neuroprotective [26,27], anti-inflammatory [28], and antioxidant effects [29], which can reduce the damage caused by focal cerebral ischemia-reperfusion [30]. However, the results showed that some compounds of oxidative stress, such as allantoin (No. 8 in Table 2), were found in the brain tissue of rats in MCAO + AR group, indicating that the MCAO model successfully caused oxidative stress; on the other hand, subsequent experiments would further quantitatively analyze whether the change in allantoin content was due to the role of achyranthosa in cerebral ischemia-reperfusion injury.

Absorbable components were characterized or identified in plasma rather than in brain tissue. Rats in the AR plasma group absorbed more components than rats in the MCAO + AR plasma group. The metabolism of animals is disturbed during cerebral ischemia [31,32] and the absorption and the number of absorbed components may be reduced. The compounds hydroxycitric acid, ascorbyl, *N-trans*-sinapoyltyramine-M1, and nuciferine-M1 were identified in the AR plasma group and the MCAO+AR plasma group. Hence, these compounds could be absorbed and metabolized under cerebral ischemia. Among the components that entered the brain, rats in the AR brain group absorbed fewer than the MCAO+AR brain group. During cerebral ischemia-reperfusion, various proinflammatory factors are released by cells to increase BBB destruction, resulting in the entry of neurotoxic substances to worsen brain injury [33]. Cerebral ischemia and hypoxia induce the dissolution of the basal layer of the endothelium and the destruction of tight-junction proteins, followed by an increase in BBB permeability [34], and reperfusion usually leads to more severe BBB damage and aggravated neuron damage [35,36]. Hence, the BBB is broken, allowing more compounds to enter brain tissue. There were more absorbed components in the MCAO+AR brain group than in the AR brain group. Whether this phenomenon is due to the metabolic changes in sugars and other substances in brain tissue caused by CIRI merits further investigation. In addition, the changes in metabolites in blood and brain tissue caused by differences in animal physiological states, rather than the composition differences caused by mass spectrometry detection, need to be confirmed through further study and analysis of the blood and brain composition of model animals (MCAO) and control animals.

## 4. Materials and Methods

### 4.1. Reagents and Materials

*Achyranthes bidentata* (AR) was purchased from Shanghai Kangqiao TCM Decoction Pieces (production lot number: 2107027; Shanghai, China). MS-grade methanol, acetonitrile, and formic acid were purchased from Thermo Fisher Scientific (Waltham, MA, USA). Ultrapure deionized water was sourced from Unique-R20 Water Purification Systems (Xiamen, China). The ultrasound was purchased from Shanghai Kedao Ultrasonic Instrument Co., Ltd. (Shanghai, China). The grinding instrument was purchased from Shanghai Wanbai Biological (Shanghai, China). The anticoagulant tube was purchased from Becton, Dickinson and Company (Shanghai, China). The centrifuge was purchased from Beckman Coulter Ltd. (Waltham, MA, USA).

### 4.2. Preparation of Samples Achyranthes Bidentata (TCM-AR)

We took 100 mg of the radix hyssop of AR and added 1 mL of a methanol–water solution (3:1, *v/v*, containing 2-Chloro-l-phenylalanine (4 μg/mL)). This mixture was dissolved at −80 °C. Next, two small steel balls were added, followed by cooling at −40 °C for 2 min, and then the mixture was placed into a grinding machine (60 Hz, 2 min). Ultrasonic extraction in an ice water bath for 60 min was followed by standing at −40 °C for 30 min. After centrifugation (12,000 rpm, 30 min, 4 °C), the supernatant was diluted 10 times with a methanol–water mixture. Next, the mixture was passed through a 0.22 μm filter, and 200 μL was placed in an injection vial.

Samples of *Achyranthes bidentata* (TCM-AR) were obtained after the above treatment; that is, the positive control group.

### 4.3. Preparation of Reference Substances

The database of reference substances (LuMet-TCM) was established by Luming Biotech Co., Ltd., (Shanghai, China).

### 4.4. Animals

Specific pathogen-free-grade male Sprague Dawley rats (200 ± 20 g) were purchased from Shanghai Sipur-Bikai Experimental Animal (Experimental Animal License Number. SCXK (Shanghai, China) 2023-0009; Shanghai, China) and raised in the Experimental Animal Center of Shanghai University of Traditional Chinese Medicine (Experimental Animal License Number SYXK (Shanghai, China) 2020-0009). This room was kept at 25 °C and a relative humidity of 45~55%. Rats were exposed to a 12 h light–dark cycle and had access to food and water. After adaptive feeding, 24 rats were randomly divided into four groups of six: a control group of rats, a model group of rats (middle cerebral artery occlusion (MCAO) group), an *Achyranthes bidentata* group of rats (AR group) and a model *Achyranthes bidentata* group of rats (MCAO + AR group). In addition, three more models were made and compared with other groups. The AR group and MCAO + AR group were administered (I.G.) 25 g/kg of TCM-AR twice a day for 3 days. Simultaneously, the control group was given the same dose of distilled water. The protocol for animal experiments was approved (PZSHUTCM2303240002) by the Animal Ethics Committee of Shanghai University of Traditional Chinese Medicine (Shanghai, China).

### 4.5. Preparation of Rat Plasma and Brain Tissue

Blood samples were collected from the ocular orbit at 15 and 30 min, 1, 2, and 4 h after the last administration [37] in the AR group and MCAO + AR group, and plasma was collected using an EDTA (Ethylenediaminetetraacetic Acid) anticoagulant tube. After standing at room temperature for a short time, the tube was centrifuged (4000 rpm, 10 min, 4 °C) and the supernatant was removed to obtain plasma. Equal amounts of plasma at different time points were then mixed to obtain mixed plasma samples. Plasma samples were stored at −80 °C. Before loading, plasma (150 μL) was swirled with 450 μL of methanol–acetonitrile (2:1, *v/v*, containing 2-Chloro-l-phenylalanine (4 μg/mL)) for 1 min. After mixing and centrifugation (12,000 rpm, 10 min, 4 °C) and standing at −40 °C for 2 h, 500 μL of the supernatant was placed in a liquid chromatography–mass spectrometry (LC–MS) sample vial and dried. Then, 150 μL of methanol–acetonitrile–water (2:1:1, *v/v/v*) was added, followed by vortex-mixing for 1 min, and ultrasonic agitation for 3 min. After overnight storage at −40 °C and centrifugation (12,000 rpm, 10 min, 4 °C), 100 μL of supernatant was used for HR-MS.

After the last collection of blood, rats were killed. Brain tissue was removed, rinsed with pure water, drained, and stored at −80 °C. After thawing, a 100 mg sample was added to 400 μL of methanol–water (4:1, *v/v*, containing 2-Chloro-l-phenylalanine (4 μg/mL)). Two small steel balls were added, followed by cooling at −40 °C for 2 min. After grinding in a machine (60 Hz, 2 min), ultrasonic extraction was carried out in an ice water bath for 10 min. After standing at −40 °C for 30 min, we centrifuged the sample (12,000 rpm, 10 min, 4 °C). Next, we took 300 μL of the supernatant and placed it in a LC–MS sample vial to dry. Then, 300 μL of methanol–acetonitrile–water (2:1:1, *v/v/v*) was added, followed by vortex-mixing for 1 min, and ultrasonic agitation for 3 min. After overnight storage at −40 °C and centrifugation (12,000 rpm, 10 min, 4 °C), 100 μL of supernatant was used for HR-MS.

### 4.6. Staining with 2,3,5-Triphenyltetrazolium Chloride (TTC)

After the last administration of AR, three rats were selected randomly from each group for deep anesthesia. Their brain tissues were removed, and residual blood was washed out with physiologic (0.9%) saline. Then, the brain tissue was frozen at −20 °C for 20 min, and cut into 2 mm slices with a surgical blade (Shanghai Pudong Jinghuan Medical Products, Shanghai, China). Sections were prepared with 2% TTC (purity > 98.0%; Shanghai yuanye Biotechnology, Shanghai, China) at 37 °C for 30 min. The percentage of tissue affected by cerebral infarction was measured using ImageJ (US National Institutes of Health, Bethesda, MD, USA).

### 4.7. Instruments and Experimental Conditions

We employed a LC–MS system composed of a high-performance liquid chromatograph (ACQUITY UPLC I-Class; Waters, Waltham, MA, USA) and mass spectrometer (Q Exactive Orbitrap™; Thermo Fisher Scientific). The mass spectrometer was equipped with a heated electrospray ionization source. Chromatography was undertaken on an ACQUITY HSS T3 UPLC column (100 mm × 2.1 mm, 1.8 µm) at a flow rate of 0.35 mL/min. The column temperature was 45 °C. An aqueous solution of 0.1% formic acid was phase A and acetonitrile solution was phase B [38]. The gradient elution was as follows: 0–2 min, 5% B; 2–4 min, 5–30% B; 4–8 min, 30–50% B; 8–10 min, 50–80% B; 10–14 min, 80–100% B; 14–15 min, 100% B; 15–15.1 min, 100–5% B; 15.1–16 min, 5% B. The injection volume was 5.0 μL.

MS was carried out on a mass spectrometer (Q Exactive Orbitrap; Thermo Fisher Scientific) equipped with a heated electrospray ionization source. Positive and negative ion scanning modes and data-dependent acquisition (DDA) mode were used for data acquisition. The full scan range was 100–1200 *m/z* for full MS (resolution = 70,000) and MS/MS (resolution = 17,500). The MS parameters of positive ion mode were sheath gas flow = 35 Arb, auxiliary gas flow = 8 Arb, capillary temperature = 320 °C, and auxiliary gas heater temperature = 350 °C. Normalized collision energies were set to 10, 20, and 40 V. The spray voltage was 3.8 kV in positive ion mode and 3.0 kV in negative ion mode.

### 4.8. Data Processing and Analysis

Data preprocessing was undertaken for pattern recognition. Raw data obtained with software based on metabolomics analysis (Progenesis QI v3.0; Nonlinear Dynamics, Newcastle, UK) were used to identify the corrections for baseline filter, peak, integral, alignment, and retention time, as well as peak normalization. Compounds were identified based on precise mass numbers, secondary fragments, and isotopic distribution using the LuMet-CM database and HERB database on 28 September 2023 (http://herb.ac.cn/).

For substances detected qualitatively using Progenesis QI, substances whose total score was ≥40 points were retained as the original components of the TCM formulation. Substances whose fold change was ≥2.0 in the treatment group (administered plasma) and control group (blank plasma) were used as the components entering the plasma and passing into the brain. Substances in positive ion mode and negative ion mode were merged and de-weighted. The total content of the relative peak area of metabolites was set at 100% to obtain the qualitative and quantitative data matrix. This contained all the information extracted from the original data that could be used for subsequent analyses. Extracted ion chromatograms (EICs) and MS/MS spectra with annotations of the structures of secondary fragments were obtained for each identified original TCM formulation and its components entering the plasma and brain. Pie charts were drawn for all identified components of TCM formulations according to their type and quantity.

## 5. Conclusions

UHPLC–HR-MS was used to rapidly analyze the components and metabolites of AR in the plasma and brain of rats under normal and pathologic conditions and to comprehensively characterize the components of TCM-AR, which will be helpful to explore the material basis of pharmacological effects of AR in future. We also analyzed and compared the absorbable components and metabolites of normal rats under CIRI to explore the potential mechanism of action. However, the prototype components and metabolites in rats under normal and case conditions are significantly different, suggesting that cerebral ischemia-reperfusion may cause inflammation, nerve damage, and blood–brain barrier damage, etc. However, only qualitative characterization was performed in this paper, without quantitative characterization, which has certain limitations, and further discussion and verification will be conducted in the future. This method could be applied to various Chinese herbs and disease models, which could promote the modernization of TCM.

## Figures and Tables

**Table 1:** Identification of the chemical composition of TCM-AR based on UHPLC–HR-MS.

NO	Adducts	RT (min)	Theoretical *m*/*z*	*m*/*z*	Mass Error (ppm)	Formula	Metabolites	Ratio of Peak Area %	Fragment Ions	InChIKey
1	[M+H]^+^	3.83	219.1128	219.1125	−1.32	C_12_H_14_N_2_O_2_	Abrine	0.149137	88.0397, 132.0807, 144.0807, 146.0598, 188.0703, 200.1278, 219.1132	CZCIKBSVHDNIDH-NSHDSACASA-N
2	[M+H]^+^	4.07	295.1288	295.1286	−0.86	C_14_H_18_N_2_O_5_	gamma-Glu-Phe	0.004603	84.045, 120.0809, 130.0501, 136.0756, 166.0861, 186.0913, 232.096, 278.1018, 295.1286	XHHOHZPNYFQJKL-QWRGUYRKSA-N
3	[M-H]^−^	1.34	115.0037	115.0039	1.49	C_4_H_4_O_4_	Fumaric acid	0.008435	71.014, 97.9314, 114.9342, 115.0039	VZCYOOQTPOCHFL-OWOJBTEDSA-N
4	[M+H]^+^	9.33	304.1444	304.1438	−1.97	C_19_H_17_N_3_O	Evodiamine	0.009191	134.0599, 171.0913, 304.1442	TXDUTHBFYKGSAH-SFHVURJKSA-N
5	[M-H, 2M-H]^−^	6.1	431.0984	431.0983	−0.13	C_21_H_20_O_10_	Emodin-8-glucoside	0.004102	269.0454, 431.0979	HSWIRQIYASIOBE-JNHRPPPUSA-N
6	[M-H]^−^	7.43	229.1445	229.1446	0.24	C_12_H_22_O_4_	Dodecanedioic acid	0.001865	99.9259, 112.9857, 116.9293, 130.0875, 167.144, 211.1339, 228.1591, 229.1444	TVIDDXQYHWJXFK-UHFFFAOYSA-N
7	[M+H]^+^	5.58	302.1288	302.1282	−2.03	C_19_H_15_N_3_O	Dehydroevodiamine	0.009602	287.1049, 302.1282	VXHNSVKJHXSKKM-UHFFFAOYSA-N
8	[M+FA-H]^−^	5.37	277.0679	277.0669	−3.58	C_8_H_12_N_2_O_6_	Kifunensine	0.010155	209.0795	OIURYJWYVIAOCW-UHFFFAOYSA-N
9	[M+H-H_2_O]^+^	0.71	163.0601	163.0599	−0.9	C_6_H_12_O_6_	d-Mannose	0.007622	107.0494, 117.0547, 118.0350, 119.0491, 123.0404, 127.0389, 128.0706, 141.0506, 162.0759, 163.0386	WQZGKKKJIJFFOK-PQMKYFCFSA-N
10	[M-H]^−^	3.87	259.1299	259.1299	−0.06	C_11_H_20_N_2_O_5_	gamma-Glutamylleucine	0.001003	128.0355, 130.0873, 146.9383, 173.9944, 179.0359, 197.1286, 223.1078, 241.1191, 259.0633, 259.1295	MYFMARDICOWMQP-YUMQZZPRSA-N
11	[M-H_2_O-H, M-H]^−^	1.23	191.0197	191.0199	1.06	C_6_H_8_O_7_	Citric acid	1.885035	85.0296, 111.0089, 128.0353, 129.0195	KRKNYBCHXYNGOX-UHFFFAOYSA-N
12	[M-H]^−^	5.37	187.0976	187.0978	0.95	C_9_H_16_O_4_	Azelaic acid	0.09136	97.0658, 125.0973, 187.0978	BDJRBEYXGGNYIS-UHFFFAOYSA-N
13	[M+H-H_2_O]^+^	9.35	471.3469	471.3464	−0.96	C_30_H_48_O_5_	Arjunolic acid	0.000739	233.1534, 277.1783, 407.3284, 413.3041, 425.3384, 435.3241, 441.3327, 453.3359, 470.3333, 471.3466	RWNHLTKFBKYDOJ-DDHMHSPCSA-N
14	[M-H, M+FA-H]^−^	0.85	195.051	195.0511	0.59	C_5_H_10_O_5_	Arabinose	0.568192	75.0088, 80.9170, 85.0294, 87.0088, 89.0245, 120.9545, 121.0294, 149.0092, 149.0244, 149.0455	SRBFZHDQGSBBOR-SQOUGZDYSA-N
15	[M-H]^−^	0.91	157.0367	157.0369	0.92	C_4_H_6_N_4_O_3_	Allantoin	0.079695	71.0254, 89.0246, 96.9223, 96.9602, 97.0045, 114.031, 140.0101, 157.0367	POJWUDADGALRAB-UHFFFAOYSA-N
16	[M+H]^+^	1.28	268.104	268.1036	−1.51	C_10_H_13_N_5_O_4_	Adenosine	0.091131	136.0618, 268.1037	OIRDTQYFTABQOQ-KQYNXXCUSA-N
17	[M-H]^−^	1.55	182.0459	182.0461	1.15	C_8_H_9_NO_4_	4-Pyridoxic acid	0.001768	112.9858, 115.0401, 119.0353, 121.0447, 124.0072, 138.0561, 138.9071, 163.0612, 181.0720, 182.0457	HXACOUQIXZGNBF-UHFFFAOYSA-N
18	[M+H]^+^	1.22	140.0342	140.0341	−0.7	C_6_H_5_NO_3_	3-Hydroxypicolinic acid	0.077143	74.0971, 112.0395, 140.034	BRARRAHGNDUELT-UHFFFAOYSA-N
19	[M+H]^+^	4.92	481.316	481.3152	−1.68	C_27_H_44_O_7_	25R-Inokosterone	3.047055	165.1271, 173.0958, 249.1460, 303.1949, 371.2210, 409.2725, 427.2835, 445.2941, 463.3046, 481.3154	JQNVCUBPURTQPQ-GYVHUXHASA-N
20	[M-H]^−^	4.12	353.0878	353.0879	0.15	C_16_H_18_O_9_	Chlorogenic acid	0.001511	191.0561, 353.0881	CWVRJTMFETXNAD-JUHZACGLSA-N
21	[M-H]^−^	12.13	271.2279	271.2278	−0.31	C_16_H_32_O_3_	2-Hydroxyhexadecanoic acid	0.002268	225.2223, 271.2277	JGHSBPIZNUXPLA-HNNXBMFYSA-N
22	[M-H, M+FA-H, 2M-H]^−^	0.83	179.0561	179.0563	1.1	C_6_H_12_O_6_	Glucose	1.037797	72.9931, 77.0547, 85.0295, 89.0246, 95.0132, 101.0243, 113.0249, 119.0352, 121.0445, 179.0567	GZCGUPFRVQAUEE-VANKVMQKSA-N
23	[M+H, M+Na]^+^	4.09	247.1441	247.1439	−0.76	C_14_H_18_N_2_O_2_	Hypaphorine	0.000864	59.0611, 60.0816, 85.029, 145.9325, 146.06, 188.0705, 207.1015, 246.1242, 246.1698, 247.144	AOHCBEAZXHZMOR-ZDUSSCGKSA-N
24	[M-H]^−^	6.8	215.1289	215.1289	0.22	C_11_H_20_O_4_	Undecanedioic acid	0.004839	153.1286, 197.1187, 215.0101, 215.129	LWBHHRRTOZQPDM-UHFFFAOYSA-N
25	[M+H]^+^	4.52	497.3109	497.3103	−1.24	C_27_H_44_O_8_	Turkesterone	0.124028	345.2051, 371.2209, 385.2366, 387.2155, 407.2562, 425.2677, 443.2786, 461.2887, 479.3007, 497.3104	WSBAGDDNVWTLOM-XHZKDPLLSA-N
26	[M+NH_4_]^+^	0.93	360.1501	360.1492	−2.31	C_12_H_22_O_11_	Turanose	0.478689	69.0342, 85.0288, 97.0287, 127.0389, 145.0493, 163.0597, 180.0863, 289.0909, 325.1123	RULSWEULPANCDV-PIXUTMIVSA-N
27	[M+FA-H]^−^	4.68	186.1135	186.1138	1.36	C_8_H_15_NO	Tropine	0.001222	79.9574, 80.9652, 97.0658, 107.0503, 125.0973, 142.1239, 186.1136	CYHOMWAPJJPNMW-DHBOJHSNSA-N
28	[M-H]^−^	8.48	243.1602	243.1602	0.11	C_13_H_24_O_4_	Tridecanedioic acid	0.001745	146.9611, 174.9559, 181.1595, 225.1494, 242.1766, 243.1597	DXNCZXXFRKPEPY-UHFFFAOYSA-N
29	[M-H]^−^	9.28	257.1758	257.176	0.82	C_14_H_26_O_4_	Tetradecanedioic acid	0.000628	195.1759, 239.1652, 257.1758	HQHCYKULIHKCEB-UHFFFAOYSA-N
30	[M-H]^−^	4.85	173.0819	173.0821	0.99	C_8_H_14_O_4_	Suberic acid	0.008054	93.0346, 99.9259, 104.9538, 111.0817, 115.9207, 116.9283, 129.0923, 130.0875, 172.0978, 173.0820	TYFQFVWCELRYAO-UHFFFAOYSA-N
31	[M+NH_4_]^+^	9.64	302.3054	302.3049	−1.49	C_18_H_36_O_2_	Stearic acid	0.007109	88.0763, 91.0547, 302.305	QIQXTHQIDYTFRH-UHFFFAOYSA-N
32	[M-H]^−^	10.29	285.2071	285.2071	−0.16	C_16_H_30_O_4_	Hexadecanedioic acid	0.002157	223.2067, 267.197, 285.2072	QQHJDPROMQRDLA-UHFFFAOYSA-N
33	[M-H, M+FA-H]^−^	0.8	711.2201	711.2198	−0.33	C_24_H_42_O_21_	Stachyose	3.048184	89.0245, 101.0244, 113.0246, 125.0241, 143.0347, 161.0451, 179.0560, 221.0663, 341.1080, 665.2141	UQZIYBXSHAGNOE-XNSRJBNMSA-N
34	[M+H, M+Na]^+^	8.97	343.1176	343.1169	−2.01	C_19_H_18_O_6_	Scutellarein tetramethyl ether	0.154561	157.0128, 313.0701, 343.1171	URSUMOWUGDXZHU-UHFFFAOYSA-N
35	[M-H]^−^	5.59	137.0244	137.0246	1.42	C_7_H_6_O_3_	Salicylic acid	0.021021	93.0348, 137.0247	YGSDEFSMJLZEOE-UHFFFAOYSA-N
36	[M+FA-H]^−^	4.47	431.1923	431.1921	−0.38	C_19_H_30_O_8_	Roseoside	0.027857	71.0140, 89.0246, 101.0249, 113.0244, 119.0355, 153.0917, 179.0567, 223.1350, 385.1871, 431.1916	SWYRVCGNMNAFEK-MHXFFUGFSA-N
37	[M+H]^+^	1.22	130.0499	130.0499	0.07	C_5_H_7_NO_3_	Pyroglutamic acid	1.325712	84.045, 84.0814, 87.0046, 96.0098, 98.5125, 113.9639, 130.05	ODHCTXKNWHHXJC-VKHMYHEASA-N
38	[M-H, M+FA-H]^−^	4.82	435.1297	435.1296	−0.28	C_20_H_22_O_8_	Polydatin	0.001234	185.0606, 227.0715	HSTZMXCBWJGKHG-CUYWLFDKSA-N
39	[M+H]^+^	4.96	153.0546	153.0545	−1.03	C_8_H_8_O_3_	Isovanillin	0.022396	111.0205, 111.0442, 111.9685, 125.0597, 126.0548, 129.9789, 134.0598, 136.0755, 152.0704, 153.0545	JVTZFYYHCGSXJV-UHFFFAOYSA-N
40	[M+H-H_2_O, M+NH_4_]^+^	0.92	684.2557	684.2544	−1.88	C_24_H_42_O_21_	Isomaltotetraose	0.761064	162.0757, 163.0597, 180.0863, 259.0810, 271.0804, 289.0912, 325.1121, 343.1226, 487.1653, 684.2551	DFKPJBWUFOESDV-NGZVDTABSA-N
41	[2M-H]^−^	10.53	291.1615	291.1601	−4.77	C_9_H_10_N_2_	Indole-3-methanamine	0.013972	219.1752, 235.171, 263.1653, 291.1603	JXYGLMATGAAIBU-UHFFFAOYSA-N
42	[M-H]^−^	6.01	201.1132	201.1134	0.89	C_10_H_18_O_4_	Sebacic acid	0.008393	89.0245, 116.9289, 121.066, 139.113, 183.103, 201.0226, 201.1131	CXMXRPHRNRROMY-UHFFFAOYSA-N
43	[M+H]^+^	14.88	338.3417	338.3411	−1.84	C_22_H_43_NO	13-Docosenamide	0.062269	69.0705, 71.0861, 81.0704, 83.0861, 95.0858, 97.1015, 111.1174, 303.3048, 321.3154, 338.3410	UAUDZVJPLUQNMU-UHFFFAOYSA-N
44	[M-H]^−^	1.39	282.0844	282.0844	−0.03	C_10_H_13_N_5_O_5_	Guanosine	0.009596	133.0159, 150.0421, 282.0841	NYHBQMYGNKIUIF-UUOKFMHZSA-N
45	[M-H, M+FA-H]^−^	1.21	243.0623	243.0622	−0.3	C_9_H_12_N_2_O_6_	1-*β*-d-Arabinofuranosyluracil	0.012177	128.0360, 152.0354, 153.0305, 158.6527, 174.8874, 185.9930, 200.0569, 213.3648, 216.4419, 243.0624	DRTQHJPVMGBUCF-CCXZUQQUSA-N
46	[M+H-H_2_O, M+H]^+^	0.89	147.0764	147.0763	−1	C_5_H_10_N_2_O_3_	l-Glutamine	0.50798	83.0609, 84.045, 84.0813, 129.0659	ZDXPYRJPNDTMRX-VKHMYHEASA-N
47	[M-H]^−^	0.78	154.0622	154.0624	1.45	C_6_H_9_N_3_O_2_	l-Histidine	0.00218	93.0459, 94.9252, 96.9222, 96.9602, 96.969, 110.0725, 137.0358, 154.0623	HNDVDQJCIGZPNO-YFKPBYRVSA-N
48	[M+H]^+^	1.5	132.1019	132.1019	−0.07	C_6_H_13_NO_2_	l-Leucine	0.482585	69.0706, 72.9378, 86.0969, 88.0047, 97.0099, 113.9641, 132.1021	ROHFNLRQFUQHCH-YFKPBYRVSA-N
49	[M+H-H_2_O, M+H]^+^	0.73	147.1128	147.1127	−0.55	C_6_H_14_N_2_O_2_	l-Lysine	0.008875	84.0449, 84.0813	KDXKERNSBIXSRK-YFKPBYRVSA-N
50	[M+H]^+^	1.14	150.0583	150.0582	−0.52	C_5_H_11_NO_2_S	l-Methionine	0.001225	56.0503, 61.0115, 74.0244, 74.0608, 76.0764, 87.0269, 102.0554, 104.0532, 133.0318, 150.0582	FFEARJCKVFRZRR-BYPYZUCNSA-N
51	[M-H]^−^	2.26	164.0717	164.0719	1.37	C_9_H_11_NO_2_	l-Phenylalanine	0.002097	96.9602, 96.9693, 119.0502, 120.0456, 121.0293, 136.9325, 147.0453, 163.0616, 164.0355, 164.0723	COLNVLDHVKWLRT-QMMMGPOBSA-N
52	[M+H, M+NH_4_]^+^	10.78	279.2319	279.2313	−1.99	C_18_H_30_O_2_	Punicic acid	0.044704	57.0706, 67.0548, 81.0703, 95.0859, 109.1014, 123.1167, 137.1319, 149.0231, 173.1325, 279.2314	CUXYLFPMQMFGPL-MRZTUZPCSA-N
53	[M-H, M+FA-H]^−^	0.78	873.2729	873.2725	−0.46	C_30_H_52_O_26_	Maltopentaose	2.750419	71.0140, 89.0245, 101.0246, 113.0244, 125.0212, 143.0350, 161.0451, 179.0564, 221.0666, 827.2667	FJCUPROCOFFUSR-GMMZZHHDSA-N
54	[M-H, M+FA-H]^−^	0.8	549.1672	549.1671	−0.25	C_18_H_32_O_16_	Manninotriose	3.098184	113.0244, 119.0348, 143.0353, 161.0453, 179.0562, 221.0661, 323.0975, 341.1084, 383.1195, 503.1615	FZWBNHMXJMCXLU-YRBKNLIBSA-N
55	[M+H-H_2_O, M+H, M+K, M+Na]^+^	0.76	162.0761	162.0758	−1.35	C_6_H_13_NO_5_	Mannosamine	0.970436	101.0237, 102.0552, 103.0393, 114.0550, 115.0391, 116.0708, 126.0549, 127.0389, 144.0652, 162.0758	MSWZFWKMSRAUBD-CBPJZXOFSA-N
56	[M+H]^+^	4.23	154.0499	154.0498	−0.32	C_7_H_7_NO_3_	Methyl 5-hydroxypyridine-2-carboxylate	0.021269	72.9378, 90.9481, 112.0395, 113.9638, 131.9742, 140.034, 154.0505	YYAYXDDHGPXWTA-UHFFFAOYSA-N
57	[M-H]^−^	1.36	117.0193	117.0195	1.81	C_4_H_6_O_4_	Methylmalonic acid	0.050209	73.0296, 99.0089, 99.926, 116.9287, 117.0195	ZIYVHBGGAOATLY-UHFFFAOYSA-N
58	[M-H]^−^	1.46	292.1402	292.1403	0.37	C_12_H_23_NO_7_	*N*-(1-Deoxy-1-fructosyl)leucine	0.051817	101.0246, 128.0356, 130.0875, 202.1084	KGTRBDVOUPALMB-PPNLDZOPSA-N
59	[M-H_2_O-H, M-H]^−^	0.96	133.0142	133.0145	1.63	C_4_H_6_O_5_	Malic acid	1.602263	71.014, 114.9343, 115.0038	BJEPYKJPYRNKOW-REOHCLBHSA-N
60	[M+NH4, M+Na]^+^	7.84	949.4404	949.4384	−2.11	C_46_H_70_O_19_	Achyranthoside E	0.392011	189.1633, 191.1792, 201.1634, 203.1791, 205.1948, 247.1689, 309.0447, 393.3502, 439.3565, 944.4835	DVEJWYUSLPQXTD-UHFFFAOYSA-N
61	[M-H_2_O-H, M-H]^−^	0.93	189.0041	189.0043	0.96	C_6_H_8_O_8_	Hydroxycitric acid	0.480247	73.0296, 83.0139, 85.0296, 87.0088, 99.0088, 111.0088, 127.0038, 129.0195, 189.0041	ZMJBYMUCKBYSCP-CVYQJGLWSA-N
62	[M-H]^−^	9.9	313.2384	313.2384	0.04	C_18_H_34_O_4_	12,13-DHOME	0.06907	129.0922, 183.1393, 201.1133, 313.238	CQSLTKIXAJTQGA-BTDPBSJTSA-N
63	[M+H-H_2_O, M+H]^+^	2.28	328.1391	328.1383	−2.45	C_15_H_21_NO_7_	*N*-(1-Deoxy-1-fructosyl)phenylalanine	0.231268	97.0288, 120.081, 127.0392, 132.0808, 166.0864, 178.0862, 264.1228, 292.118, 310.0899, 310.1281	FAVRCIXPIVJIPN-VJDSNFAGSA-N
64	[M-H]^−^	1.43	130.051	130.0512	1.72	C_5_H_9_NO_3_	*N*-Acetyl-l-alanine	0.011574	71.0141, 74.0248, 85.0296, 86.0612, 86.9402, 87.0452, 88.0405, 101.0246, 129.0197, 130.0512	KTHDTJVBEPMMGL-VKHMYHEASA-N
65	[M+H, 2M+H, 2M+Na, M+Na]^+^	3.76	205.0972	205.0969	−1.27	C_11_H_12_N_2_O_2_	l-Tryptophan	0.060655	118.0651, 144.0807, 146.06, 159.0917, 188.0705	QIVBCDIJIAJPQS-VIFPVBQESA-N
66	[M-H]^−^	1.23	147.0299	147.0301	1.64	C_5_H_8_O_5_	l-2-Hydroxyglutaric acid	0.020338	87.0088, 87.0453, 89.0246, 101.0245, 101.0610, 102.9490, 103.0401, 129.0195, 129.0558, 147.0301	HWXBTNAVRSUOJR-VKHMYHEASA-N
67	[M+FA-H]^−^	0.86	135.0298	135.0301	2.72	C_3_H_6_O_3_	d-Lactic acid	0.019063	61.9884, 72.9932, 75.0088, 89.0246, 117.0195, 134.0473, 135.0301	JVTAAEKCZFNVCJ-UWTATZPHSA-N
68	[M-H]^−^	4.98	206.0823	206.0824	0.41	C_11_H_13_NO_3_	*N*-Acetyl-l-phenylalanine	0.007067	73.0296, 79.9576, 85.0296, 89.0248, 131.0352, 147.0453, 161.0457, 164.0718, 166.0000, 206.0823	CBQJSKKFNMDLON-JTQLQIEISA-N
69	[M+H, M+K]^+^	0.76	175.119	175.1188	−0.68	C_6_H_14_N_4_O_2_	l-Arginine	0.314091	112.0869, 113.0711, 116.0709, 129.1024, 130.0977, 135.0028, 151.9380, 158.0925, 159.0765, 175.1190	ODKSFYDXXFIFQN-BYPYZUCNSA-N
70	[M+H-H_2_O]^+^	0.92	130.0499	130.0499	0.07	C_5_H_9_NO_4_	l-Glutamic acid	0.771336	83.061, 84.0451, 84.0814, 130.0501	WHUUTDBJXJRKMK-VKHMYHEASA-N
71	[M+FA-H]^−^	5.51	507.2964	507.2961	−0.45	C_27_H_42_O_6_	Podecdysone B	0.096931	75.009, 159.103, 301.1814, 461.2914, 507.2961	AEFMTBQZWMUASH-IILZZRPCSA-N
72	[M-H]^−^	2.76	218.1034	218.1035	0.26	C_9_H_17_NO_5_	Pantothenic acid	0.025937	71.0139, 88.0405, 92.9281, 116.9069, 146.0823, 159.8601, 218.1028	GHOKWGTUZJEAQD-ZETCQYMHSA-N
73	[M-H_2_O-H, M+FA-H, M-H]^−^	0.82	387.1144	387.1143	−0.38	C_12_H_22_O_11_	Palatinose	3.615434	59.014, 71.014, 89.0246, 101.0245, 113.0246, 119.0351, 179.0563, 341.1089	PVXPPJIGRGXGCY-UHFFFAOYSA-N
74	[M-H_2_O-H, M-H]^−^	0.91	145.0142	145.0144	1.35	C_5_H_6_O_5_	Oxoglutaric acid	0.016136	101.0608, 101.0721, 102.0562, 107.0252, 109.0409, 125.0360, 126.0196, 127.0515, 128.0356, 145.0143	KPGXRSRHYNQIFN-UHFFFAOYSA-N
75	[M-H]^−^	11.03	313.2384	313.2384	−0.02	C_18_H_34_O_4_	Octadecanedioic acid	0.001225	251.2379, 295.2279, 313.2384	BNJOQKFENDDGSC-UHFFFAOYSA-N
76	[2M-H]^−^	10.4	315.2541	315.2539	−0.55	C_9_H_18_O_2_	Pelargonic acid	0.010138	112.9855, 246.9448, 315.2537	FBUKVWPVBMHYJY-UHFFFAOYSA-N
77	[M+Na]^+^	0.95	689.2111	689.2098	−1.92	C_24_H_42_O_21_	Nystose	4.056686	185.042, 203.0528, 347.094, 365.1043, 527.1553, 689.209	FLDFNEBHEXLZRX-DLQNOBSRSA-N
78	[M+H, M+Na]^+^	0.76	189.1598	189.1596	−1.04	C_9_H_20_N_2_O_2_	*N*6,*N*6,*N*6-Trimethyl-l-lysine	0.010983	60.0816, 80.9485, 84.0814, 130.0865, 143.118, 144.1126, 188.1395, 189.1334, 189.1597	MXNRLFUSFKVQSK-QMMMGPOBSA-N
79	[M-H, M+FA-H]^−^	6.03	342.1347	342.1345	−0.61	C_19_H_21_NO_5_	*N*-*trans*-sinapoyltyramine	0.102446	135.045, 148.0532, 178.0507, 190.0516, 327.1111, 342.1354	IEDBNTAKVGBZEP-VMPITWQZSA-N
80	[M+H, 2M+Na, M+K]^+^	5.9	314.1387	314.138	−2.05	C_18_H_19_NO_4_	*N*-Feruloyltyramine	0.241944	121.0648, 145.0281, 177.0543, 314.138	NPNNKDMSXVRADT-WEVVVXLNSA-N
81	[M-H]^−^	5.07	328.119	328.119	−0.04	C_18_H_19_NO_5_	*N*-Feruloyloctopamine	0.01335	133.0535, 161.0245, 295.0848, 297.0404, 310.1085, 328.1202	VJSCHQMOTSXAKB-YCRREMRBSA-N
82	[M+H]^+^	5.1	247.1077	247.1075	−1.03	C_13_H_14_N_2_O_3_	*N*-Acetyltryptophan	0.00161	176.9718, 187.0865, 188.0704, 201.1019, 205.0978, 206.0813, 207.1012, 229.0990, 246.1325, 247.0998	DZTHIGRZJZPRDV-LBPRGKRZSA-N
83	[M+H, 2M+H]^+^	0.89	148.0604	148.0602	−1.36	C_5_H_9_NO_4_	*N*-Acetylserine	0.200878	84.045, 84.0814, 102.0554, 130.05	JJIHLJJYMXLCOY-BYPYZUCNSA-N
84	[M-H]^−^	4.74	172.0979	172.0982	1.55	C_8_H_15_NO_3_	*N*-Acetylleucine	0.006483	111.0818, 130.0875, 172.098	WXNXCEHXYPACJF-ZETCQYMHSA-N
85	[M+Na]^+^	6.75	387.2142	387.2135	−1.97	C_21_H_32_O_5_	Pergularin	0.011047	57.0707, 73.0655, 89.0602, 101.0965, 145.1222, 243.0989, 347.2213, 387.2136	VJMNSJUASLIQEP-UPFSRWTJSA-N
86	[M+NH_4_]^+^	10.46	241.2275	241.2269	−2.54	C_14_H_25_NO	Pellitorine	0.005497	200.2005	MAGQQZHFHJDIRE-BNFZFUHLSA-N
87	[M+H-H_2_O]^+^	7.12	633.3997	633.3984	−2.07	C_36_H_58_O_10_	Pedunculoside	0.013272		LARPFJIXBULVPK-FBAXZNBGSA-N
88	[M+H]^+^	10.39	277.2162	277.2157	−1.9	C_18_H_28_O_2_	Parinaric acid	0.020866	119.0856, 121.1013, 133.1010, 135.1166, 137.0596, 147.1167, 149.1324, 235.1693, 277.1795, 277.2158	IJTNSXPMYKJZPR-UHFFFAOYSA-N
89	[M-H]^−^	5.73	282.1136	282.1134	−0.56	C_17_H_17_NO_3_	*p*-Coumaroyltyramine	0.000862		RXGUTQNKCXHALN-BJMVGYQFSA-N
90	[M-H_2_O-H]^−^	6.45	535.1821	535.1823	0.35	C_26_H_34_O_13_	osthenol-7-*o*-*β*-gentiobioside	0.01308	78.9529, 95.0134, 96.9601, 104.6861, 110.2844, 137.3326, 152.9869, 241.0017, 513.1867, 535.1809	LCNBLLDTRINYAW-NXEOTYAVSA-N
91	[M+Na]^+^	9.11	374.0999	374.1001	0.61	C_20_H_17_NO_5_	Oxyberberine	0.000787		ZHYQCBCBTQWPLC-UHFFFAOYSA-N
92	[M+FA-H]^−^	4.71	475.1821	475.182	−0.23	C_20_H_30_O_10_	Phenethyl rutinoside	0.009461	163.0612, 167.7898, 189.8643, 205.0708, 258.2900, 269.4057, 272.7622, 429.1785, 460.7686, 475.1847	OKUGUNDXBGUFPA-UHFFFAOYSA-N
93	[M+FA-H]^−^	4.64	525.1614	525.1612	−0.3	C_23_H_28_O_11_	Paeoniflorin	0.001499		YKRGDOXKVOZESV-UHFFFAOYSA-N
94	[M+H-H_2_O]^+^	6.75	455.3519	455.351	−2.16	C_30_H_48_O_4_	Phlegmaric acid	0.060044	203.1792, 205.1588, 205.1951, 207.1739, 249.1849, 397.3095, 409.3452, 425.3403, 437.3408, 455.3515	UCBRMUIDZFUDIJ-KBUITVGKSA-N
95	[M-H, M+FA-H]^−^	4.61	541.3018	541.3018	−0.07	C_27_H_44_O_8_	5-*β*-hydroxyecdysterone	0.398516	83.0504, 85.0298, 87.0452, 99.0452, 145.0872, 157.087, 175.0977, 319.1918, 495.2964, 541.3013	GMFLGNRCCFYOKL-ACCCYTKYSA-N
96	[M+FA-H]^−^	4.38	727.2455	727.2456	0.2	C_32_H_42_O_16_	Pinoresinol Diglucoside	0.00149		ZJSJQWDXAYNLNS-FUPWJLLWSA-N
97	[2M+H]^+^	8.93	377.102	377.1034	3.93	C_11_H_8_O_3_	Plumbagin	0.002221		VCMMXZQDRFWYSE-UHFFFAOYSA-N
98	[M+H]^+^	10.43	293.2111	293.2105	−2.22	C_18_H_28_O_3_	Polyacetylene PQ-1	0.029545	81.0702, 95.0495, 99.0806, 151.1114, 163.1114, 223.1324, 257.1910, 275.2001, 293.1755, 293.2101	QSLYECSTHSYXDL-KSZLIROESA-N
99	[M-H_2_O-H]^−^	6.08	969.4701	969.4712	1.11	C_48_H_76_O_21_	Bayogenin-3-*O*-[*β*-d-Galactose-(1→3)-*β*-d-glucuronic acid-28-*O*-*β*-d-glucopyranoside	0.013741		SQVBXHAEJALFEQ-RYEUOLHJSA-N
100	[M+H]^+^	5.63	195.1016	195.1013	−1.47	C_11_H_14_O_3_	3,4-Dimethoxycinnamyl alcohol	0.002244		OYICGYUCCHVYRR-ONEGZZNKSA-N

**Table 2:** Prototype components absorbed by the AR plasma group, MCAO+AR plasma group, AR brain group, and MCAO+AR brain group.

No	Adducts	RT (min)	Theoretical *m*/*z*	*m*/*z*	Mass Error (ppm)	Formula	Metabolites	Ratio of Peak Area %	Fragment Ions	InChIKey
1	[M+H]^+^	3.83	219.1128	219.1125	−1.32	C_12_H_14_N_2_O_2_	Abrine	0.149136	88.0397, 132.0807, 144.0807, 146.0598, 188.0703, 200.1278, 219.1132	CZCIKBSVHDNIDH-NSHDSACASA-N
2	[M-H_2_O-H, M-H]^−^	0.93	189.0041	189.0043	0.96	C_6_H_8_O_8_	Hydroxycitric acid	0.480244	73.0296, 83.0139, 85.0296, 87.0088, 99.0088, 111.0088, 127.0038, 129.0195, 189.0041	ZMJBYMUCKBYSCP-CVYQJGLWSA-N
3	[M+FA-H]^−^	1.27	221.0303	221.0303	0.02	C_6_H_8_O_6_	Ascorbyl	0.007983	59.0139, 72.9933, 73.0297, 83.014, 99.009, 103.0039, 127.0039, 159.0298, 189.0042, 221.0295	CIWBSHSKHKDKBQ-UHFFFAOYSA-N
4	[M-H]^−^	9.19	955.4544	955.4541	−0.37	C_47_H_72_O_20_	Betavulgaroside III	1.005851	71.0139, 89.0245, 101.0244, 113.0245, 455.3524, 569.3864, 793.437, 835.4518, 955.4528	GNCYMXULNXKROG-UHFFFAOYSA-N
5	[M-H_2_O-H, M-H]^−^	0.91	189.0041	189.0043	0.89	C_6_H_8_O_8_	Hydroxycitric acid	0.542071	73.0296, 83.0139, 85.0296, 87.0088, 99.0089, 111.0088, 127.0037, 129.0194, 189.0041	ZMJBYMUCKBYSCP-CVYQJGLWSA-N
6	[M+H-H_2_O, M+H]^+^	9.97	399.3257	399.325	−1.74	C_27_H_44_O_3_	Sarsasapogenin	0.018722	115.0756, 121.1013, 147.1165, 159.1167, 161.1324, 255.2105, 285.2569, 359.2144, 381.3141, 399.3253	GMBQZIIUCVWOCD-WWASVFFGSA-N
7	[M+FA-H]^−^	1.27	221.0303	221.0302	−0.48	C_6_H_8_O_6_	Ascorbyl	0.009148	72.9931, 73.0296, 83.0140, 94.9252, 99.0086, 103.0037, 127.0037, 159.0297, 189.0039, 221.0292	CIWBSHSKHKDKBQ-UHFFFAOYSA-N
8	[M-H]^−^	0.92	157.0367	157.0367	0.19	C_4_H_6_N_4_O_3_	Allantoin	0.072752	71.0251, 89.0244, 96.0457, 97.0044, 114.0309, 140.0101, 157.0368	POJWUDADGALRAB-UHFFFAOYSA-N
9	[M+H, M+Na, M+NH_4_]^+^	0.91	365.1055	365.1048	−1.94	C_12_H_22_O_11_	Sucrose	2.398857	185.0419, 203.0522, 365.1049	CZMRCDWAGMRECN-UGDNZRGBSA-N
10	[M-H, M+FA-H, M-H_2_O-H]^−^	0.81	387.1144	387.1142	−0.6	C_12_H_22_O_11_	Palatinose	3.321968	59.014, 71.0139, 89.0245, 101.0244, 113.0243, 119.0349, 161.0456, 179.0562, 341.1085	PVXPPJIGRGXGCY-UHFFFAOYSA-N
11	[M+NH_4_, M+Na]^+^	0.91	527.1583	527.1574	−1.72	C_18_H_32_O_16_	Melezitose	2.832384	144.0652, 145.0493, 162.0757, 163.0597, 180.0864, 259.0801, 289.0912, 325.1122, 343.1228, 522.2019	QWIZNVHXZXRPDR-WSCXOGSTSA-N
12	[M+FA-H]^−^	4.46	431.1923	431.1921	−0.42	C_19_H_30_O_8_	Roseoside	0.0257	61.9884, 71.0139, 89.0249, 101.0244, 119.0354, 153.0924, 161.044, 179.0566, 385.1876, 431.1905	SWYRVCGNMNAFEK-MHXFFUGFSA-N
13	[M-H]^−^	6.12	301.0354	301.0354	0.11	C_15_H_10_O_7_	Morin	0.000739	107.0139, 121.0295, 151.0037, 178.9985, 301.0351	YXOLAZRVSSWPPT-UHFFFAOYSA-N

**Table 3:** Absorbed metabolites in the AR plasma group, MCAO+AR plasma group, AR brain group, and MCAO+AR brain group.

No	Adducts	RT (min)	*m*/*z*	Mass Error (ppm)	Parent Compound	Formula	Metabolites	Transformations	Metabolism Type
1	[M-H_2_O-H]^−^	5.20	529.1331	−3.69	(+)-Balanophonin	C_26_H_28_O_13_	(+)-Balanophonin_M1	Hydroxylation, glucuronidation	I, II
2	[M+H-H_2_O]^+^	8.54	427.2838	−1.17	(25*R*)-Spirosta-1,4-diene-3,12-dione	C_27_H_40_O_5_	(25R)-Spirosta-1,4-diene-3,12-dione_M1	Deglycosidation, reduction	I
3	[M-H]^−^	6.51	469.2081	0.41	1-Dehydro-6-gingerdione	C_23_H_34_O_10_	1-Dehydro-6-gingerdione_M1	Reduction, reduction, glucuronidation	I, II
4	[M-H_2_O-H]^−^	6.56	567.2991	−1.09	16-Oxoalisol A	C_30_H_50_O_9_S	16-Oxoalisol A_M1	Reduction, sulfation	I, II
5	[M+H]^+^	9.42	469.3306	−1.39	16α-Hydroxydehydrotrametenolic acid	C_30_H_44_O_4_	16α-Hydroxydehydrotrametenolic acid_M1	Oxidation	I
6	[M+FA-H]^−^	9.41	531.3330	0.62	16α-Hydroxydehydrotrametenolic acid	C_30_H_46_O_5_	16α-Hydroxydehydrotrametenolic acid_M2-1	Hydroxylation	I
7	[M-H]^−^	9.95	485.3271	−0.39	16α-Hydroxydehydrotrametenolic acid	C_30_H_46_O_5_	16α-Hydroxydehydrotrametenolic acid_M2-2	Hydroxylation	I
8	[M+FA-H]^−^	9.95	531.3330	0.52	16α-Hydroxydehydrotrametenolic acid	C_30_H_46_O_5_	16α-Hydroxydehydrotrametenolic acid_M2-3	Hydroxylation	I
9	[M-H]^−^	7.15	565.2842	0.18	16α-Hydroxydehydrotrametenolic acid	C_30_H_46_O_8_S	16α-Hydroxydehydrotrametenolic acid_M3	Hydroxylation, sulfation	I, II
10	[M+H]^+^	9.26	503.3358	−1.73	16α-Hydroxydehydrotrametenolic acid	C_30_H_46_O_6_	16α-Hydroxydehydrotrametenolic acid_M4	Hydroxylation, hydroxylation	I
11	[M-H]^−^	10.01	483.3118	0.43	18α-Glycyrrhetinic acid	C_30_H_44_O_5_	18α-Glycyrrhetinic acid_M1	Oxidation, hydroxylation	I
12	[M-H_2_O-H]^−^	9.03	423.1462	2.86	3-Isomangostin	C_24_H_26_O_8_	3-Isomangostin_M1	Hydroxylation, hydroxylation	I
13	[M-H]^−^	5.75	473.1091	0.28	5,6,7-Trimethoxyflavone	C_23_H_22_O_11_	5,6,7-Trimethoxyflavone_M1	Demethylation, glucuronidation	I, II
14	[M-H]^−^	4.45	337.0934	1.43	5-Hydroxy-1-tetralone	C_16_H_18_O_8_	5-Hydroxy-1-tetralone_M1	Glucuronidation	II
15	[M-H_2_O-H]^−^	6.30	491.2629	−4.24	5α-Pregnane-3β,6α-diol-20-one	C_27_H_42_O_9_	5α-Pregnane-3β,6α-diol-20-one_M1	Glucuronidation	II
16	[M+FA-H]^−^	9.36	533.3490	1.28	Ainidiol	C_30_H_48_O_5_	Ainidiol_M1-1	Carboxylation, hydroxylation	I
17	[M-H]^−^	9.38	487.3436	1.37	Ainidiol	C_30_H_48_O_5_	Ainidiol_M1-2	Carboxylation, hydroxylation	I
18	[2M-H]^−^	8.59	567.3178	0.65	Artemether	C_15_H_24_O_5_	Artemether_M1	Demethylation	I
19	[2M-H]^−^	9.49	499.3069	0.71	Costunolide	C_15_H_22_O_3_	Costunolide_M1	Hydroxylation, reduction	I
20	[M-H_2_O-H]^−^	5.24	427.0650	−4.70	Damnacanthol	C_21_H_18_O_11_	Damnacanthol_M1	Demethylation, glucuronidation	I, II
21	[M+H-H_2_O]^+^	9.48	297.1845	−1.16	Dehydroabietic acid	C_20_H_26_O_3_	Dehydroabietic acid_M1	Hydroxylation, oxidation	I
22	[M-H]^−^	7.32	491.2288	0.31	Dehydroabietic acid	C_26_H_36_O_9_	Dehydroabietic acid_M2	Hydroxylation, glucuronidation	I, II
23	[M-H]^−^	5.77	531.1512	0.67	Dehydrodiisoeugenol	C_26_H_28_O_12_	Dehydrodiisoeugenol_M1	Carboxylation, glucuronidation	I, II
24	[M+H-H_2_O]^+^	4.80	485.0763	2.92	Demethylsuberosin	C_20_H_22_O_13_S	Demethylsuberosin_M1	Hydroxylation, glucuronidation, sulfation	I, II
25	[M+NH_4_]^+^	6.84	566.2227	−0.83	Dihydrocurcumin	C_27_H_32_O_12_	Dihydrocurcumin_M1	Reduction, glucuronidation	I, II
26	[M+Na]^+^	5.12	471.0896	−0.51	Flavokawain C	C_21_H_20_O_11_	Flavokawain C_M1	Demethylation, demethylation, glucuronidation	I, II
27	[M+FA-H]^−^	9.56	393.1925	1.93	Jolkinolide B	C_20_H_28_O_5_	Jolkinolide B_M1	Epoxide hydrolysis	I
28	[M-H]^−^	7.85	493.2447	0.75	Kaurenoic acid	C_26_H_38_O_9_	Kaurenoic acid_M1	Hydroxylation, glucuronidation	I, II
29	[M-H]^−^	6.58	509.2388	−0.83	Kaurenoic acid	C_26_H_38_O_10_	Kaurenoic acid_M2	Hydroxylation, hydroxylation, glucuronidation	I, II
30	[2M+H]^+^	9.52	501.3203	−1.61	Kissoone A	C_15_H_22_O_3_	Kissoone A_M1	Hydroxylation, hydroxylation	I
31	[M+H-H_2_O]^+^	8.26	405.1679	−4.15	Licoflavone B	C_25_H_26_O_6_	Licoflavone B_M1	Hydroxylation, hydroxylation	I
32	[M-H_2_O-H]^−^	5.84	403.1400	0.36	Lindenenol	C_21_H_26_O_9_	Lindenenol_M1	Hydroxylation, glucuronidation	I, II
33	[M+Na]^+^	5.52	519.1495	4.36	Methyl mycophenolate	C_23_H_28_O_12_	Methyl mycophenolate_M1	Demethylation, glucuronidation	I, II
34	[M-H]^−^	7.23	567.3003	0.95	Momordicine I	C_30_H_48_O_8_S	Momordicine I_M1	Hydroxylation, sulfation	I, II
35	[M+H-H_2_O]^+^	9.79	430.2947	−1.06	*N-*Benzyloleamide	C_26_H_41_NO_5_	*N-*Benzyloleamide_M1	Carboxylation, hydroxylation	I
36	[M+H]^+^	4.89	490.1702	−1.18	*N-*Feruloyltyramine	C_24_H_27_NO_10_	*N-*Feruloyltyramine_M1	Glucuronidation	II
37	[M-H]^−^	4.96	518.1671	0.52	*N-*trans-sinapoyltyramine	C_25_H_29_NO_11_	*N-*trans-sinapoyltyramine_M1-1	Glucuronidation	II
38	[M+H]^+^	4.98	520.1808	−0.95	*N-*trans-sinapoyltyramine	C_25_H_29_NO_11_	*N-*trans-sinapoyltyramine_M1-2	Glucuronidation	II
39	[M-H]^−^	11.09	485.3274	0.23	Nigranoic acid	C_30_H_46_O_5_	Nigranoic acid_M1	Hydroxylation	I
40	[2M+H]^+^	5.59	623.3127	1.79	Nuciferine	C_19_H_21_NO_3_	Nuciferine_M1	Oxidation	I
41	[M-H]^−^	4.91	349.0569	1.07	Padmatin	C_16_H_14_O_9_	Padmatin_M1	Hydroxylation, hydroxylation	I
42	[M-H_2_O-H]^−^	5.49	517.1354	0.43	Peucedanocoumarin II	C_25_H_28_O_13_	Peucedanocoumarin II_M1	Deacetylation, hydroxylation, glucuronidation	I, II
43	[M-H]^−^	4.68	537.1982	0.82	Secoisolariciresinol	C_26_H_34_O_12_	Secoisolariciresinol_M1	Glucuronidation	II
44	[M-H]^−^	5.09	593.1880	0.78	Syringaresinol	C_28_H_34_O_14_	Syringaresinol_M1	Glucuronidation	II
45	[M-H]^−^	9.58	485.3275	0.57	Ursolic acid acetic acid	C_30_H_46_O_5_	Ursolic acid acetic acid_M1	Deacetylation, carboxylation	I
46	[M-H]^−^	10.34	485.3271	−0.21	Ursolic aldehyde	C_30_H_46_O_5_	Ursolic aldehyde_M1	Carboxylation, hydroxylation	I
47	[M-H]^−^	4.43	399.1661	0.06	Vomifoliol	C_19_H_28_O_9_	Vomifoliol_M1	Glucuronidation	II
48	[M+FA-H]^−^	4.69	481.0989	0.29	p-hydroxy-5,6-dehydrokawain	C_20_H_20_O_11_	p-hydroxy-5,6-dehydrokawain_M1	Hydroxylation, glucuronidation	I, II
49	[2M+NH_4_]^+^	11.86	1042.7327	−1.41	(3α,4β)-3-(Acetyloxy)ursa-5,12-dien-23-oic acid	C_32_H_48_O_5_	(3α,4β)-3-(Acetyloxy)ursa-5,12-dien-23-oic acid_M1	Hydroxylation	I
50	[2M+NH_4_]^+^	8.67	354.2632	−2.03	1,8-Cineole	C_10_H_16_O_2_	1,8-Cineole_M1	Hydroxylation, oxidation	I
51	[M+FA-H]^−^	4.03	305.0335	−0.45	3,4-Dimethoxycinnamyl alcohol	C_10_H_12_O_6_S	3,4-Dimethoxycinnamyl alcohol_M1	Demethylation, sulfation	I, II
52	[2M+H]^+^	5.53	697.0680	−0.03	7-Hydroxyisoflavone	C_16_H_12_O_7_S	7-Hydroxyisoflavone_M1	Hydroxylation, methylation, sulfation	I, II
53	[M+NH4]^+^	5.75	570.2174	−1.30	Angelol A	C_26_H_32_O_13_	Angelol A_M1	Glucuronidation	II
54	[M-H]^−^	7.21	351.1812	−0.31	Arnicolide C	C_19_H_28_O_6_	Arnicolide C_M1-1	Reduction, hydroxylation	I
55	[M+H-H_2_O]^+^	7.19	335.1846	−2.08	Arnicolide C	C_19_H_28_O_6_	Arnicolide C_M1-2	Reduction, hydroxylation	I
56	[M+NH4]^+^	7.77	554.2225	−1.34	Guaiacin	C_26_H_32_O_12_	Guaiacin_M1	Hydroxylation, hydroxylation, glucuronidation	I, II
57	[M+NH4]^+^	10.92	334.2734	−1.98	Guggulsterone	C_21_H_32_O_2_	Guggulsterone_M1	Reduction, reduction	I
58	[2M-H]^−^	5.79	599.2627	−3.92	Honokiol	C_18_H_20_O_4_	Honokiol_M1	Vinyl oxidation	I
59	[M-H_2_O-H]^−^	11.07	317.2119	−0.80	Incensole Acetic acid	C_20_H_32_O_4_	Incensole Acetic acid_M1	Deacetylation, carboxylation	I
60	[M-H]^−^	4.98	518.1664	−0.74	*N-*trans-sinapoyltyramine	C_25_H_29_NO_11_	*N-*trans-sinapoyltyramine_M1	Glucuronidation	II
61	[2M+H]^+^	5.60	623.3119	0.58	Nuciferine	C_19_H_21_NO_3_	Nuciferine_M1	Oxidation	I
62	[M-H_2_O-H]^−^	4.71	447.0571	0.50	3,4,8,9,10-Pentahydroxy Urolithin	C_20_H_18_O_13_	3,4,8,9,10-Pentahydroxy Urolithin_M1	Glucuronidation, methylation	II
63	[M-H_2_O-H]^−^	4.15	335.0776	1.01	3-*O*-Caffeoylquinic acid methyl ester	C_16_H_18_O_9_	3-*O*-Caffeoylquinic acid methyl ester_M1	Hydrolysis	I
64	[M-H]^−^	10.23	437.2912	0.69	Methyl cholate	C_25_H_42_O_6_	Methyl cholate_M1	Hydroxylation	I
65	[M+H-H_2_O]^+^	6.88	489.3569	−1.03	Olean-12-ene-3β,16β,21β,23,28-pentol	C_30_H_50_O_6_	Olean-12-ene-3β,16β,21β,23,28-pentol_M1	Hydroxylation	I
66	[M-H_2_O-H]^−^	4.30	401.0880	0.51	Oxyresveratrol	C_20_H_20_O_10_	Oxyresveratrol_M1	Glucuronidation	II
67	[M+NH_4_]^+^	4.71	260.0585	−0.99	5-Hydroxy-1-tetralone	C_10_H_10_O_5_S	5-Hydroxy-1-tetralone_M1	Sulfation	II
68	[M-H_2_O-H]^−^	9.73	333.2071	−0.09	Ginkgolic Acid (C13:0)	C_20_H_32_O_5_	Ginkgolic Acid (C13:0)_M1	Hydroxylation, hydroxylation	I

## Data Availability

Date will be made available on request.

## Supplementary Materials

The following supporting information can be downloaded at: https://www.mdpi.com/article/10.3390/molecules29122840/s1.

## Abbreviations

AR	Achyranthes bidentata
BBB	Blood–brain barrier
BPC	Base peak chromatogram
CIRI	Cerebral ischemia-reperfusion injury
DDA	Data-dependent acquisition
EDTA	Ethylenediaminetetraacetic acid
EIC	Extracted ion chromatogram
HR–MS	High resolution-mass spectrometry
LC–MS	Liquid chromatography–mass spectrometry
MCAO	Middle cerebral artery occlusion
SD	Sprague Dawley
TTC	2,3, 5-Triphenyltetrazolium chloride staining
TCM	Traditional Chinese medicine
UHPLC	Ultrahigh performance liquid chromatography
UHPLC–HR-MS	Ultrahigh performance liquid chromatography–high-resolution mass spectrometry
UPLC–MS/MS	ultra performance liquid chromatography/tandem mass spectrometry
UHPLC-Q Exactive Orbitrap-HRMS	Ultrahigh-performance liquid chromatography-Q Exactive Orbitrap-High resolution-mass spectrometry
