# Supplementary figures and images for: Characterization of the Components and Metabolites of Achyranthes Bidentata in the Plasma and Brain Tissue of Rats Based on Ultrahigh Performance Liquid Chromatography–High-Resolution Mass Spectrometry (UHPLC–HR-MS) (part 2 of 2)
Source: Molecules. 2024 Jun 14;29(12):2840. doi: 10.3390/molecules29122840 (PMC11206857; doi:10.3390/molecules29122840)

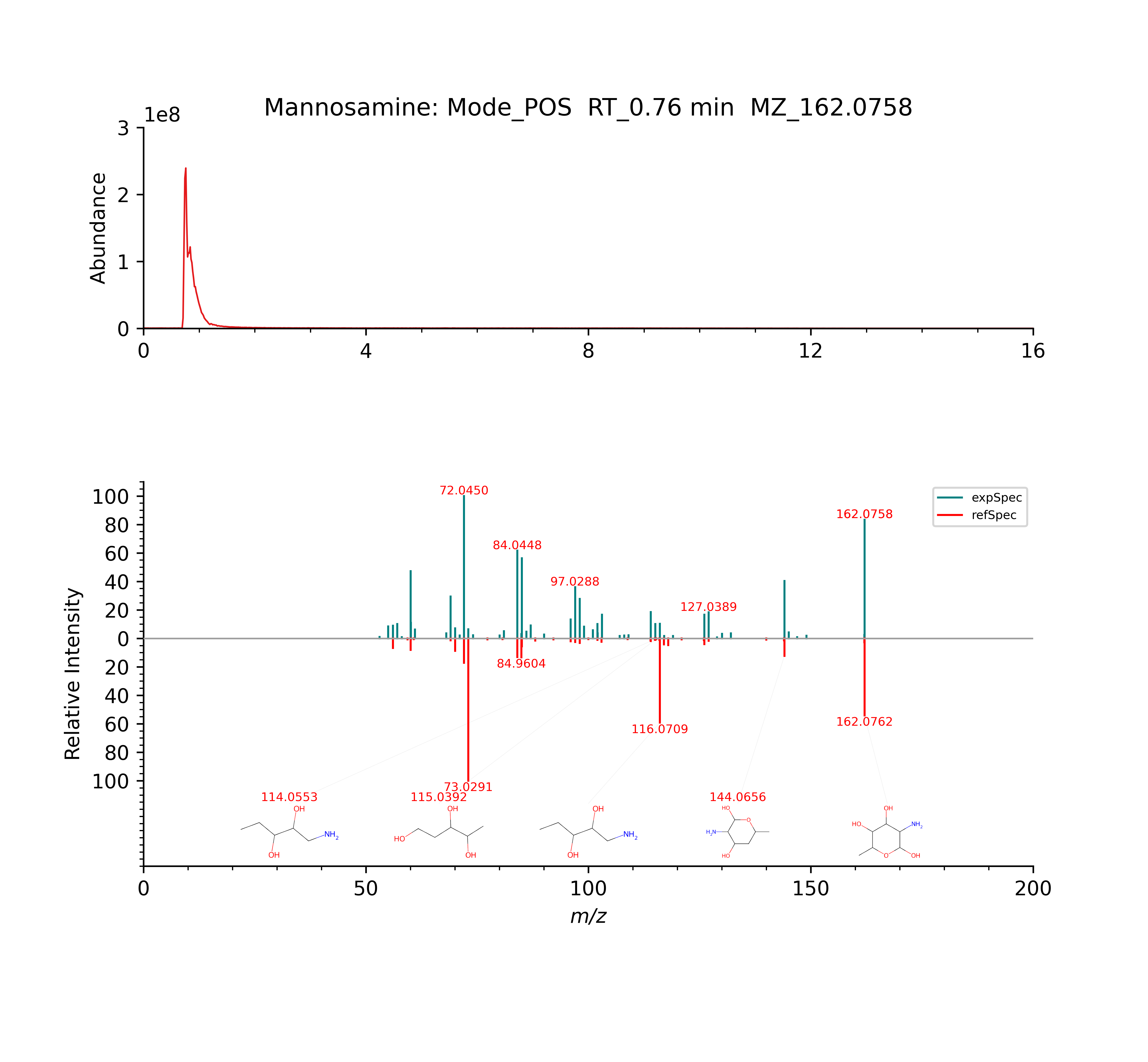

Supplement: Supplementary file 1 [file molecules-29-02840-s001.zip › Supplementary Figure s1/Identification from LuMet-CM datebase/png/compound00058.png]

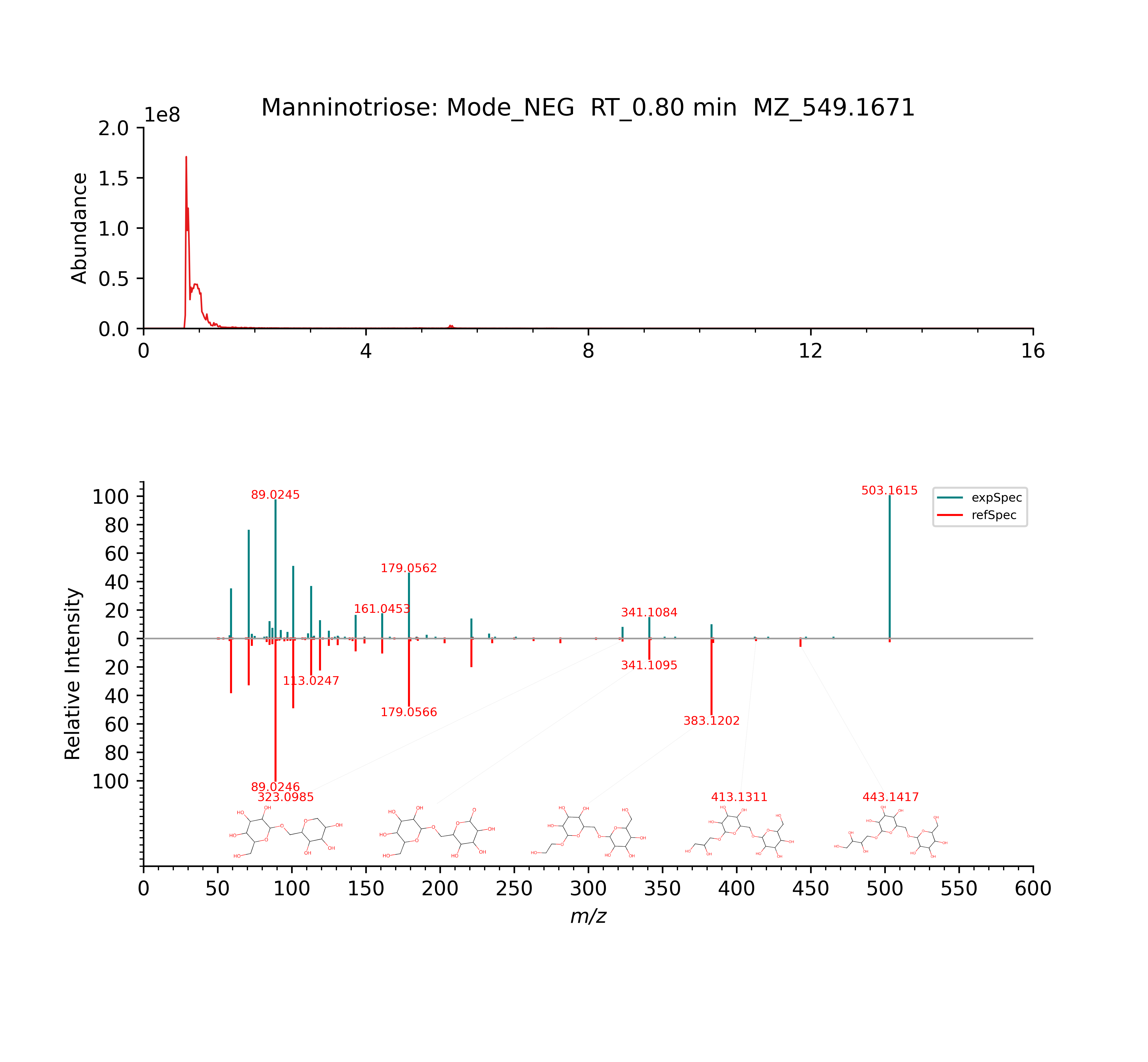

Supplement: Supplementary file 1 [file molecules-29-02840-s001.zip › Supplementary Figure s1/Identification from LuMet-CM datebase/png/compound00059.png]

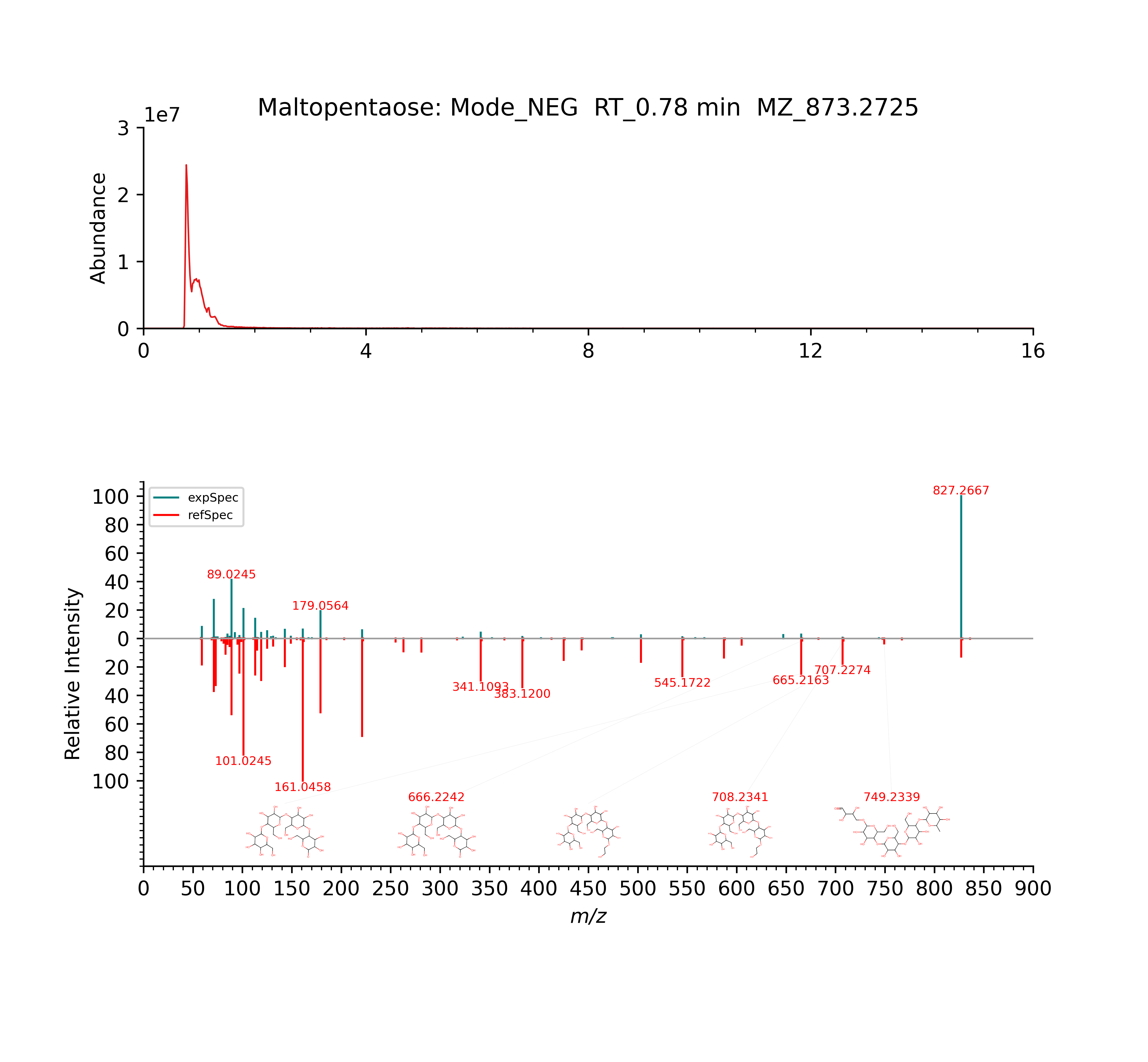

Supplement: Supplementary file 1 [file molecules-29-02840-s001.zip › Supplementary Figure s1/Identification from LuMet-CM datebase/png/compound00060.png]

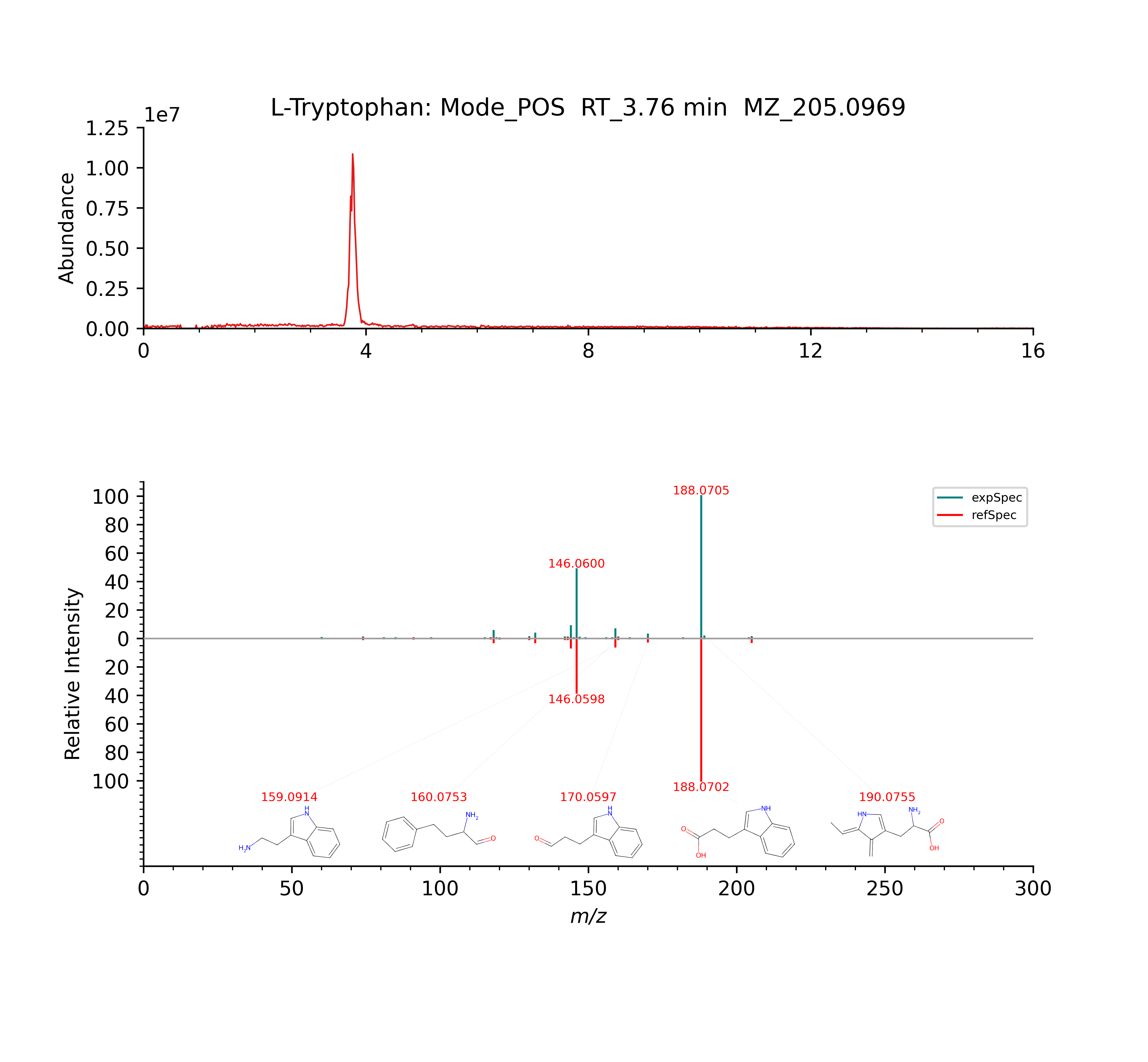

Supplement: Supplementary file 1 [file molecules-29-02840-s001.zip › Supplementary Figure s1/Identification from LuMet-CM datebase/png/compound00061.png]

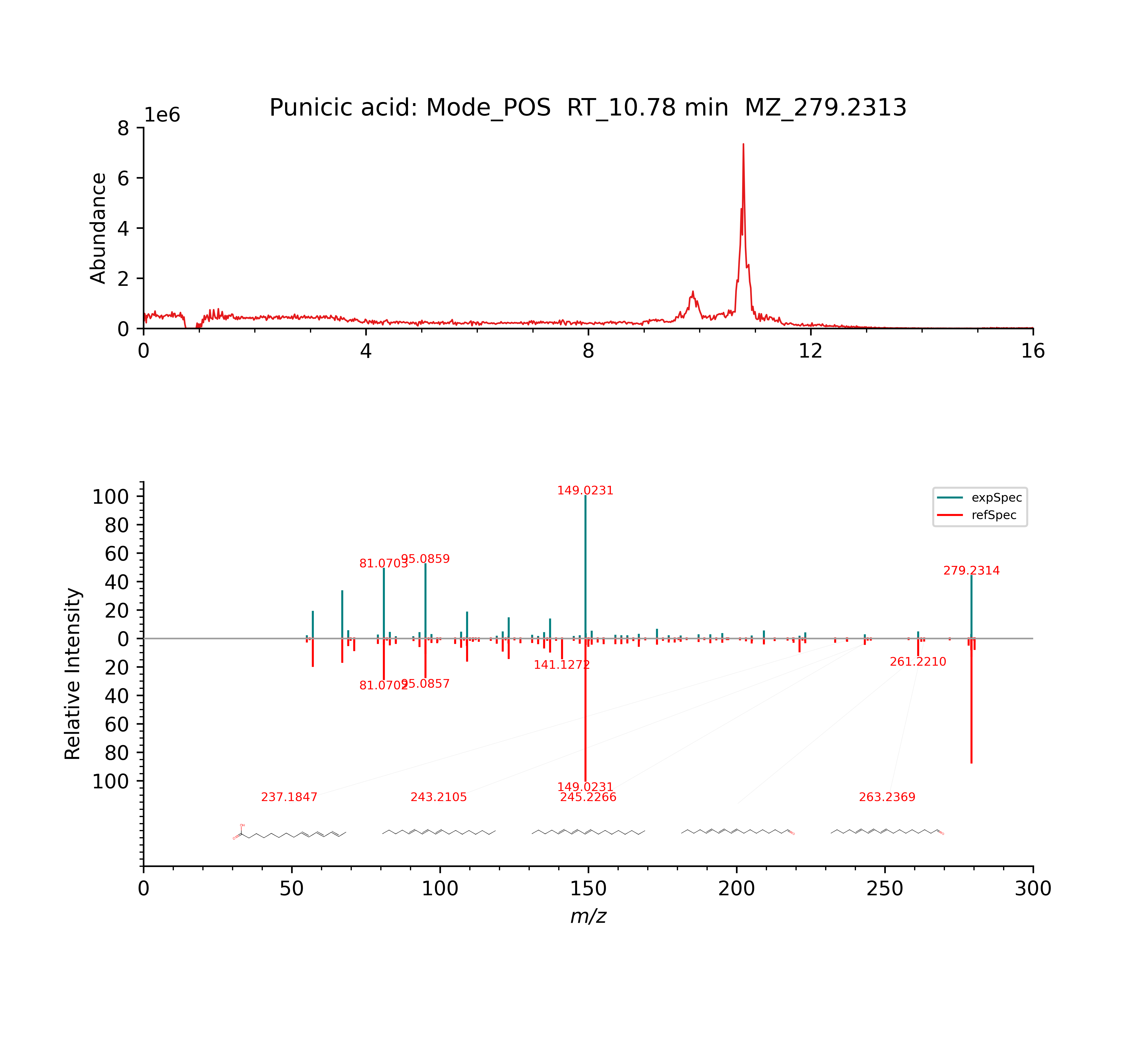

Supplement: Supplementary file 1 [file molecules-29-02840-s001.zip › Supplementary Figure s1/Identification from LuMet-CM datebase/png/compound00062.png]

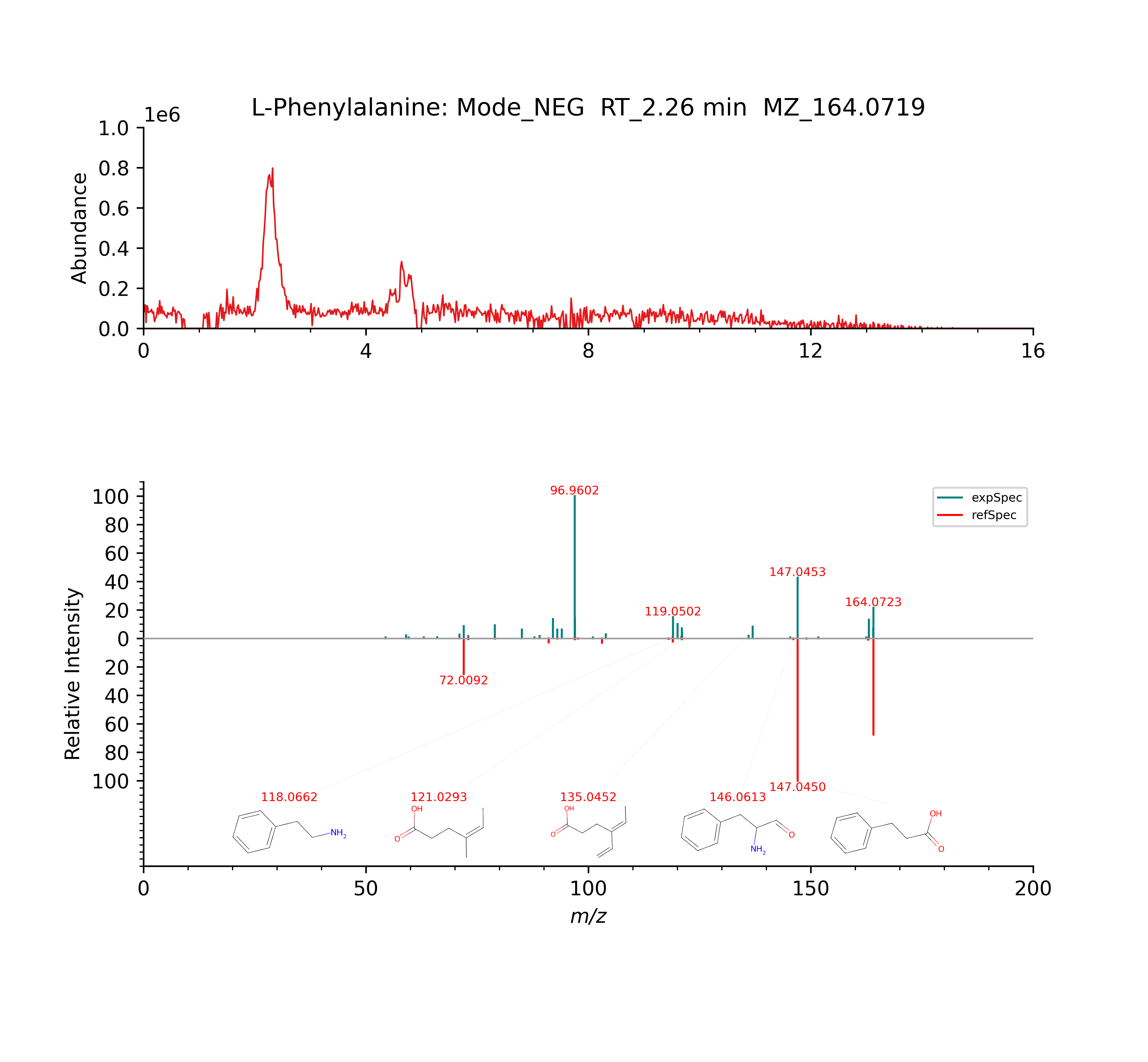

Supplement: Supplementary file 1 [file molecules-29-02840-s001.zip › Supplementary Figure s1/Identification from LuMet-CM datebase/png/compound00063.png]

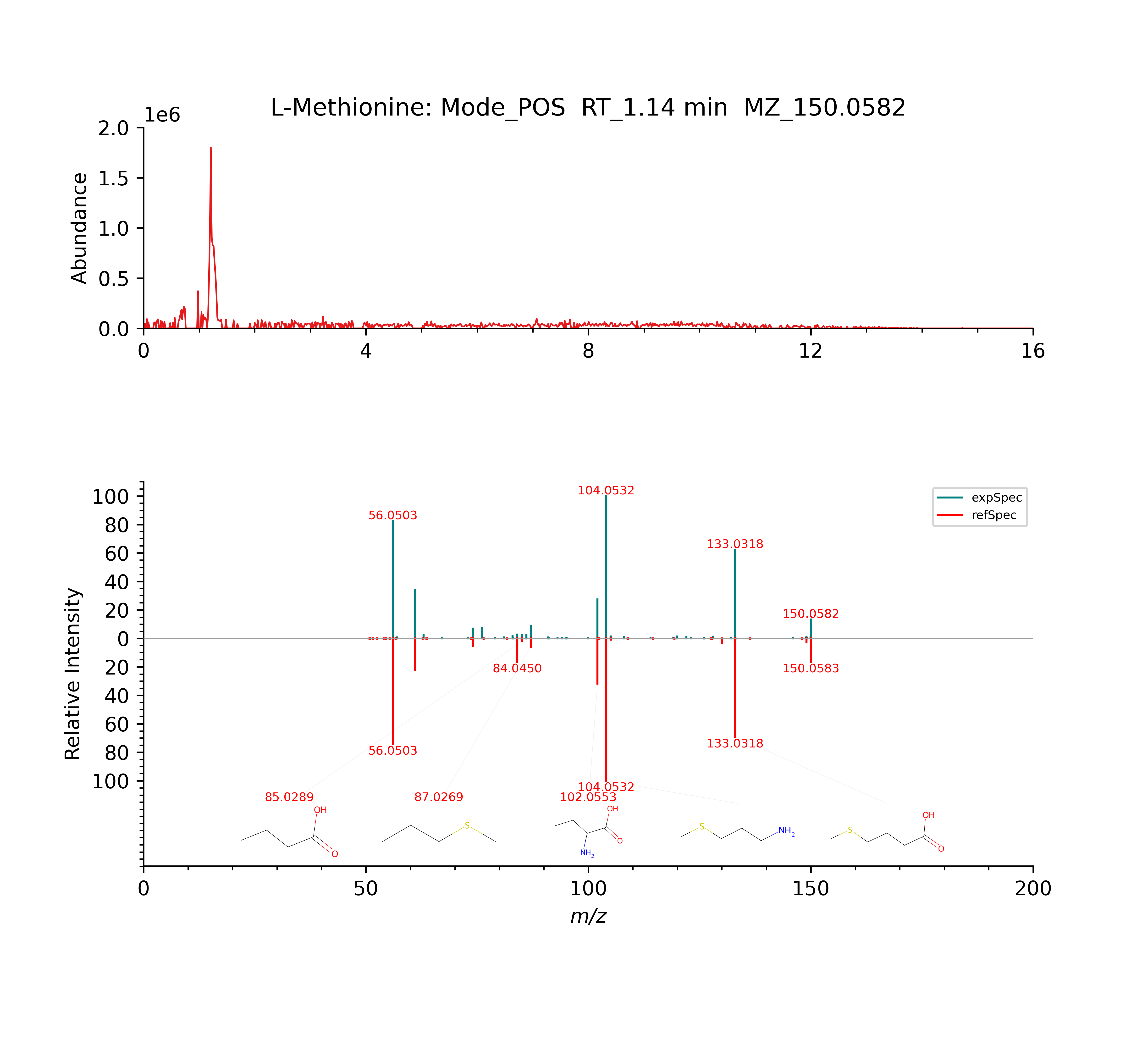

Supplement: Supplementary file 1 [file molecules-29-02840-s001.zip › Supplementary Figure s1/Identification from LuMet-CM datebase/png/compound00064.png]

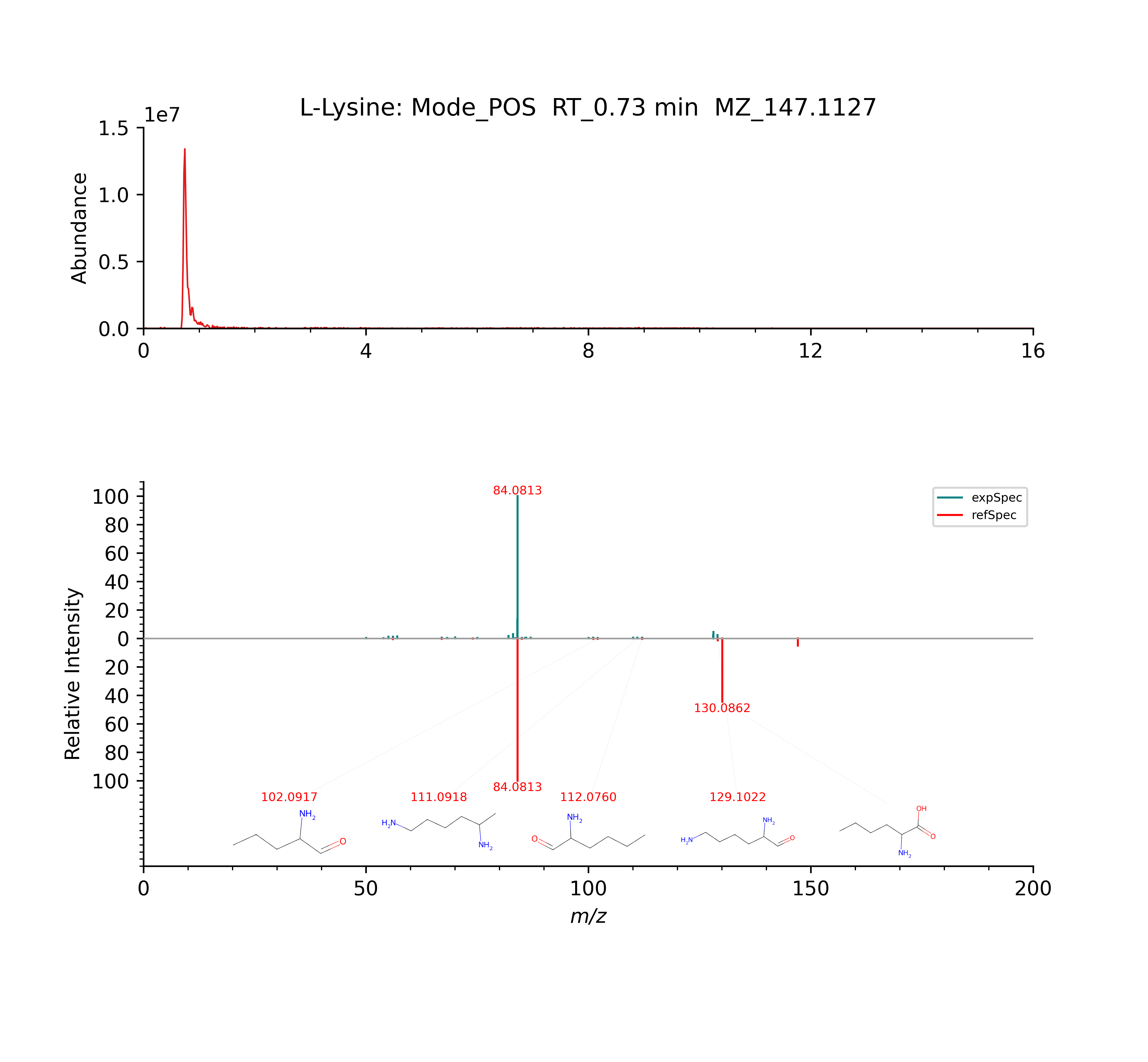

Supplement: Supplementary file 1 [file molecules-29-02840-s001.zip › Supplementary Figure s1/Identification from LuMet-CM datebase/png/compound00065.png]

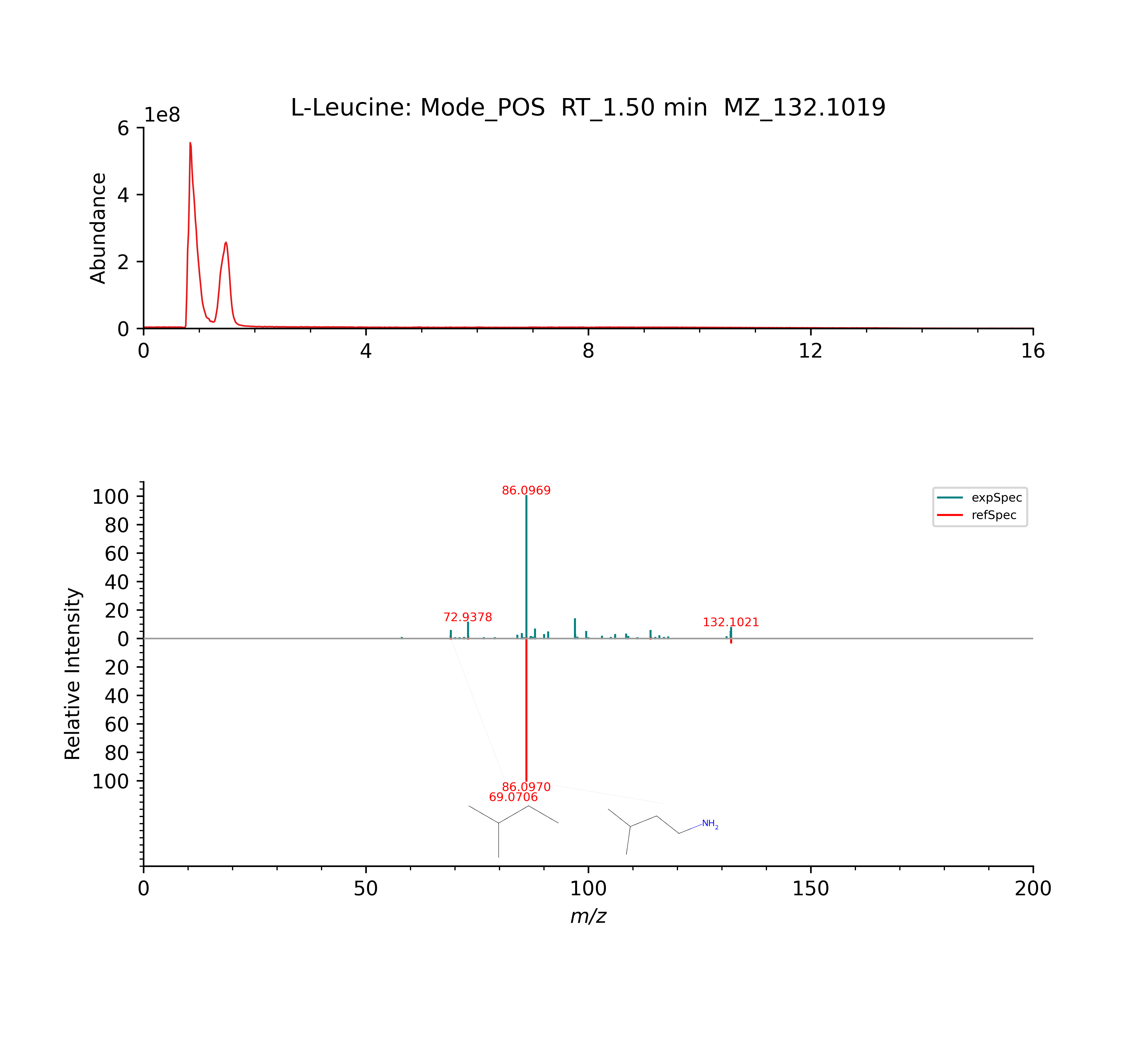

Supplement: Supplementary file 1 [file molecules-29-02840-s001.zip › Supplementary Figure s1/Identification from LuMet-CM datebase/png/compound00066.png]

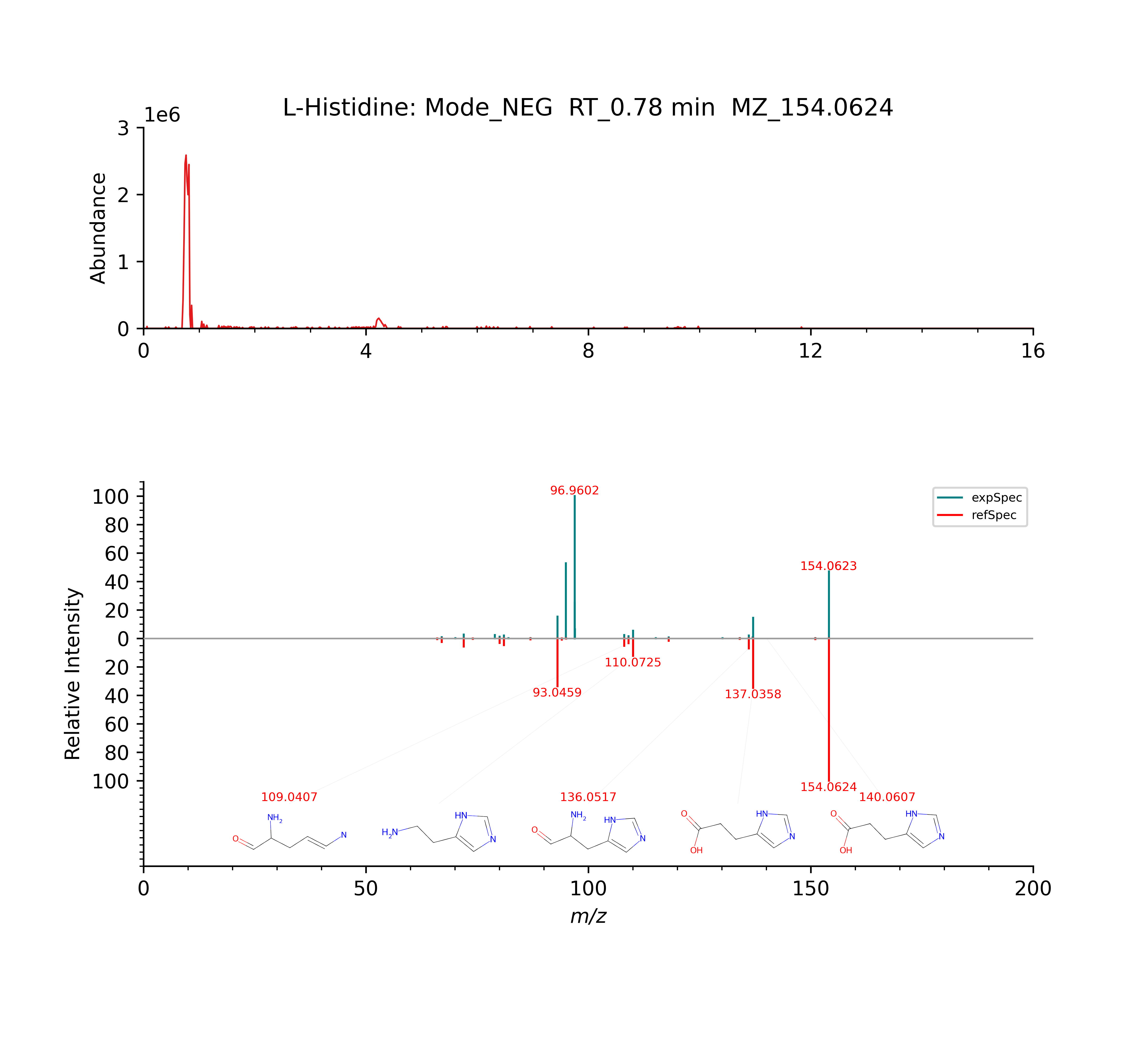

Supplement: Supplementary file 1 [file molecules-29-02840-s001.zip › Supplementary Figure s1/Identification from LuMet-CM datebase/png/compound00067.png]

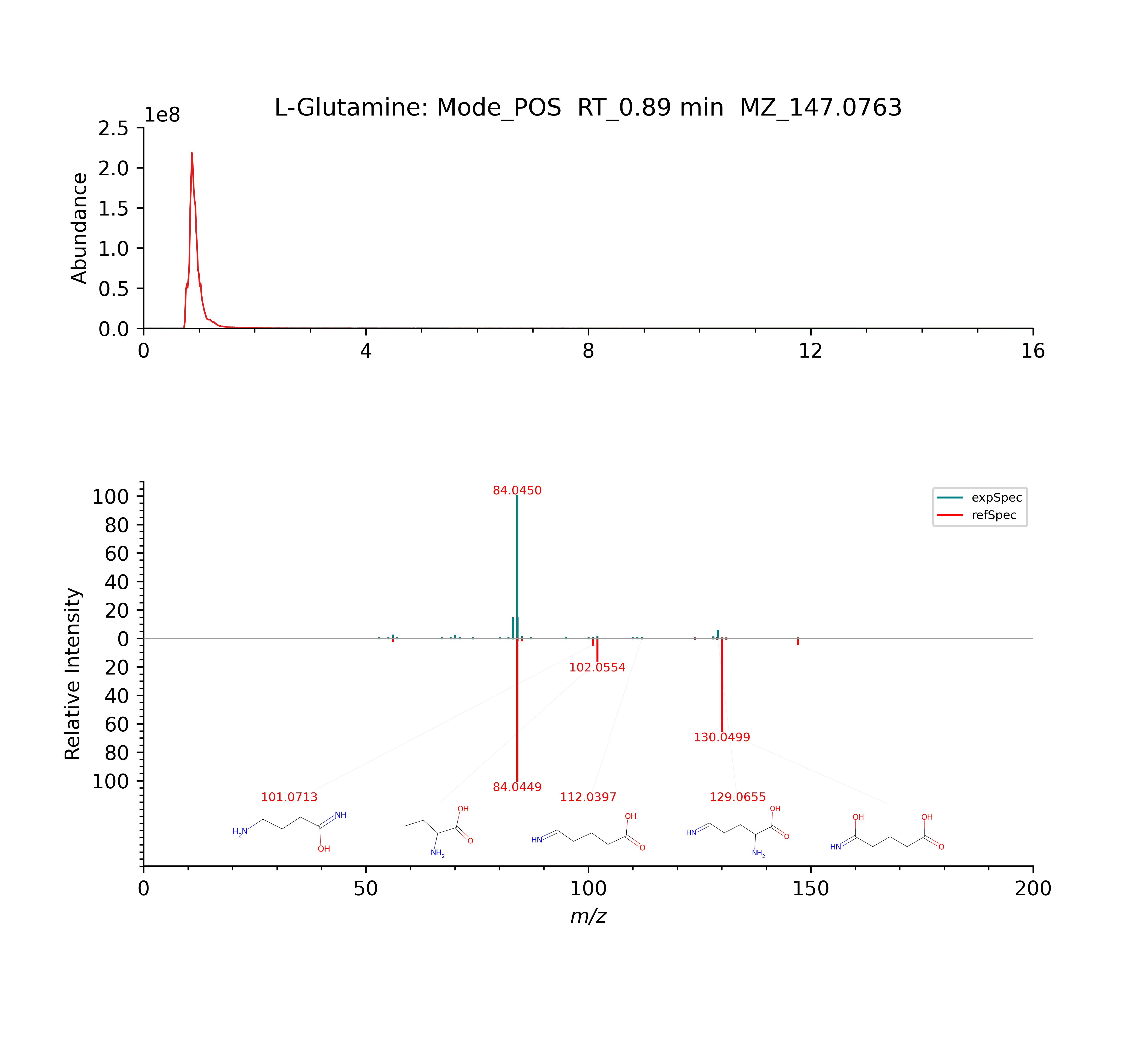

Supplement: Supplementary file 1 [file molecules-29-02840-s001.zip › Supplementary Figure s1/Identification from LuMet-CM datebase/png/compound00068.png]

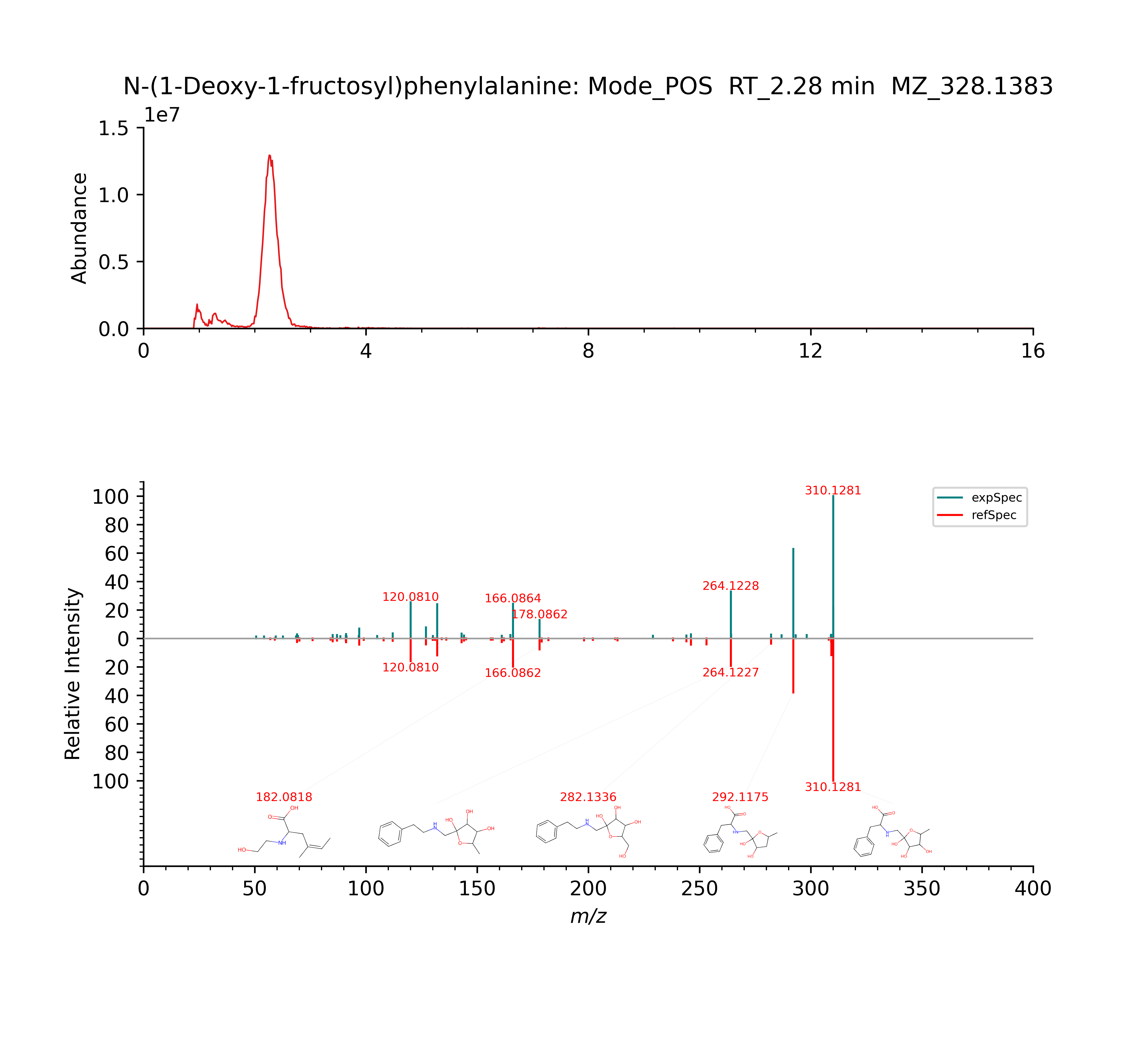

Supplement: Supplementary file 1 [file molecules-29-02840-s001.zip › Supplementary Figure s1/Identification from LuMet-CM datebase/png/compound00069.png]

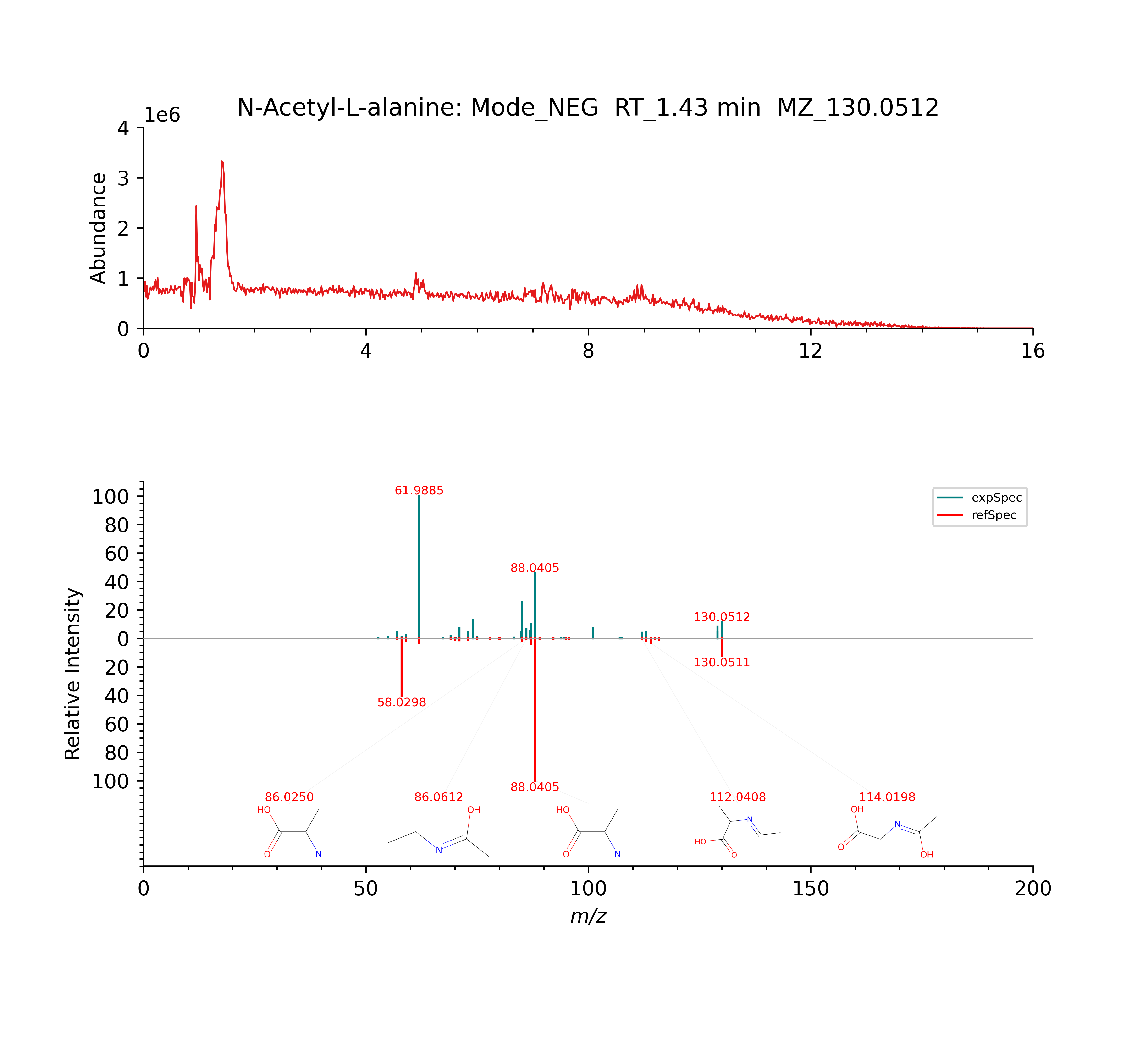

Supplement: Supplementary file 1 [file molecules-29-02840-s001.zip › Supplementary Figure s1/Identification from LuMet-CM datebase/png/compound00070.png]

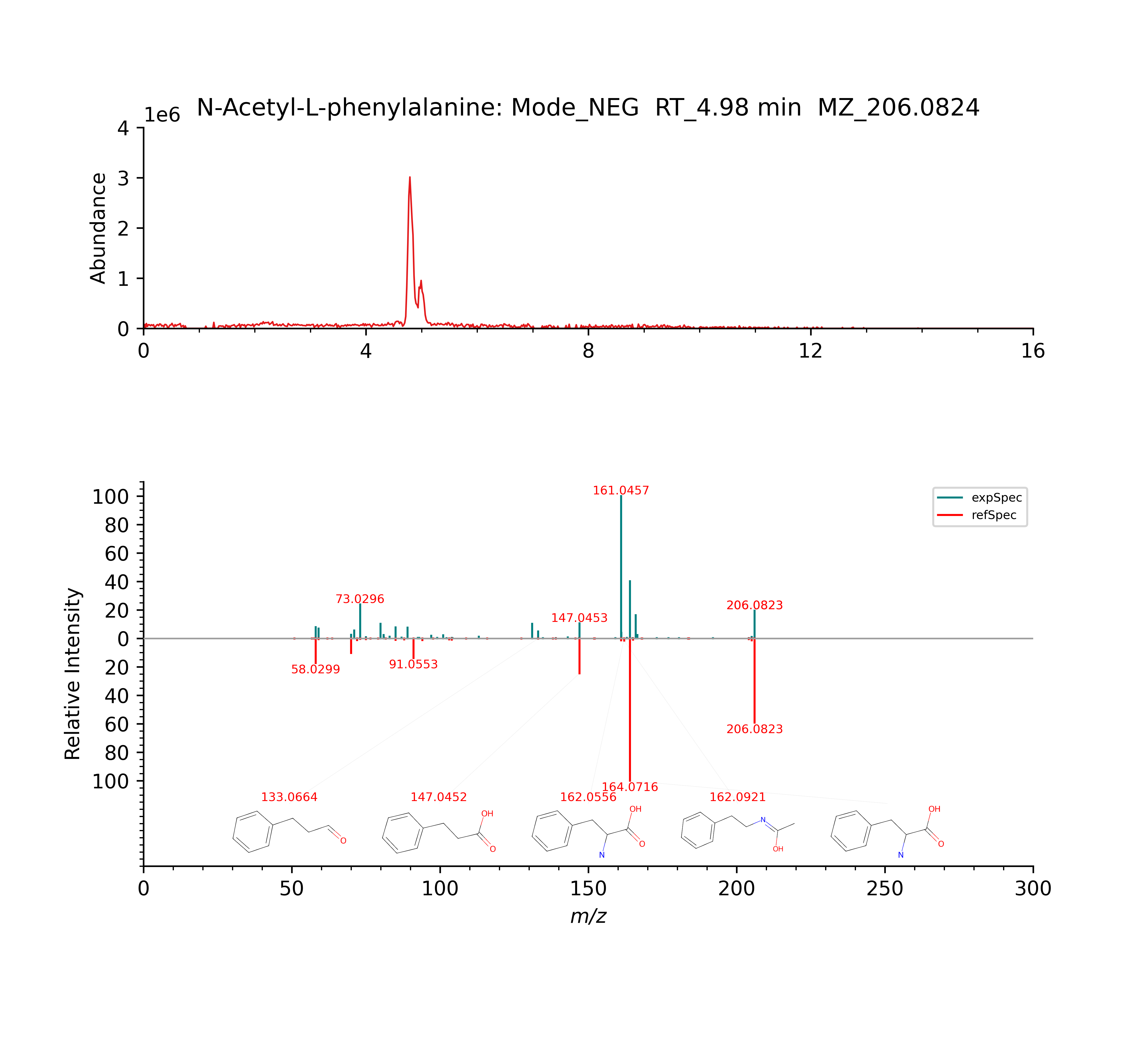

Supplement: Supplementary file 1 [file molecules-29-02840-s001.zip › Supplementary Figure s1/Identification from LuMet-CM datebase/png/compound00071.png]

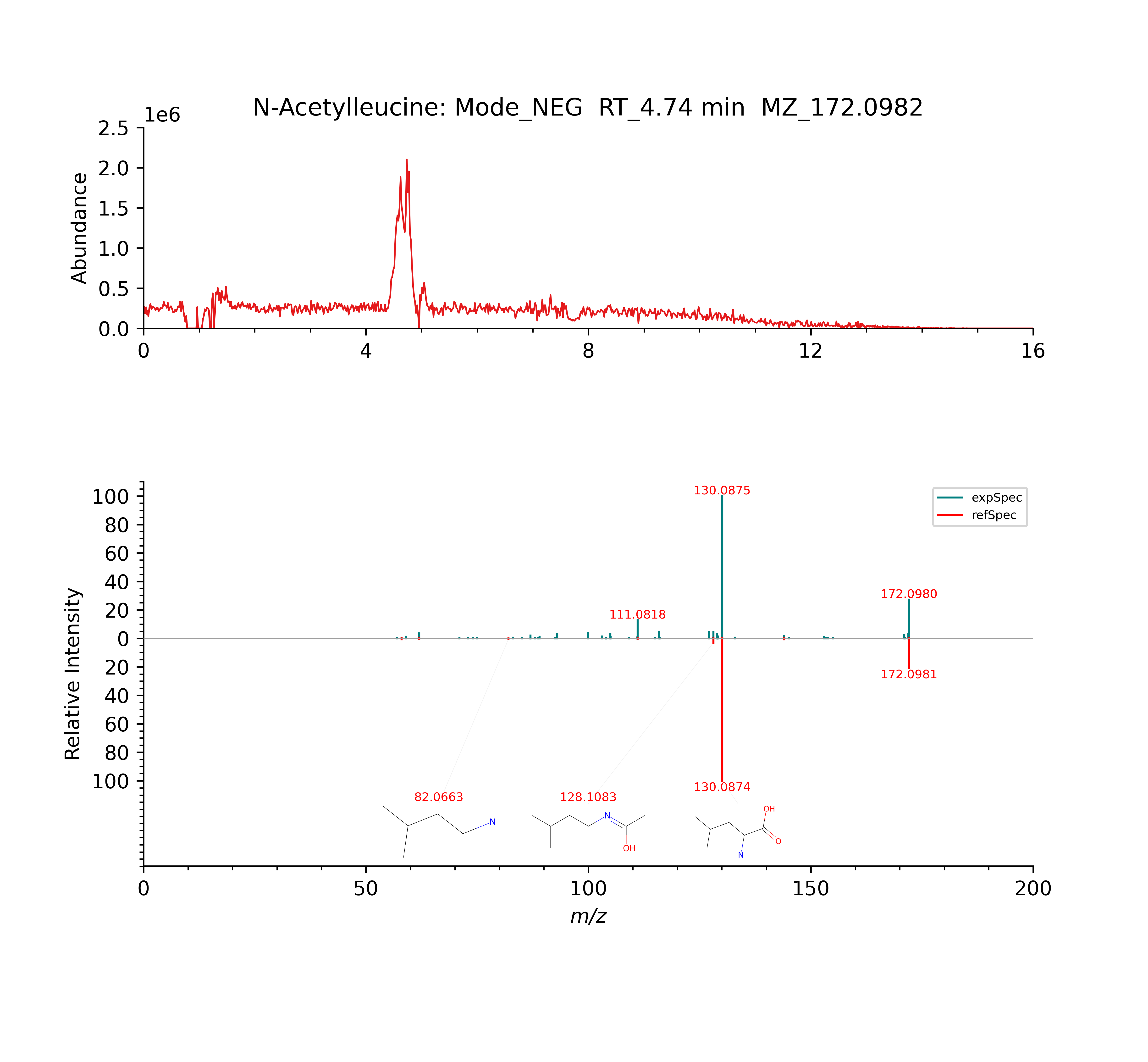

Supplement: Supplementary file 1 [file molecules-29-02840-s001.zip › Supplementary Figure s1/Identification from LuMet-CM datebase/png/compound00072.png]

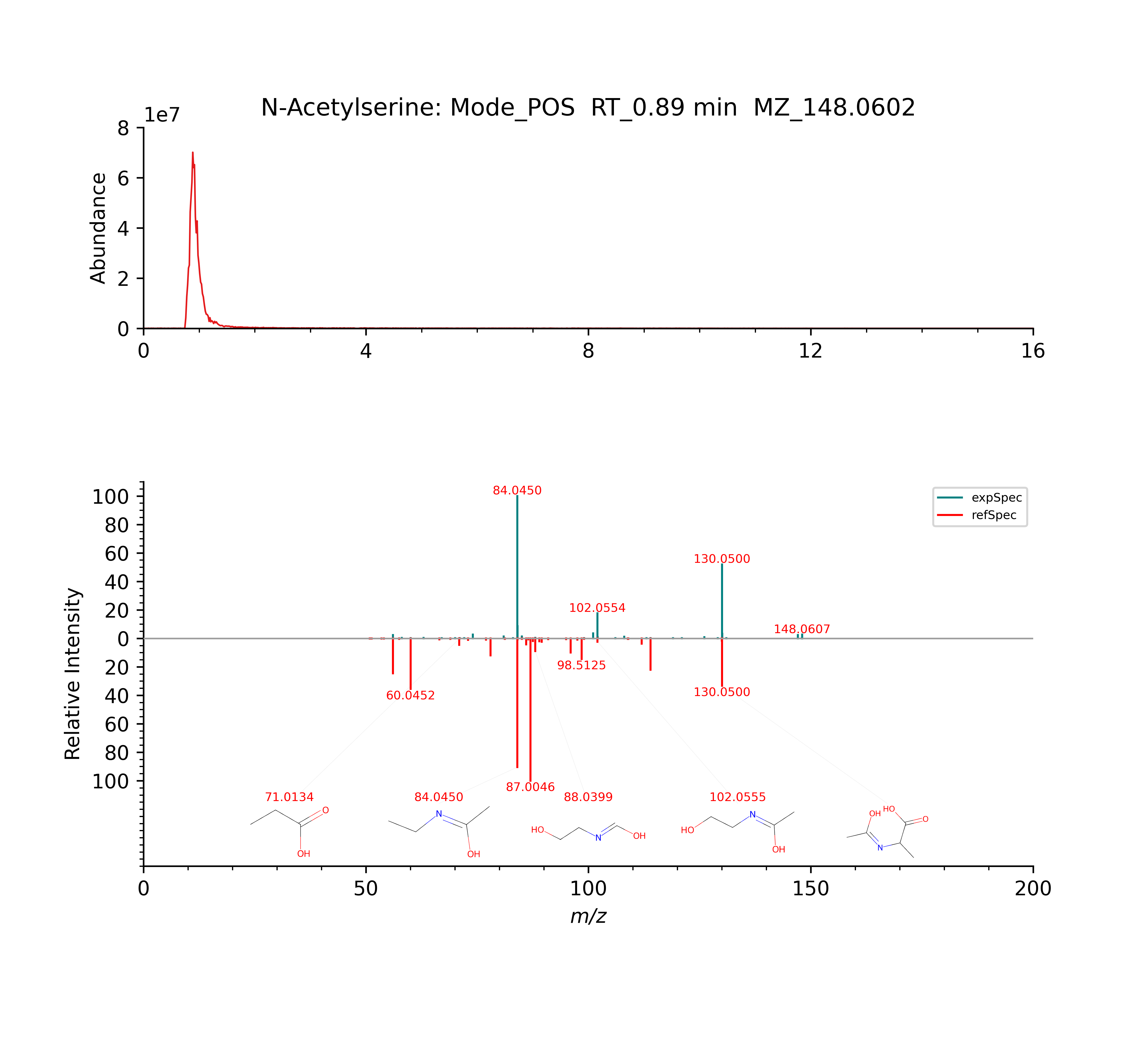

Supplement: Supplementary file 1 [file molecules-29-02840-s001.zip › Supplementary Figure s1/Identification from LuMet-CM datebase/png/compound00073.png]

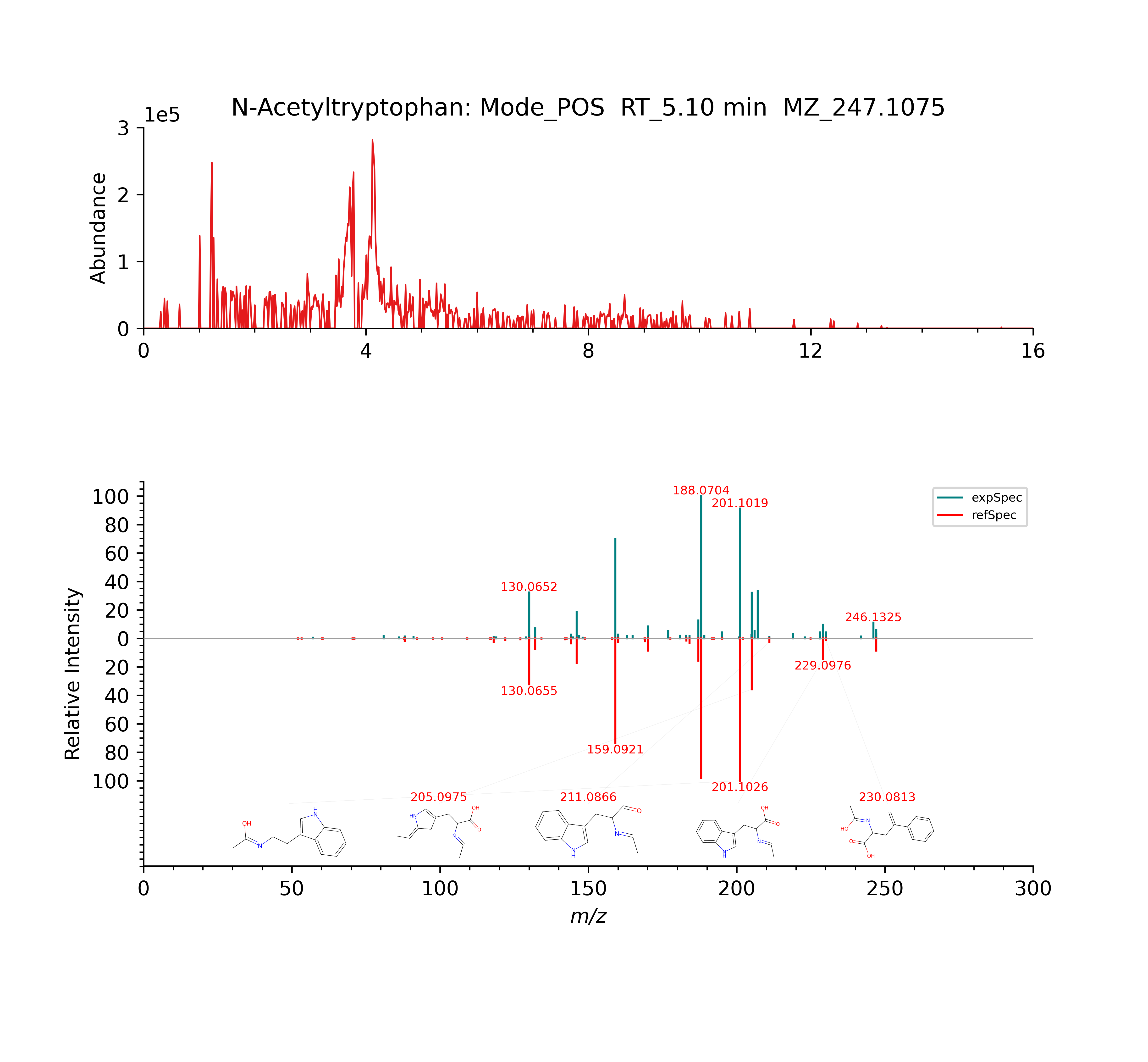

Supplement: Supplementary file 1 [file molecules-29-02840-s001.zip › Supplementary Figure s1/Identification from LuMet-CM datebase/png/compound00074.png]

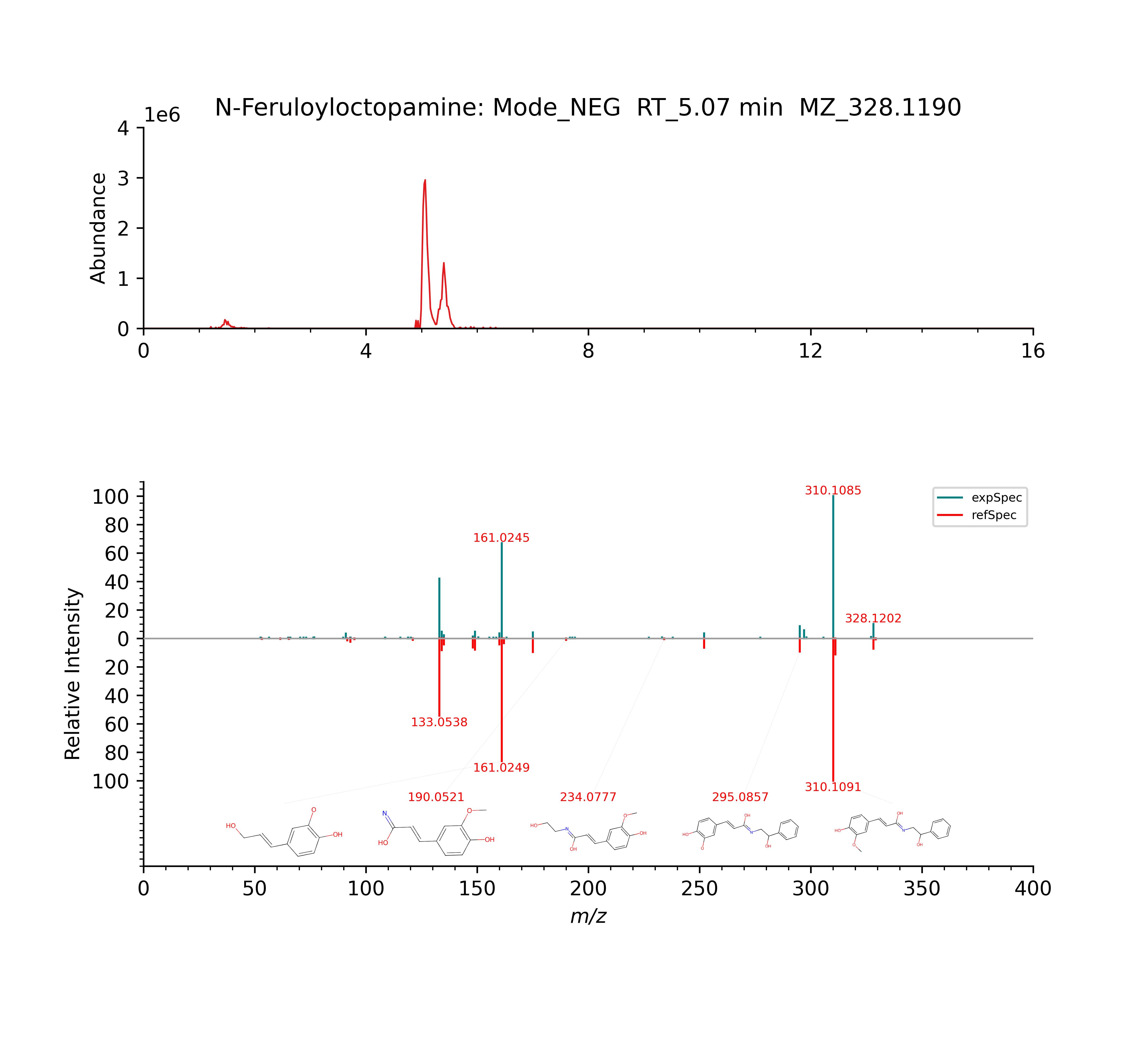

Supplement: Supplementary file 1 [file molecules-29-02840-s001.zip › Supplementary Figure s1/Identification from LuMet-CM datebase/png/compound00075.png]

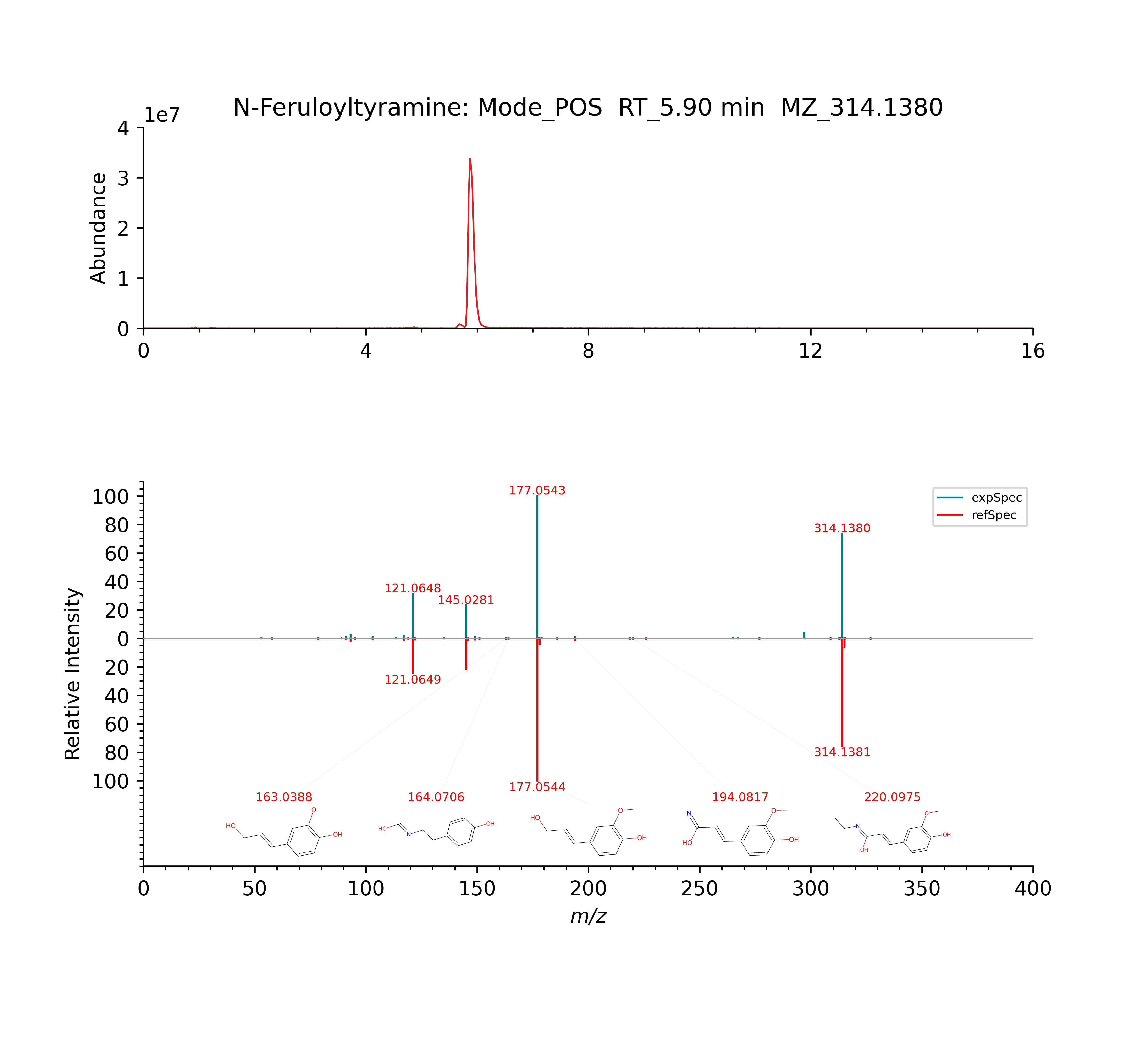

Supplement: Supplementary file 1 [file molecules-29-02840-s001.zip › Supplementary Figure s1/Identification from LuMet-CM datebase/png/compound00076.png]

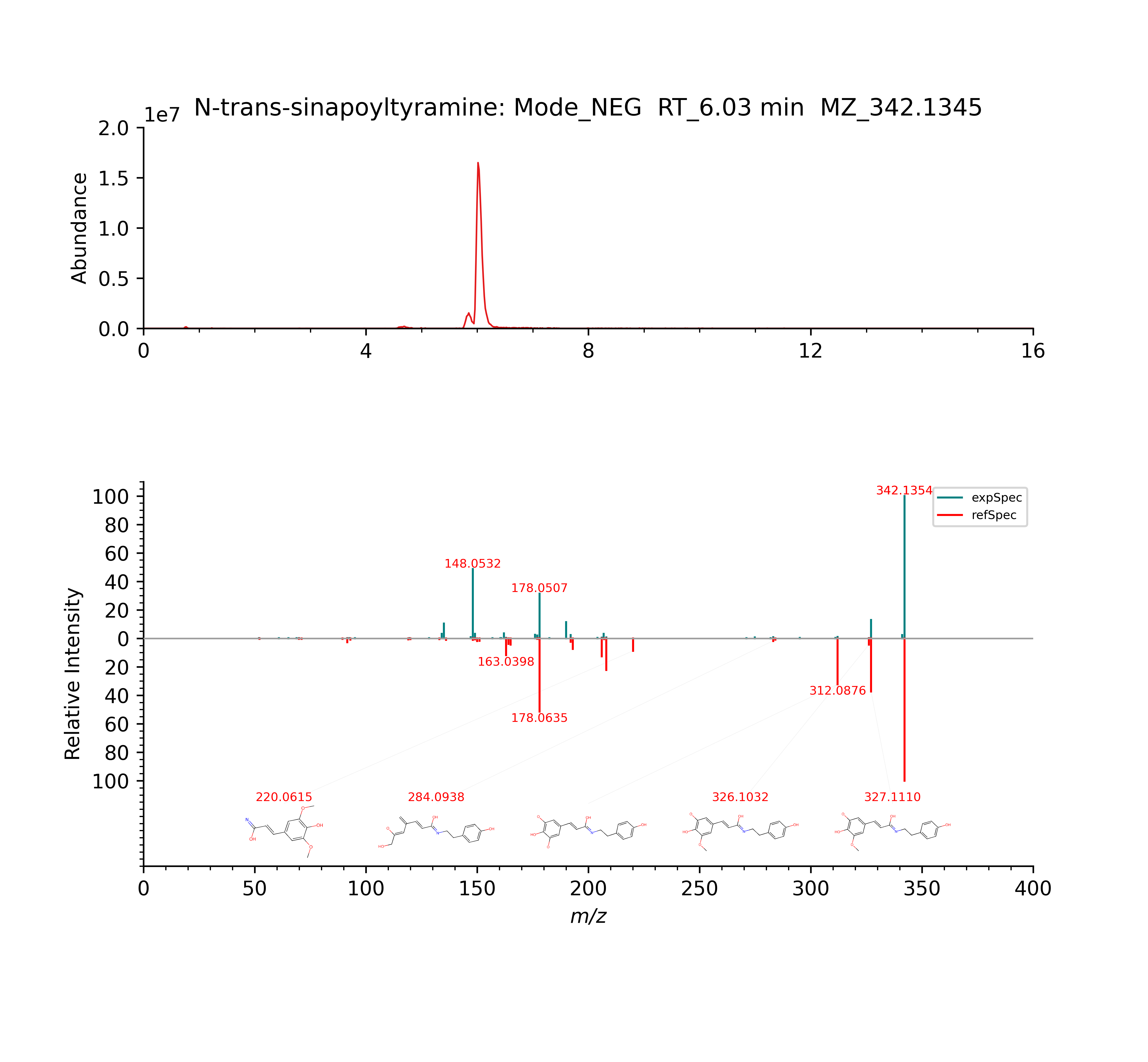

Supplement: Supplementary file 1 [file molecules-29-02840-s001.zip › Supplementary Figure s1/Identification from LuMet-CM datebase/png/compound00077.png]

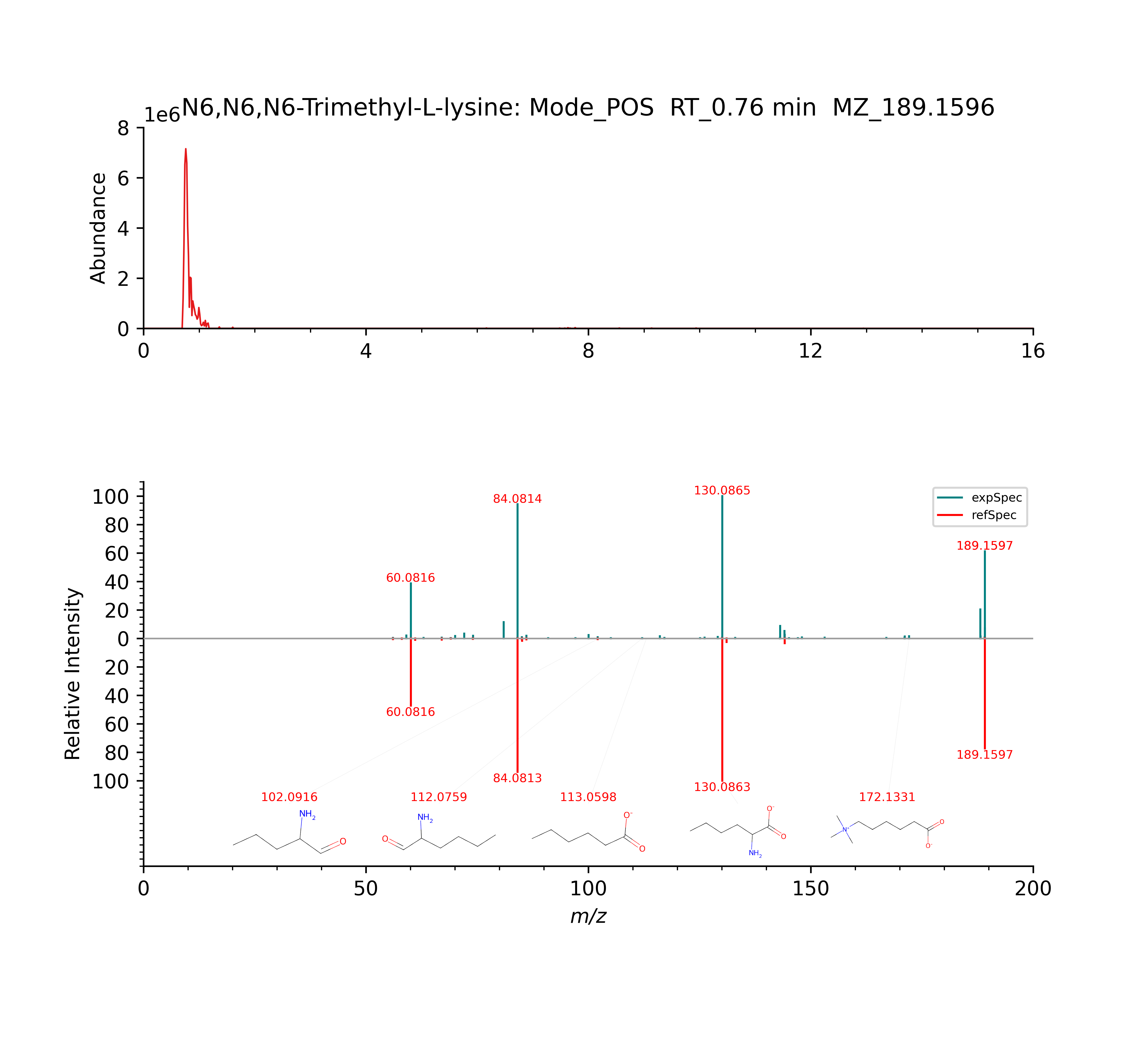

Supplement: Supplementary file 1 [file molecules-29-02840-s001.zip › Supplementary Figure s1/Identification from LuMet-CM datebase/png/compound00078.png]

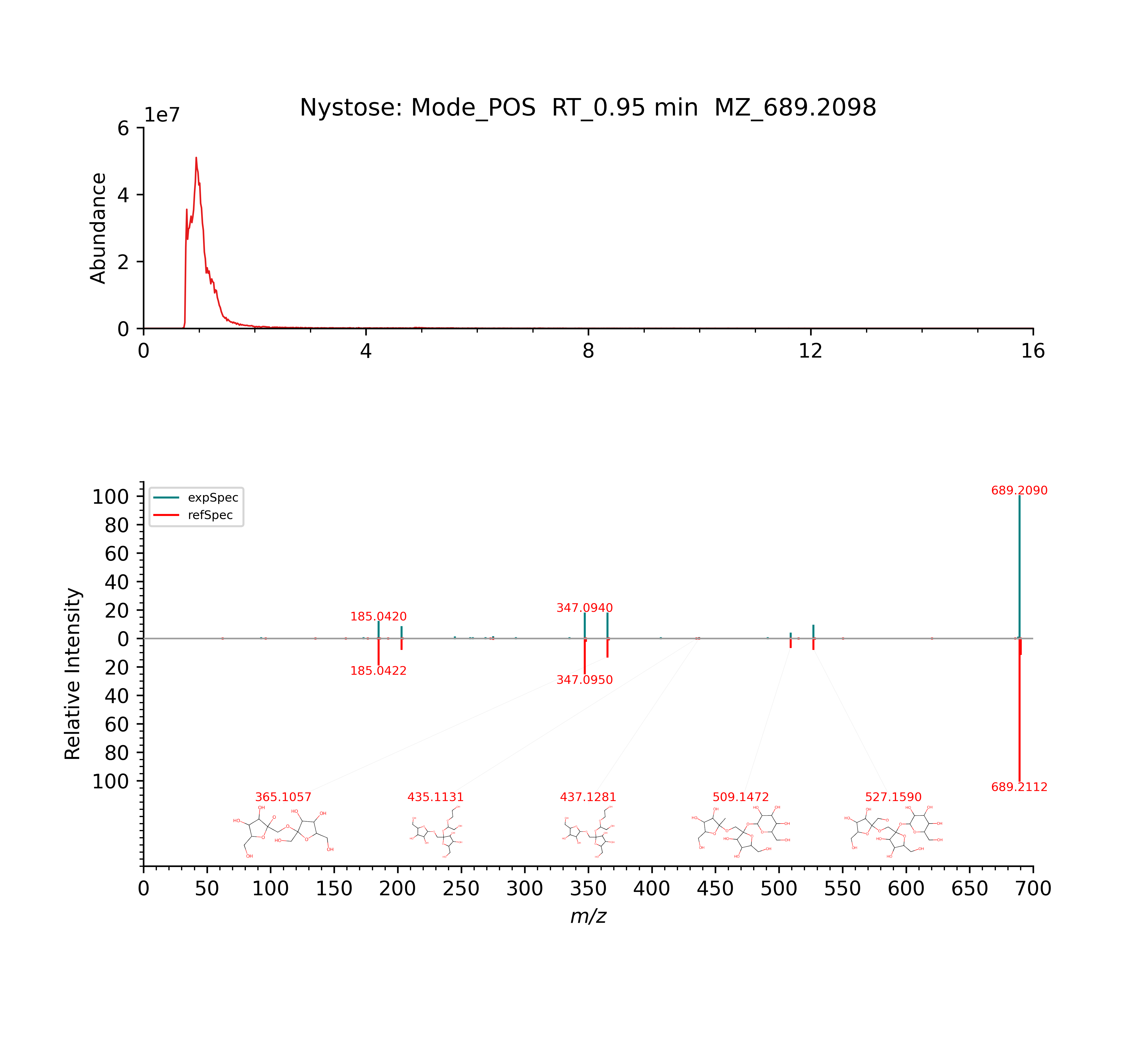

Supplement: Supplementary file 1 [file molecules-29-02840-s001.zip › Supplementary Figure s1/Identification from LuMet-CM datebase/png/compound00079.png]

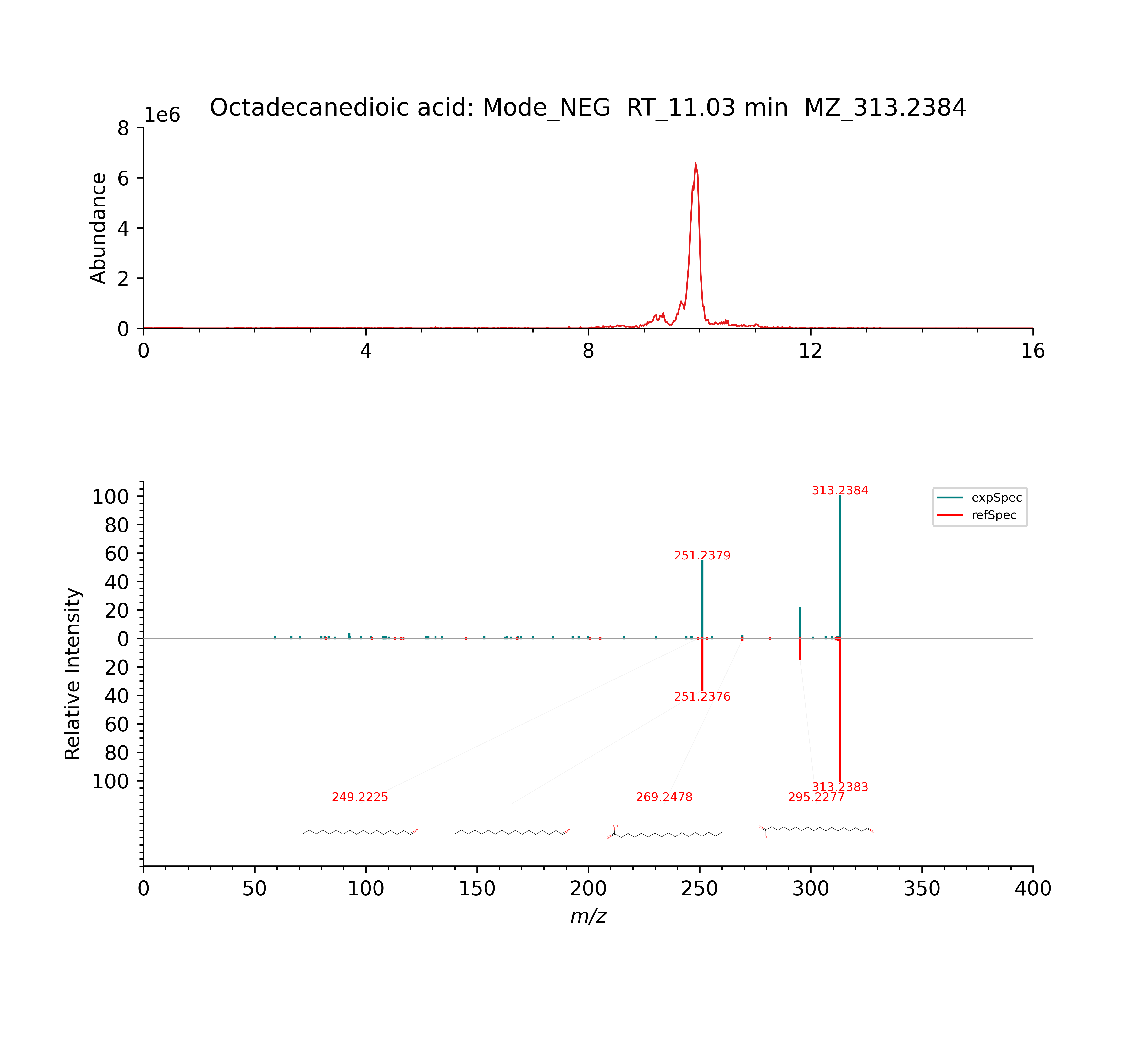

Supplement: Supplementary file 1 [file molecules-29-02840-s001.zip › Supplementary Figure s1/Identification from LuMet-CM datebase/png/compound00080.png]

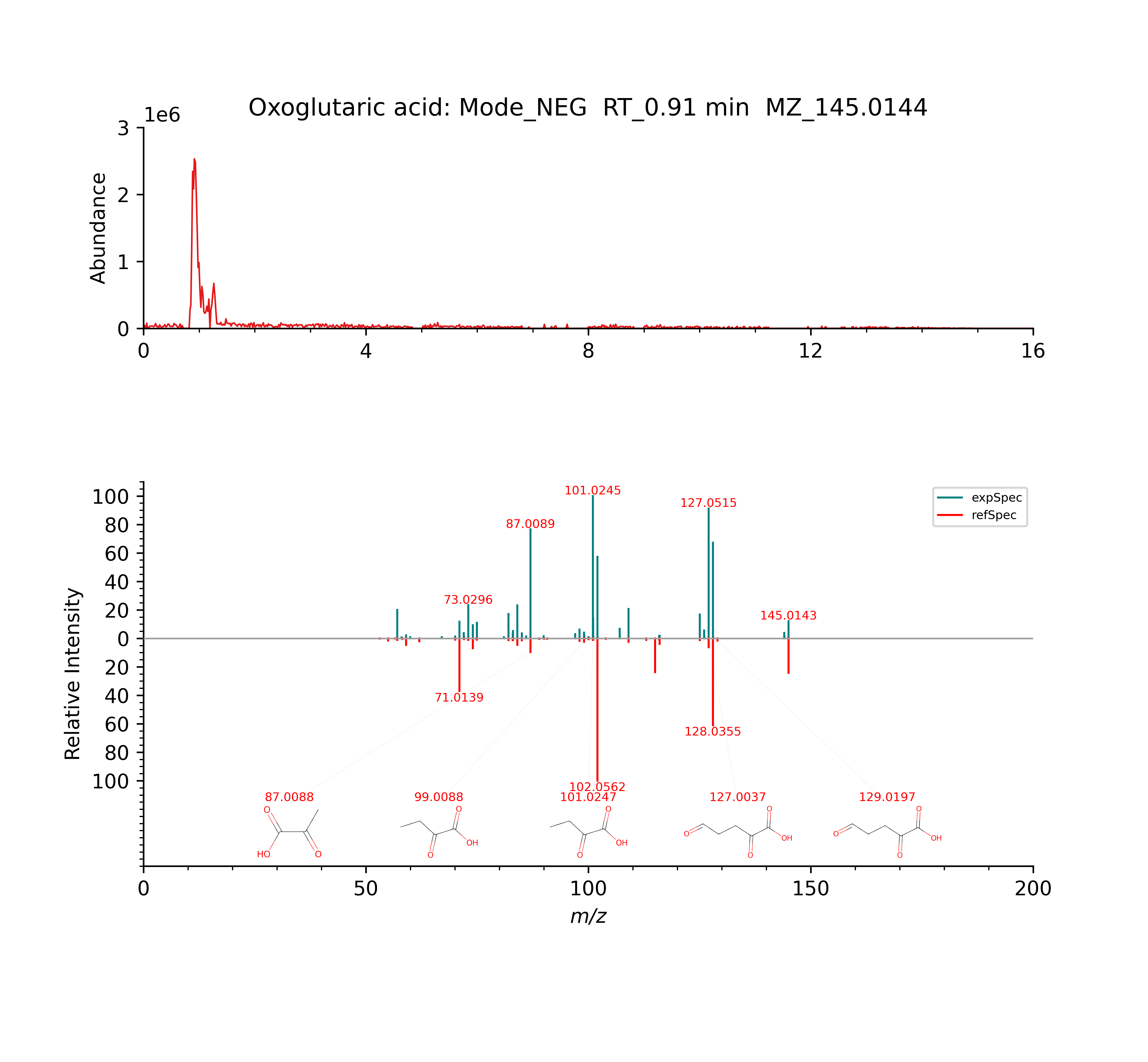

Supplement: Supplementary file 1 [file molecules-29-02840-s001.zip › Supplementary Figure s1/Identification from LuMet-CM datebase/png/compound00081.png]

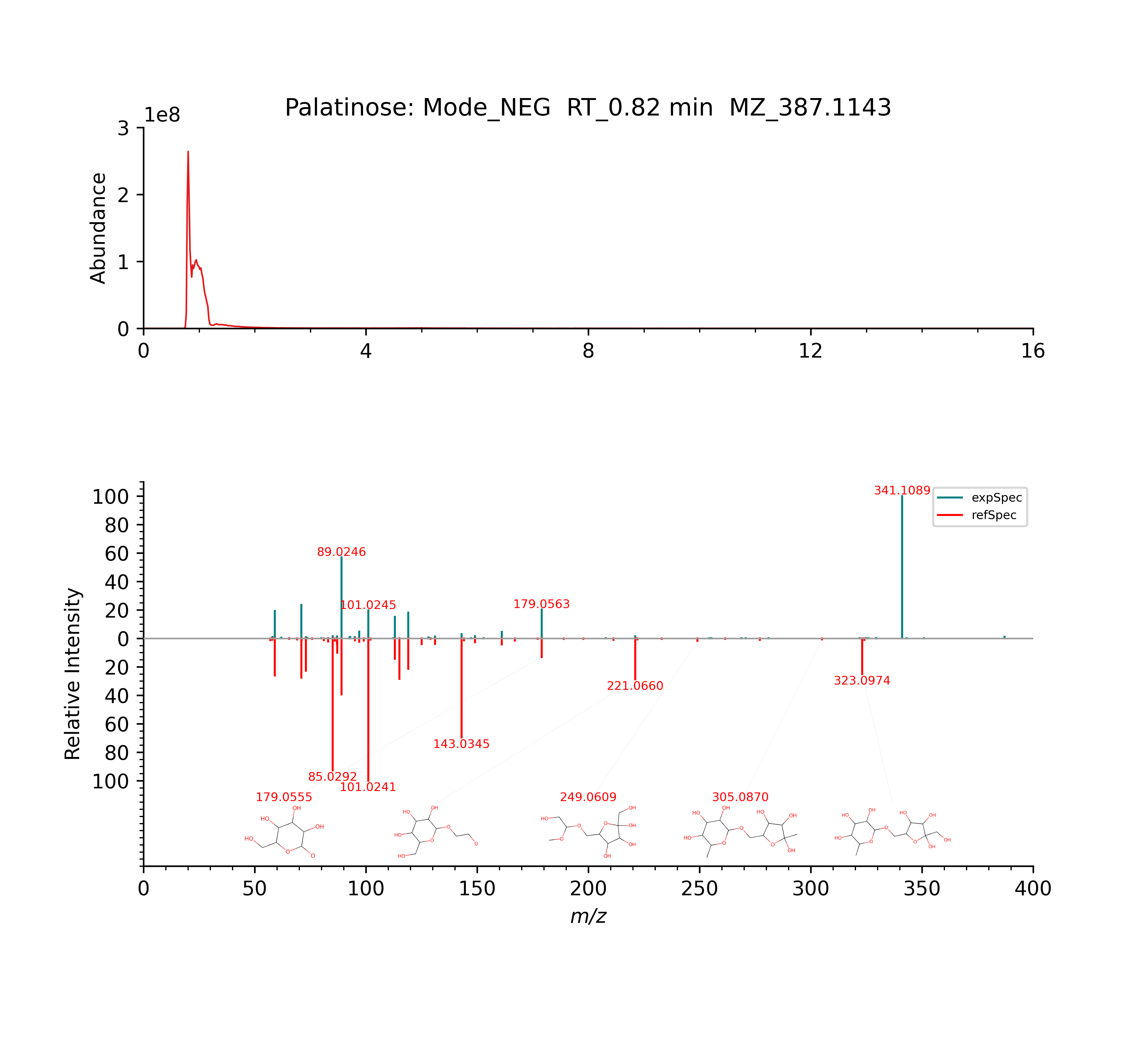

Supplement: Supplementary file 1 [file molecules-29-02840-s001.zip › Supplementary Figure s1/Identification from LuMet-CM datebase/png/compound00082.png]

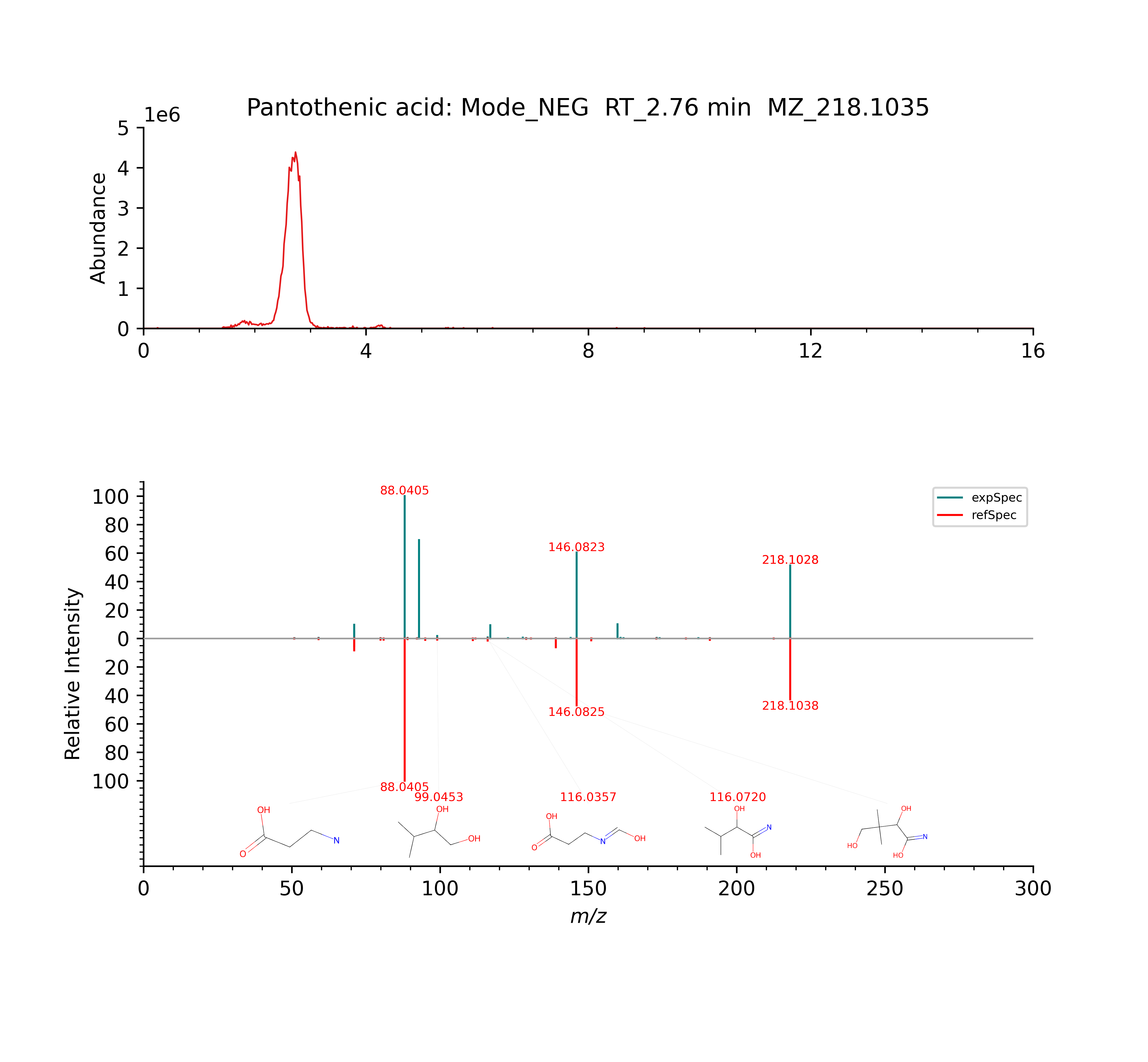

Supplement: Supplementary file 1 [file molecules-29-02840-s001.zip › Supplementary Figure s1/Identification from LuMet-CM datebase/png/compound00083.png]

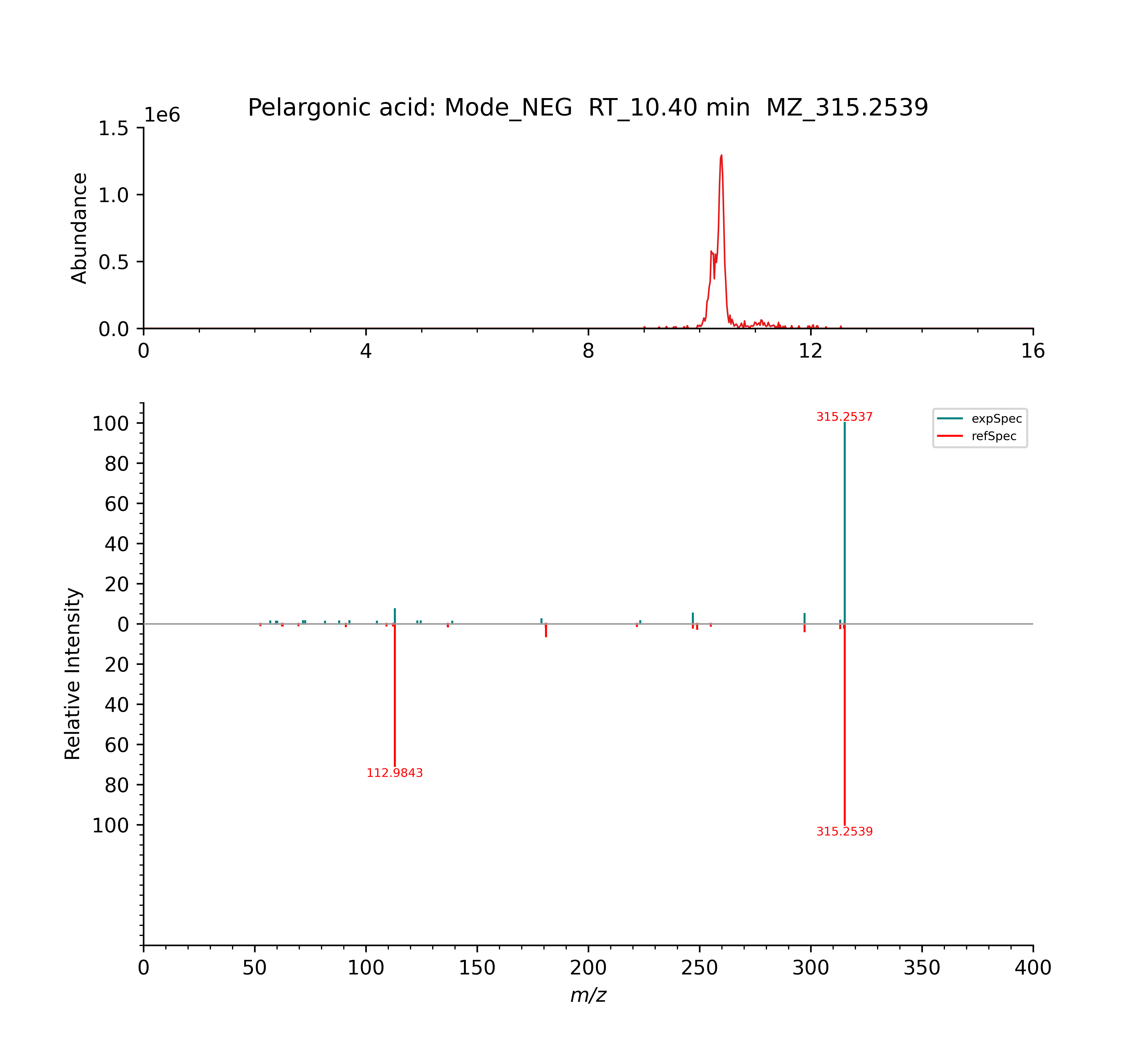

Supplement: Supplementary file 1 [file molecules-29-02840-s001.zip › Supplementary Figure s1/Identification from LuMet-CM datebase/png/compound00084.png]

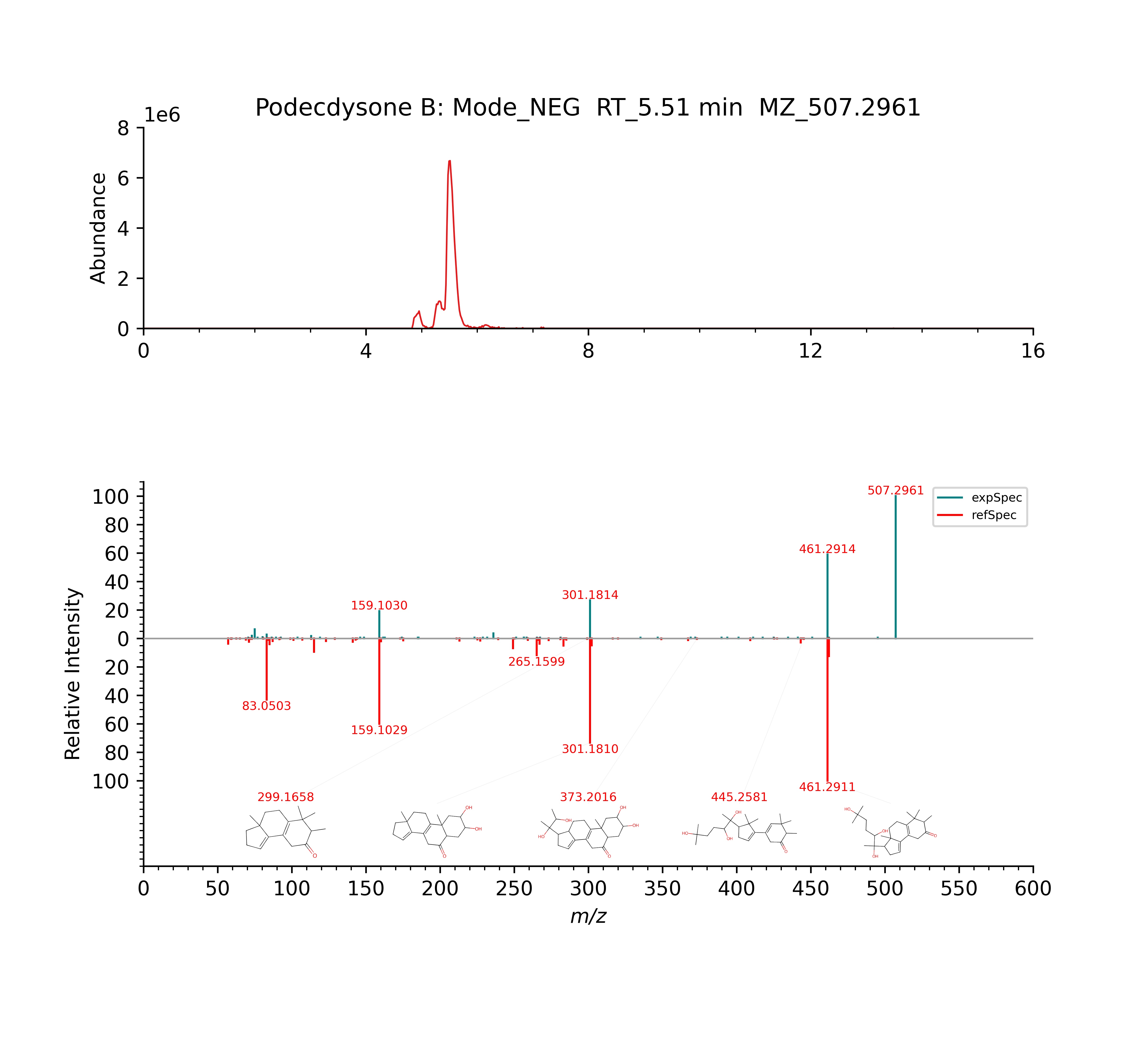

Supplement: Supplementary file 1 [file molecules-29-02840-s001.zip › Supplementary Figure s1/Identification from LuMet-CM datebase/png/compound00085.png]

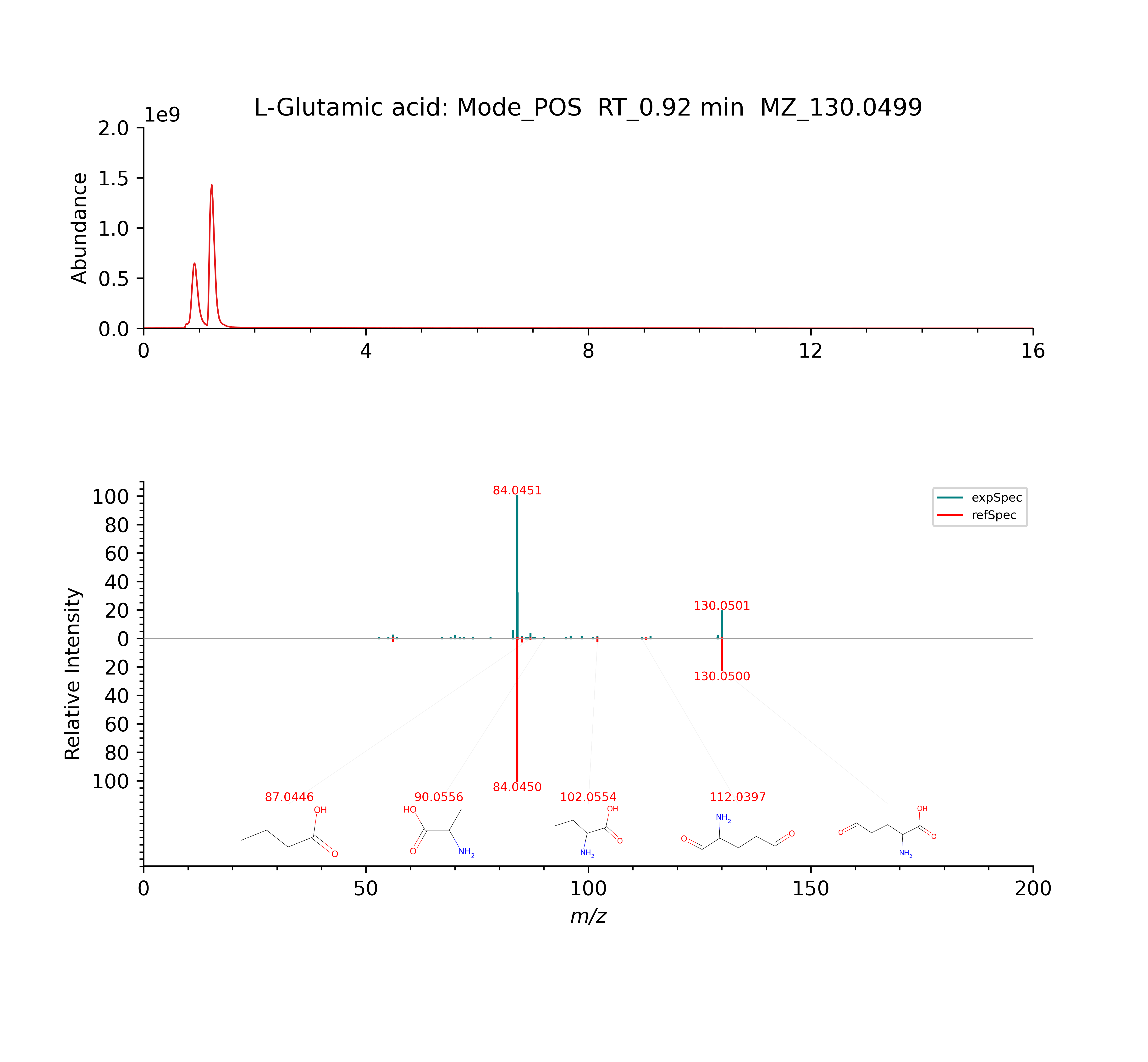

Supplement: Supplementary file 1 [file molecules-29-02840-s001.zip › Supplementary Figure s1/Identification from LuMet-CM datebase/png/compound00086.png]

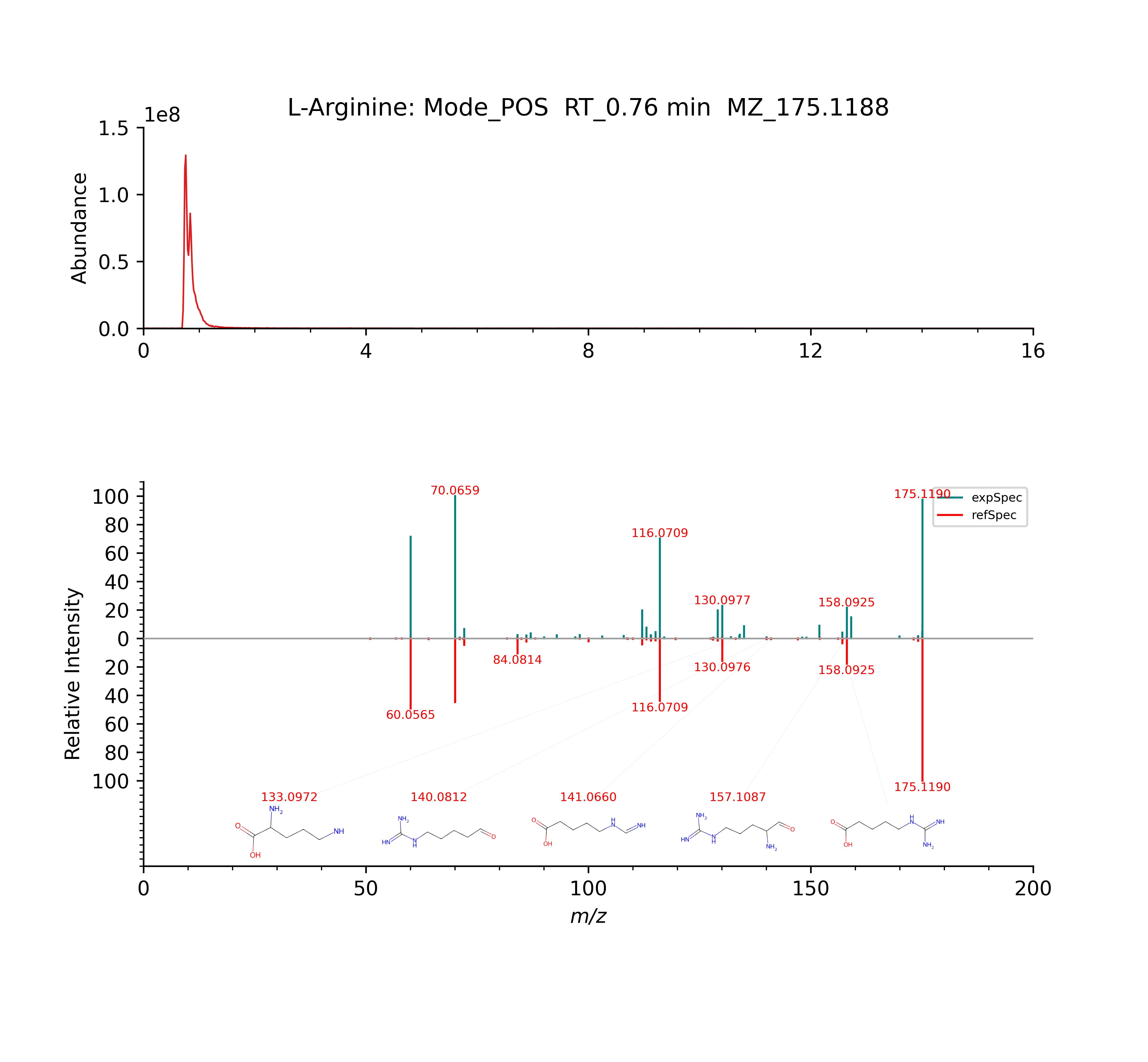

Supplement: Supplementary file 1 [file molecules-29-02840-s001.zip › Supplementary Figure s1/Identification from LuMet-CM datebase/png/compound00087.png]

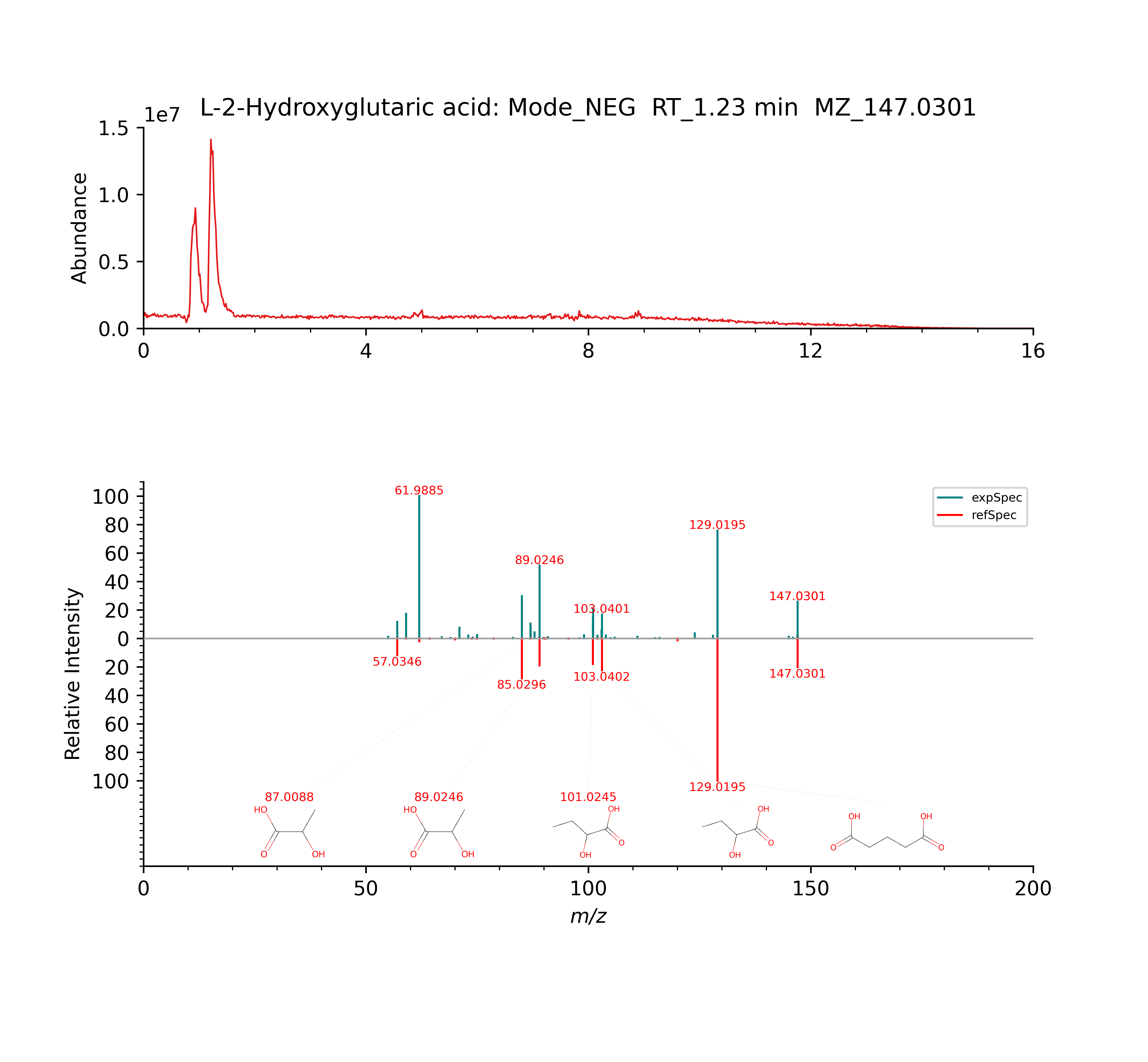

Supplement: Supplementary file 1 [file molecules-29-02840-s001.zip › Supplementary Figure s1/Identification from LuMet-CM datebase/png/compound00088.png]

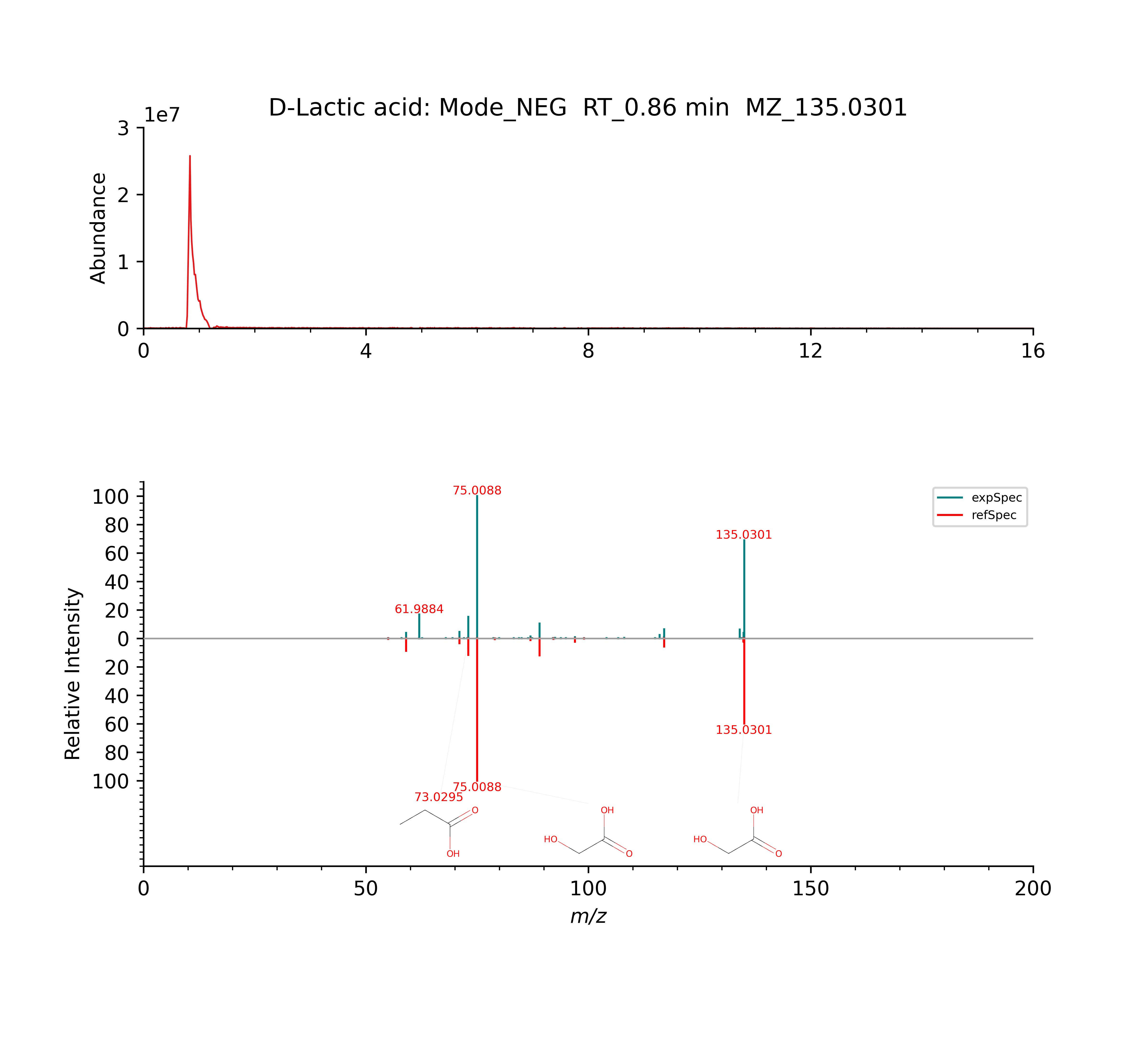

Supplement: Supplementary file 1 [file molecules-29-02840-s001.zip › Supplementary Figure s1/Identification from LuMet-CM datebase/png/compound00089.png]

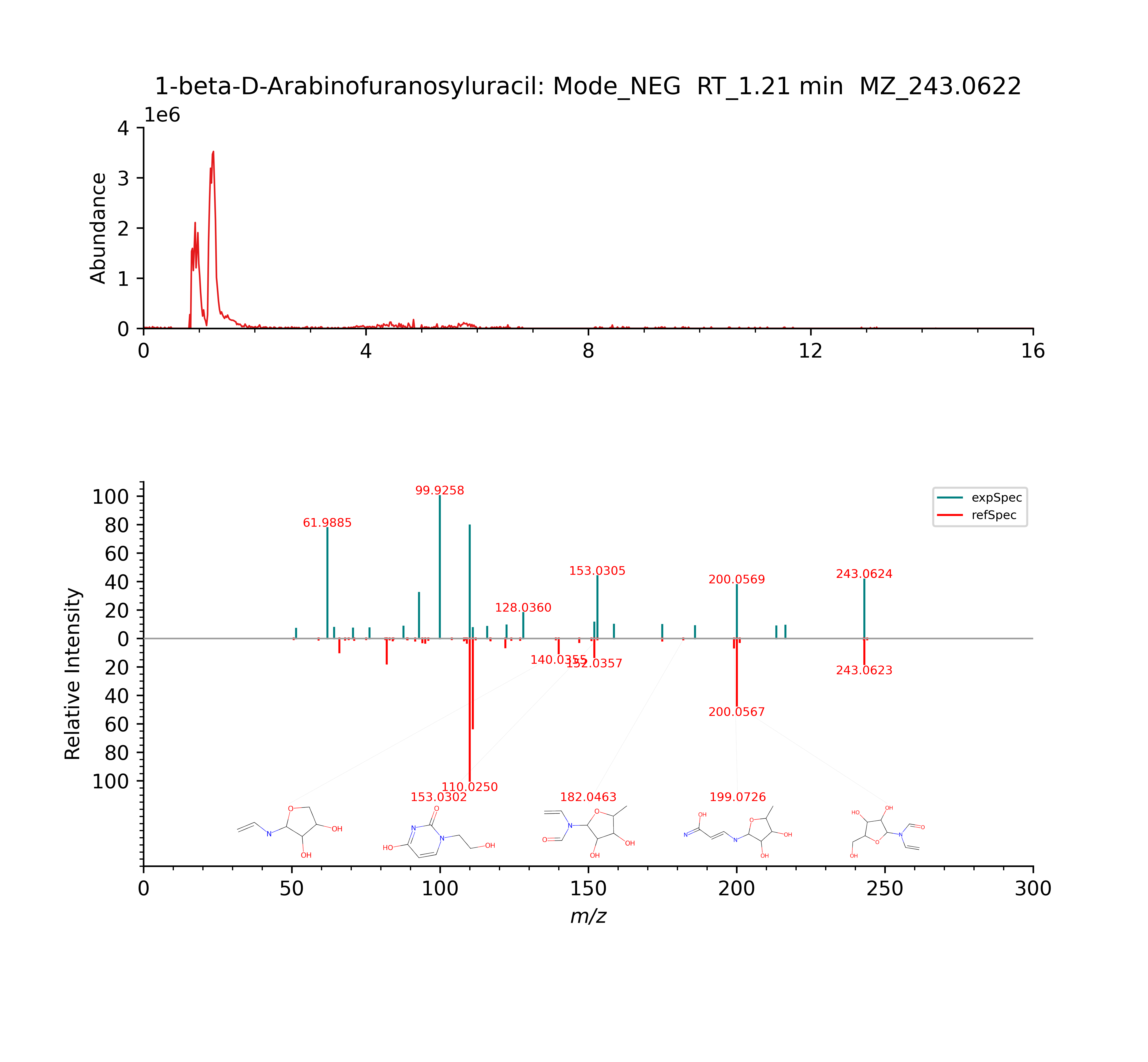

Supplement: Supplementary file 1 [file molecules-29-02840-s001.zip › Supplementary Figure s1/Identification from LuMet-CM datebase/png/compound00090.png]

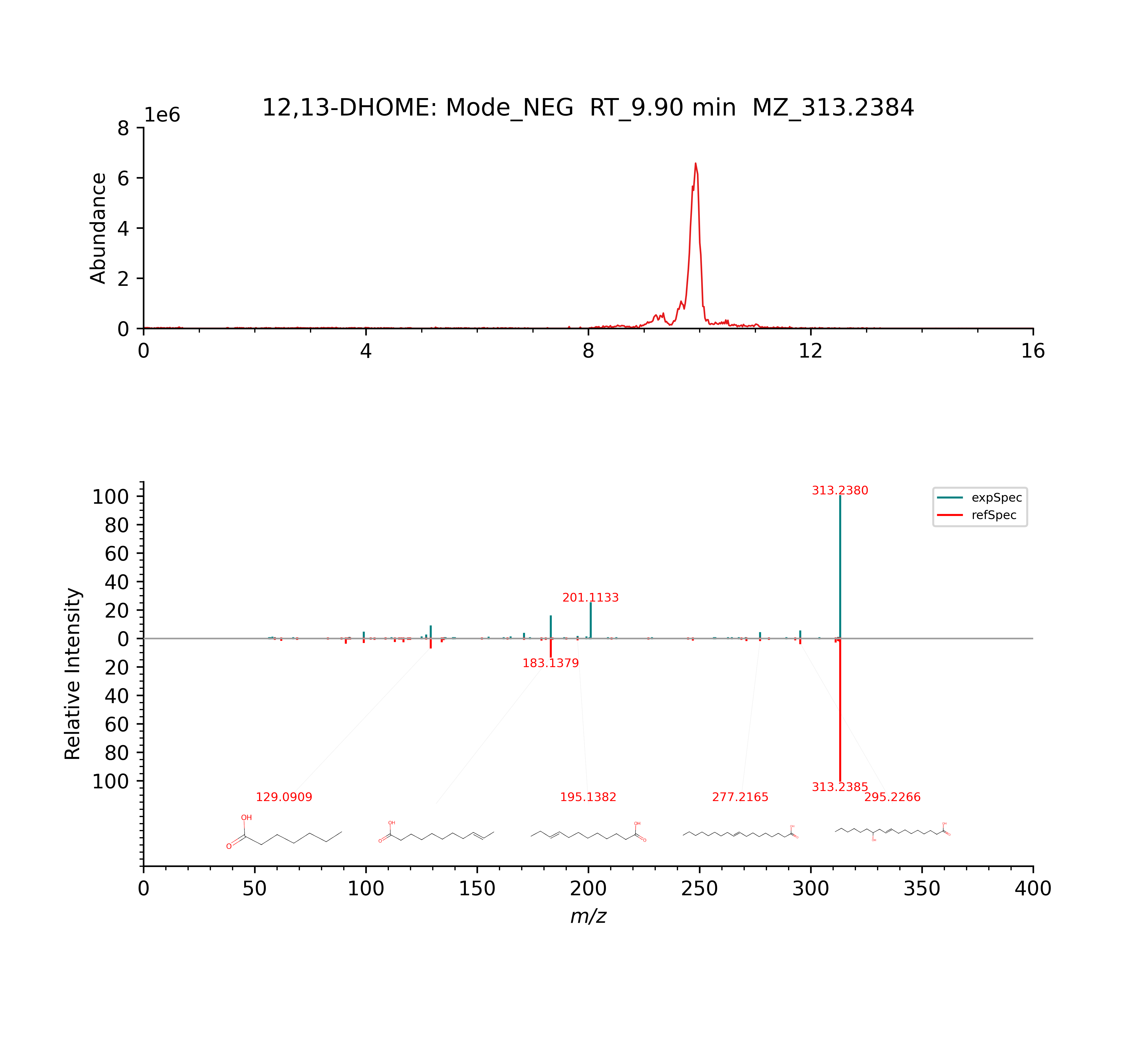

Supplement: Supplementary file 1 [file molecules-29-02840-s001.zip › Supplementary Figure s1/Identification from LuMet-CM datebase/png/compound00091.png]

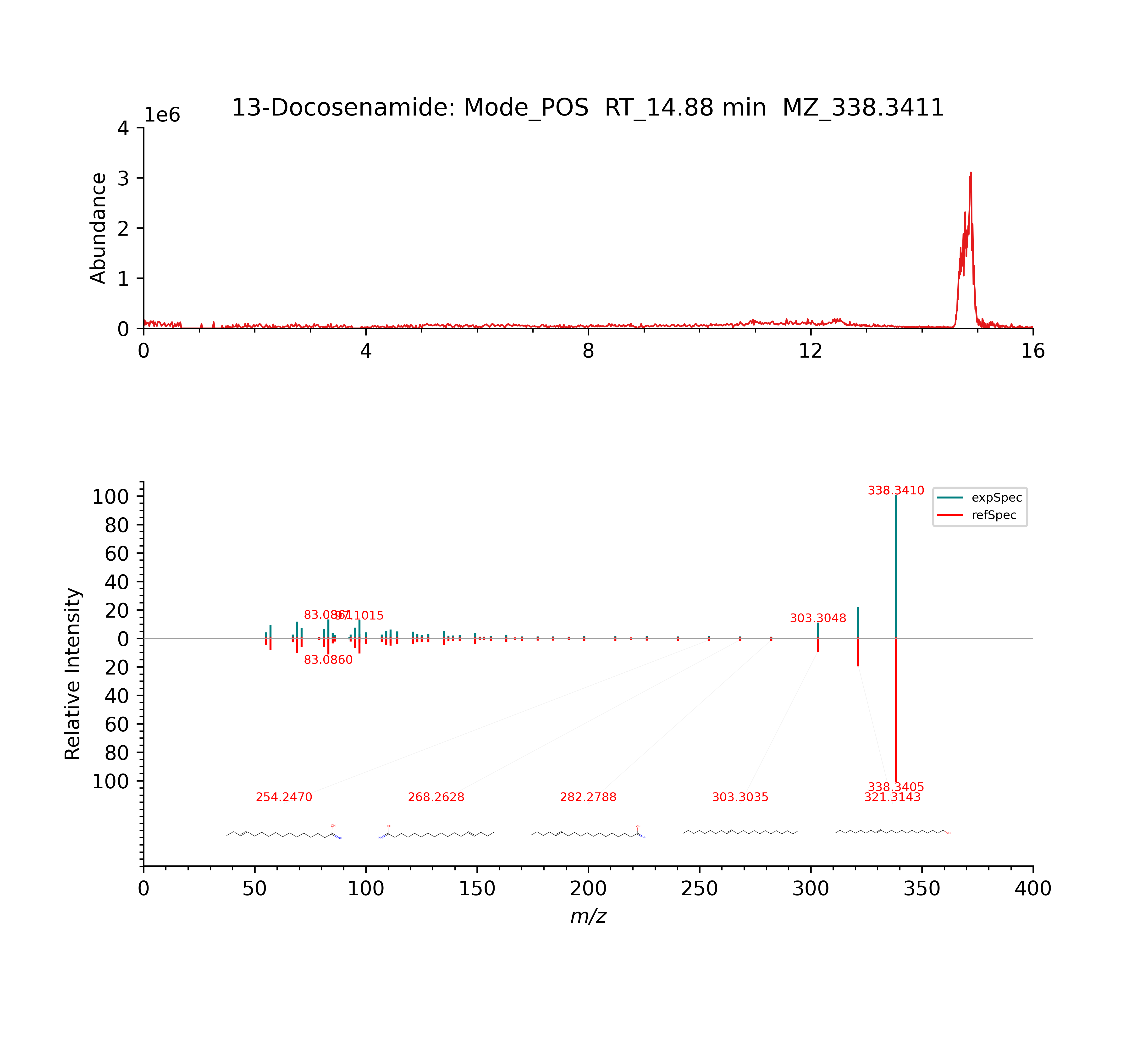

Supplement: Supplementary file 1 [file molecules-29-02840-s001.zip › Supplementary Figure s1/Identification from LuMet-CM datebase/png/compound00092.png]

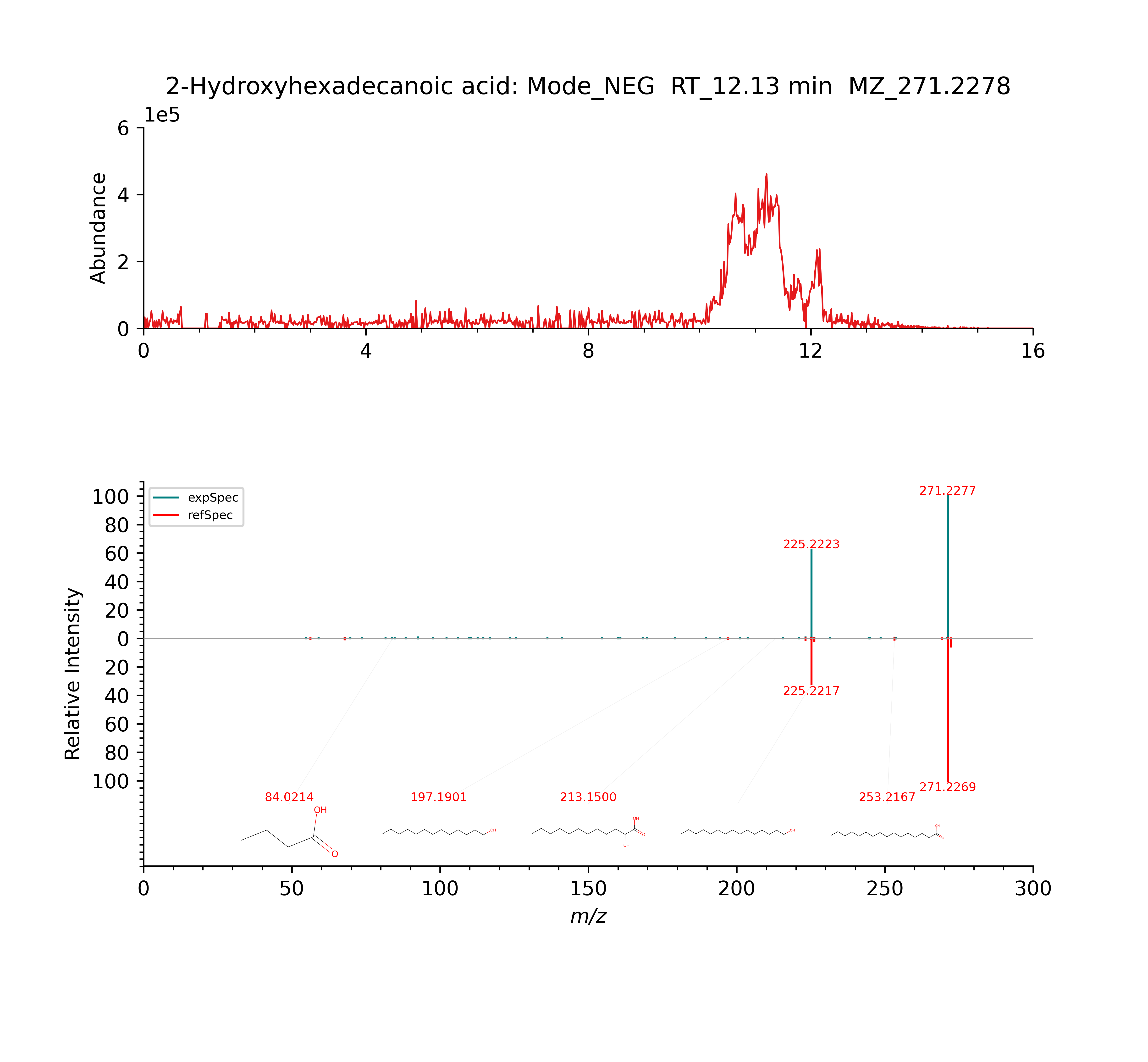

Supplement: Supplementary file 1 [file molecules-29-02840-s001.zip › Supplementary Figure s1/Identification from LuMet-CM datebase/png/compound00093.png]

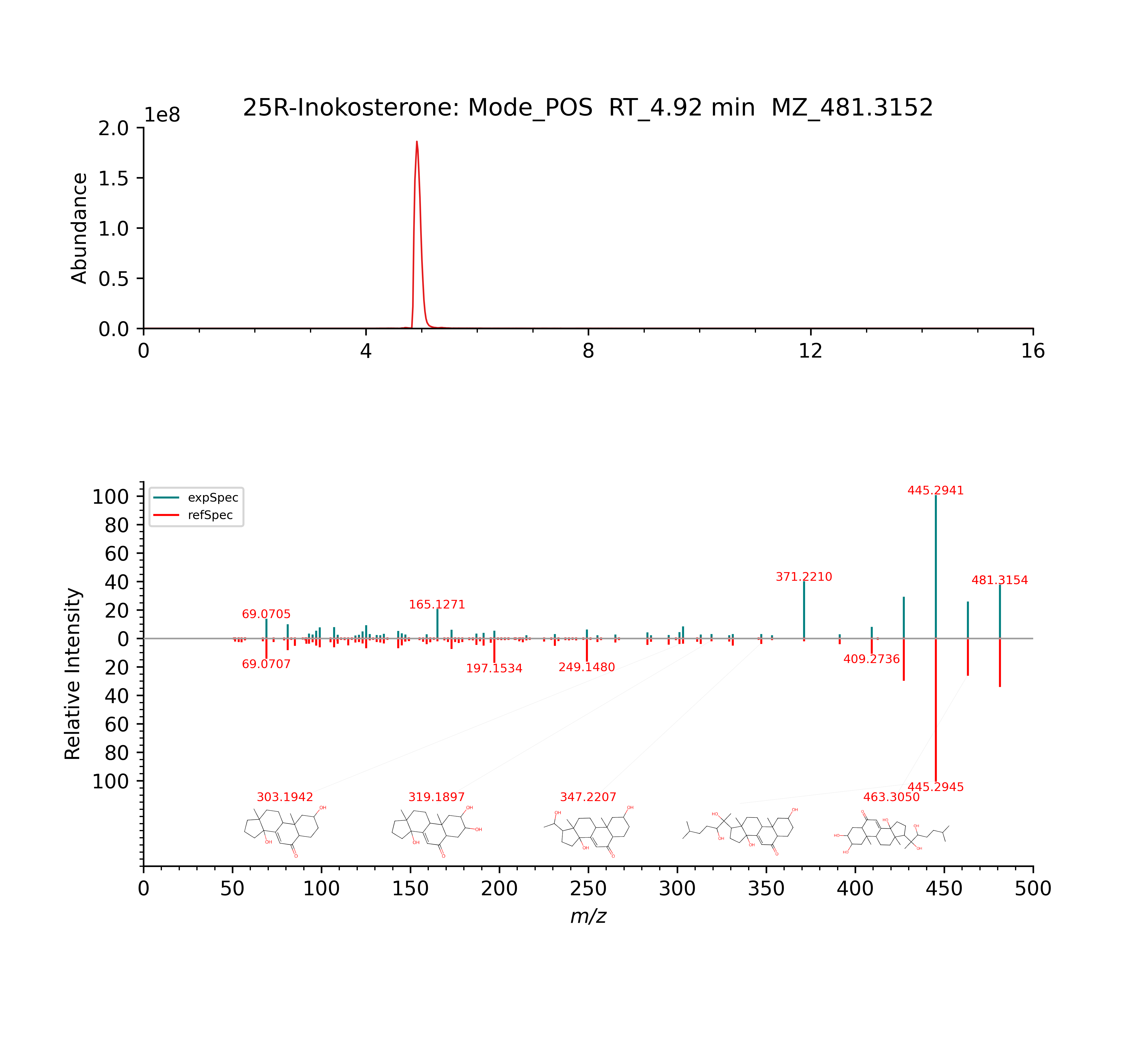

Supplement: Supplementary file 1 [file molecules-29-02840-s001.zip › Supplementary Figure s1/Identification from LuMet-CM datebase/png/compound00094.png]

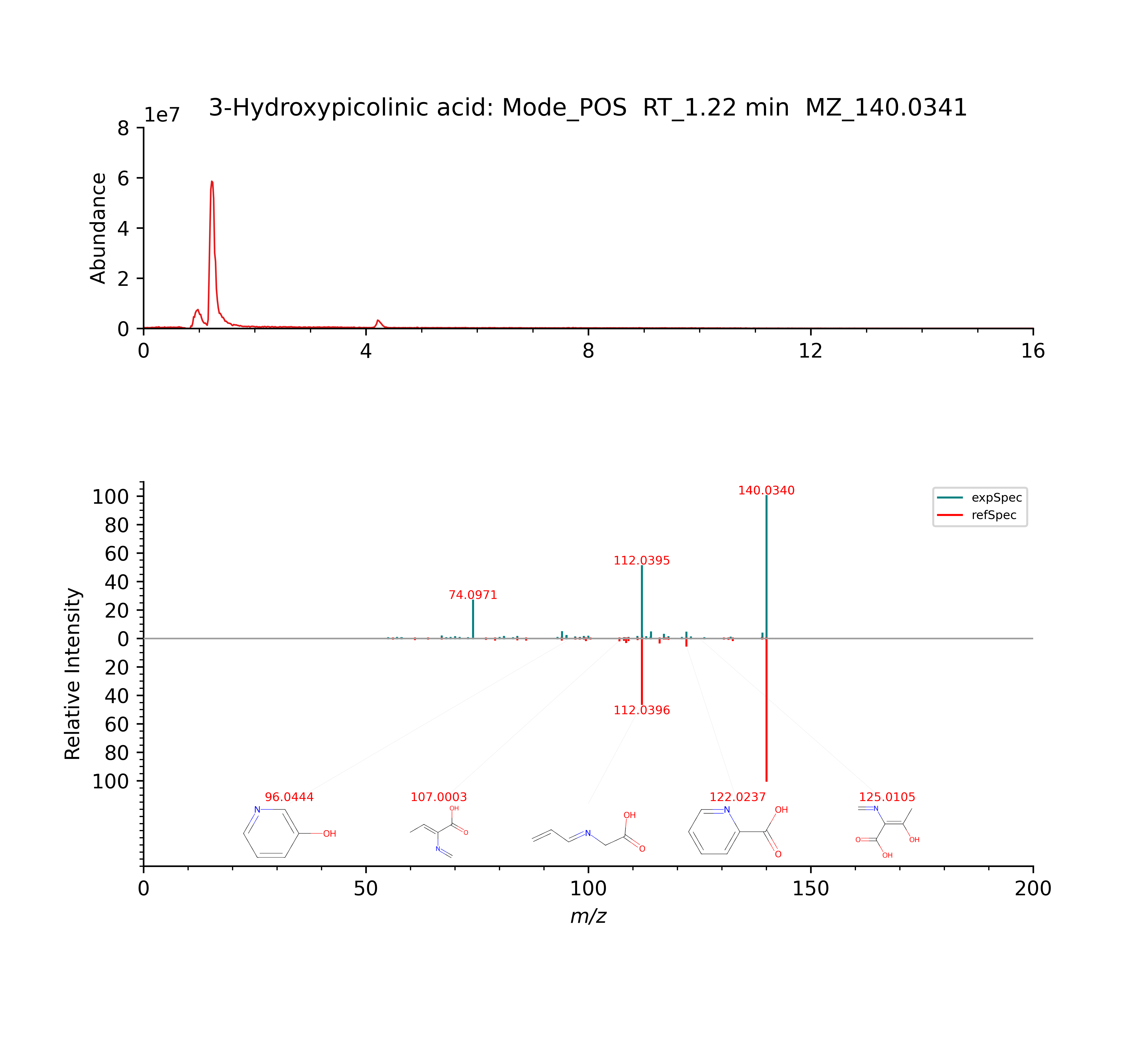

Supplement: Supplementary file 1 [file molecules-29-02840-s001.zip › Supplementary Figure s1/Identification from LuMet-CM datebase/png/compound00095.png]

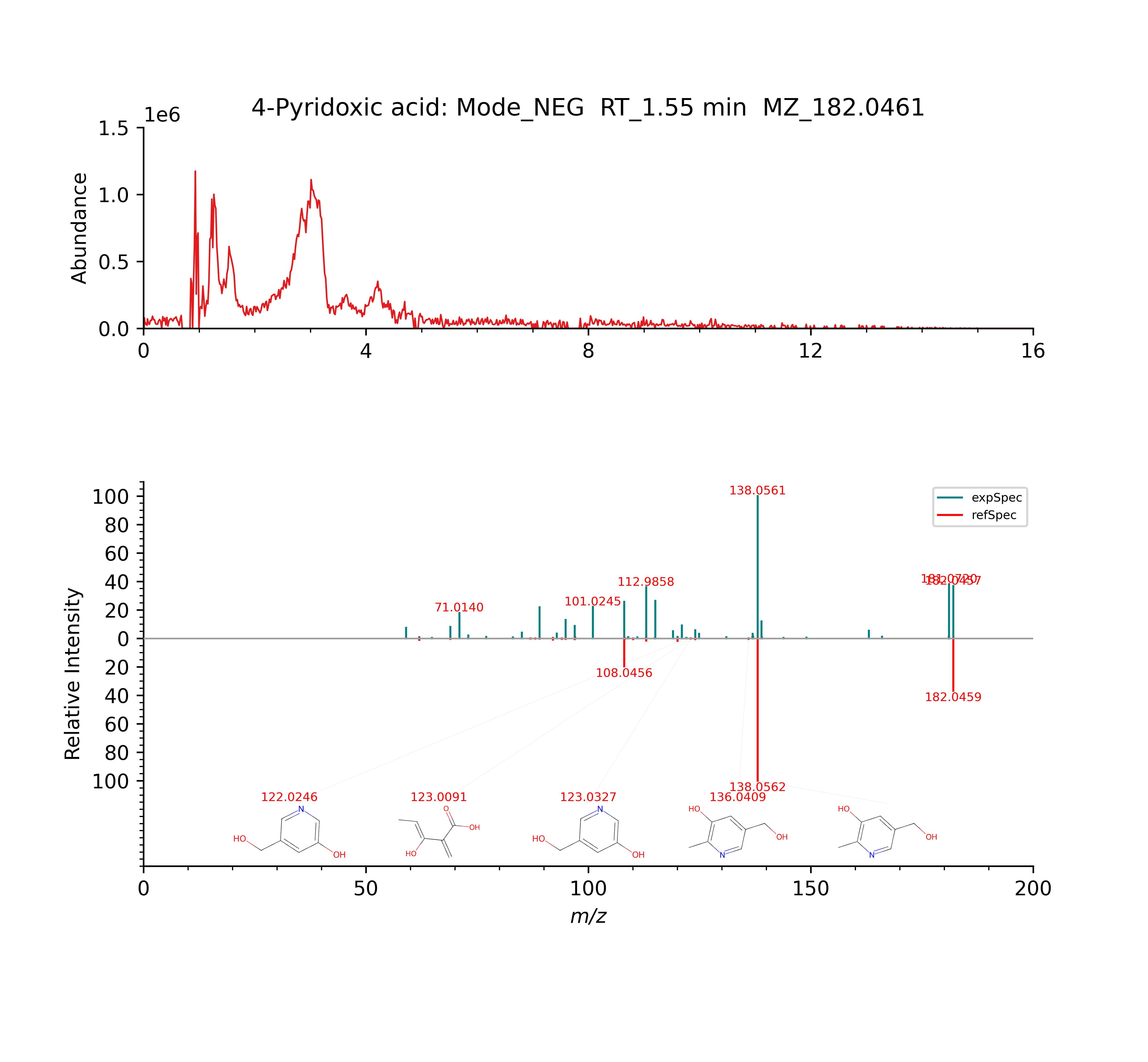

Supplement: Supplementary file 1 [file molecules-29-02840-s001.zip › Supplementary Figure s1/Identification from LuMet-CM datebase/png/compound00096.png]

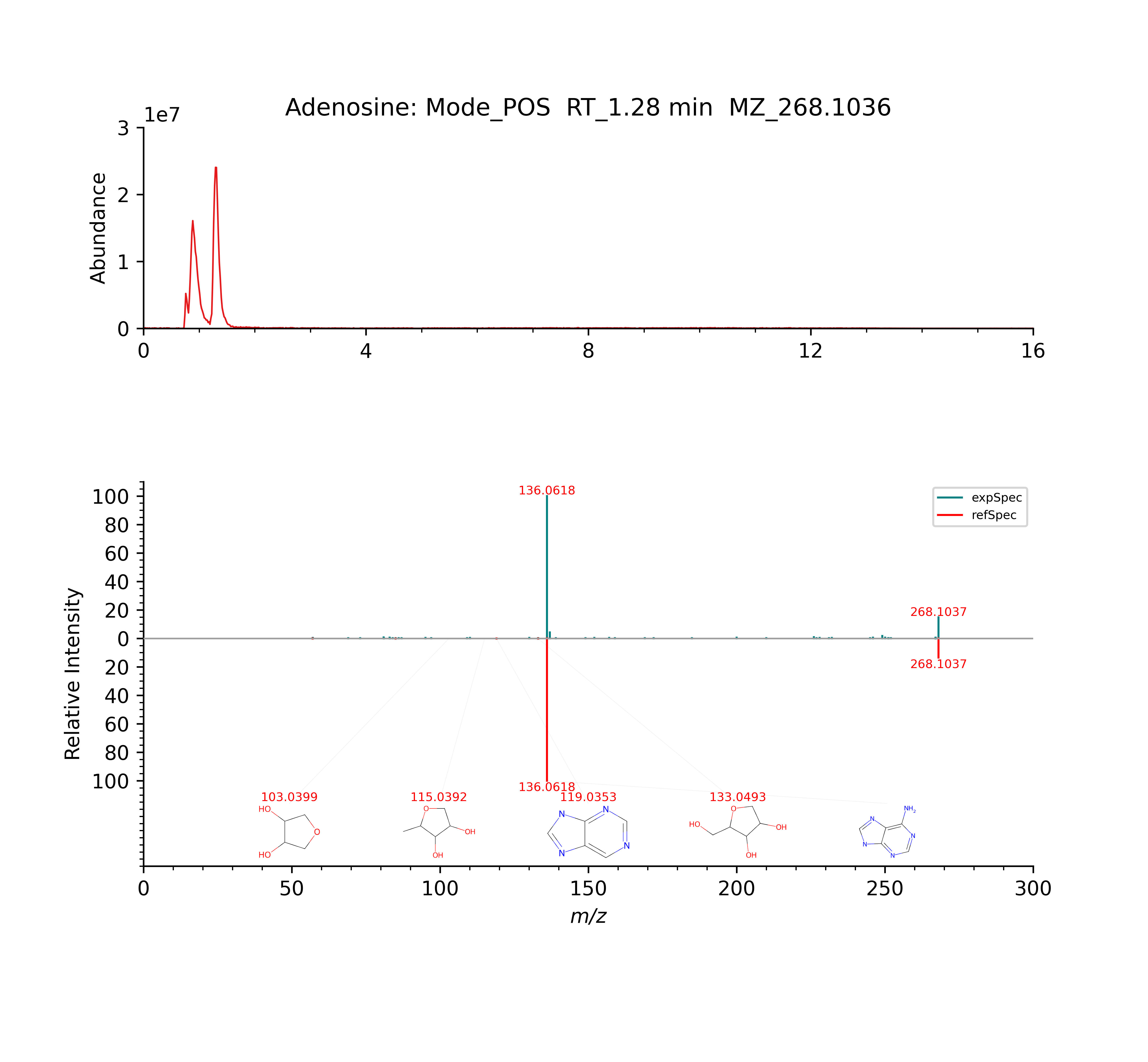

Supplement: Supplementary file 1 [file molecules-29-02840-s001.zip › Supplementary Figure s1/Identification from LuMet-CM datebase/png/compound00097.png]

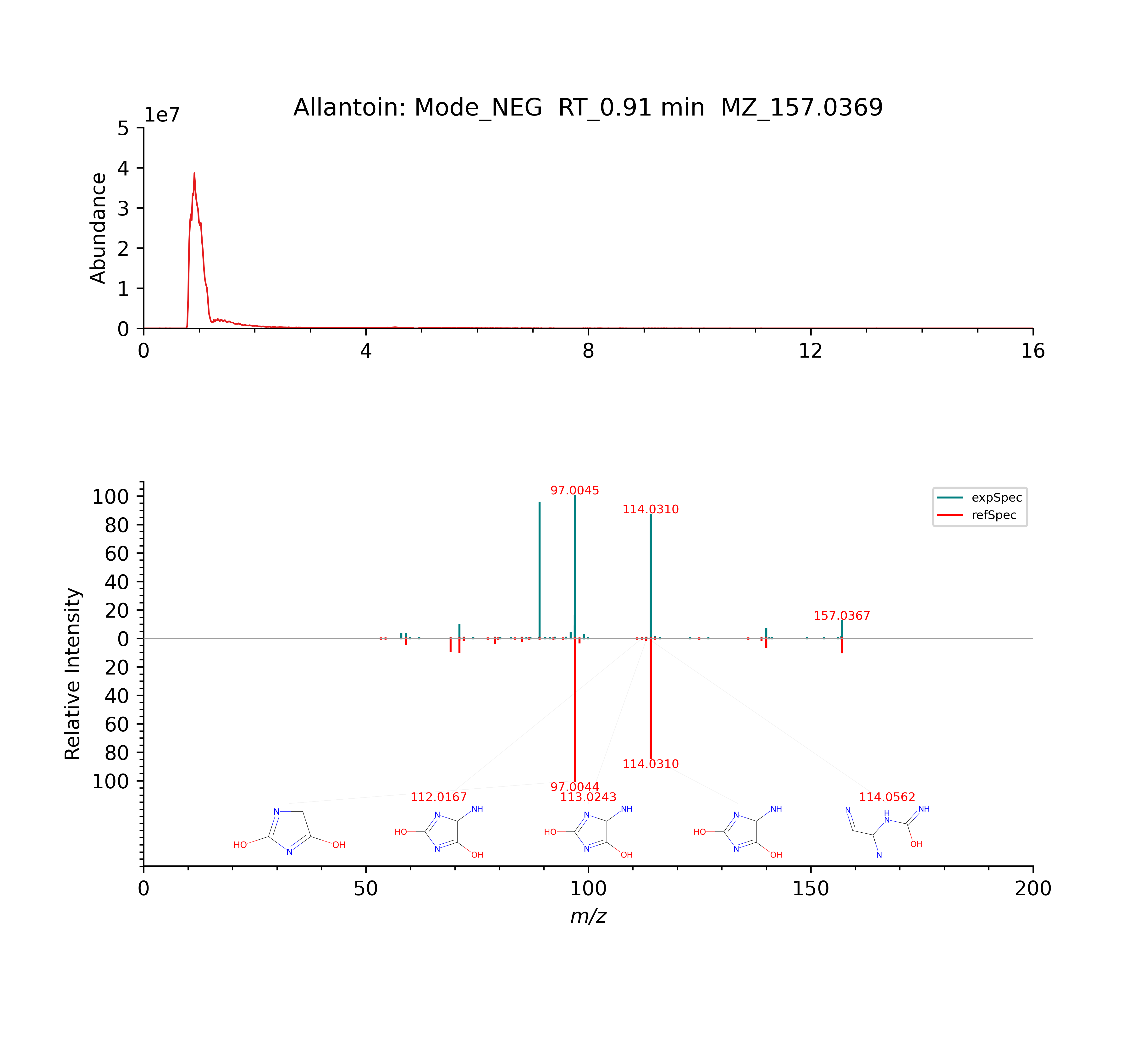

Supplement: Supplementary file 1 [file molecules-29-02840-s001.zip › Supplementary Figure s1/Identification from LuMet-CM datebase/png/compound00098.png]

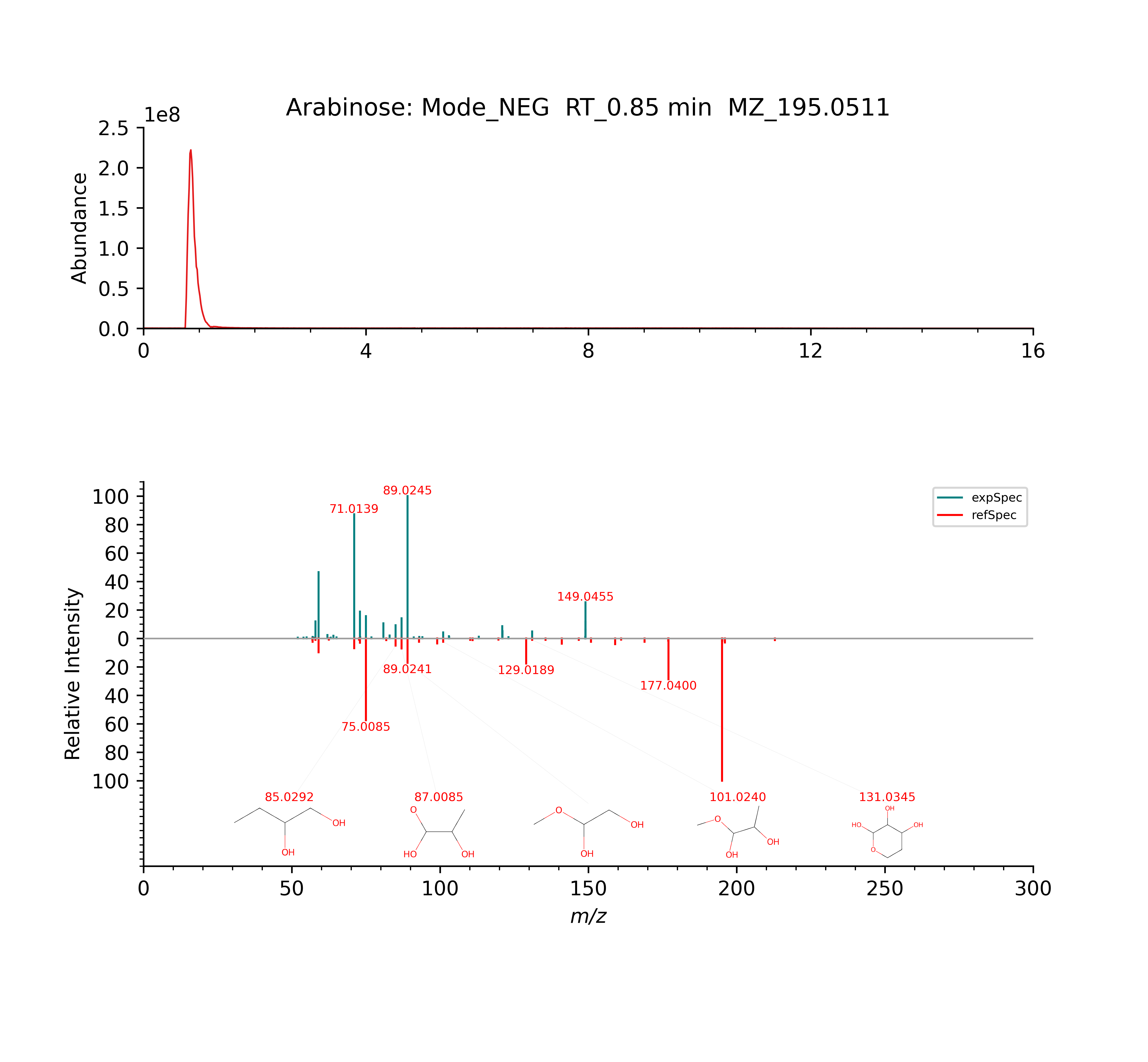

Supplement: Supplementary file 1 [file molecules-29-02840-s001.zip › Supplementary Figure s1/Identification from LuMet-CM datebase/png/compound00099.png]

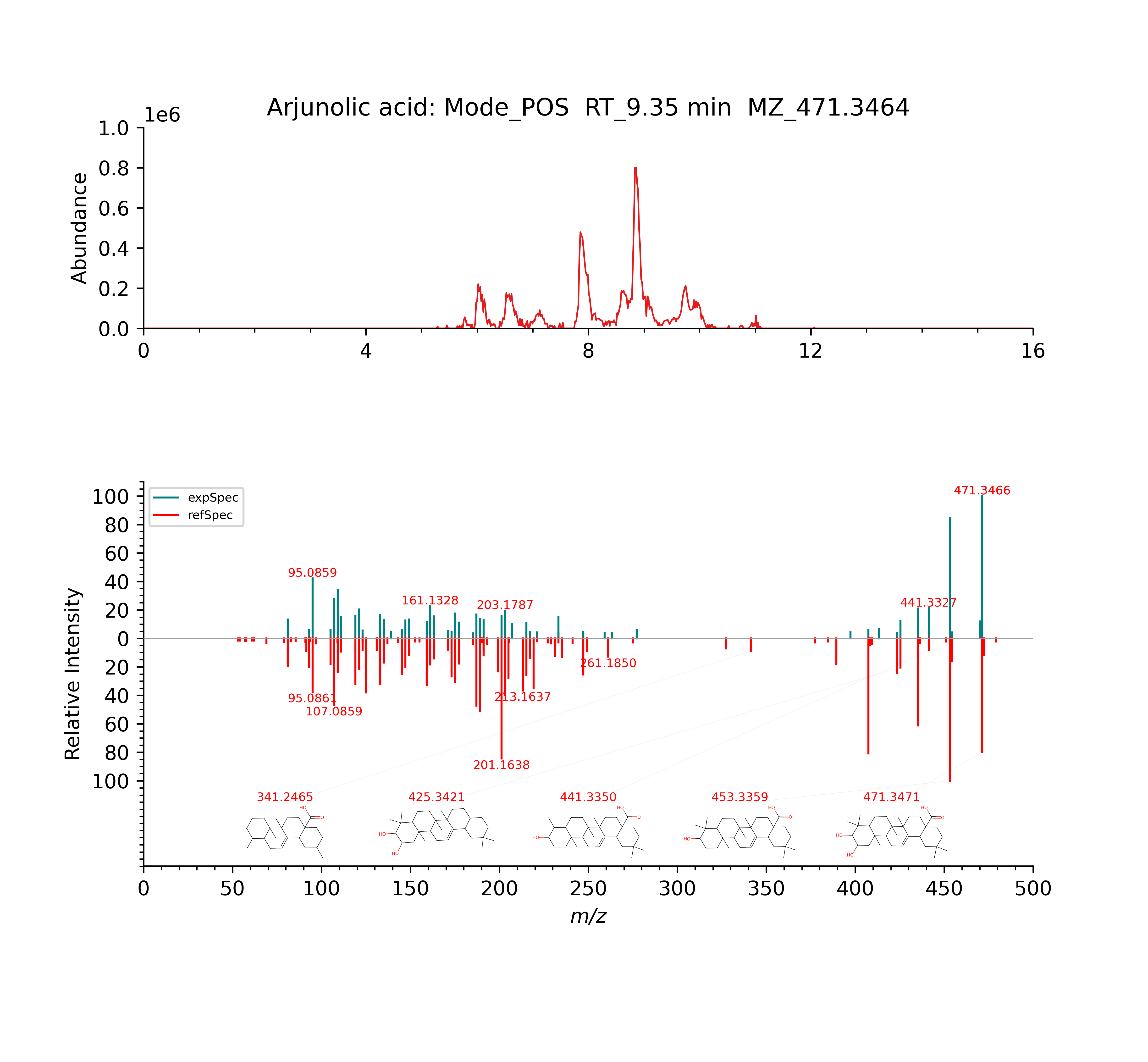

Supplement: Supplementary file 1 [file molecules-29-02840-s001.zip › Supplementary Figure s1/Identification from LuMet-CM datebase/png/compound00100.png]

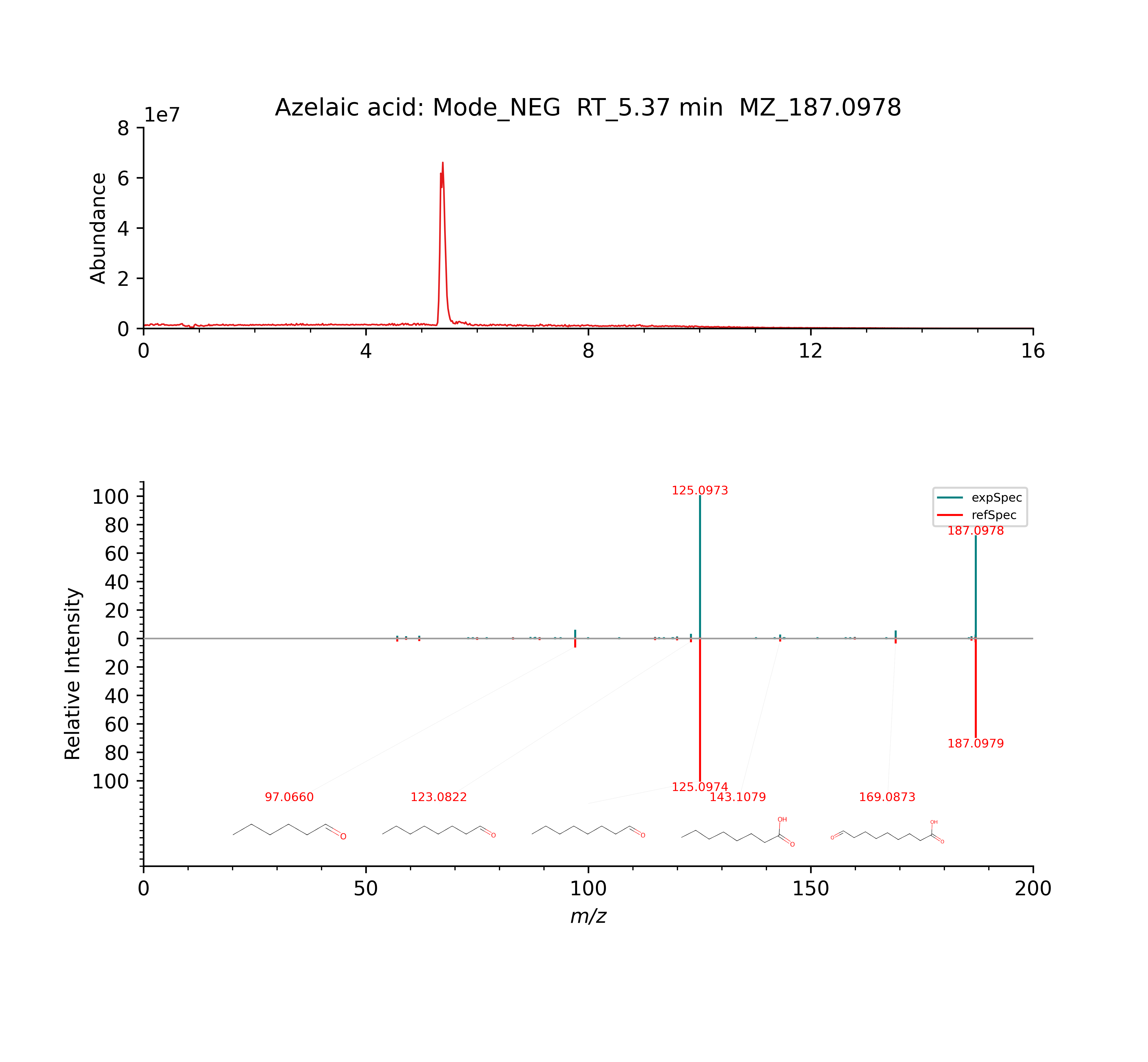

Supplement: Supplementary file 1 [file molecules-29-02840-s001.zip › Supplementary Figure s1/Identification from LuMet-CM datebase/png/compound00101.png]

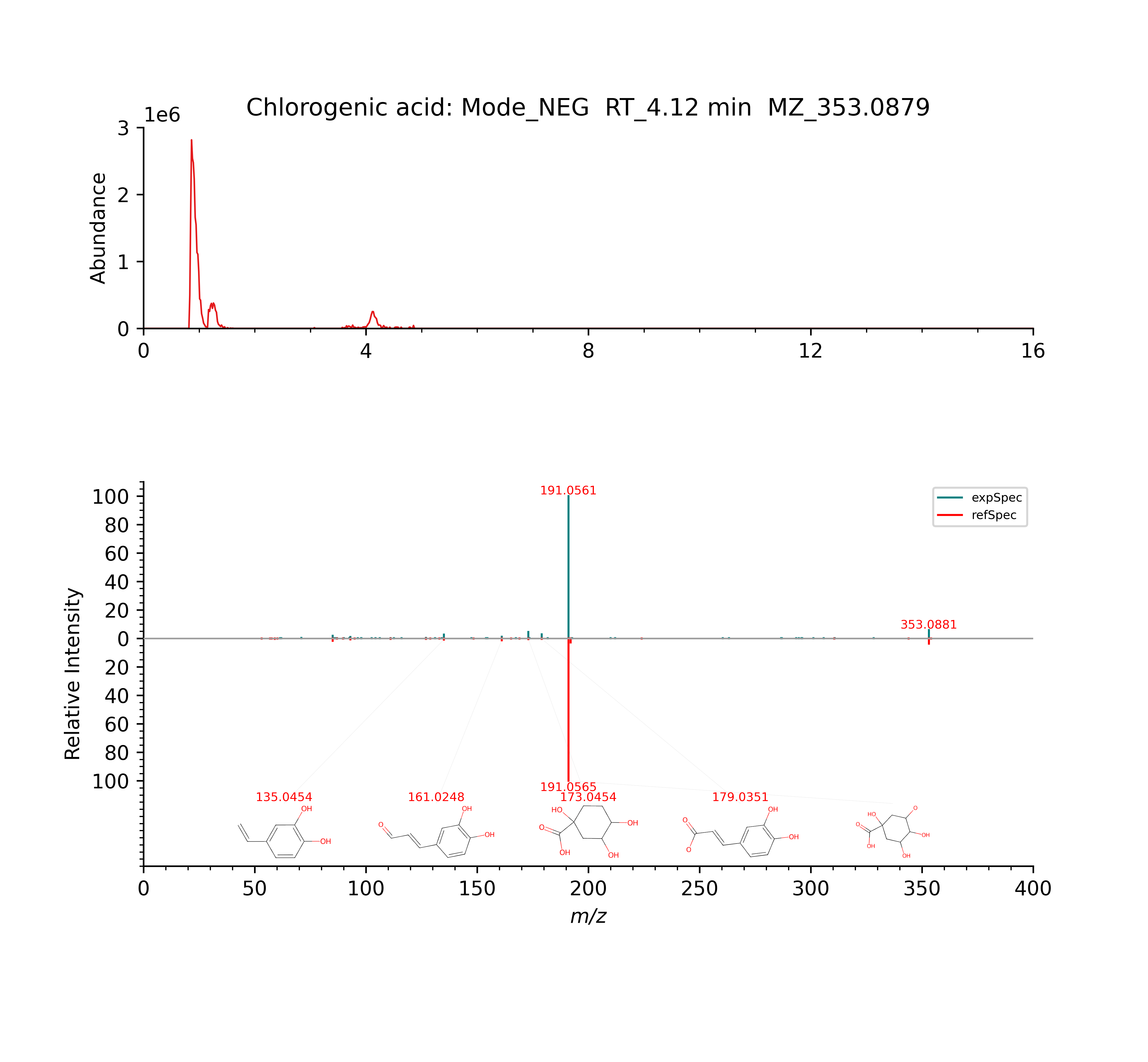

Supplement: Supplementary file 1 [file molecules-29-02840-s001.zip › Supplementary Figure s1/Identification from LuMet-CM datebase/png/compound00102.png]

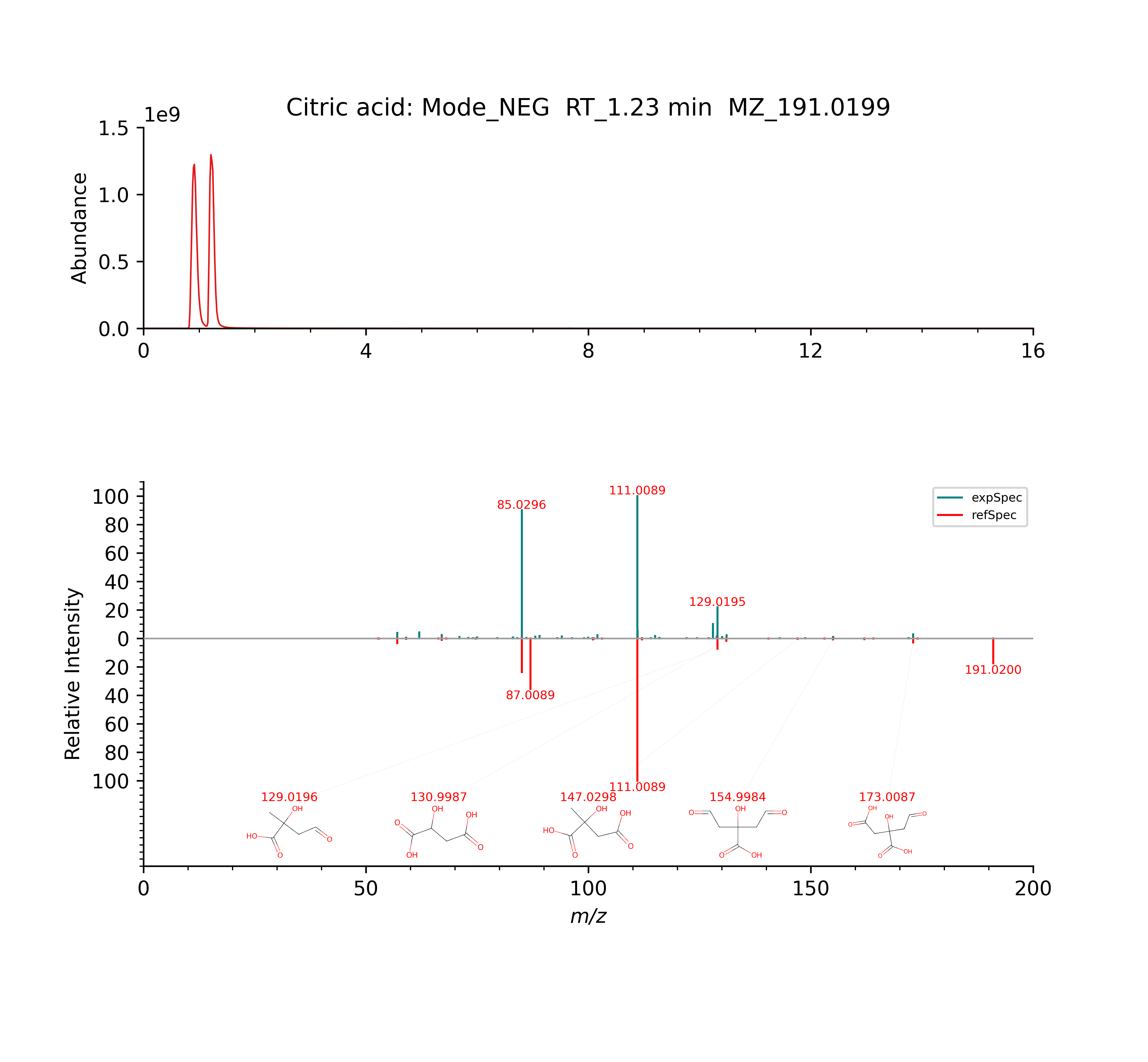

Supplement: Supplementary file 1 [file molecules-29-02840-s001.zip › Supplementary Figure s1/Identification from LuMet-CM datebase/png/compound00103.png]

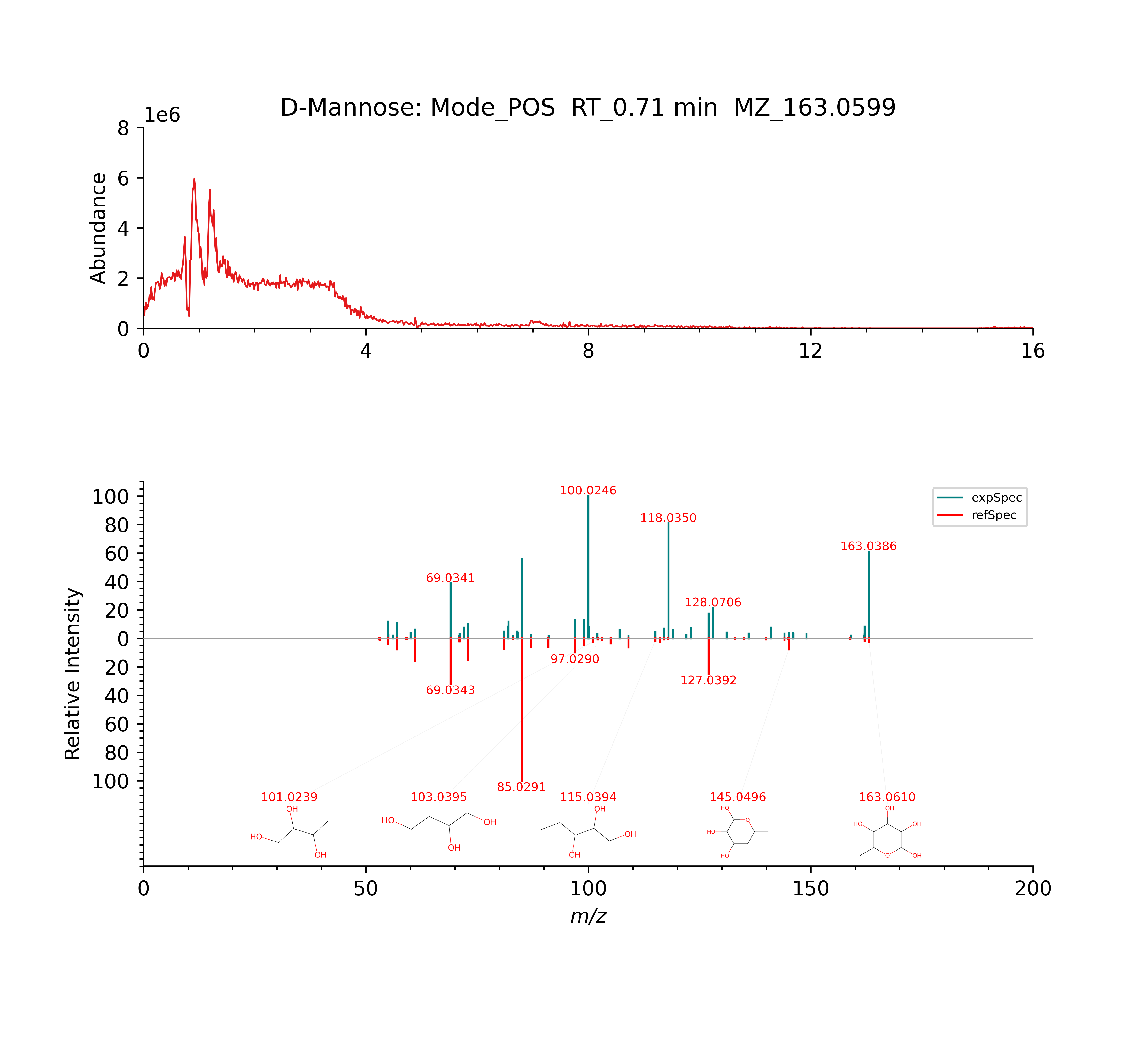

Supplement: Supplementary file 1 [file molecules-29-02840-s001.zip › Supplementary Figure s1/Identification from LuMet-CM datebase/png/compound00104.png]

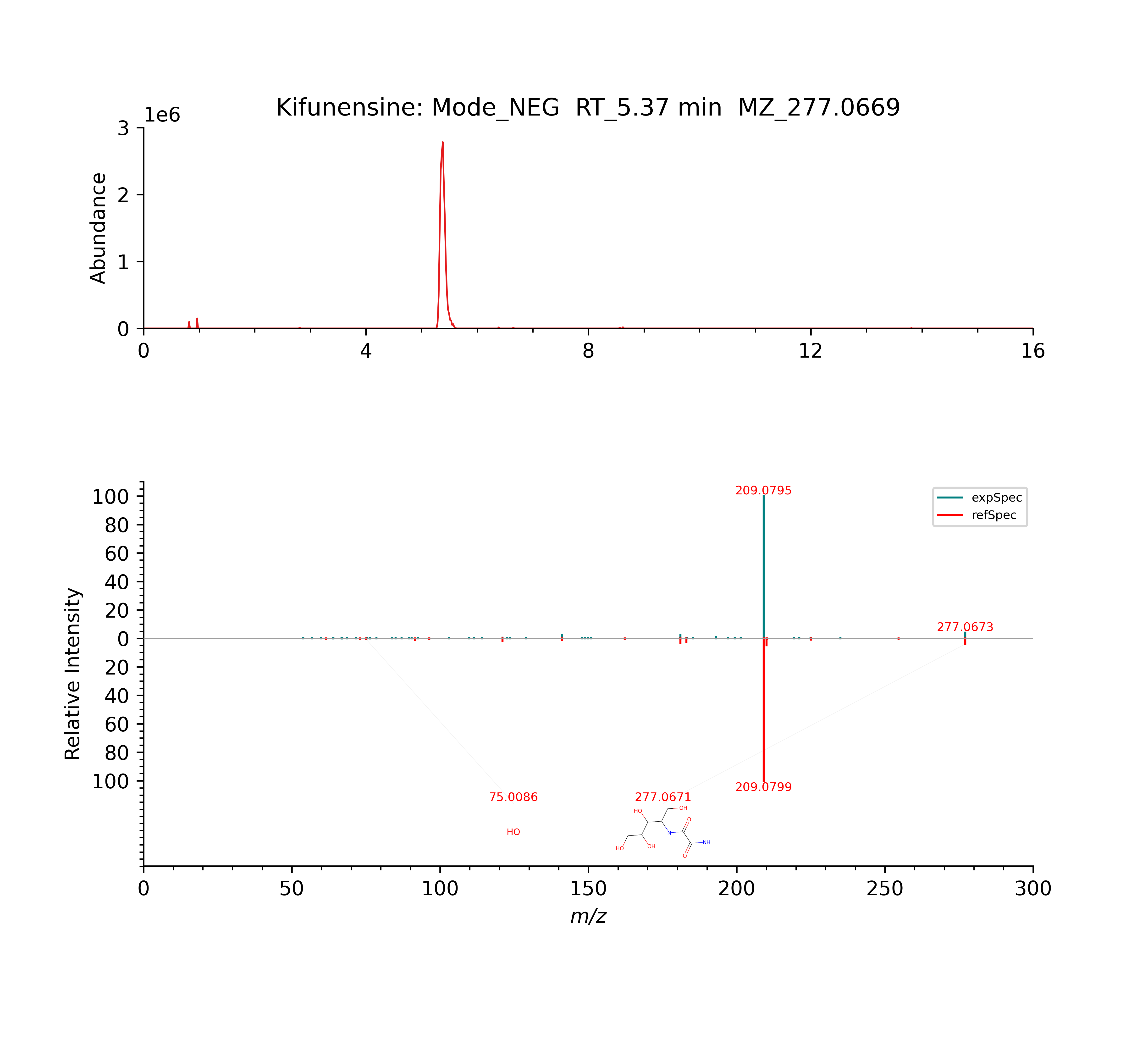

Supplement: Supplementary file 1 [file molecules-29-02840-s001.zip › Supplementary Figure s1/Identification from LuMet-CM datebase/png/compound00105.png]

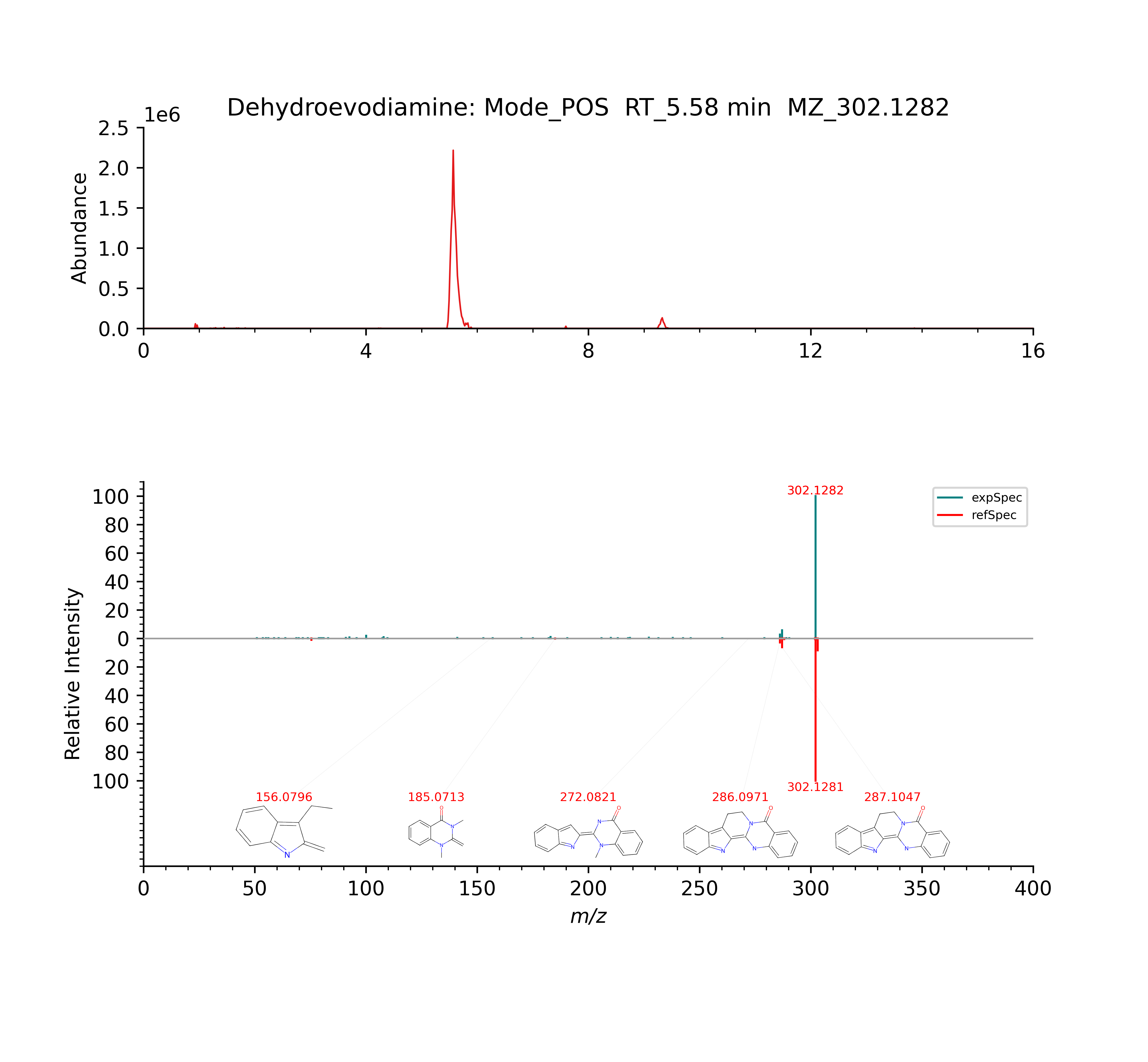

Supplement: Supplementary file 1 [file molecules-29-02840-s001.zip › Supplementary Figure s1/Identification from LuMet-CM datebase/png/compound00106.png]

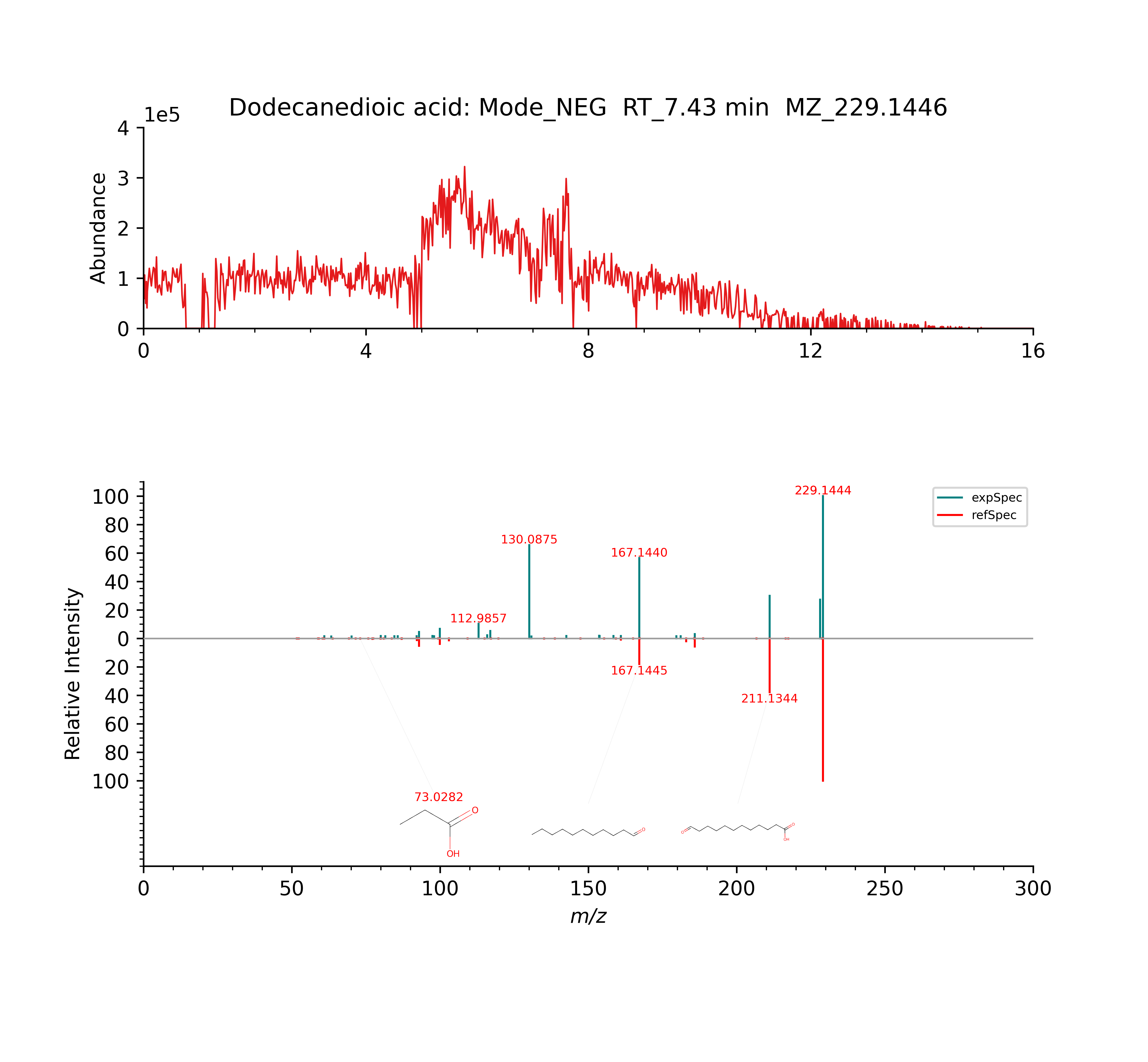

Supplement: Supplementary file 1 [file molecules-29-02840-s001.zip › Supplementary Figure s1/Identification from LuMet-CM datebase/png/compound00107.png]

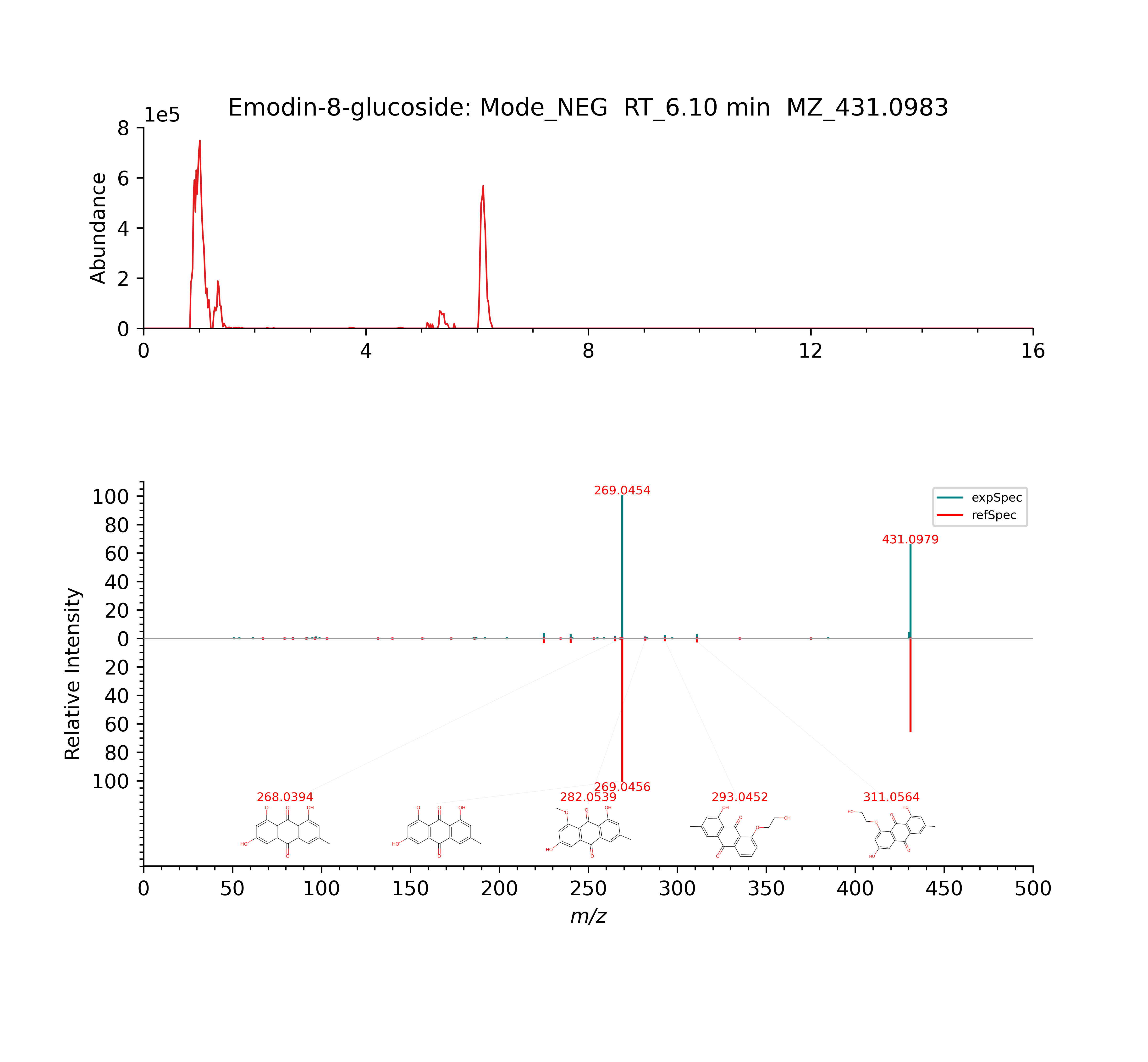

Supplement: Supplementary file 1 [file molecules-29-02840-s001.zip › Supplementary Figure s1/Identification from LuMet-CM datebase/png/compound00108.png]

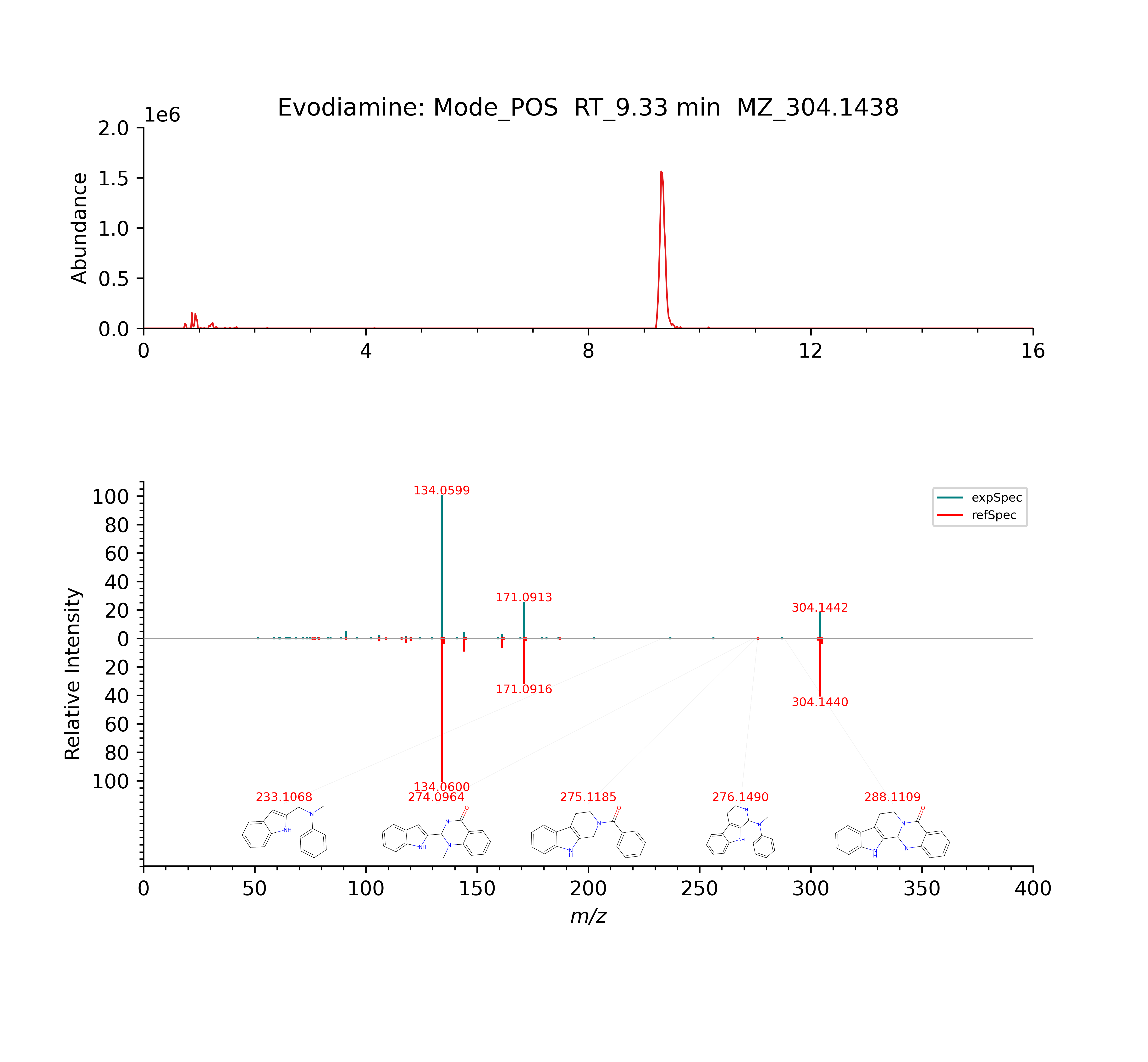

Supplement: Supplementary file 1 [file molecules-29-02840-s001.zip › Supplementary Figure s1/Identification from LuMet-CM datebase/png/compound00109.png]

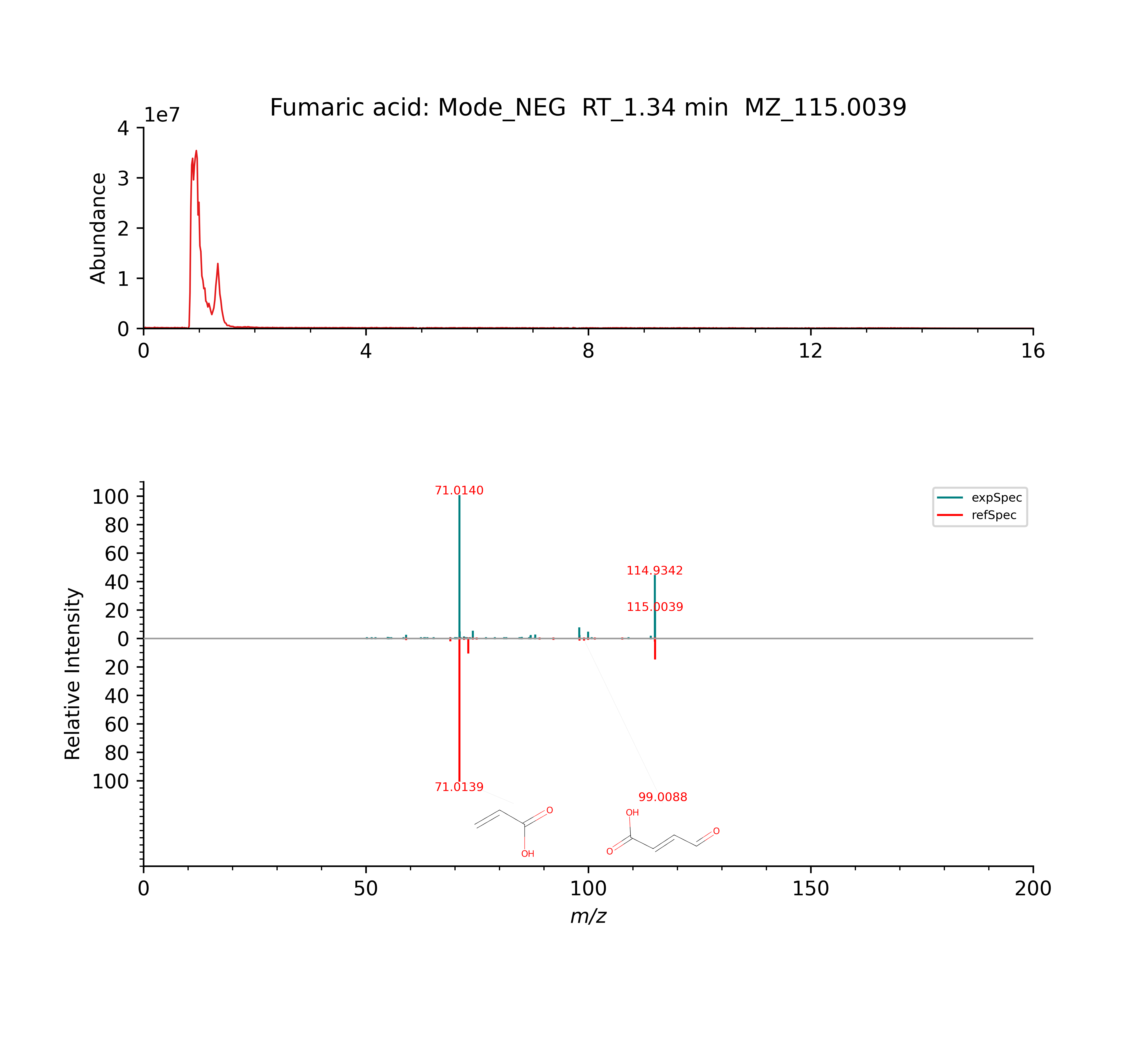

Supplement: Supplementary file 1 [file molecules-29-02840-s001.zip › Supplementary Figure s1/Identification from LuMet-CM datebase/png/compound00110.png]

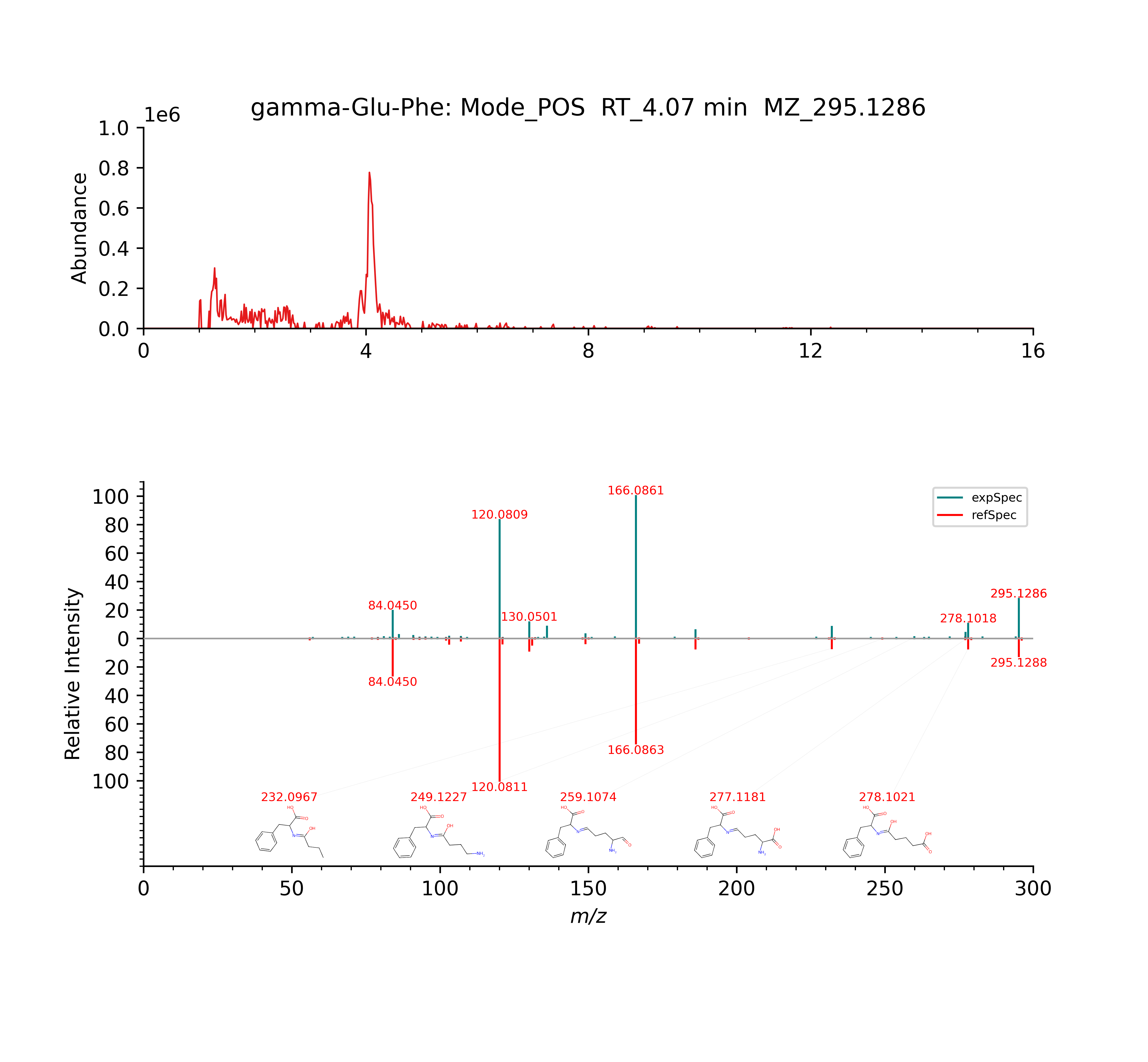

Supplement: Supplementary file 1 [file molecules-29-02840-s001.zip › Supplementary Figure s1/Identification from LuMet-CM datebase/png/compound00111.png]

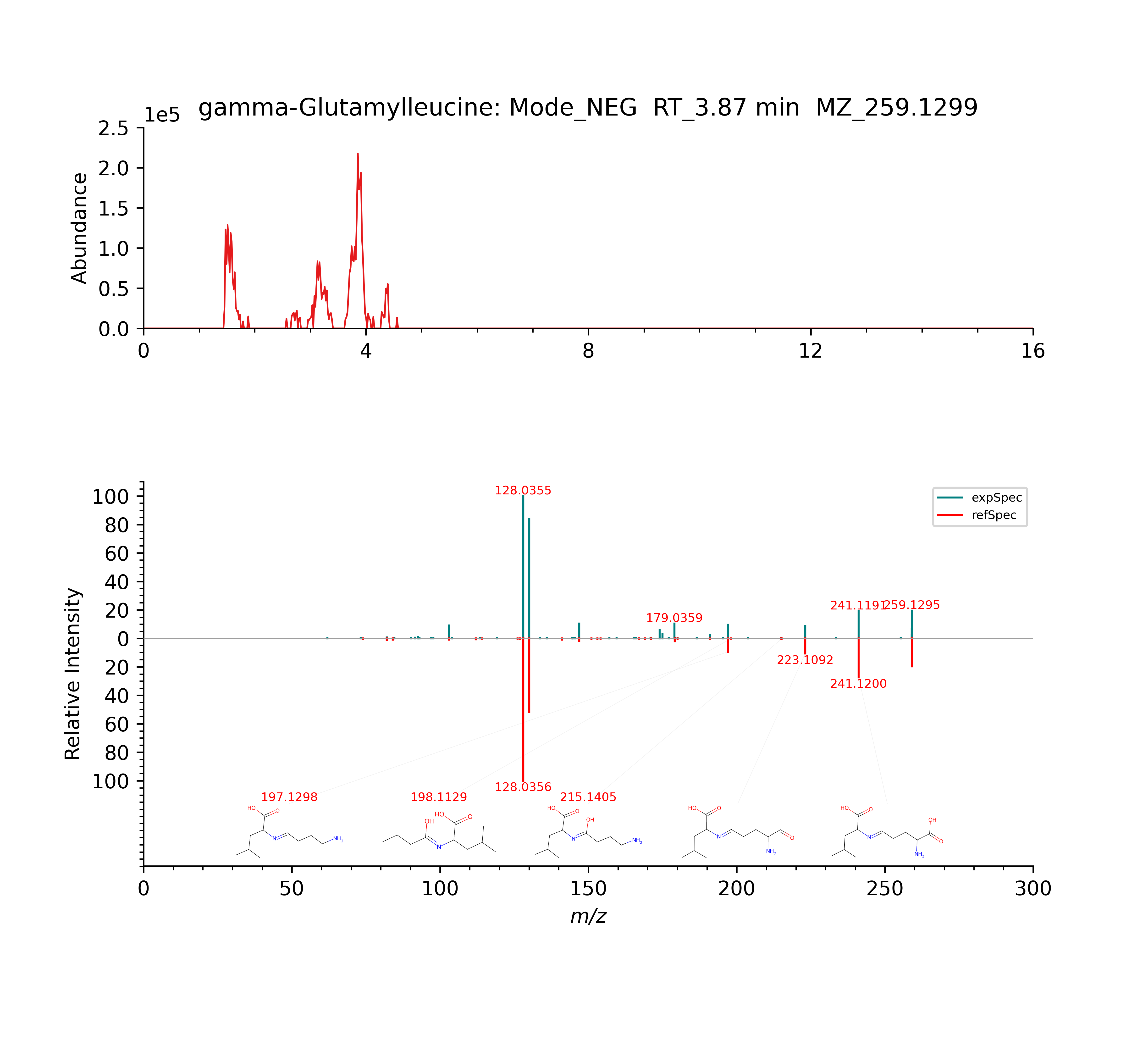

Supplement: Supplementary file 1 [file molecules-29-02840-s001.zip › Supplementary Figure s1/Identification from LuMet-CM datebase/png/compound00112.png]

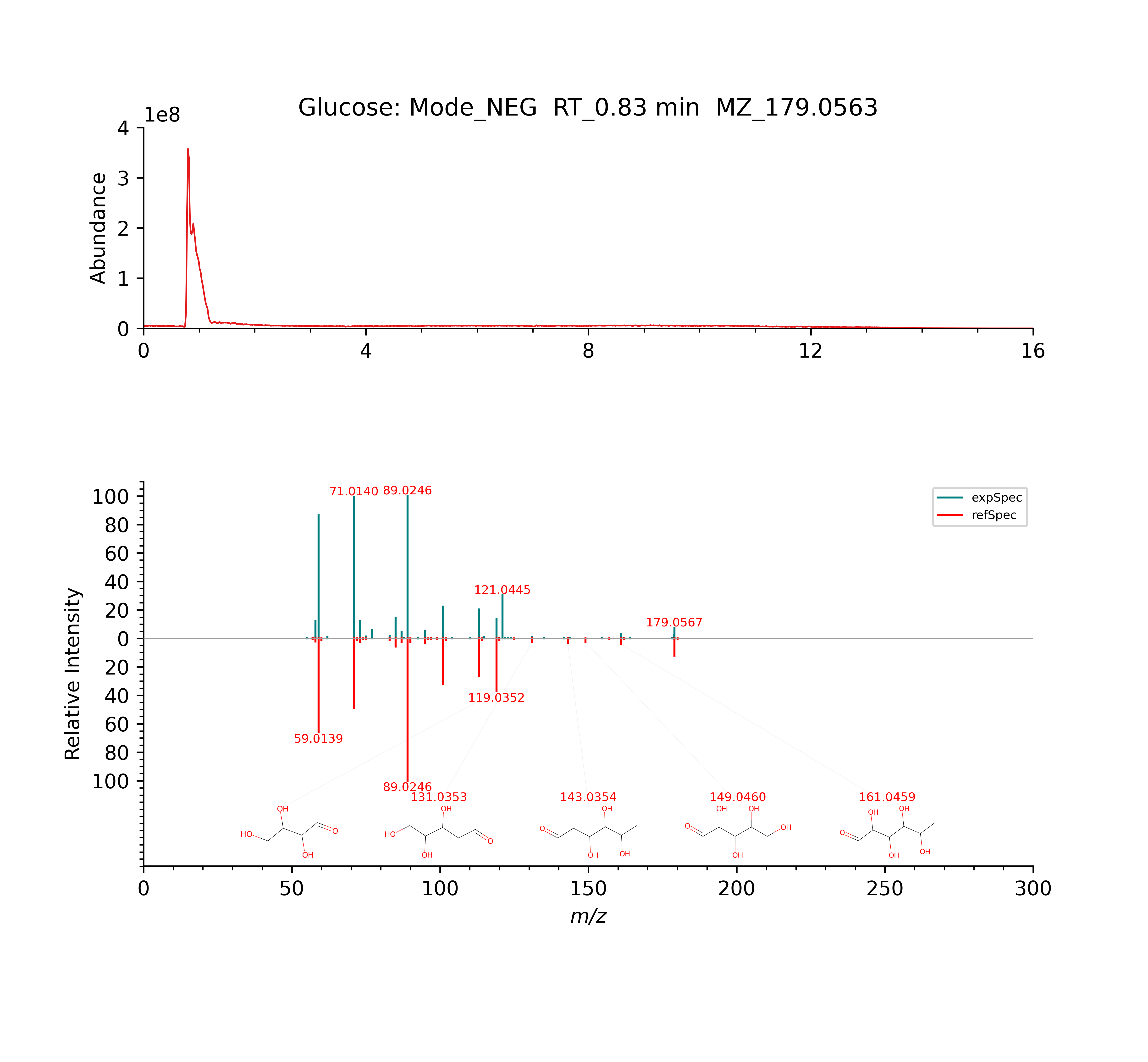

Supplement: Supplementary file 1 [file molecules-29-02840-s001.zip › Supplementary Figure s1/Identification from LuMet-CM datebase/png/compound00113.png]

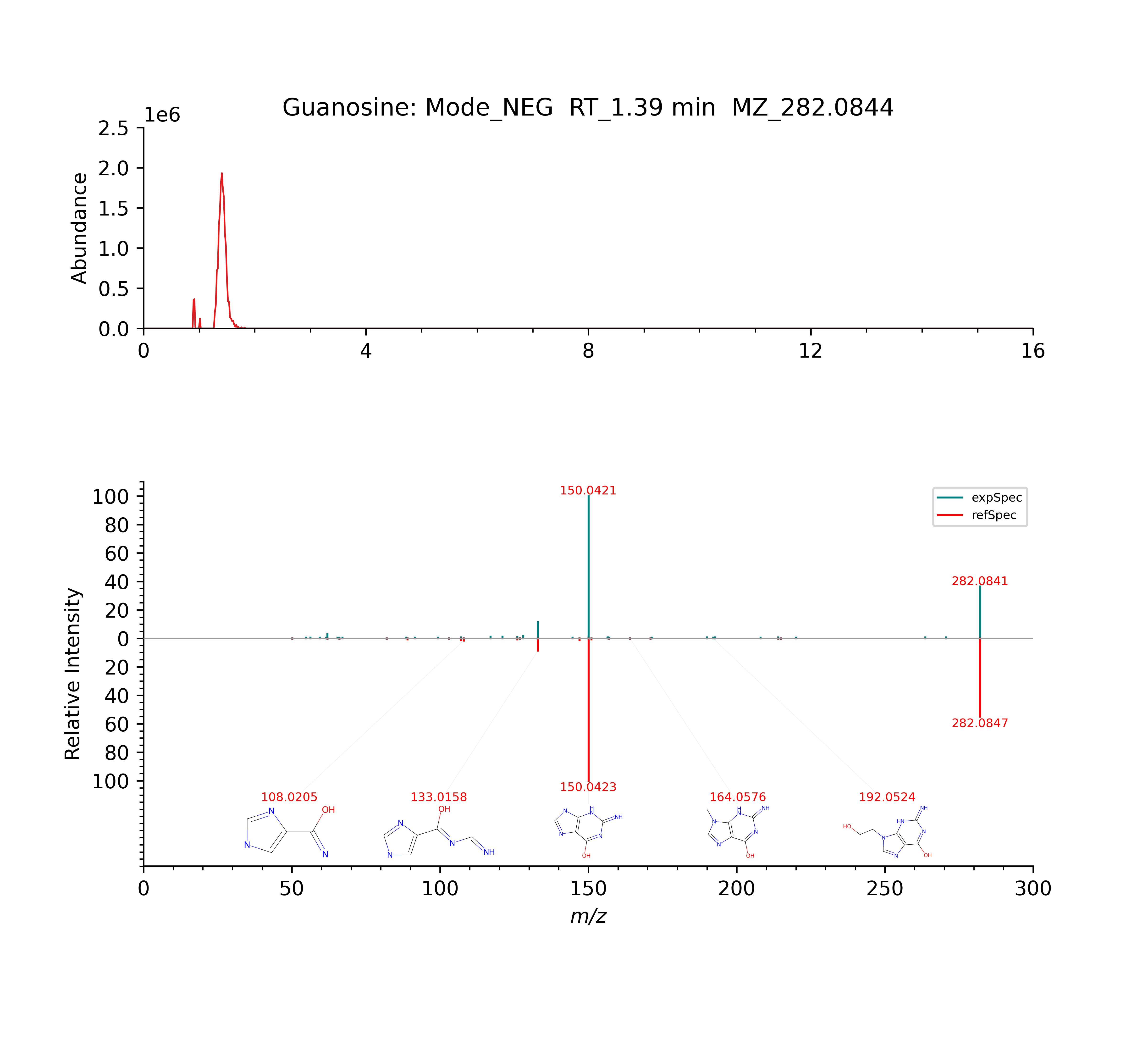

Supplement: Supplementary file 1 [file molecules-29-02840-s001.zip › Supplementary Figure s1/Identification from LuMet-CM datebase/png/compound00114.png]

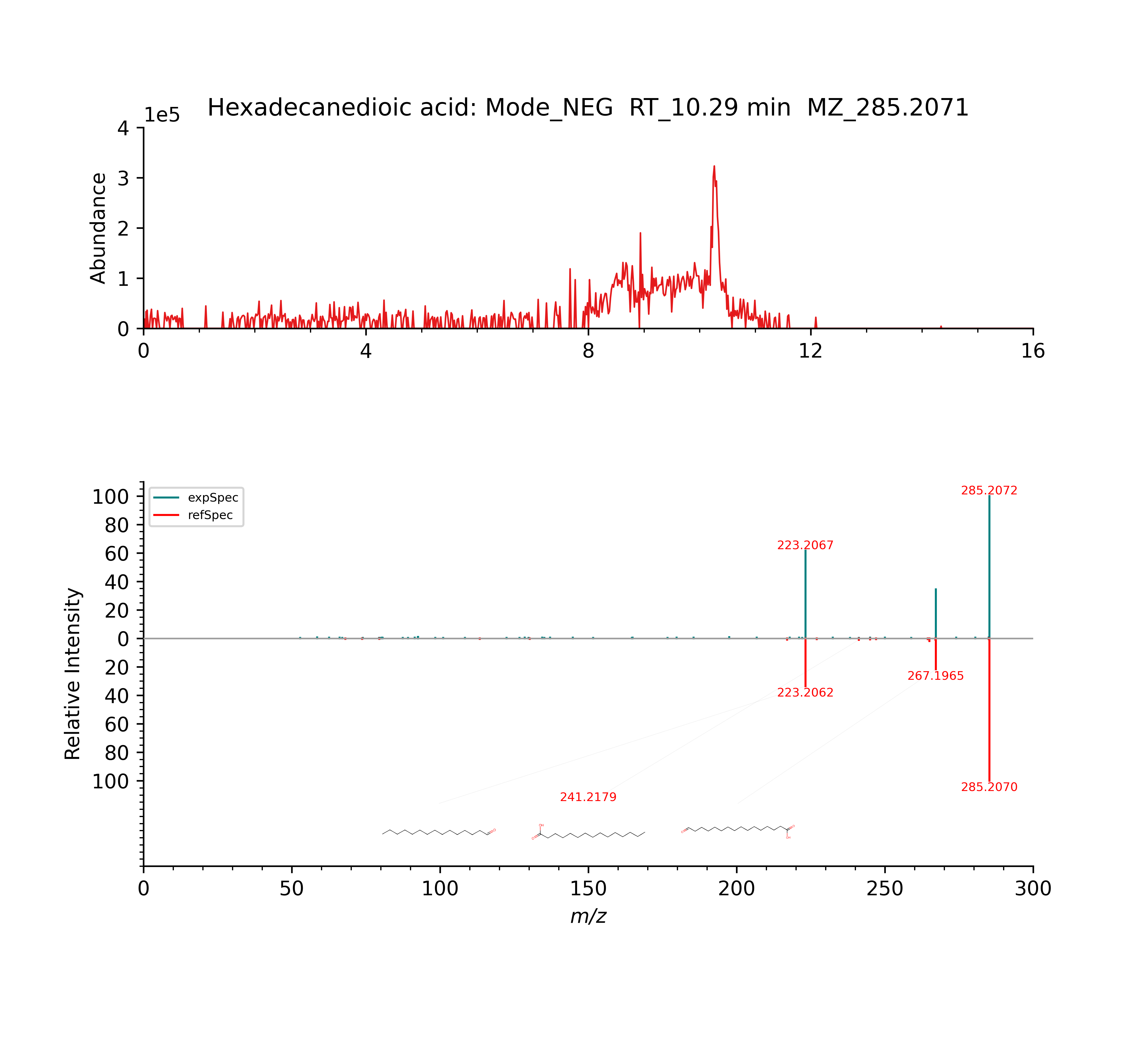

Supplement: Supplementary file 1 [file molecules-29-02840-s001.zip › Supplementary Figure s1/Identification from LuMet-CM datebase/png/compound00115.png]

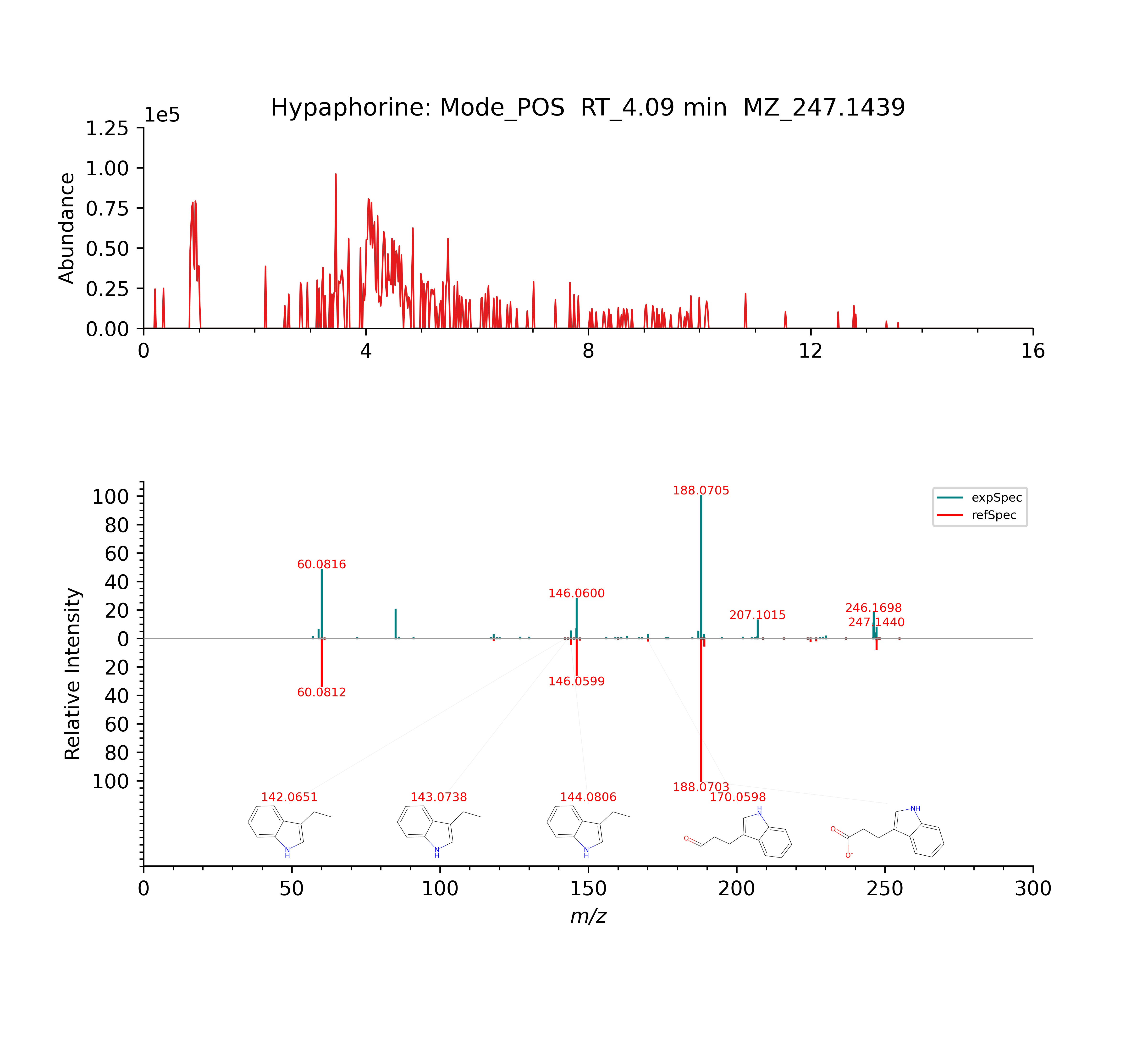

Supplement: Supplementary file 1 [file molecules-29-02840-s001.zip › Supplementary Figure s1/Identification from LuMet-CM datebase/png/compound00116.png]

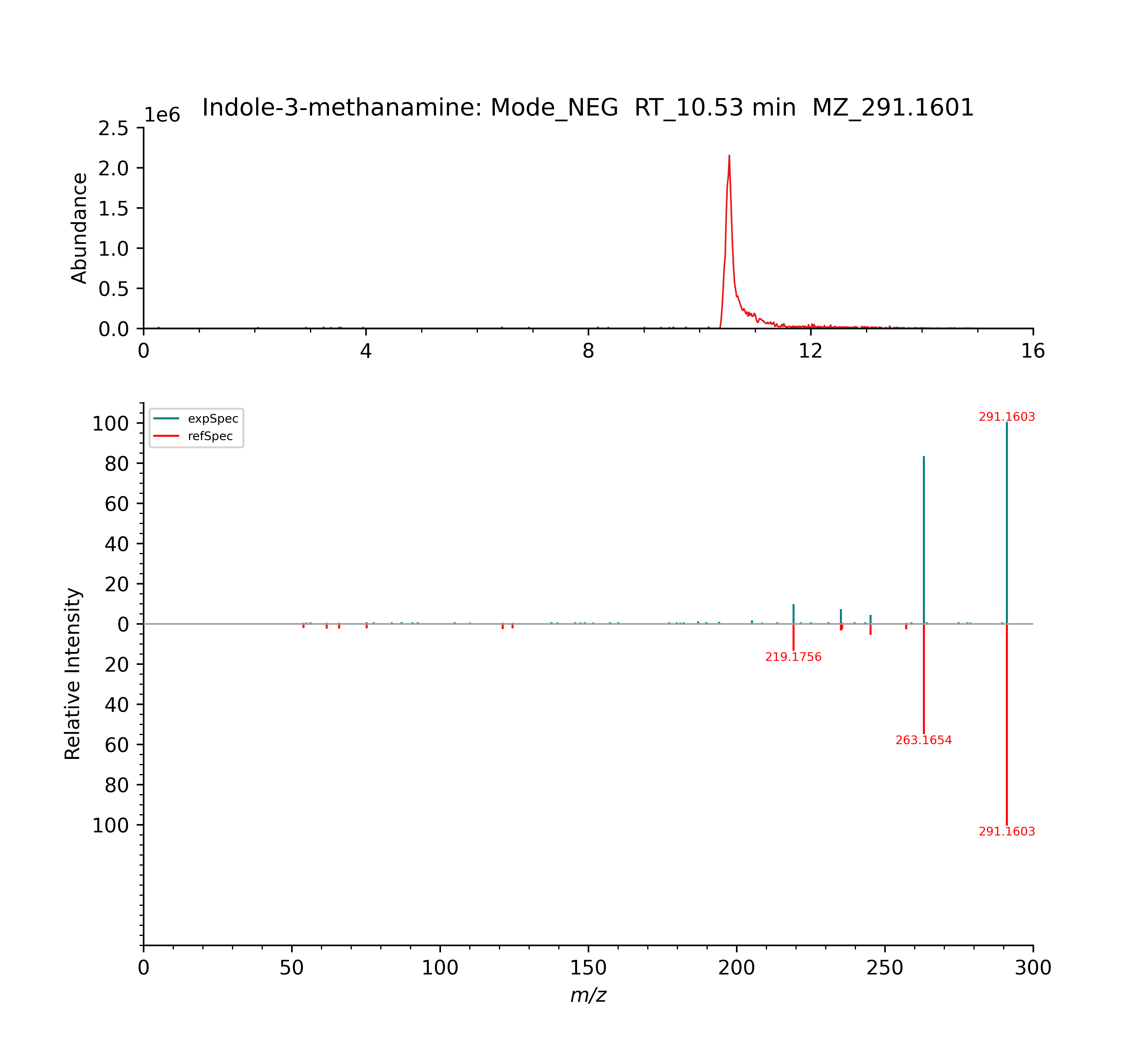

Supplement: Supplementary file 1 [file molecules-29-02840-s001.zip › Supplementary Figure s1/Identification from LuMet-CM datebase/png/compound00117.png]

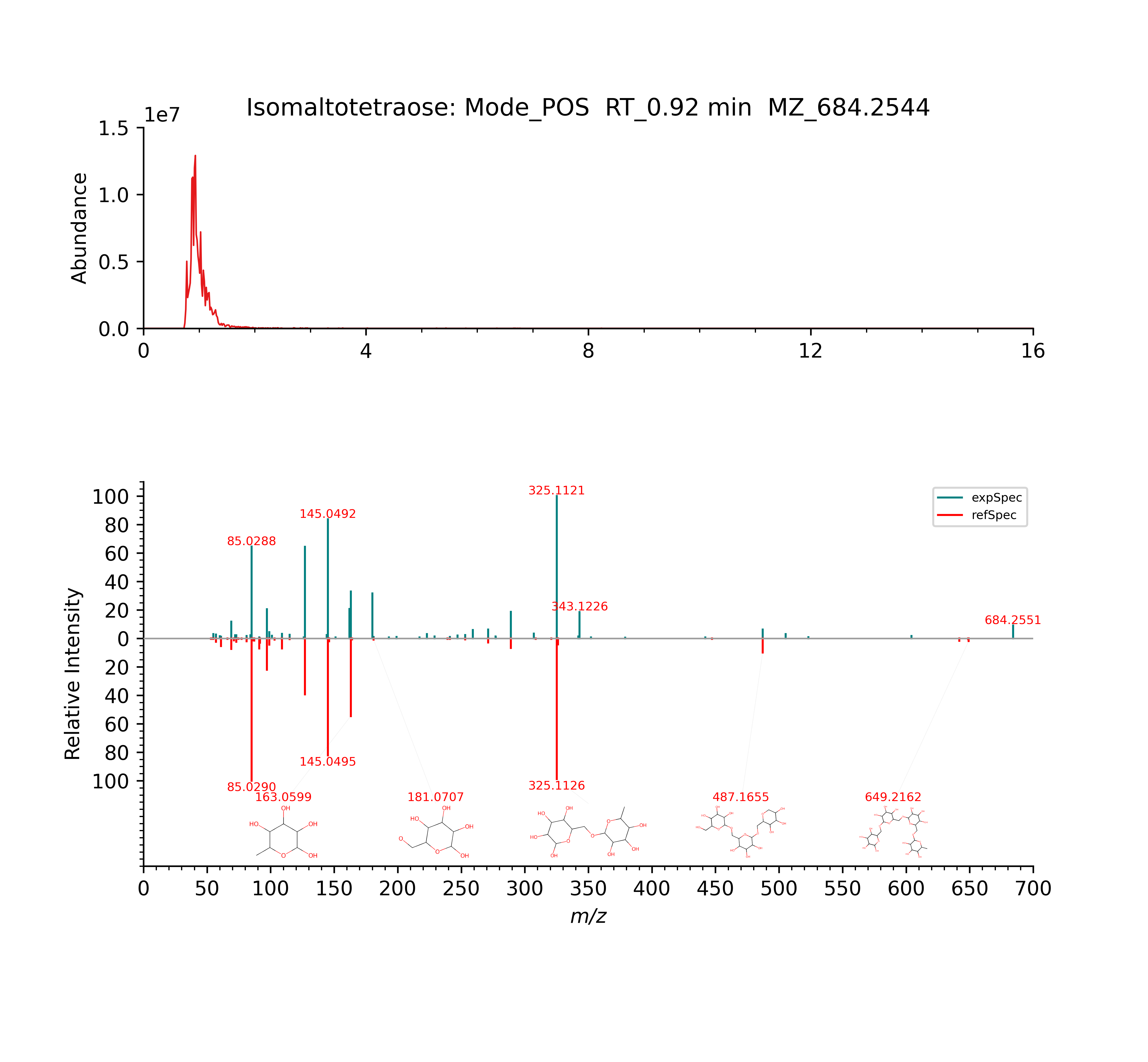

Supplement: Supplementary file 1 [file molecules-29-02840-s001.zip › Supplementary Figure s1/Identification from LuMet-CM datebase/png/compound00118.png]

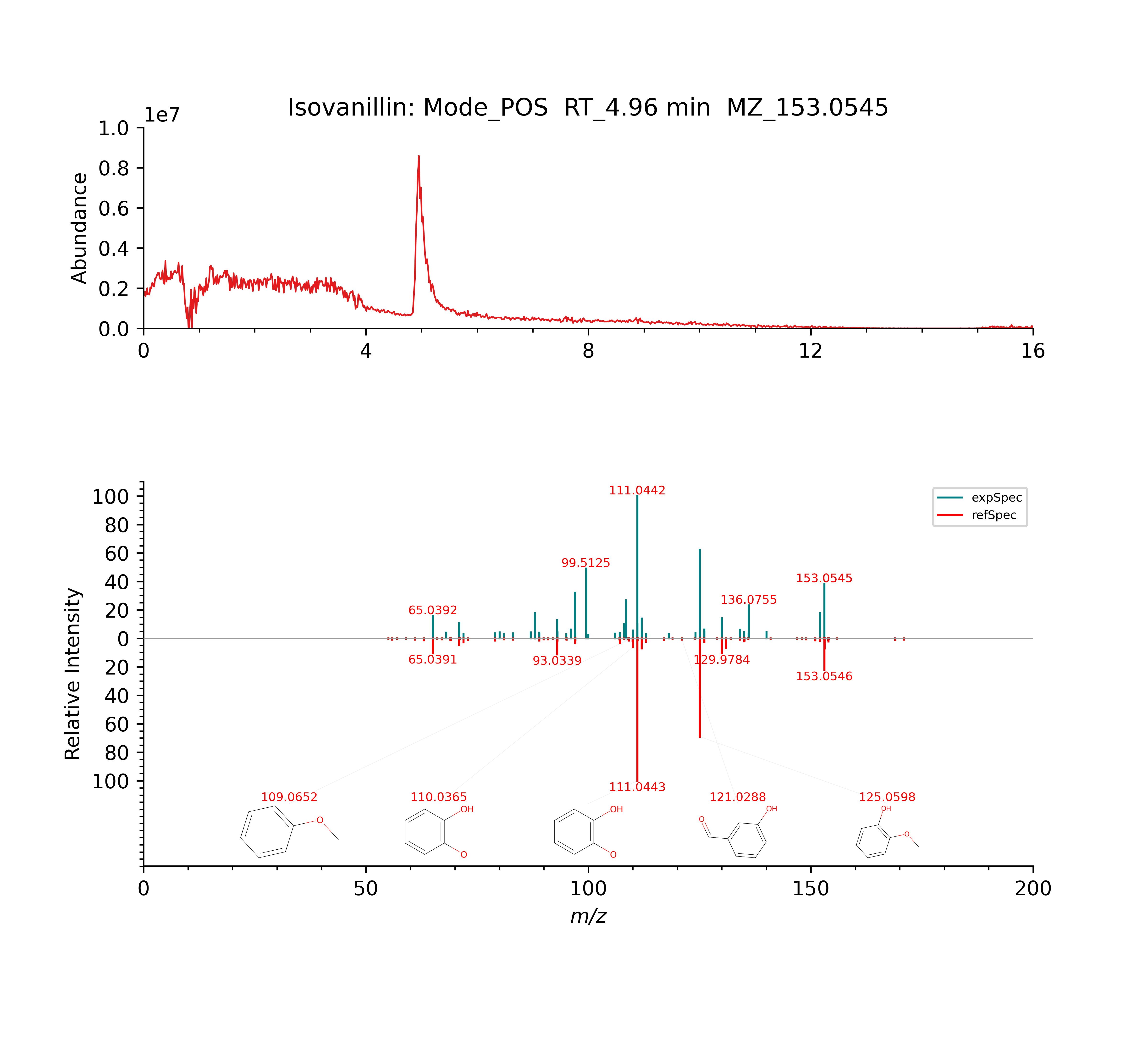

Supplement: Supplementary file 1 [file molecules-29-02840-s001.zip › Supplementary Figure s1/Identification from LuMet-CM datebase/png/compound00119.png]

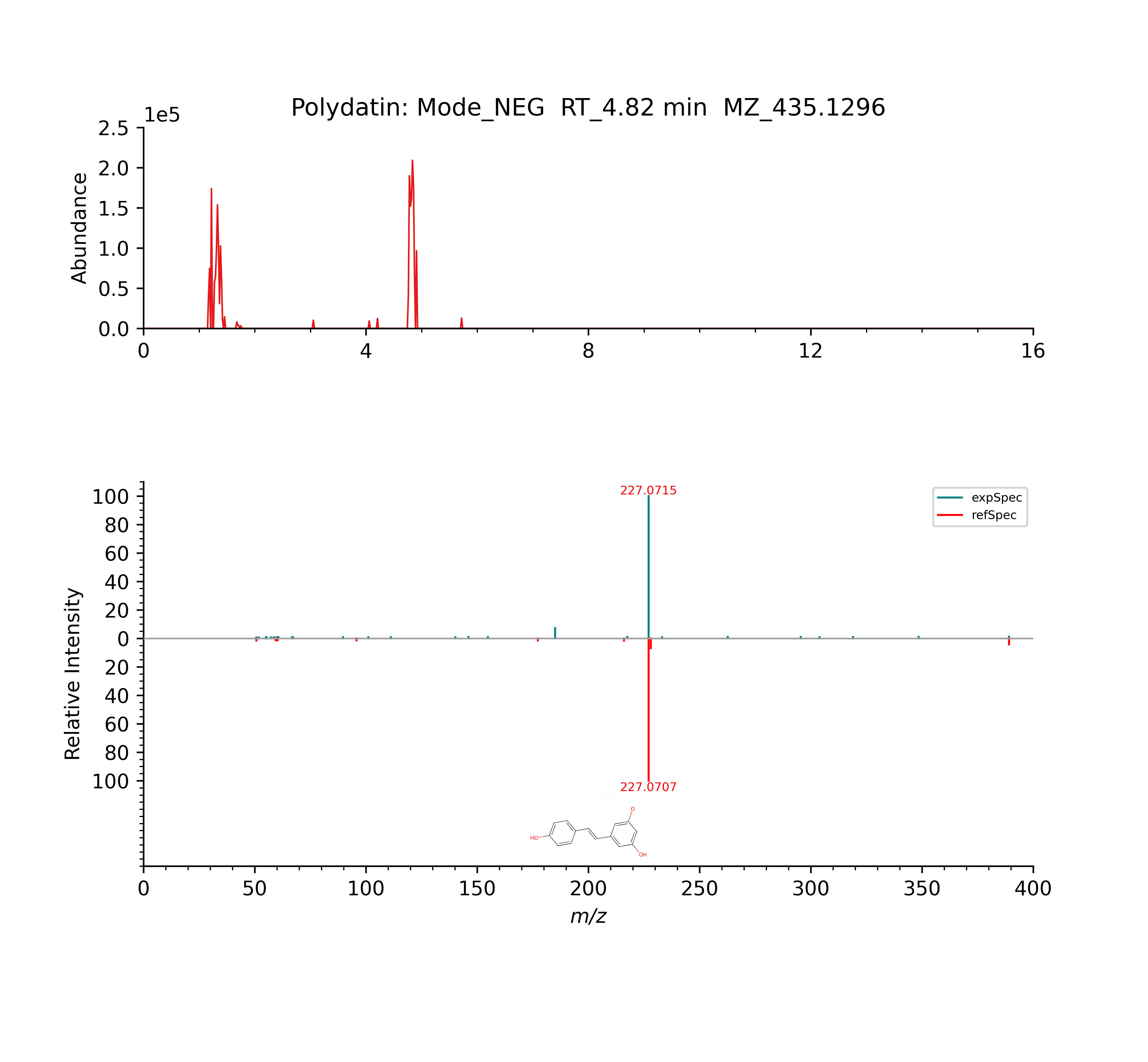

Supplement: Supplementary file 1 [file molecules-29-02840-s001.zip › Supplementary Figure s1/Identification from LuMet-CM datebase/png/compound00120.png]

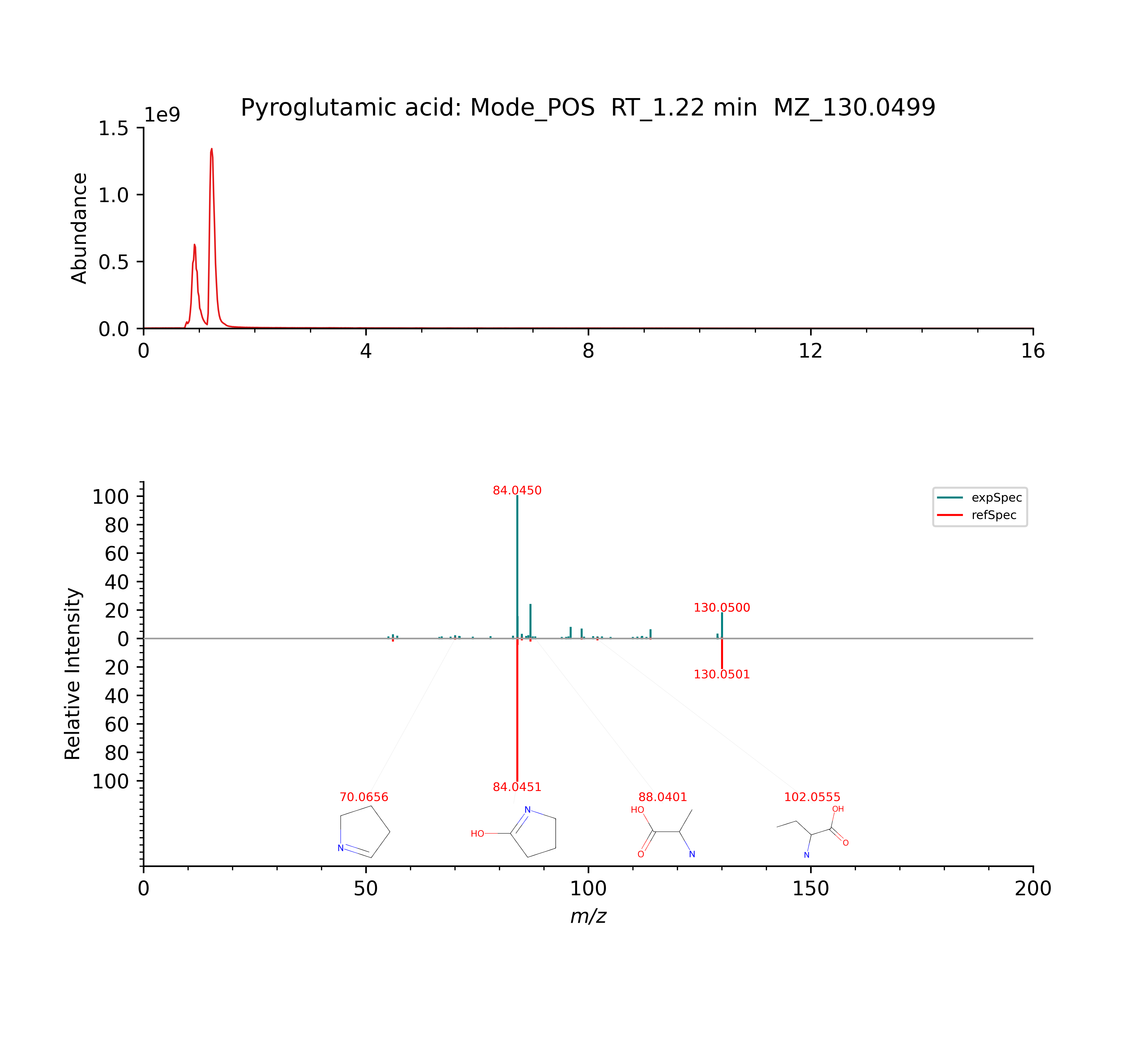

Supplement: Supplementary file 1 [file molecules-29-02840-s001.zip › Supplementary Figure s1/Identification from LuMet-CM datebase/png/compound00121.png]

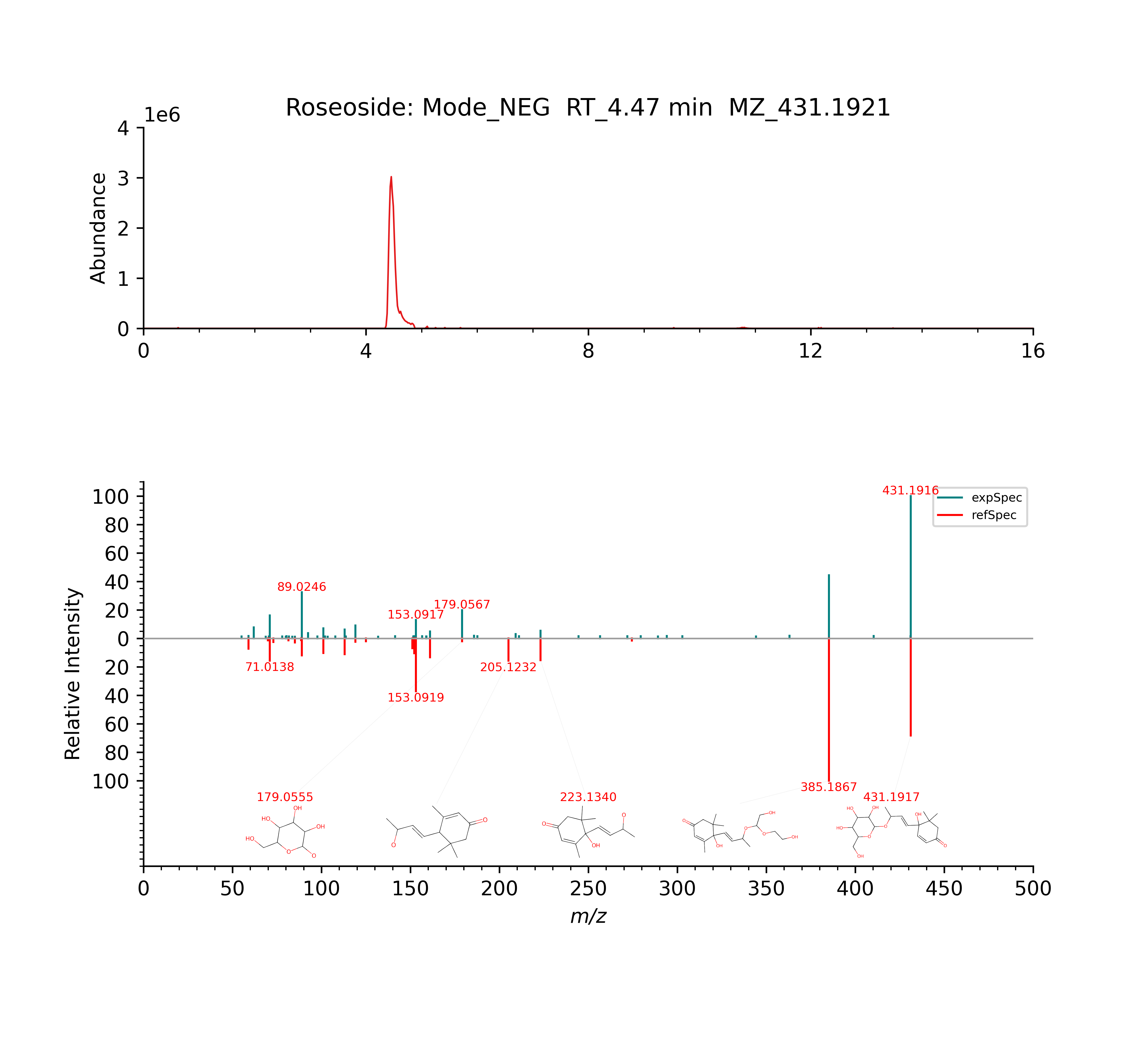

Supplement: Supplementary file 1 [file molecules-29-02840-s001.zip › Supplementary Figure s1/Identification from LuMet-CM datebase/png/compound00122.png]

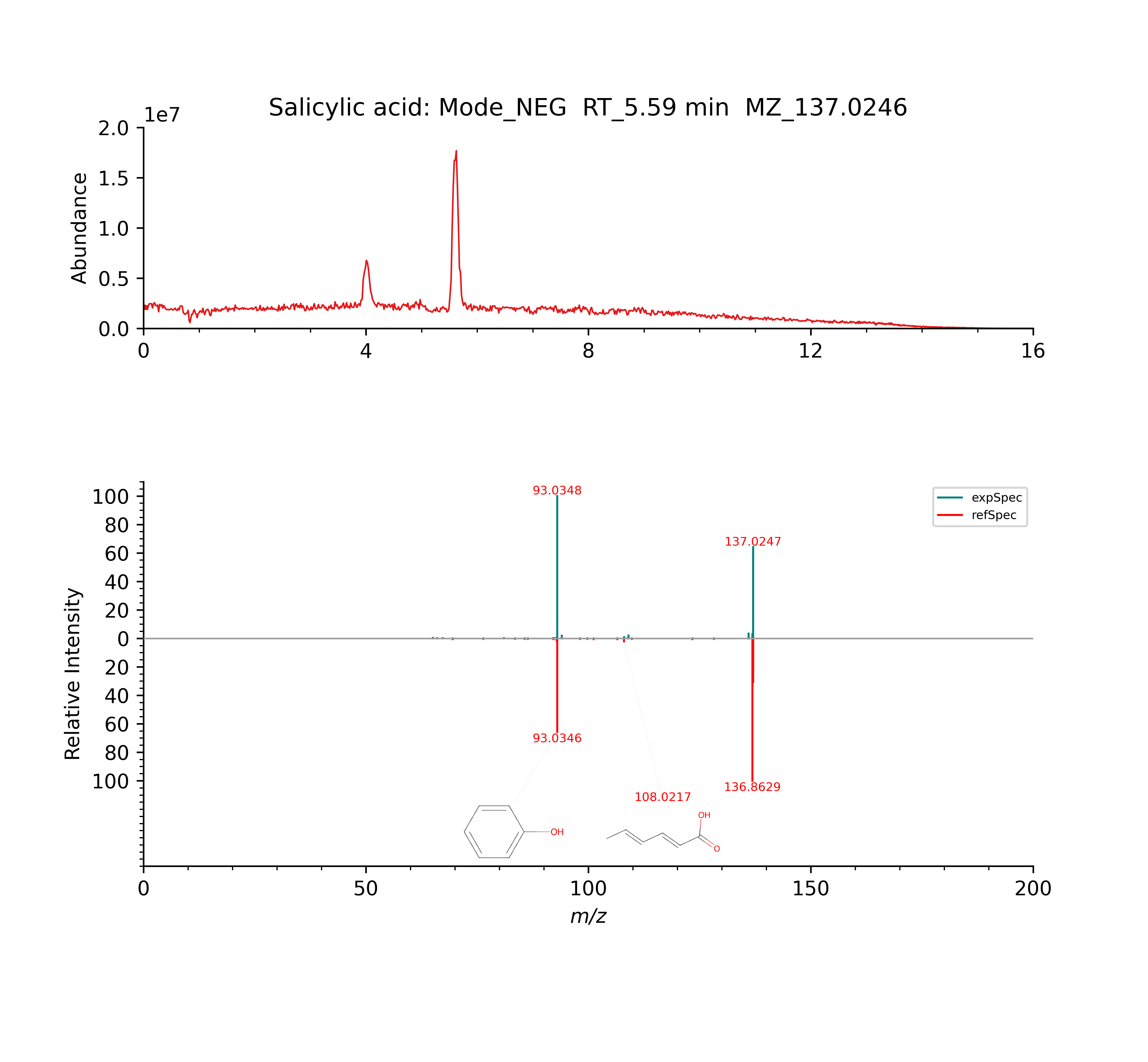

Supplement: Supplementary file 1 [file molecules-29-02840-s001.zip › Supplementary Figure s1/Identification from LuMet-CM datebase/png/compound00123.png]

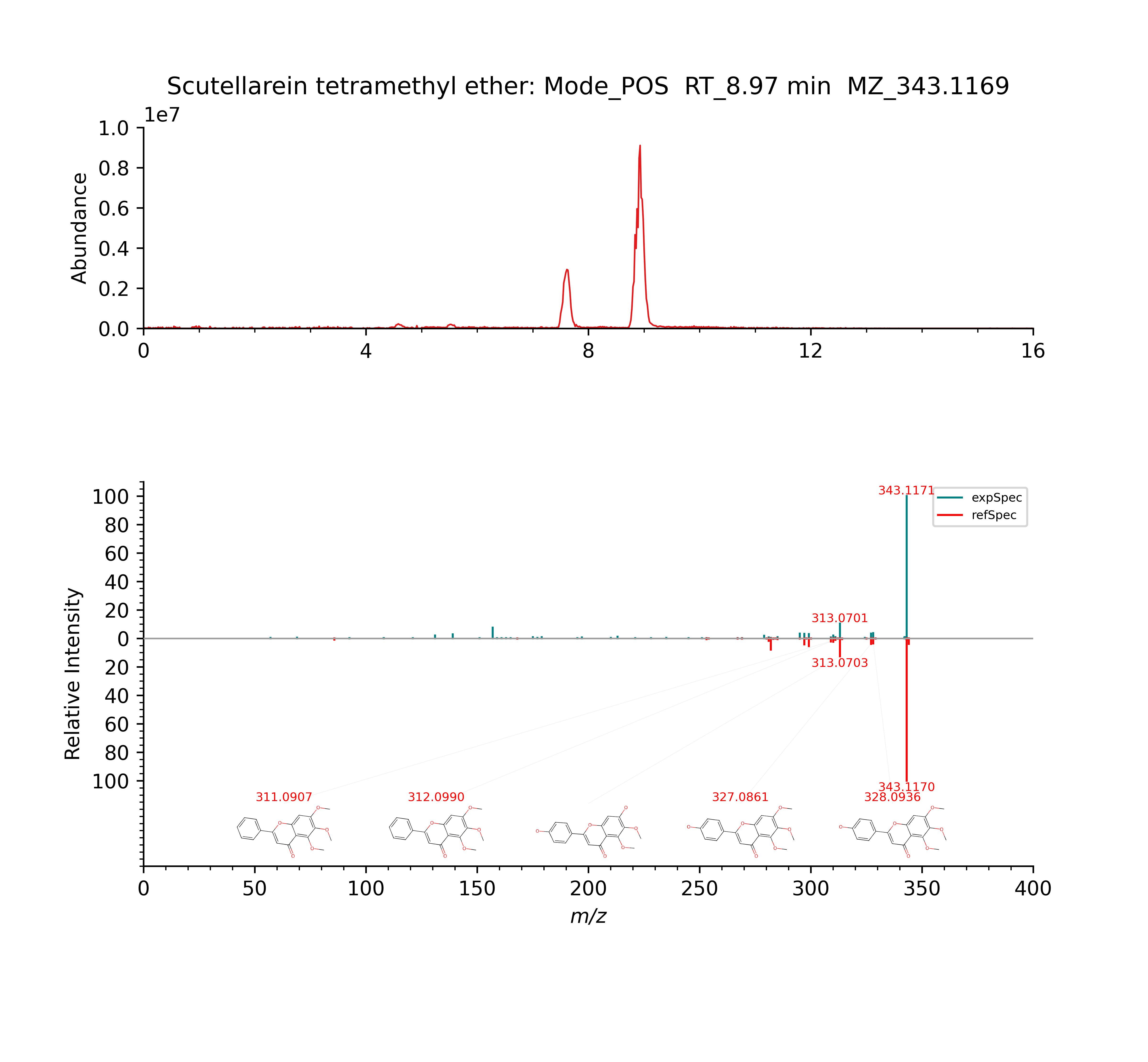

Supplement: Supplementary file 1 [file molecules-29-02840-s001.zip › Supplementary Figure s1/Identification from LuMet-CM datebase/png/compound00124.png]

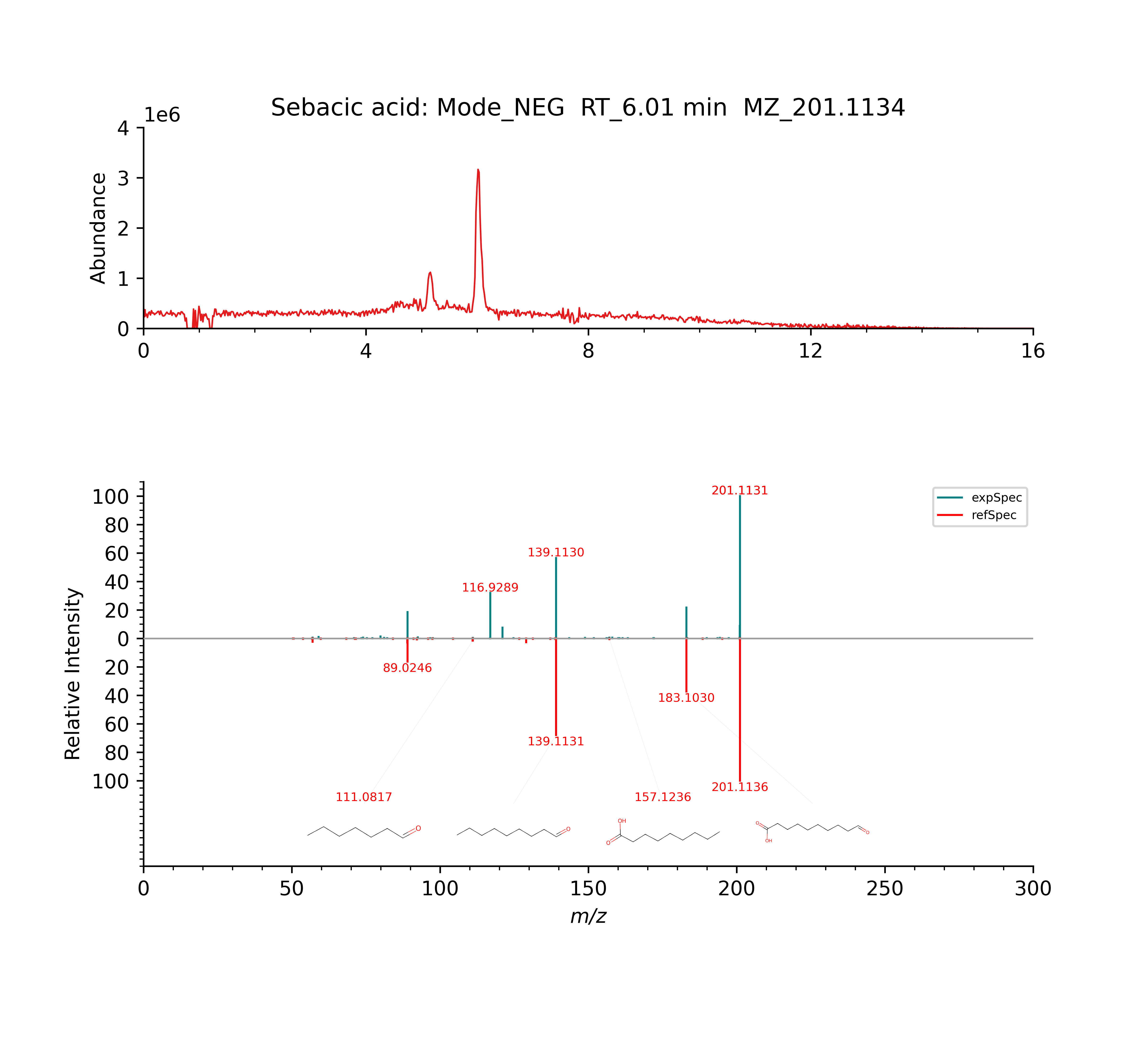

Supplement: Supplementary file 1 [file molecules-29-02840-s001.zip › Supplementary Figure s1/Identification from LuMet-CM datebase/png/compound00125.png]

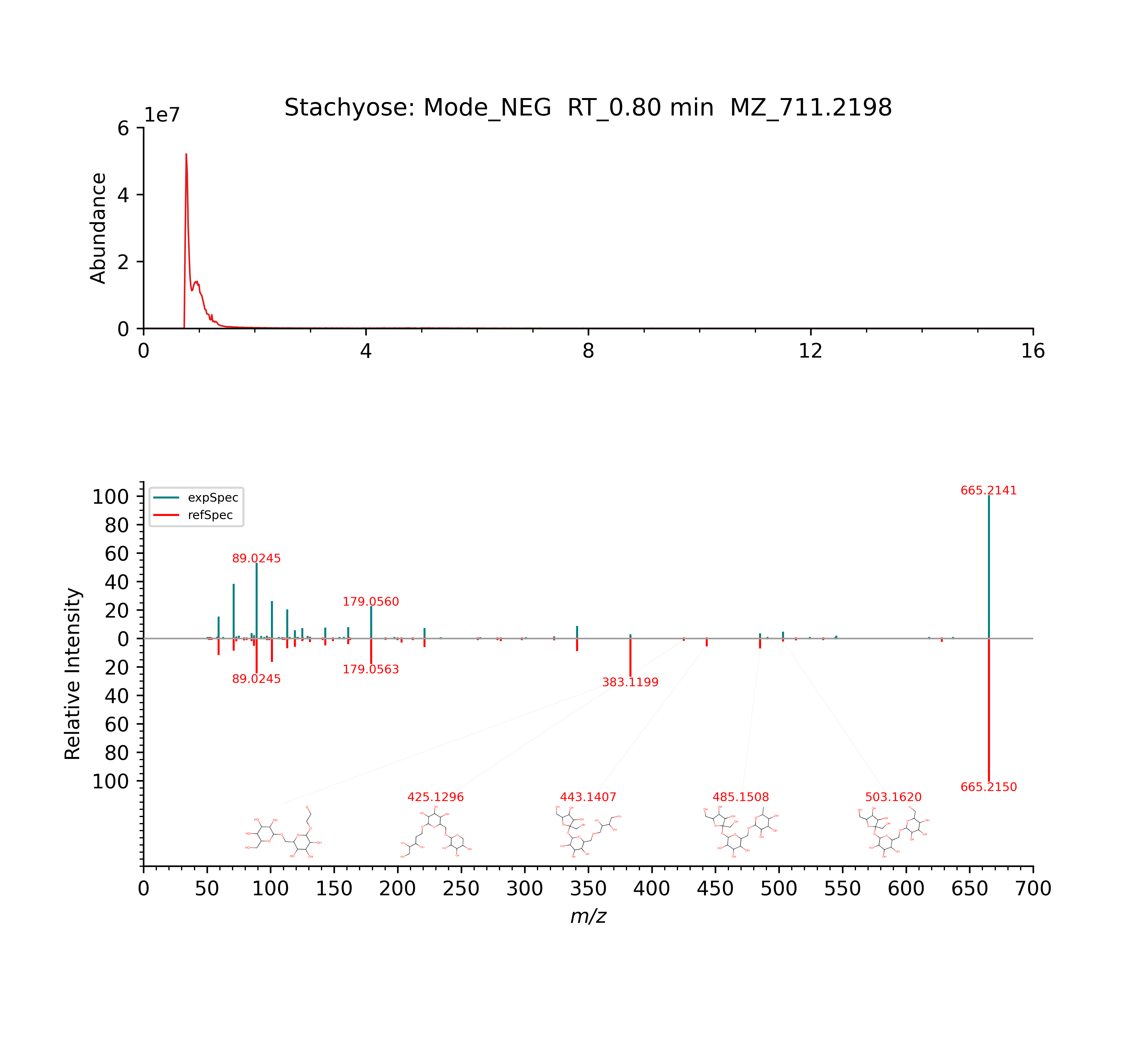

Supplement: Supplementary file 1 [file molecules-29-02840-s001.zip › Supplementary Figure s1/Identification from LuMet-CM datebase/png/compound00126.png]

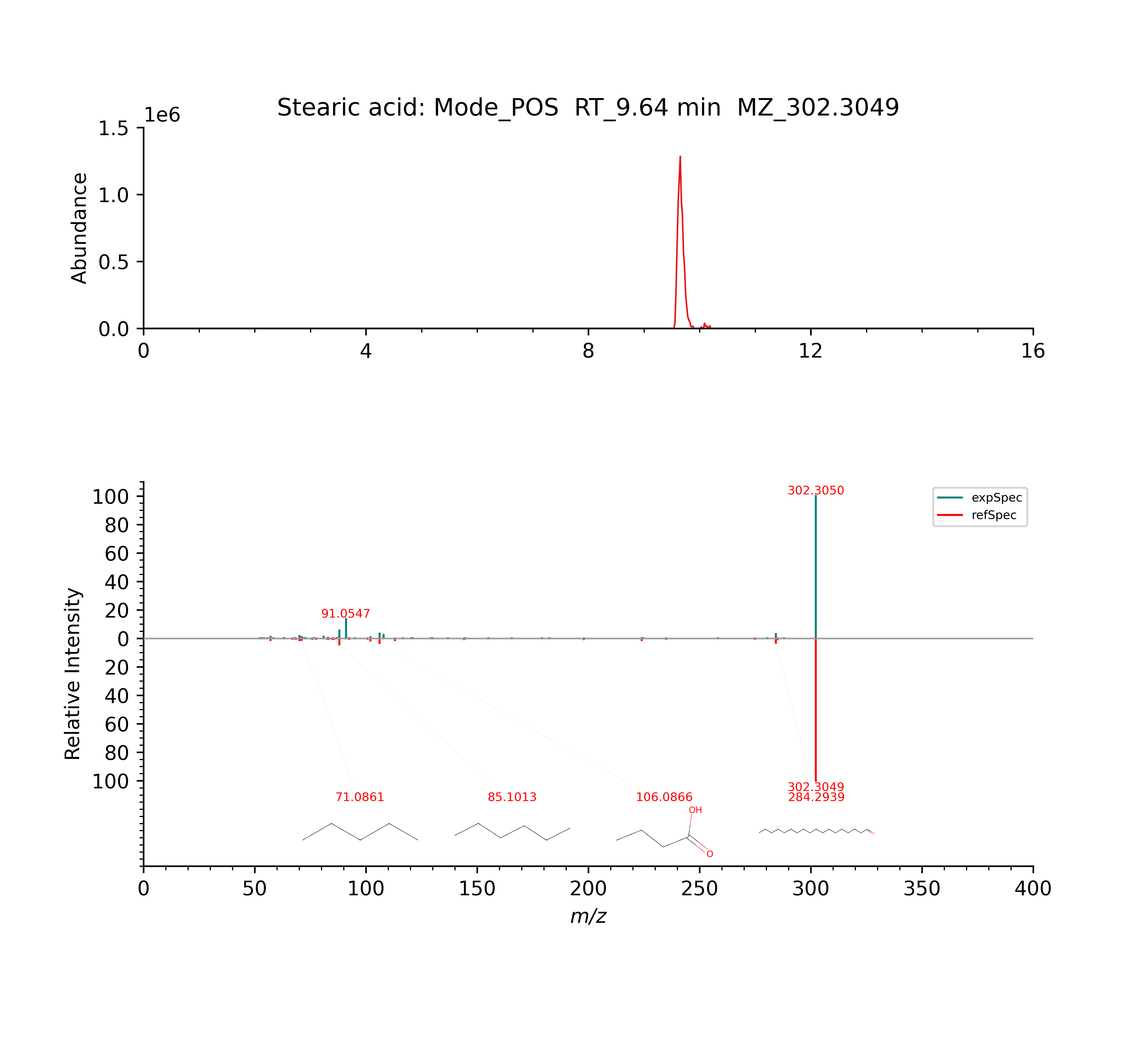

Supplement: Supplementary file 1 [file molecules-29-02840-s001.zip › Supplementary Figure s1/Identification from LuMet-CM datebase/png/compound00127.png]

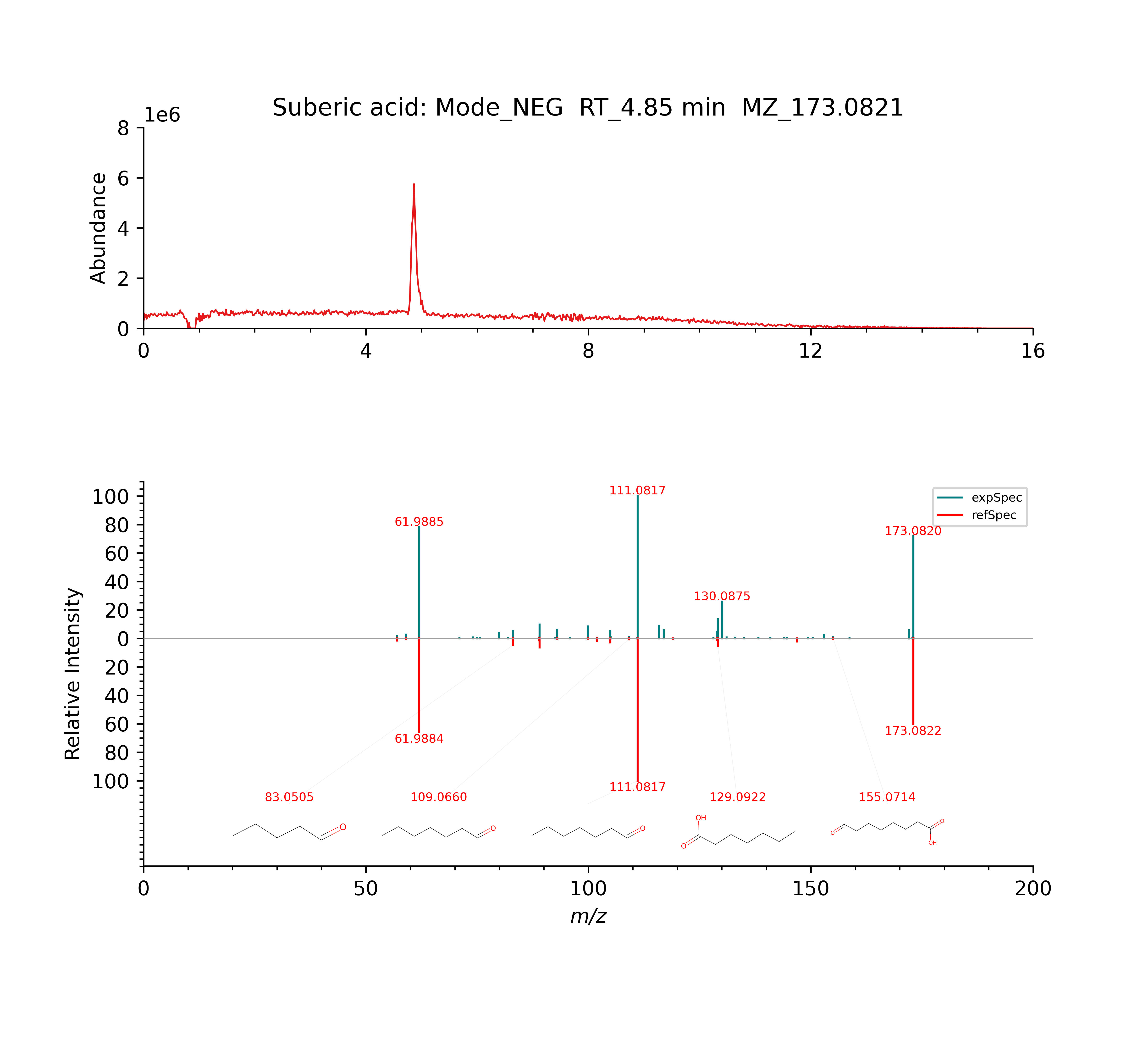

Supplement: Supplementary file 1 [file molecules-29-02840-s001.zip › Supplementary Figure s1/Identification from LuMet-CM datebase/png/compound00128.png]

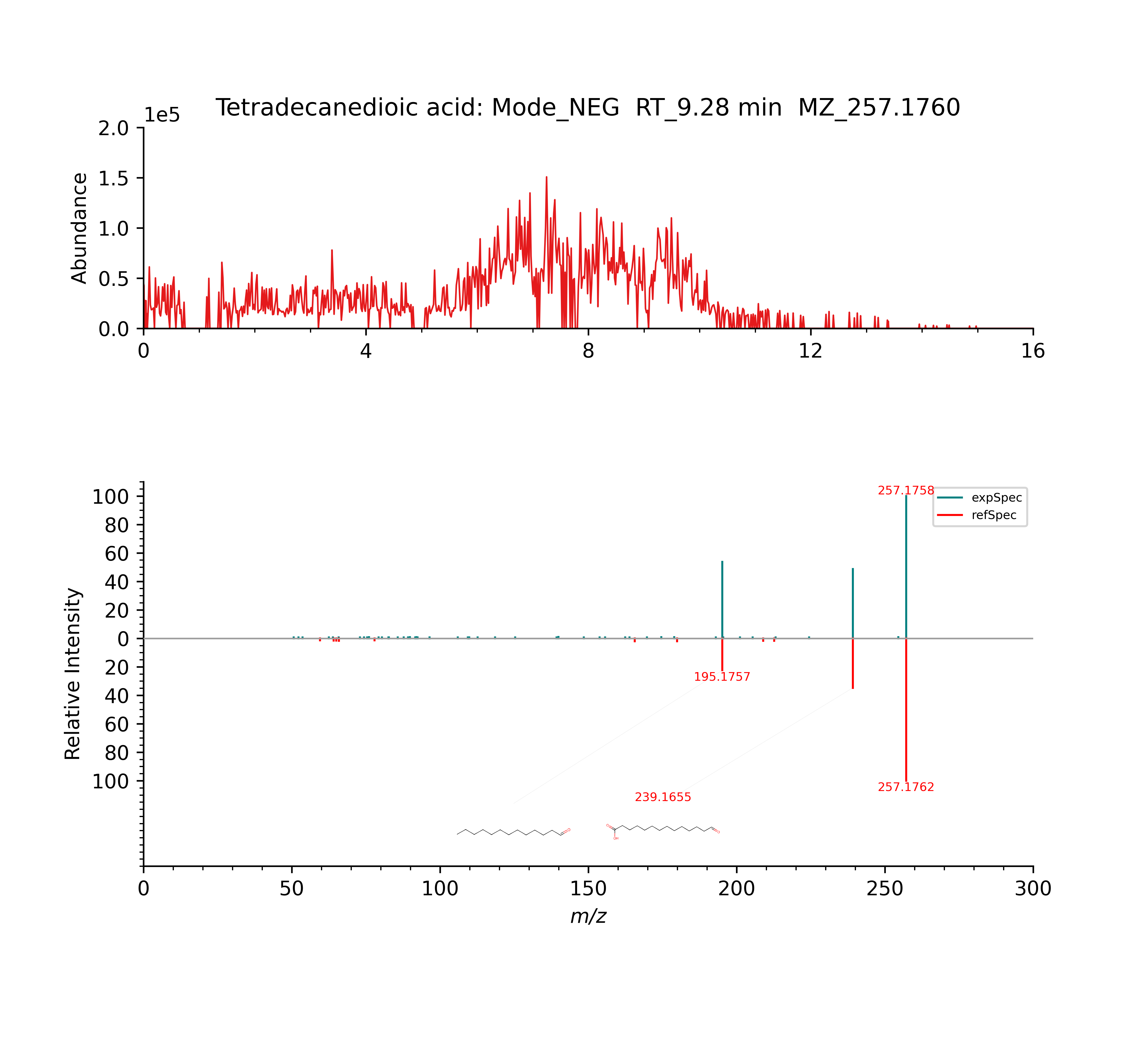

Supplement: Supplementary file 1 [file molecules-29-02840-s001.zip › Supplementary Figure s1/Identification from LuMet-CM datebase/png/compound00129.png]

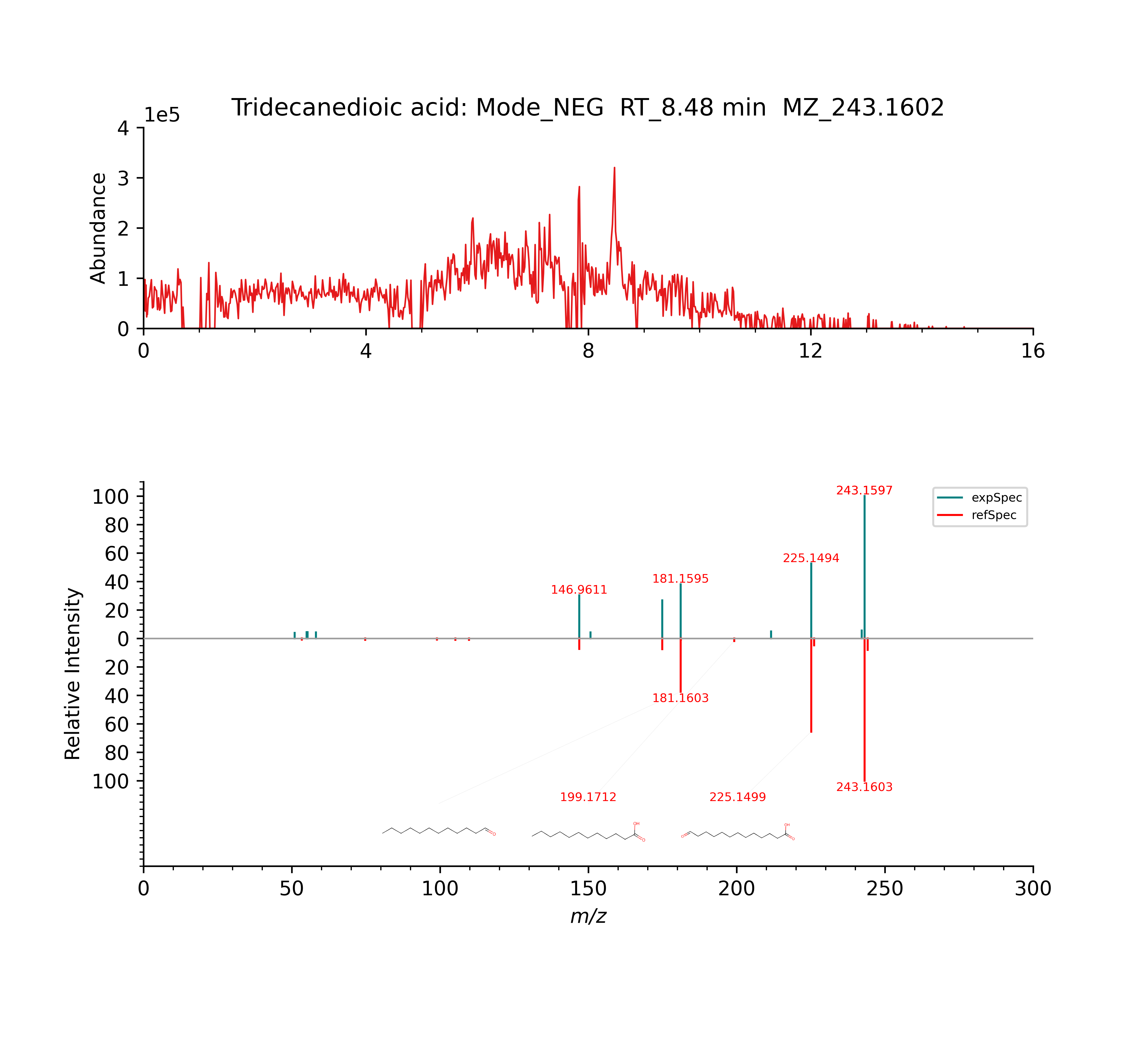

Supplement: Supplementary file 1 [file molecules-29-02840-s001.zip › Supplementary Figure s1/Identification from LuMet-CM datebase/png/compound00130.png]

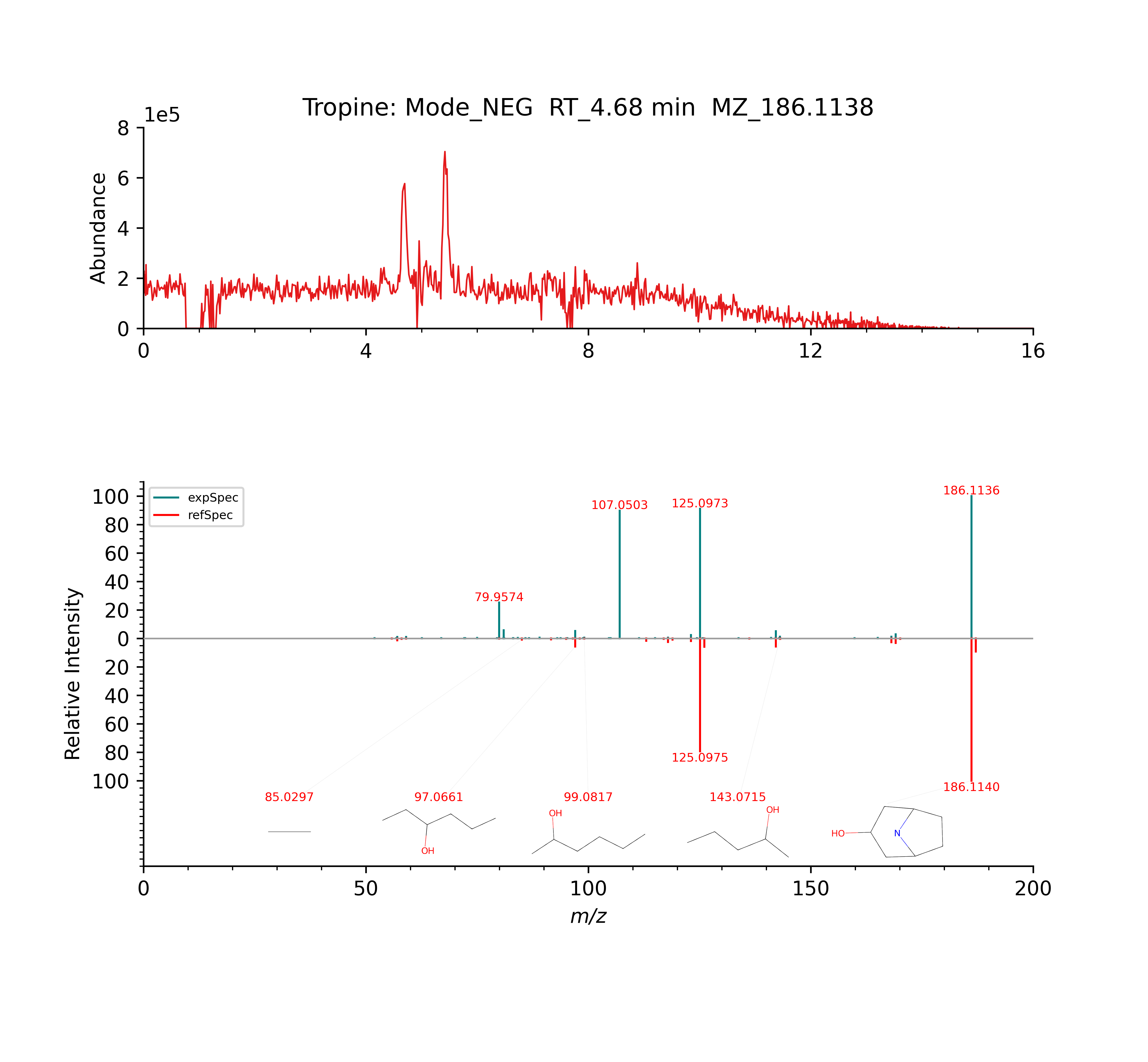

Supplement: Supplementary file 1 [file molecules-29-02840-s001.zip › Supplementary Figure s1/Identification from LuMet-CM datebase/png/compound00131.png]

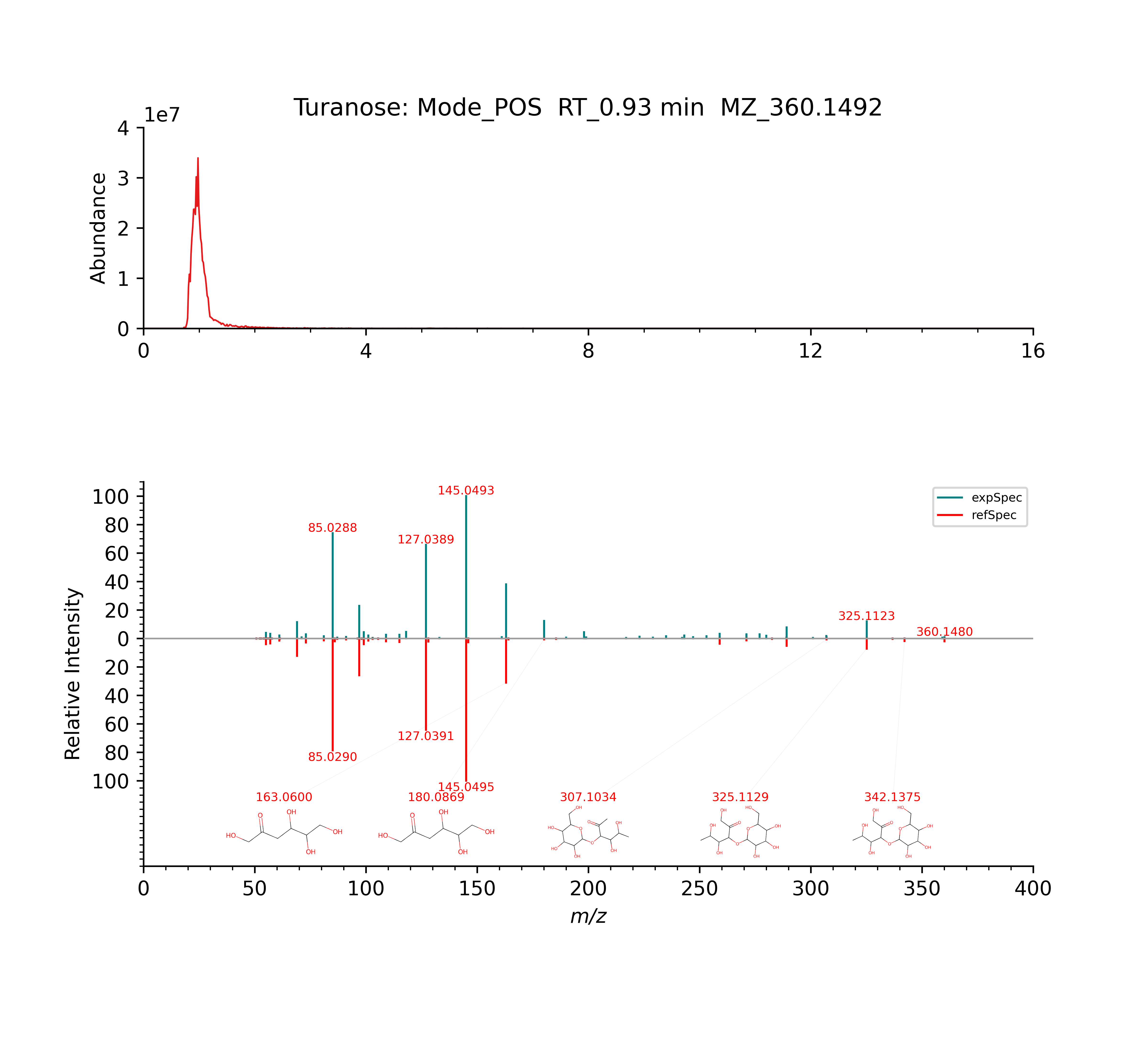

Supplement: Supplementary file 1 [file molecules-29-02840-s001.zip › Supplementary Figure s1/Identification from LuMet-CM datebase/png/compound00132.png]

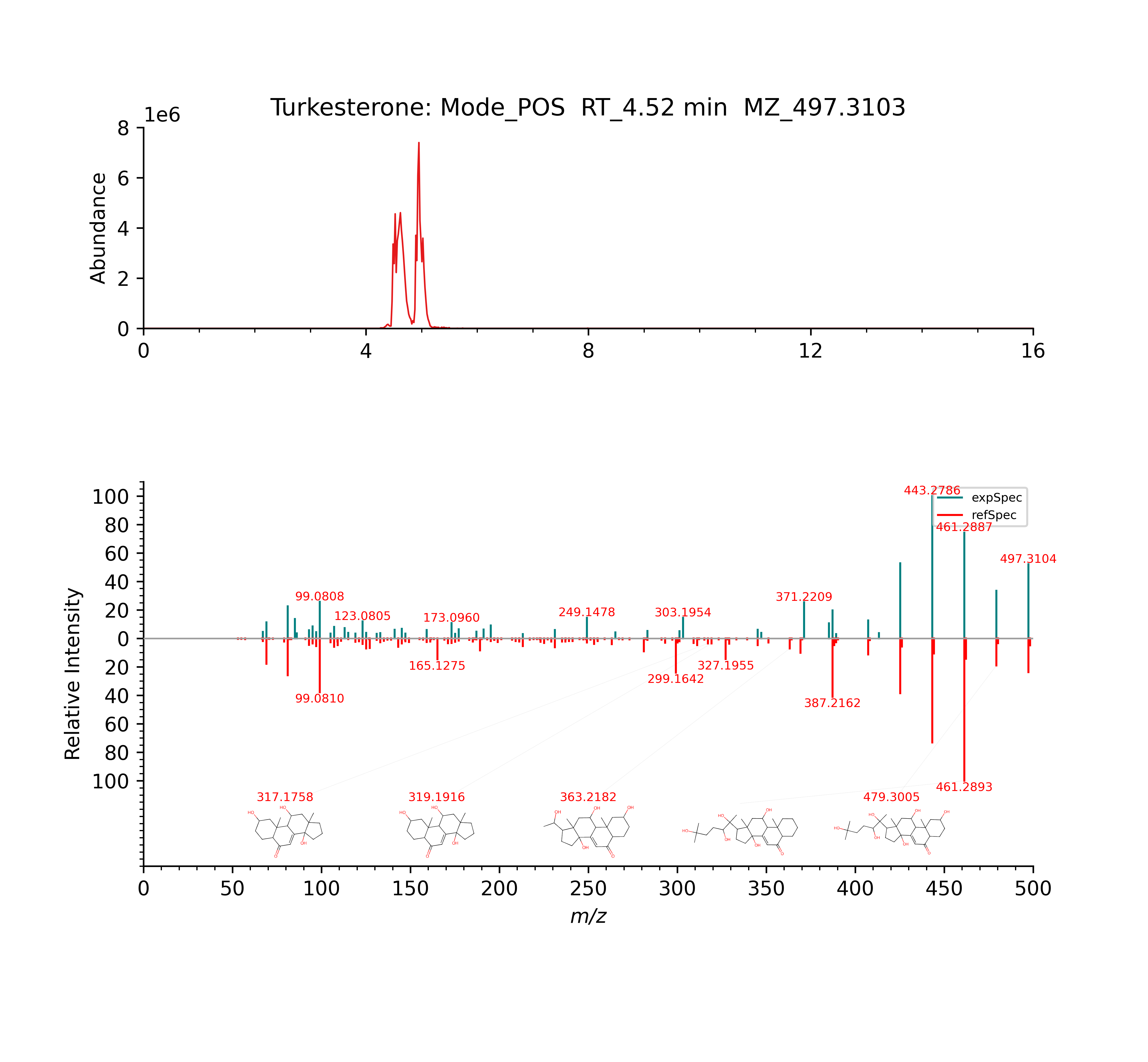

Supplement: Supplementary file 1 [file molecules-29-02840-s001.zip › Supplementary Figure s1/Identification from LuMet-CM datebase/png/compound00133.png]

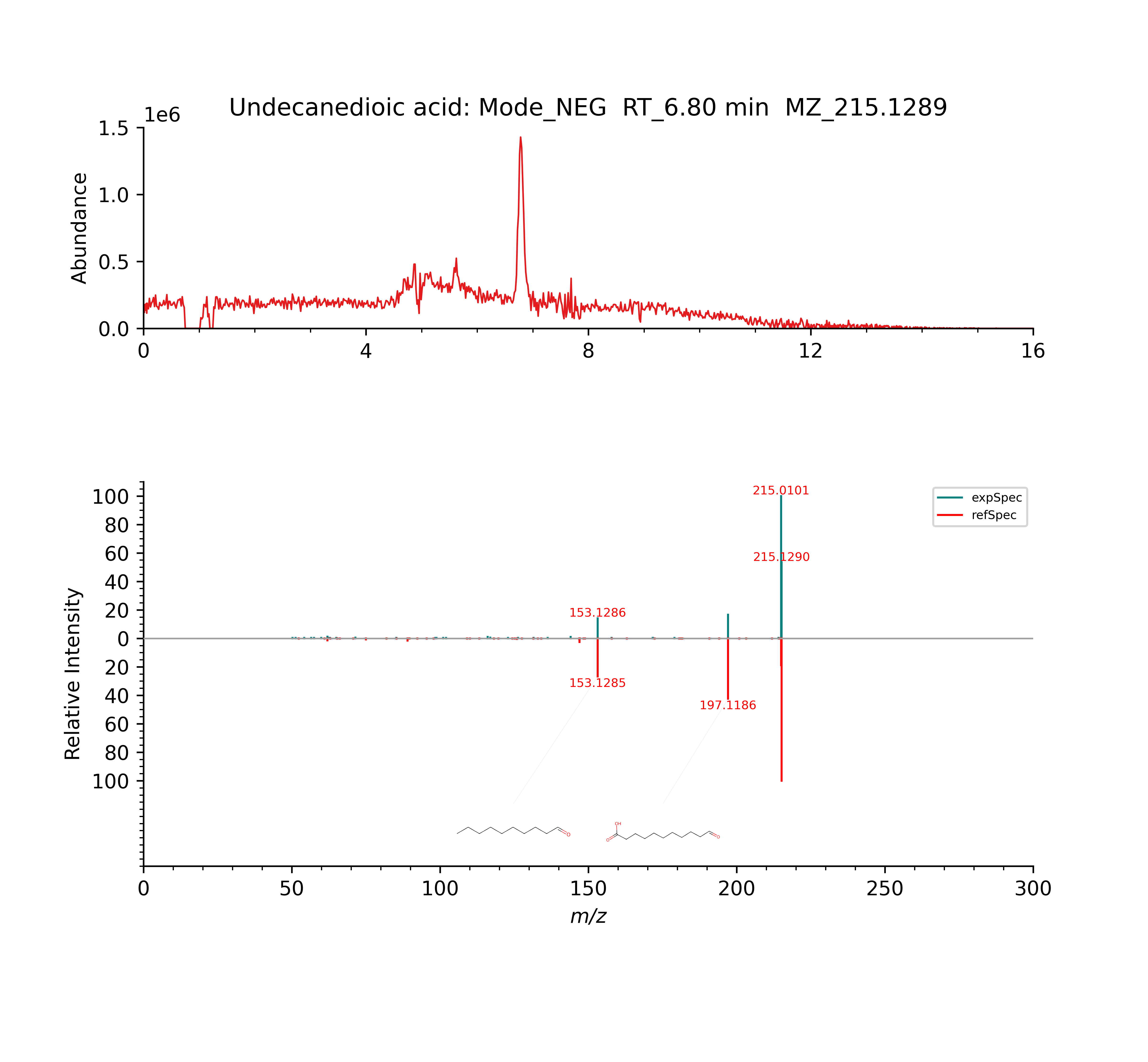

Supplement: Supplementary file 1 [file molecules-29-02840-s001.zip › Supplementary Figure s1/Identification from LuMet-CM datebase/png/compound00134.png]

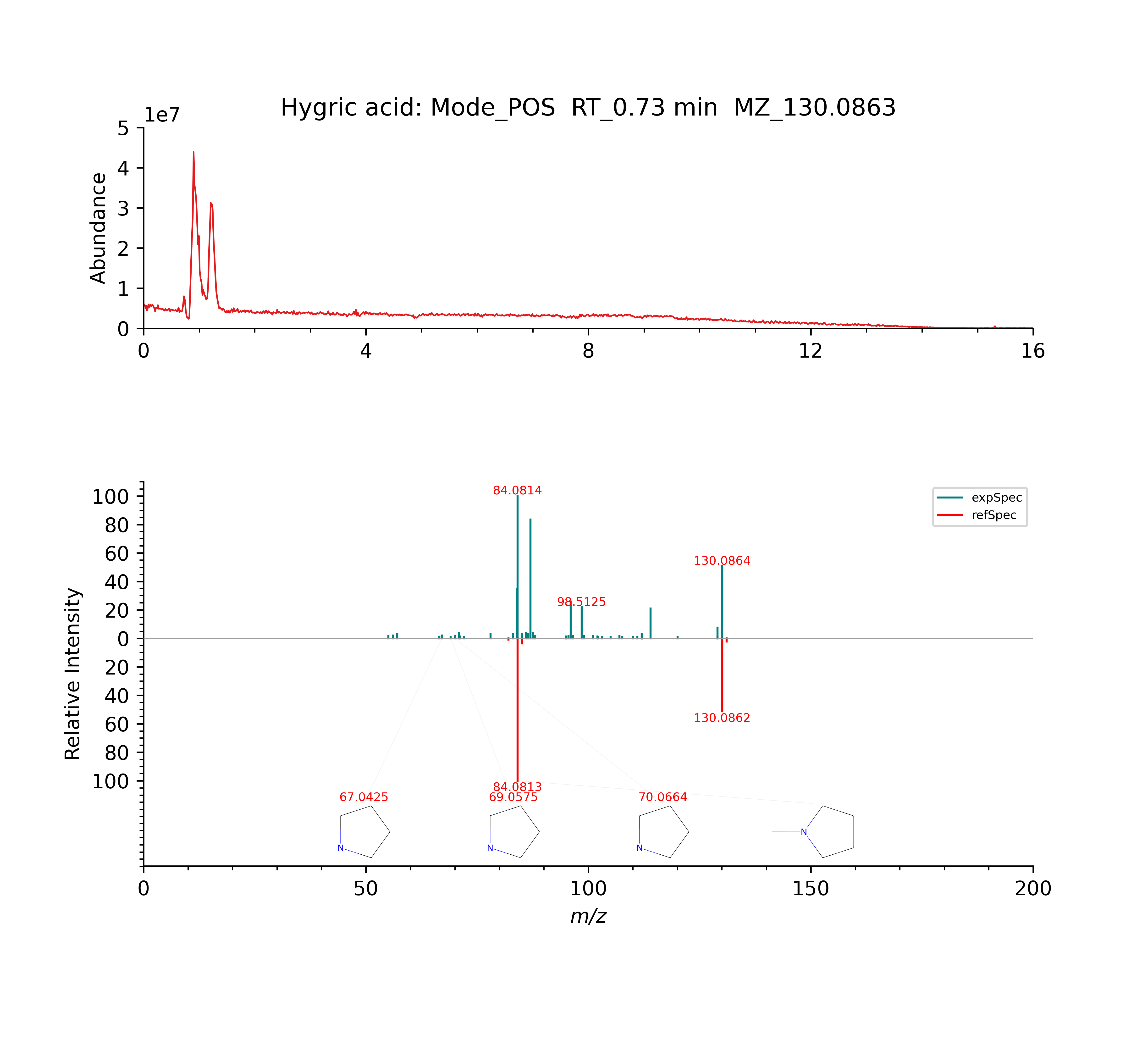

Supplement: Supplementary file 1 [file molecules-29-02840-s001.zip › Supplementary Figure s1/Identification from LuMet-CM datebase/png/compound00137.png]

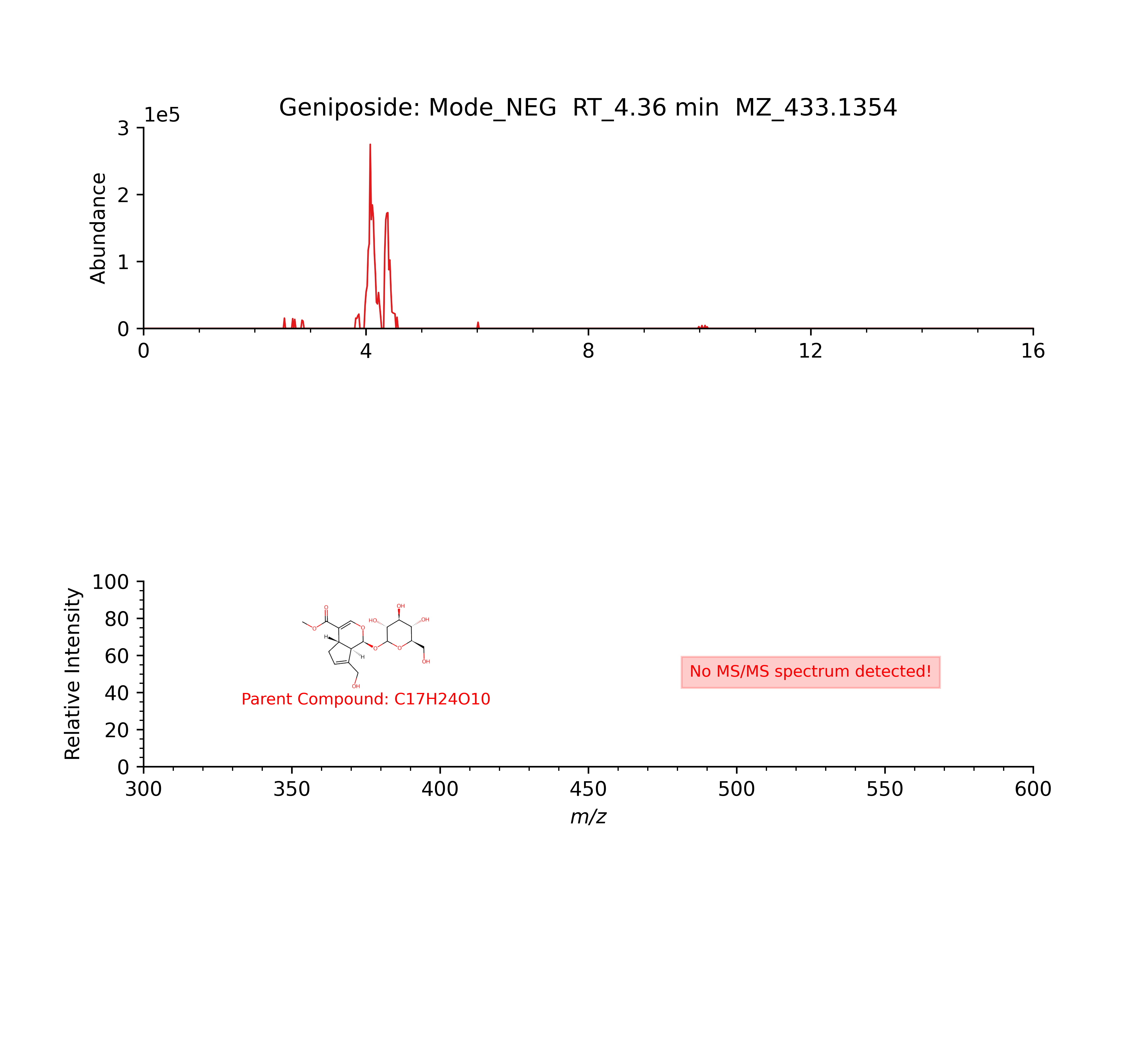

Supplement: Supplementary file 1 [file molecules-29-02840-s001.zip › Supplementary Figure s1/Identification from LuMet-CM datebase/png/compound00138.png]

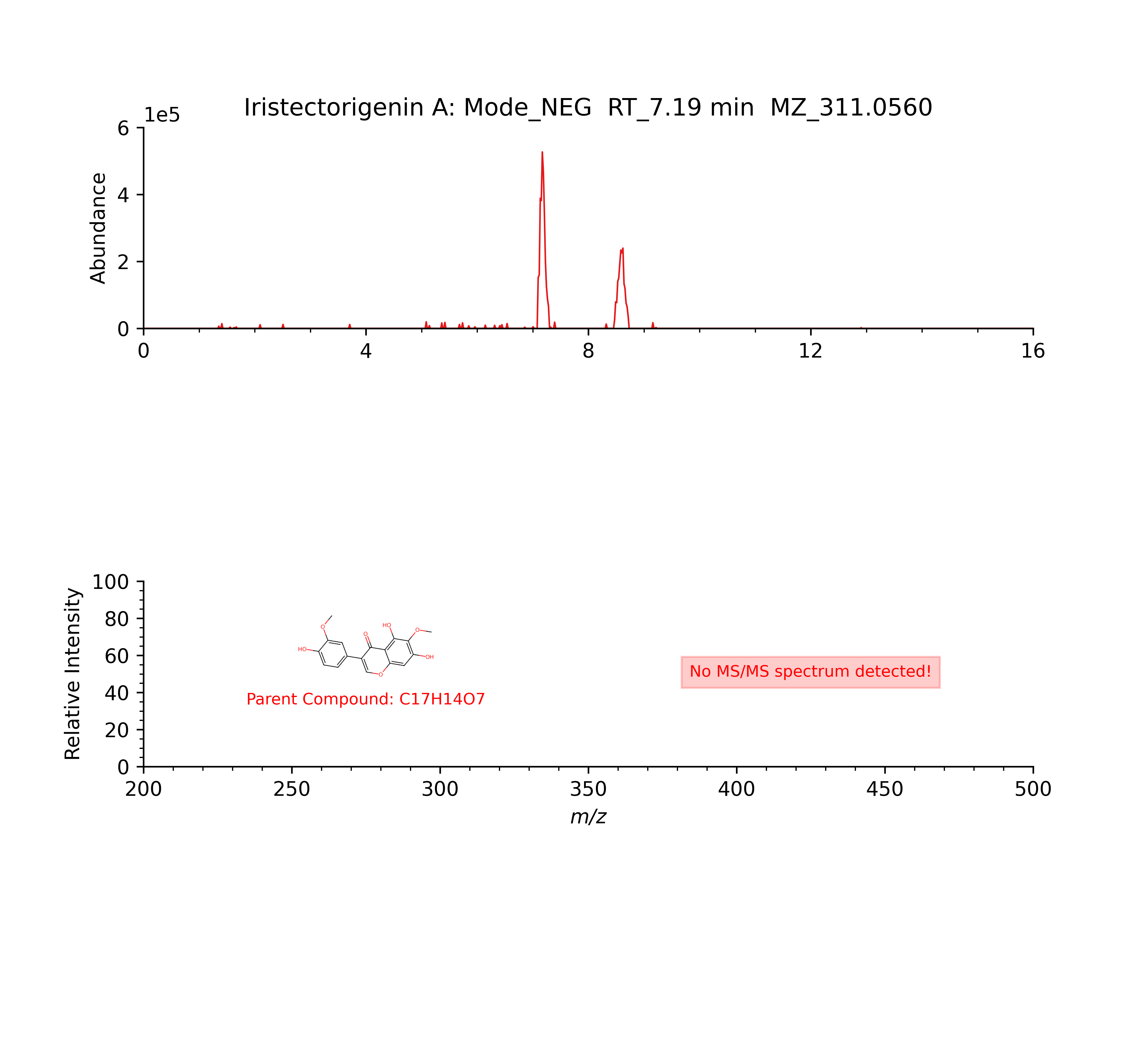

Supplement: Supplementary file 1 [file molecules-29-02840-s001.zip › Supplementary Figure s1/Identification from LuMet-CM datebase/png/compound00141.png]

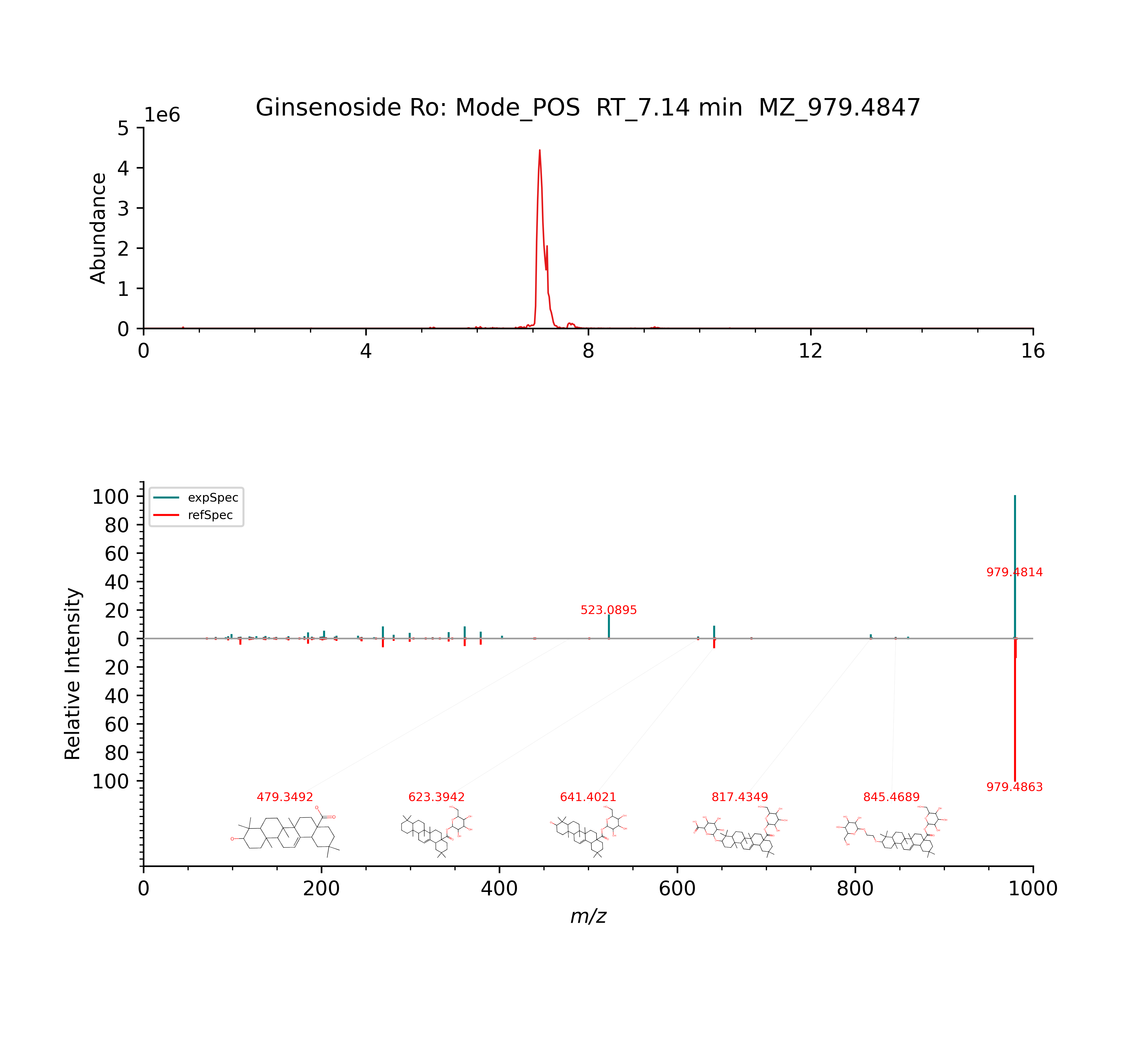

Supplement: Supplementary file 1 [file molecules-29-02840-s001.zip › Supplementary Figure s1/Identification from LuMet-CM datebase/png/compound00143.png]

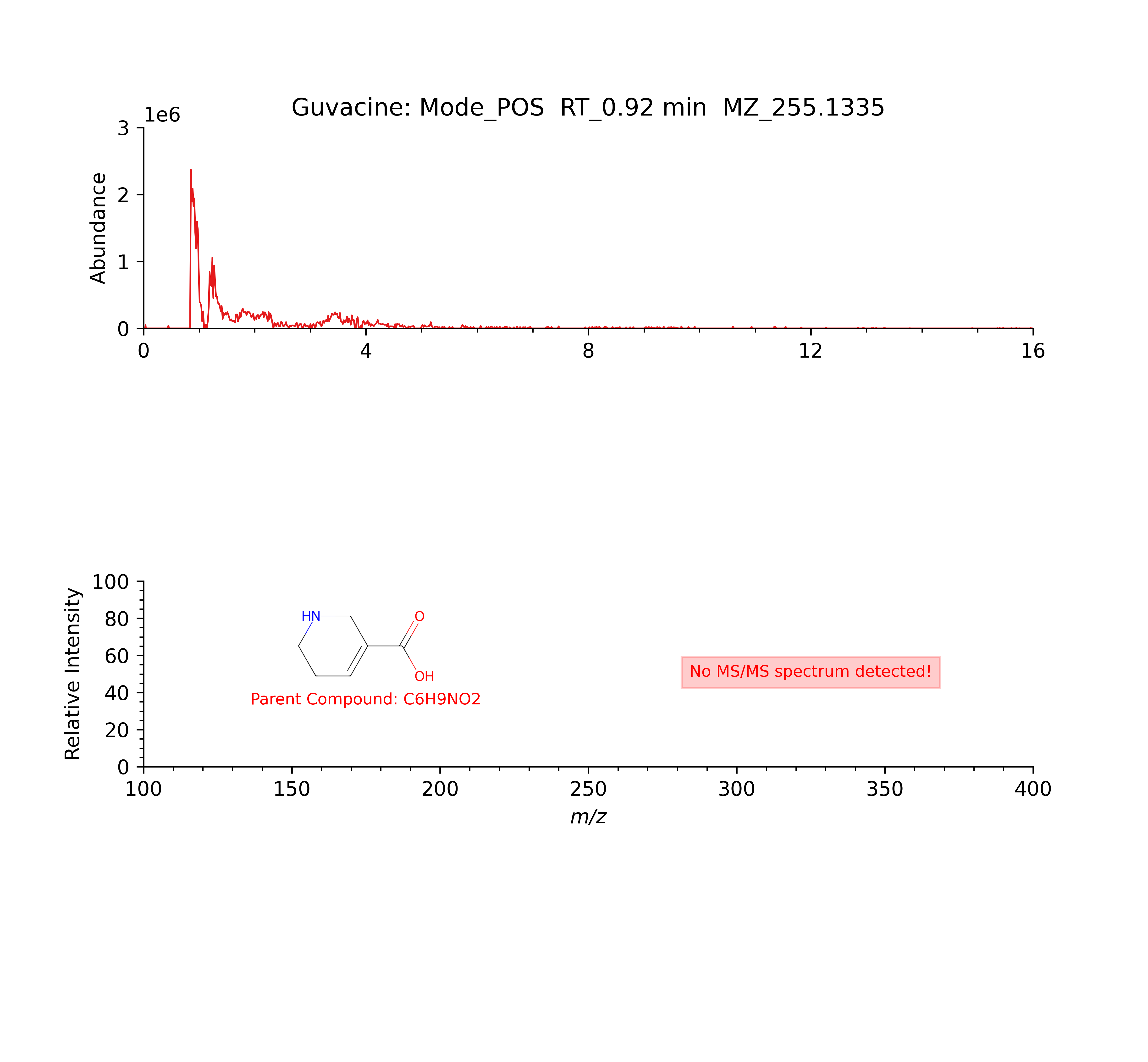

Supplement: Supplementary file 1 [file molecules-29-02840-s001.zip › Supplementary Figure s1/Identification from LuMet-CM datebase/png/compound00144.png]

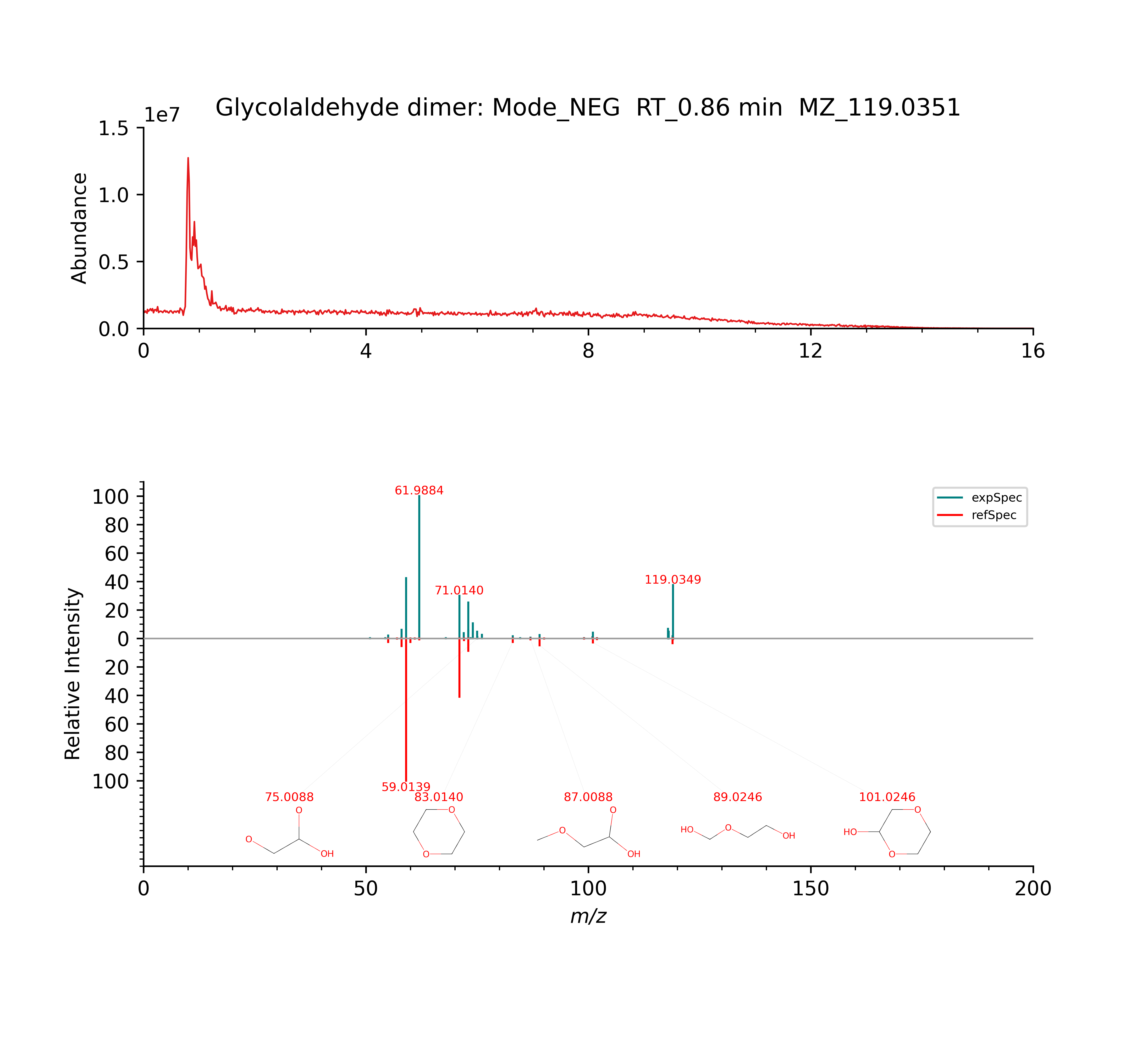

Supplement: Supplementary file 1 [file molecules-29-02840-s001.zip › Supplementary Figure s1/Identification from LuMet-CM datebase/png/compound00146.png]

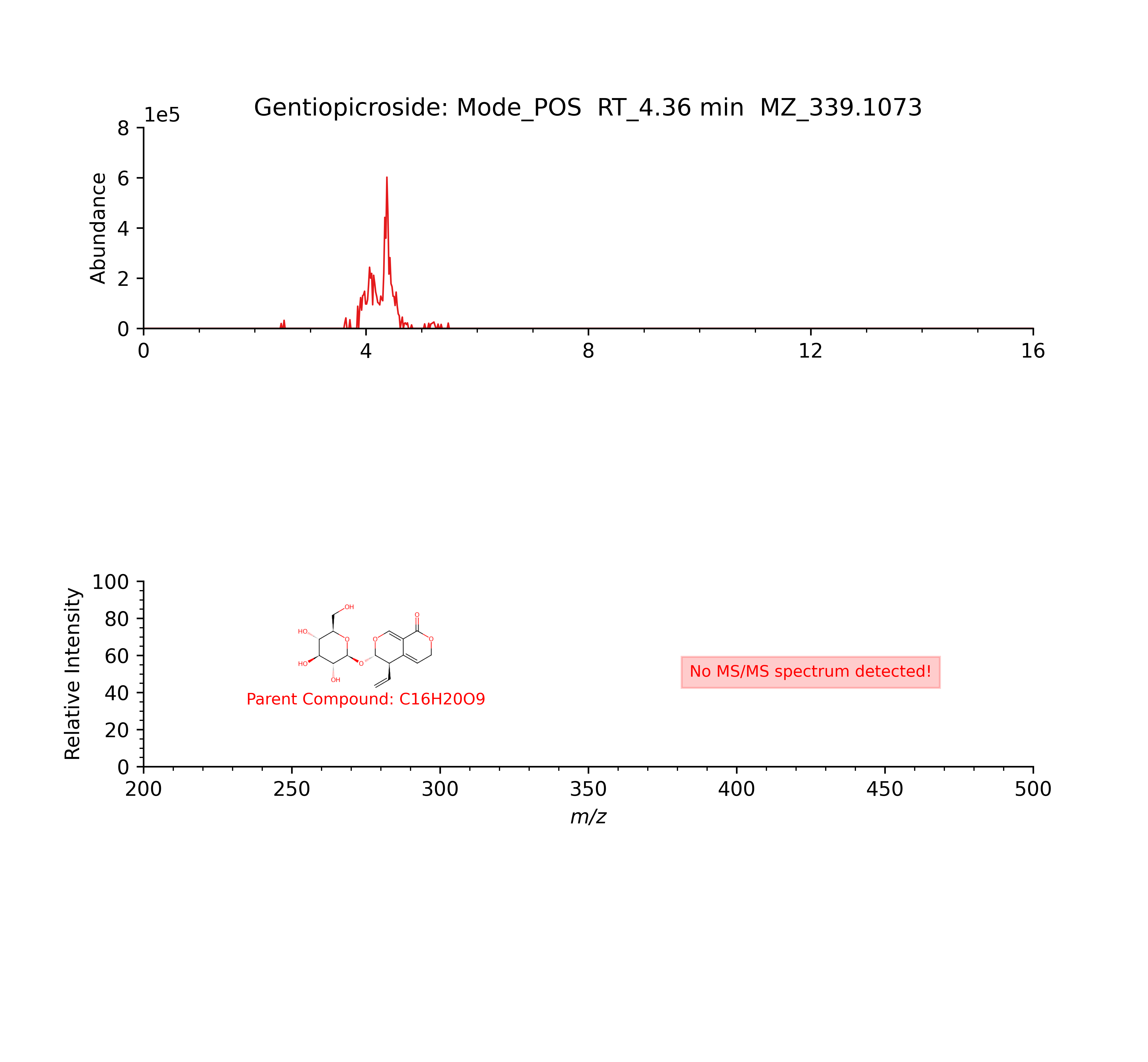

Supplement: Supplementary file 1 [file molecules-29-02840-s001.zip › Supplementary Figure s1/Identification from LuMet-CM datebase/png/compound00148.png]

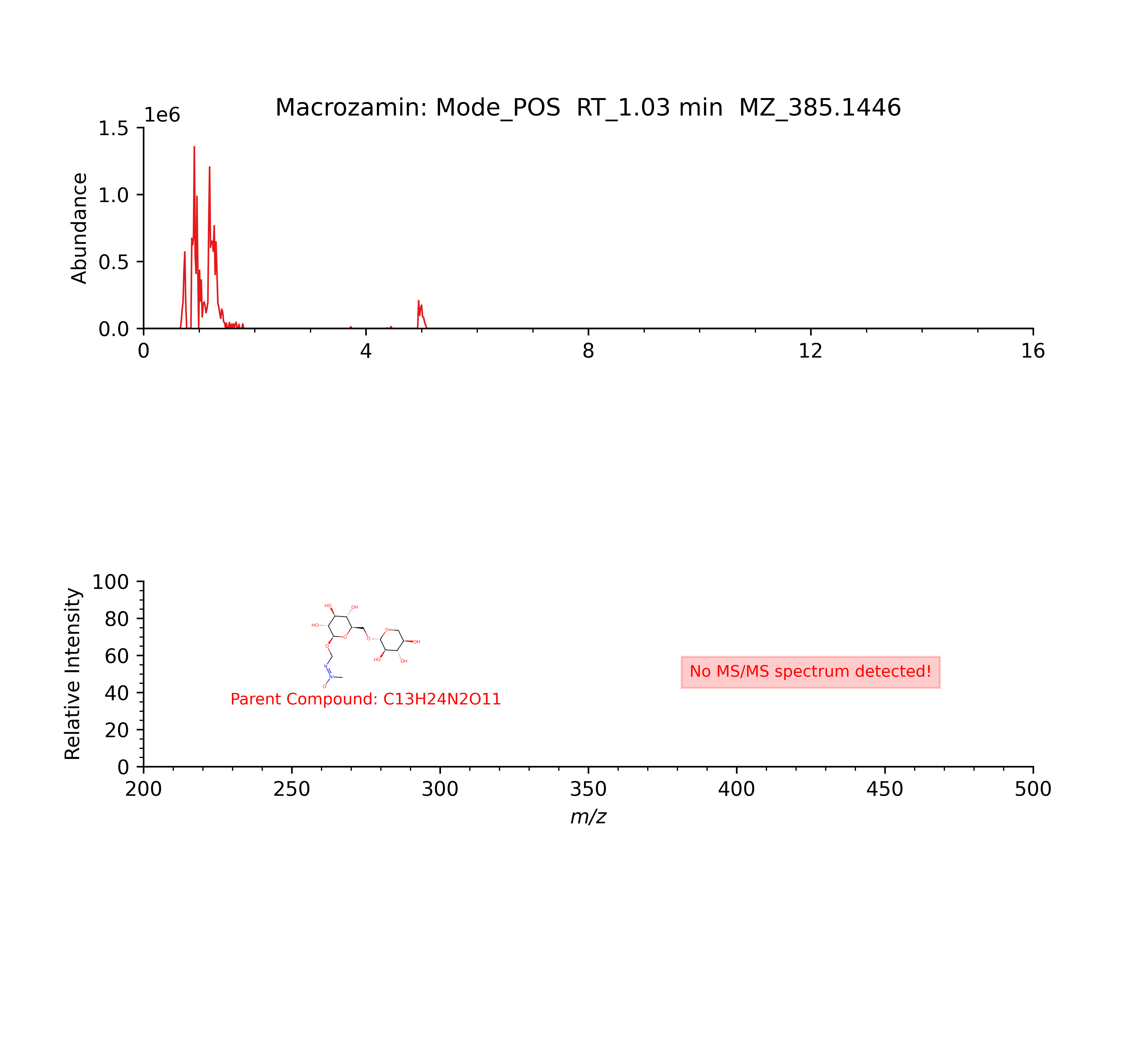

Supplement: Supplementary file 1 [file molecules-29-02840-s001.zip › Supplementary Figure s1/Identification from LuMet-CM datebase/png/compound00151.png]

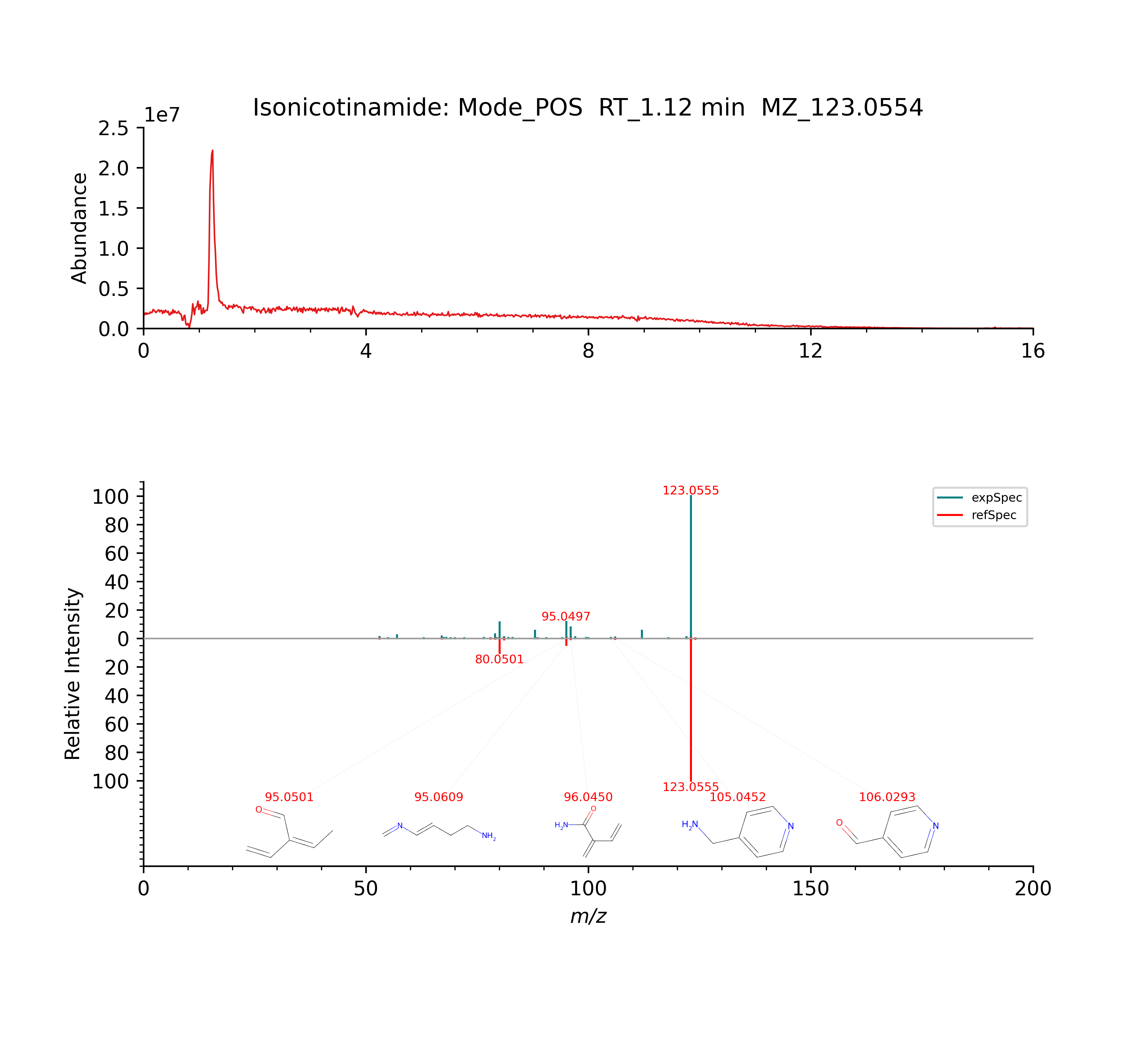

Supplement: Supplementary file 1 [file molecules-29-02840-s001.zip › Supplementary Figure s1/Identification from LuMet-CM datebase/png/compound00152.png]

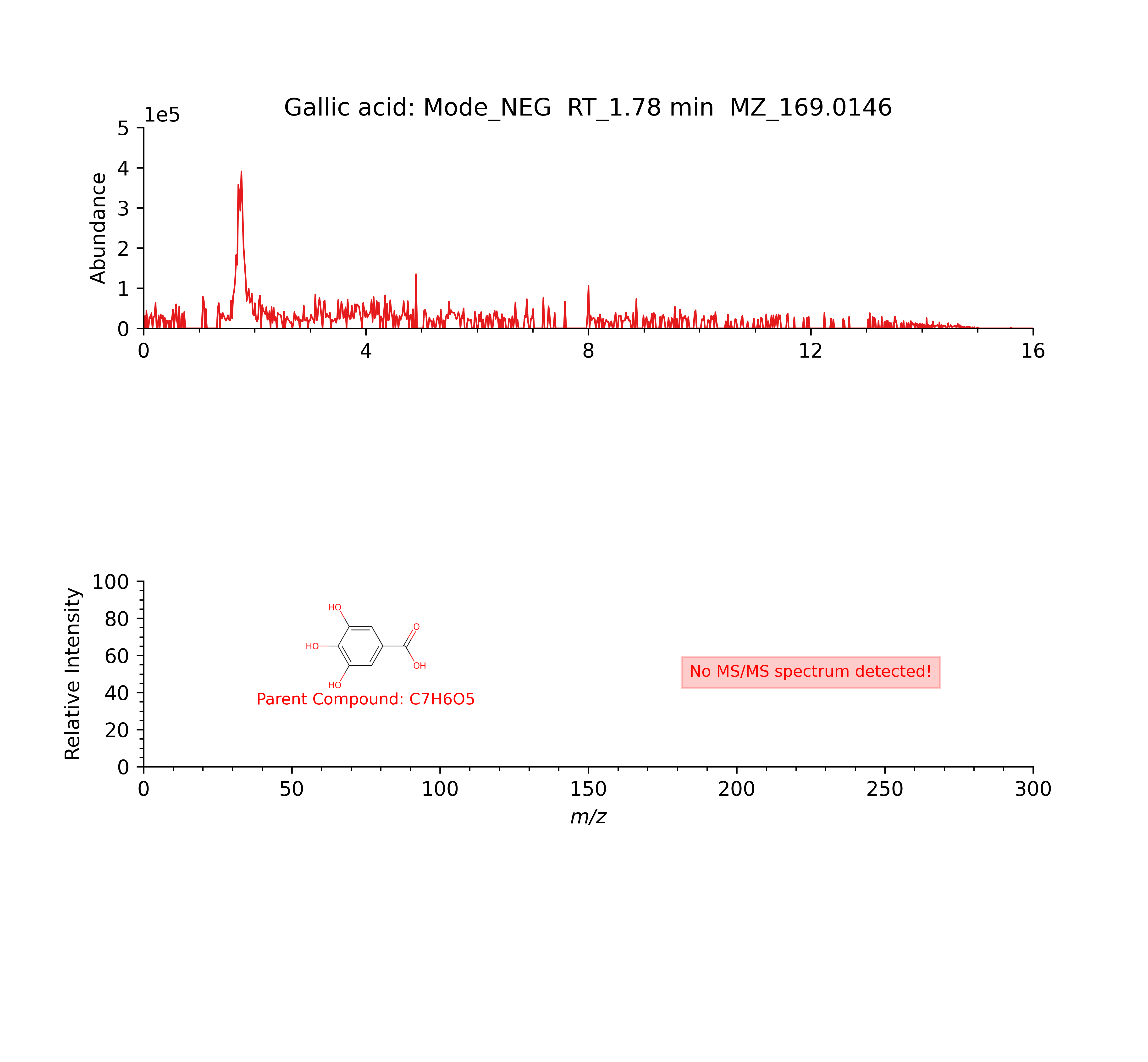

Supplement: Supplementary file 1 [file molecules-29-02840-s001.zip › Supplementary Figure s1/Identification from LuMet-CM datebase/png/compound00154.png]

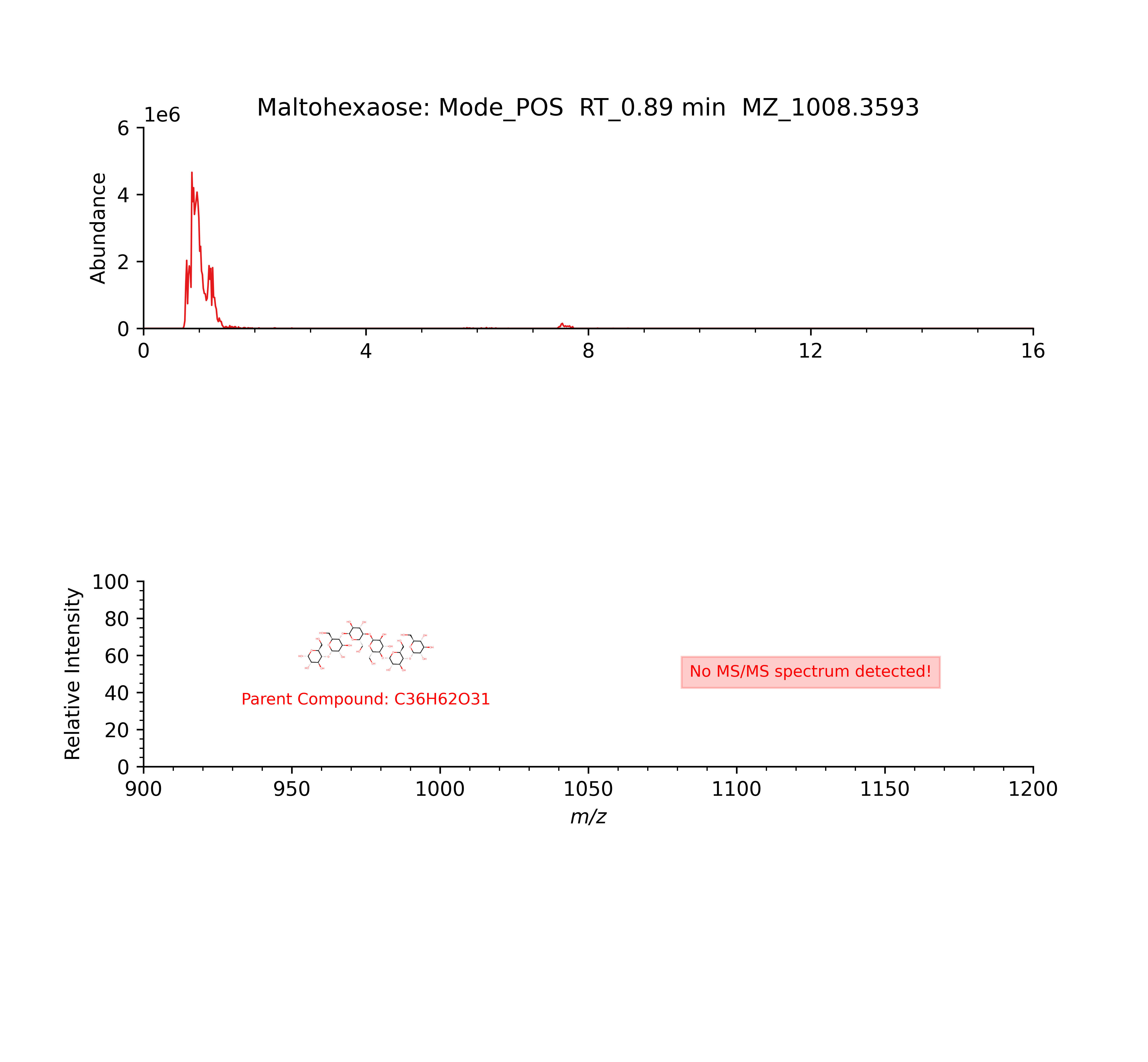

Supplement: Supplementary file 1 [file molecules-29-02840-s001.zip › Supplementary Figure s1/Identification from LuMet-CM datebase/png/compound00155.png]

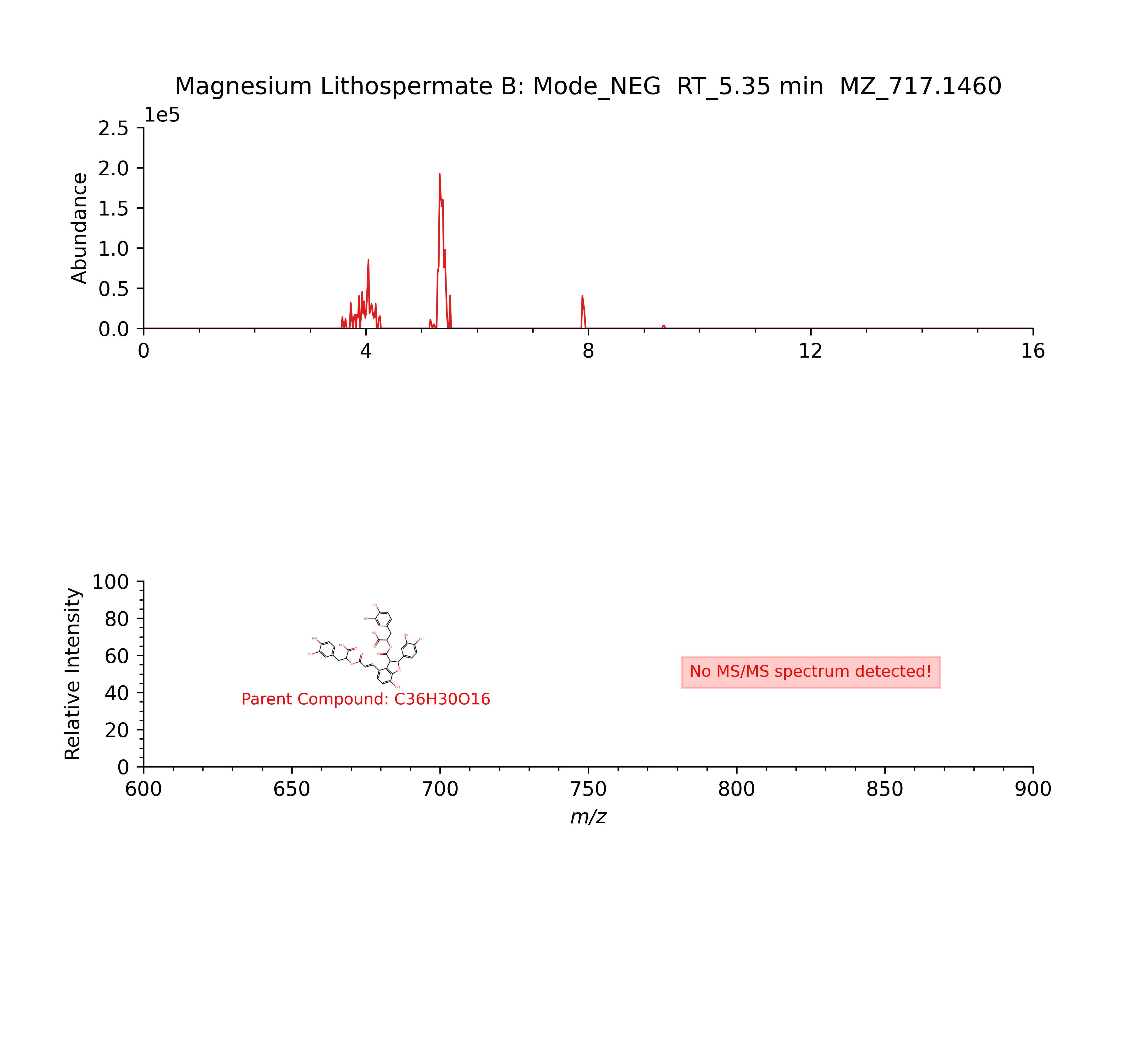

Supplement: Supplementary file 1 [file molecules-29-02840-s001.zip › Supplementary Figure s1/Identification from LuMet-CM datebase/png/compound00156.png]

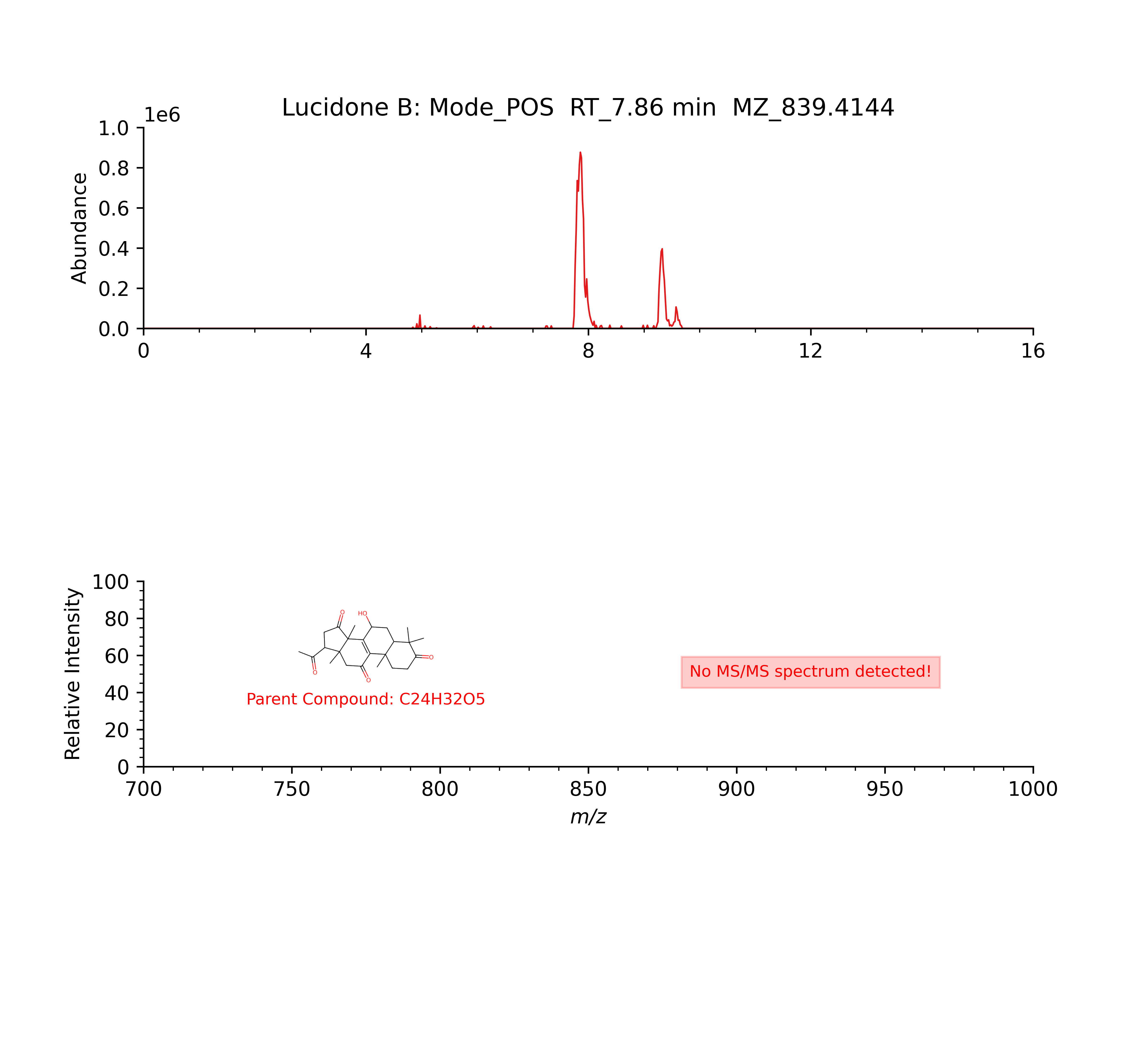

Supplement: Supplementary file 1 [file molecules-29-02840-s001.zip › Supplementary Figure s1/Identification from LuMet-CM datebase/png/compound00158.png]

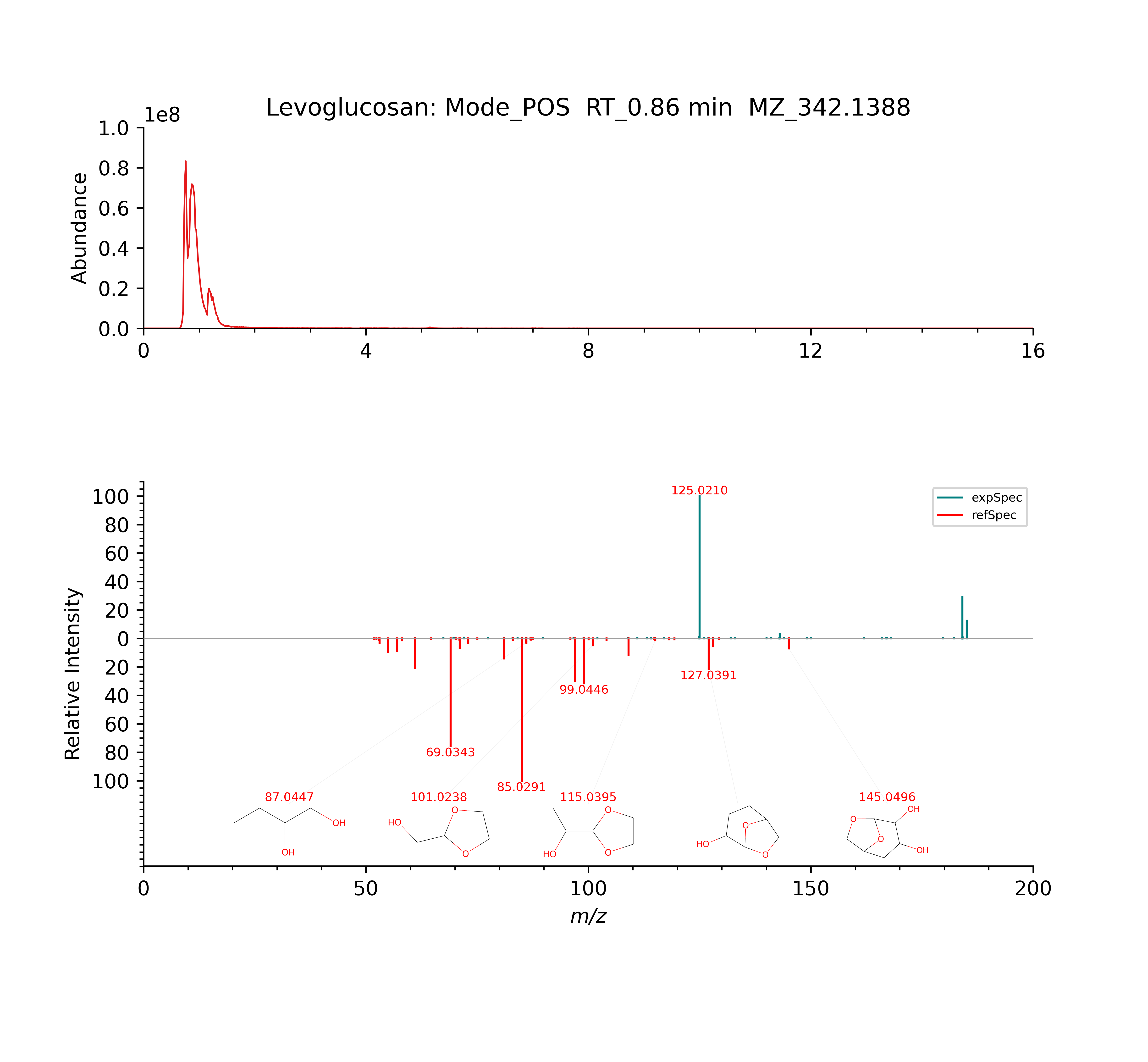

Supplement: Supplementary file 1 [file molecules-29-02840-s001.zip › Supplementary Figure s1/Identification from LuMet-CM datebase/png/compound00162.png]

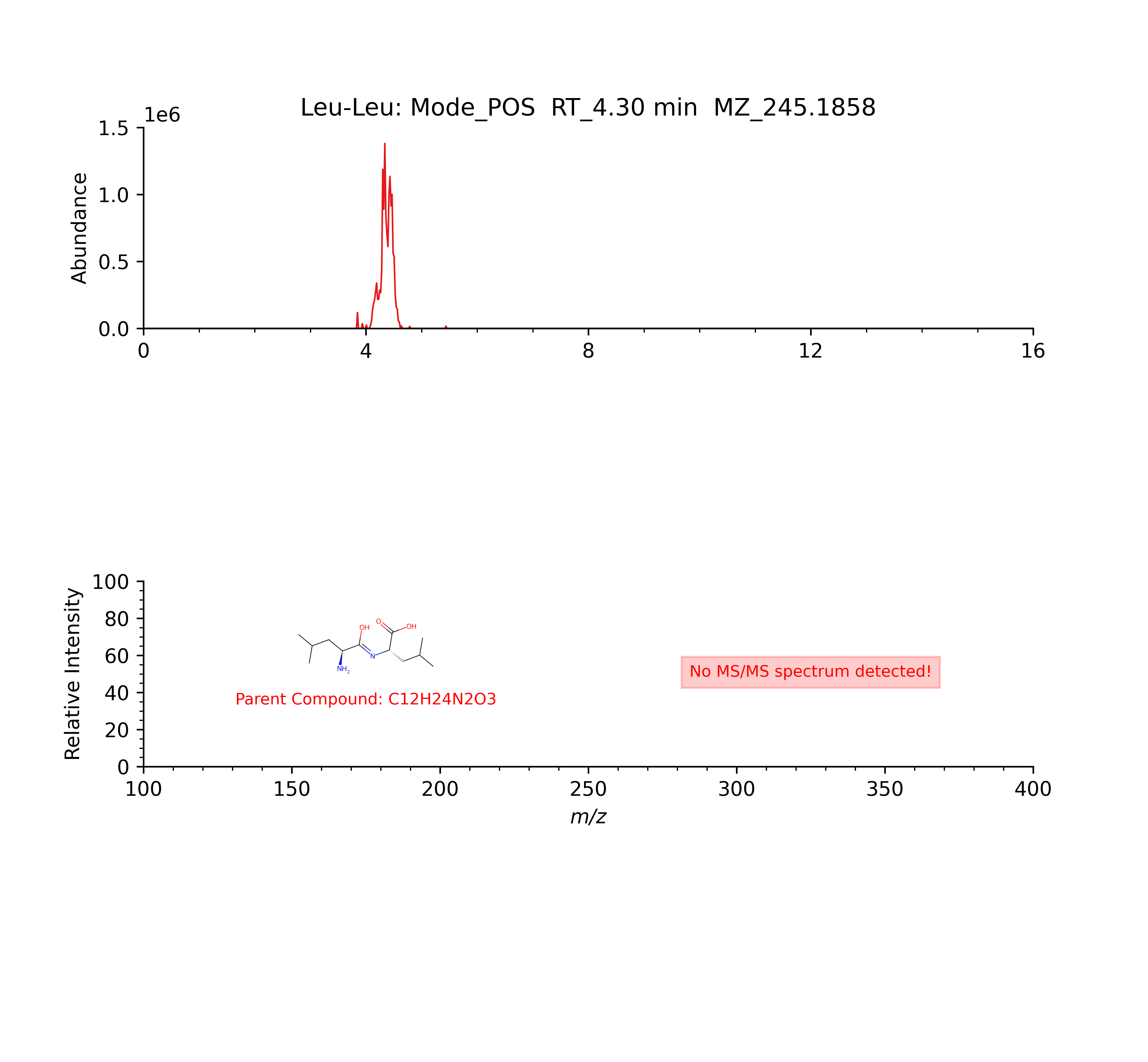

Supplement: Supplementary file 1 [file molecules-29-02840-s001.zip › Supplementary Figure s1/Identification from LuMet-CM datebase/png/compound00163.png]

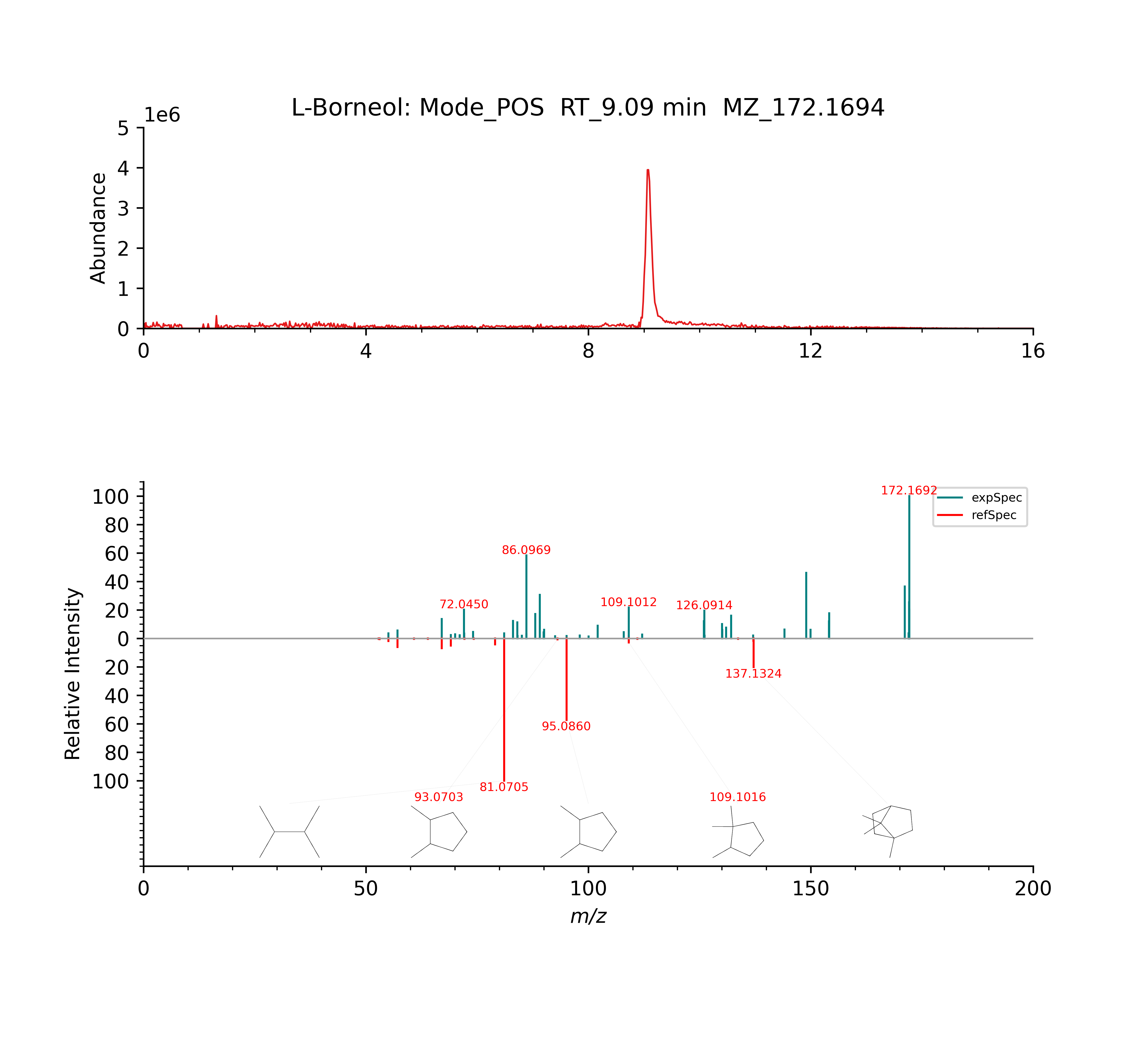

Supplement: Supplementary file 1 [file molecules-29-02840-s001.zip › Supplementary Figure s1/Identification from LuMet-CM datebase/png/compound00164.png]

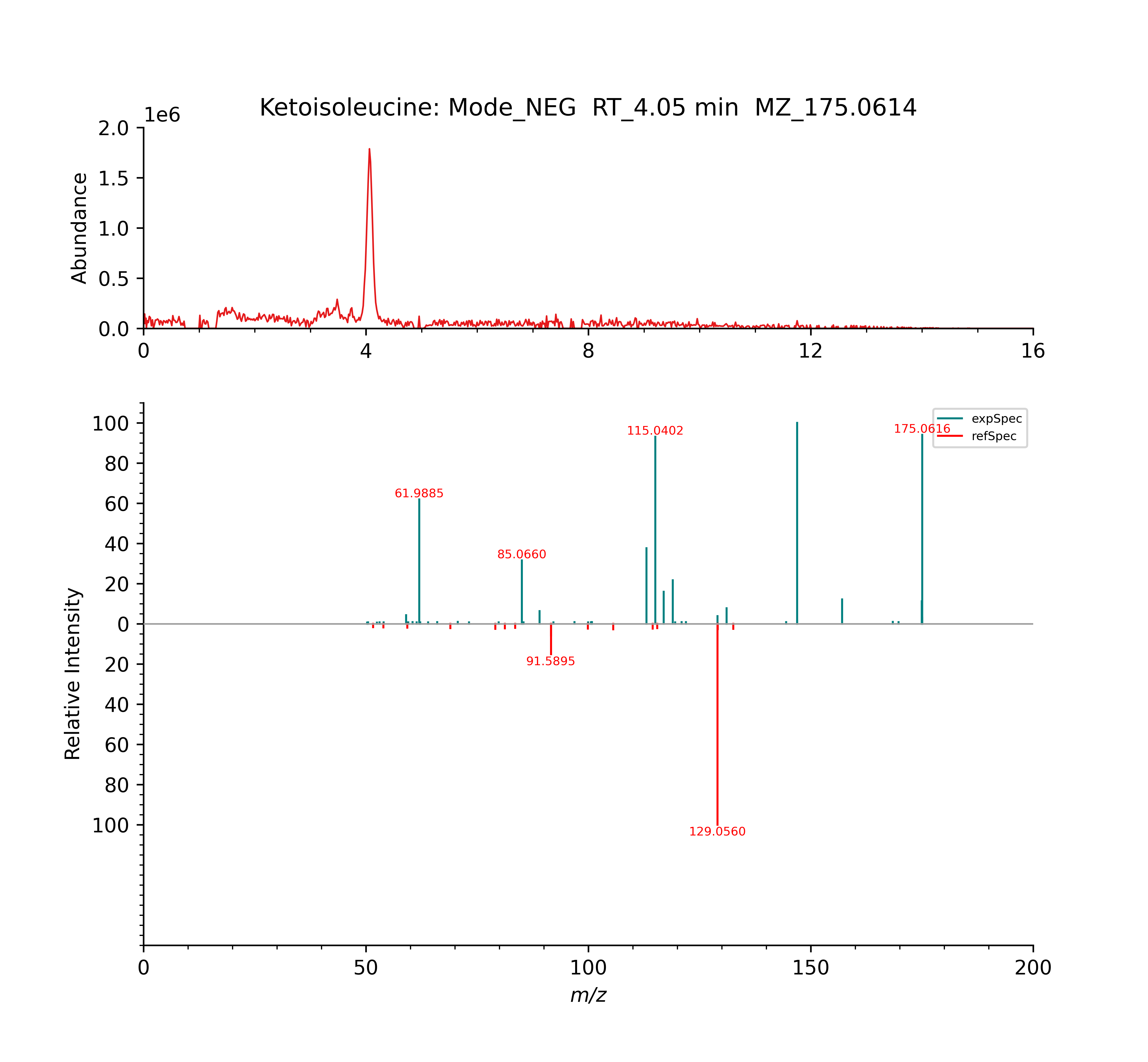

Supplement: Supplementary file 1 [file molecules-29-02840-s001.zip › Supplementary Figure s1/Identification from LuMet-CM datebase/png/compound00165.png]

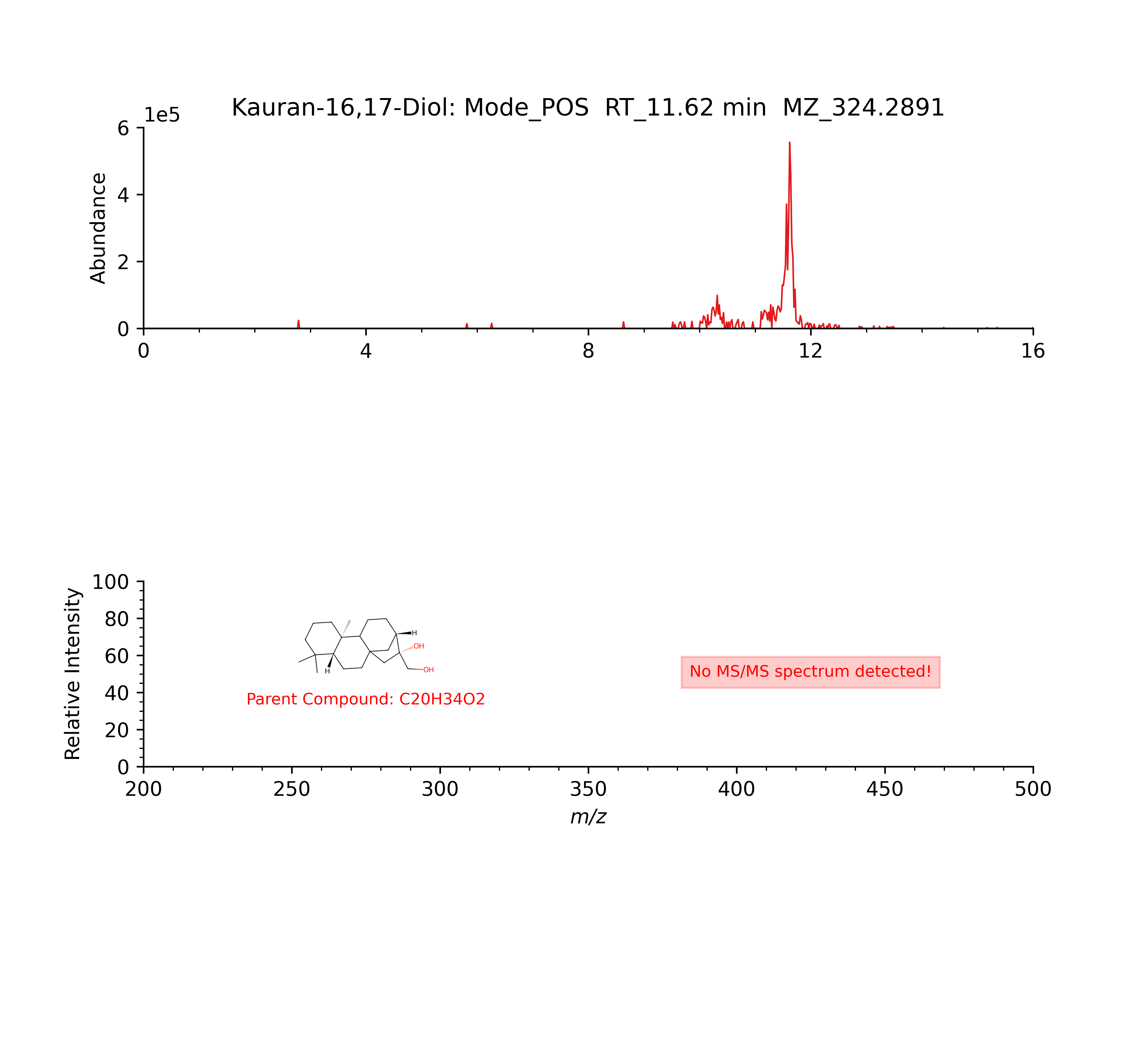

Supplement: Supplementary file 1 [file molecules-29-02840-s001.zip › Supplementary Figure s1/Identification from LuMet-CM datebase/png/compound00166.png]

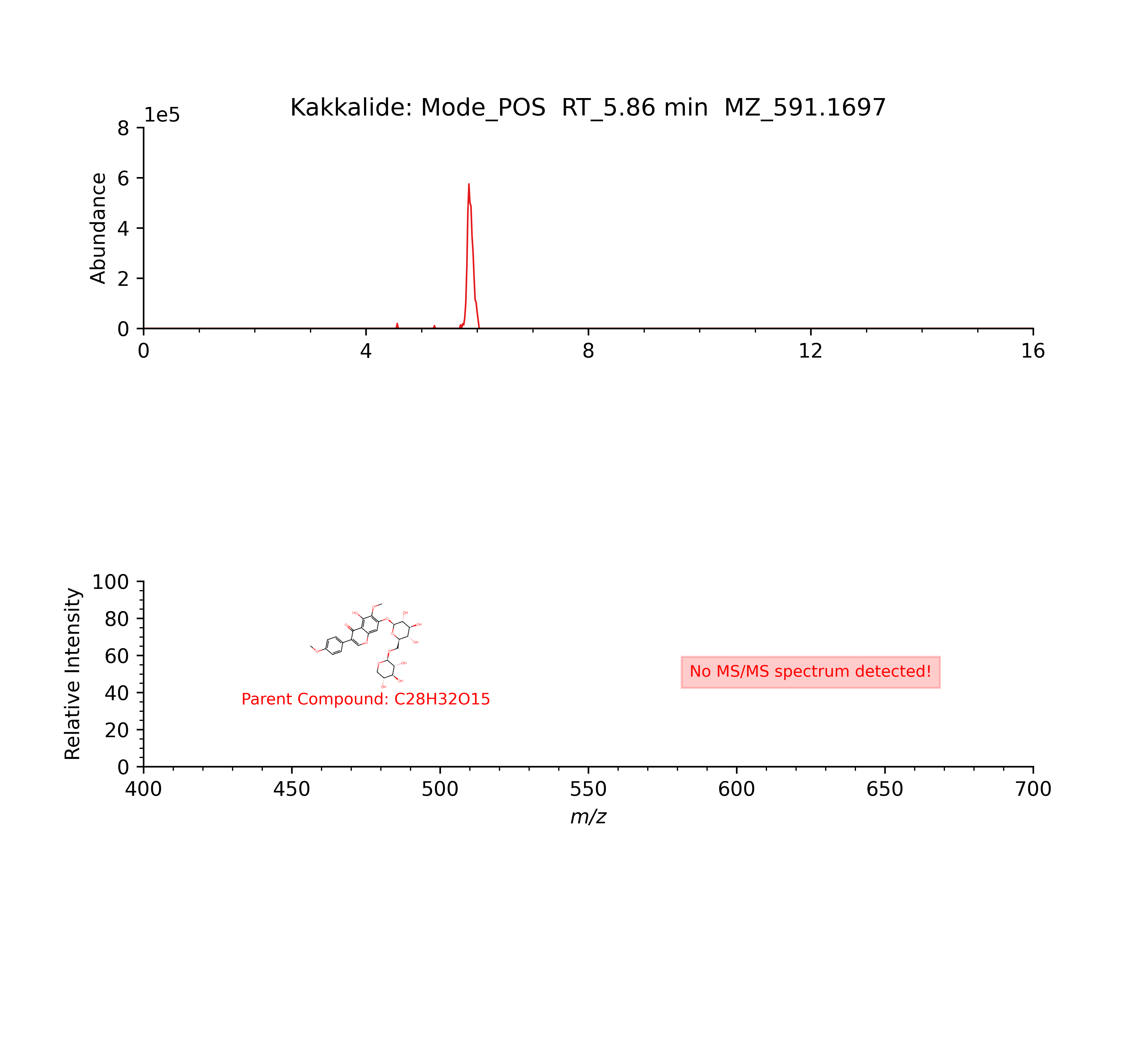

Supplement: Supplementary file 1 [file molecules-29-02840-s001.zip › Supplementary Figure s1/Identification from LuMet-CM datebase/png/compound00167.png]

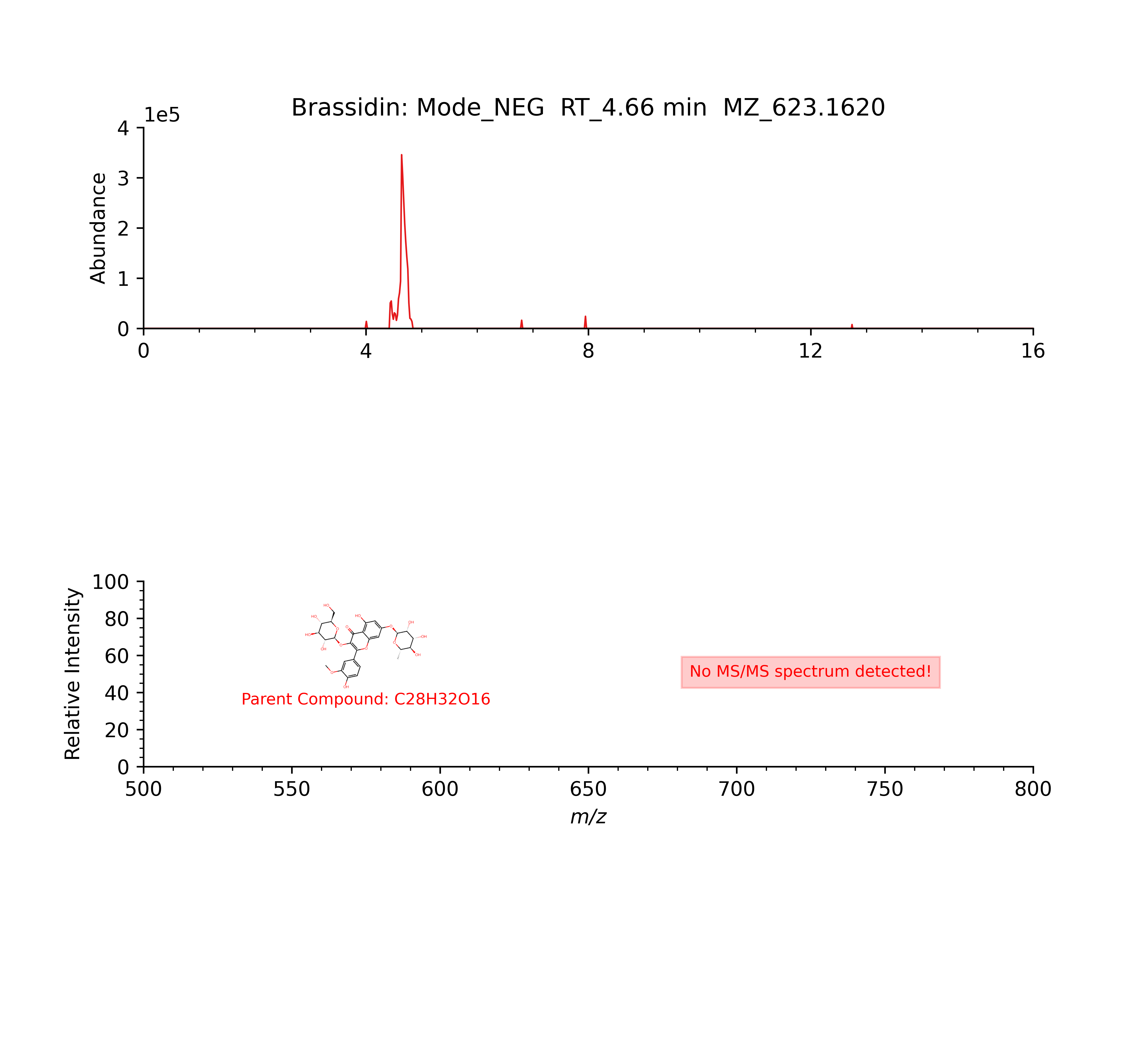

Supplement: Supplementary file 1 [file molecules-29-02840-s001.zip › Supplementary Figure s1/Identification from LuMet-CM datebase/png/compound00169.png]

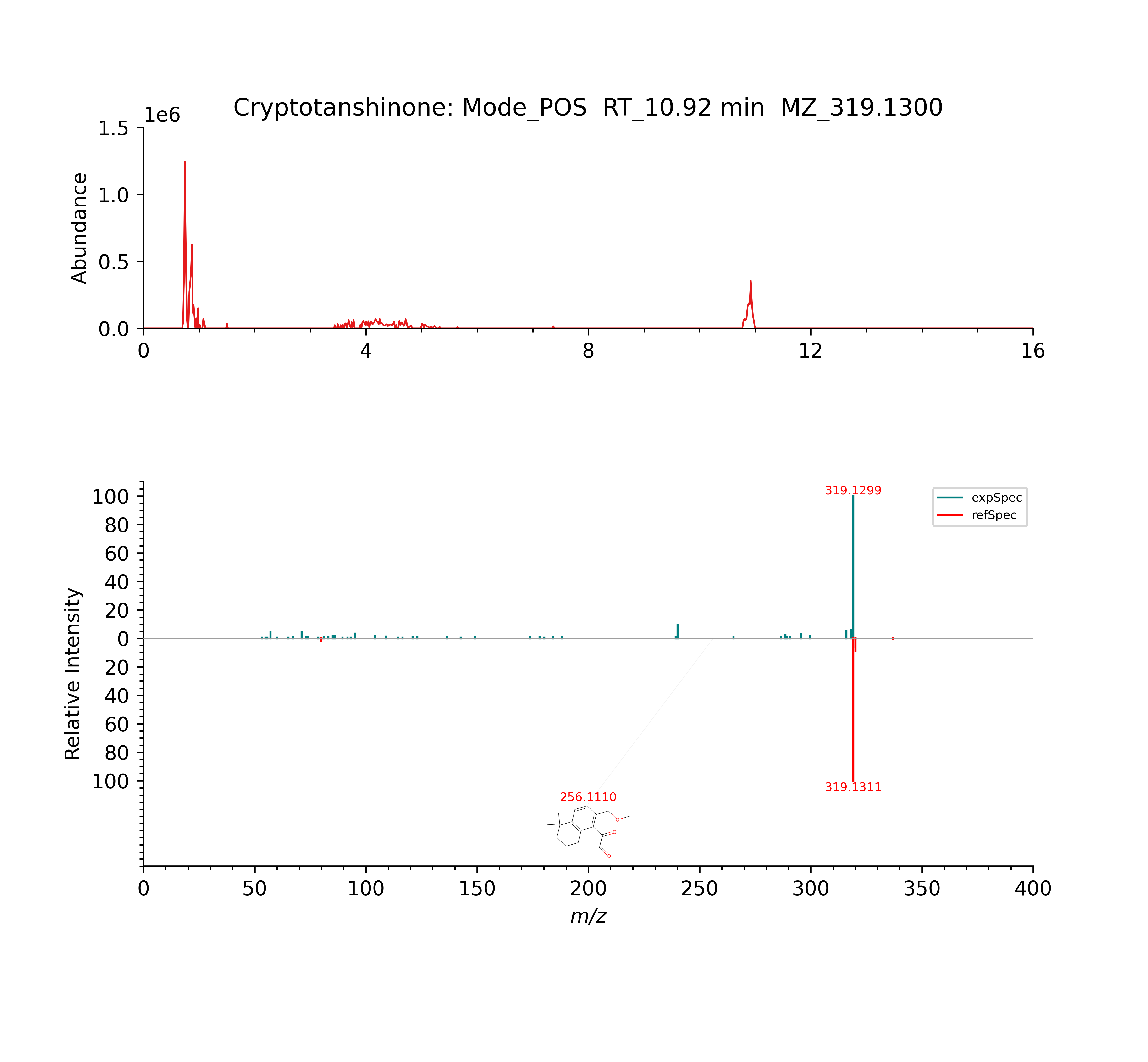

Supplement: Supplementary file 1 [file molecules-29-02840-s001.zip › Supplementary Figure s1/Identification from LuMet-CM datebase/png/compound00171.png]

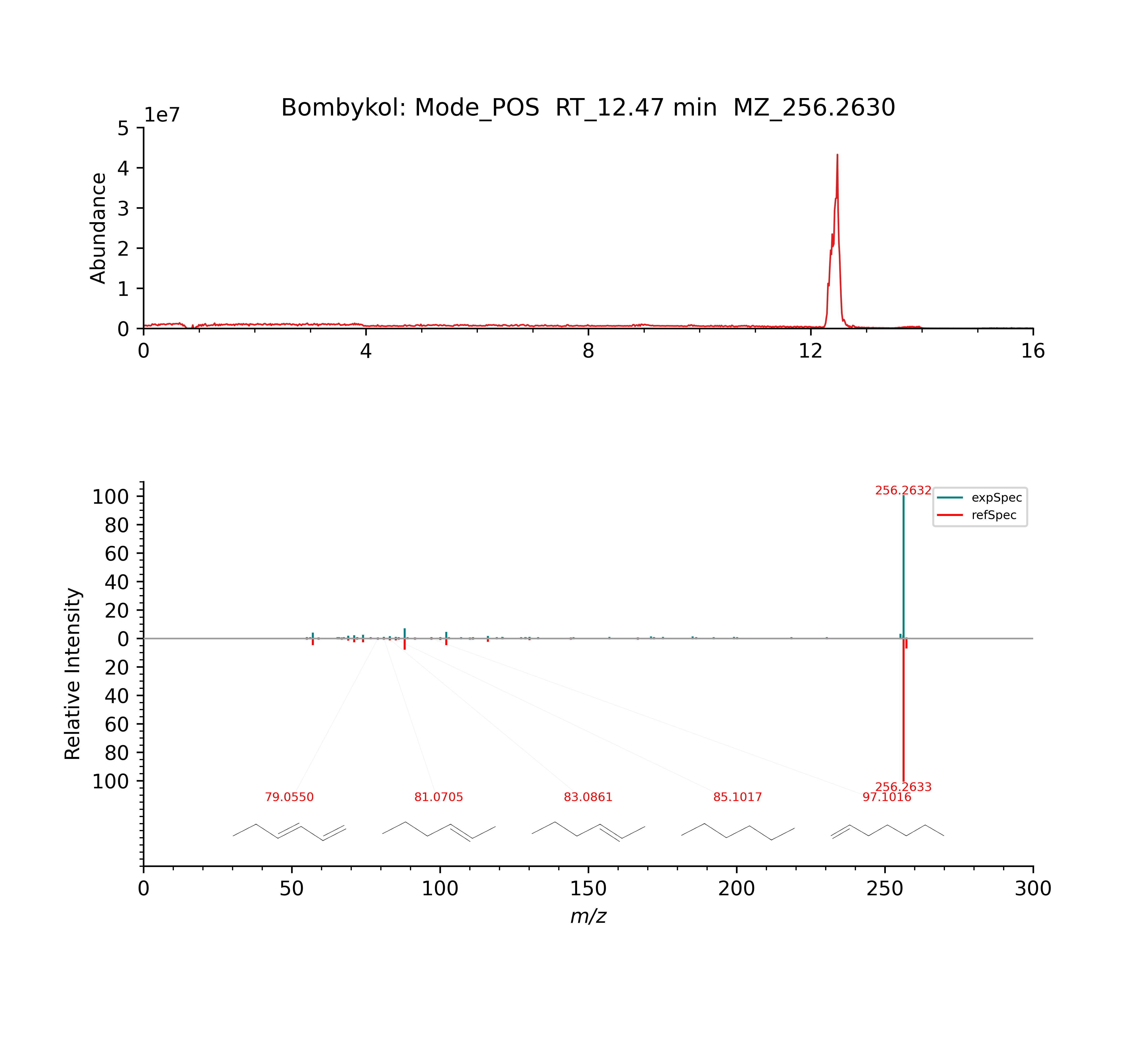

Supplement: Supplementary file 1 [file molecules-29-02840-s001.zip › Supplementary Figure s1/Identification from LuMet-CM datebase/png/compound00173.png]

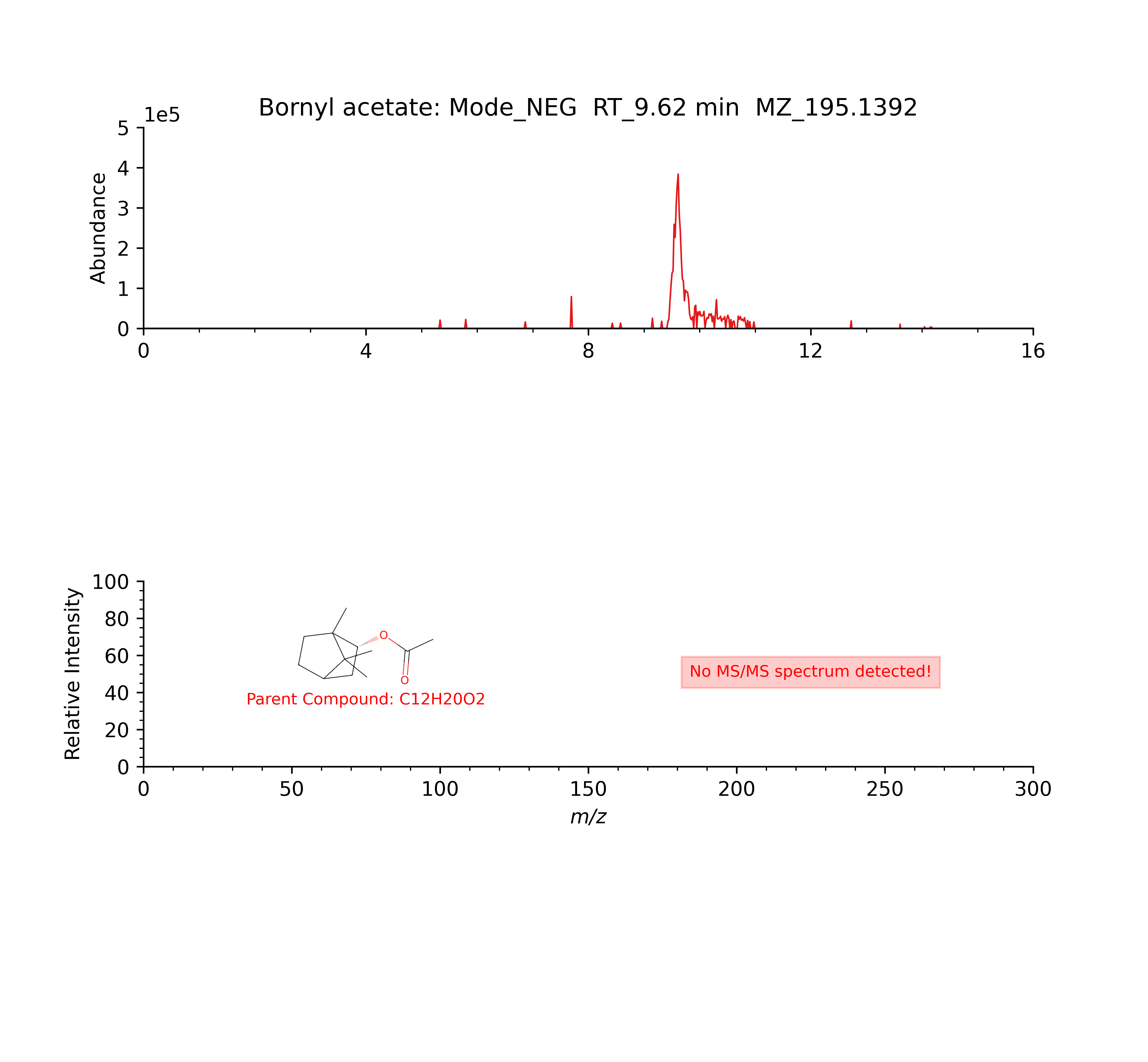

Supplement: Supplementary file 1 [file molecules-29-02840-s001.zip › Supplementary Figure s1/Identification from LuMet-CM datebase/png/compound00174.png]
