# Supplementary figures and images for: Characterization of the Components and Metabolites of Achyranthes Bidentata in the Plasma and Brain Tissue of Rats Based on Ultrahigh Performance Liquid Chromatography–High-Resolution Mass Spectrometry (UHPLC–HR-MS) (part 1 of 2)
Source: Molecules. 2024 Jun 14;29(12):2840. doi: 10.3390/molecules29122840 (PMC11206857; doi:10.3390/molecules29122840)

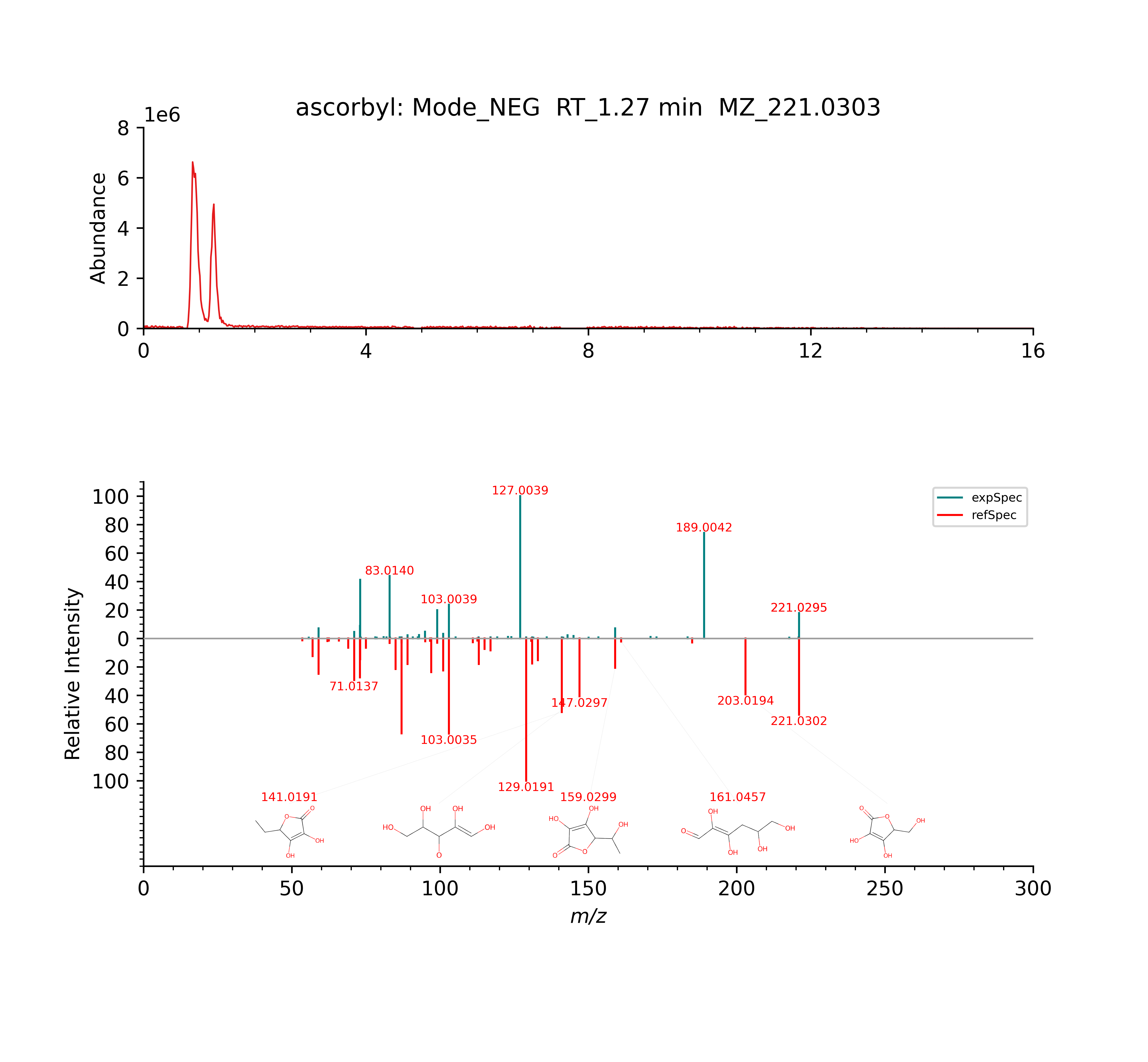

Supplement: Supplementary file 1 [file molecules-29-02840-s001.zip › Supplementary Figure s1/Identification from HerbDB datebase/png/compound00050.png]

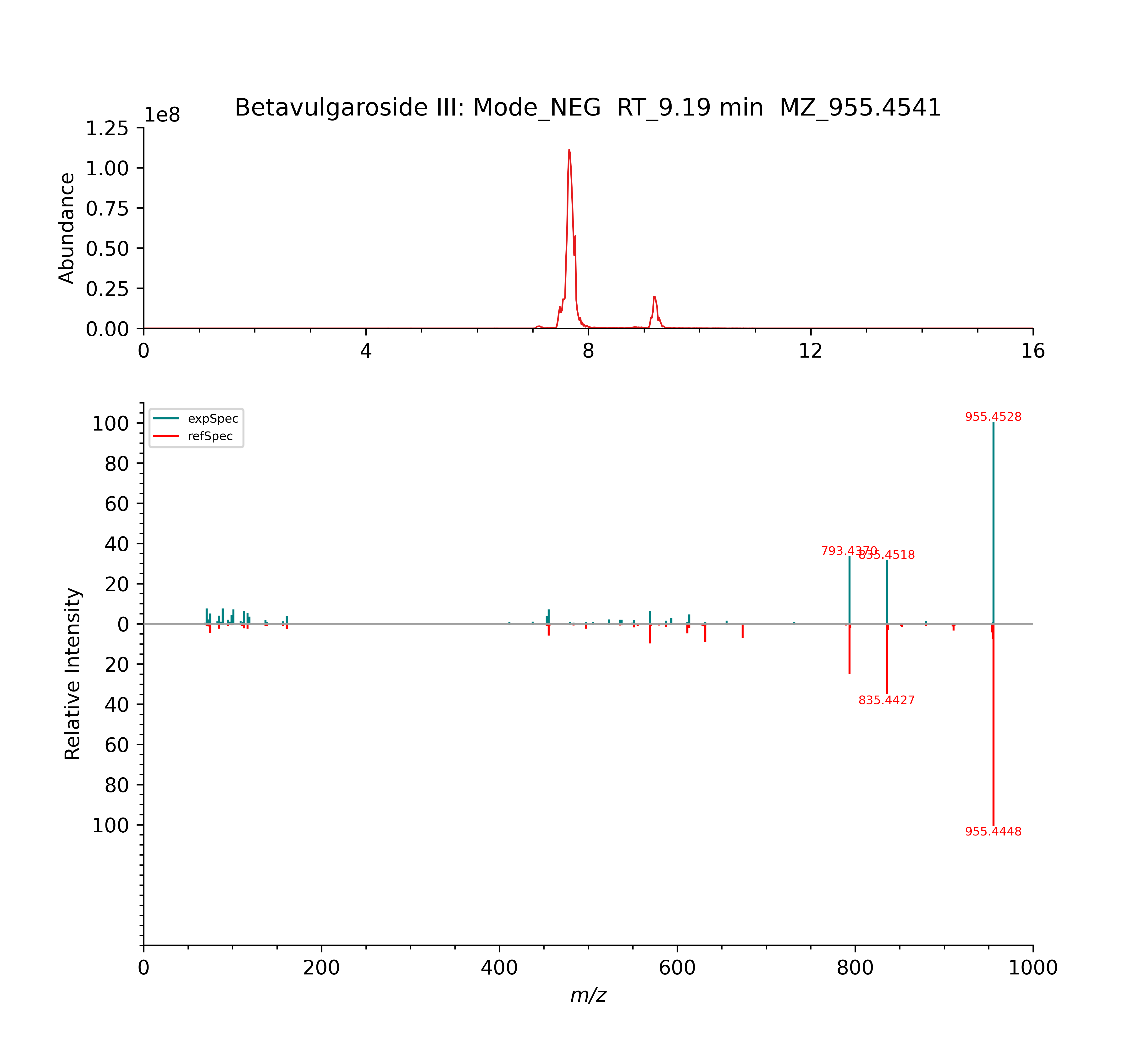

Supplement: Supplementary file 1 [file molecules-29-02840-s001.zip › Supplementary Figure s1/Identification from HerbDB datebase/png/compound00051.png]

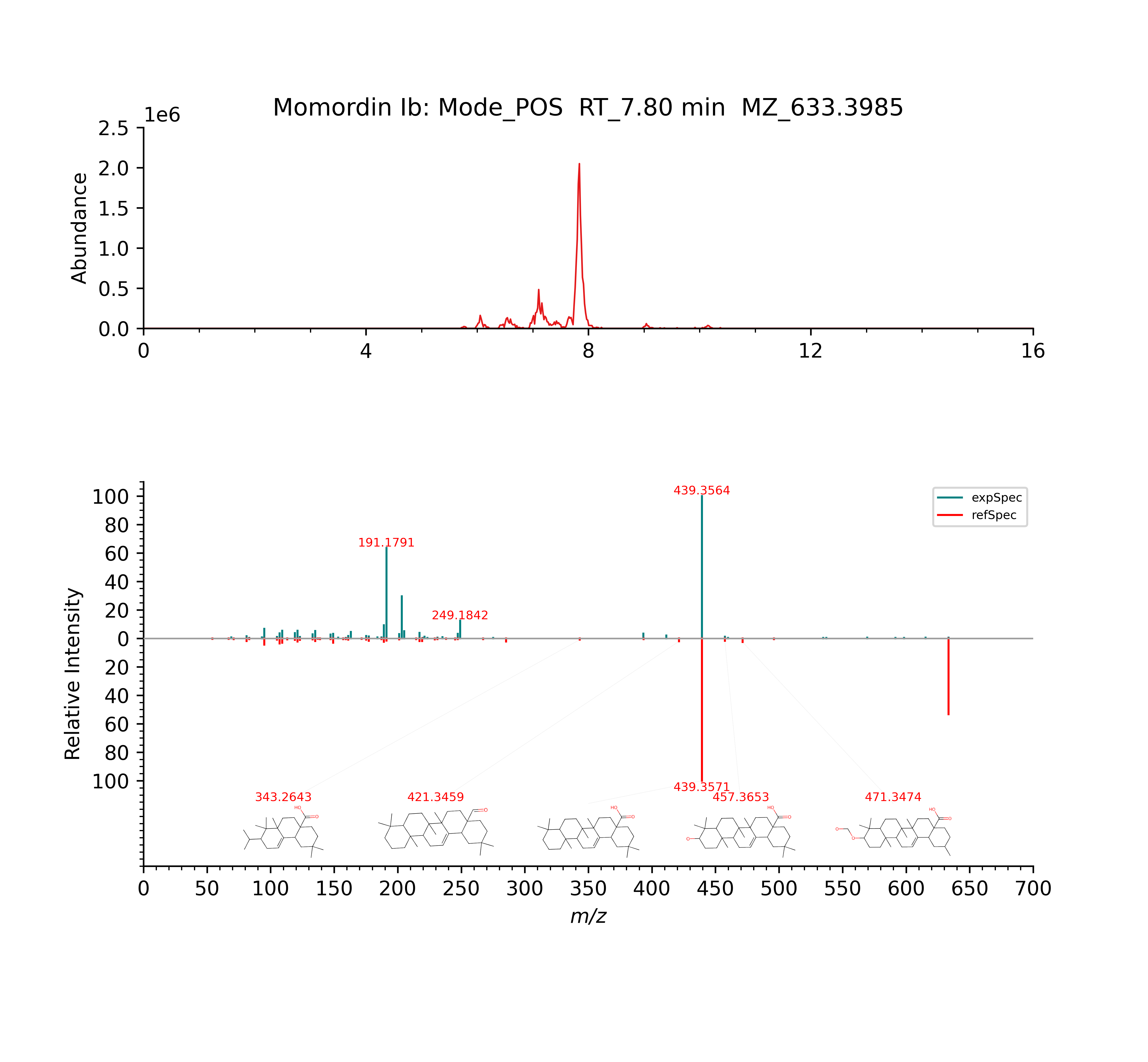

Supplement: Supplementary file 1 [file molecules-29-02840-s001.zip › Supplementary Figure s1/Identification from HerbDB datebase/png/compound00135.png]

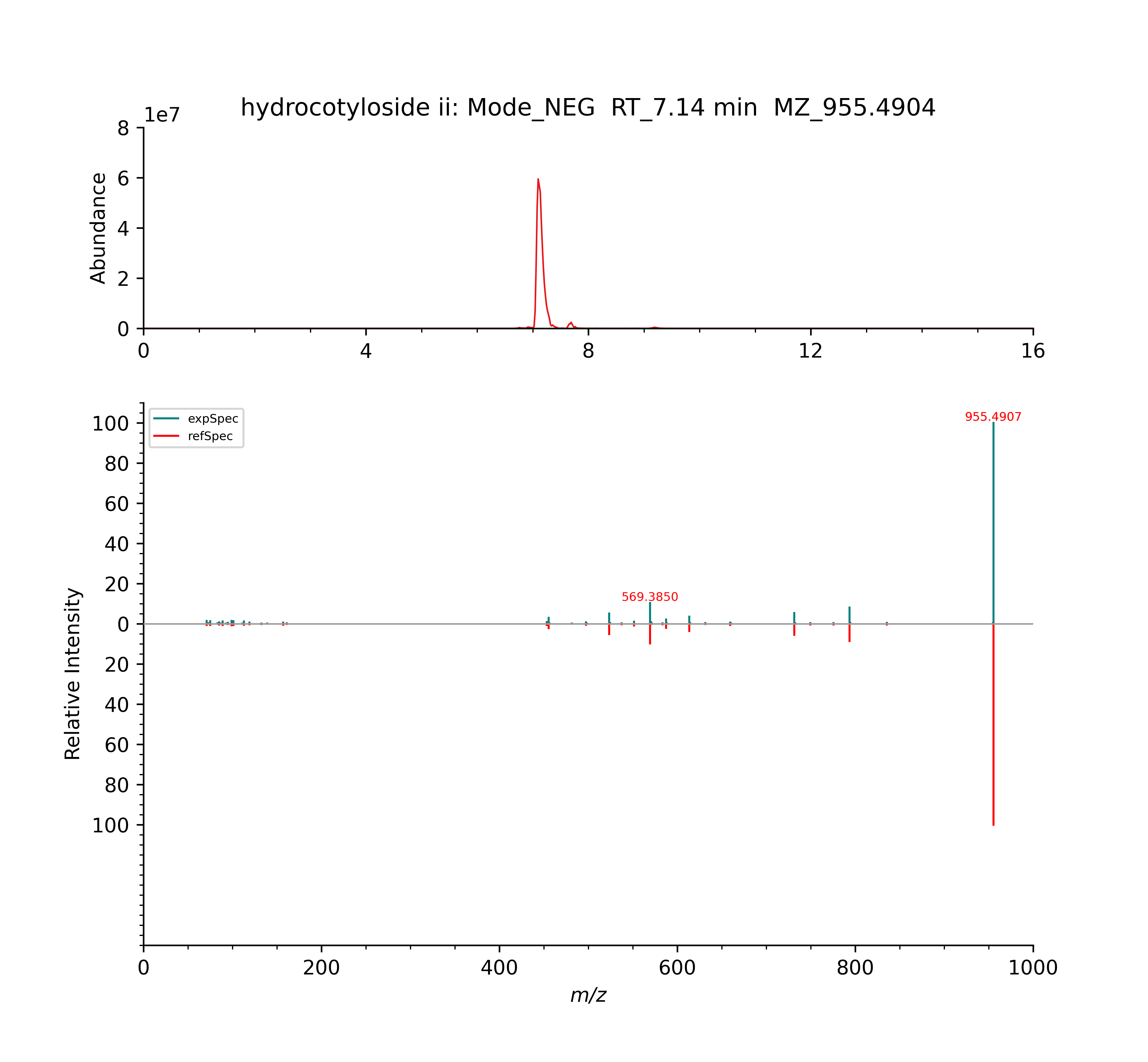

Supplement: Supplementary file 1 [file molecules-29-02840-s001.zip › Supplementary Figure s1/Identification from HerbDB datebase/png/compound00136.png]

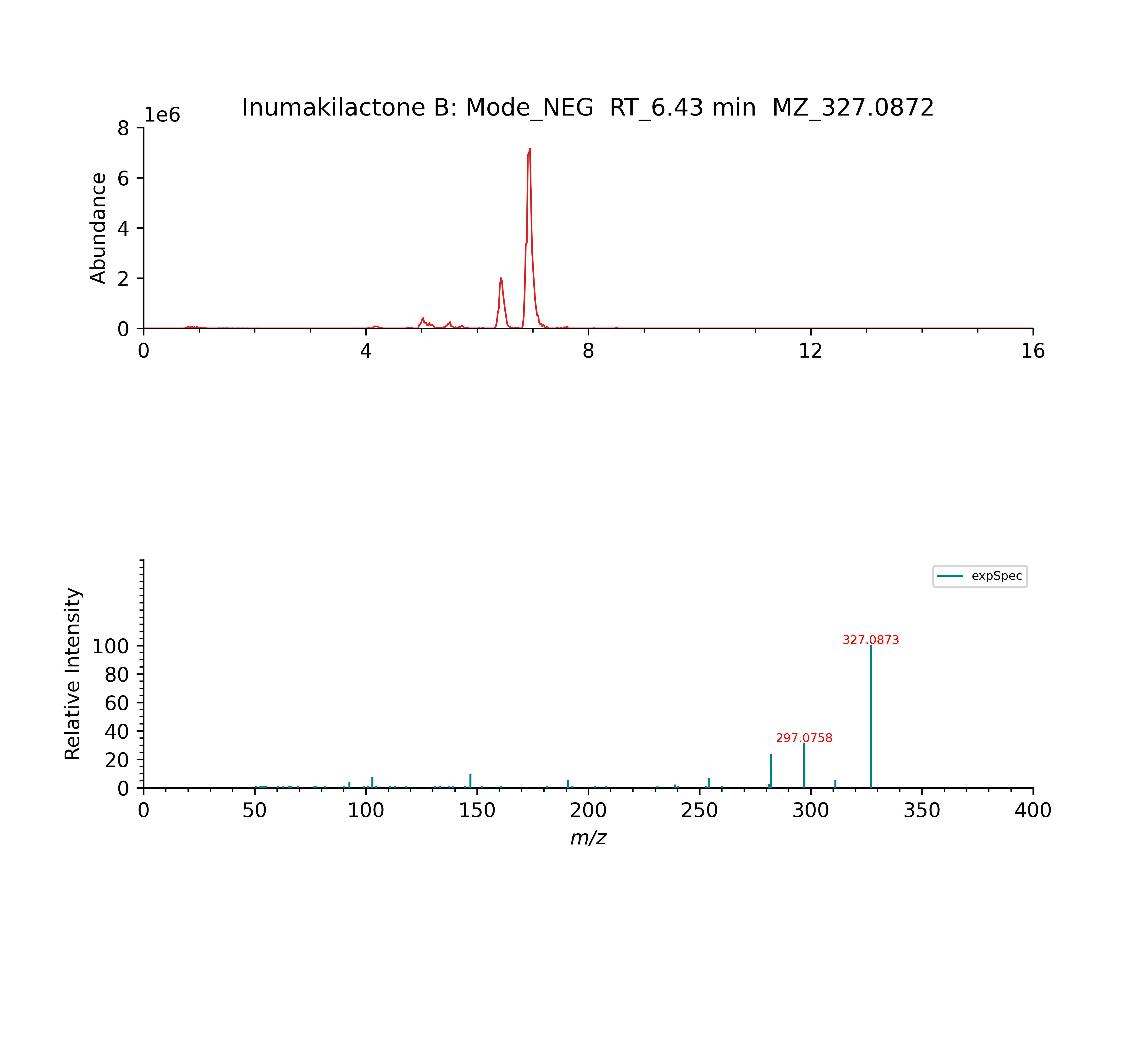

Supplement: Supplementary file 1 [file molecules-29-02840-s001.zip › Supplementary Figure s1/Identification from HerbDB datebase/png/compound00139.png]

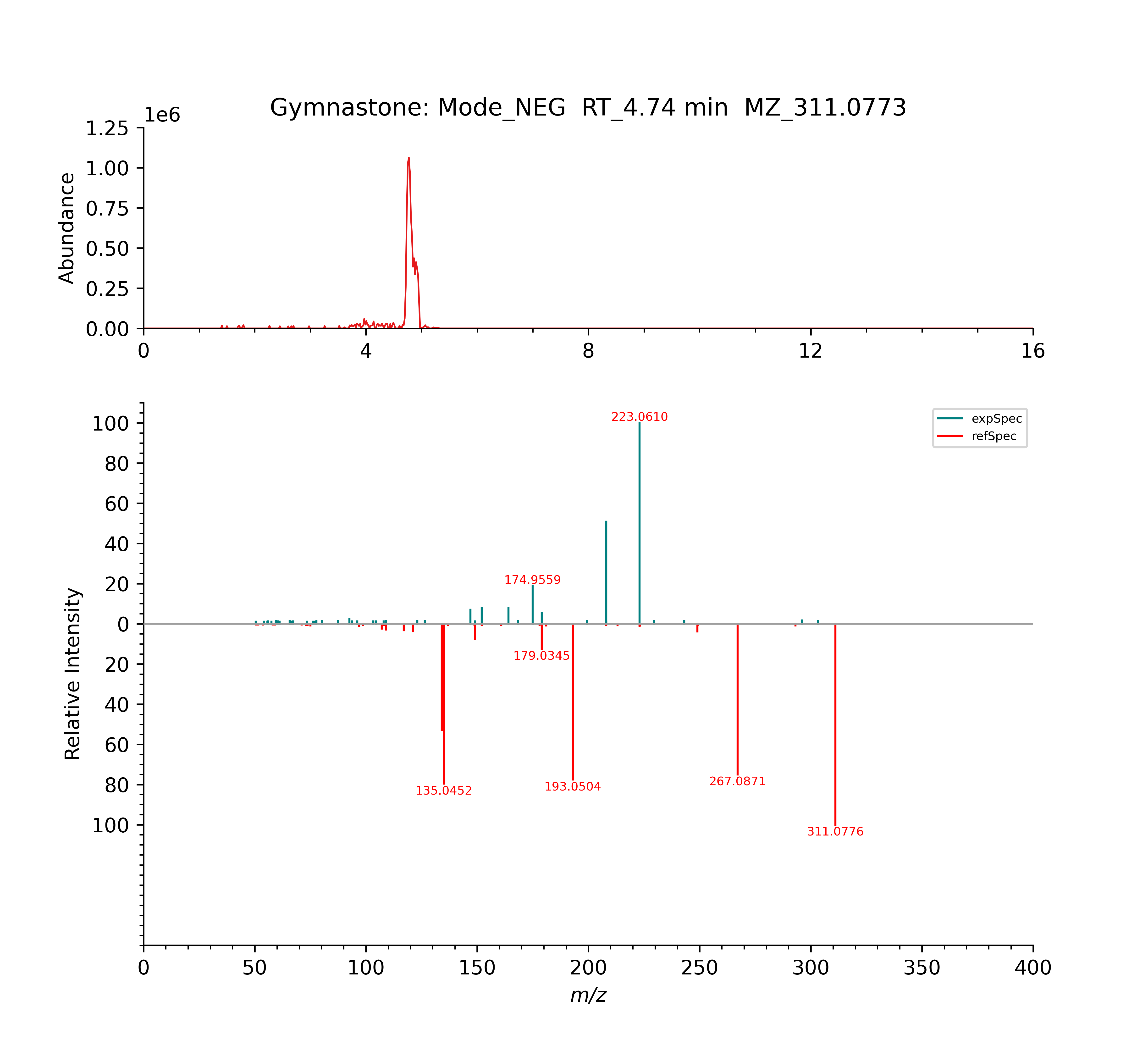

Supplement: Supplementary file 1 [file molecules-29-02840-s001.zip › Supplementary Figure s1/Identification from HerbDB datebase/png/compound00140.png]

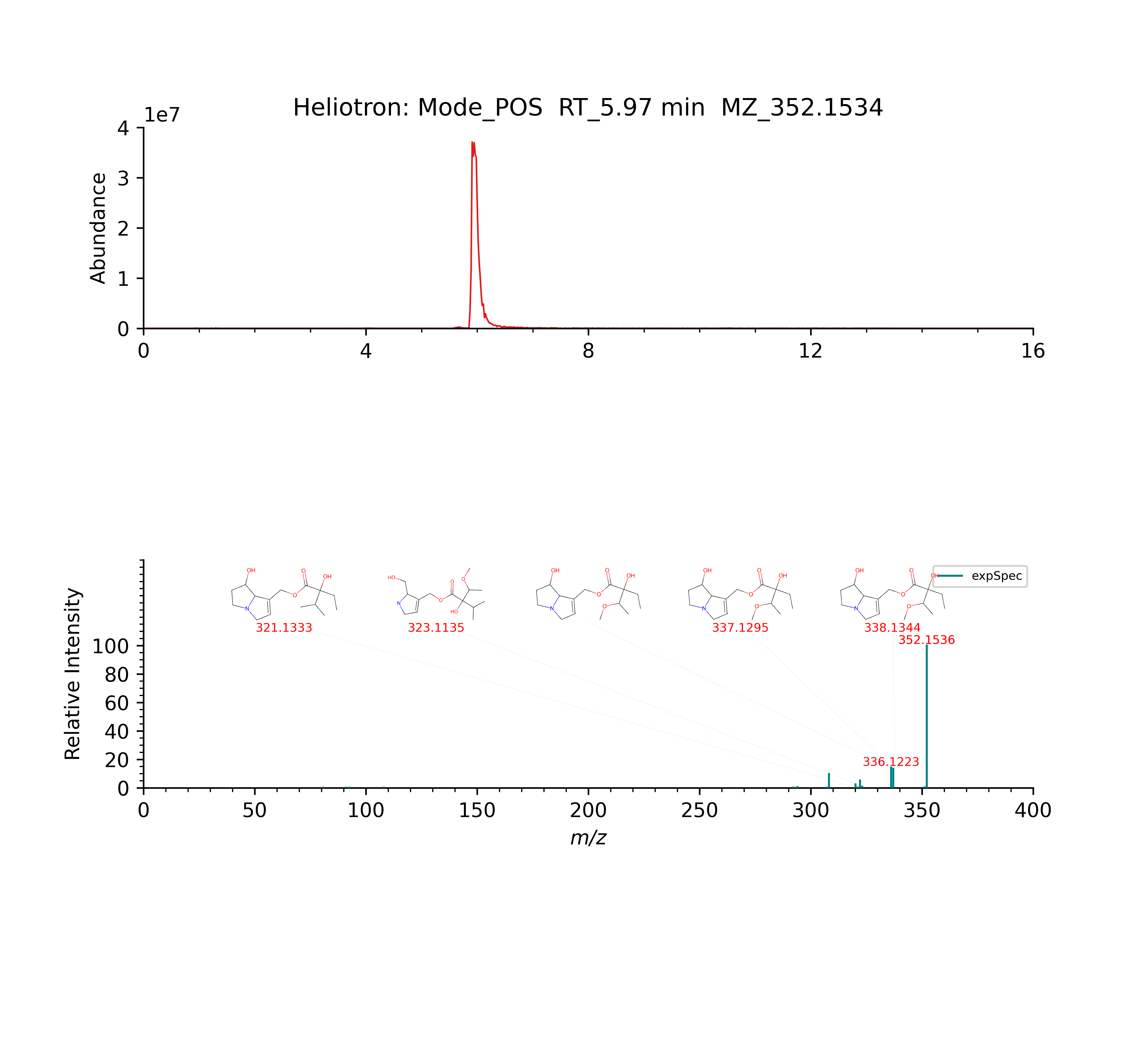

Supplement: Supplementary file 1 [file molecules-29-02840-s001.zip › Supplementary Figure s1/Identification from HerbDB datebase/png/compound00142.png]

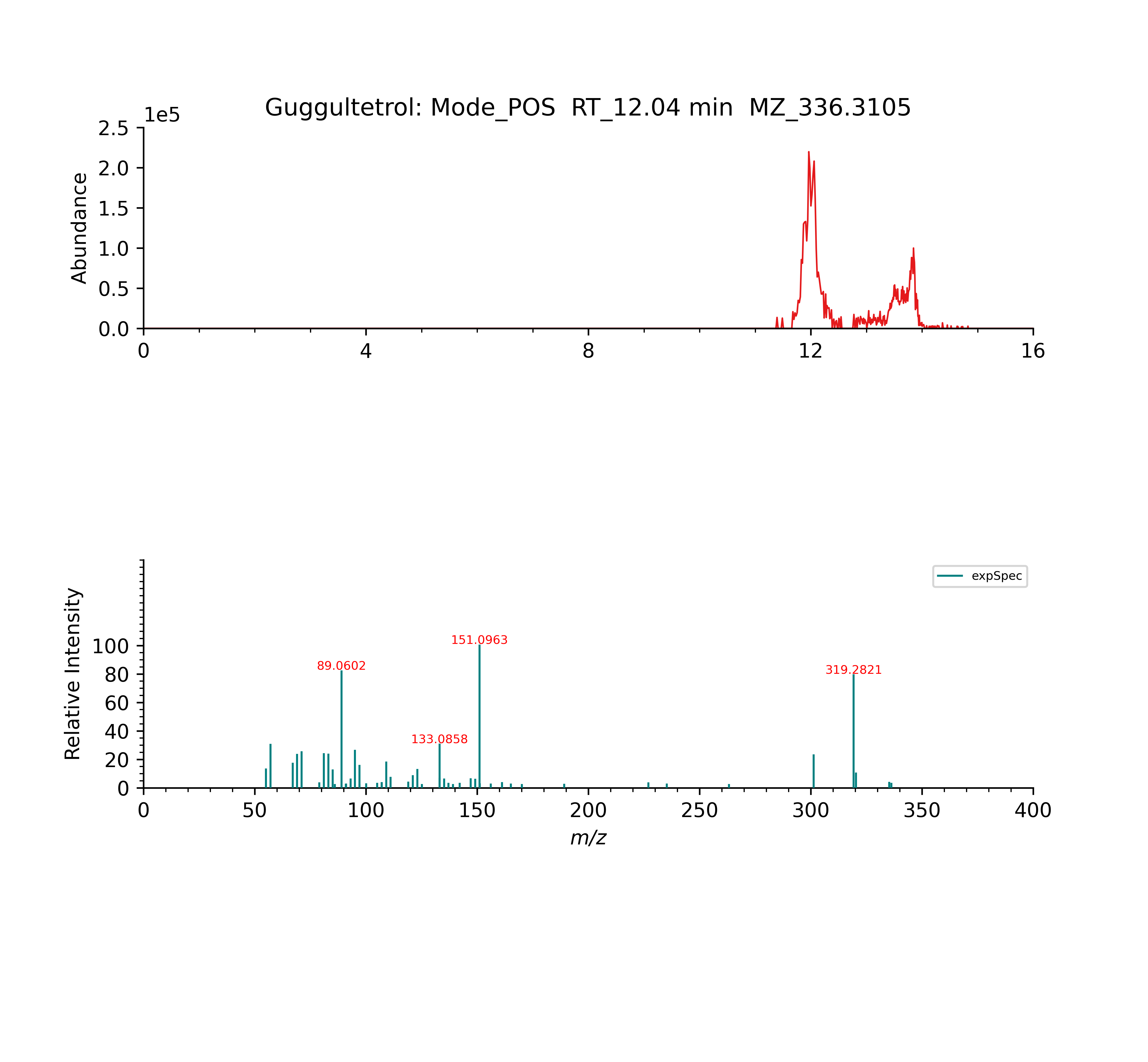

Supplement: Supplementary file 1 [file molecules-29-02840-s001.zip › Supplementary Figure s1/Identification from HerbDB datebase/png/compound00145.png]

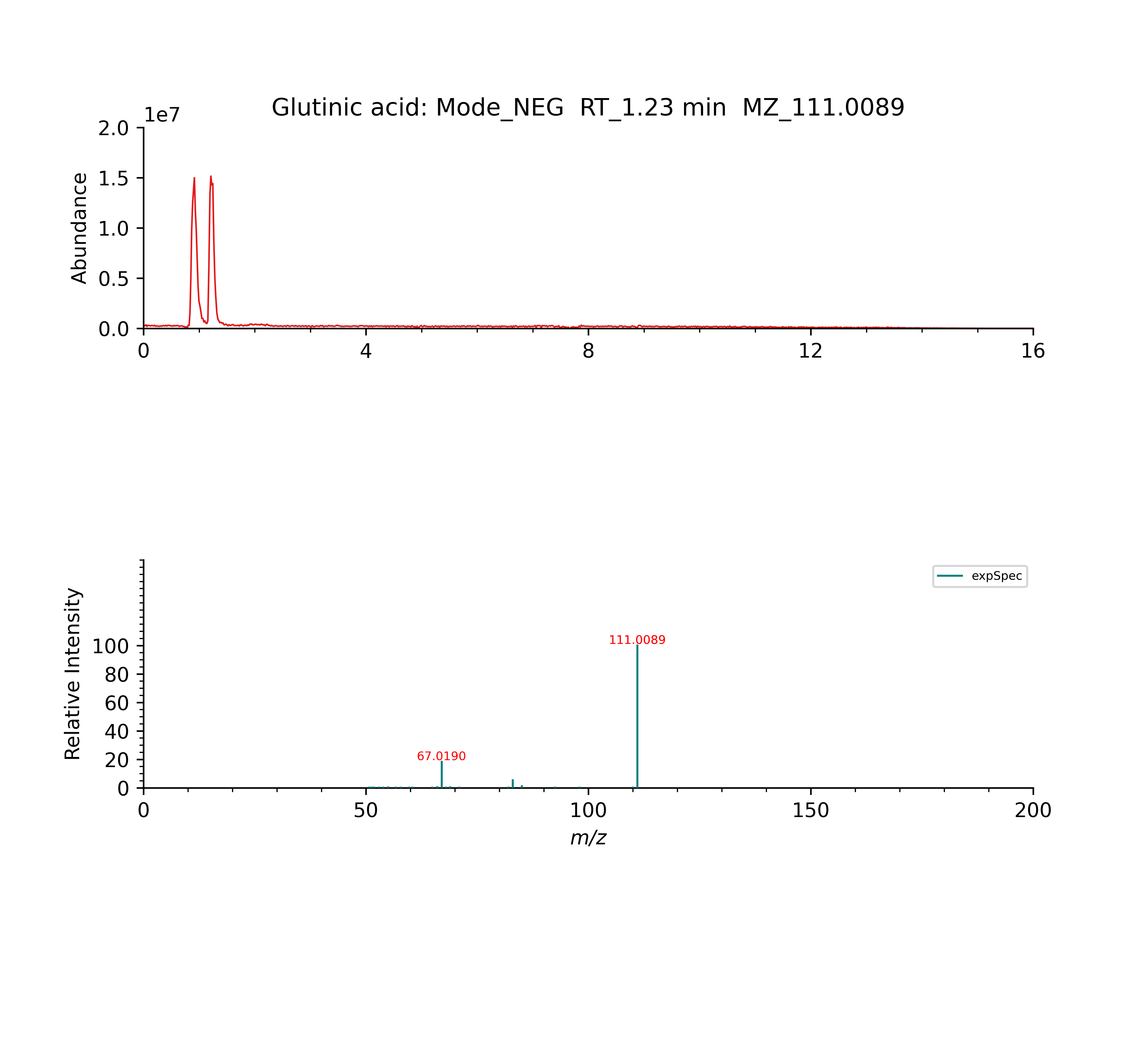

Supplement: Supplementary file 1 [file molecules-29-02840-s001.zip › Supplementary Figure s1/Identification from HerbDB datebase/png/compound00147.png]

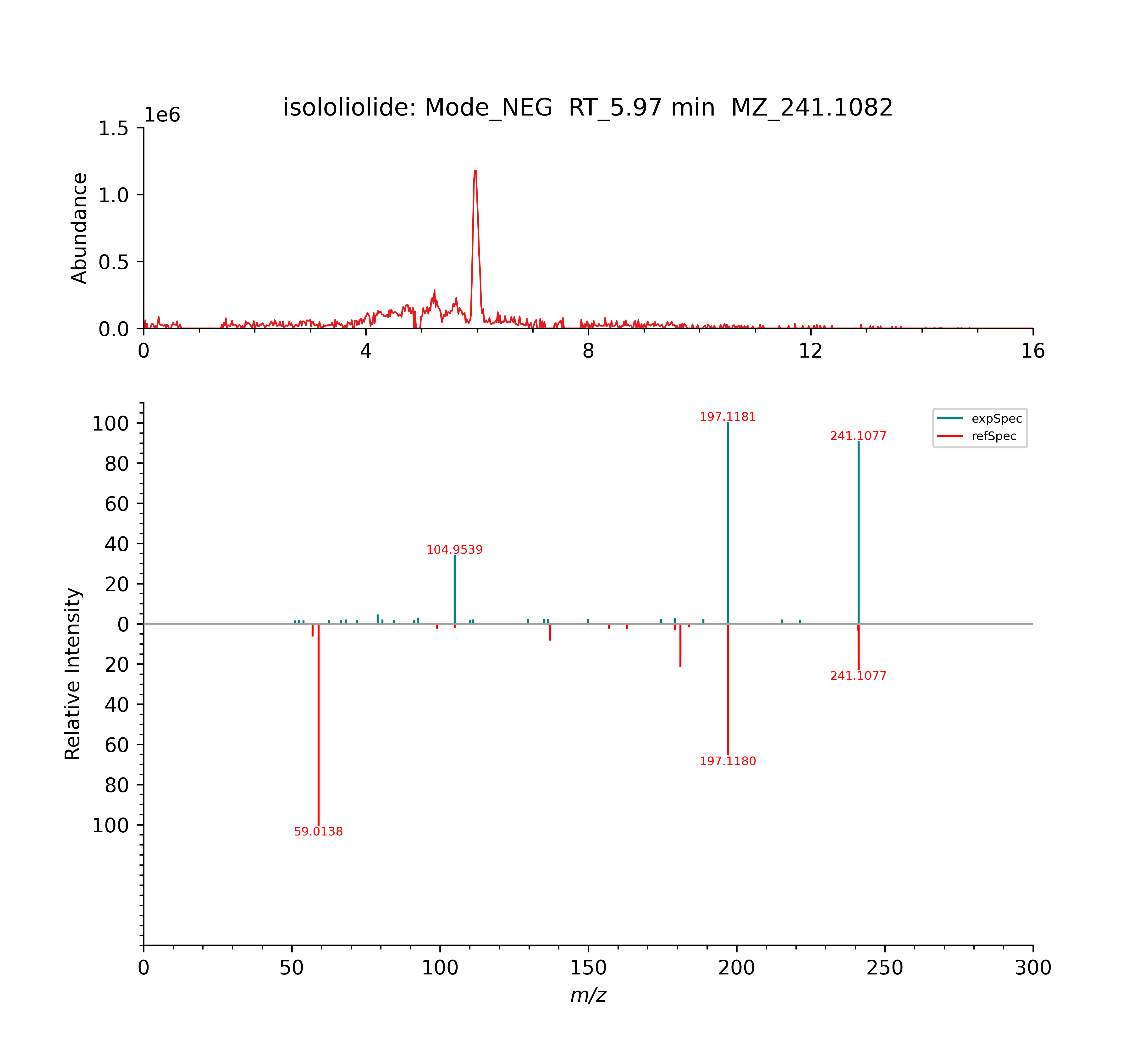

Supplement: Supplementary file 1 [file molecules-29-02840-s001.zip › Supplementary Figure s1/Identification from HerbDB datebase/png/compound00149.png]

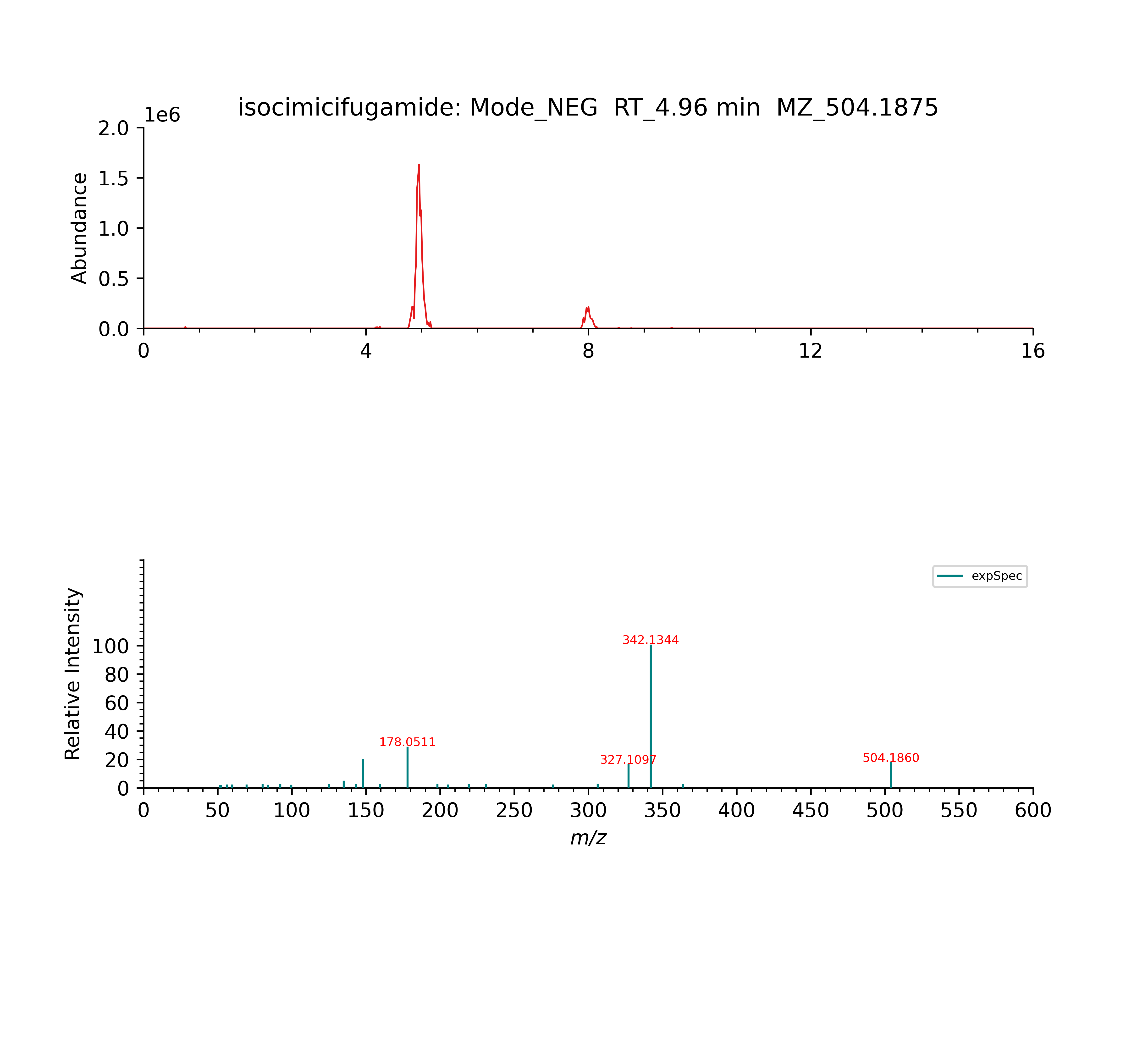

Supplement: Supplementary file 1 [file molecules-29-02840-s001.zip › Supplementary Figure s1/Identification from HerbDB datebase/png/compound00150.png]

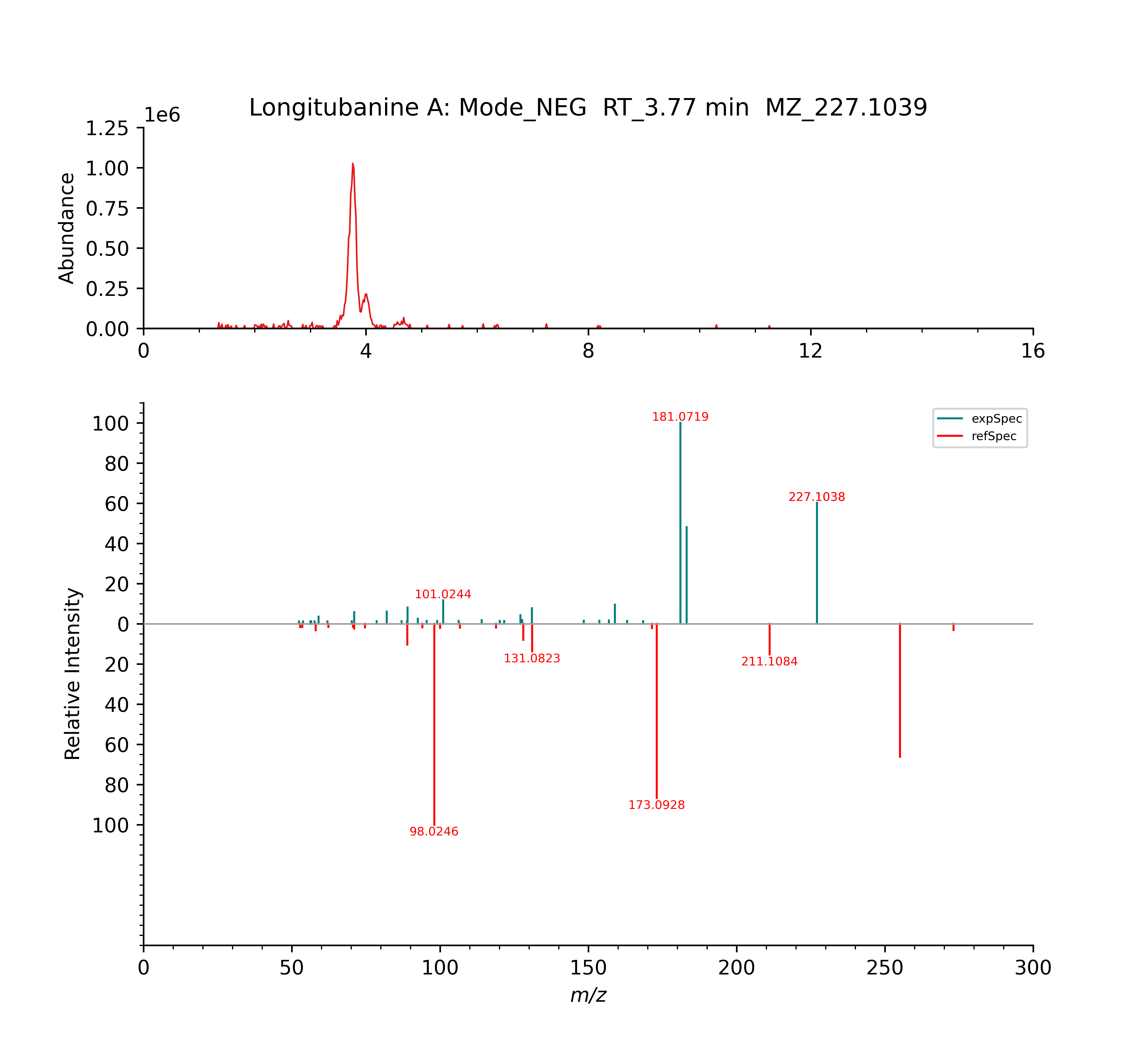

Supplement: Supplementary file 1 [file molecules-29-02840-s001.zip › Supplementary Figure s1/Identification from HerbDB datebase/png/compound00153.png]

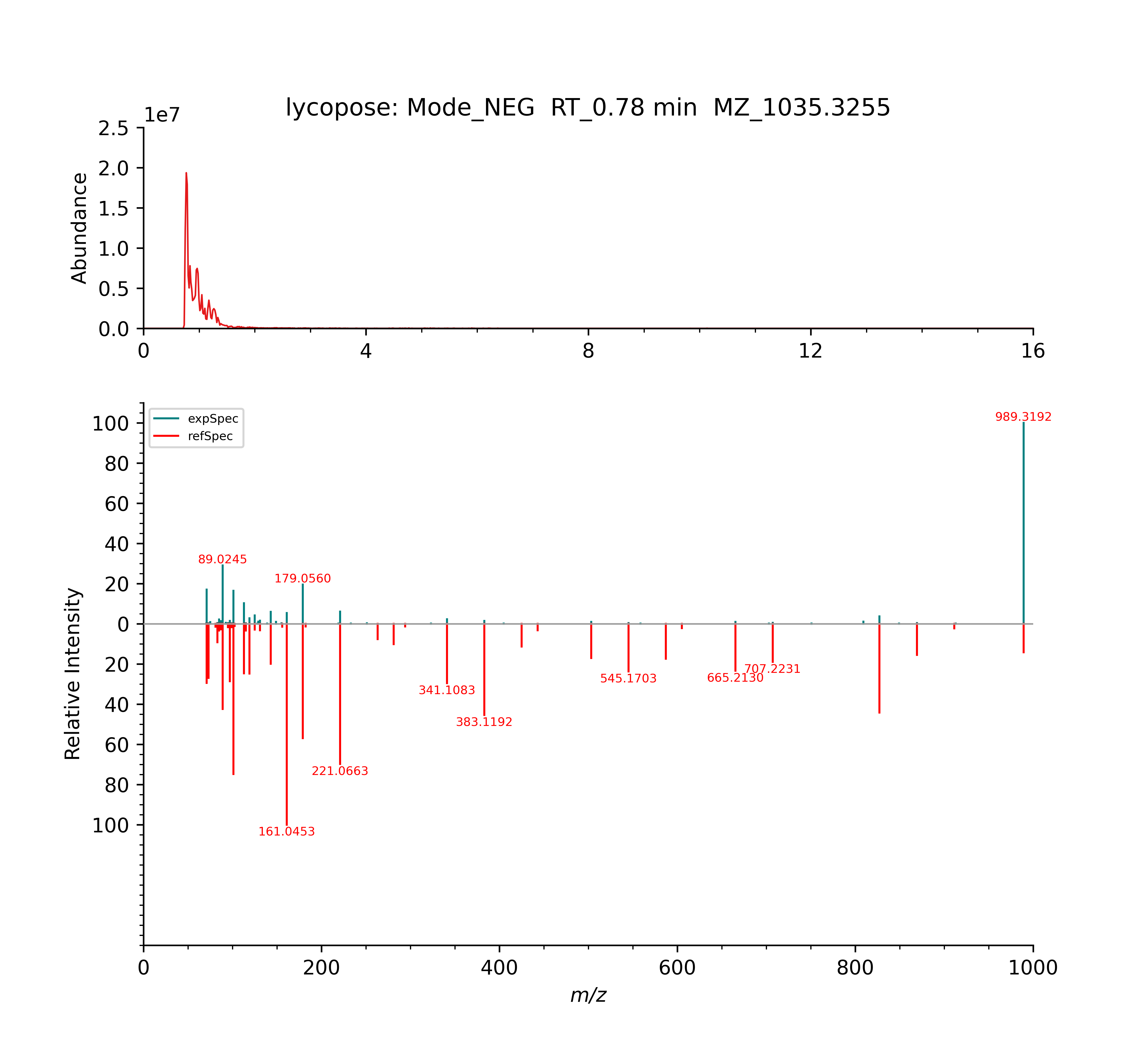

Supplement: Supplementary file 1 [file molecules-29-02840-s001.zip › Supplementary Figure s1/Identification from HerbDB datebase/png/compound00157.png]

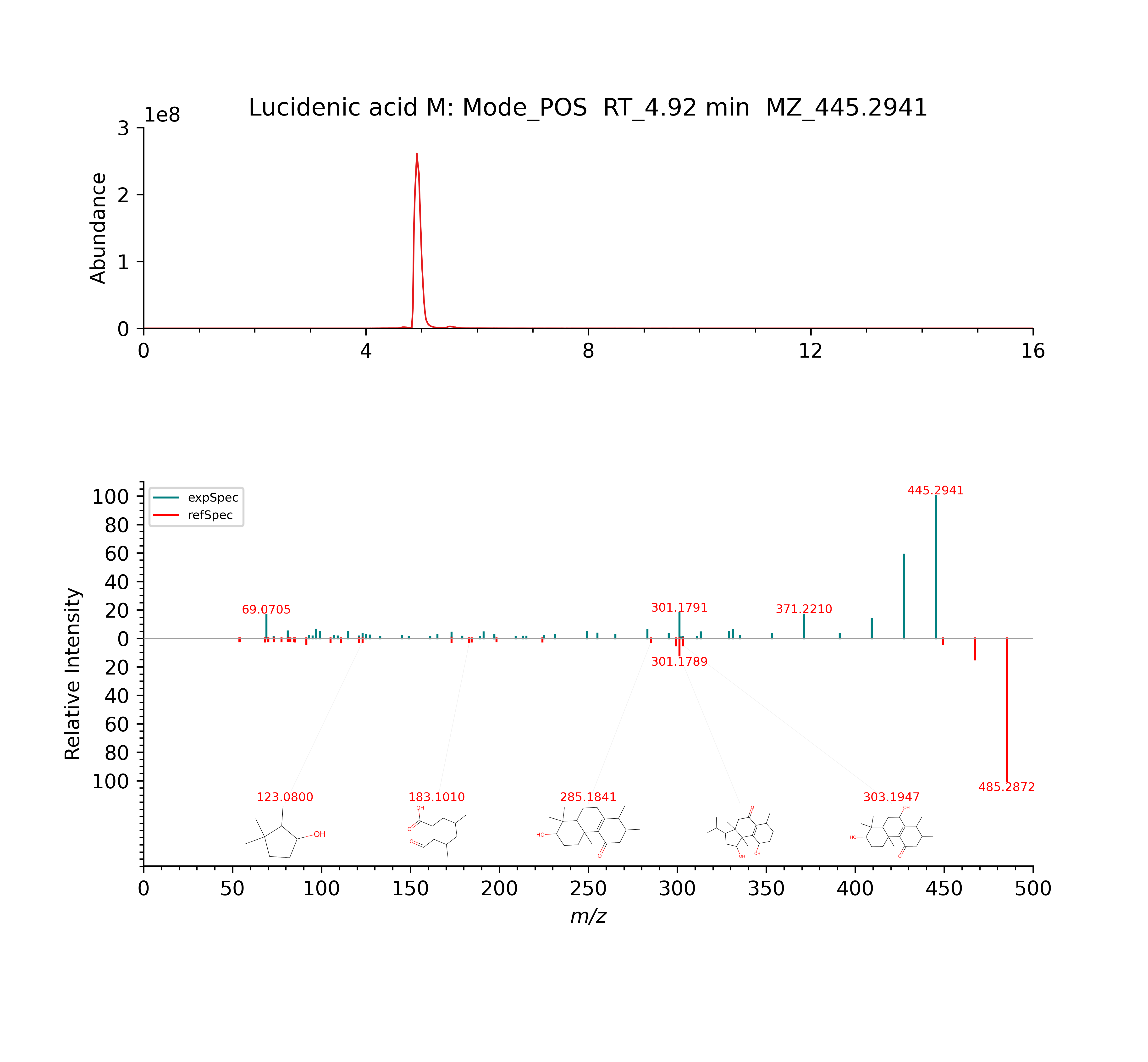

Supplement: Supplementary file 1 [file molecules-29-02840-s001.zip › Supplementary Figure s1/Identification from HerbDB datebase/png/compound00159.png]

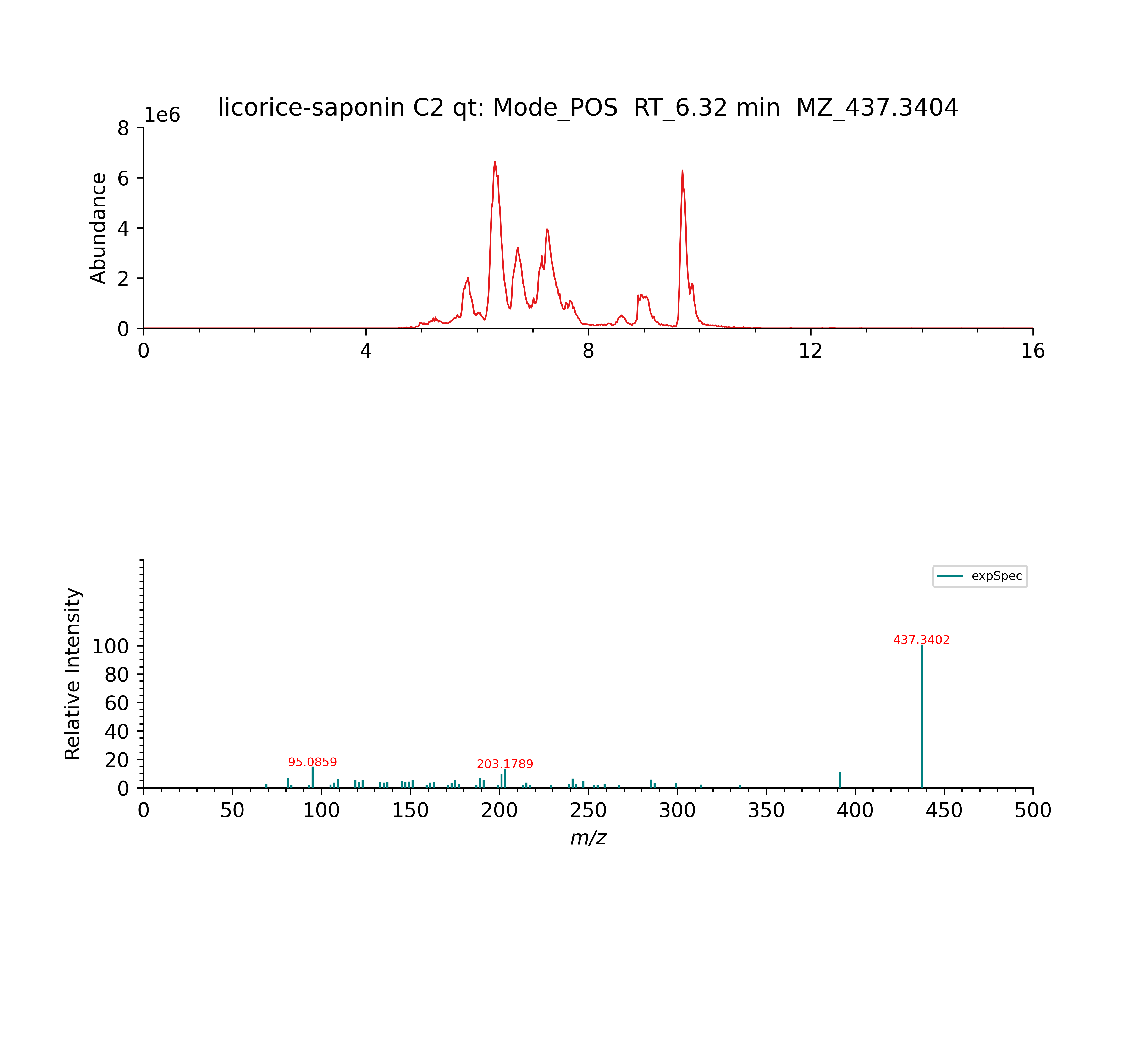

Supplement: Supplementary file 1 [file molecules-29-02840-s001.zip › Supplementary Figure s1/Identification from HerbDB datebase/png/compound00160.png]

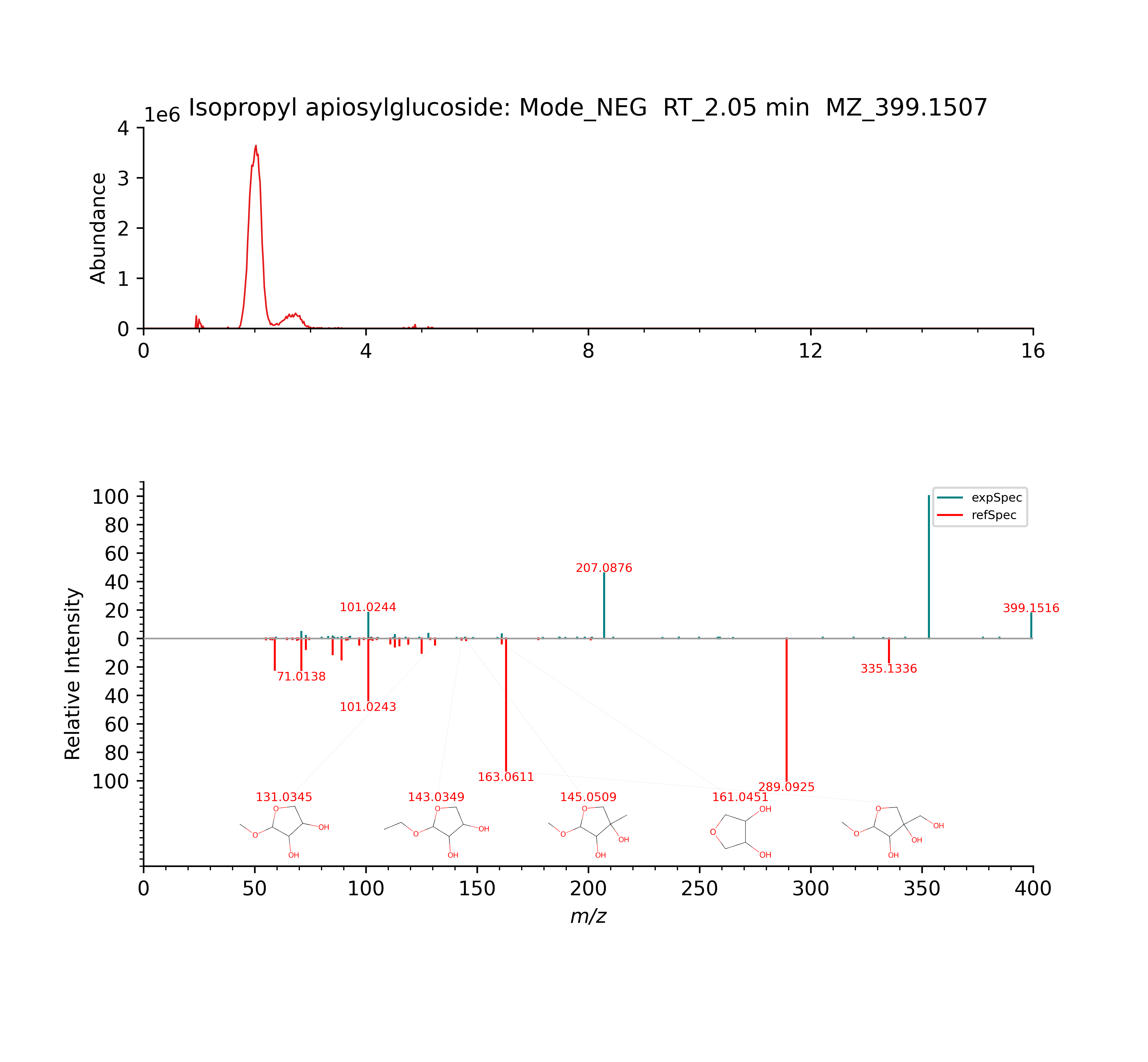

Supplement: Supplementary file 1 [file molecules-29-02840-s001.zip › Supplementary Figure s1/Identification from HerbDB datebase/png/compound00161.png]

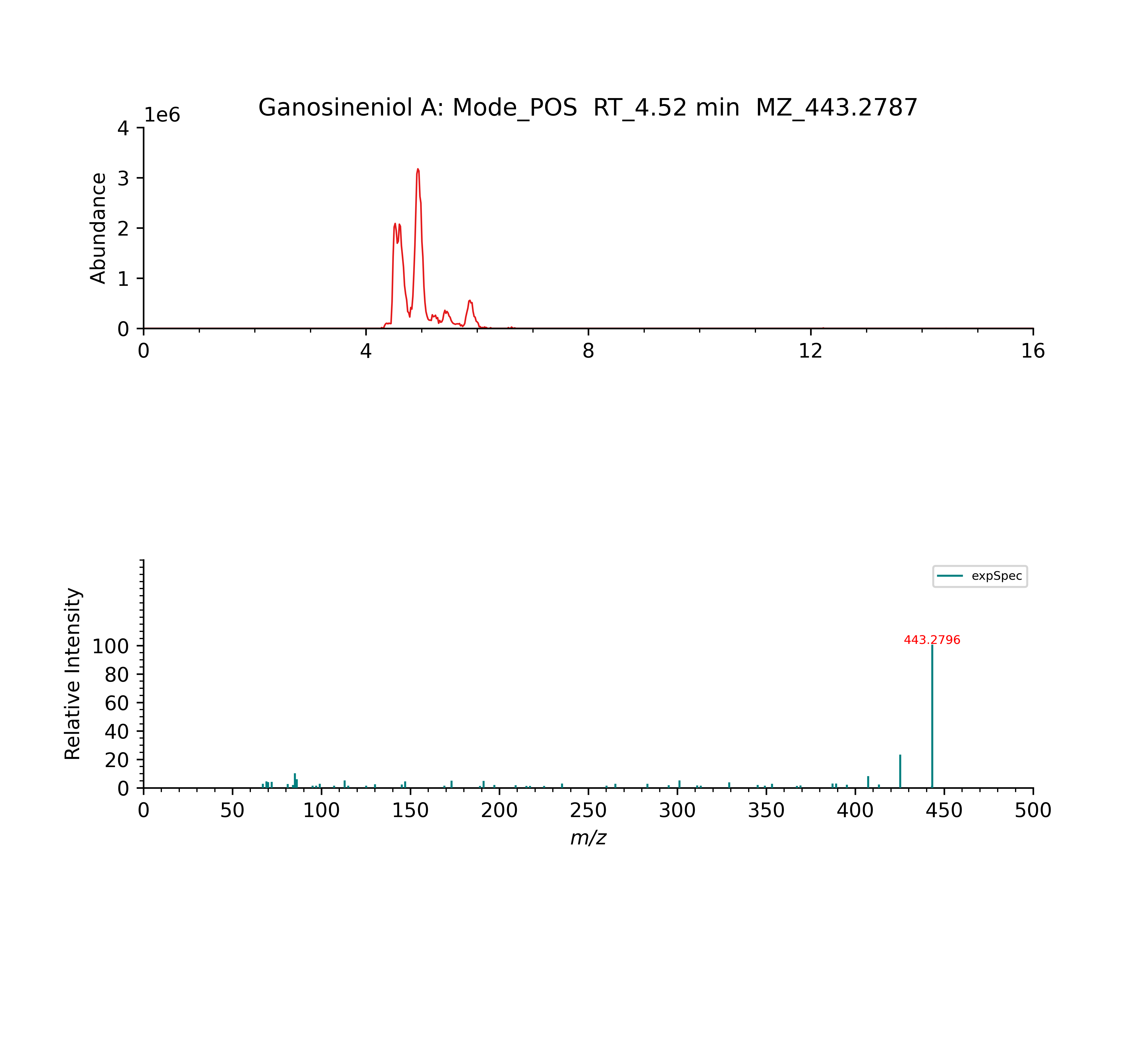

Supplement: Supplementary file 1 [file molecules-29-02840-s001.zip › Supplementary Figure s1/Identification from HerbDB datebase/png/compound00168.png]

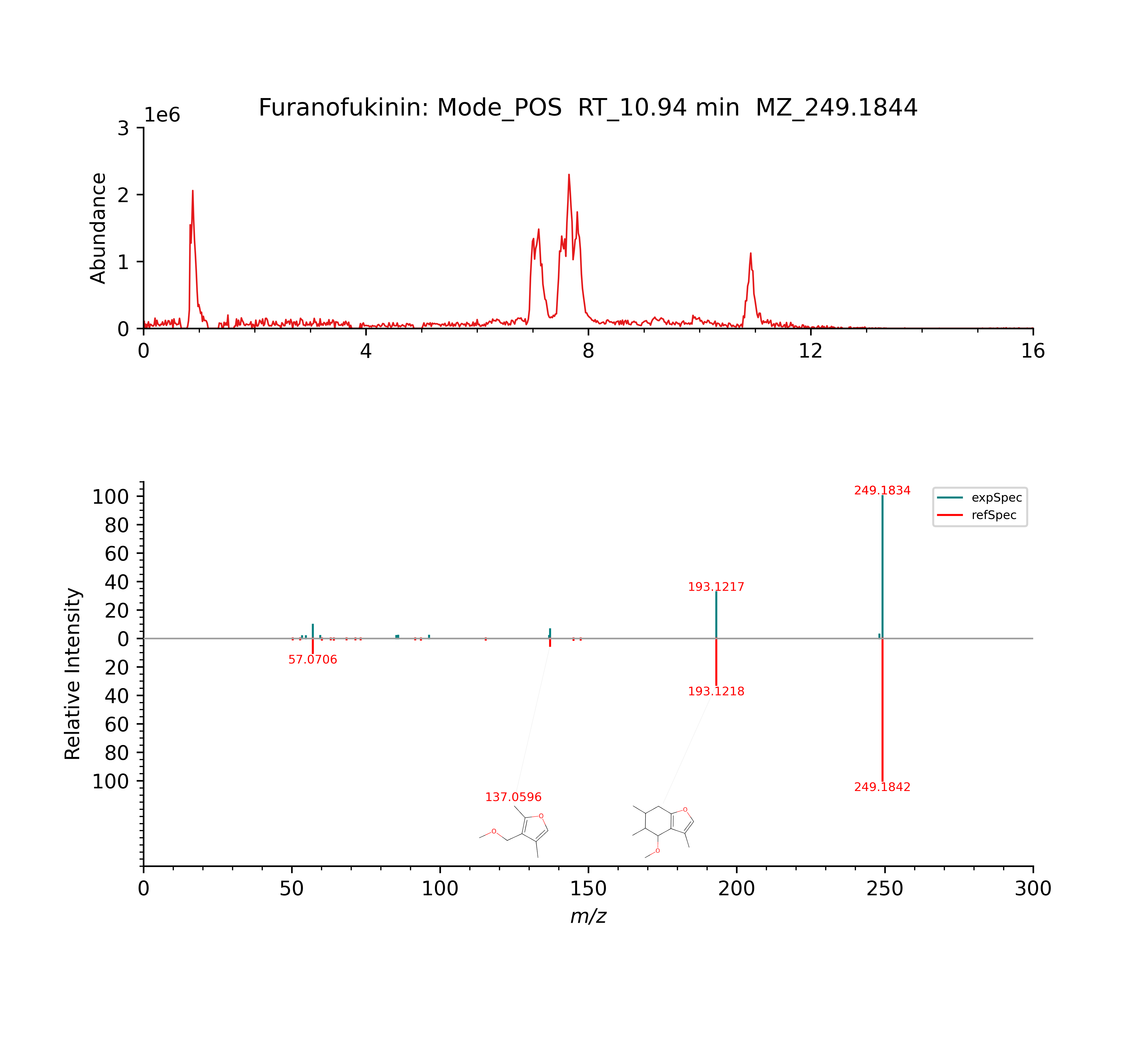

Supplement: Supplementary file 1 [file molecules-29-02840-s001.zip › Supplementary Figure s1/Identification from HerbDB datebase/png/compound00170.png]

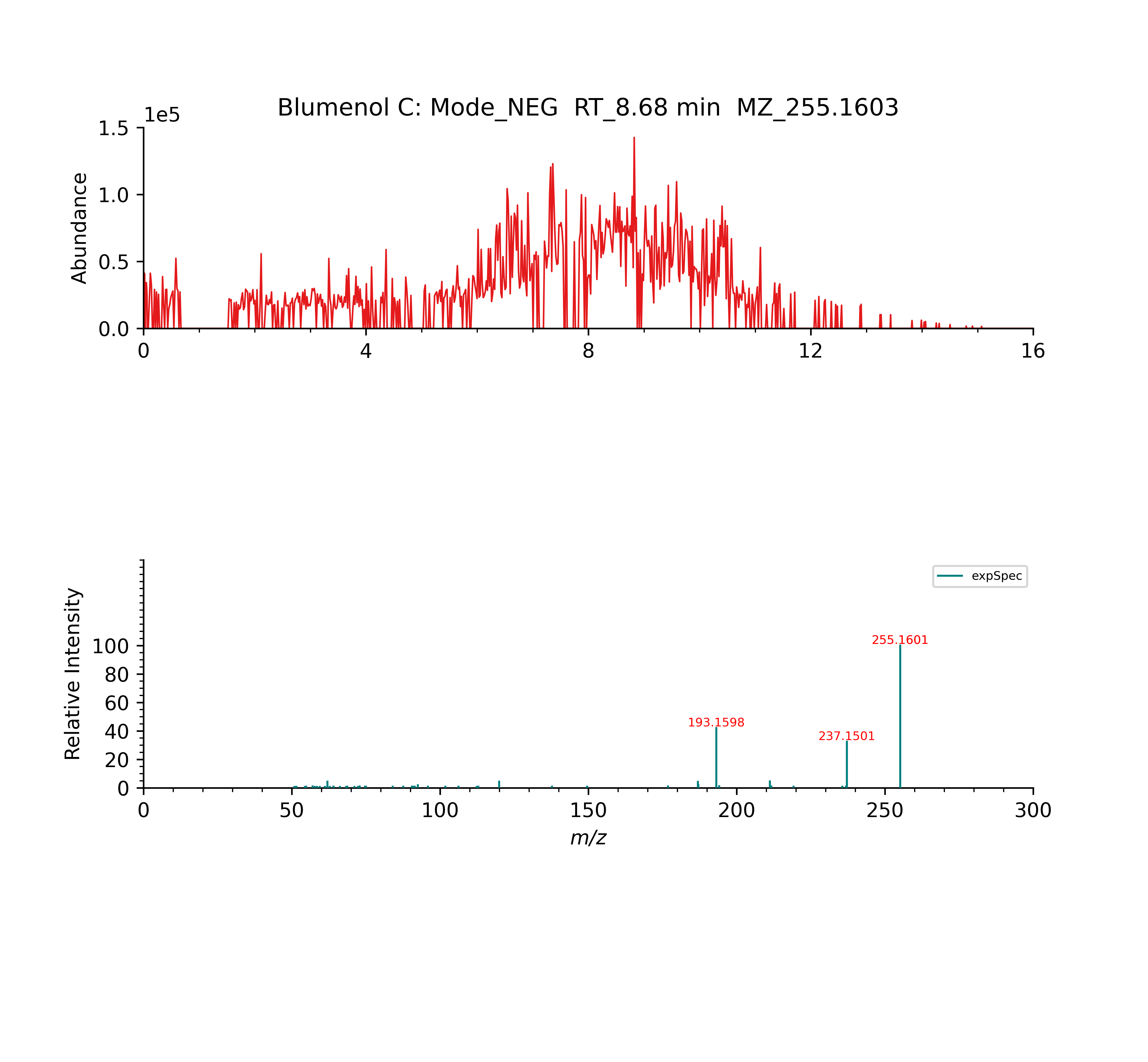

Supplement: Supplementary file 1 [file molecules-29-02840-s001.zip › Supplementary Figure s1/Identification from HerbDB datebase/png/compound00172.png]

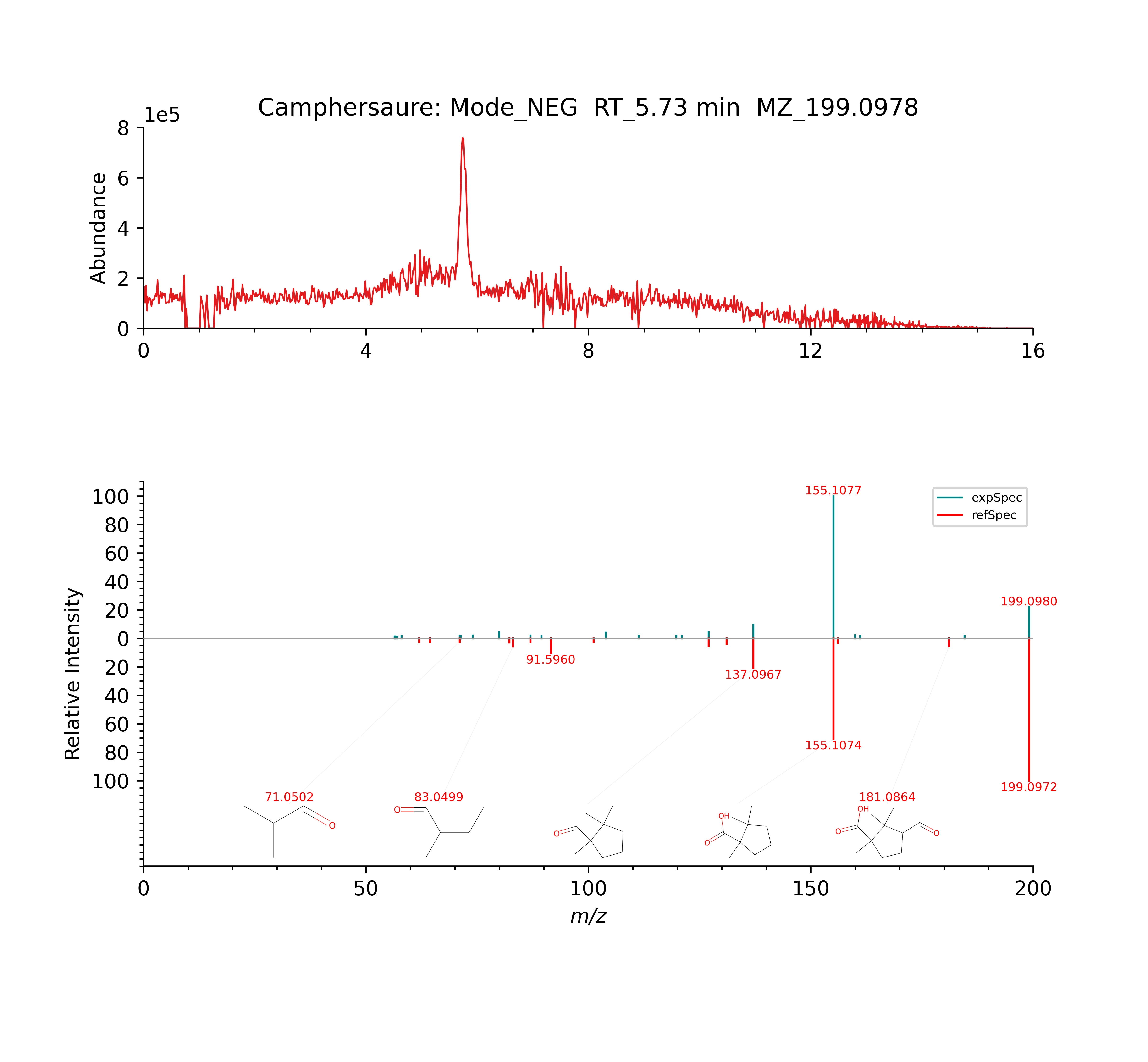

Supplement: Supplementary file 1 [file molecules-29-02840-s001.zip › Supplementary Figure s1/Identification from HerbDB datebase/png/compound00178.png]

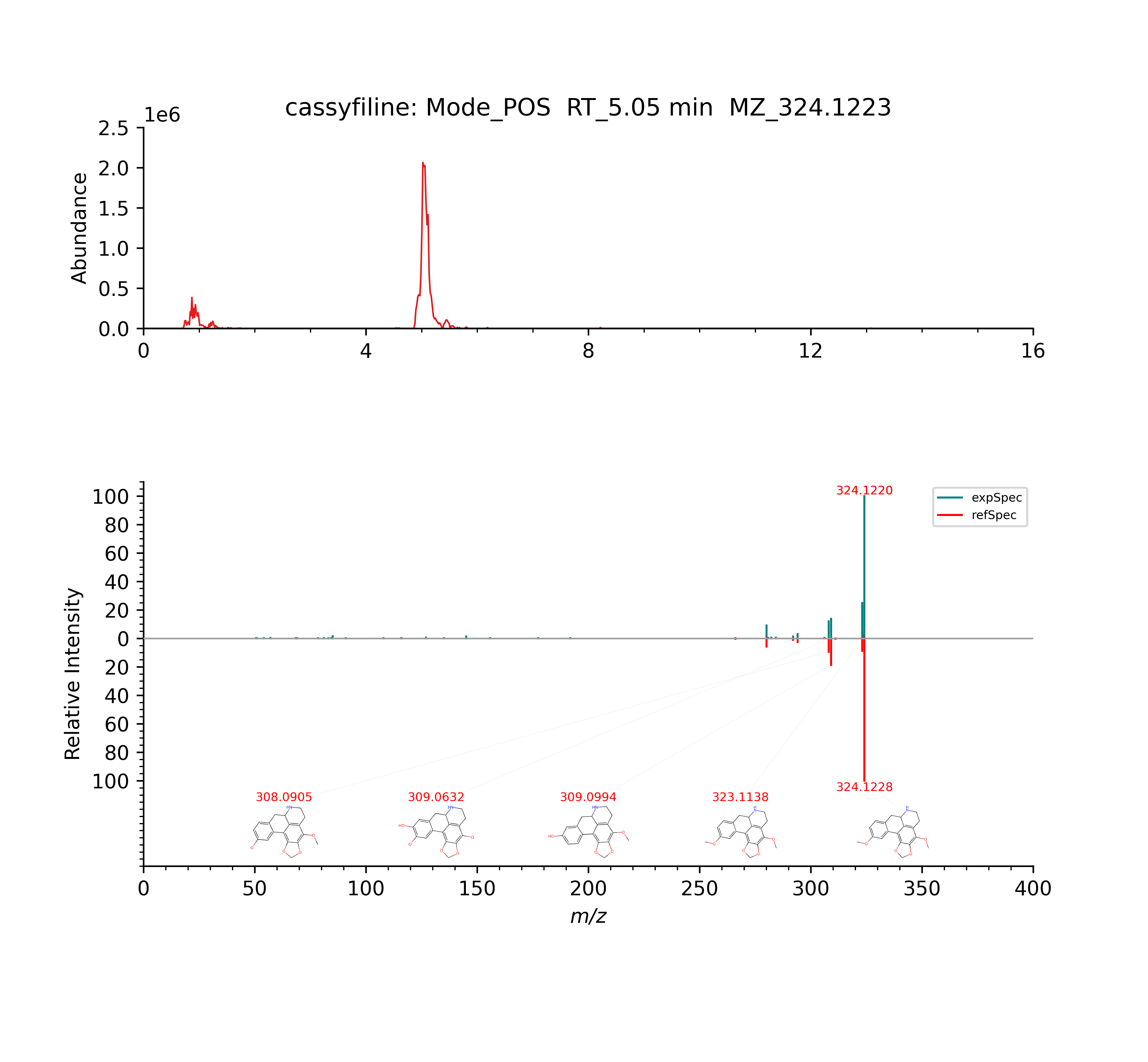

Supplement: Supplementary file 1 [file molecules-29-02840-s001.zip › Supplementary Figure s1/Identification from HerbDB datebase/png/compound00179.png]

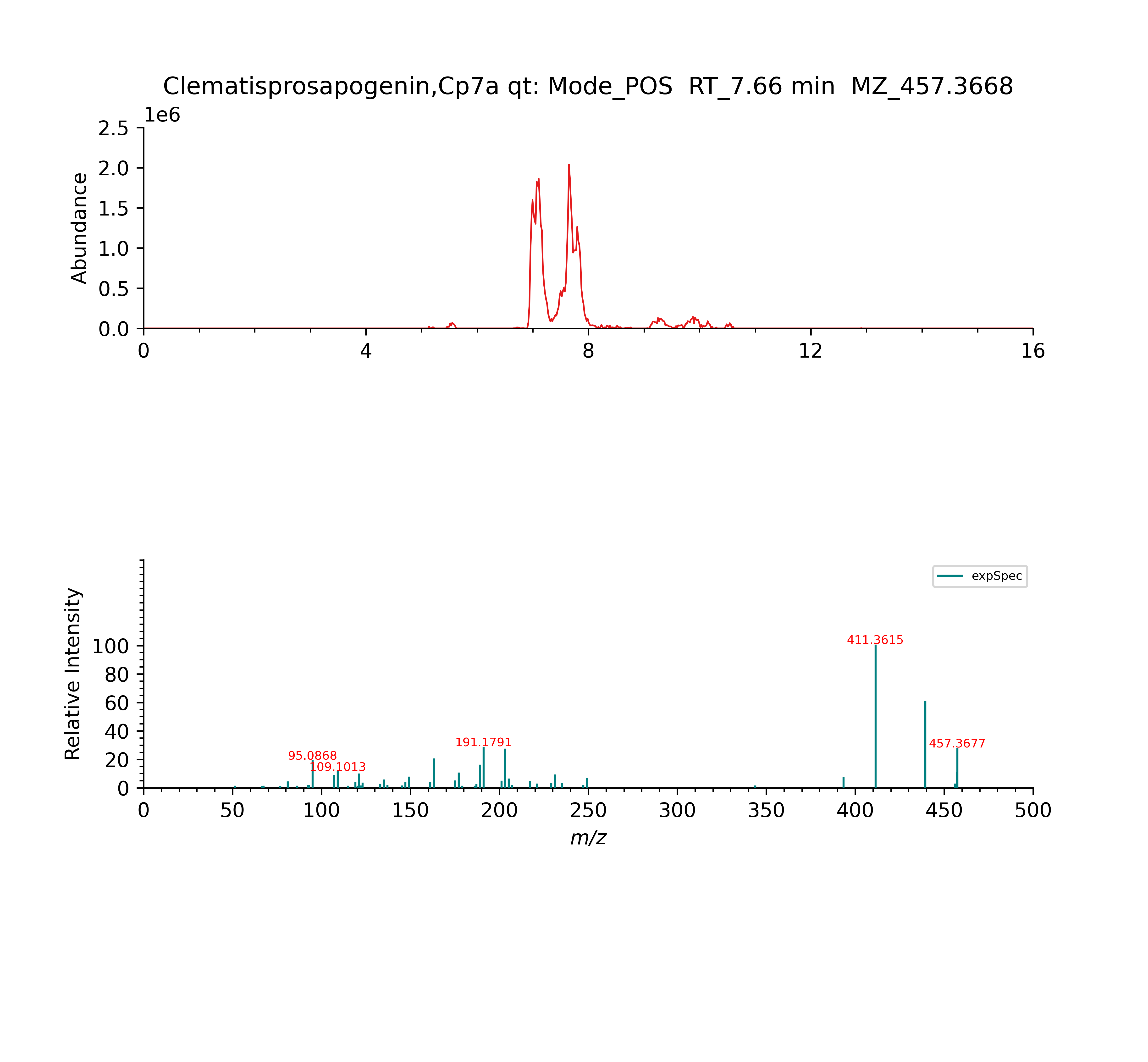

Supplement: Supplementary file 1 [file molecules-29-02840-s001.zip › Supplementary Figure s1/Identification from HerbDB datebase/png/compound00184.png]

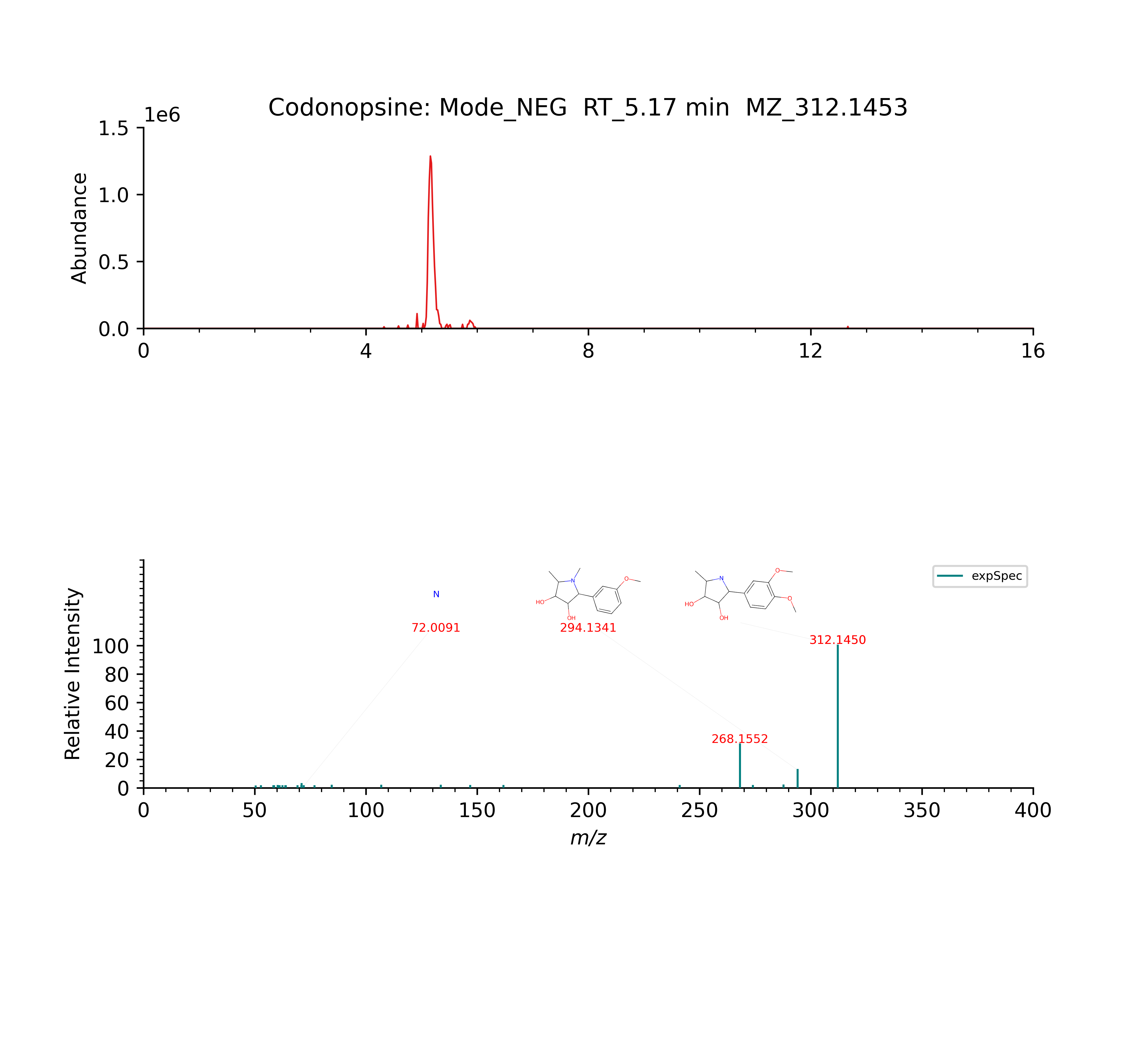

Supplement: Supplementary file 1 [file molecules-29-02840-s001.zip › Supplementary Figure s1/Identification from HerbDB datebase/png/compound00185.png]

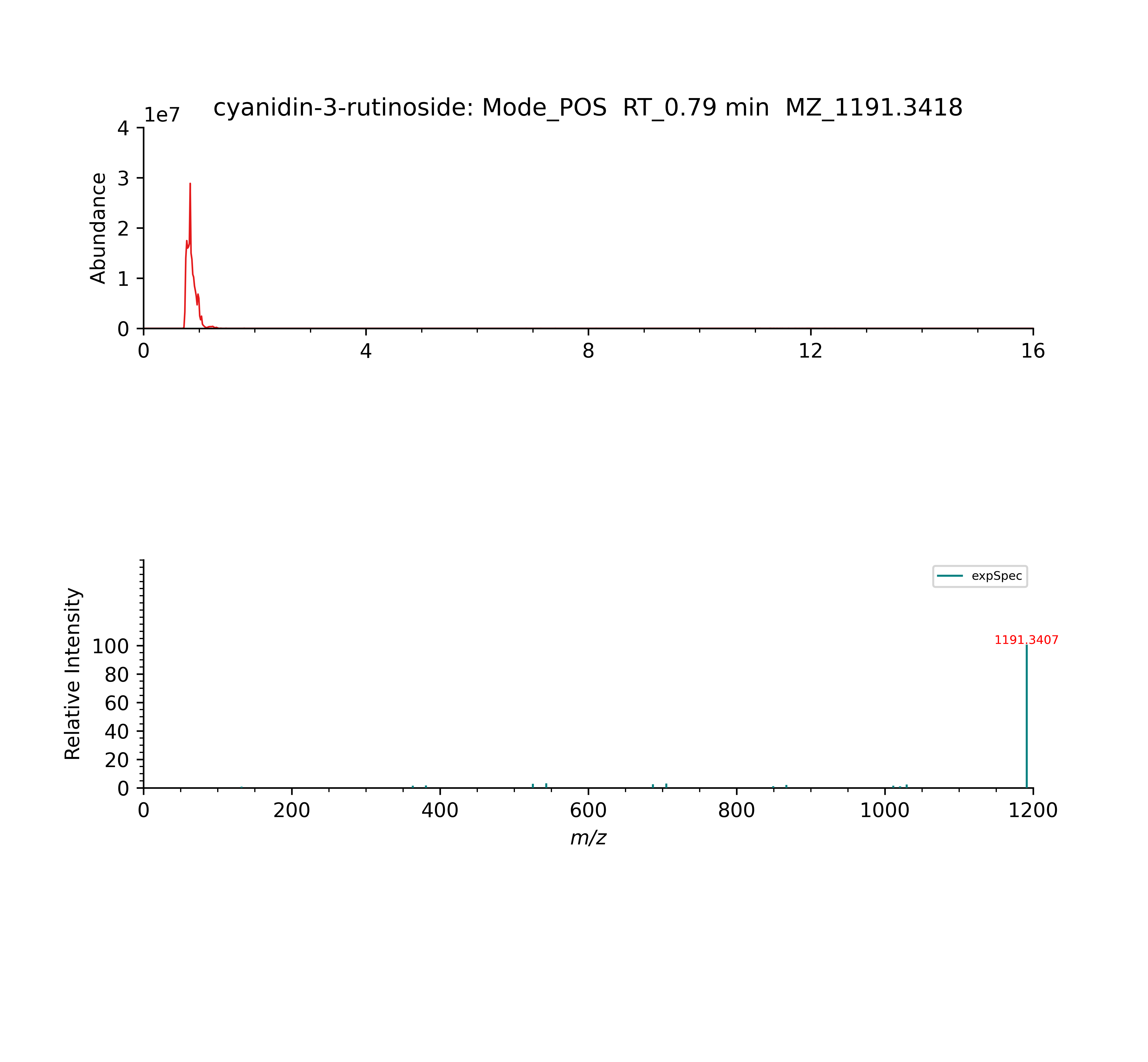

Supplement: Supplementary file 1 [file molecules-29-02840-s001.zip › Supplementary Figure s1/Identification from HerbDB datebase/png/compound00186.png]

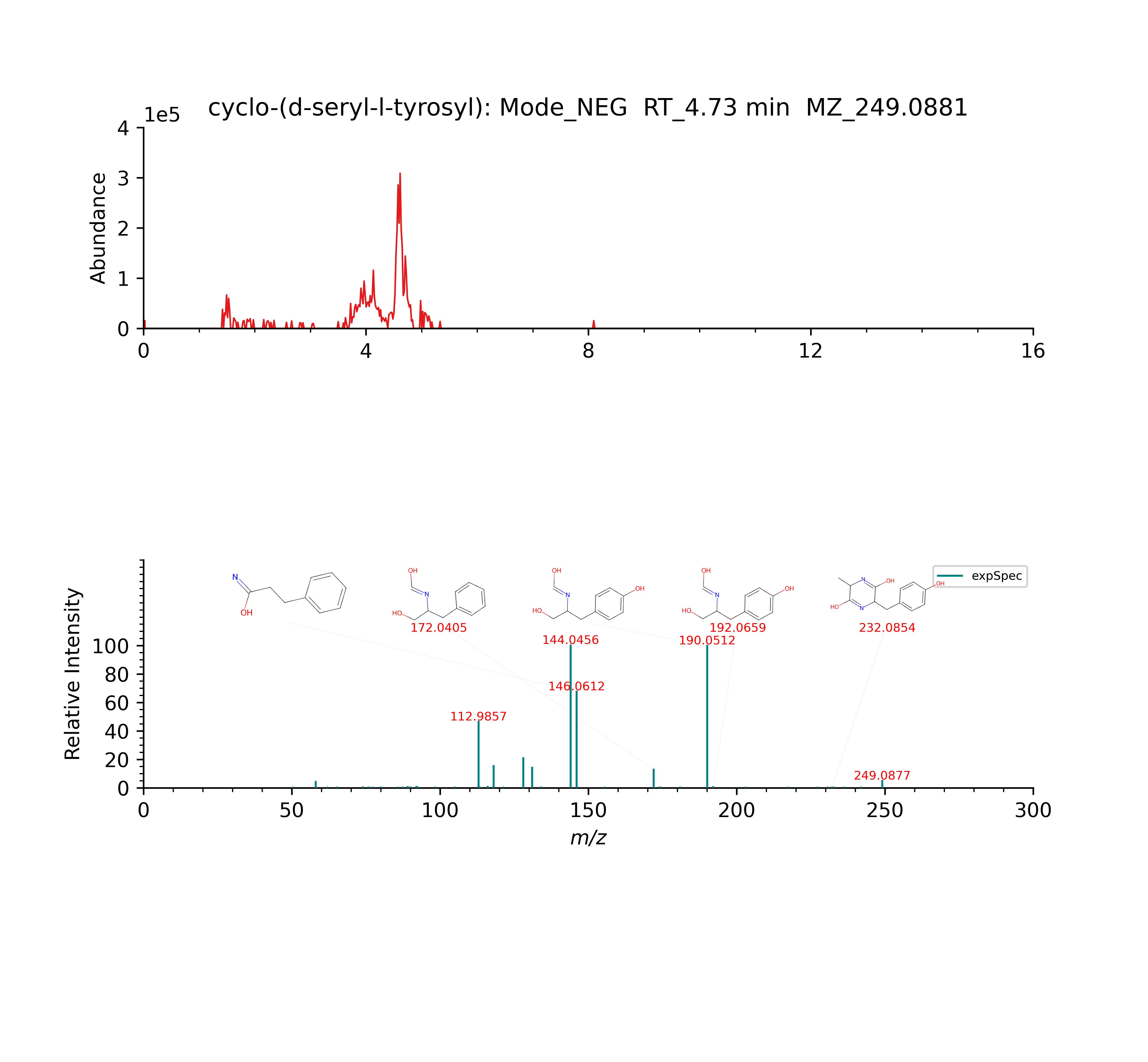

Supplement: Supplementary file 1 [file molecules-29-02840-s001.zip › Supplementary Figure s1/Identification from HerbDB datebase/png/compound00189.png]

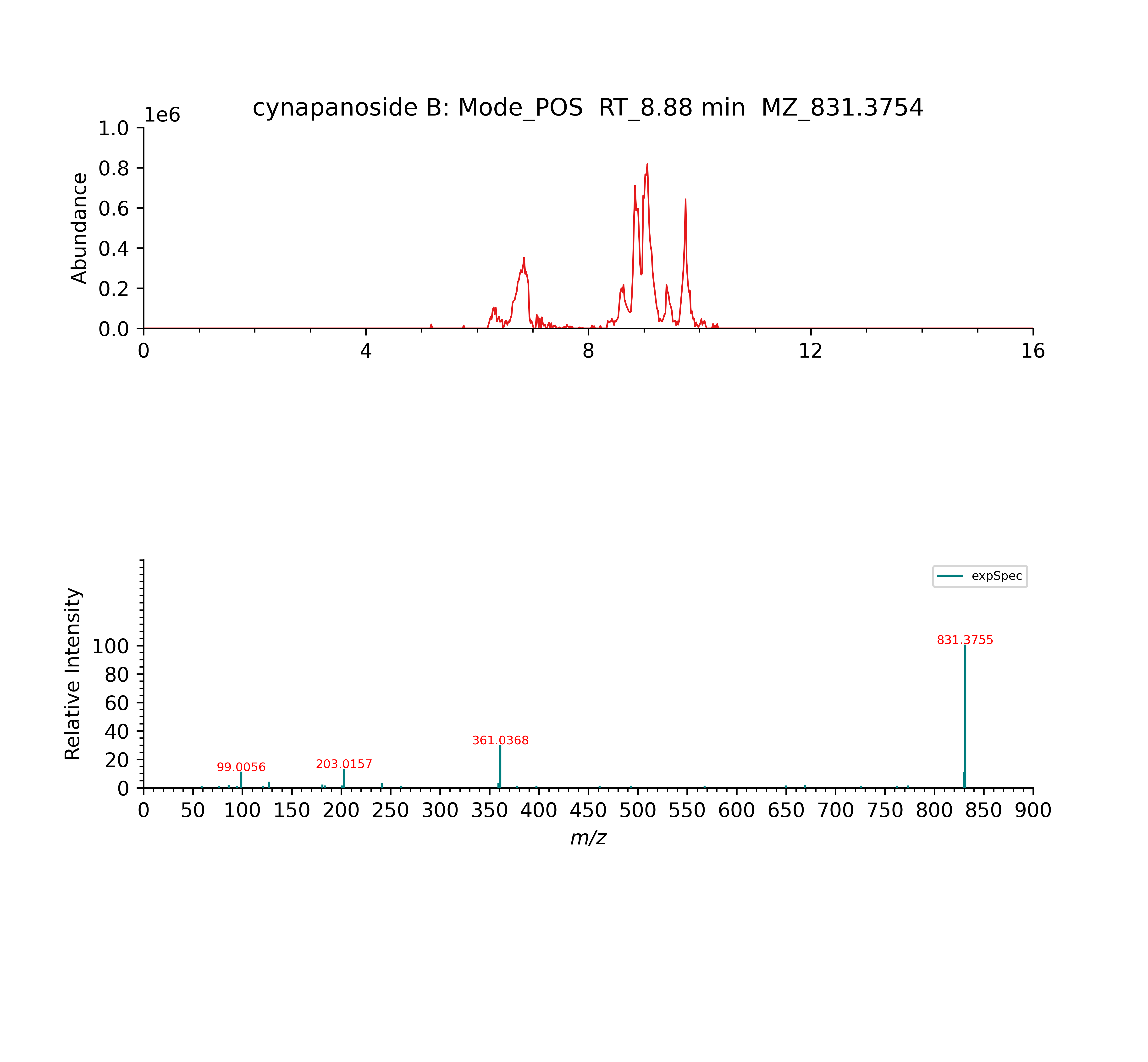

Supplement: Supplementary file 1 [file molecules-29-02840-s001.zip › Supplementary Figure s1/Identification from HerbDB datebase/png/compound00190.png]

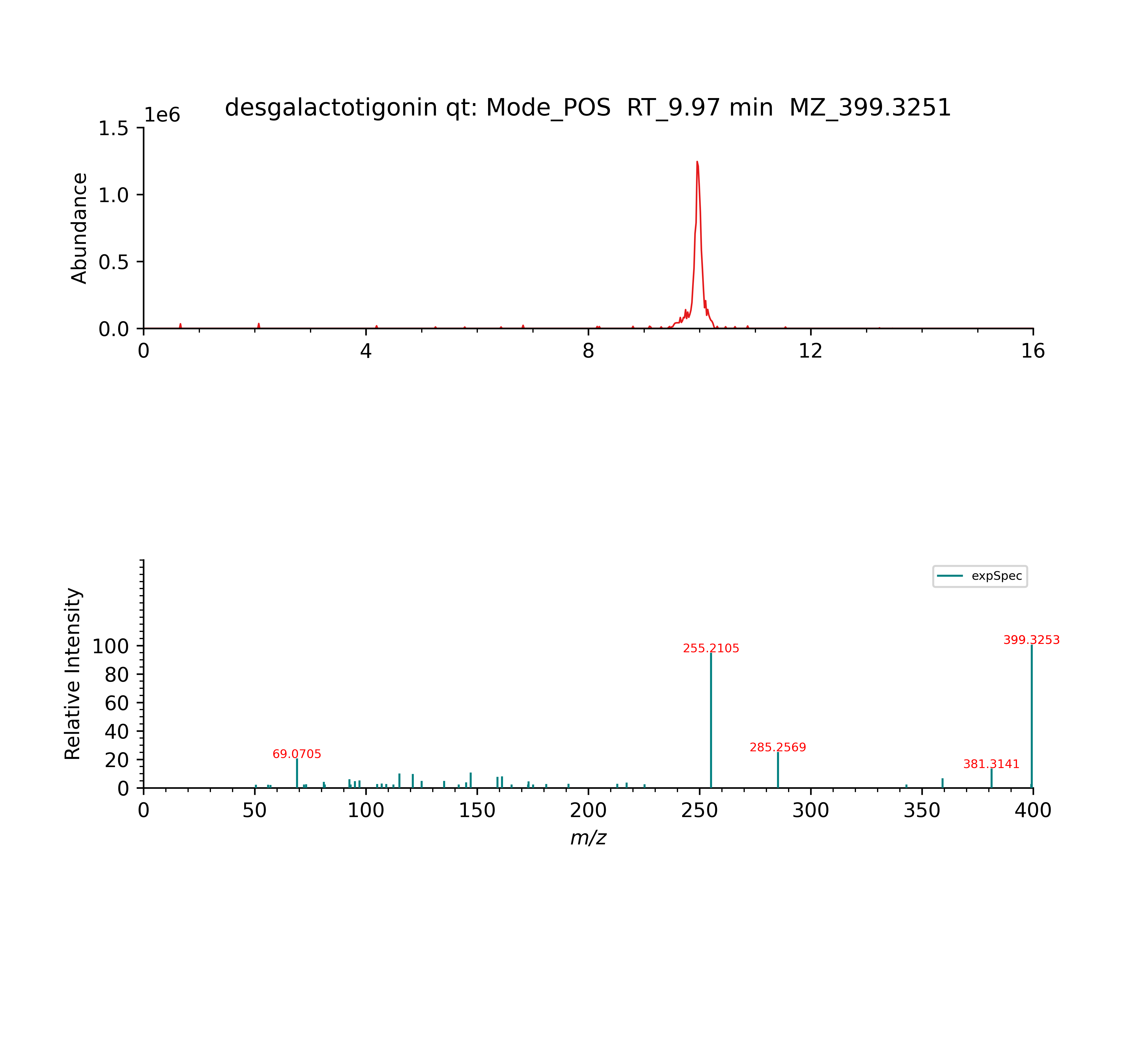

Supplement: Supplementary file 1 [file molecules-29-02840-s001.zip › Supplementary Figure s1/Identification from HerbDB datebase/png/compound00192.png]

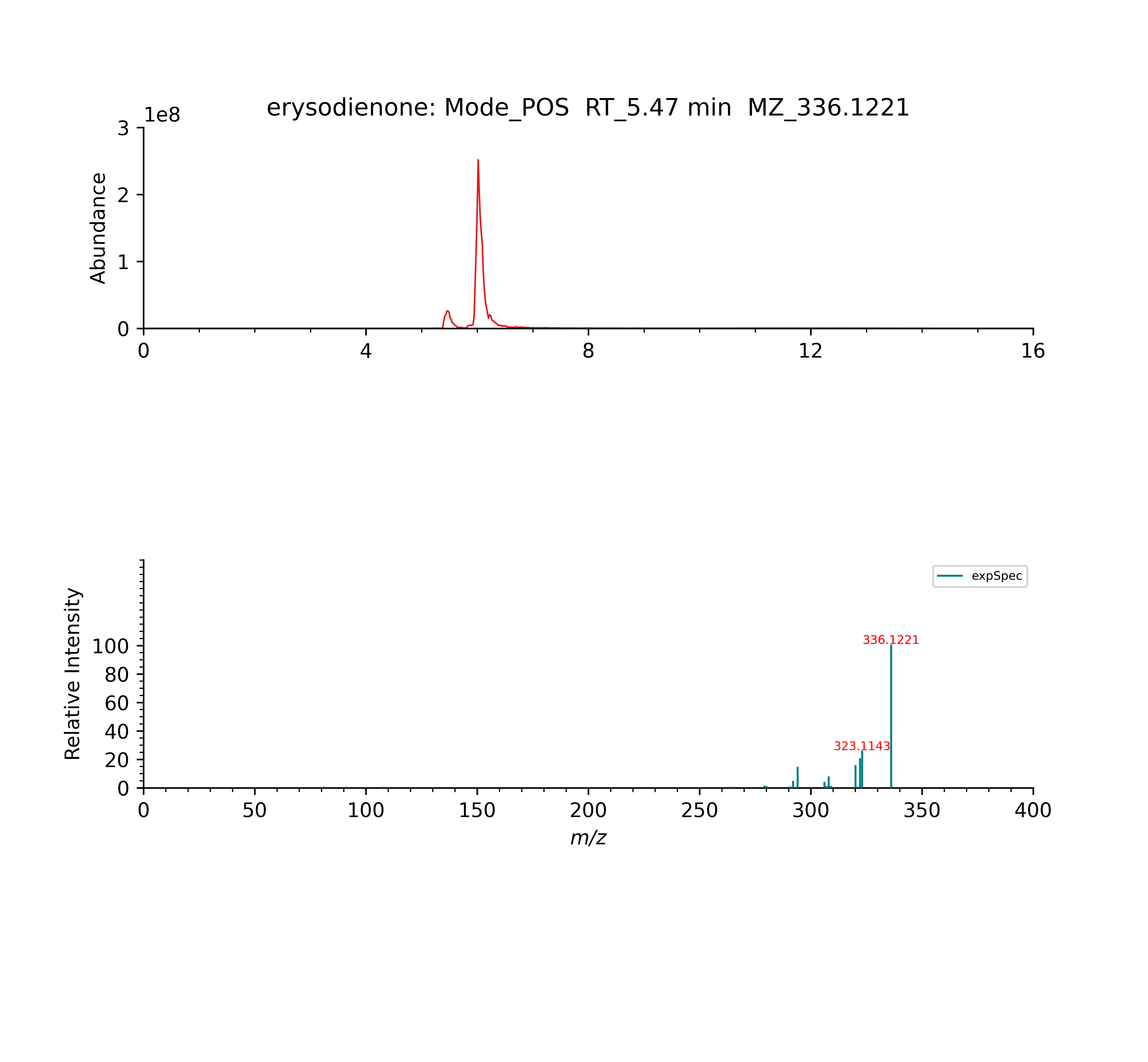

Supplement: Supplementary file 1 [file molecules-29-02840-s001.zip › Supplementary Figure s1/Identification from HerbDB datebase/png/compound00195.png]

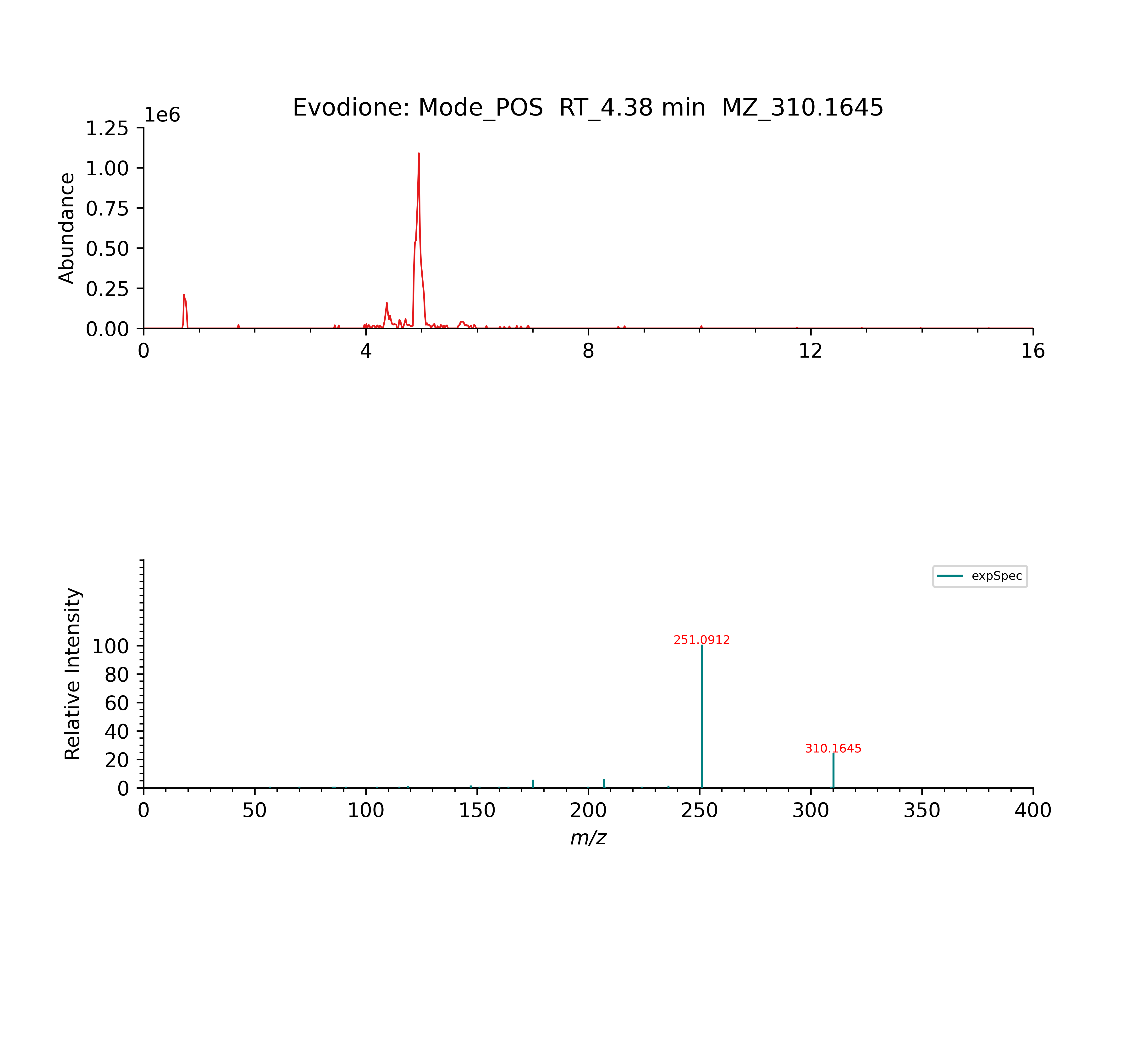

Supplement: Supplementary file 1 [file molecules-29-02840-s001.zip › Supplementary Figure s1/Identification from HerbDB datebase/png/compound00200.png]

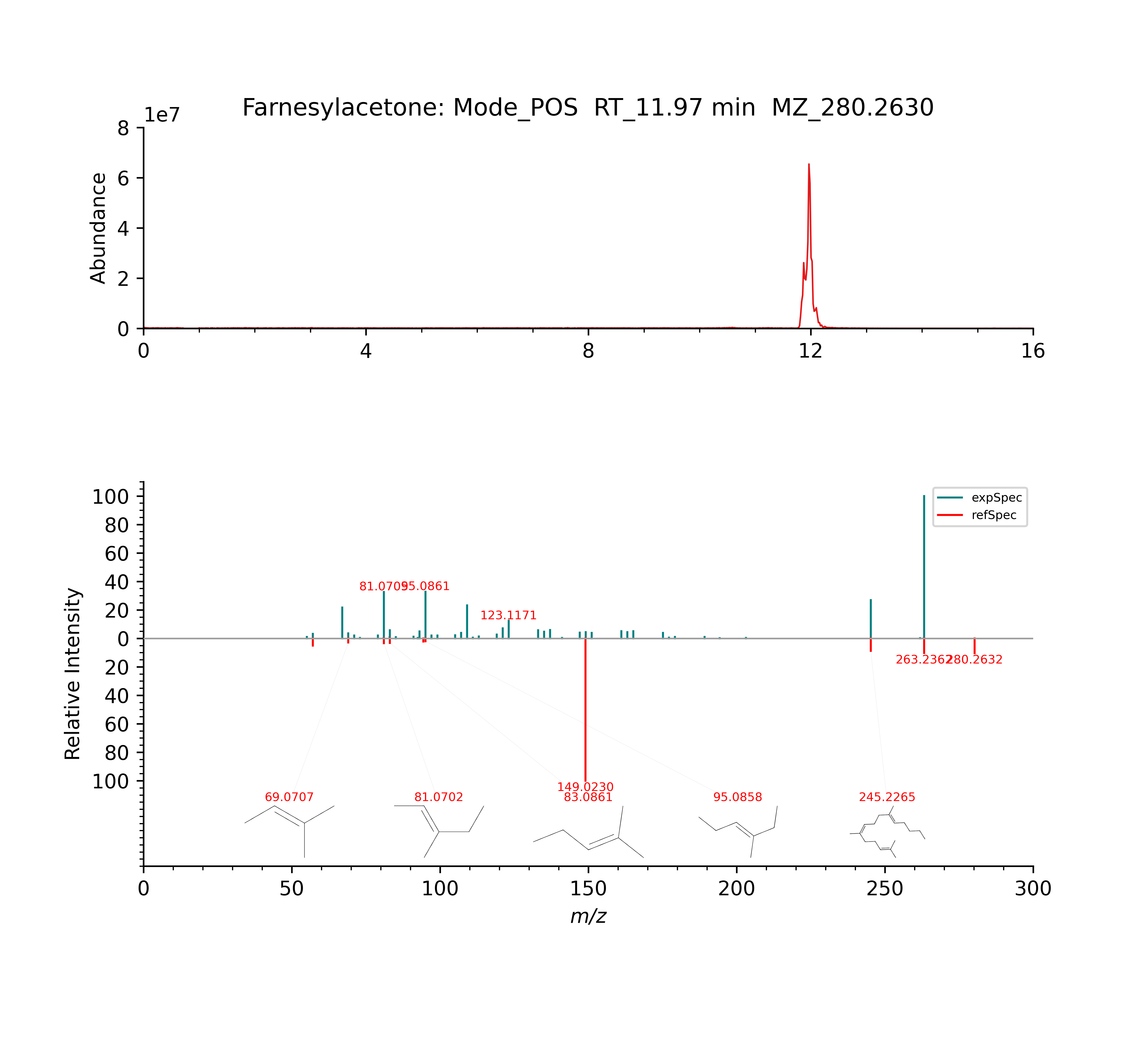

Supplement: Supplementary file 1 [file molecules-29-02840-s001.zip › Supplementary Figure s1/Identification from HerbDB datebase/png/compound00201.png]

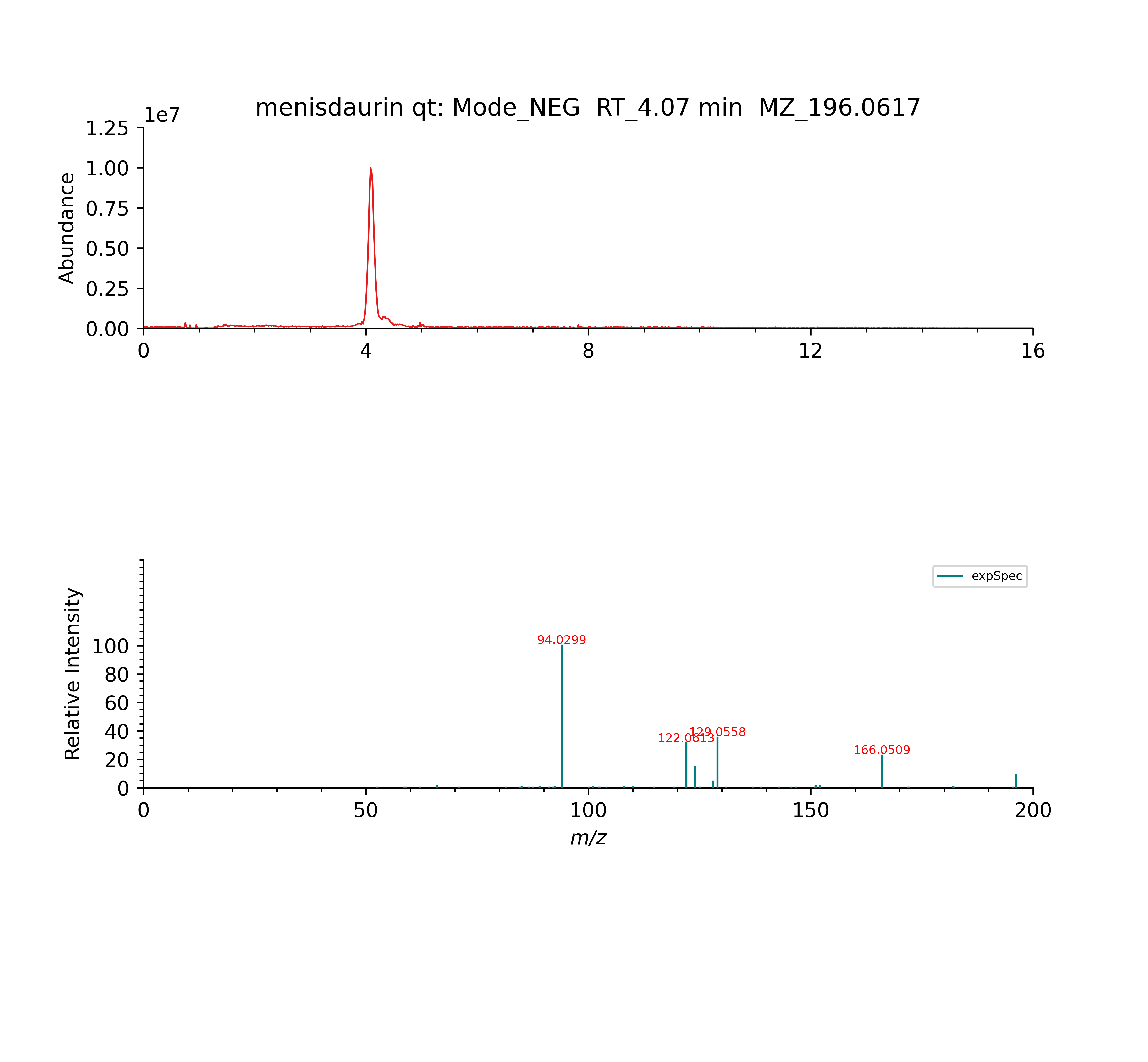

Supplement: Supplementary file 1 [file molecules-29-02840-s001.zip › Supplementary Figure s1/Identification from HerbDB datebase/png/compound00204.png]

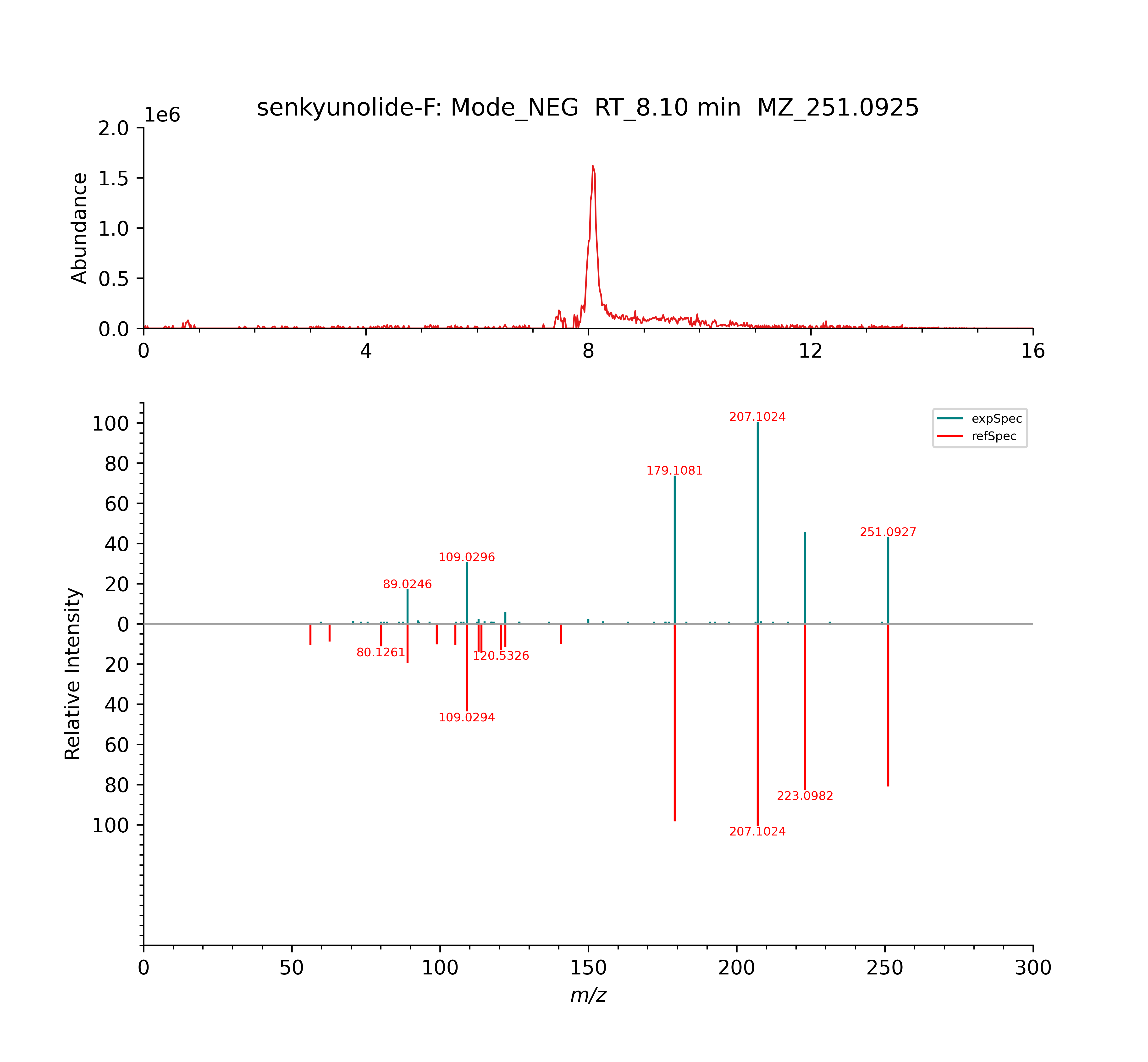

Supplement: Supplementary file 1 [file molecules-29-02840-s001.zip › Supplementary Figure s1/Identification from HerbDB datebase/png/compound00207.png]

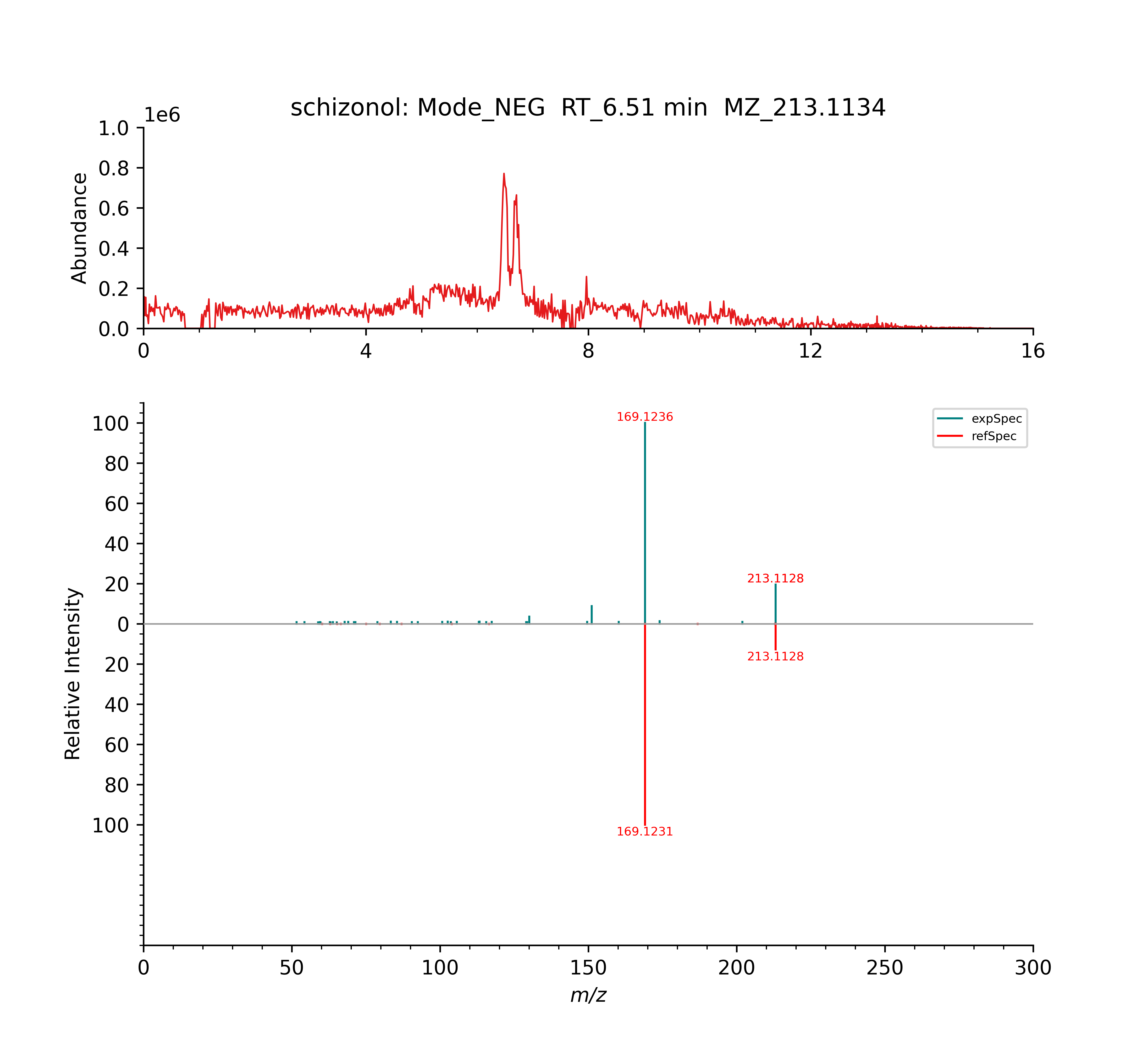

Supplement: Supplementary file 1 [file molecules-29-02840-s001.zip › Supplementary Figure s1/Identification from HerbDB datebase/png/compound00210.png]

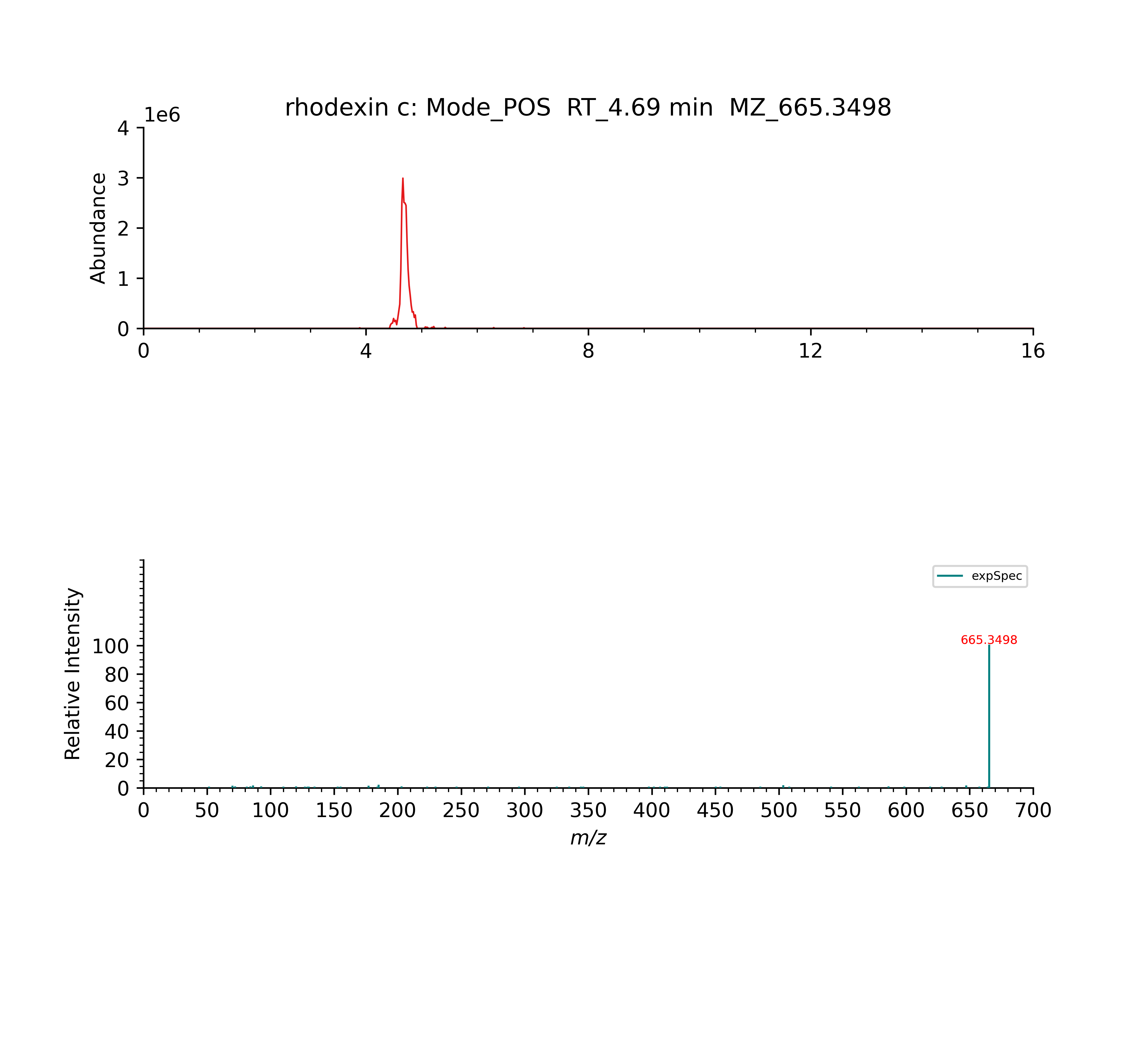

Supplement: Supplementary file 1 [file molecules-29-02840-s001.zip › Supplementary Figure s1/Identification from HerbDB datebase/png/compound00212.png]

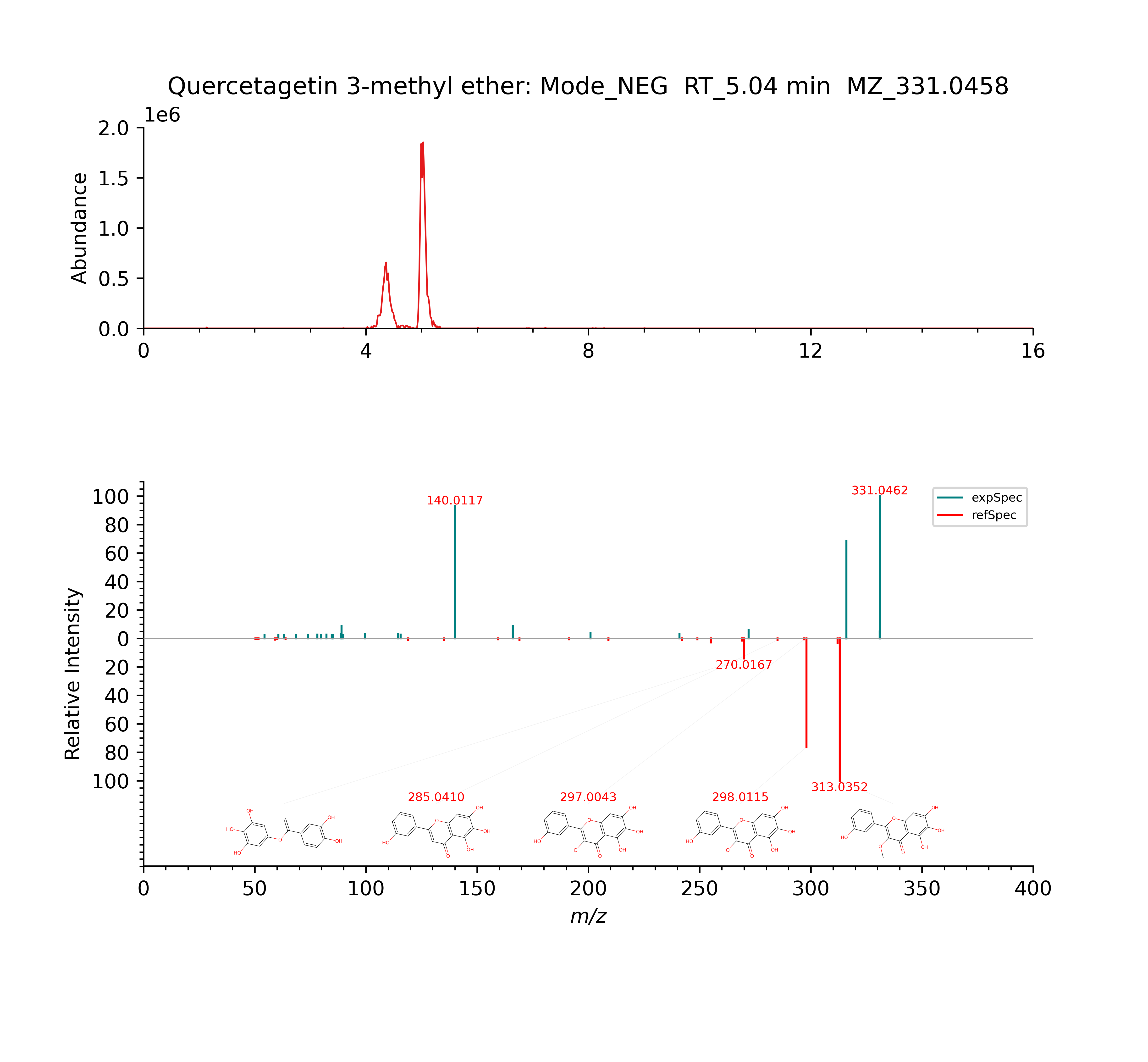

Supplement: Supplementary file 1 [file molecules-29-02840-s001.zip › Supplementary Figure s1/Identification from HerbDB datebase/png/compound00215.png]

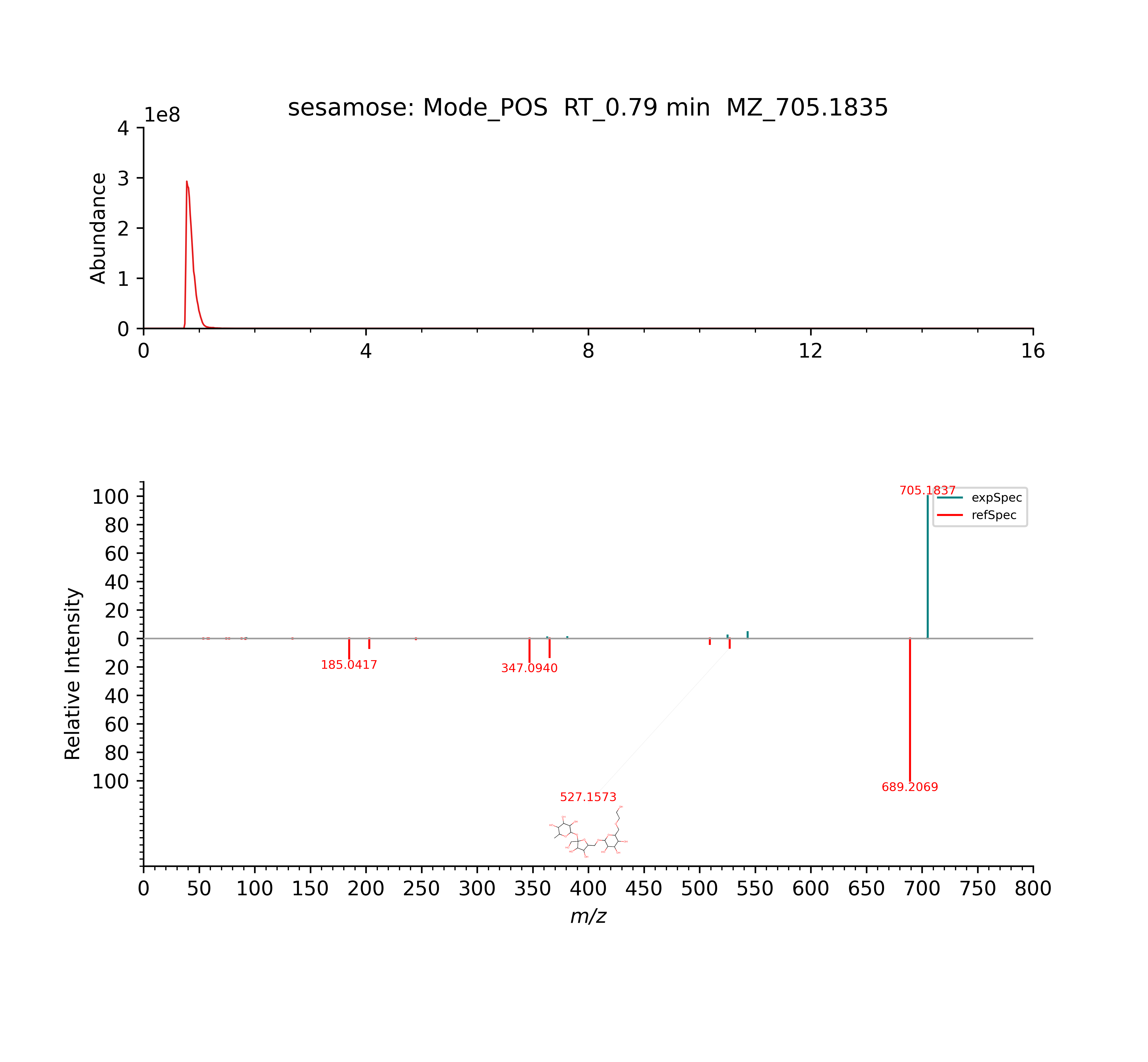

Supplement: Supplementary file 1 [file molecules-29-02840-s001.zip › Supplementary Figure s1/Identification from HerbDB datebase/png/compound00220.png]

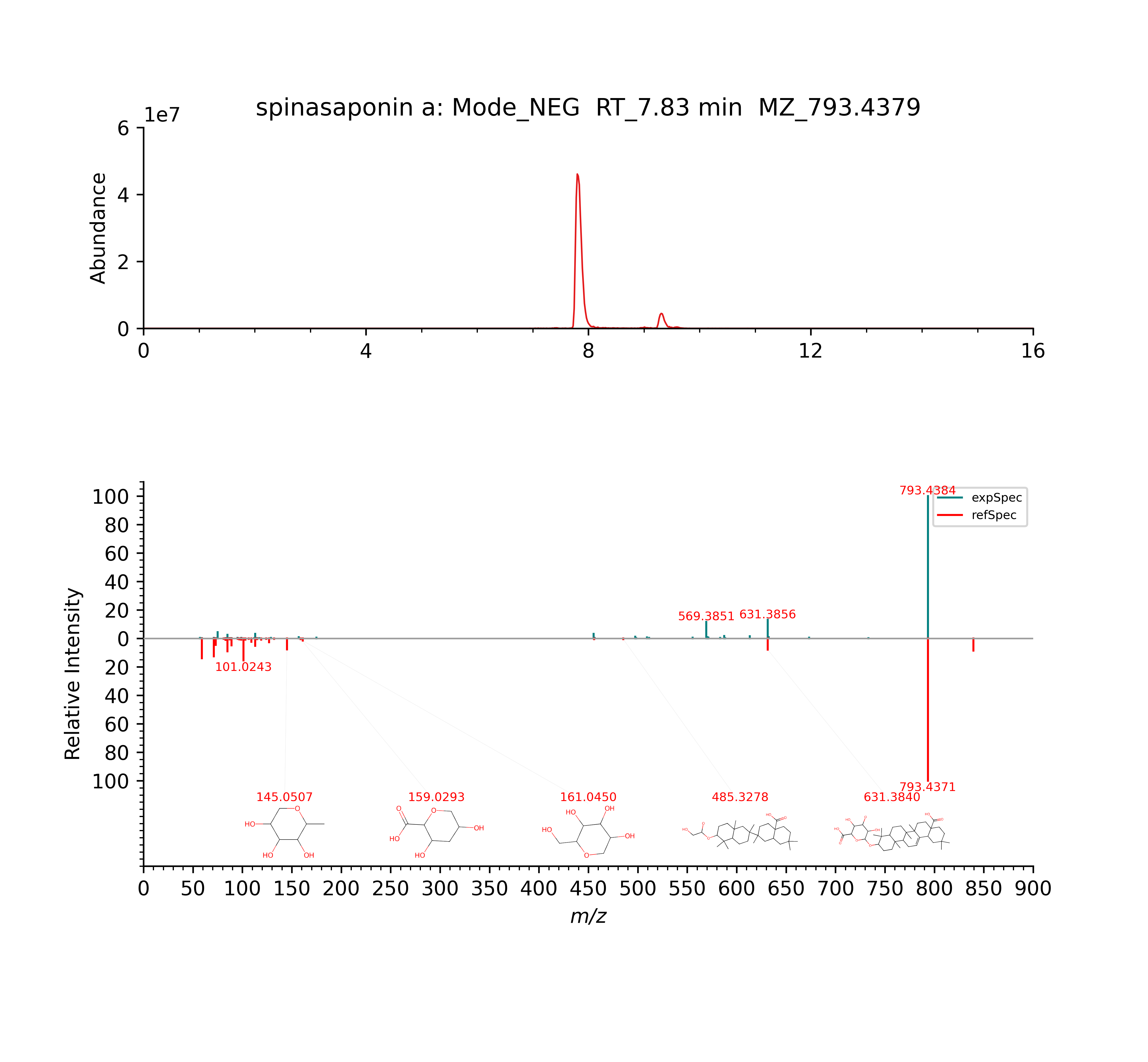

Supplement: Supplementary file 1 [file molecules-29-02840-s001.zip › Supplementary Figure s1/Identification from HerbDB datebase/png/compound00225.png]

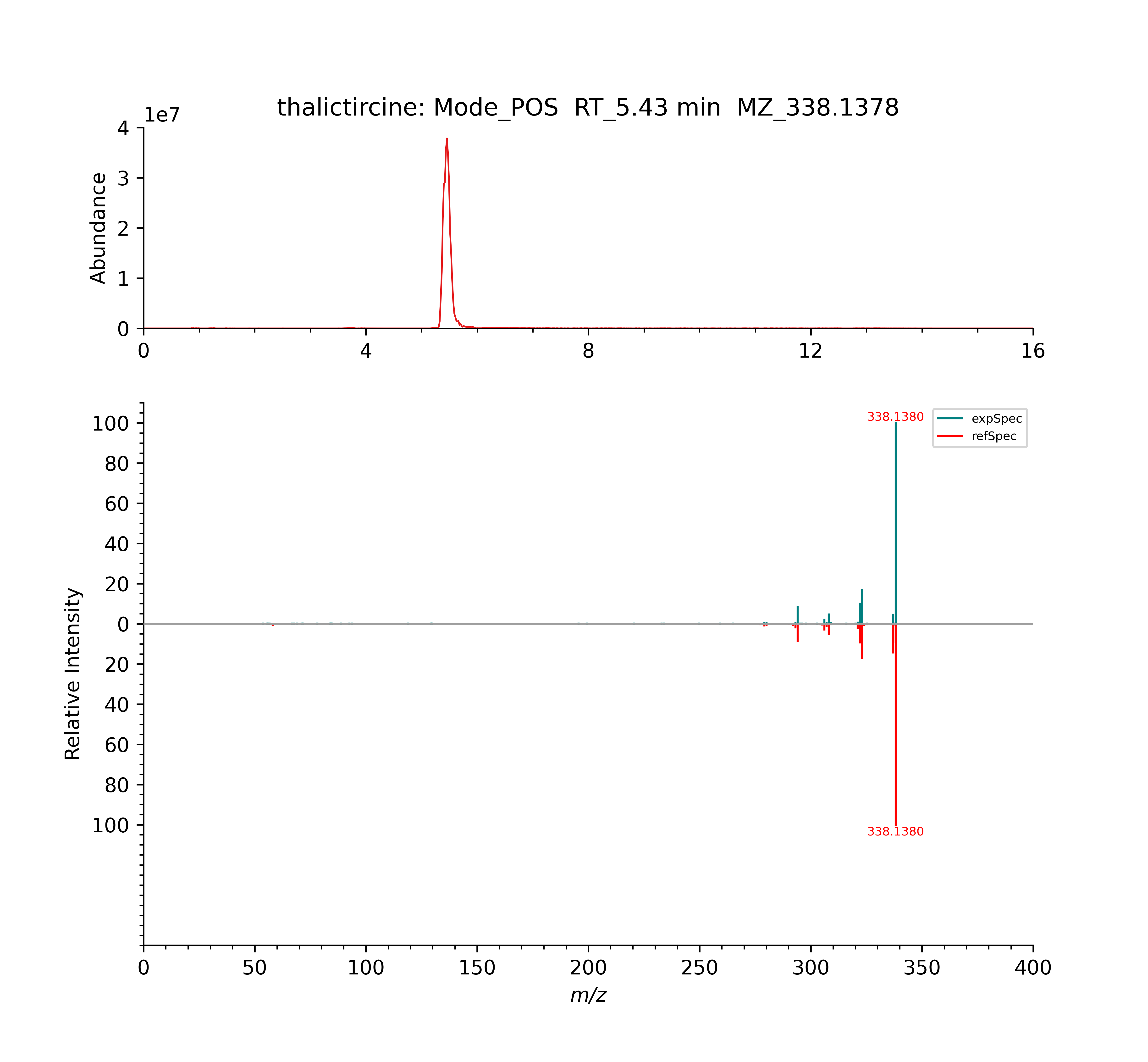

Supplement: Supplementary file 1 [file molecules-29-02840-s001.zip › Supplementary Figure s1/Identification from HerbDB datebase/png/compound00230.png]

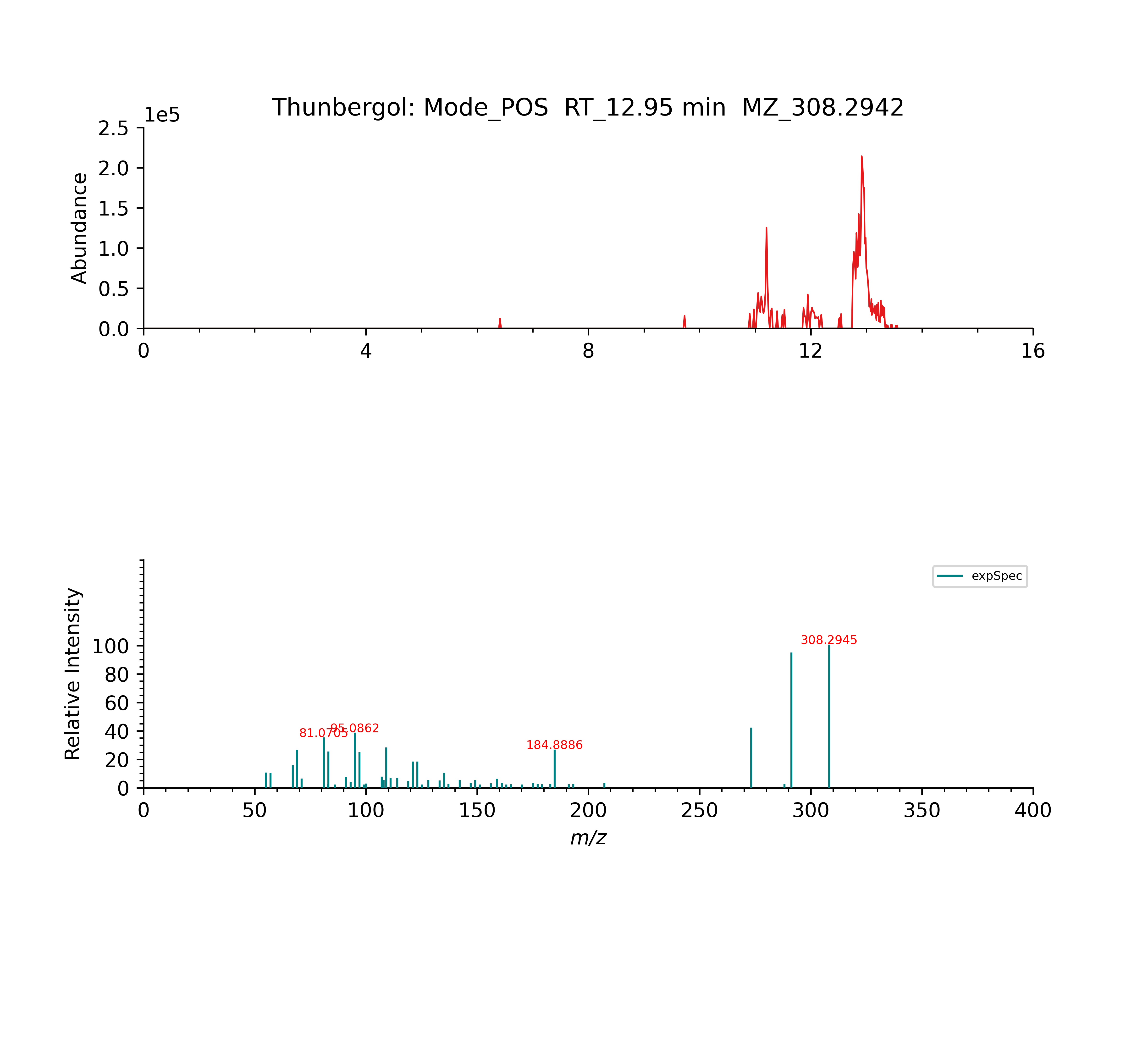

Supplement: Supplementary file 1 [file molecules-29-02840-s001.zip › Supplementary Figure s1/Identification from HerbDB datebase/png/compound00231.png]

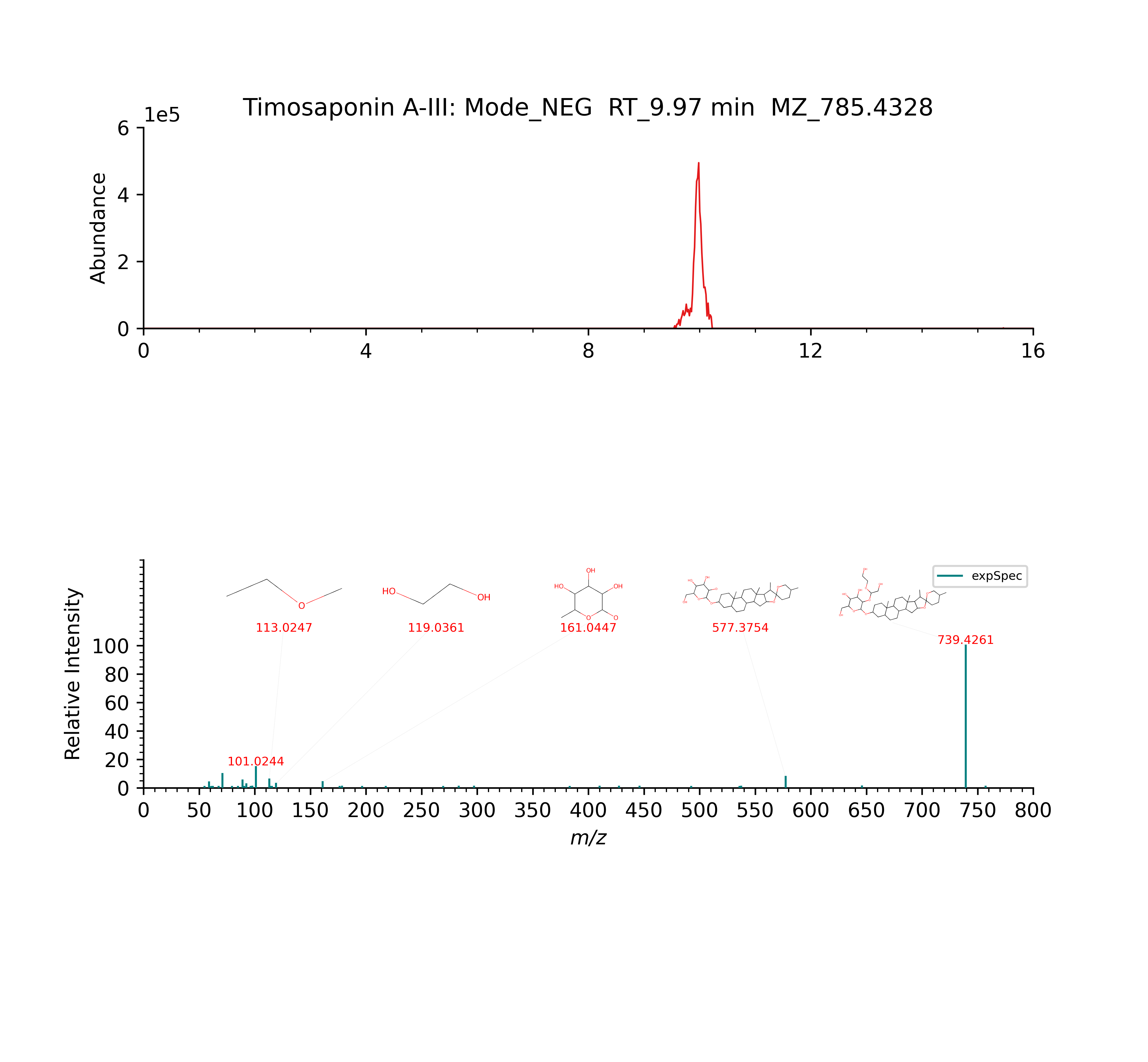

Supplement: Supplementary file 1 [file molecules-29-02840-s001.zip › Supplementary Figure s1/Identification from HerbDB datebase/png/compound00232.png]

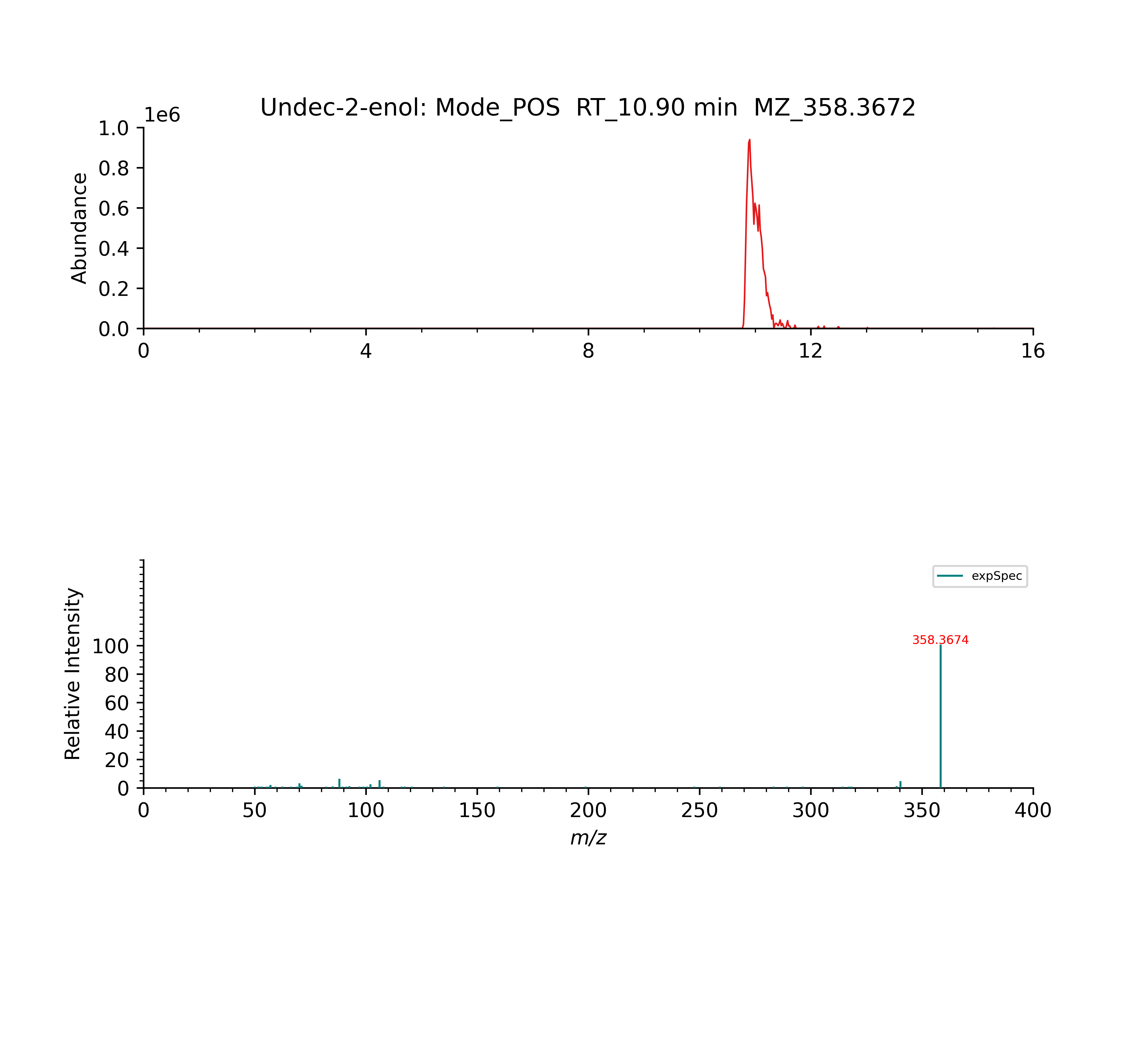

Supplement: Supplementary file 1 [file molecules-29-02840-s001.zip › Supplementary Figure s1/Identification from HerbDB datebase/png/compound00234.png]

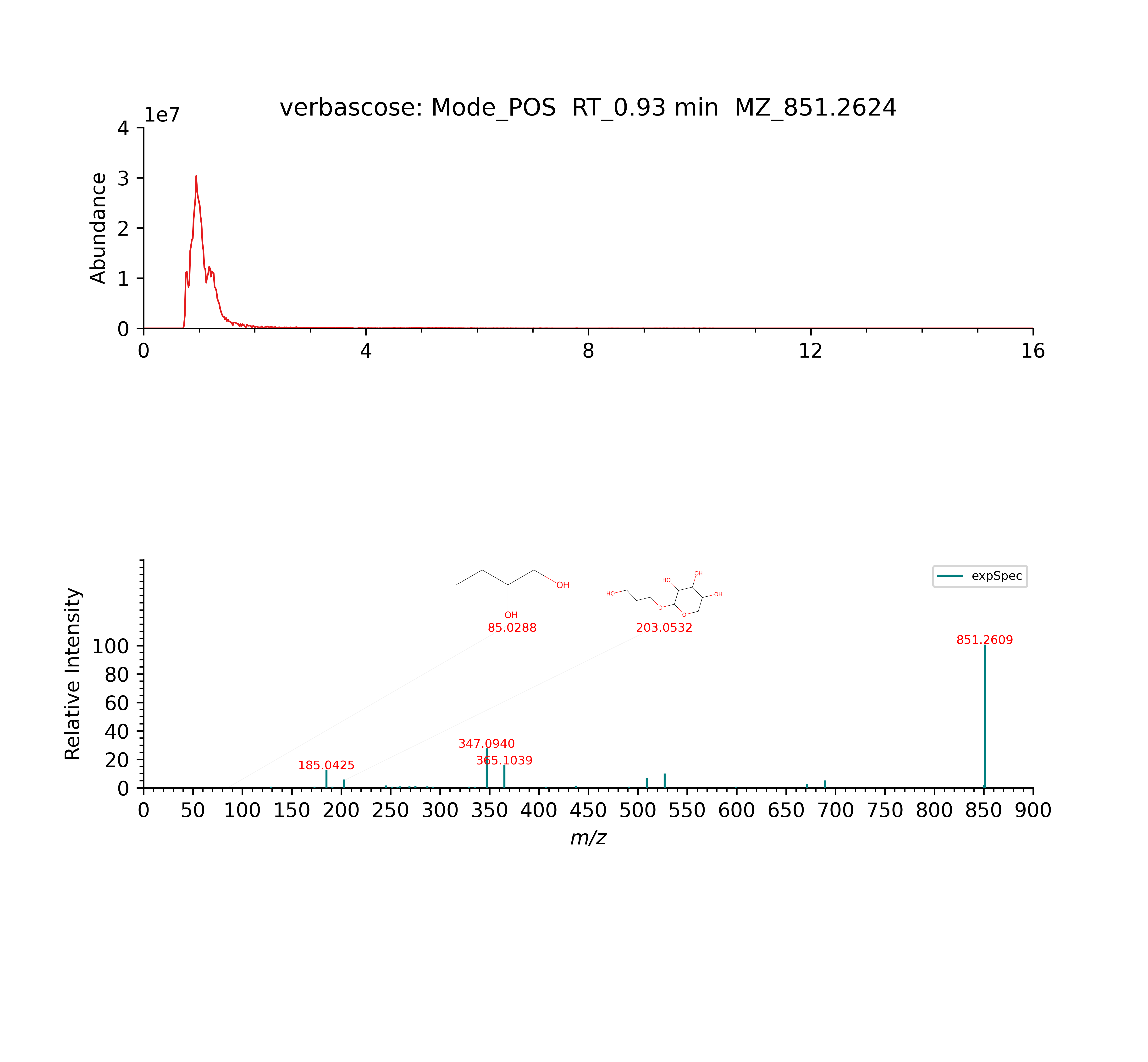

Supplement: Supplementary file 1 [file molecules-29-02840-s001.zip › Supplementary Figure s1/Identification from HerbDB datebase/png/compound00235.png]

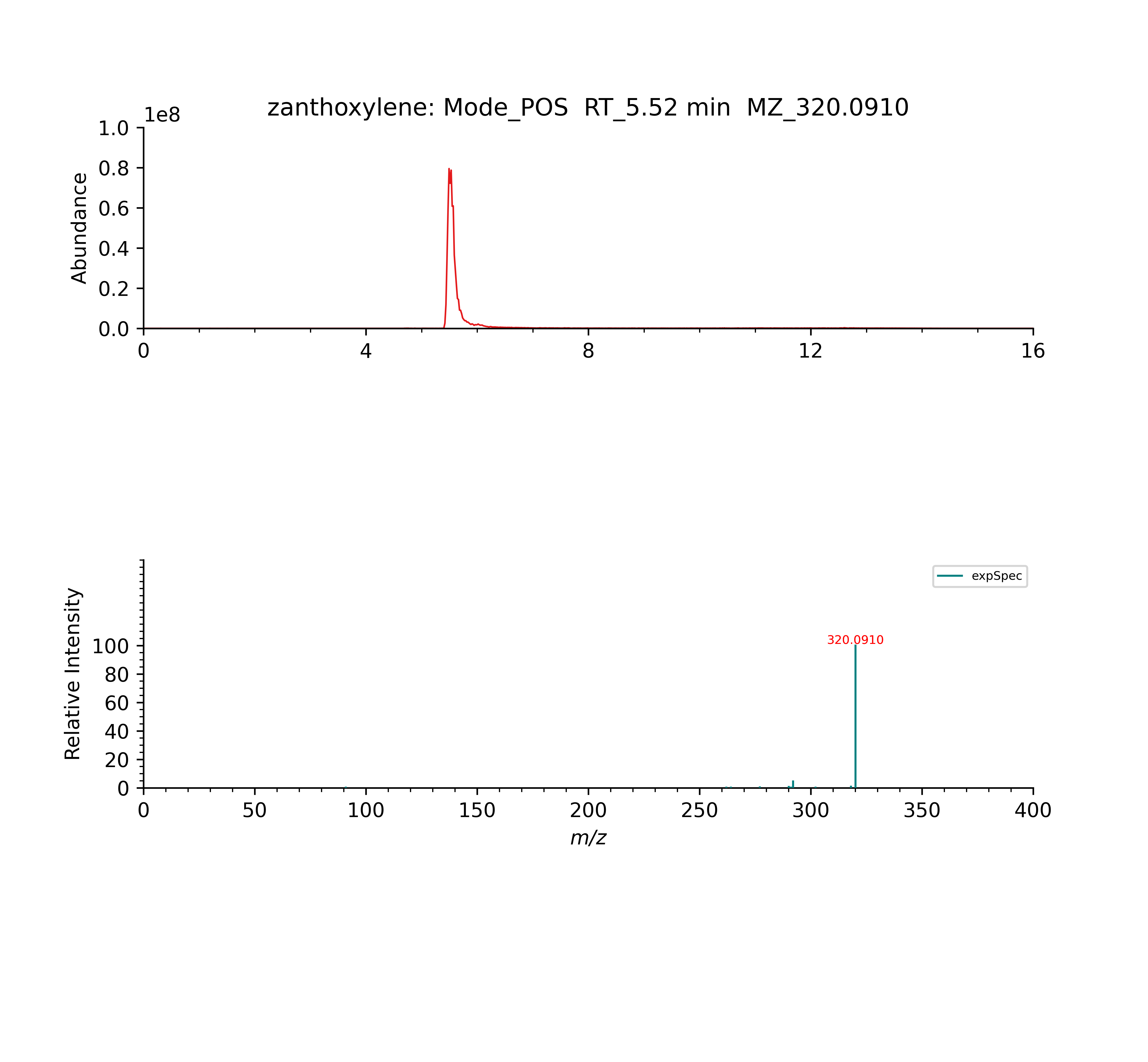

Supplement: Supplementary file 1 [file molecules-29-02840-s001.zip › Supplementary Figure s1/Identification from HerbDB datebase/png/compound00236.png]

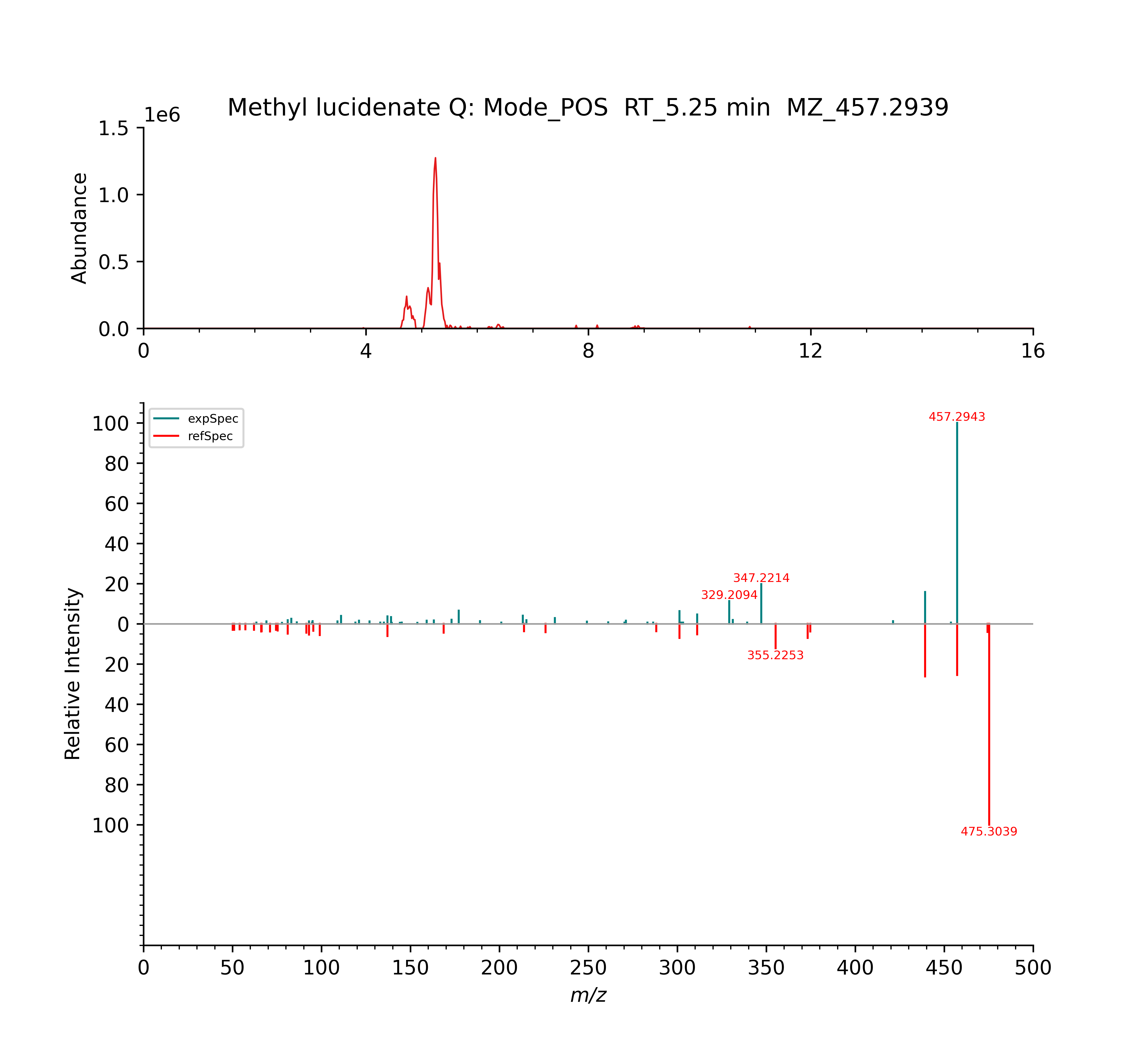

Supplement: Supplementary file 1 [file molecules-29-02840-s001.zip › Supplementary Figure s1/Identification from HerbDB datebase/png/compound00239.png]

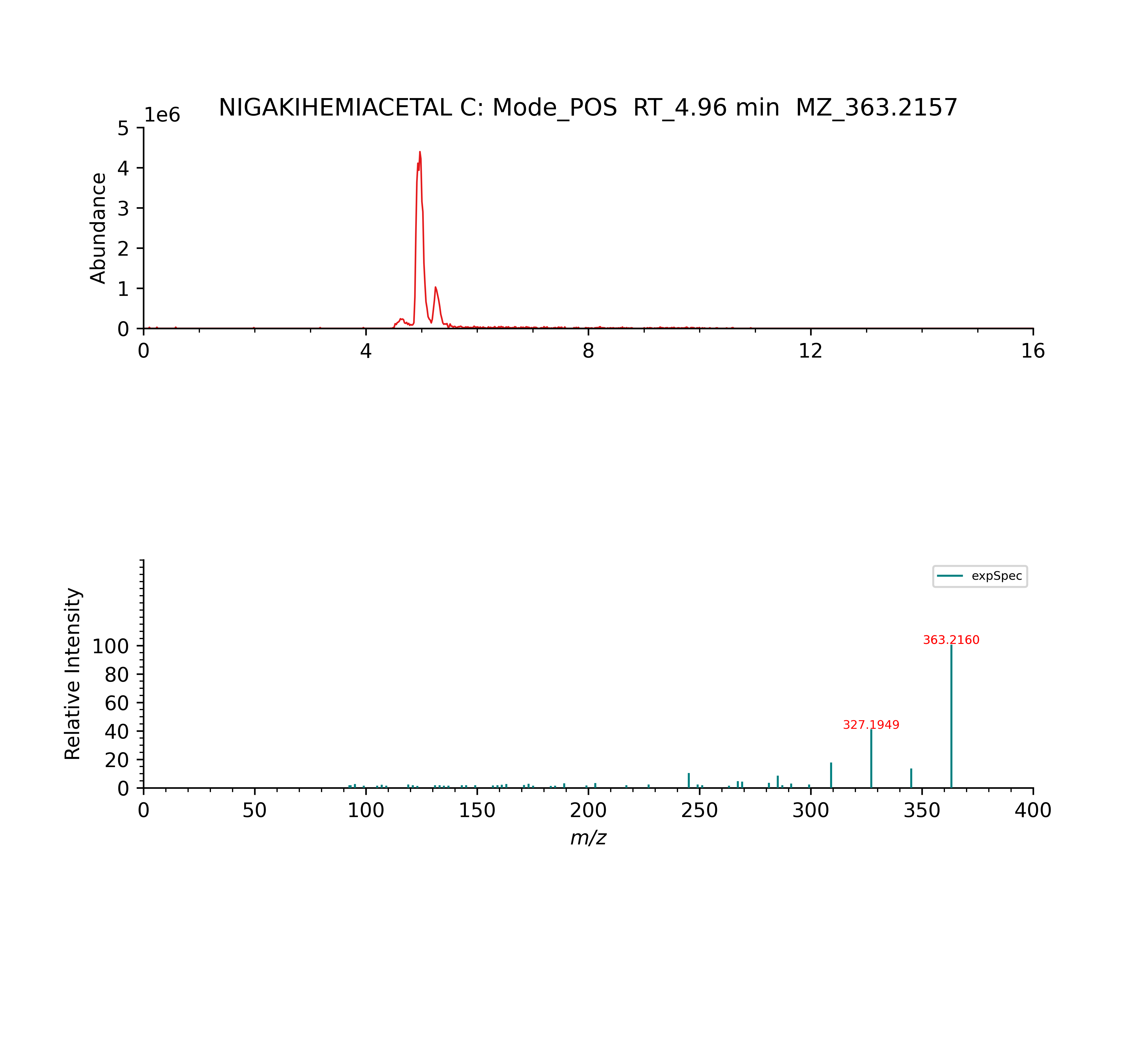

Supplement: Supplementary file 1 [file molecules-29-02840-s001.zip › Supplementary Figure s1/Identification from HerbDB datebase/png/compound00243.png]

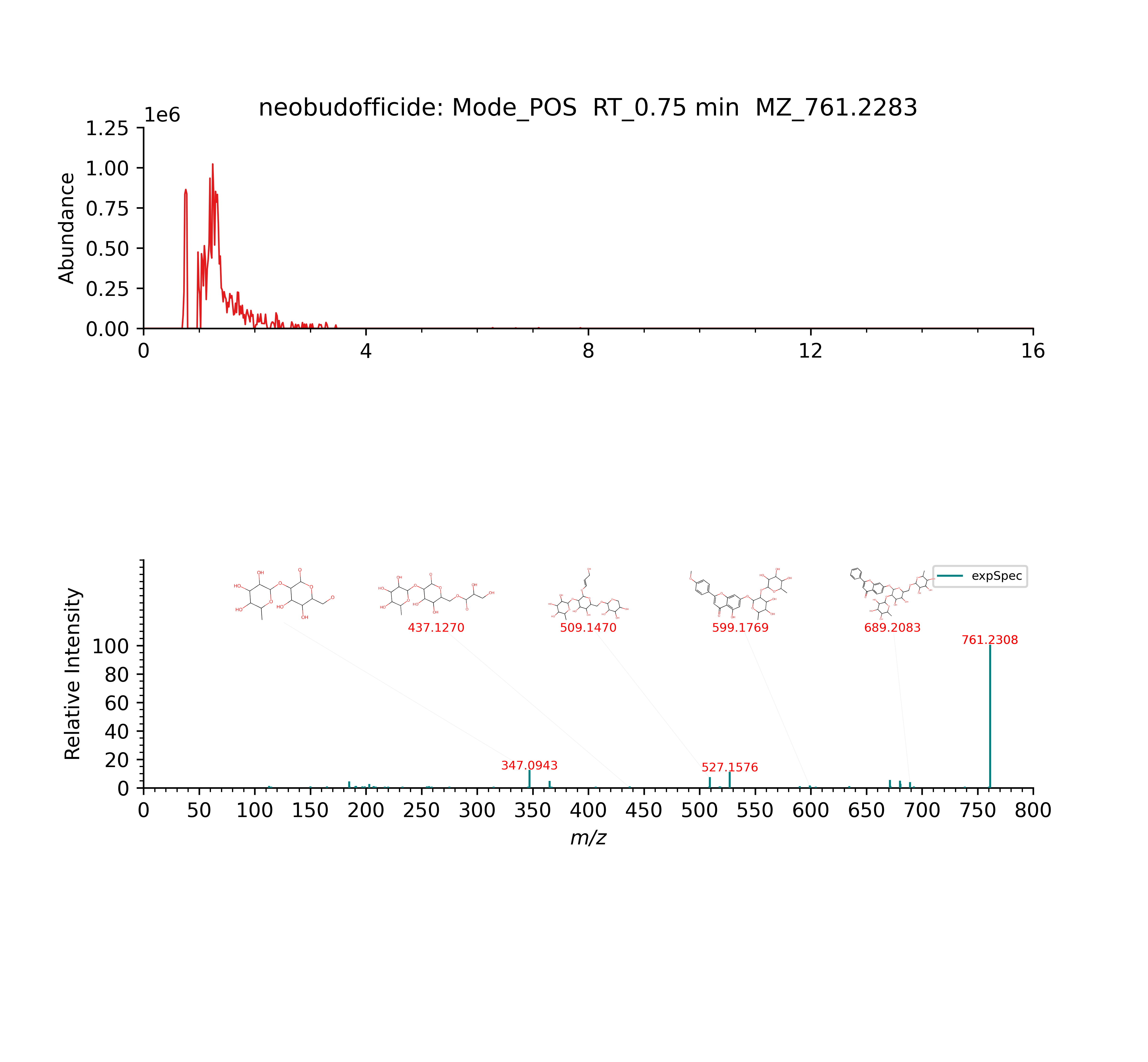

Supplement: Supplementary file 1 [file molecules-29-02840-s001.zip › Supplementary Figure s1/Identification from HerbDB datebase/png/compound00245.png]

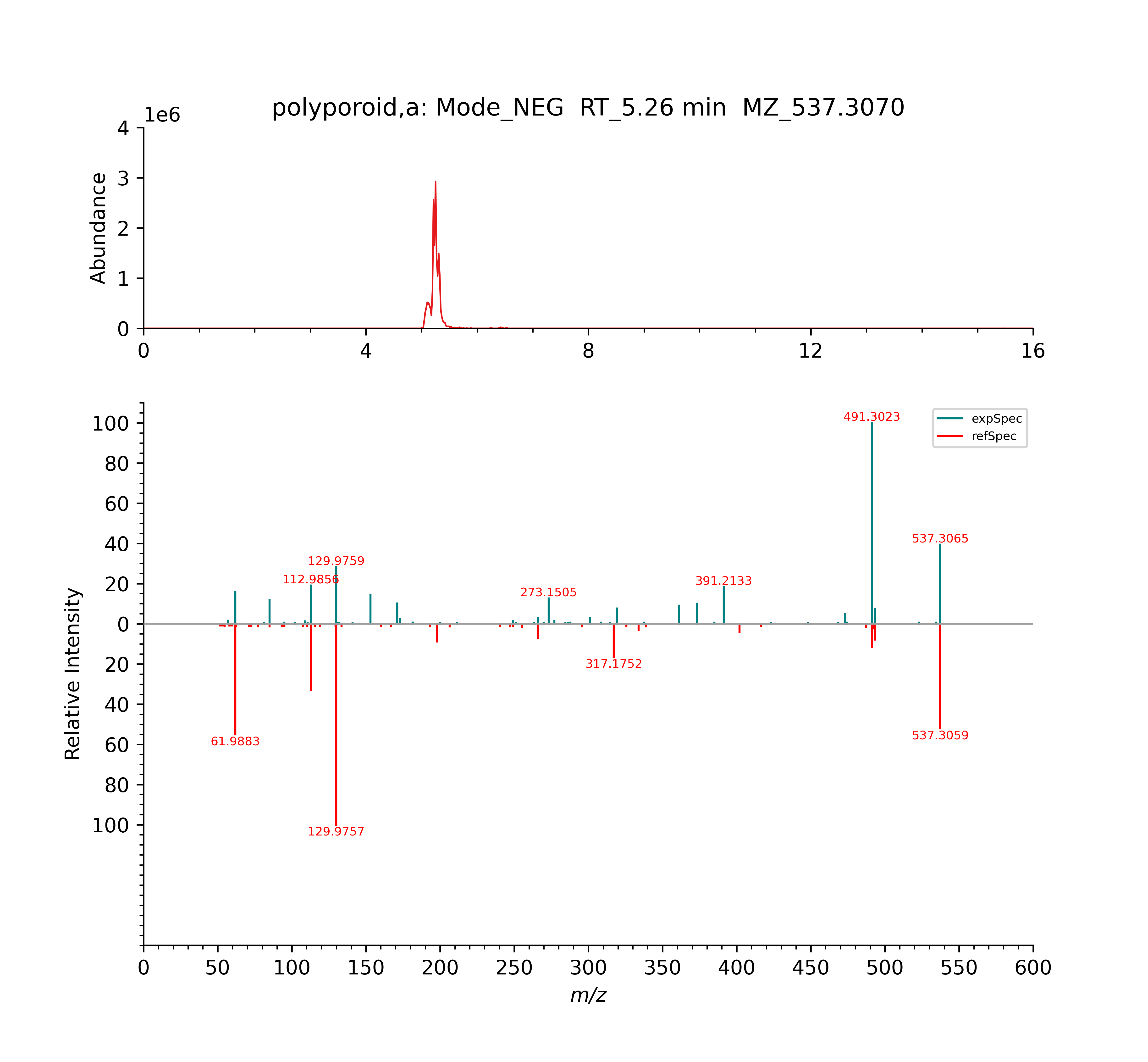

Supplement: Supplementary file 1 [file molecules-29-02840-s001.zip › Supplementary Figure s1/Identification from HerbDB datebase/png/compound00248.png]

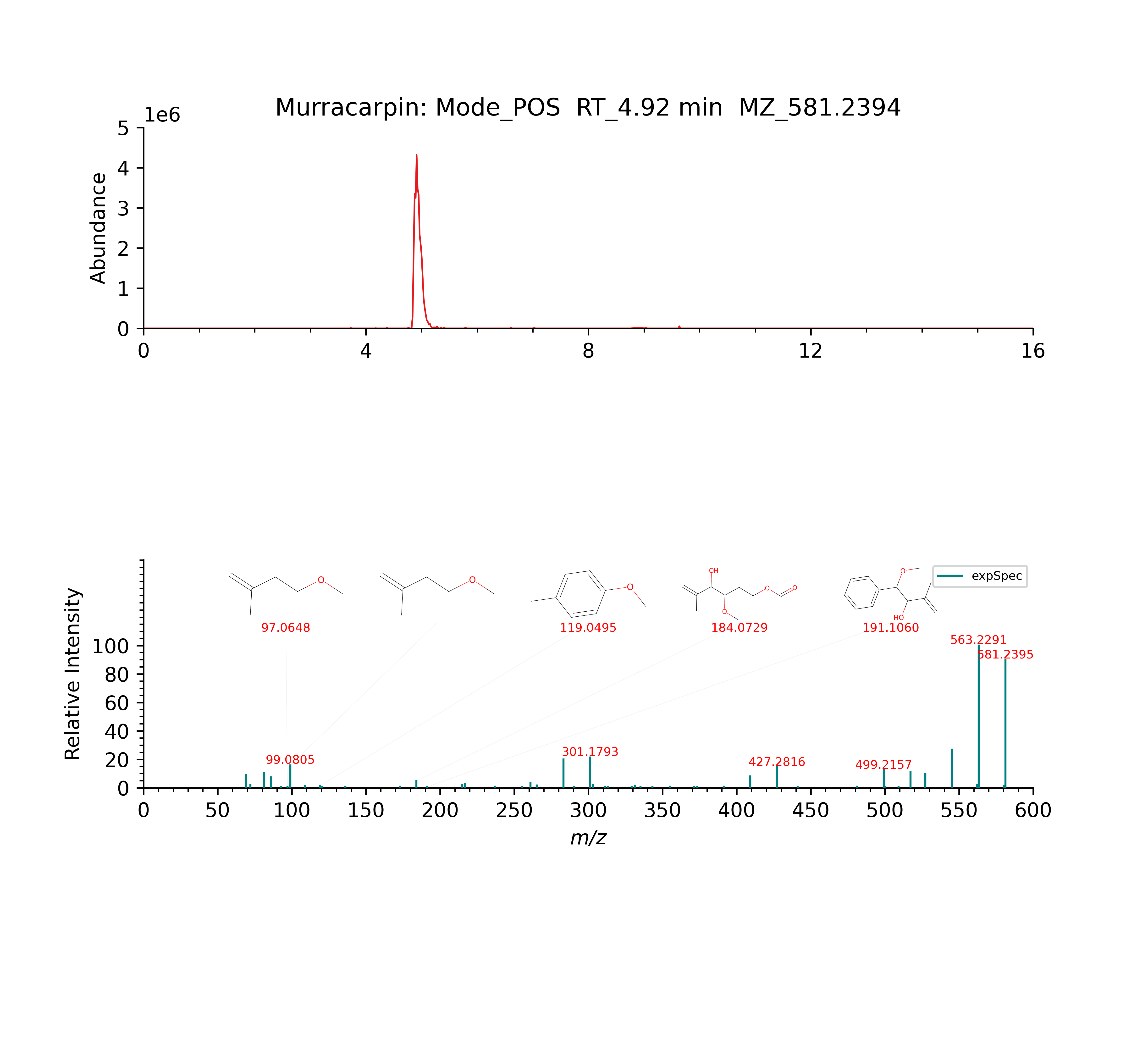

Supplement: Supplementary file 1 [file molecules-29-02840-s001.zip › Supplementary Figure s1/Identification from HerbDB datebase/png/compound00250.png]

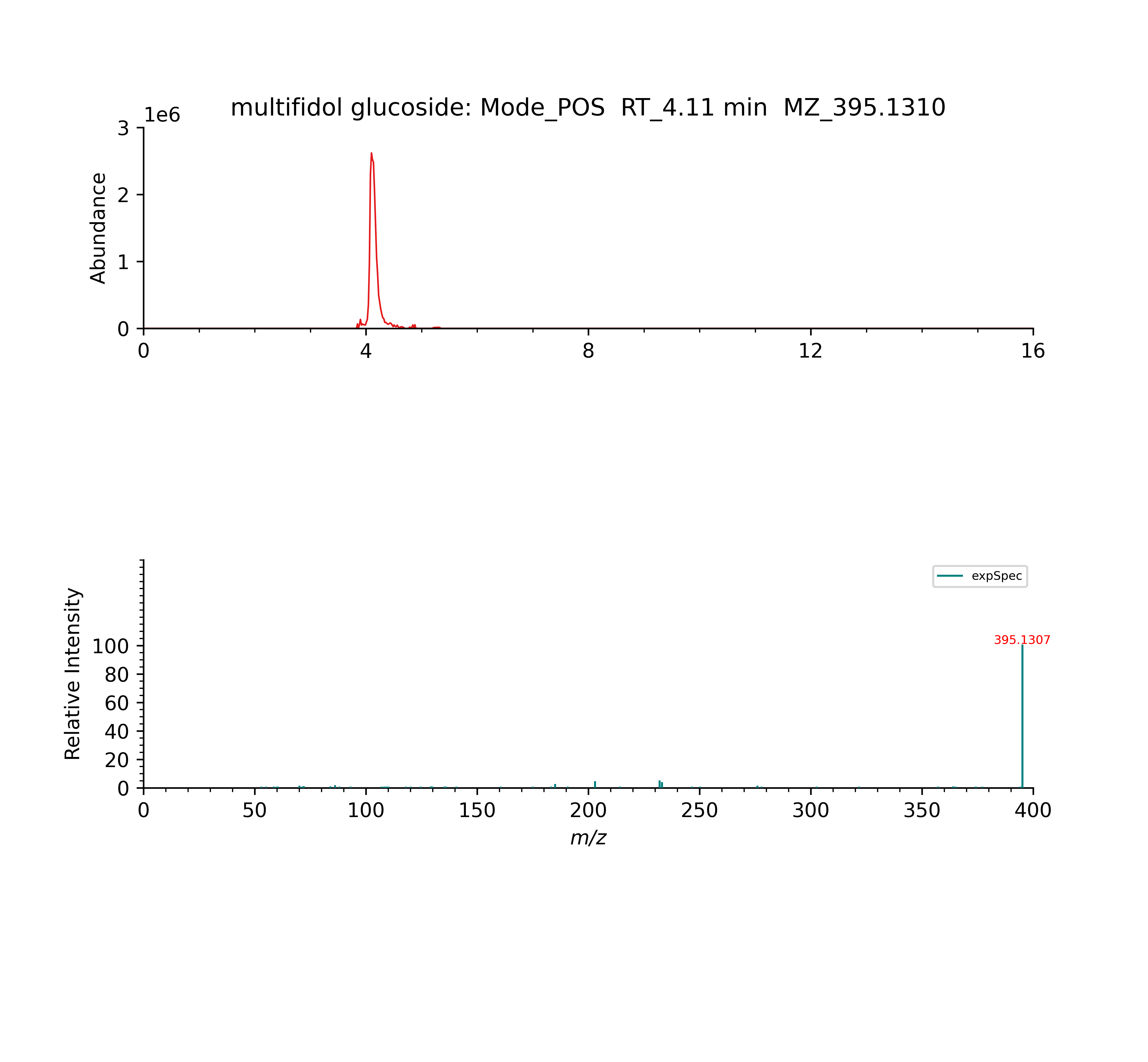

Supplement: Supplementary file 1 [file molecules-29-02840-s001.zip › Supplementary Figure s1/Identification from HerbDB datebase/png/compound00251.png]

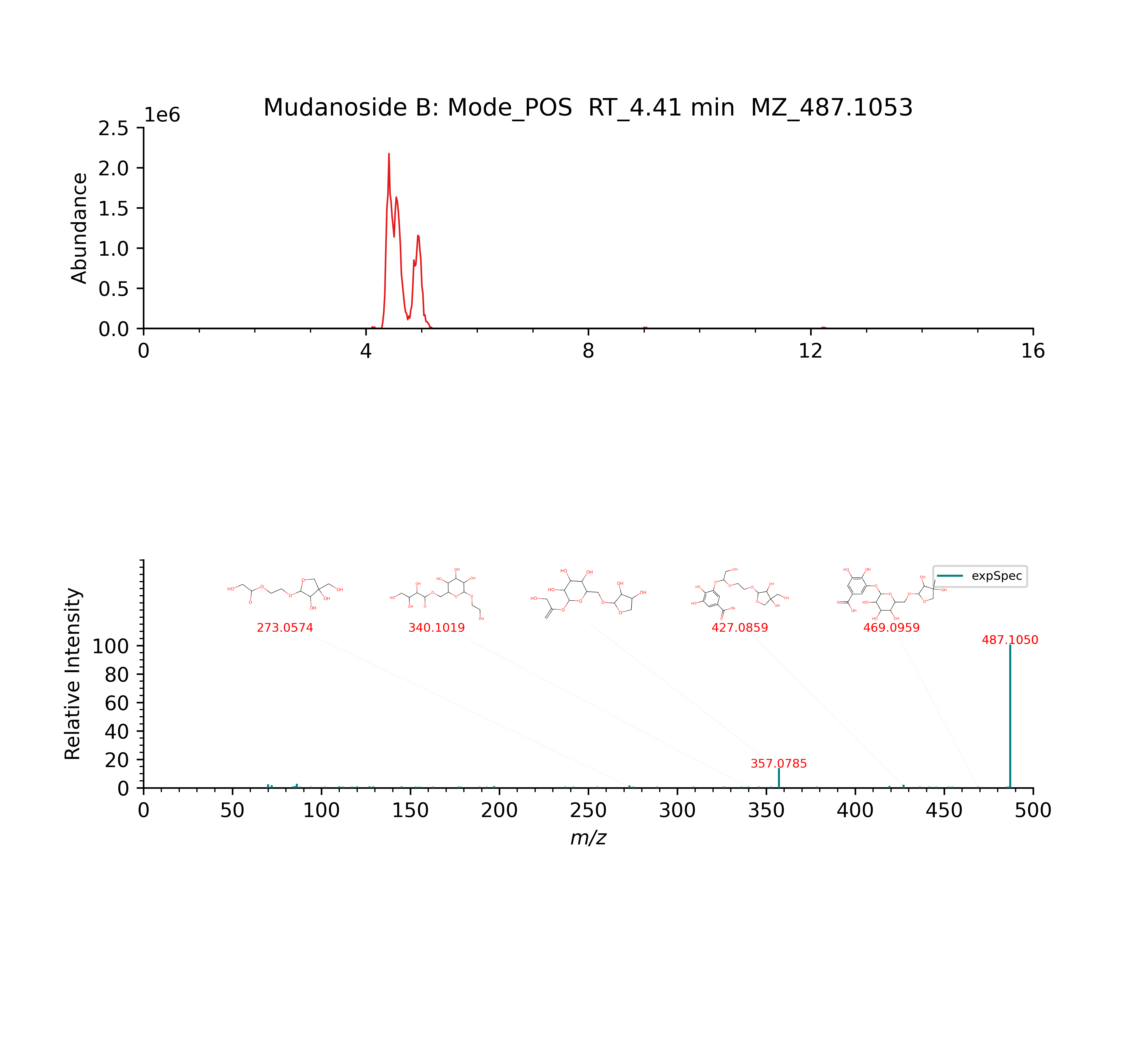

Supplement: Supplementary file 1 [file molecules-29-02840-s001.zip › Supplementary Figure s1/Identification from HerbDB datebase/png/compound00252.png]

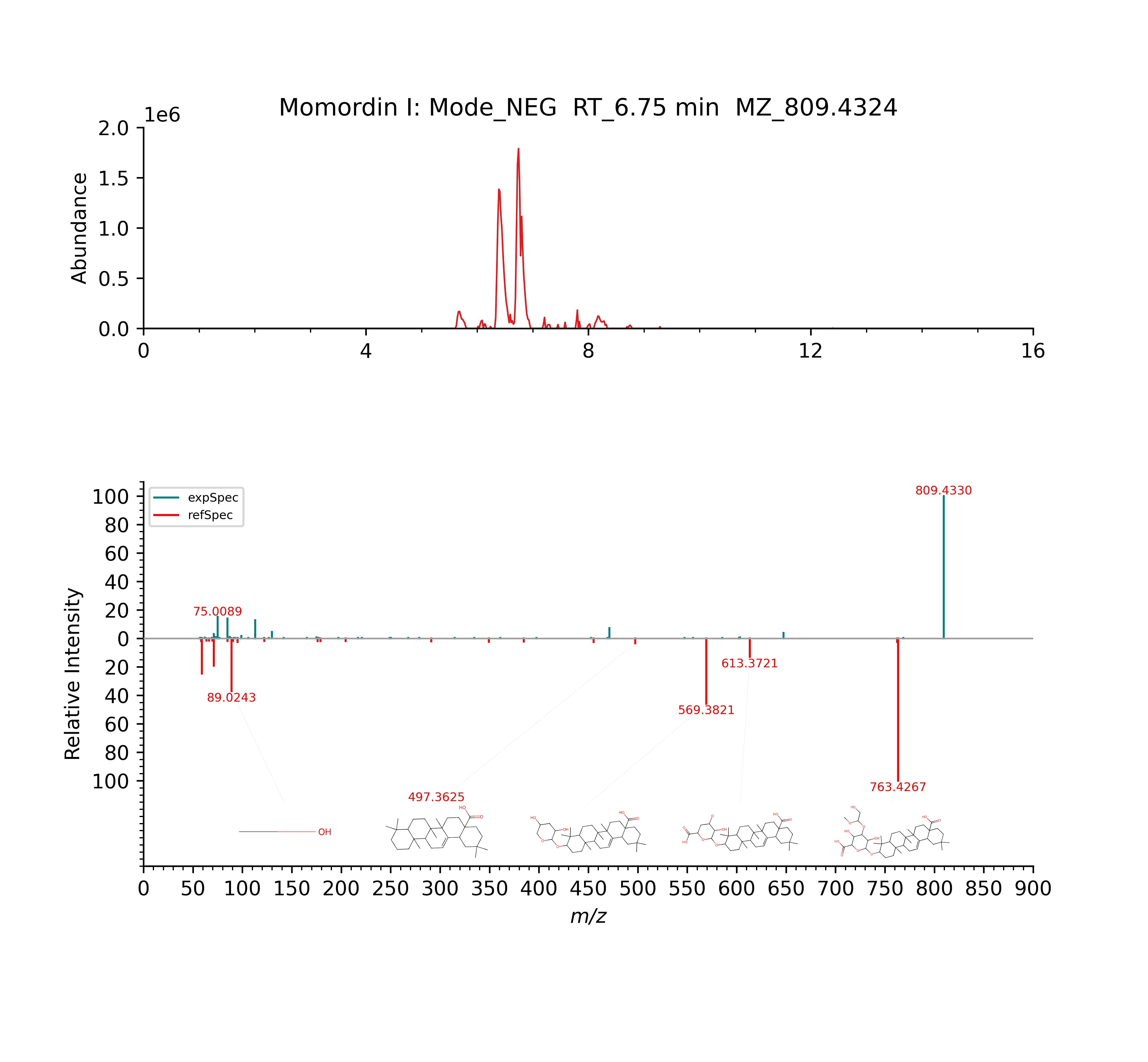

Supplement: Supplementary file 1 [file molecules-29-02840-s001.zip › Supplementary Figure s1/Identification from HerbDB datebase/png/compound00254.png]

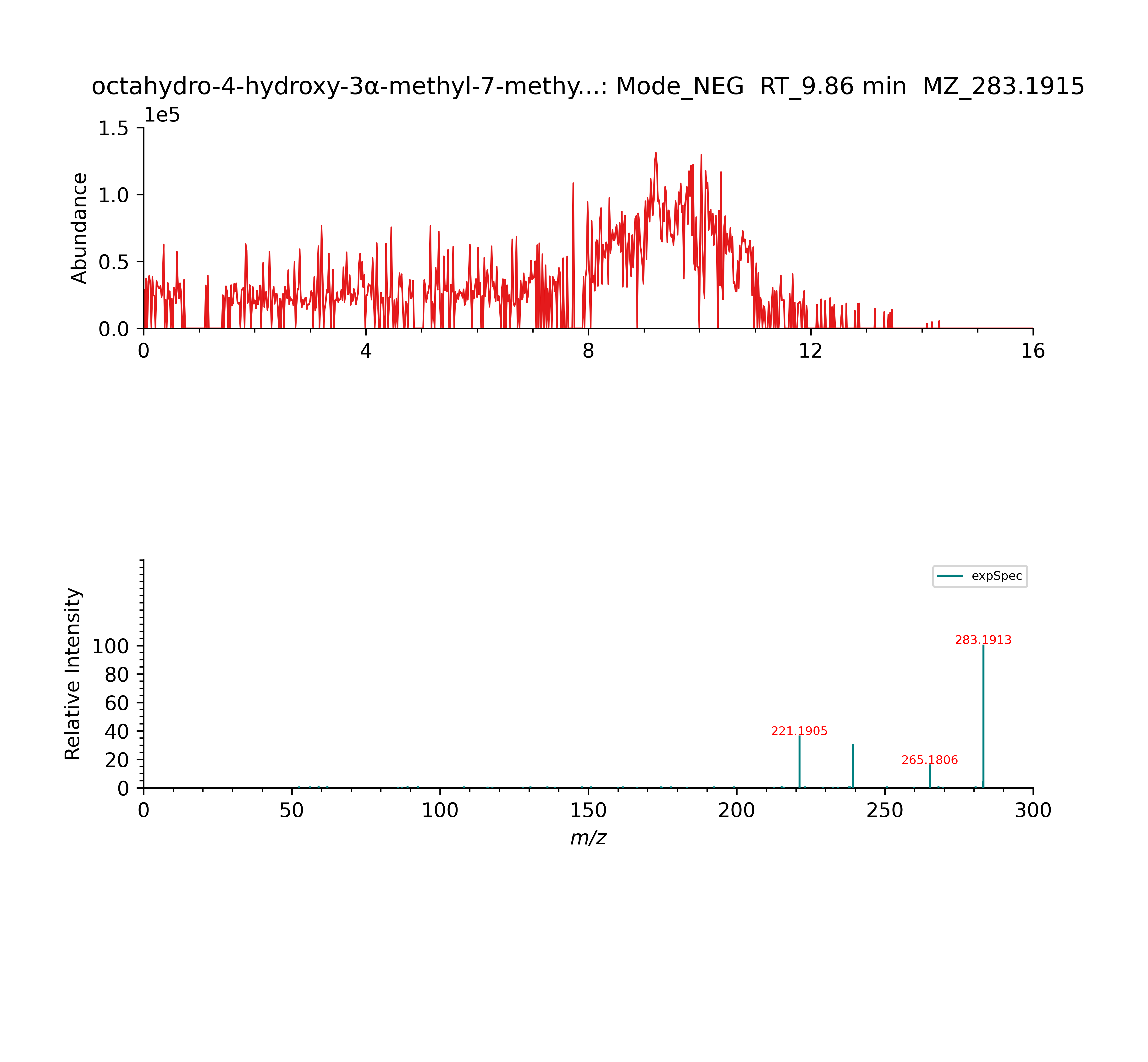

Supplement: Supplementary file 1 [file molecules-29-02840-s001.zip › Supplementary Figure s1/Identification from HerbDB datebase/png/compound00255.png]

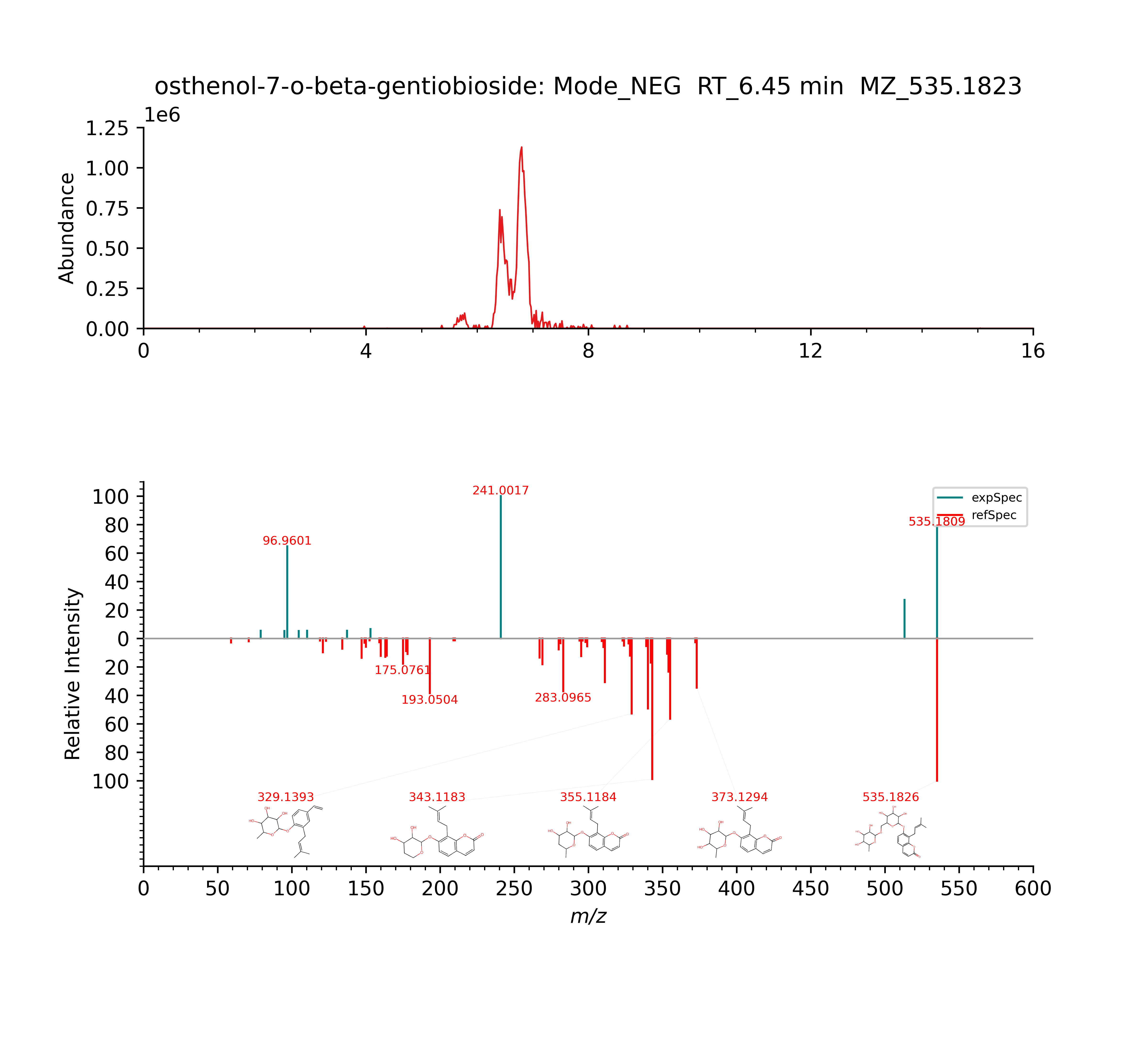

Supplement: Supplementary file 1 [file molecules-29-02840-s001.zip › Supplementary Figure s1/Identification from HerbDB datebase/png/compound00258.png]

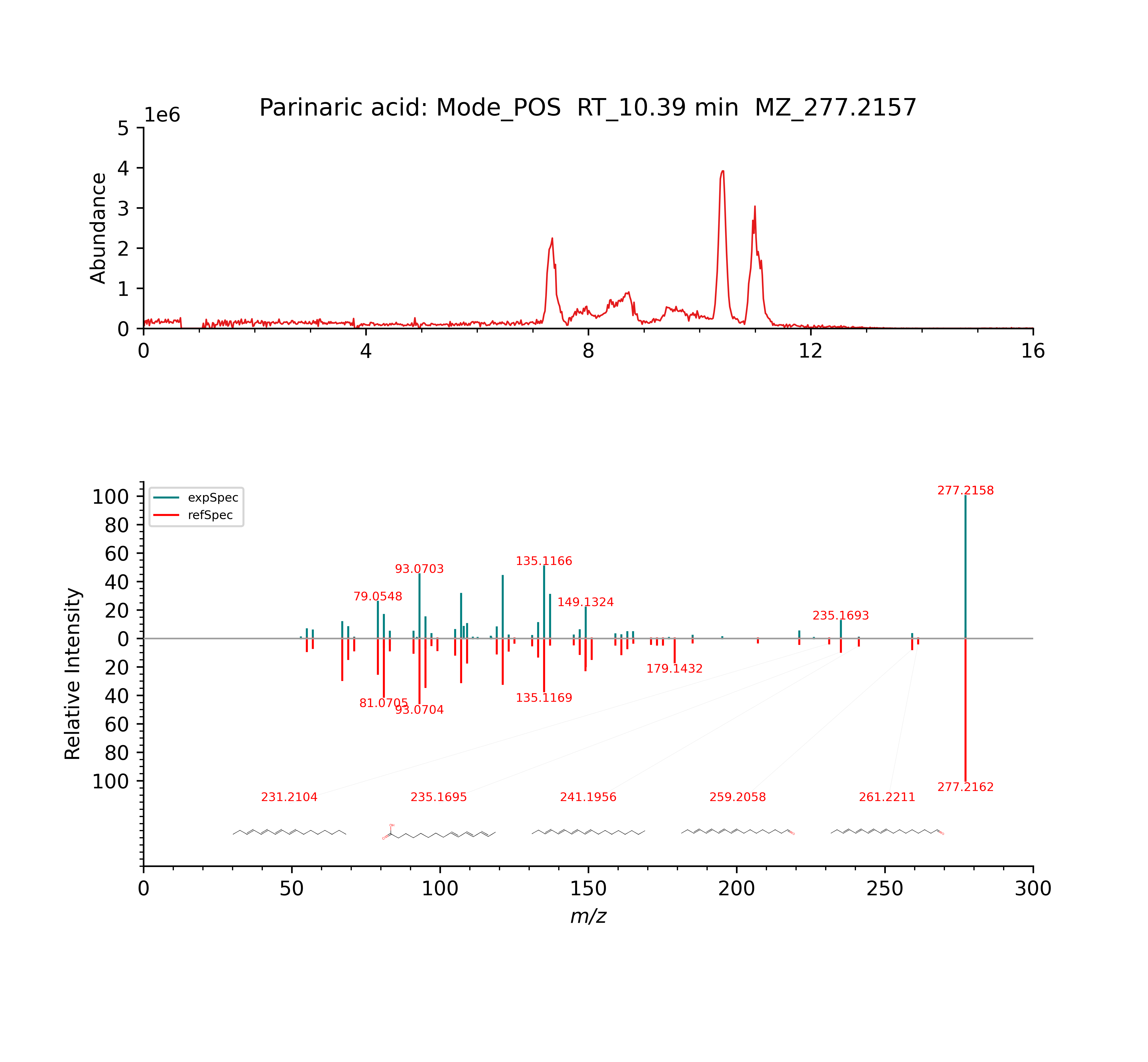

Supplement: Supplementary file 1 [file molecules-29-02840-s001.zip › Supplementary Figure s1/Identification from HerbDB datebase/png/compound00262.png]

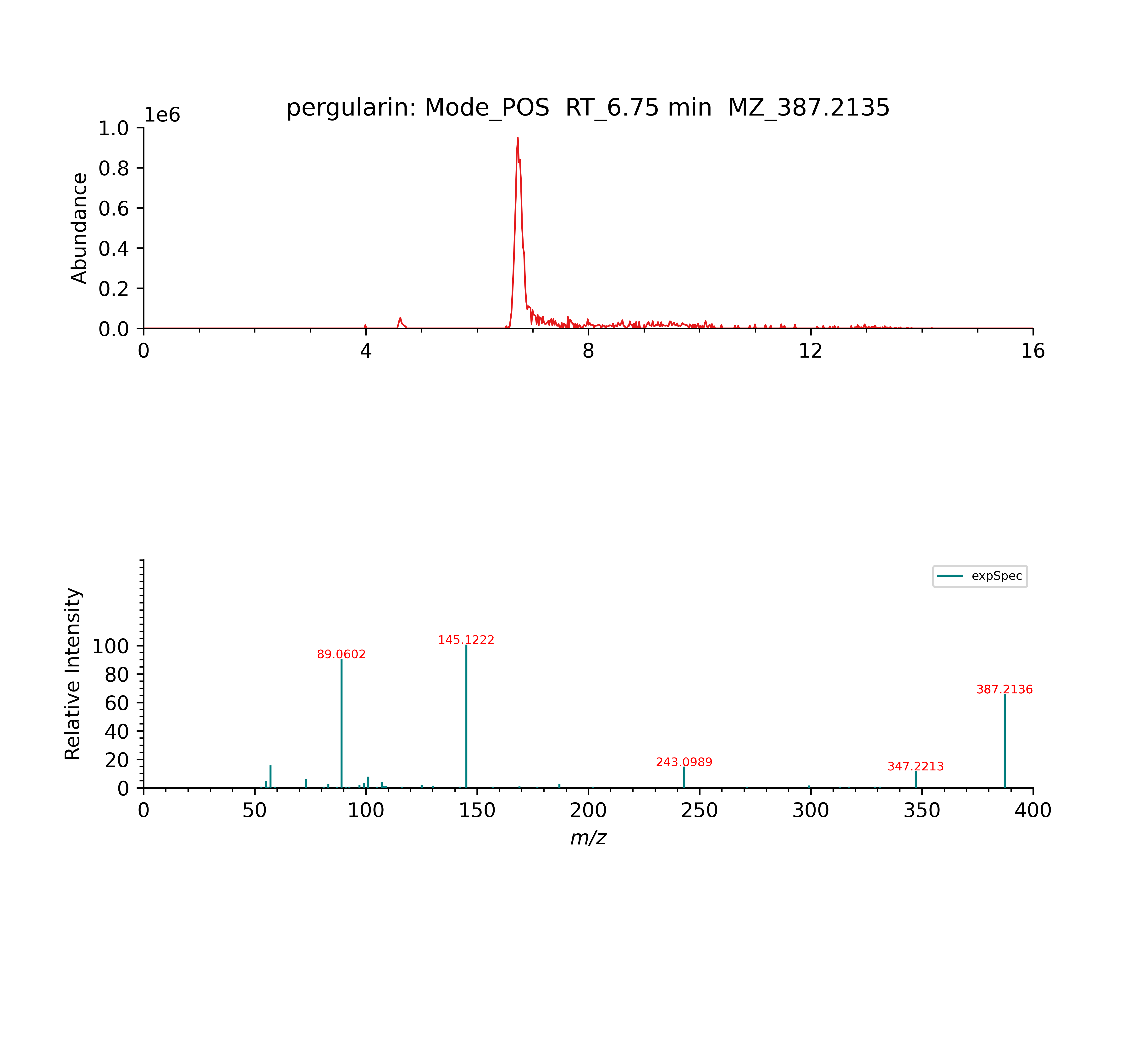

Supplement: Supplementary file 1 [file molecules-29-02840-s001.zip › Supplementary Figure s1/Identification from HerbDB datebase/png/compound00265.png]

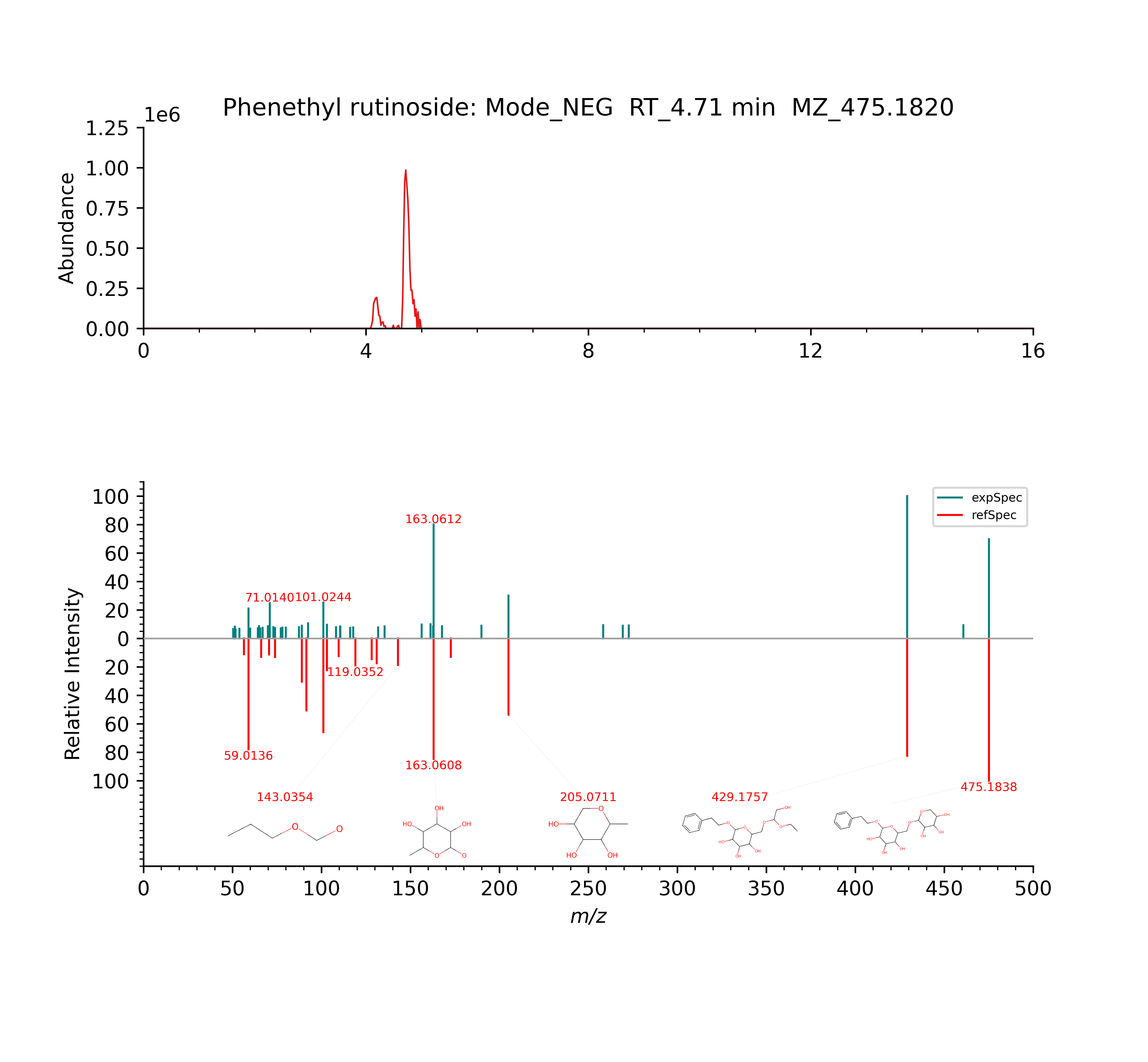

Supplement: Supplementary file 1 [file molecules-29-02840-s001.zip › Supplementary Figure s1/Identification from HerbDB datebase/png/compound00266.png]

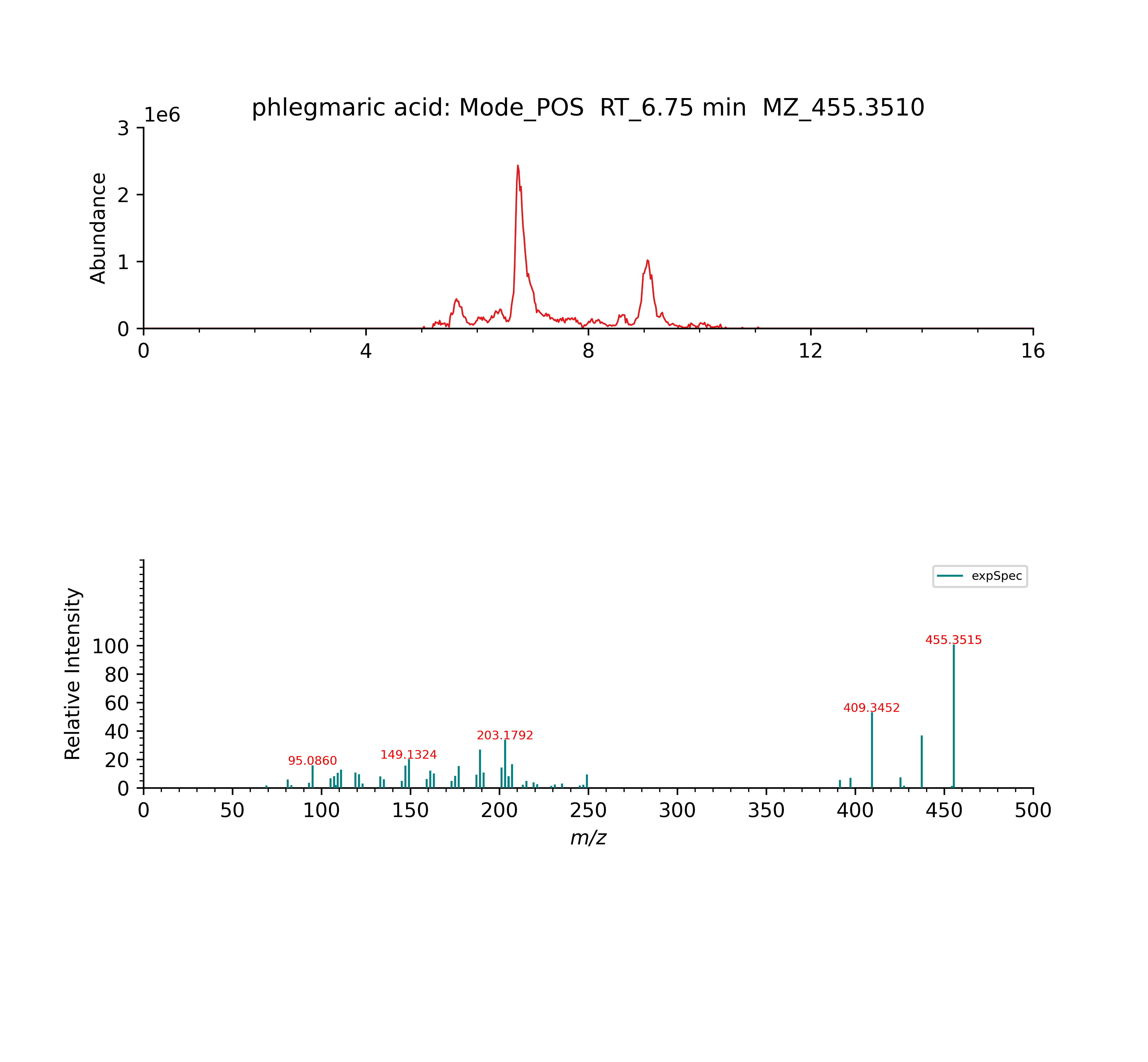

Supplement: Supplementary file 1 [file molecules-29-02840-s001.zip › Supplementary Figure s1/Identification from HerbDB datebase/png/compound00267.png]

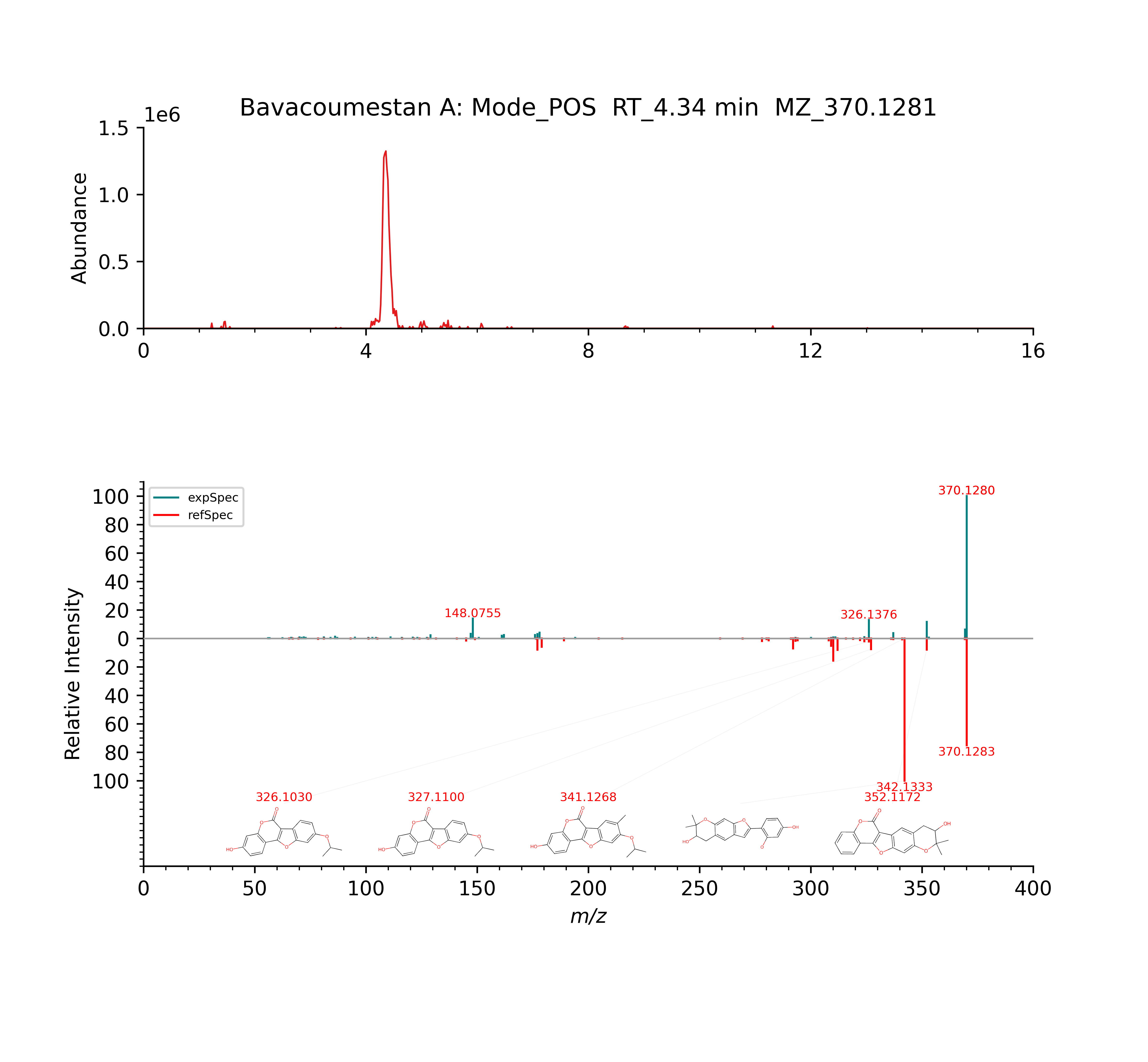

Supplement: Supplementary file 1 [file molecules-29-02840-s001.zip › Supplementary Figure s1/Identification from HerbDB datebase/png/compound00268.png]

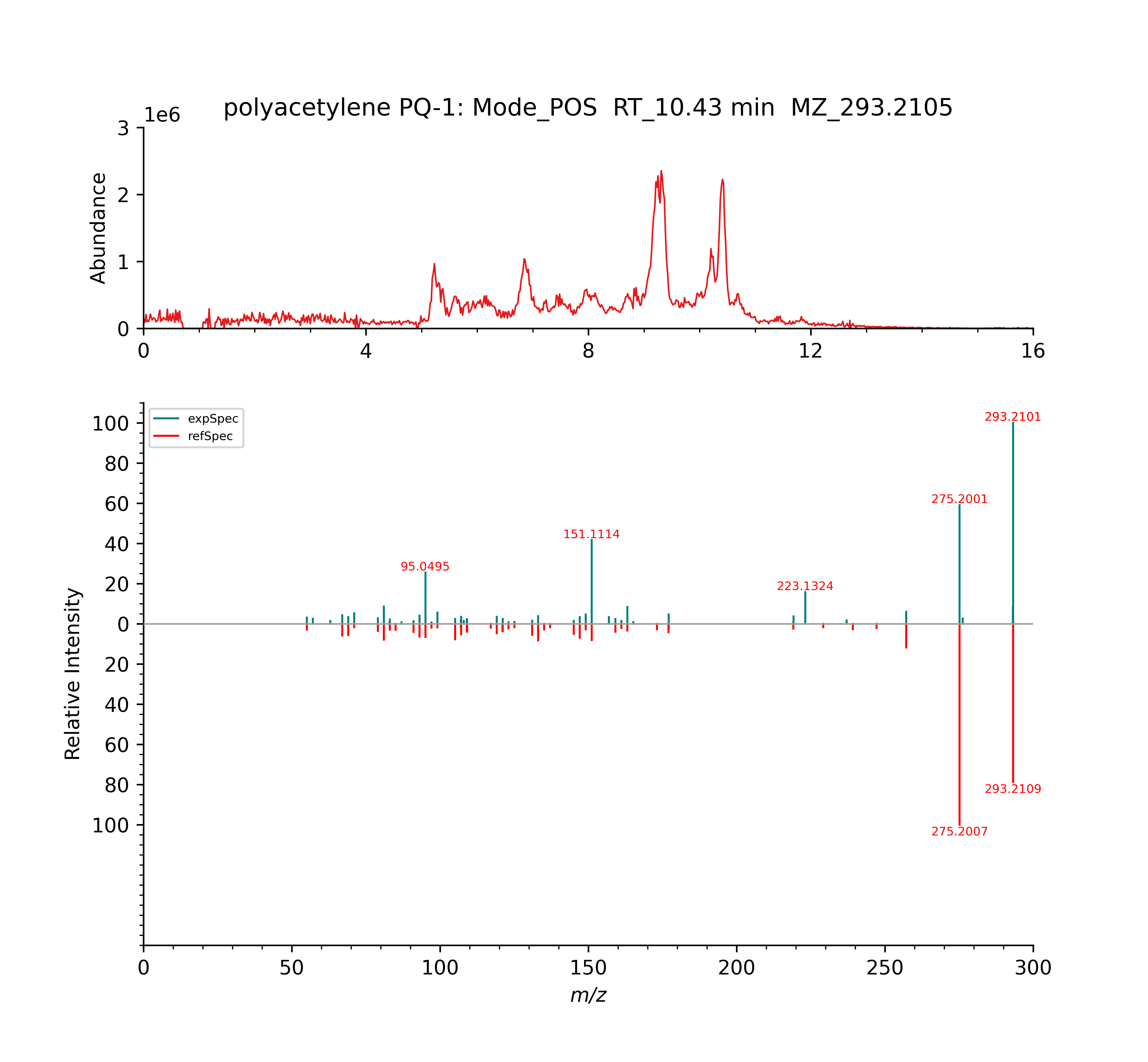

Supplement: Supplementary file 1 [file molecules-29-02840-s001.zip › Supplementary Figure s1/Identification from HerbDB datebase/png/compound00271.png]

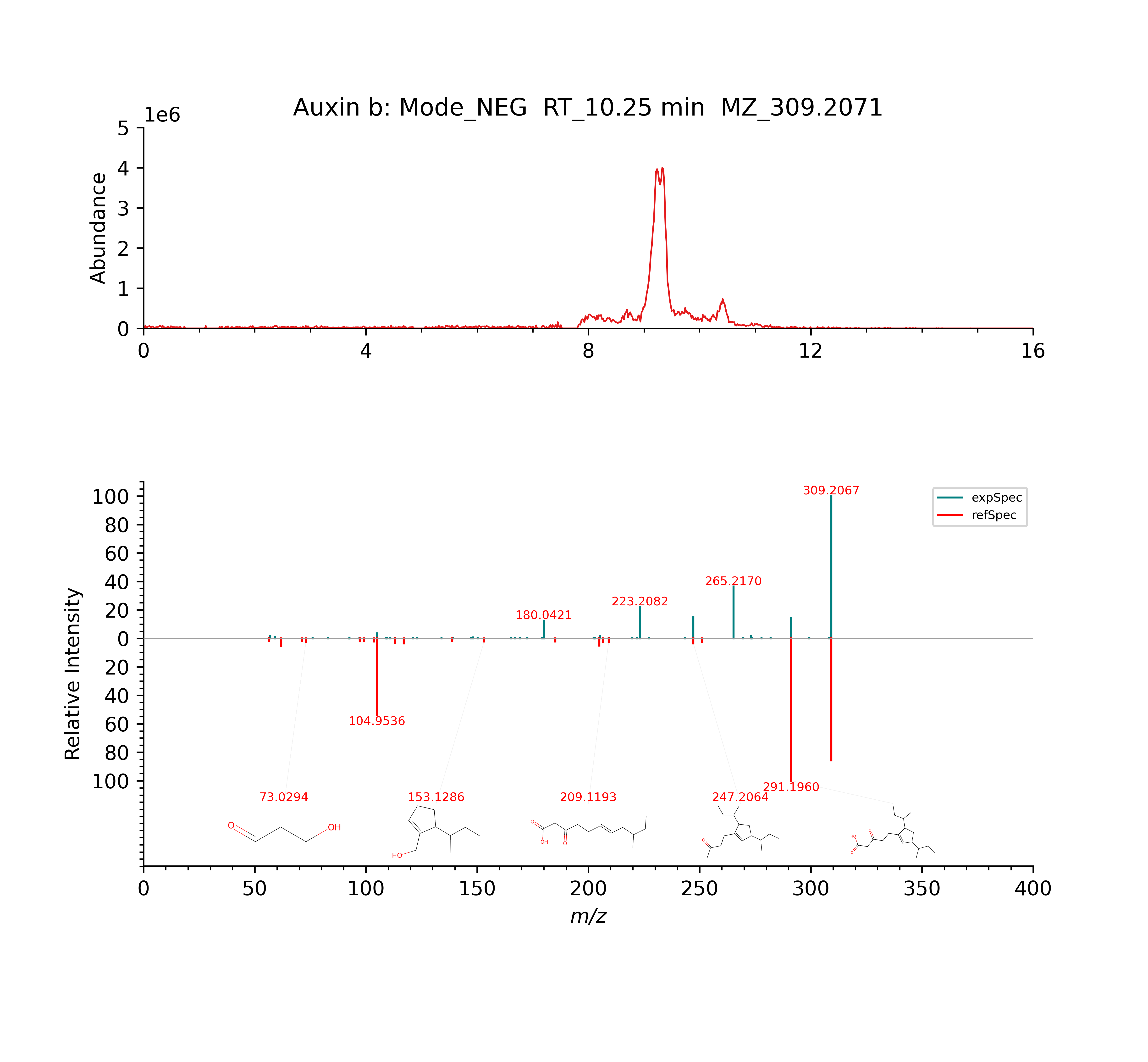

Supplement: Supplementary file 1 [file molecules-29-02840-s001.zip › Supplementary Figure s1/Identification from HerbDB datebase/png/compound00275.png]

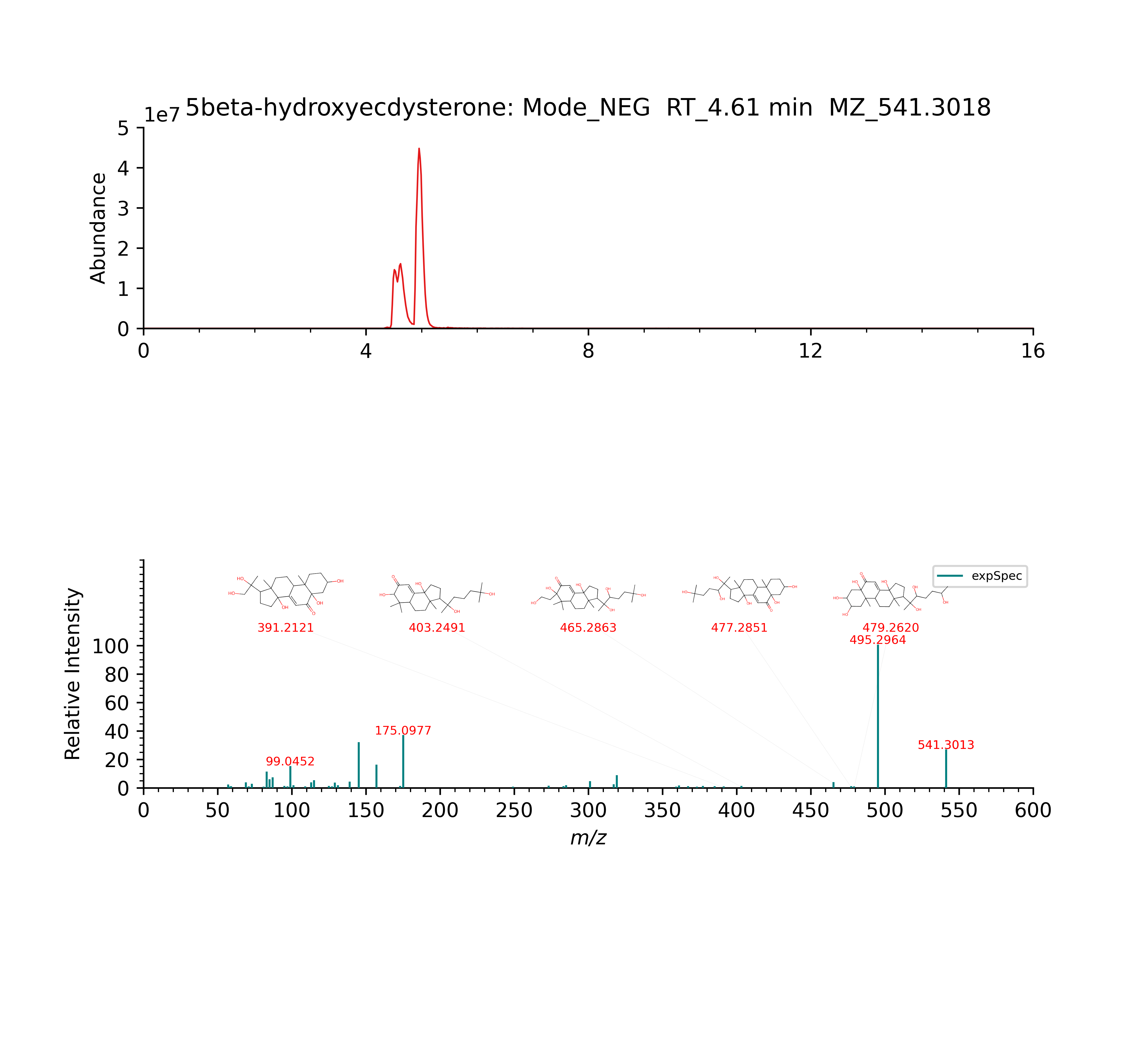

Supplement: Supplementary file 1 [file molecules-29-02840-s001.zip › Supplementary Figure s1/Identification from HerbDB datebase/png/compound00277.png]

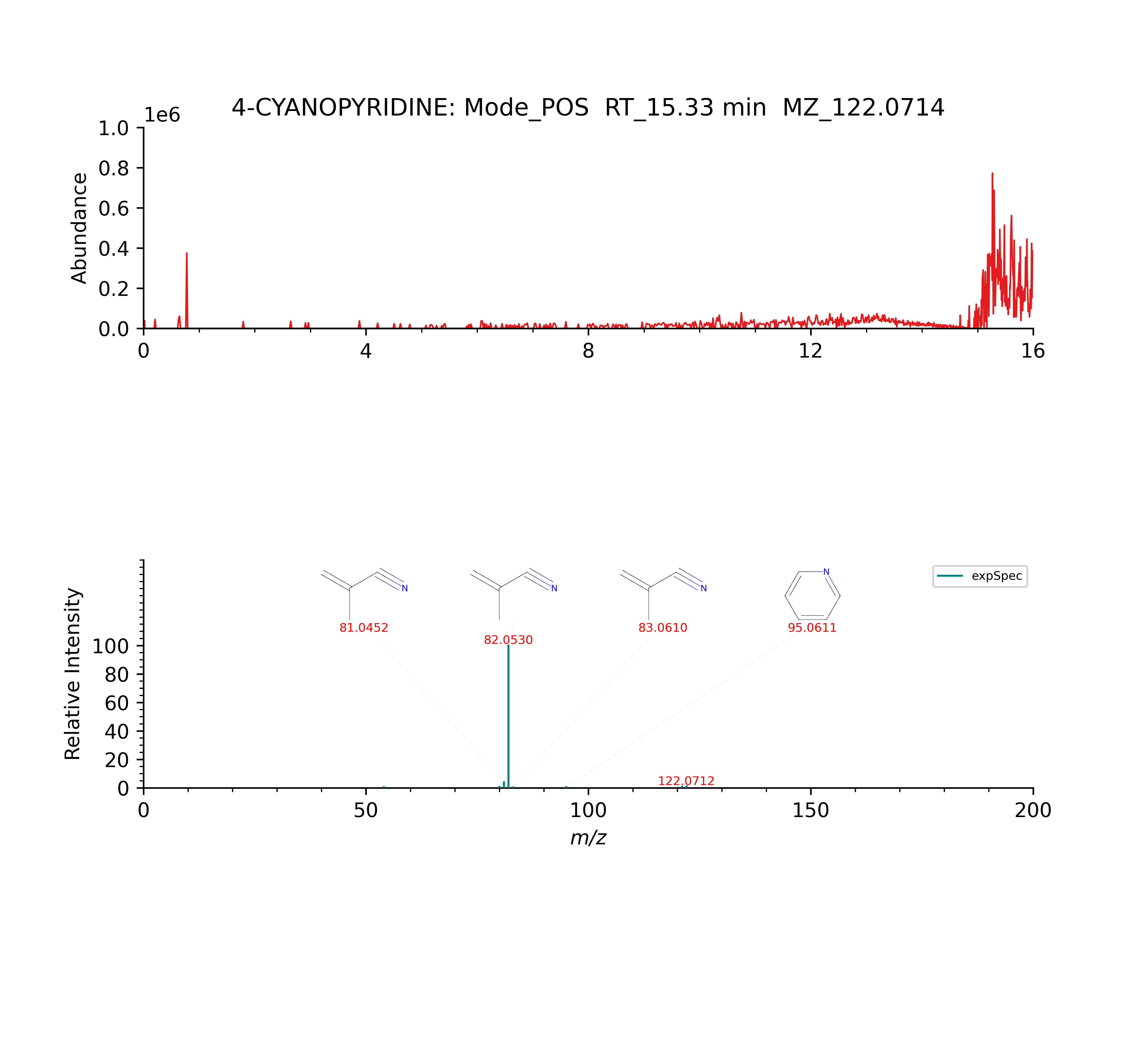

Supplement: Supplementary file 1 [file molecules-29-02840-s001.zip › Supplementary Figure s1/Identification from HerbDB datebase/png/compound00281.png]

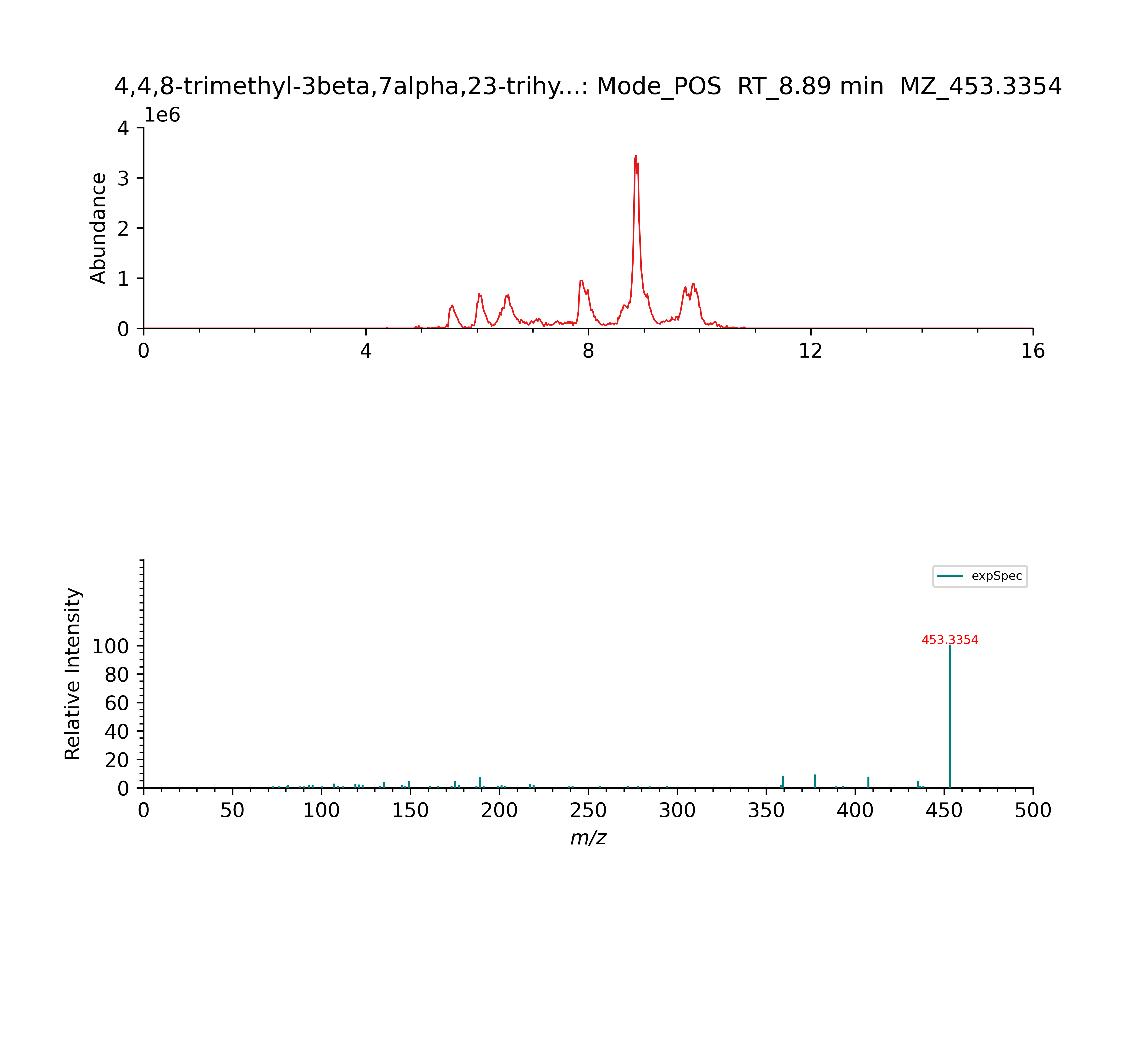

Supplement: Supplementary file 1 [file molecules-29-02840-s001.zip › Supplementary Figure s1/Identification from HerbDB datebase/png/compound00282.png]

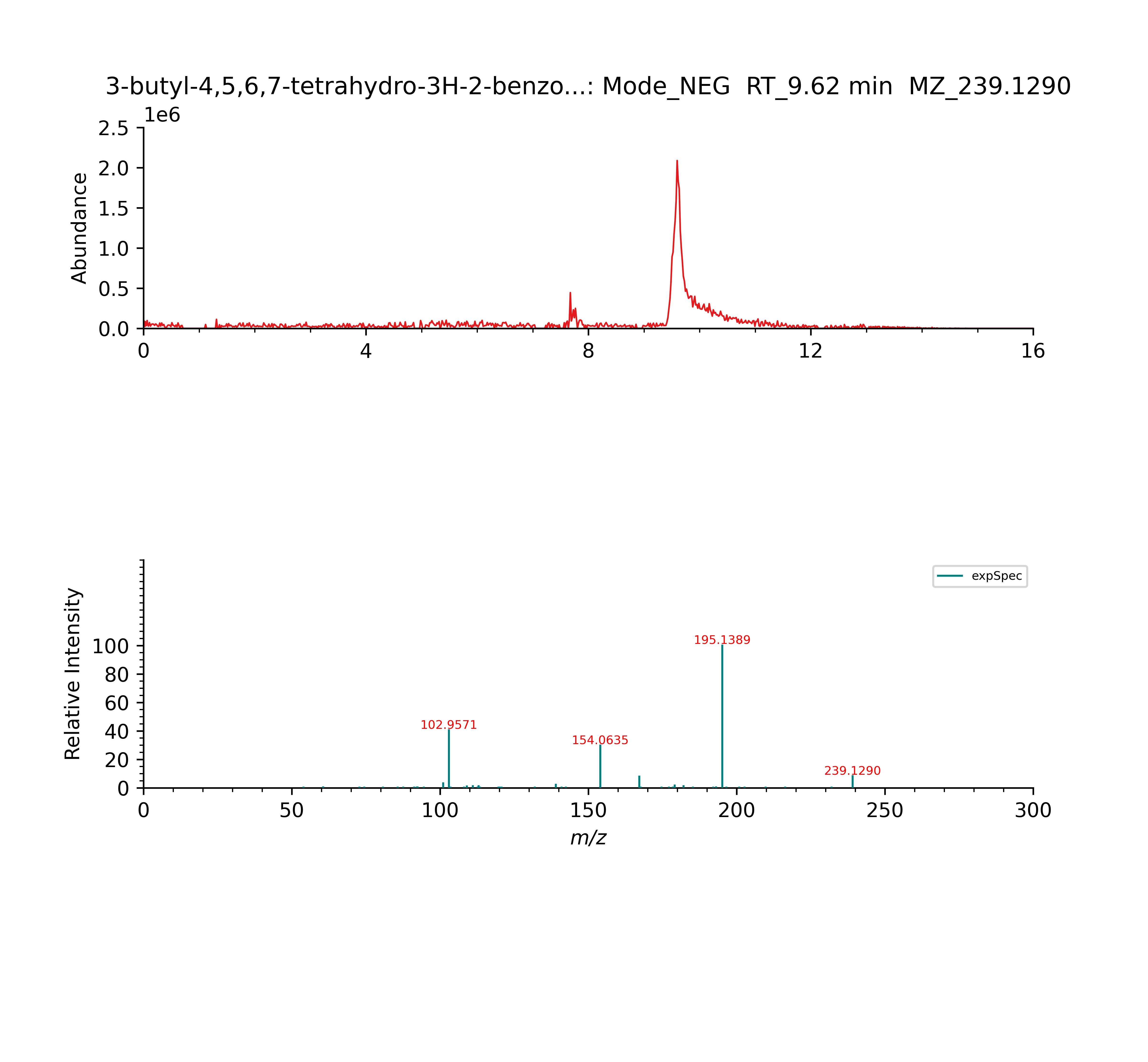

Supplement: Supplementary file 1 [file molecules-29-02840-s001.zip › Supplementary Figure s1/Identification from HerbDB datebase/png/compound00285.png]

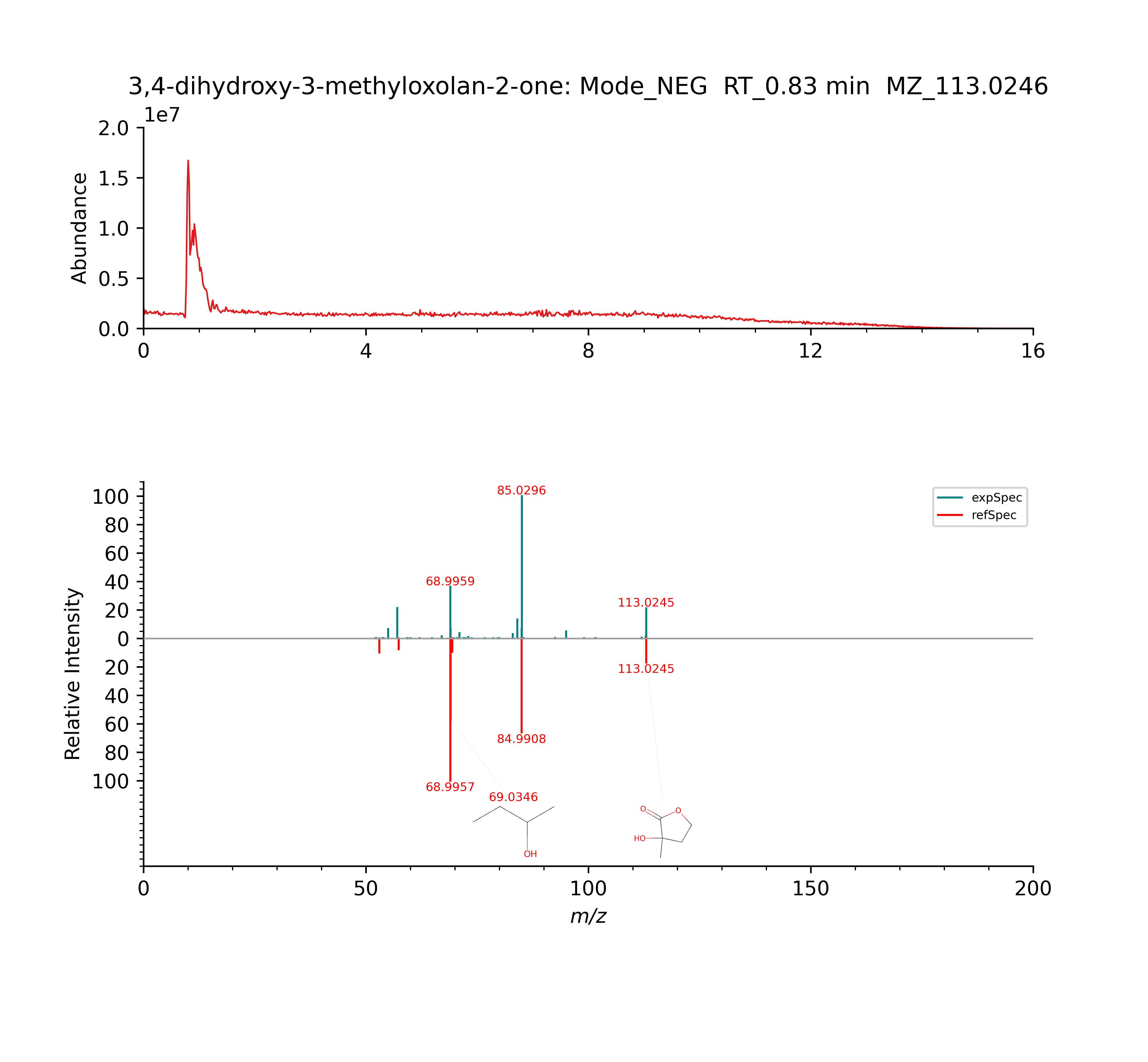

Supplement: Supplementary file 1 [file molecules-29-02840-s001.zip › Supplementary Figure s1/Identification from HerbDB datebase/png/compound00286.png]

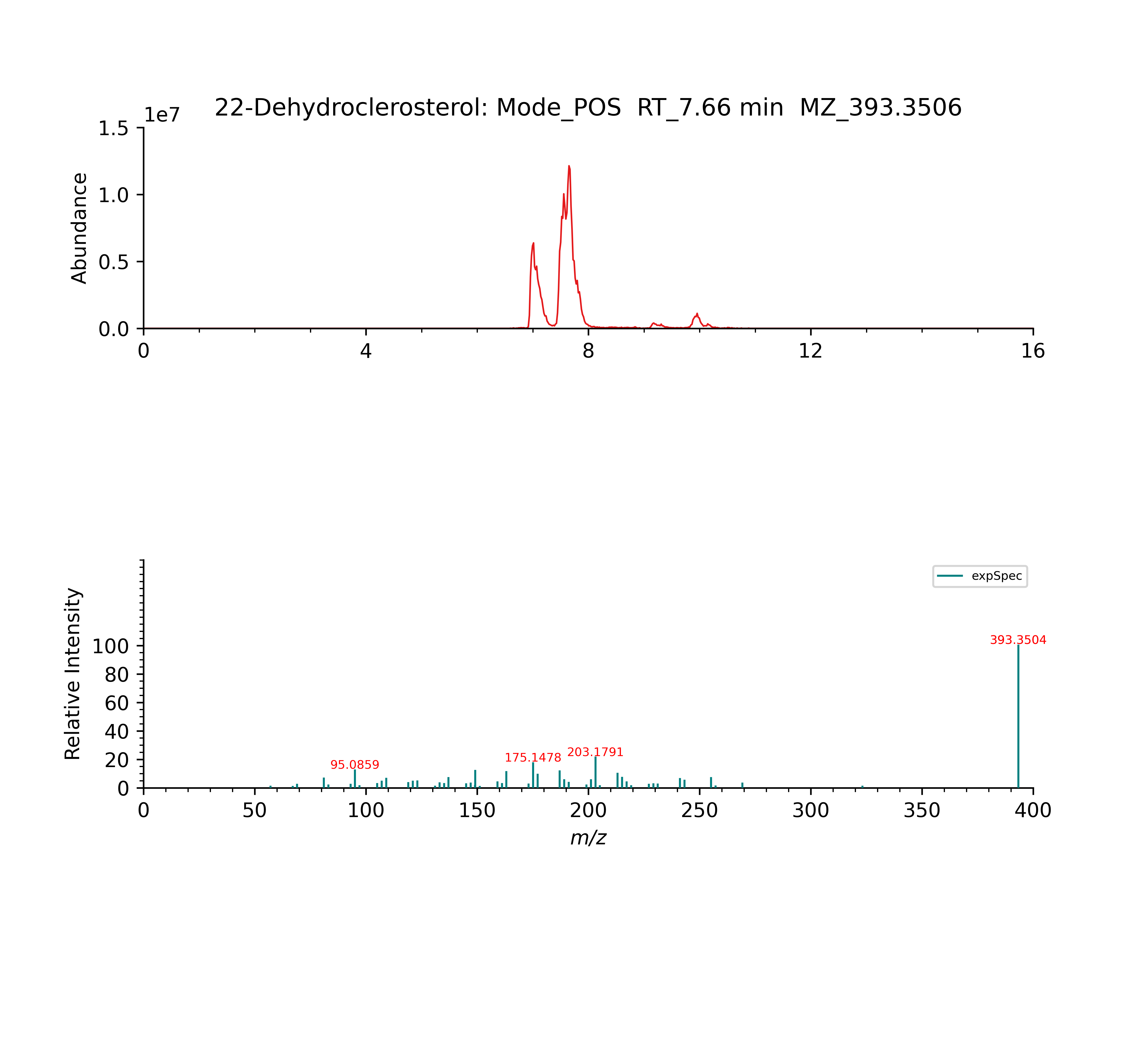

Supplement: Supplementary file 1 [file molecules-29-02840-s001.zip › Supplementary Figure s1/Identification from HerbDB datebase/png/compound00287.png]

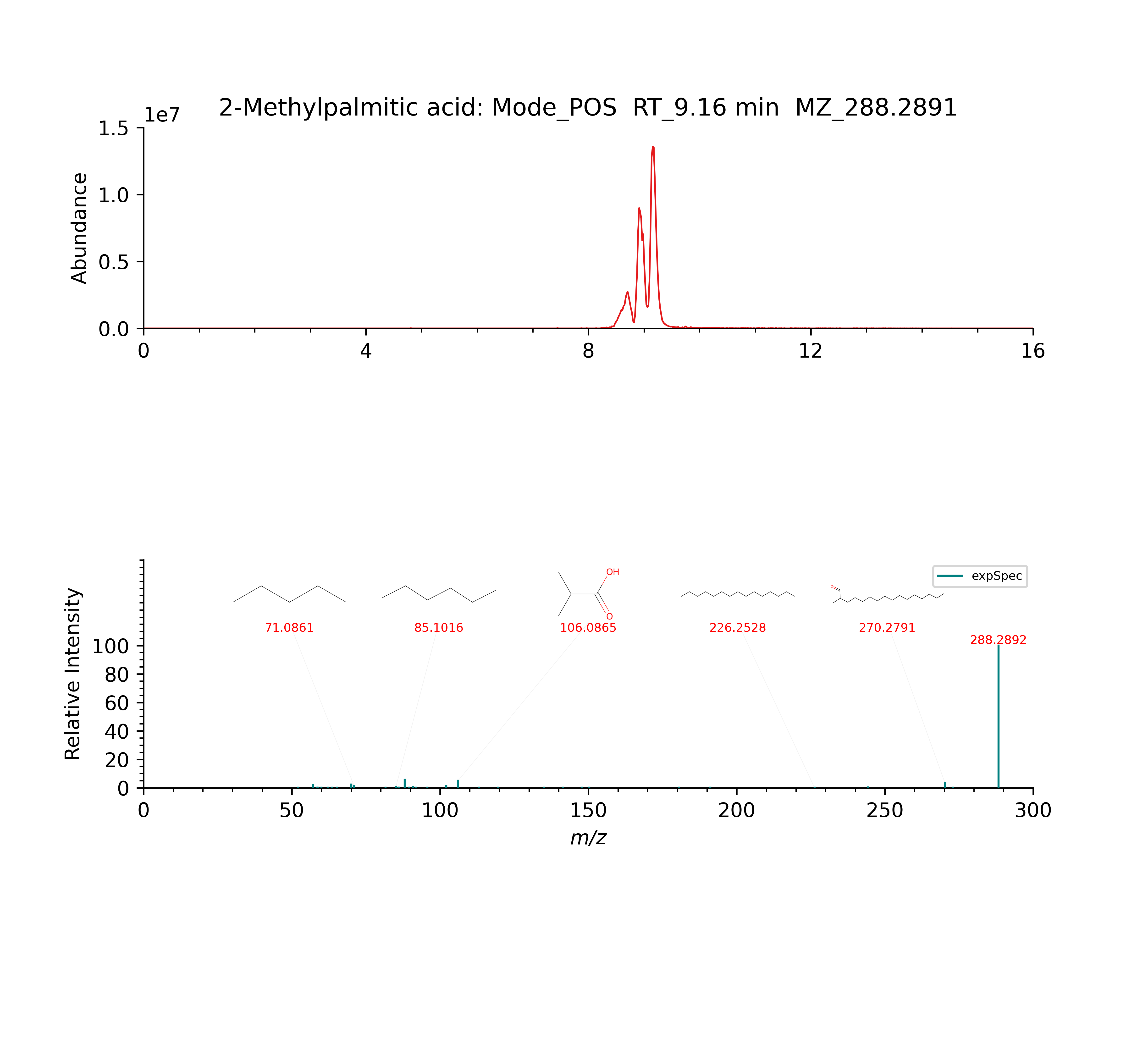

Supplement: Supplementary file 1 [file molecules-29-02840-s001.zip › Supplementary Figure s1/Identification from HerbDB datebase/png/compound00289.png]

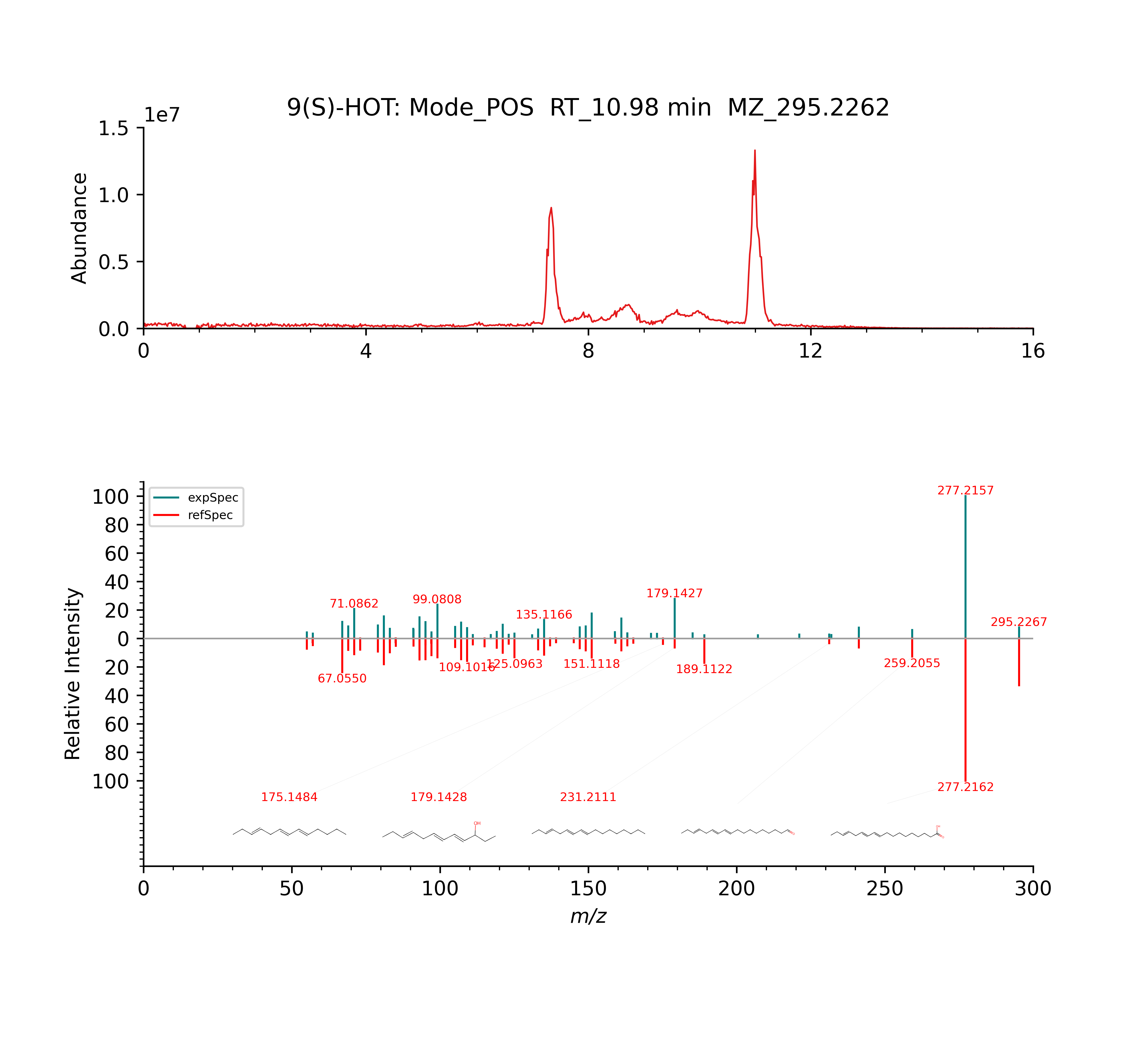

Supplement: Supplementary file 1 [file molecules-29-02840-s001.zip › Supplementary Figure s1/Identification from HerbDB datebase/png/compound00292.png]

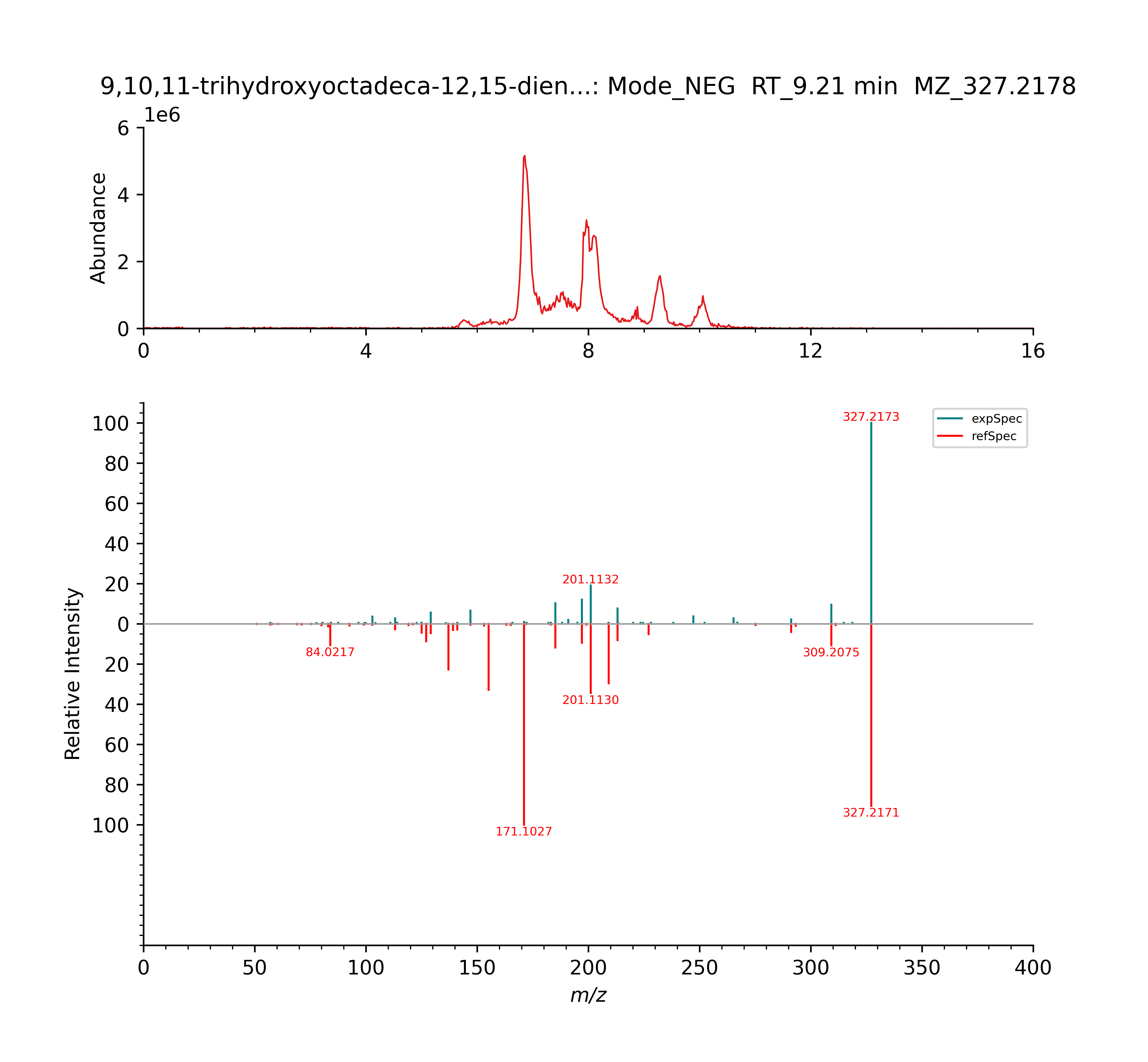

Supplement: Supplementary file 1 [file molecules-29-02840-s001.zip › Supplementary Figure s1/Identification from HerbDB datebase/png/compound00293.png]

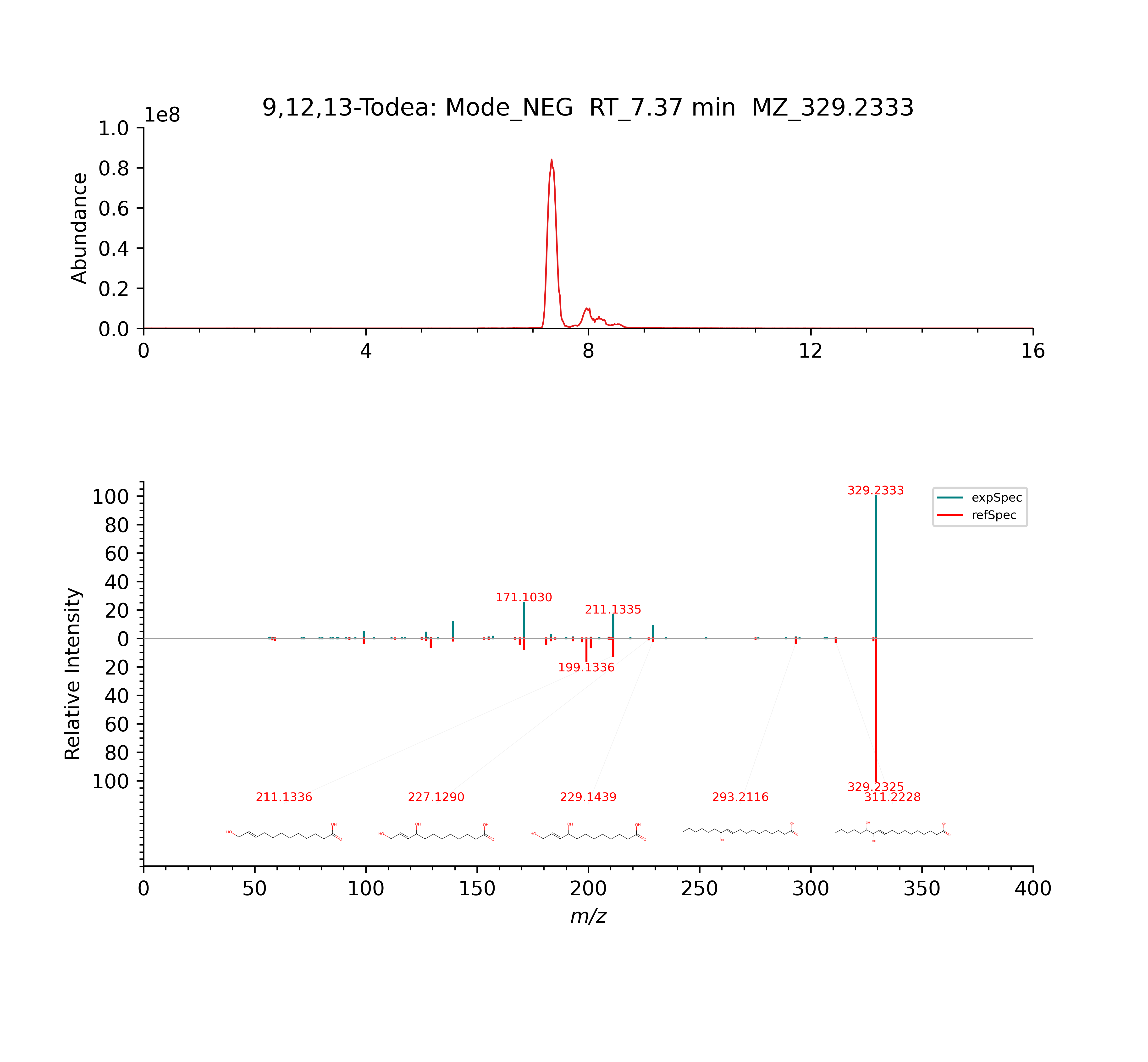

Supplement: Supplementary file 1 [file molecules-29-02840-s001.zip › Supplementary Figure s1/Identification from HerbDB datebase/png/compound00294.png]

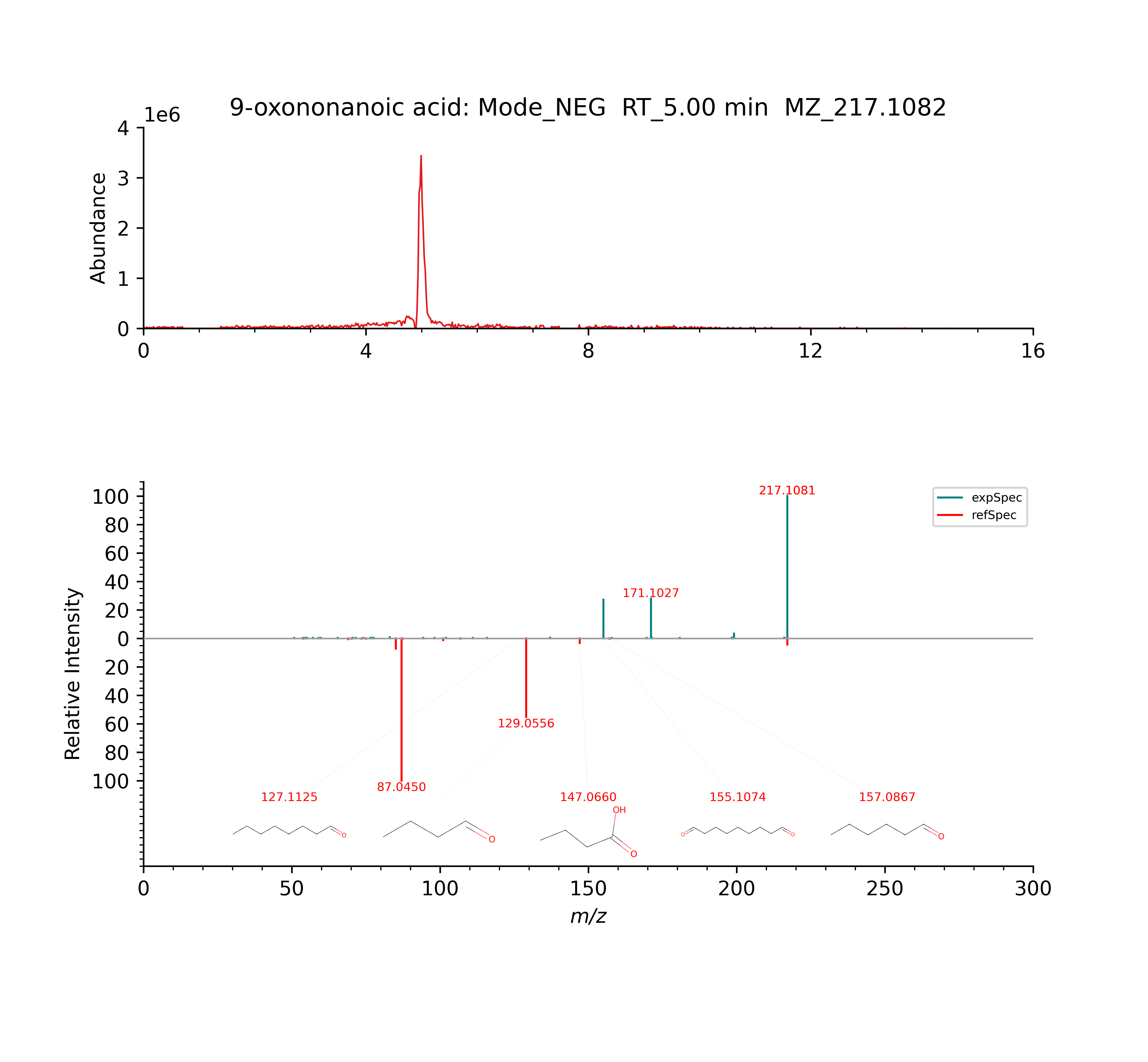

Supplement: Supplementary file 1 [file molecules-29-02840-s001.zip › Supplementary Figure s1/Identification from HerbDB datebase/png/compound00295.png]

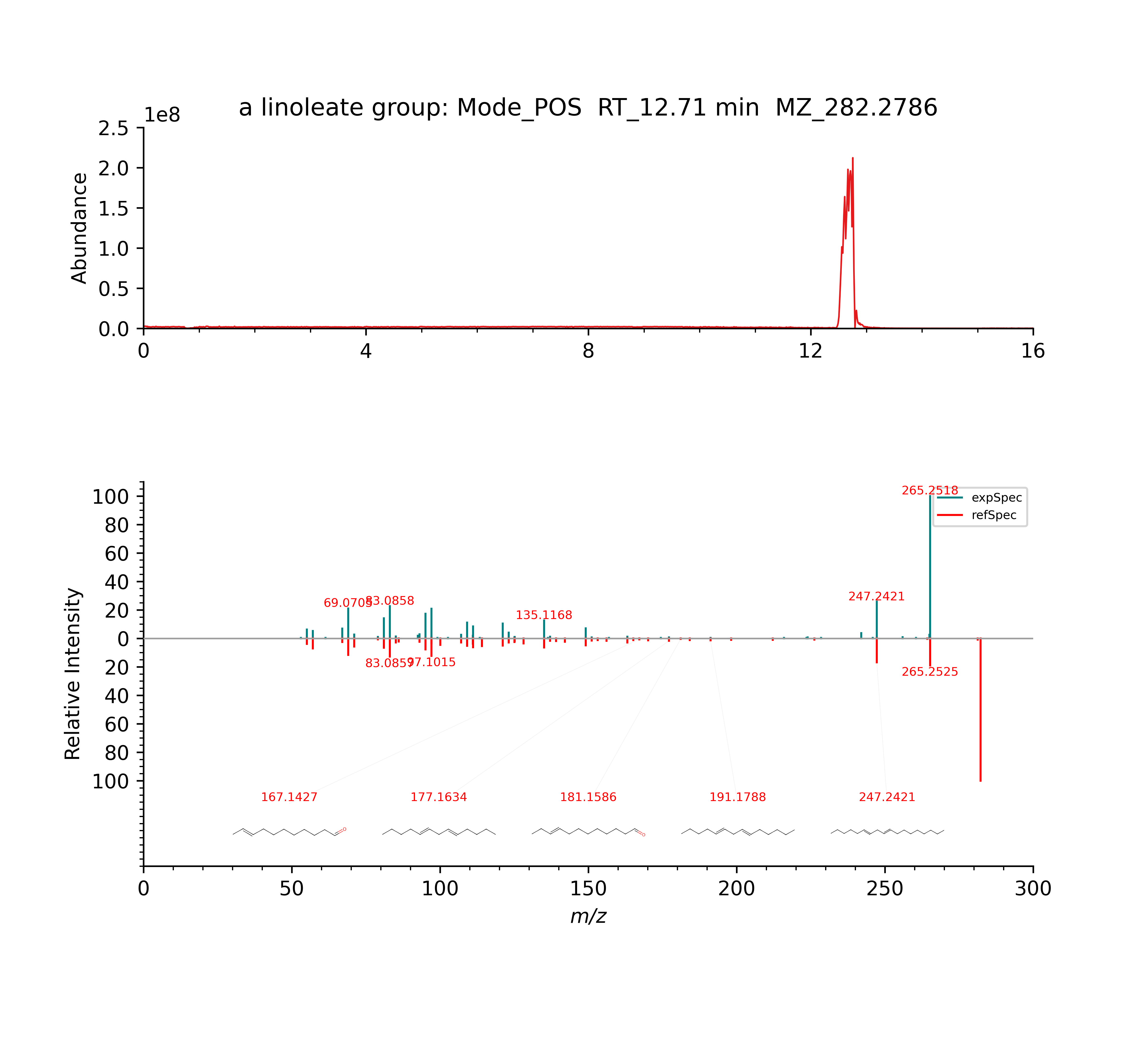

Supplement: Supplementary file 1 [file molecules-29-02840-s001.zip › Supplementary Figure s1/Identification from HerbDB datebase/png/compound00297.png]

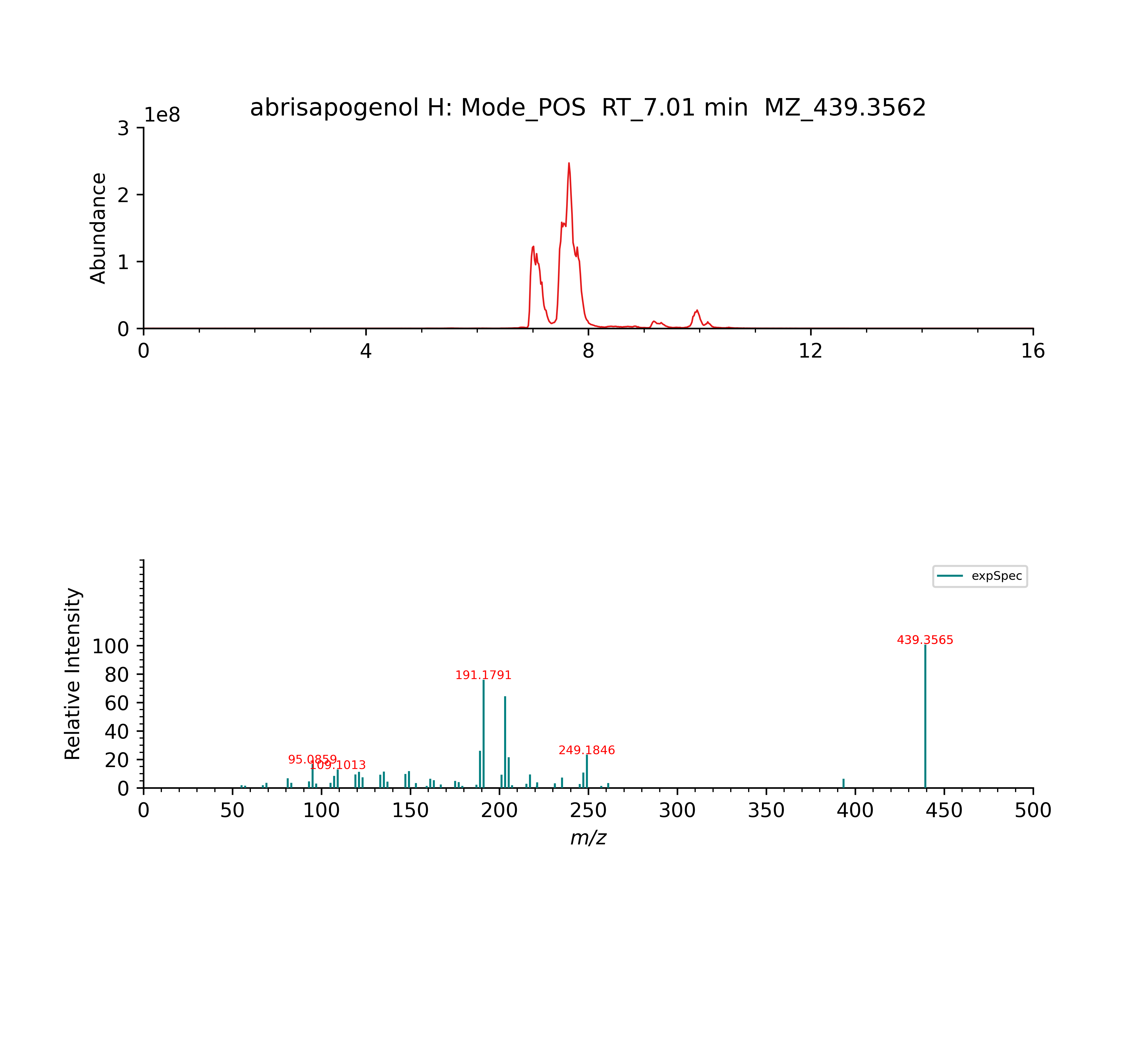

Supplement: Supplementary file 1 [file molecules-29-02840-s001.zip › Supplementary Figure s1/Identification from HerbDB datebase/png/compound00298.png]

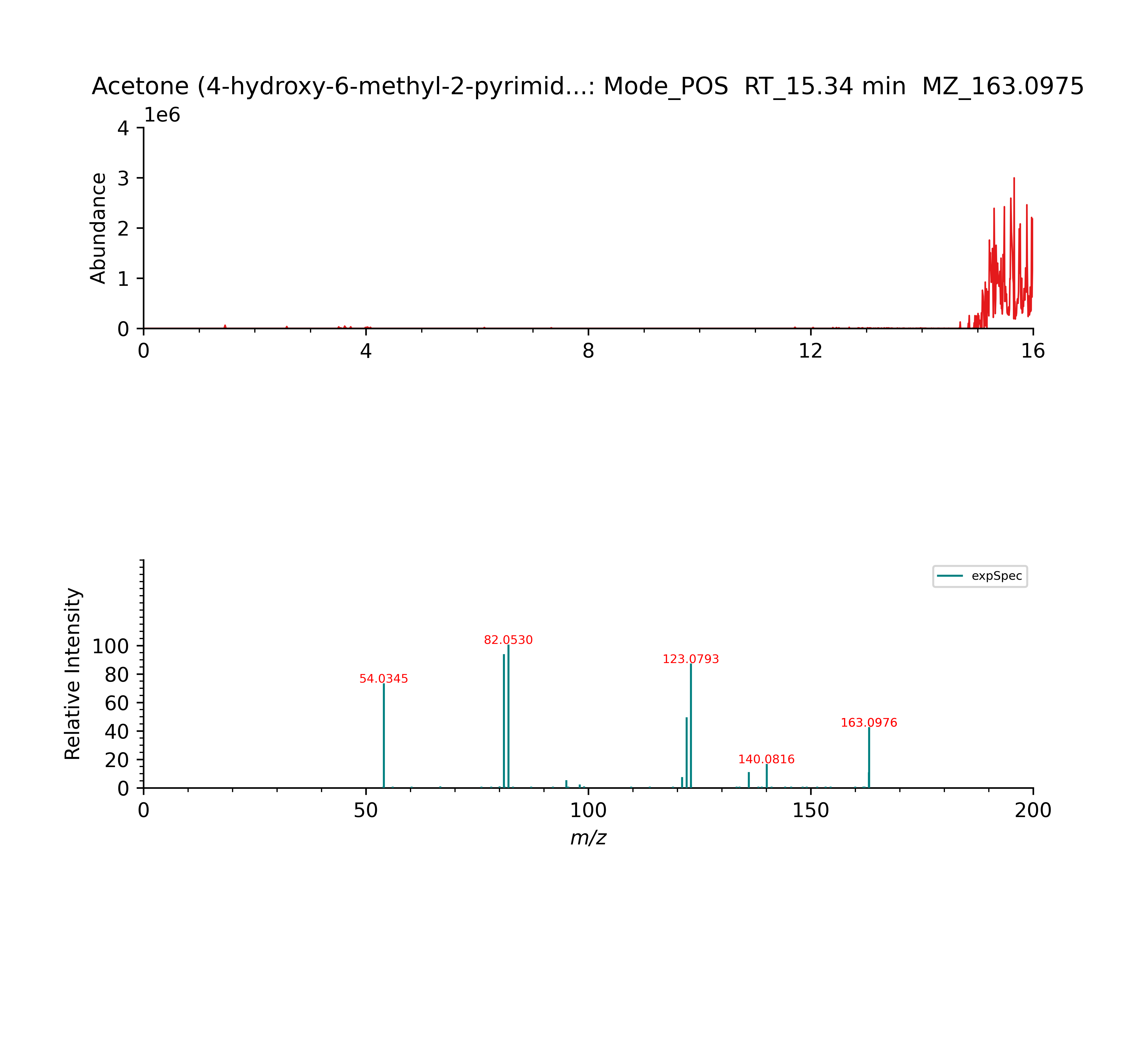

Supplement: Supplementary file 1 [file molecules-29-02840-s001.zip › Supplementary Figure s1/Identification from HerbDB datebase/png/compound00299.png]

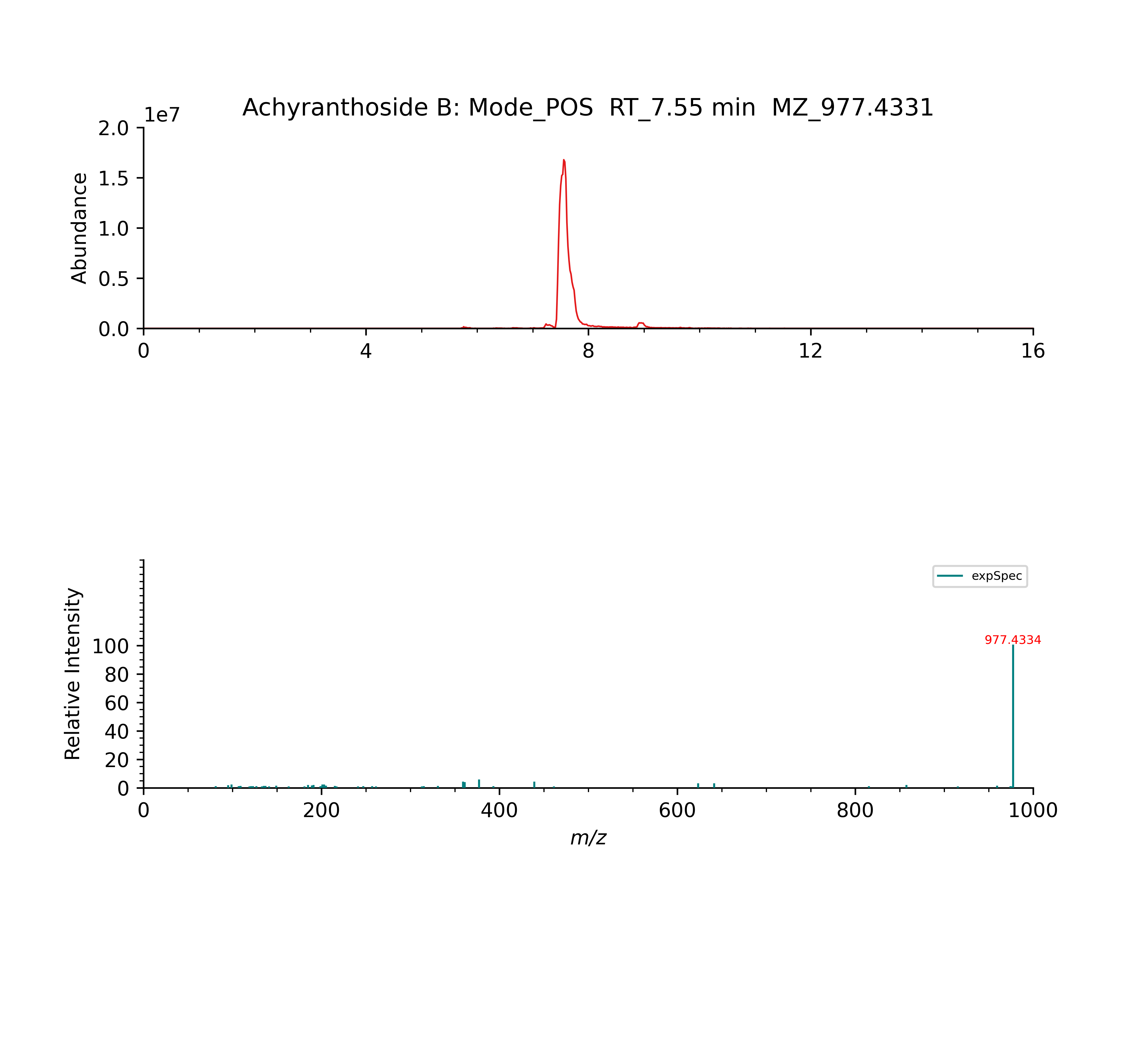

Supplement: Supplementary file 1 [file molecules-29-02840-s001.zip › Supplementary Figure s1/Identification from HerbDB datebase/png/compound00300.png]

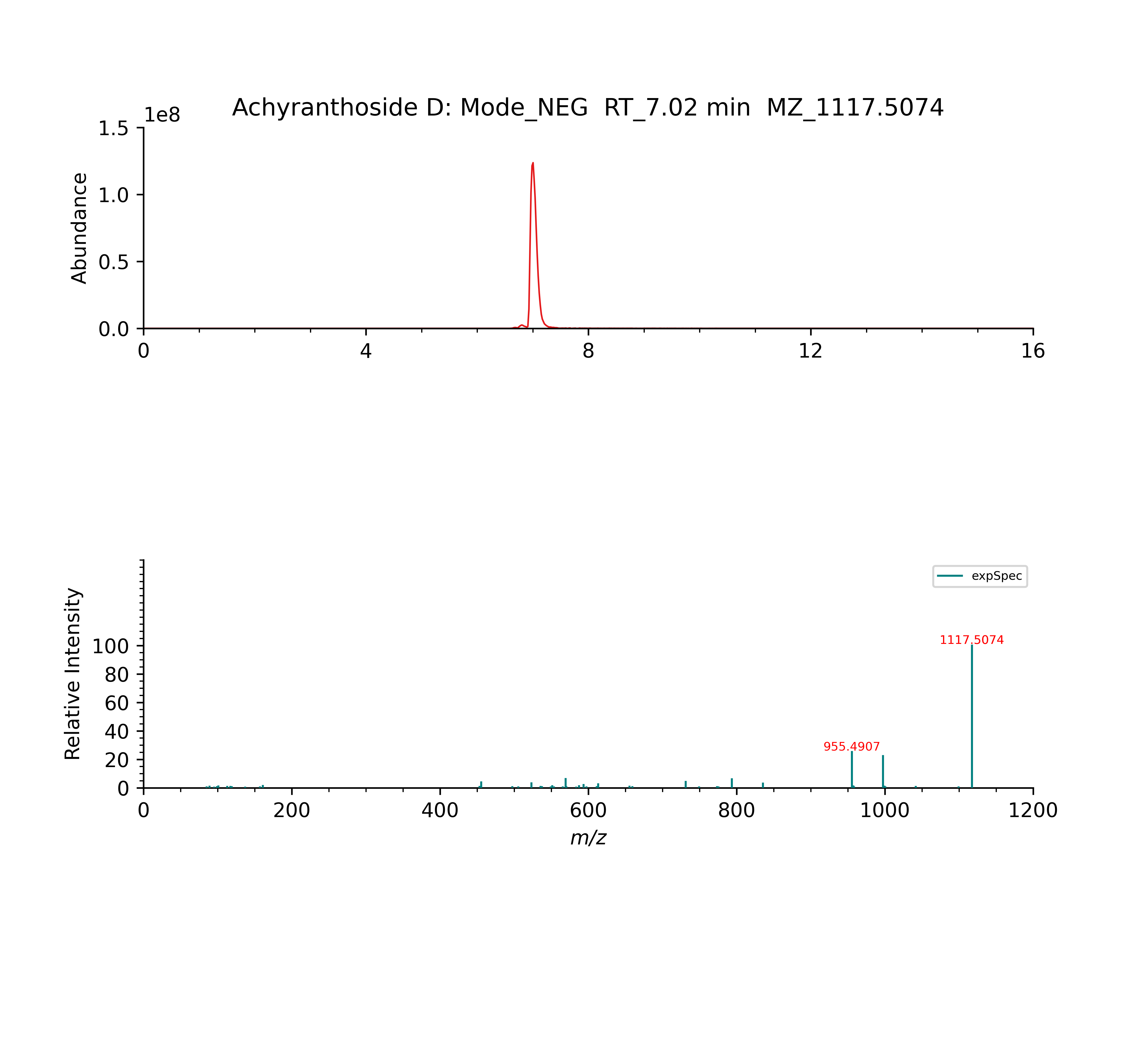

Supplement: Supplementary file 1 [file molecules-29-02840-s001.zip › Supplementary Figure s1/Identification from HerbDB datebase/png/compound00301.png]

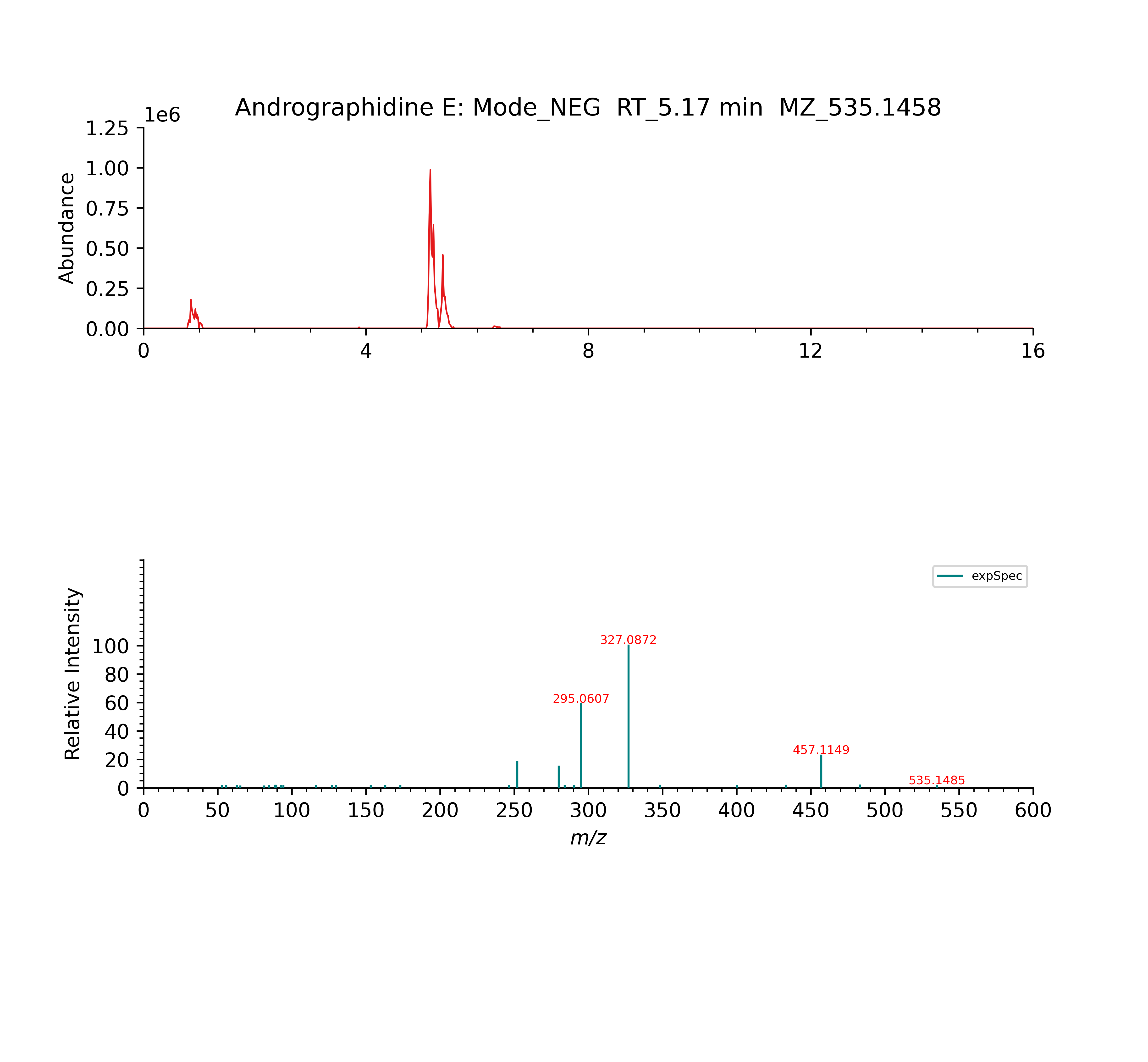

Supplement: Supplementary file 1 [file molecules-29-02840-s001.zip › Supplementary Figure s1/Identification from HerbDB datebase/png/compound00303.png]

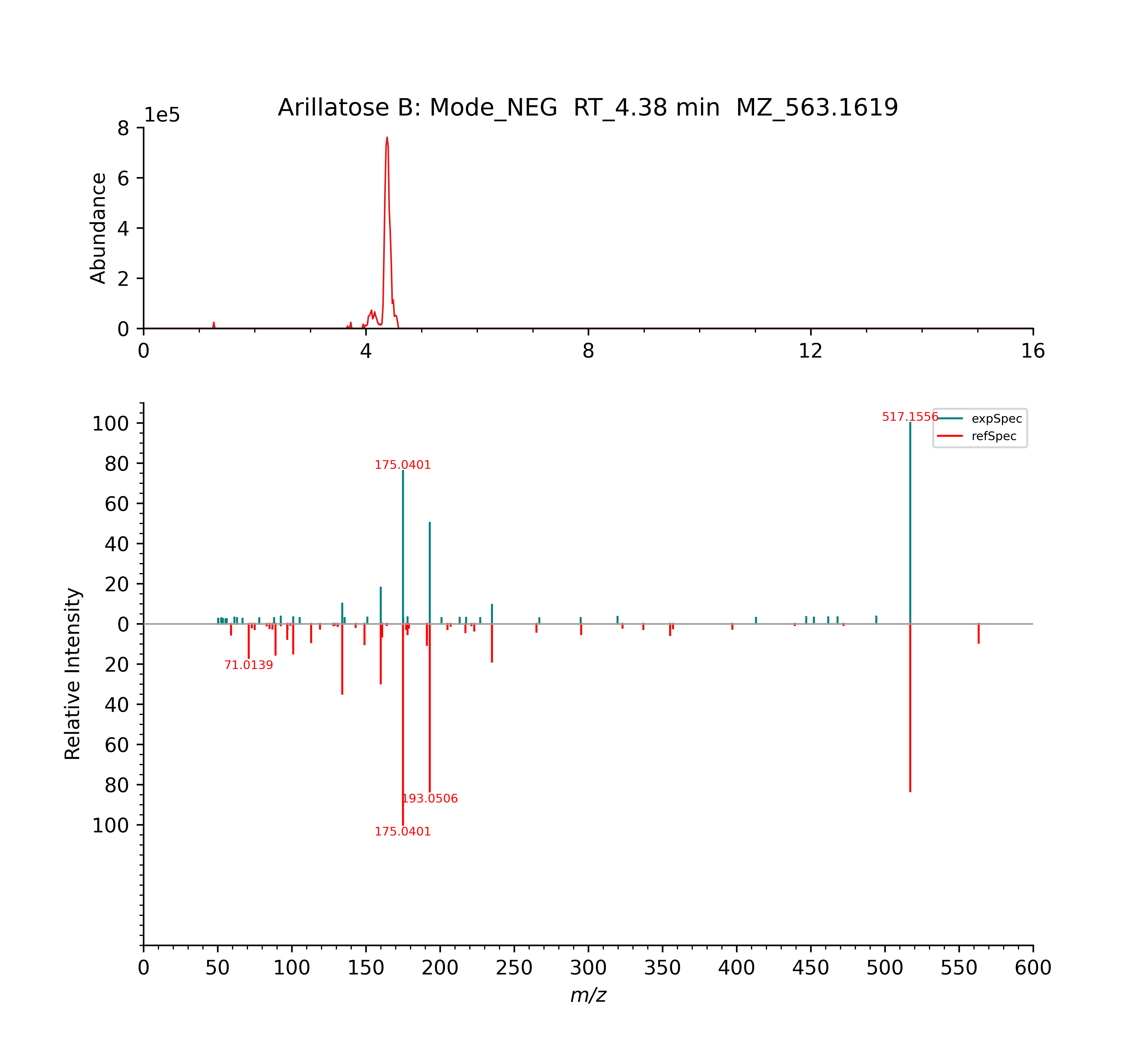

Supplement: Supplementary file 1 [file molecules-29-02840-s001.zip › Supplementary Figure s1/Identification from HerbDB datebase/png/compound00305.png]

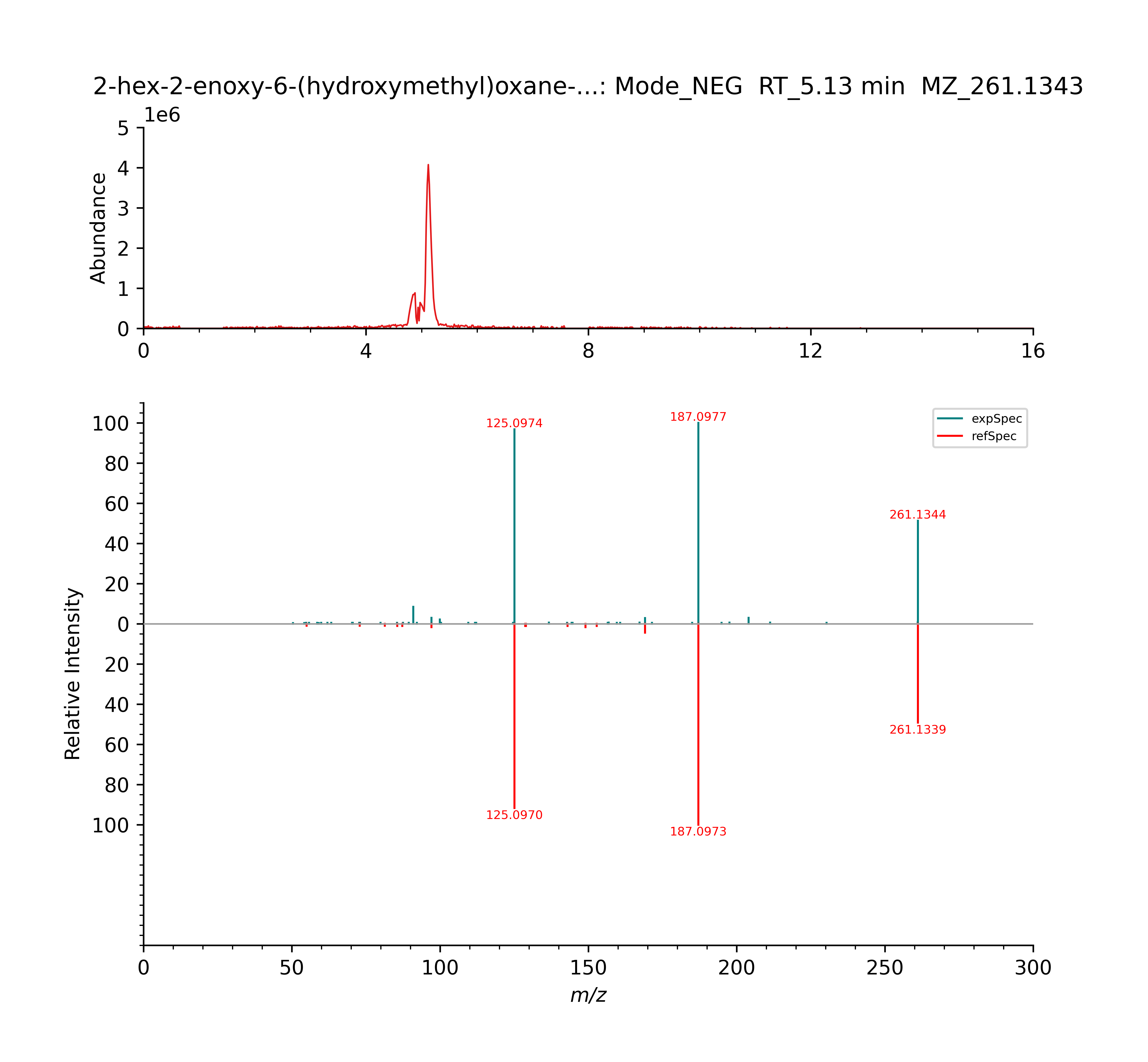

Supplement: Supplementary file 1 [file molecules-29-02840-s001.zip › Supplementary Figure s1/Identification from HerbDB datebase/png/compound00307.png]

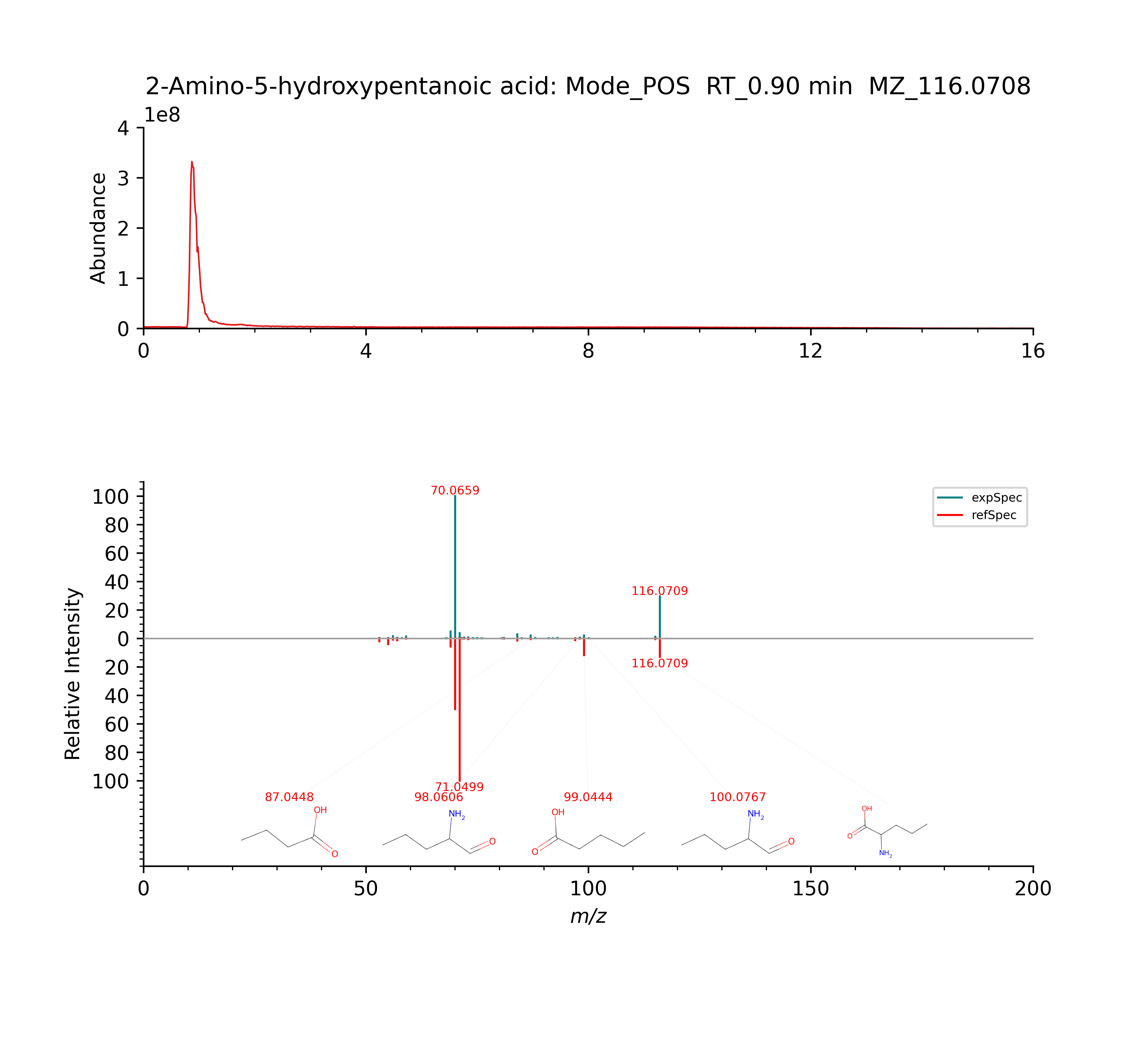

Supplement: Supplementary file 1 [file molecules-29-02840-s001.zip › Supplementary Figure s1/Identification from HerbDB datebase/png/compound00309.png]

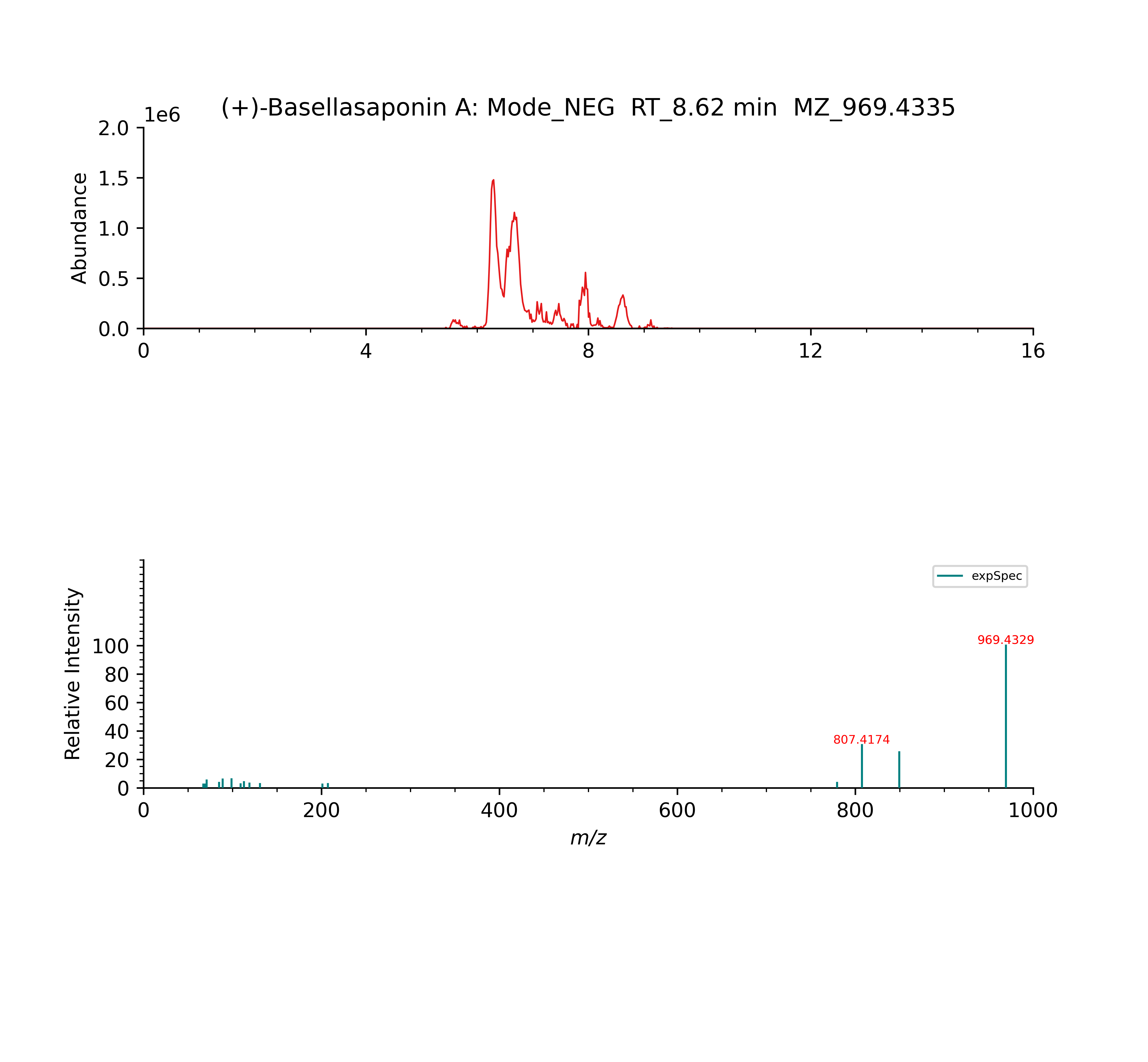

Supplement: Supplementary file 1 [file molecules-29-02840-s001.zip › Supplementary Figure s1/Identification from HerbDB datebase/png/compound00311.png]

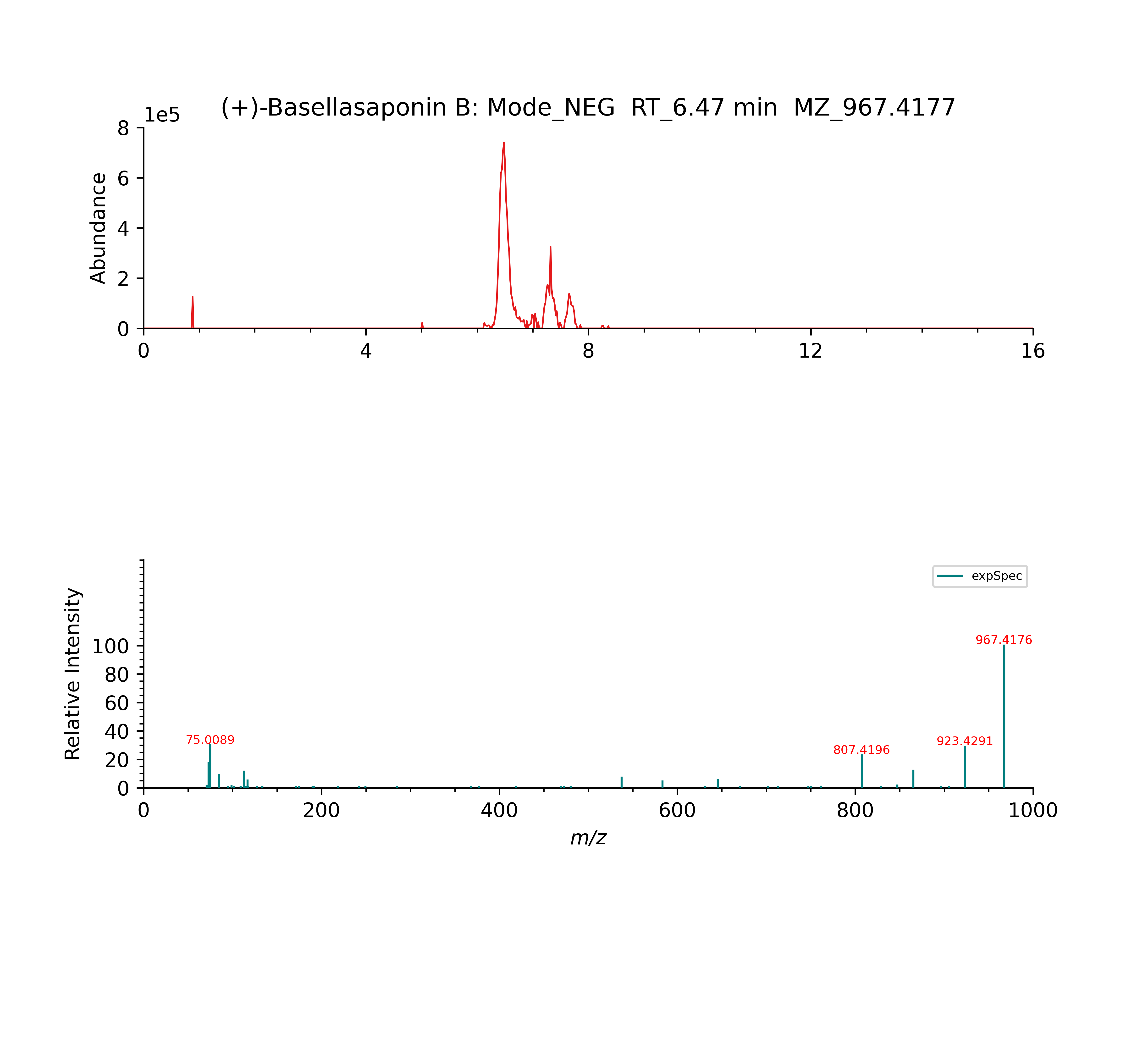

Supplement: Supplementary file 1 [file molecules-29-02840-s001.zip › Supplementary Figure s1/Identification from HerbDB datebase/png/compound00312.png]

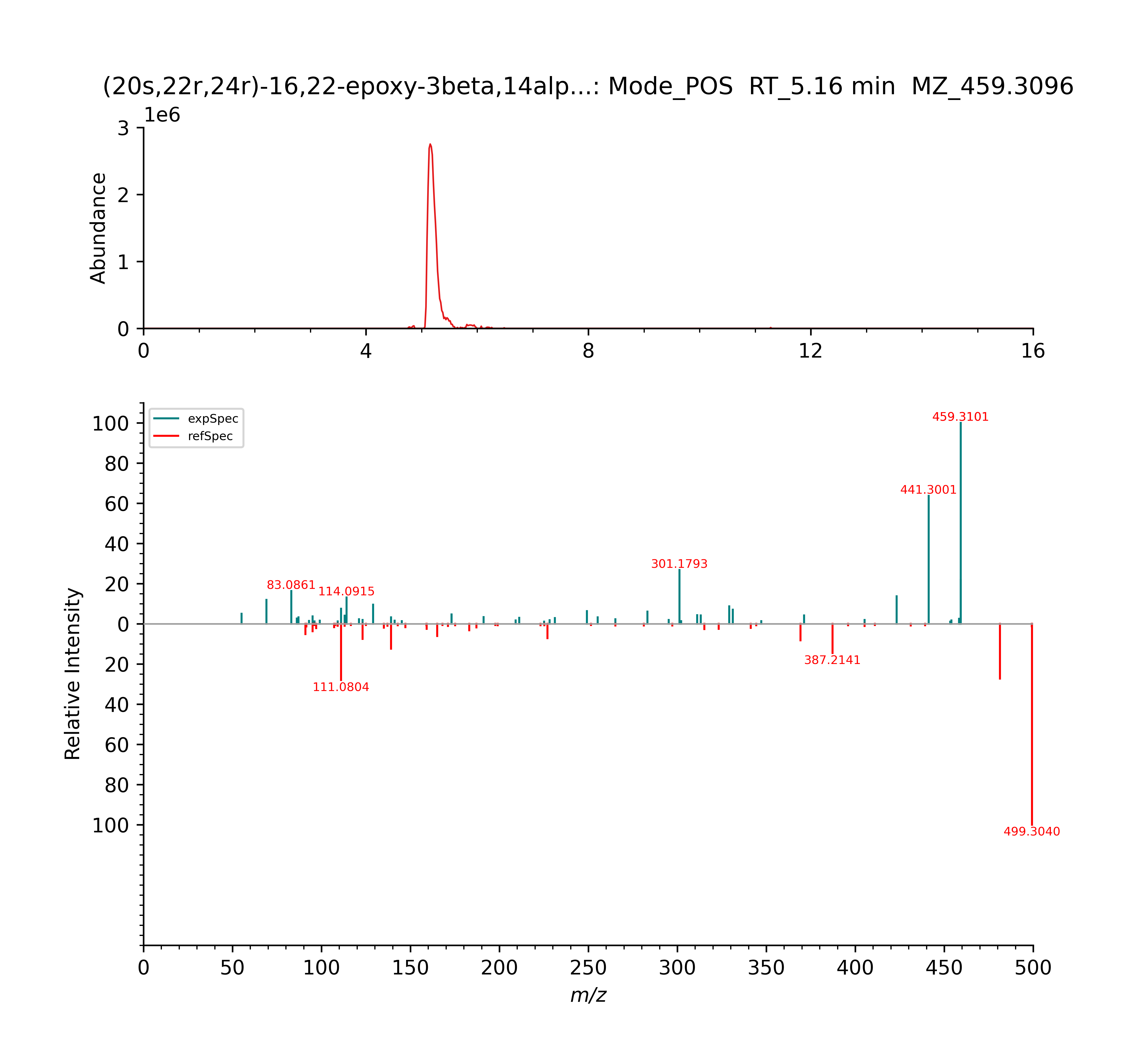

Supplement: Supplementary file 1 [file molecules-29-02840-s001.zip › Supplementary Figure s1/Identification from HerbDB datebase/png/compound00313.png]

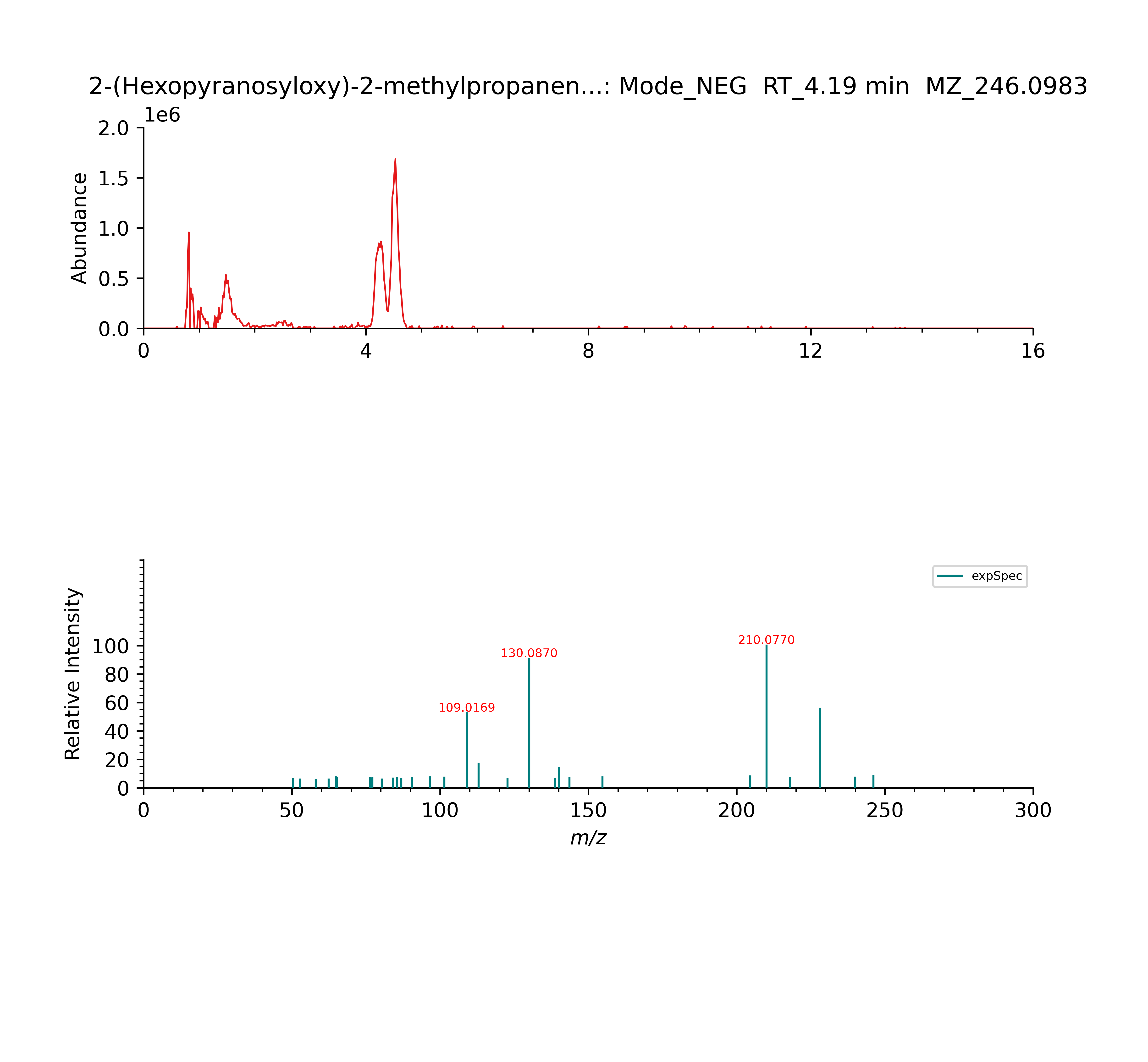

Supplement: Supplementary file 1 [file molecules-29-02840-s001.zip › Supplementary Figure s1/Identification from HerbDB datebase/png/compound00314.png]

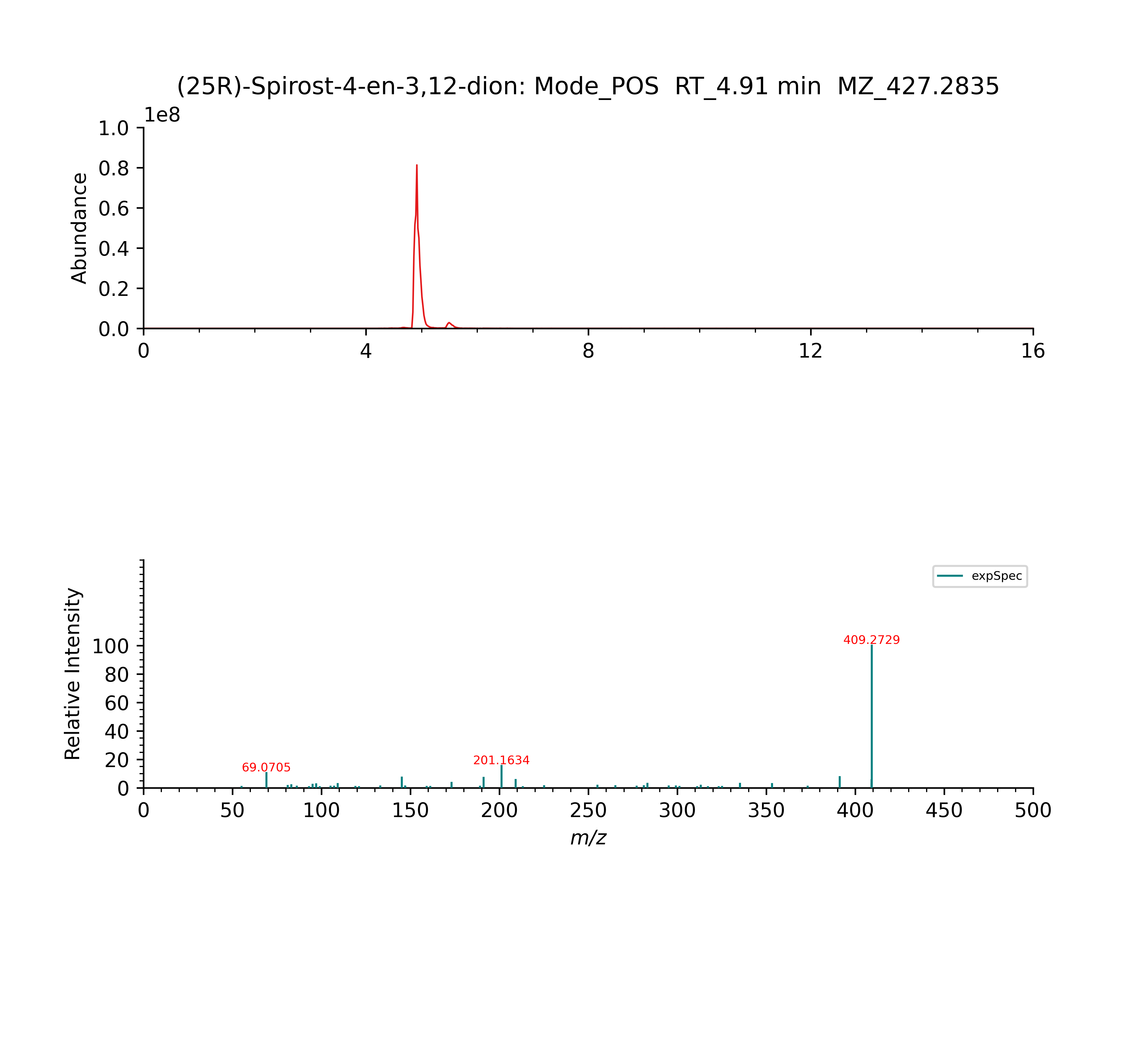

Supplement: Supplementary file 1 [file molecules-29-02840-s001.zip › Supplementary Figure s1/Identification from HerbDB datebase/png/compound00315.png]

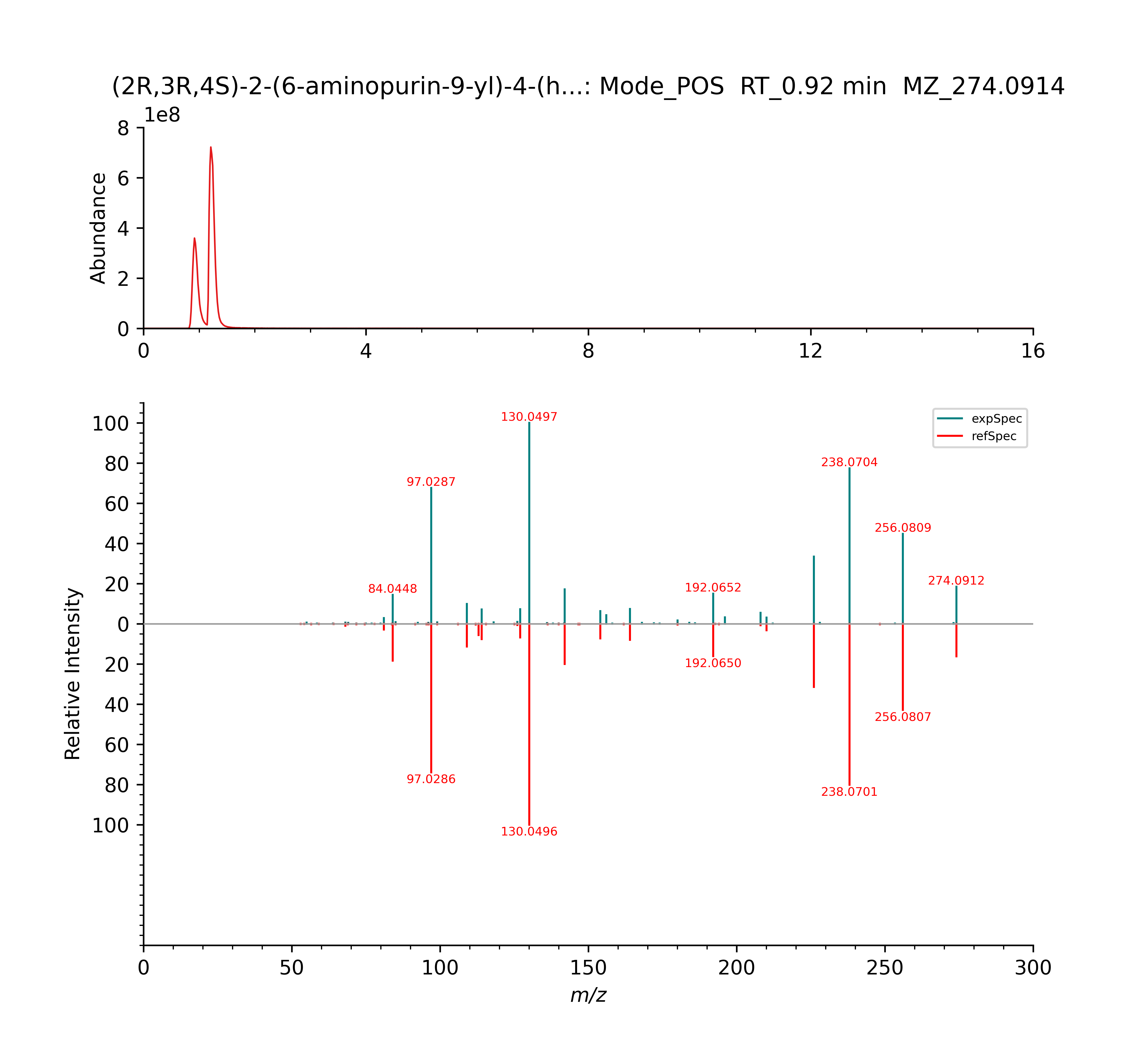

Supplement: Supplementary file 1 [file molecules-29-02840-s001.zip › Supplementary Figure s1/Identification from HerbDB datebase/png/compound00316.png]

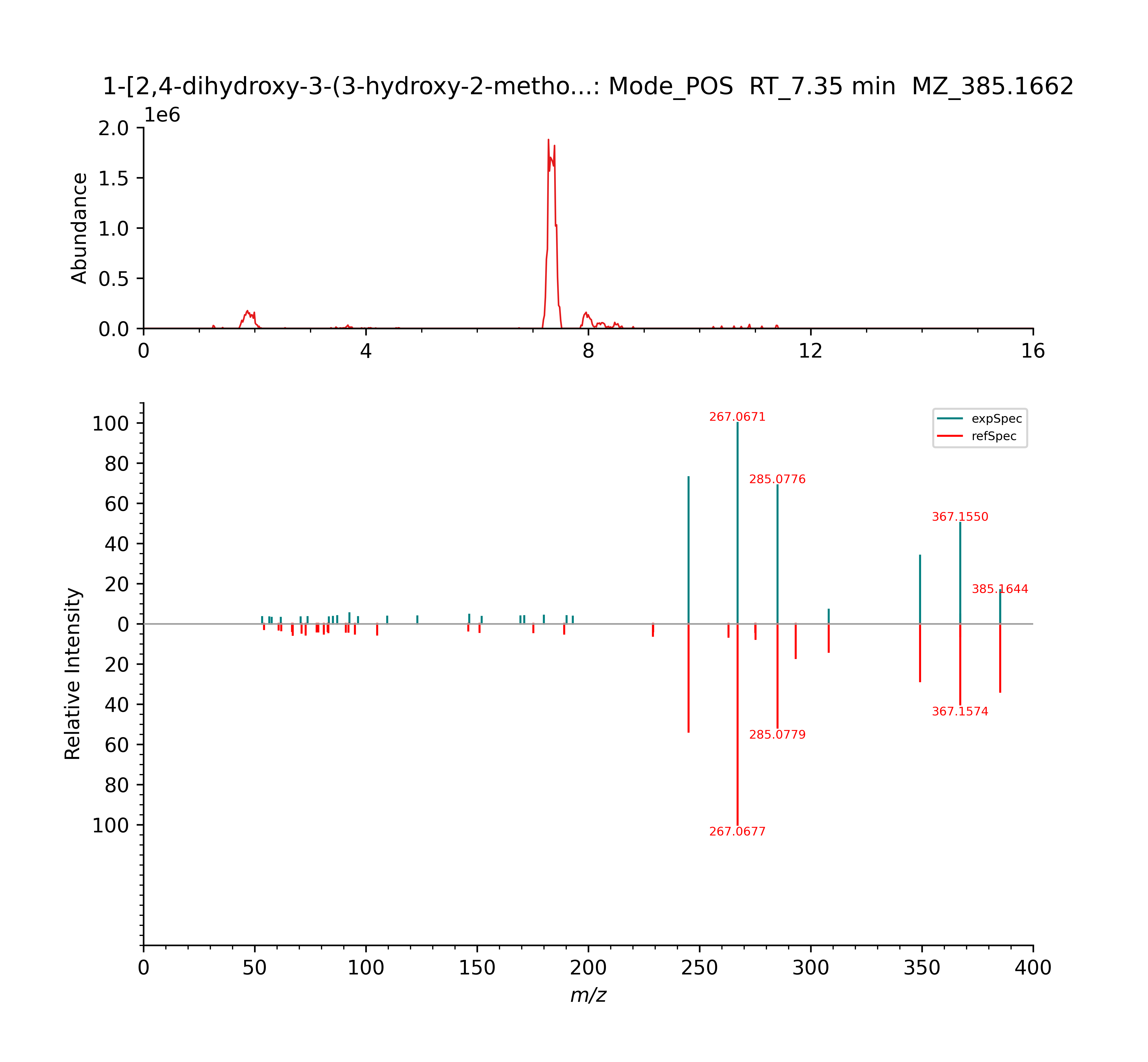

Supplement: Supplementary file 1 [file molecules-29-02840-s001.zip › Supplementary Figure s1/Identification from HerbDB datebase/png/compound00318.png]

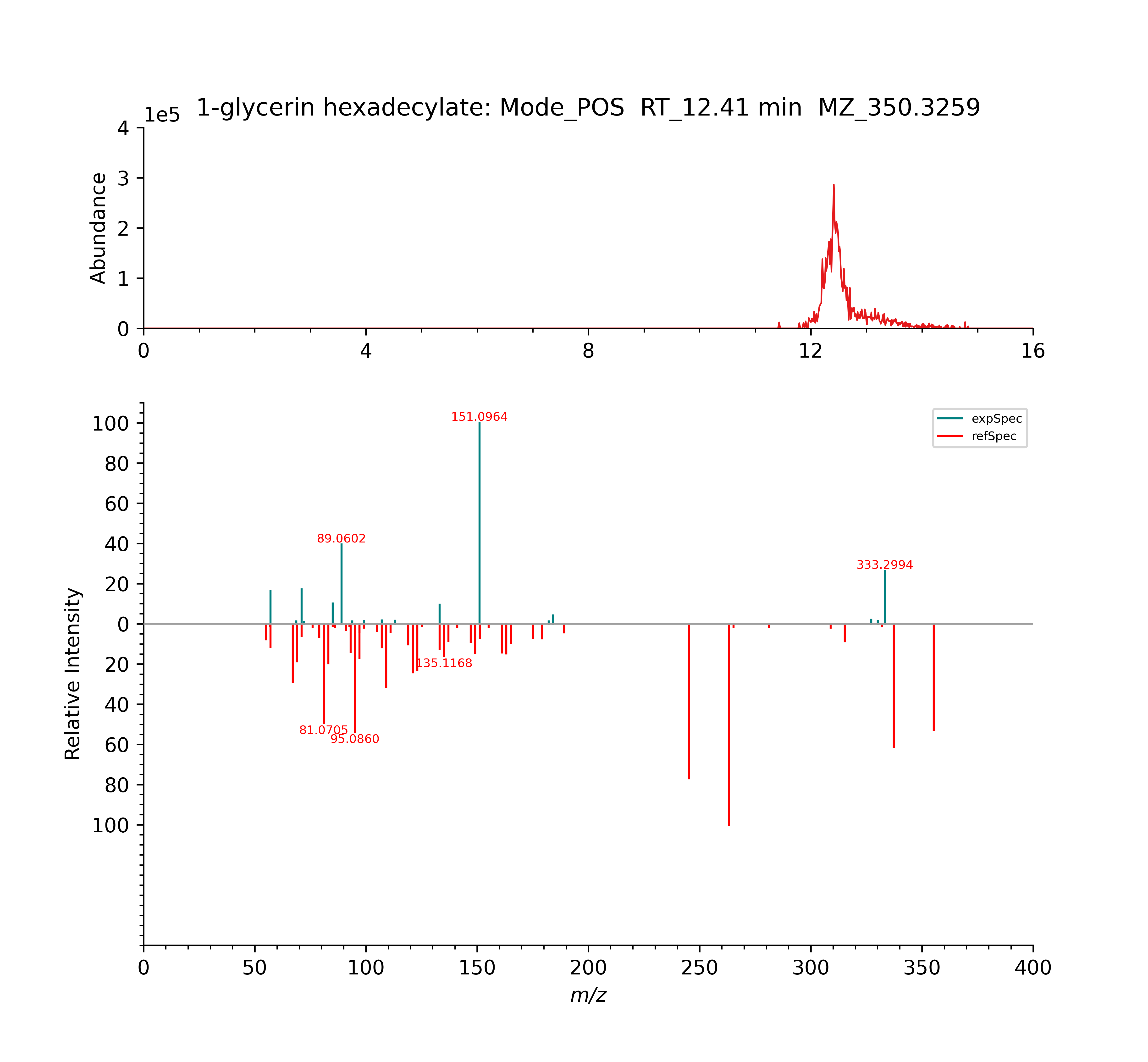

Supplement: Supplementary file 1 [file molecules-29-02840-s001.zip › Supplementary Figure s1/Identification from HerbDB datebase/png/compound00320.png]

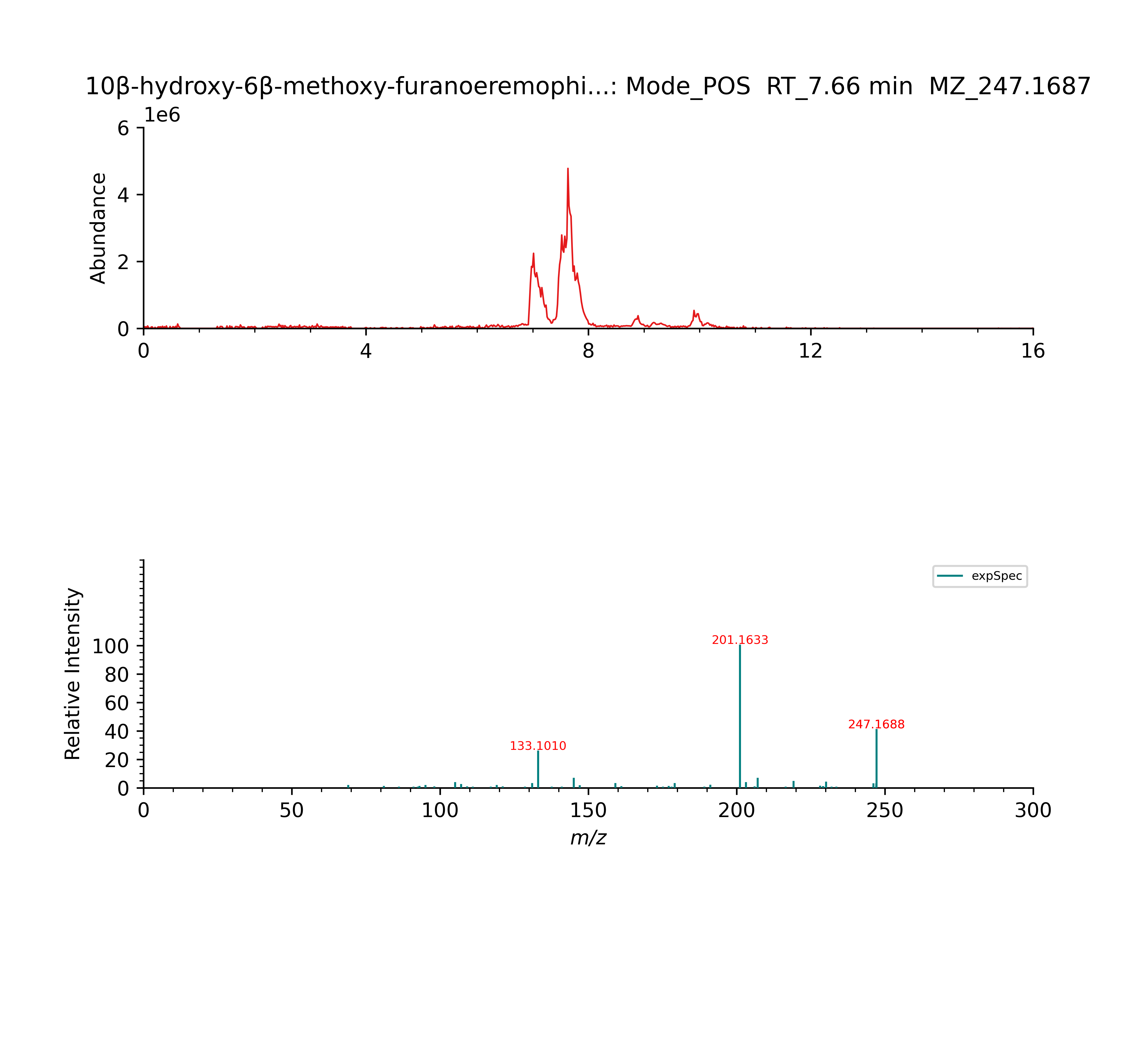

Supplement: Supplementary file 1 [file molecules-29-02840-s001.zip › Supplementary Figure s1/Identification from HerbDB datebase/png/compound00323.png]

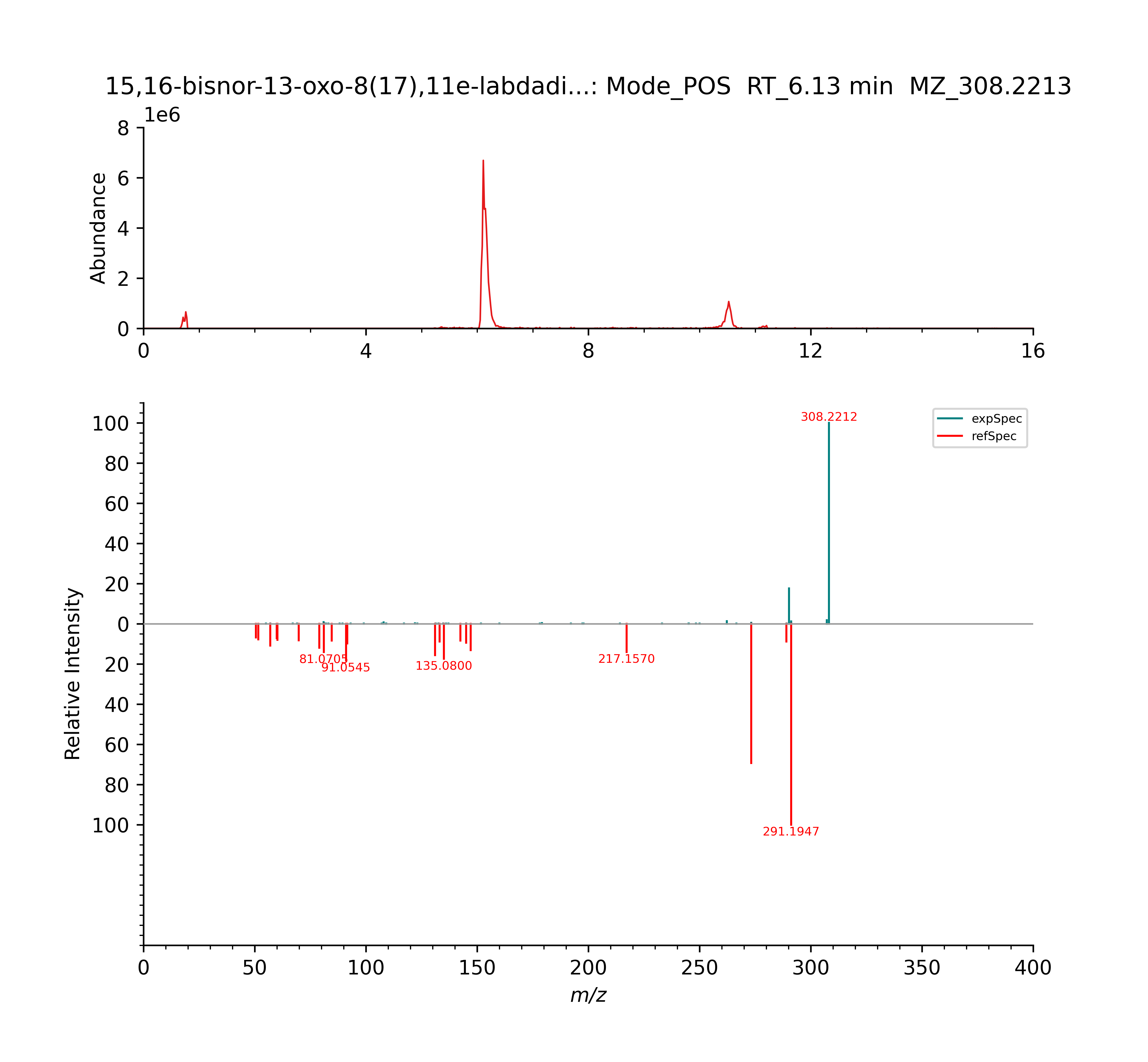

Supplement: Supplementary file 1 [file molecules-29-02840-s001.zip › Supplementary Figure s1/Identification from HerbDB datebase/png/compound00324.png]

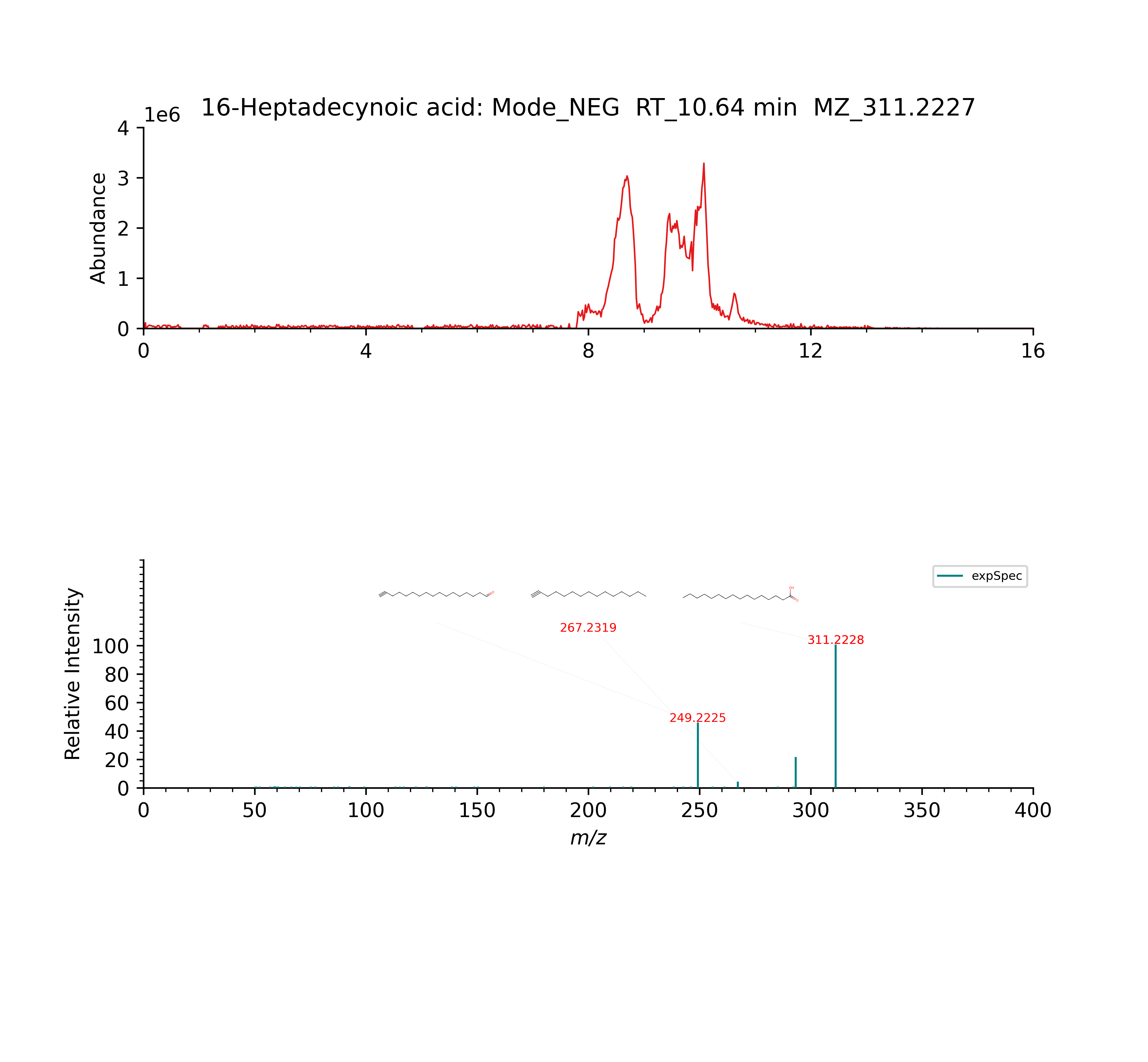

Supplement: Supplementary file 1 [file molecules-29-02840-s001.zip › Supplementary Figure s1/Identification from HerbDB datebase/png/compound00325.png]

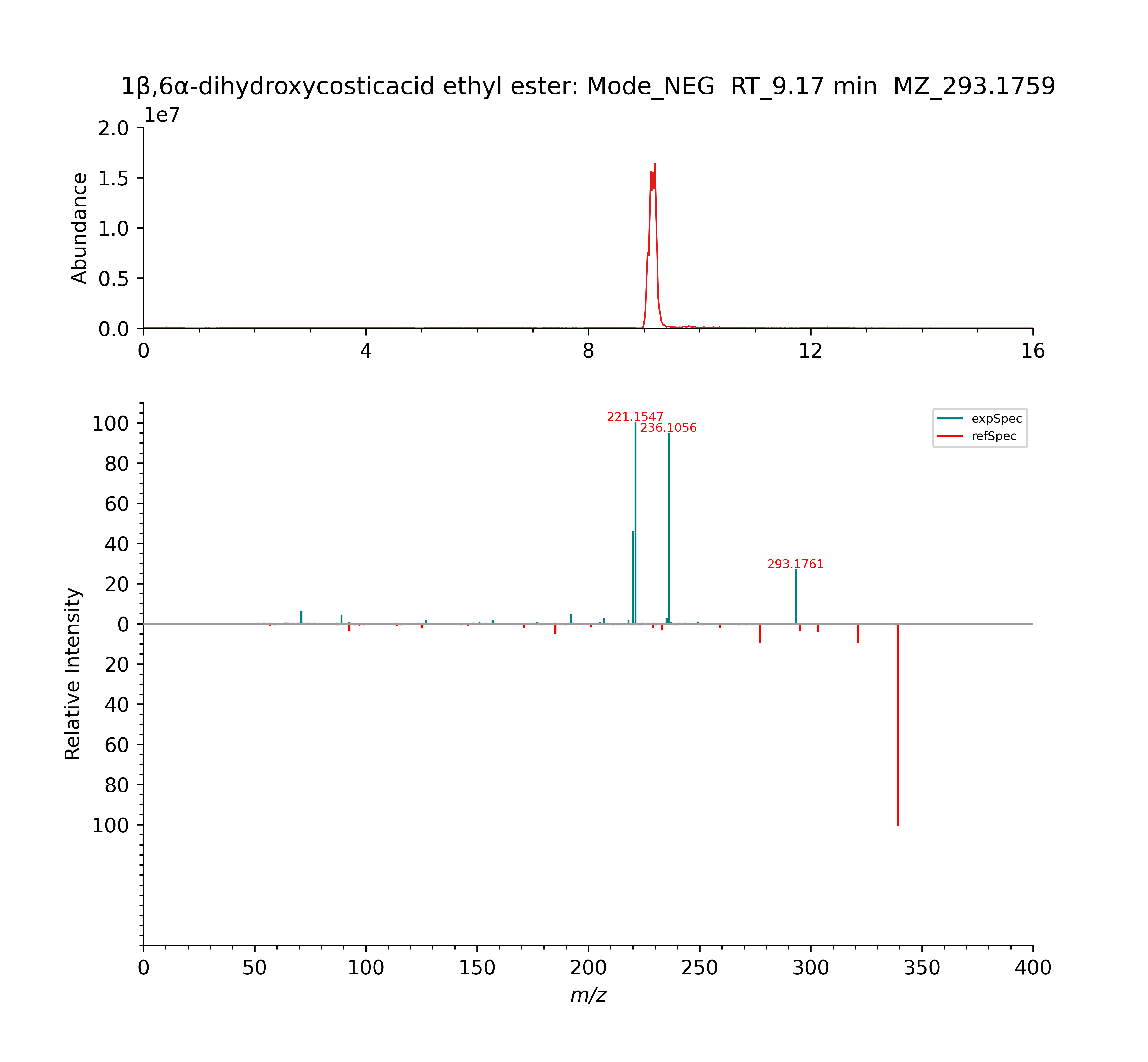

Supplement: Supplementary file 1 [file molecules-29-02840-s001.zip › Supplementary Figure s1/Identification from HerbDB datebase/png/compound00326.png]

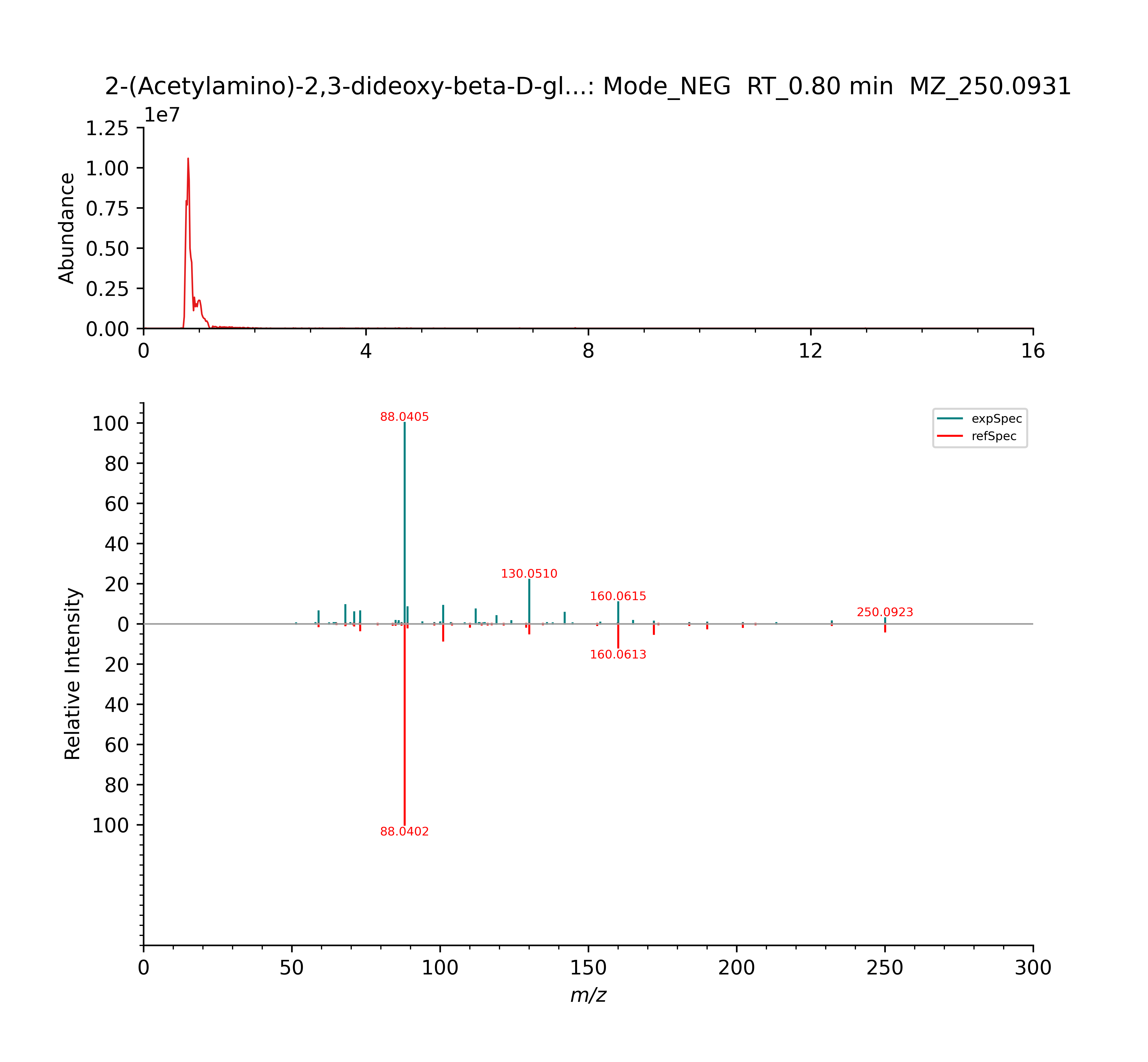

Supplement: Supplementary file 1 [file molecules-29-02840-s001.zip › Supplementary Figure s1/Identification from HerbDB datebase/png/compound00328.png]

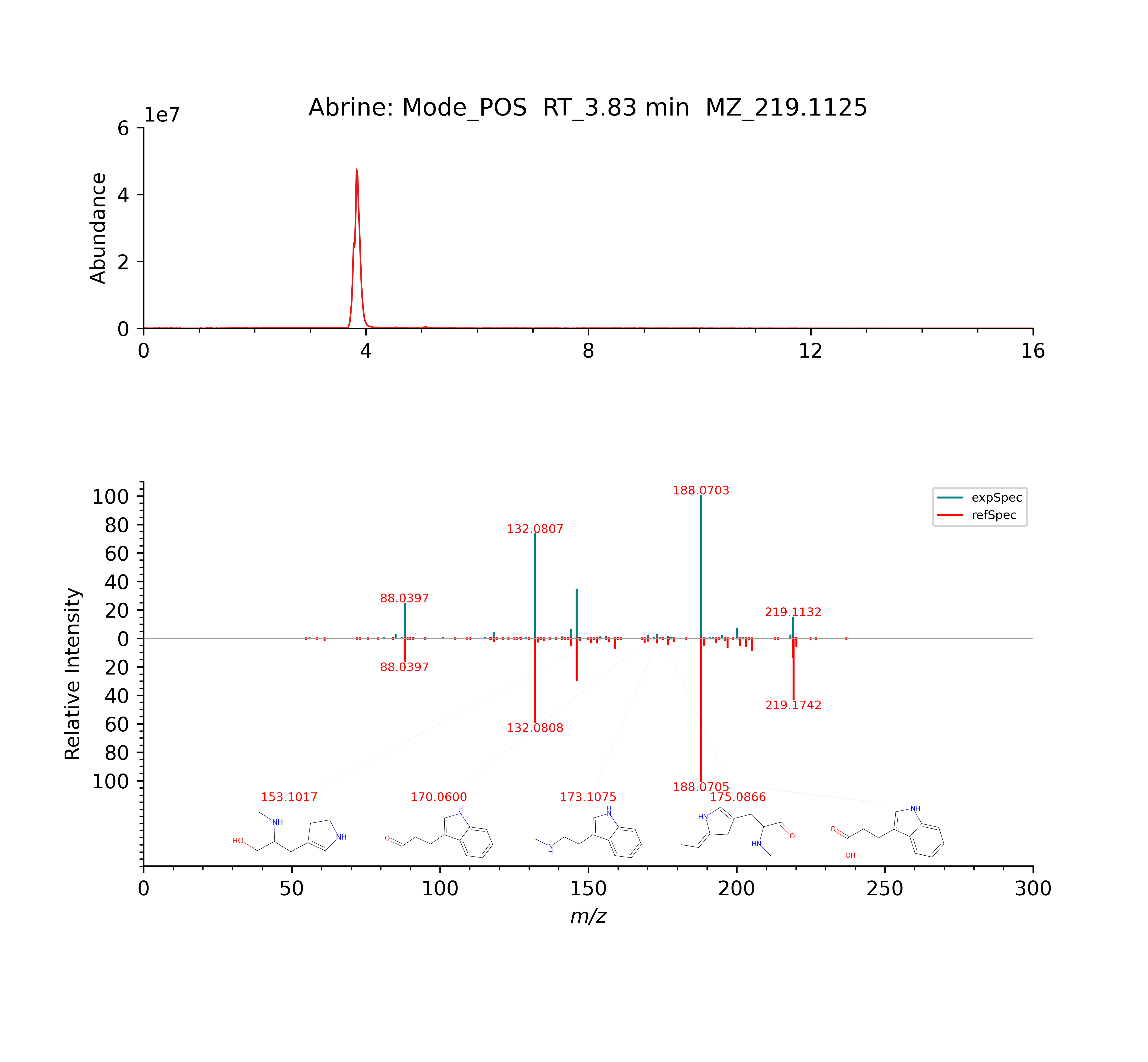

Supplement: Supplementary file 1 [file molecules-29-02840-s001.zip › Supplementary Figure s1/Identification from LuMet-CM datebase/png/compound00001.png]

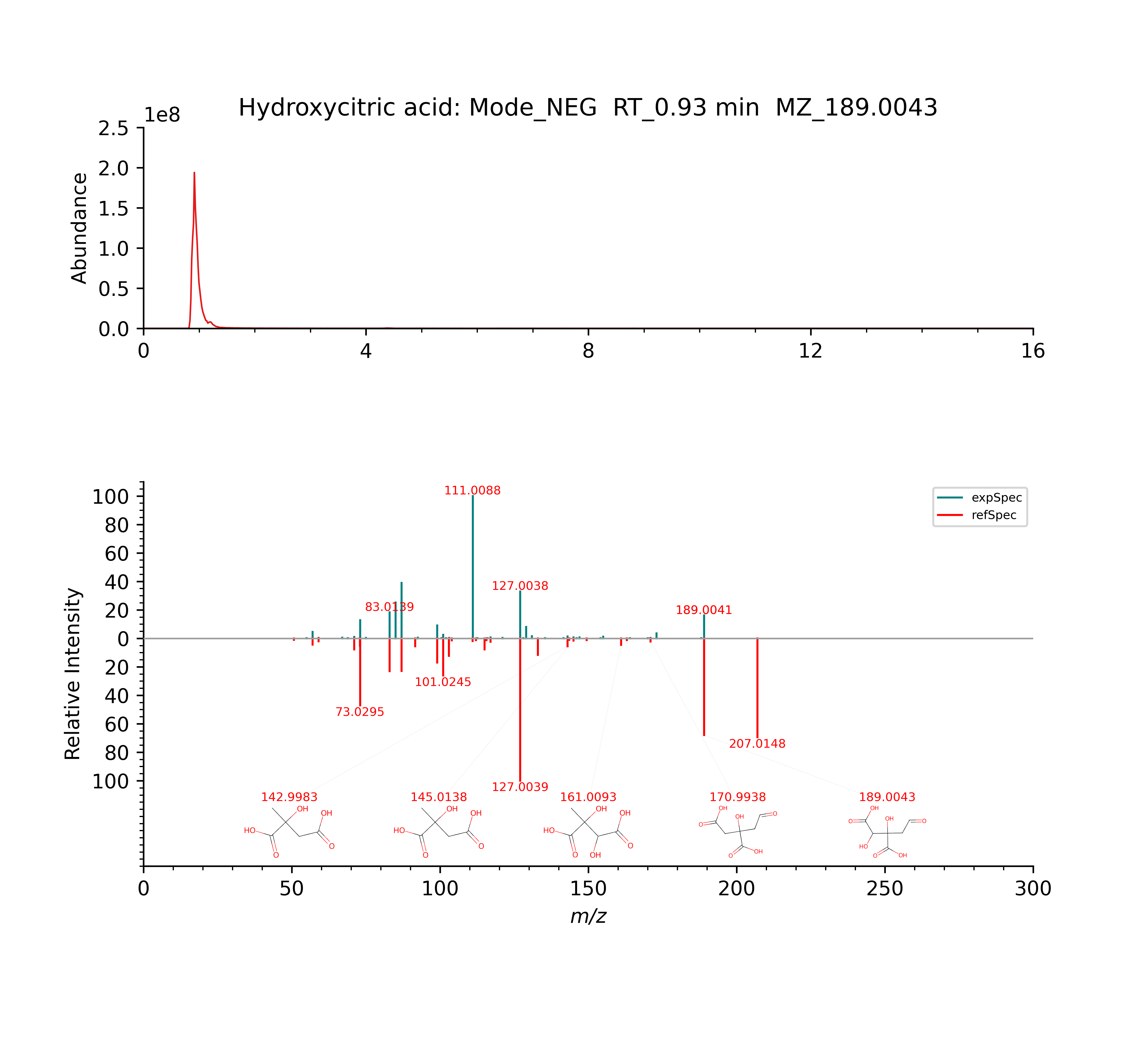

Supplement: Supplementary file 1 [file molecules-29-02840-s001.zip › Supplementary Figure s1/Identification from LuMet-CM datebase/png/compound00002.png]

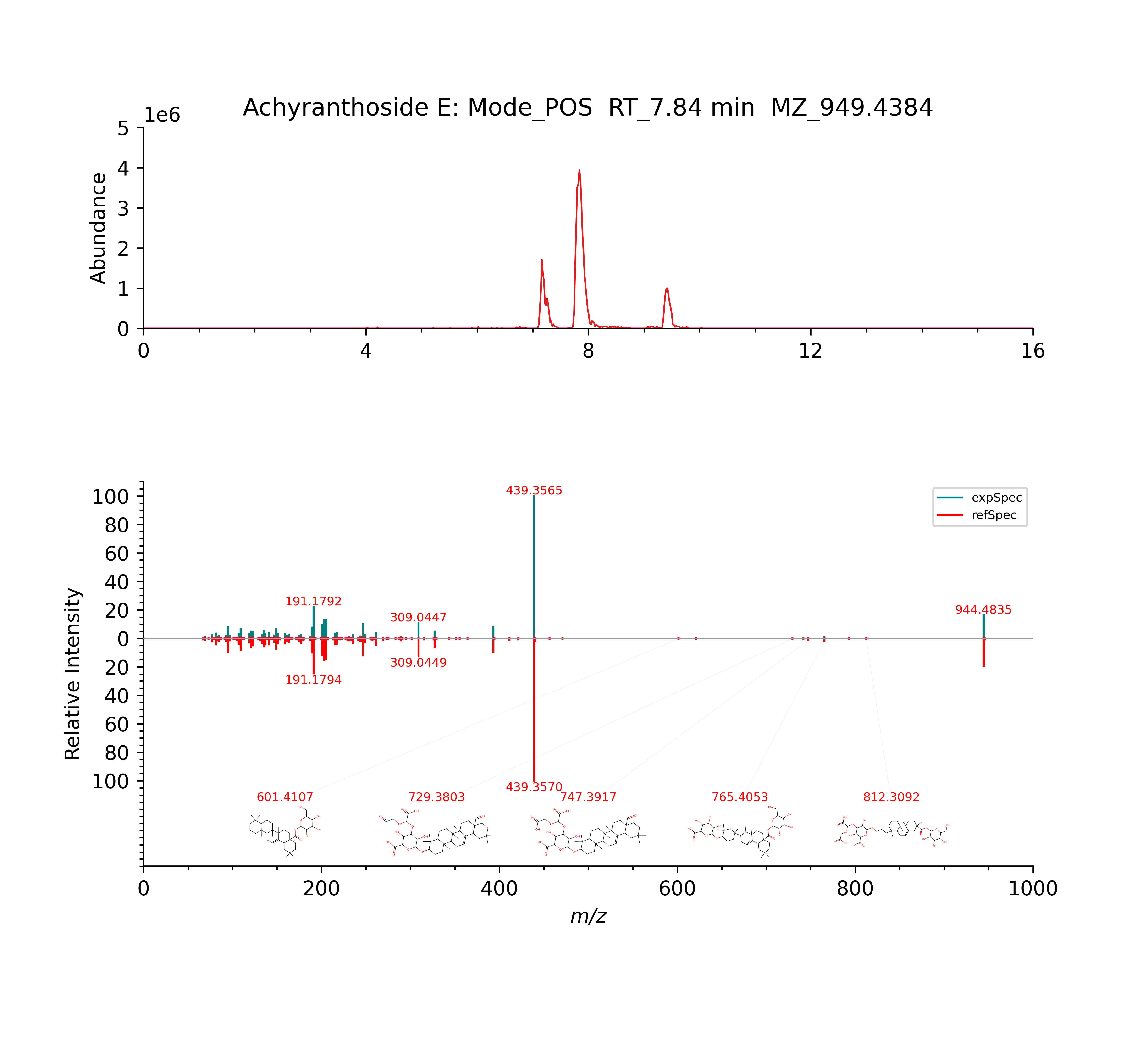

Supplement: Supplementary file 1 [file molecules-29-02840-s001.zip › Supplementary Figure s1/Identification from LuMet-CM datebase/png/compound00053.png]

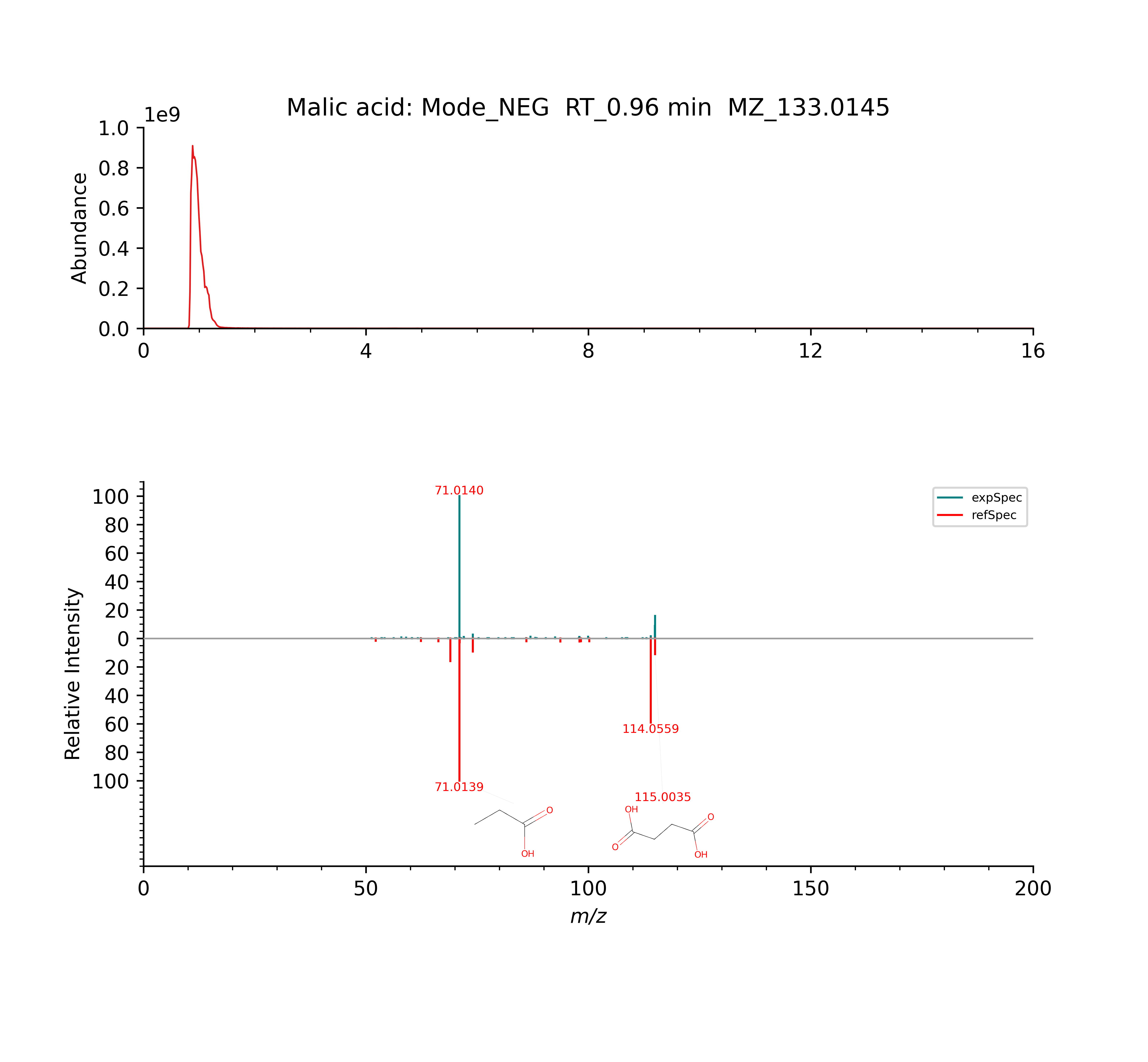

Supplement: Supplementary file 1 [file molecules-29-02840-s001.zip › Supplementary Figure s1/Identification from LuMet-CM datebase/png/compound00054.png]

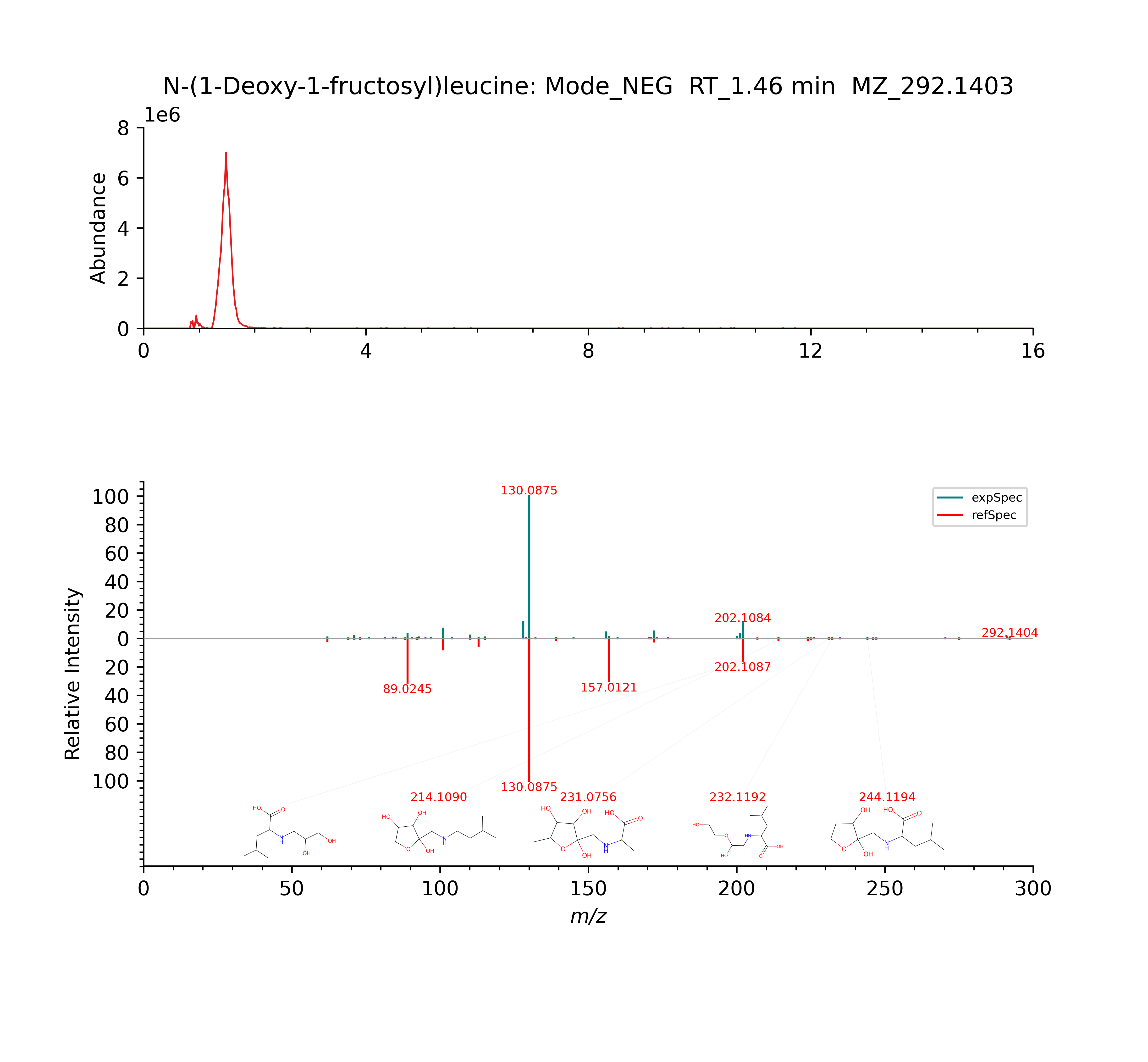

Supplement: Supplementary file 1 [file molecules-29-02840-s001.zip › Supplementary Figure s1/Identification from LuMet-CM datebase/png/compound00055.png]

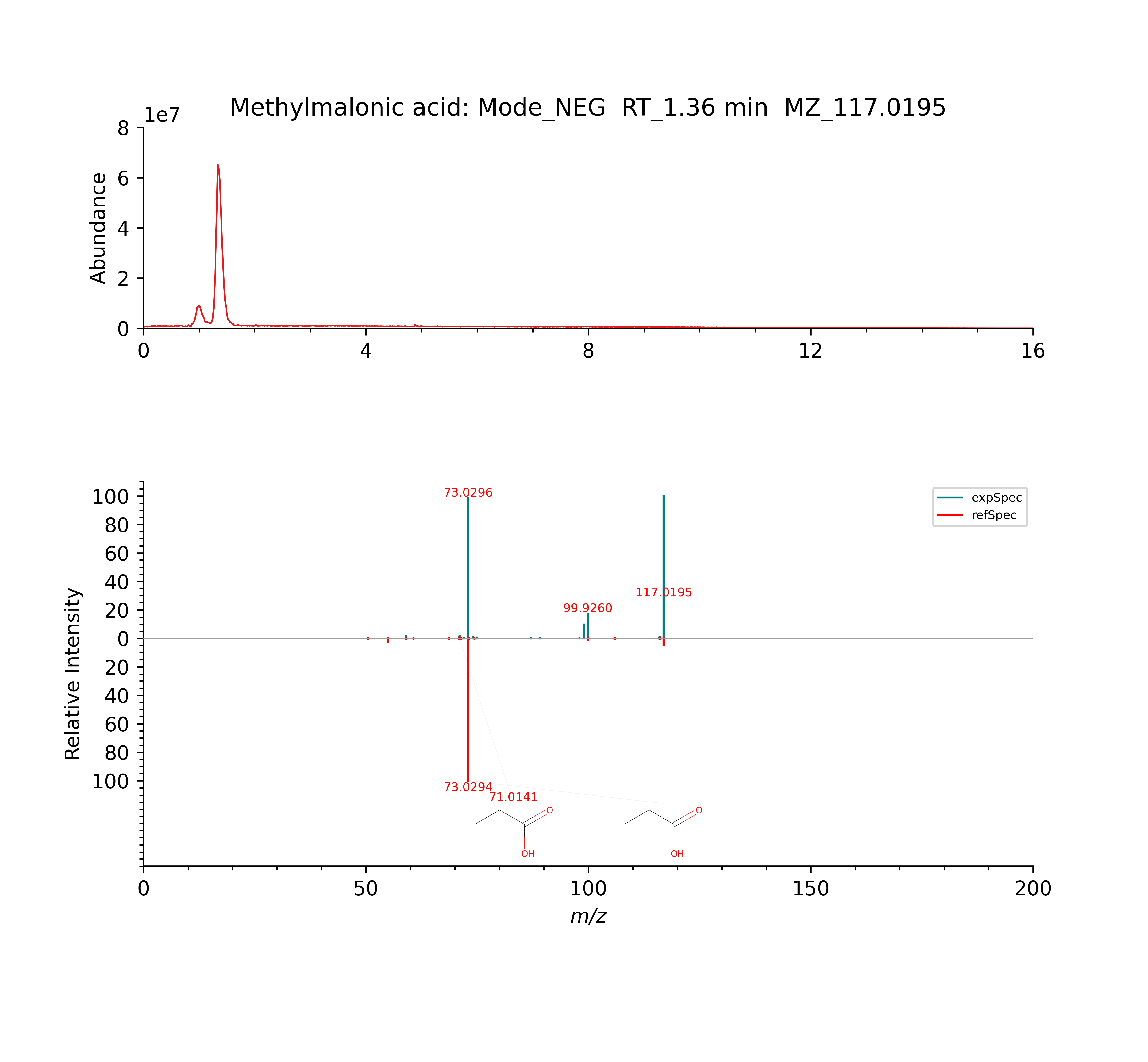

Supplement: Supplementary file 1 [file molecules-29-02840-s001.zip › Supplementary Figure s1/Identification from LuMet-CM datebase/png/compound00056.png]

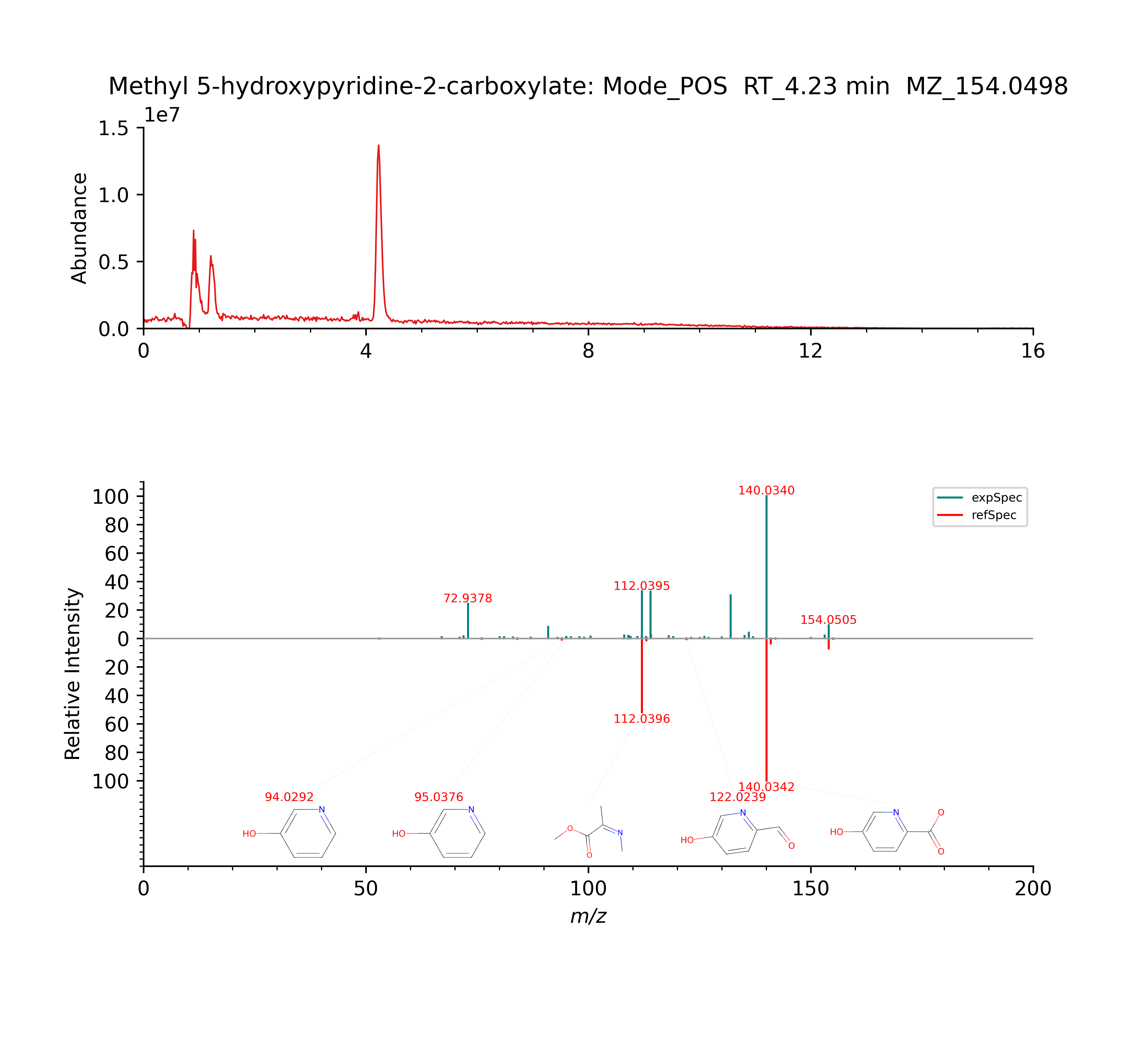

Supplement: Supplementary file 1 [file molecules-29-02840-s001.zip › Supplementary Figure s1/Identification from LuMet-CM datebase/png/compound00057.png]
